# Biodegradable nanoparticles induce cGAS/STING-dependent reprogramming of myeloid cells to promote tumor immunotherapy

**DOI:** 10.3389/fimmu.2022.887649

**Published:** 2022-08-18

**Authors:** Joseph R. Podojil, Andrew C. Cogswell, Ming-Yi Chiang, Valerie Eaton, Igal Ifergan, Tobias Neef, Dan Xu, Khyati A. Meghani, Yanni Yu, Sophia M. Orbach, Tushar Murthy, Michael T. Boyne, Adam Elhofy, Lonnie D. Shea, Joshua J. Meeks, Stephen D. Miller

**Affiliations:** ^1^ Department of Microbiology-Immunology, Northwestern University Feinberg School of Medicine, Chicago, IL, United States; ^2^ Cour Pharmaceutical Development Company, Northbrook, IL, United States; ^3^ Department of Urology, Northwestern University Feinberg School of Medicine, Chicago, IL, United States; ^4^ Immunobiology Center, Northwestern University Feinberg School of Medicine, Chicago, IL, United States; ^5^ Biomedical Engineering, University of Michigan, Ann Arbor, MI, United States

**Keywords:** tumor immunity, cGAS/STING, IL-15, NK cell, nanoparticle

## Abstract

Cancer treatment utilizing infusion therapies to enhance the patient’s own immune response against the tumor have shown significant functionality in a small subpopulation of patients. Additionally, advances have been made in the utilization of nanotechnology for the treatment of disease. We have previously reported the potent effects of 3-4 daily intravenous infusions of immune modifying poly(lactic-co-glycolic acid) (PLGA) nanoparticles (IMPs; named ONP-302) for the amelioration of acute inflammatory diseases by targeting myeloid cells. The present studies describe a novel use for ONP-302, employing an altered dosing scheme to reprogram myeloid cells resulting in significant enhancement of tumor immunity. ONP-302 infusion decreased tumor growth *via* the activation of the cGAS/STING pathway within myeloid cells, and subsequently increased NK cell activation *via* an IL-15-dependent mechanism. Additionally, ONP-302 treatment increased PD-1/PD-L1 expression in the tumor microenvironment, thereby allowing for functionality of anti-PD-1 for treatment in the B16.F10 melanoma tumor model which is normally unresponsive to monotherapy with anti-PD-1. These findings indicate that ONP-302 allows for tumor control *via* reprogramming myeloid cells *via* activation of the STING/IL-15/NK cell mechanism, as well as increasing anti-PD-1 response rates.

## Introduction

Recent advances in the field of cancer immunology have highlighted the importance of the immune system for eliminating tumors. Numerous studies have shown that tumor-infiltrating immune cells such as antigen-presenting cells (APCs) (1), T cells (2, 3), and natural killer (NK) cells (4, 5) play critical roles in tumor control. However, the inflammatory anti-tumor immune response is counteracted by the induction of immune regulatory mechanisms within the tumor microenvironment (TME). These findings have led to the development of immune-targeted therapies, which are aimed at activating anti-tumor immune signaling pathways and enhancing anti-tumor immune function (6–10). While immunotherapies, especially checkpoint inhibitors and CAR-Ts, have revolutionized the treatment of several solid tumors and leukemias, at best response rates remain low at 25%-30% (11), and a portion of patients eventually develop resistance to therapy leading to disease progression and mortality (12). Several factors contribute to low response rates and development of resistance including low immune infiltrates in the tumor microenvironment (13): immune exhaustion due to upregulation of PD-1/PD-L1 on immune cells in the TME (14, 15), lack of antigen presentation by APCs, and absence of tumor antigen-specific immune activation *via* expression of PD-L1, CTLA-4, B7-H4 and other immune inhibitory molecules, and contributions from the tumor stroma that blunt anti-tumor immune responses by acting as physical barriers and actively producing immune inhibitory, pro-angiogenic and pro-tumor factors (16).

Among the inhibitory immune cell types present within the TME, myeloid-derived suppressor cells (MDSCs) and tumor-associated macrophages (TAMs) have been spotlighted as major contributors to tumor immune evasion (1, 17, 18). Both MDSCs and TAMs are derived from immature bone-marrow-derived myeloid precursor cells (19). Studies in both humans and mice have shown that these immature monocyte precursors, as well as CD8+ T cells and natural killer (NK) cells are actively recruited into tumors *via* tumor-derived chemokines (e.g., CCL2/MCP-1, CCL3/MIP-1⍺, and CCL4/MIP-1β) (20). This is a continual process as MDSCs and TAMs within the TME turnover naturally and are replaced by cells from the periphery (21). Once in the TME, monocytic precursors are exposed to chemokines and cytokines in the TME that induce their differentiation into pro-tumor MDSCs and TAMs. Within the TME, MDSCs, and TAMs produce anti-inflammatory factors (e.g., IL-10, TGF-b, and Arginase-1) (18) that promote immune suppression in the TME and pro-tumor factors (e.g., MMP9, growth factors, and cytokines) (22). Collectively, these findings have identified myeloid cells, such as monocytes and macrophages, as attractive targets for cancer immunotherapy.

cGAS/STING is a cytosolic sensor of DNA that binds cytosolic DNA and the downstream adaptor molecule STING (stimulator of interferon genes). Triggering of cGAS/STING leads to the concurrent activation of IRF3 (Interferon Regulatory Factor 3) and NF-kB (Nuclear factor kappa B) leading to the production of Type I interferons (23, 24) and the downstream broad transcriptional program of ISGs (Interferon-Stimulated genes). cGAS/STING is essential for control of both bacterial (25) and viral (26) pathogens, and cGAS/STING can also be triggered by host genomic DNA in the presence of tumor cells (27). Consequently, the use of cGAS/STING pathway agonists has been suggested to be a potential therapeutic for the treatment of cancer (28). DNA damage *via* ionizing radiation (29) and certain chemotherapies (30) triggers cGAS/STING and the production of pro-inflammatory cytokines necessary for immune cell recruitment and tumor clearance. Currently several pre-clinical models in mice have shown promising results utilizing various cGAS/STING agonists to enhance tumor clearance (28).

NK cells recognize danger-associated ligands in the context of cells that lack MHC-I expression, and NK cells are essential for immune-mediated control of tumor growth and metastasis (5, 20). Upon activation by cytokines including IL-15, IL-12, IL-18, and IL-2, NK cells can directly lyse target cells (31). In addition, NK cells rapidly produce cytokines and chemokines, such as TNF-⍺, IFN-ɣ, and CCL5 to promote activation of the adaptive immune response (32). NK cell activity has been shown to inversely correlate with tumor progression (4, 13). Existing pre-clinical tumor models have tried to leverage the anti-tumor function of NK cells *via* the use of NK-CAR cells to detect specific cancer associated antigens (33), Bi- and Tri-specific Killer Engagement (BiKE & TriKE respectively) antibodies (34), as well as the infusion of NK cells of various sources ex vivo expanded *via* cytokines (9).

We have previously reported that immune modifying carboxylated 500 nM diameter poly(lactic-co-glycolic acid) (PLGA) nanoparticles (IMPs; named ONP-302) designed for uptake by myeloid cells (e.g., monocytes, macrophages, and neutrophils) *via* scavenger receptors, particularly the macrophage receptor with collagenous structure (MARCO), demonstrated beneficial therapeutic immune modulatory effects in multiple models of acute and chronic inflammation (35–37). ONP-302 particles, free from additional active therapeutic agents, are preferentially taken up by monocytes and macrophages which undergo apoptosis in the spleen and liver leading to inhibition of inflammatory monocyte trafficking into sites of inflammation and resolution of inflammation (35, 36). We initially hypothesized that targeting immature myeloid cells with ONP-302 would be an effective tool for the treatment of cancers by disrupting the influx of immature myeloid precursors of MDSCs and TAMs from the periphery into the TME thus diminishing the immunosuppressive microenvironment within the tumor. Additionally, we speculated that ONP-302 treatment would provide enhanced efficacy when co-administered with immunotherapies targeted at inducing T cell and NK cell activation. Here, we report that ONP-302 particle treatment inhibits the growth of tumors that are either responsive or unresponsive to checkpoint immunotherapy. Additionally, ONP-302 treatment enhanced the sensitivity of a normally checkpoint-resistant tumor to later anti-PD-1 therapy. Lastly, we demonstrate that ONP-302-induced regulation of tumor growth is initiated by uptake of the nanoparticles by myeloid cells which induces the activation of the cGAS/STING pathway. The increased activation of the cGAS/STING pathway resulted in the production of IL-15, increased activation of NK and CD8+ T cells, and an increased level of tumor cell necrosis.

## Materials and methods

### ONP-302 fabrication

ONP-302 nanoparticles were composed of poly(lactic-co-glycolic acid) (PLGA) (Lactel^®^, Durect Corporation) using a double emulsion technique. Briefly, a primary emulsion containing PLGA dissolved in an organic solvent was homogenized. The primary emulsion was then combined with a secondary emulsion containing a proprietary blend of surfactants, stabilizers, and organic solvents and homogenized. Particles were hardened by evaporating the solvents from the solution overnight with stirring at room temperature in a fume hood. Hardened particles were washed using a Tangential Flow Filtration (TFF) System. Particle physiochemical properties were characterized using Dynamic Light Scattering (DLS, Malvern) and scanning electron microscopy.

### Cell culture

MC38 (Kerafast) and B16.F10 (ATCC) mouse tumor cells were maintained in monolayer culture in DMEM (ATCC^®^ 30-2002™) supplemented with 10% fetal calf serum (FCS) (Gemini) at 37°C in a tissue-culture incubator with 5% CO2. Cells at low passage and in exponential growth phase were harvested using 0.25% Trypsin-EDTA (Sigma) for injection into mice. A mouse melanoma cell line (B16.F10), mouse colon adenocarcinoma (MC38) and mouse Macrophage (RAW264.7) cells were purchased from American Type Culture Collection. Mouse Macrophages lacking cGAS were purchased from *In vivo* Gen (San Diego, CA).

### Murine tumor models

Female C57BL/6, STING gt/gt and, RAG1-/- mice (8-10 weeks of age) were purchased from Jackson Labs. All procedures were reviewed and approved by the Northwestern University Institutional Animal Care and Use Committee (IACUC) and conducted in accordance with the regulations of the Association for Assessment and Accreditation of Laboratory Animal Care (AAALAC). Mice were housed in the animal care facility for one-week prior to manipulation. MC38 or B16.F10 tumor cells (5x10^5^ cells/mouse) were injected into the shaved right flank of isofluorane anesthetized mice. Tumor growth was measured routinely using standard calipers and tumor volumes were calculated using the formula: Tumor volume (mm3) = 1/2(length) × (width2). Animals received saline (i.v.), ONP-302 (i.v.), or anti-PD1 (Rat IgG2a, Clone RMP1-14) (i.p.) treatments after palpable tumor formation (50-100 μm3). Saline and ONP-302 treatments were administered once every three days, and anti-PD1 (100 µg/dose) treatment was administered every two days. Anti-NK1.1 (clone PK136; BioXCell), anti-IL-15 (clone AIO.3; BioXCell), IgG isotype control antibody (clone C1.18.4; BioXCell) were administered, when indicated, *via* i.p. injections.

### Single cell RNASeq analysis

Female C57BL/6 (8-10 weeks of age) were randomized into treatment groups, and at 24 hours after the last dose, spleens were collected and processed into single cell suspensions. Cells were re-suspended in 0.04% BSA in PBS solution and loaded into a 10X Chromium platform for Gel Bead-In Emulsion (GEM) generation and barcoding. Samples were processed using the Chromium Single GEM Single Cell 3’ reagent kit v3.1, following manufacturer’s instructions. Libraries were pooled and sequenced on an Illumina HiSeq as 150-bp paired-end reads. Reads were mapped to the mouse genome (mm10) using CellRanger (10x Genomics). The outputs were used in the Seurat pipeline as previously described (38). Upon merging the Seurat objects from each treatment condition, mitochondrial genes (starting with “mt-”) were regressed out of the data set. Differentially expressed genes were identified within the Seurat object using the FindMarkers command. In order to identify enriched pathways, these genes were converted to their human equivalents with biomaRt (39). Gene lists were ranked by fold change between ONP-302 treated samples and the saline control. Enriched pathways were then measured by Gene Set Enrichment Analysis (GSEA) using a pre-ranked gene list (40). Gene sets were sampled from the hallmark, BIOCARTA, KEGG, REACTOME, PID, and gene ontology (GO) databases obtained from the Molecular Signatures Database (MSigDB) collections.

### Generation of bone marrow derived myeloid cells

Bone marrow was harvested from 8-10 week-old C57BL/6J and STING gt/gt mice. RBC cells were lysed and cells were plated at a density of 10^6^/mL at 37°C in RPMI-1640 containing 2 mM L-glutamine, 10% (v/v) heat inactivated fetal bovine serum, 100 U/mL penicillin, 100 μg/mL streptomycin and 25 ng/mL murine GM-CSF (PeproTech). On days 3 and 5 cells were lifted from the plates using gentle scraping and counted alongside cells in suspension and replated at 10^6^/mL. On Day 7 cells were harvested for *in vitro* experiments. Cells were cultured at a density of 0.5x10^6^/mL in the appropriate medium in a 12 well plate. Twenty-four hours post culture initiation, the medium was replaced with fresh medium or medium containing various combinations of: 2.5 µg/mL ONP-302, 8 µg/mL RU521 (STING inhibitor; *In vivo*gen), 20 µM Z-VAD-FMK (Caspase inhibitor; *In vivo*gen), 400 ng/mL G3-YSD (cDNA; *In vivo*gen), and/or 1 μg/mL LPS. After 18-24 hours, cell supernatants were collected and frozen at -20°C for use in Milliplex assays.

### Tissue processing and flow cytometry

Peripheral blood was collected from Nembutal anesthetized mice *via* cardiac puncture with an EDTA rinsed syringe. Approximately 350-500 mL of whole-blood was collected. RBCs were lysed using ammonium chloride prior to processing for flow cytometry. The spleens were processed *via* tissue disruption, and RBCs were lysed using ammonium chloride prior to processing for flow cytometry. For the tumor leukocytes, single cell suspensions were prepared by mincing the tumor tissue in 2 mL of Accutase (MilliPore) plus 1 mg/mL collagenase, and the samples were incubated at 37°C for 30 min. Following the enzyme digestion, the tumor samples were disrupted with a 100 µm cell strainer, the cell strainer washed 2x with 10 mL of HBSS+ 5% FCS, and the cells pelleted. Cells were washed in PBS, stained with LIVE/DEAD^®^ Fixable Aqua Dead Cell Stain (Life Technologies; Grand Island, NY), blocked with anti-CD16/32 (ThermoFisher Scientific), and then stained with the indicated antibodies. 10^6^ viable cells were analyzed per individual sample using a BD Celesta (BD Bioscience), and the data analyzed using FloJo Version 9.5.2 software (Tree Star, Inc.; Ashland, OR). The specific antibodies used are presented in **Supplementary Table 1**.

### Cytokine analysis

Peripheral blood was collected as before, and plasma was fractionated by centrifugation and the indicated analytes were assayed using a Millipore Luminex Multiplex Assay Kit. MCYTOMAG-70K | Milliplex MAP Mouse Cytokine Chemokines kits were purchased from EMD Millipore. Cell supernatants or sera were diluted to detectable concentrations of cytokines and then analyzed according to the manufacturer’s instructions.

### Histology

Mouse tumors were harvested 24 hours after treatment with the 3rd dose of ONP-302. Tumors were immediately fixed in 10% formalin and embedded in paraffin. Four-micron thick sections were used for IHC staining for CD8 (1:1000, #D4W2Z, Cell Signaling) and NK1.1 (1:1000, #E6Y9G, Cell Signaling). IHC was performed using a Dako Autostainer Plus instrument (Dako, CO, USA), and anti-rabbit Dako EnVision+System-HRP (Dako).

### Statistical analyses

Comparisons of the percentage of animals showing clinical disease were analyzed by X2 using Fisher’s exact probability, and two-way ANOVA with a Bonferroni post-test was used to determine statistical differences between mean treatment groups values for cell populations, secreted cytokines, and tumor volumes. Single comparisons of two means were analyzed by Student’s t-test.

## Results

### ONP-302 induces the activation of cGAS/STING pathway in myeloid cells

**Figure 1:**
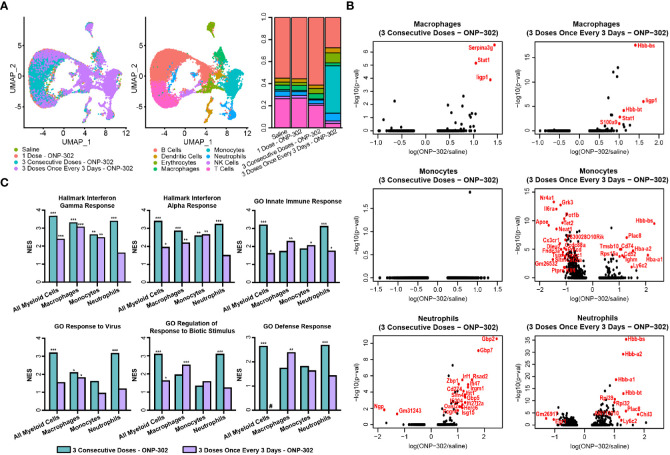
Single cell RNASeq analysis of ONP-302-induced gene expression. Naïve female C57BL/6 mice (n = 3 per group) were treated with saline or ONP-302 (1.0 mg/dose in 200 μL of saline) either once, three days consecutively (days 0, 1, and 2), or every three days (Days 0, 3, and 6) *via* i.v. injection. Twenty-four hours after the last injection, spleens were collected to assess the transcriptome *via* single cell RNASeq. **(A)** The distribution of cells within the spleen in response to saline and each of the ONP-302 dosing schemes is compared. **(B)** The ONP-302-induced changes in gene expression by macrophages, monocytes, and neutrophils is compared. Red genes have a log fold change > 1 and a *p*-value < 0.05. **(C)** The signaling pathways induced by the ONP-302 treatment relative to saline treatment alone as measured through Gene Set Enrichment Analysis (GSEA), NES = normalized enrichment score, *q < 0.1, **q < 0.01, ***q < 0.001, # pathway was not enriched in this sample.

We have reported the utility of carboxylated immune modifying poly(lactic-co-glycolic acid) (PLGA) nanoparticles (IMPs; named ONP-302) for the treatment of multiple disease states including targeting inflammatory myeloid cells for the amelioration of tissue damage in multiple acute inflammatory diseases (35, 36). To better understand the cellular and molecular mechanisms underlying the potent immunoregulatory effects of PLGA nanoparticles, we performed single cell RNASeq analysis on splenic leukocytes from naïve C57BL/6 mice injected i.v. with saline, one dose of ONP-302, three doses of ONP-302 on three consecutive days, or three doses of ONP-302 administered every three days. Treatment with three doses of ONP-302 (1 mg/dose) either on consecutive days or every three days induced significant shifts in cell population clustering as compared to saline treatment alone. The treatment of mice on three consecutive days induced an increase in the proportion of B cells, while treating mice every 3 days induced an increase in monocytes, macrophages, and neutrophils (**Figure 1A** and **Supplementary Figure 1**). To determine the transcripts and pathways altered by three doses of ONP-302 treatment as compared to saline treatment alone, differential transcript expression was analyzed for macrophages, monocytes, and neutrophils. The present data show that three doses of ONP-302 induced significant alterations in transcripts expressed by macrophages, monocytes, and neutrophils (**Figure 1B**). When the differentially expressed transcripts from the immune cell population cluster analysis were further analyzed, significant increases in transcripts associated with IFN-γ, IFN-α, the activation of the innate immune response, and cellular pathways associated with anti-viral immune responses were identified (**Figure 1C**, and **Supplementary Tables 2–9**). The present findings suggest that treatment of C57BL/6 mice with three doses of ONP-302 every three days activates cellular pathways within splenic myeloid cells that would be advantageous for immune cell control of tumor growth. The activation of these pathways are known to promote greater activation of both CD8+ T cells and NK cells (41, 42), which allows for increased tumor cell killing (42, 43).

**Figure 2:**
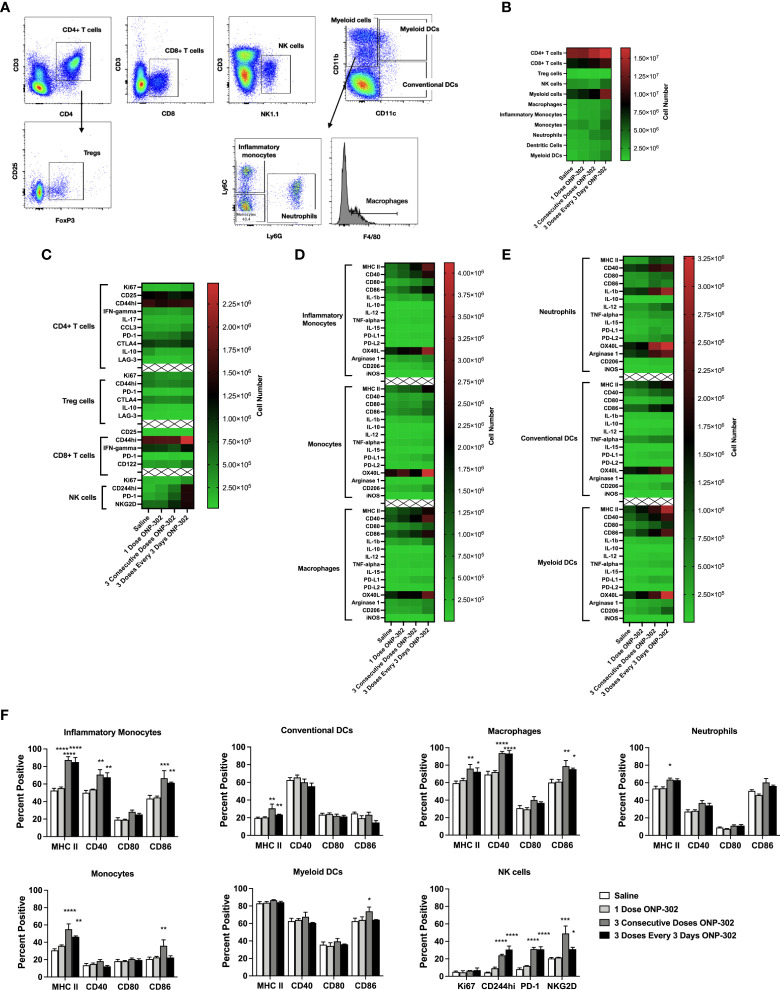
ONP-302 treatment increases the number and frequency of activated immune cells. Naïve female C57BL/6 mice (n = 3 per group) were treated similarly to **Figure 1**. Twenty-four hours after the last injection, spleens were collected to assess the immune cell populations present. A gating schematic detailing how cells were identified is shown **(A)**. The total number of cells **(B)**, as well as the phenotypes of CD4^+^ T cells, CD8^+^ T cells, NK cells **(C)**, monocytes and macrophages **(D)**, as well as other myeloid cells **(E)** are shown as heatmaps. The frequency of specific populations expressing cellular markers of activation after treatment is shown in **(F)** The data are presented as the mean percentage of cells +/- S.E.M. One representative experiment of two is presented. Asterisks (*, **, ***, ****) indicate a statistically significant difference as compared to saline treated mice, p < 0.05, < 0.01, < 0.001, and < 0.0001 respectively.

To further confirm and validate the results of the scRNAseq, using the gating schema shown in **Figure 2A**, we assessed both the number (**Figure 2B**) and phenotype of CD4+ T cells, CD8+ T cells and NK cells (**Figure 2C**), monocytes and macrophages (**Figure 2D**), as well as other myeloid populations (**Figure 2E**). Of note, repeated dosing of ONP-302 every 72 hours significantly upregulated costimulatory molecules such as CD40 and CD86 on myeloid populations as well as inducing a more activated phenotype on NK cells which significantly upregulated CD244 (2B4), PD-1, and NKG2D (**Figure 2F**). Previously published data show that PLGA nanoparticles are phagocytosed by myeloid cells within the spleen (44, 45). While the phagocytosis of these nanoparticles by myeloid cells has been shown to have therapeutic benefit, the induced downstream molecular and cellular mechanism are still in the process of being fully identified. These present data indicate that multiple treatments of ONP-302 lead to an increase in pro-inflammatory myeloid cell signatures, which in increased NK cell activation markers and possible function.

**Figure 3:**
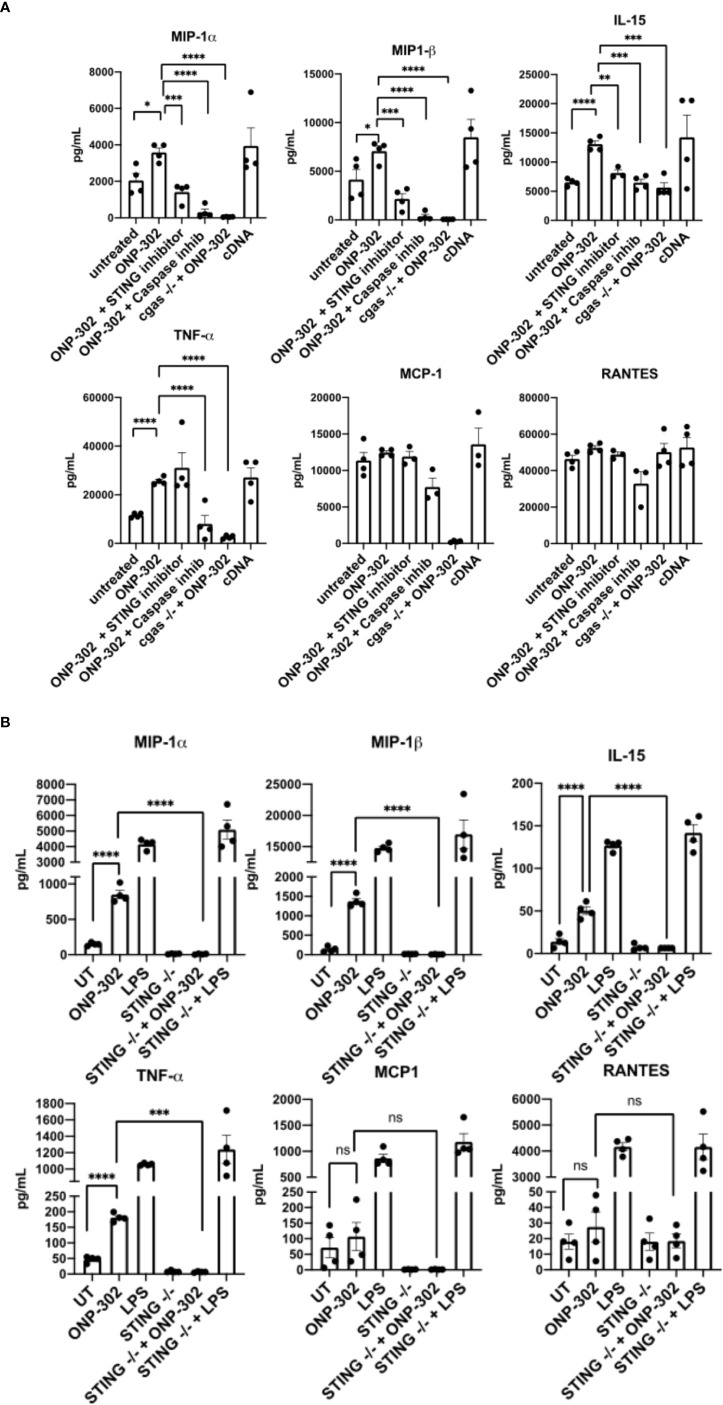
Uptake of ONP-302 by RAW cells **(A)** and bone marrow-derived macrophages (BMDM) **(B)** induces cytokine production *via* the *cGAS/*STING pathway. RAW cells possessing or lacking the *cGAS* gene (cGAS -/-) were treated with ONP-302 for 24 hours in the presence or absence of a STING inhibitor (RU.521) or a Caspase inhibitor (ZVAD-FMK) and supernatants were collected to measure the level of chemokines and cytokines. BMDMs from either wt C57BL/6 mice or STING -/- mice were treated with ONP-302 for 24 hours and supernatants were collected to measure the level of chemokines and cytokines. Each dot represents a unique well of cells tested and the bar represents the mean ± S.E.M. Asterisks (*, **, ***, ****) indicate a statistically significant difference as compared to untreated cells, p < 0.05, < 0.01, < 0.001, and < 0.0001 respectively. NS, not significant.

A powerful inducer of Interferon Stimulatory Genes (ISGs)/pro-inflammatory signals in myeloid cells is cGAS/STING signaling (23). To test whether cGAS/STING signaling was responsible for the transcriptional signatures identified following ONP-302 treatment, RAW cells were cultured in the presence of ONP-302 for 24 hours. The present data show that ONP-302 treatment significantly increased the level of TNF-α, MIP-1α, MIP-1β, and IL-15 secreted. To determine the potential role of the STING pathway in cytokine production, several approaches were employed (**Figure 3**). We found that wildtype RAW cells cultured in the presence the STING inhibitor, RU.521 (46), or cGAS-deficient RAW cells stimulated with ONP-302 failed to upregulate expression of TNF-α, MIP-1α, MIP-1β and IL-15 (**Figure 3A**). In addition, GM-CSF driven bone marrow-derived myeloid cells from wildtype vs. STING-/- mice were cultured in the presence of 25 ng/mL GM-CSF for 7 days at which time the cell phenotypes were analyzed by flow (**Supplementary Figure 2**). The remaining cells were cultured in the presence of LPS (1.0 μg/mL) or ONP-302 (2.5 μg/mL) for 24 hours and the levels of secreted cytokines/chemokines analyzed. Lipopolysaccharide (LPS)-induced the secretion of TNF-α, MIP-1α, MIP-1β, and IL-15 by both wildtype and STING-/- myeloid cells, while cytokine secretion in response to ONP-302 was lost in the absence of STING expression (**Figure 3B**). Taken together, these data show that phagocytosis of ONP-302 by myeloid cells results in activation of the cGAS/STING pathway leading to secretion of TNF-α, MIP-1α, MIP-1β, and IL-15.

### ONP-302 treatment inhibits tumor growth

**Figure 4:**
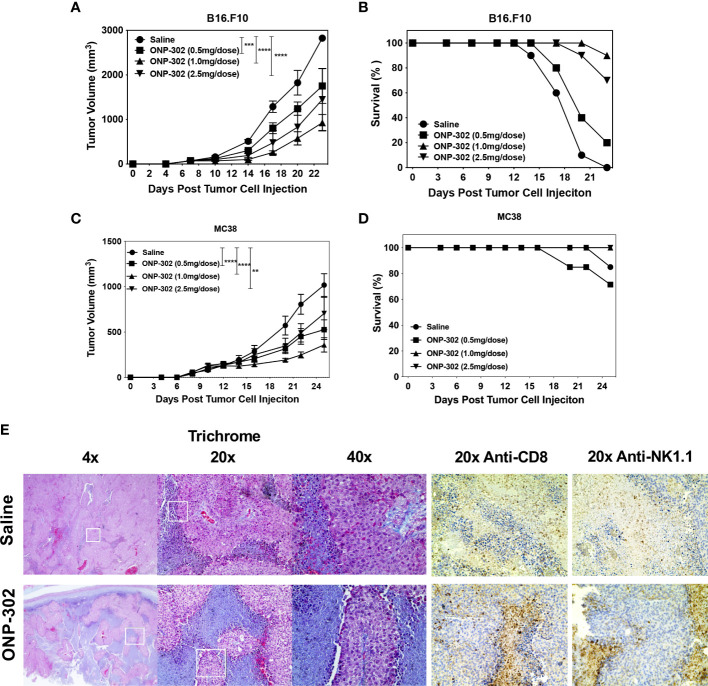
ONP-302 treatment decreases B16.F10 and MC38 tumor growth and increases tumor necrosis. Naïve female C57BL/6 mice (n = 7-10 per treatment group) were injected s.c. was B16.F10 **(A, B)** or MC38 **(C, D)** tumor cells. When the tumors were 50-100 mm^3^ in size, mice were randomized and treated every three days with saline or ONP-302 (0.5, 1.0, or 2.5 mg/dose in 200 μL of saline) *via* i.v. injection. Tumor volumes were measured on the indicted days, and the data are presented as the mean tumor volume ± S.E.M **(A, C)**. The overall survival of the mice within each treatment group is also presented **(B, D)**. Mice were euthanized when the tumor volume exceeded 1500 mm^3^. Representative micrographs of B16.F10 tumors collected from saline and ONP-302 (1.0 mg/dose) treated mice were stained for trichrome, anti-CD8, and anti-NK1.1 **(E)**. One representative experiment of two is presented. Asterisks (**, ***, ****) indicate a statistically significant difference as compared to untreated cells, p < 0.01, < 0.001, and < 0.0001 respectively.

Based on the potent modulatory effects of ONP-302 on myeloid cells both *in vitro* and *in vivo*, we evaluated the potential of ONP-302 to alter the TME to enhance tumor immunity using the B16.F10 melanoma and MC38 colon adenocarcinoma tumor models. These two models represent distinct tumor types derived from different anatomical sites, and the tumors vary in the level of immune cell infiltrate as well as the response rate to immunotherapies, such as anti-PD-1 checkpoint inhibitors. Female C57BL/6 mice were injected with B16.F10 or MC38 tumor cells (5x10^5^ cells/mouse) s.c. into the right flank. When the tumors reached an average volume of 50-100mm3 (approximately 7 days), the mice were randomized into four treatment groups (0, 0.5, 1.0, or 2.5 mg/dose of ONP-302 in 200 mL of saline *via* i.v. injection). The mice were treated every three days and the tumor volumes were measured on the indicated days. Treatment with ONP-302 significantly decreased tumor growth and prolonged the survival of B6 mice implanted with either B16.F10 (**Figures 4A, B**) or MC38 (**Figures 4C, D**) cells in a dose-dependent manner. Twenty-four hours after the third dose of saline or ONP-302 (1.0 mg/dose) tumors were harvested from B16.F10 tumor-bearing mice for histopathological analysis. B16.F10 tumors from ONP-302 treated mice showed increased necrosis and markedly enhanced numbers of both CD8+ T cells and NK cells as compared to B16.F10 tumors from saline treated mice (**Figure 4E**). ONP-302 treatment thus leads to a dose-dependent inhibition of tumor growth associated with infiltration of increased numbers of CD8+ T cells and NK cells and enhanced necrosis.

### ONP-302 induces an activated NK cell phenotype in spleen and tumor

**Figure 5:**
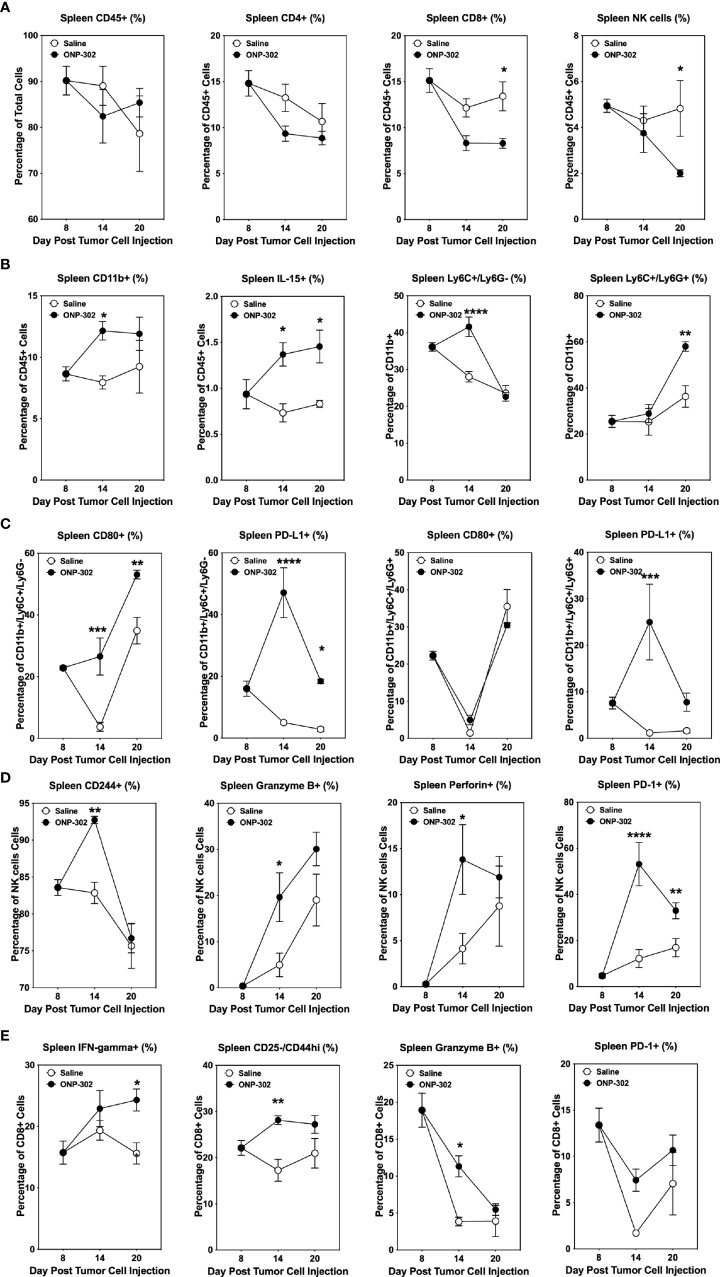
ONP-302 treatment alters immune cell populations within the spleens of B16.F10 tumor-bearing mice over time. Naïve female C57BL/6 mice (n = 9 per treatment group) were injected s.c. with B16.F10 tumor cells. When the tumors were 50-100 mm^3^ in size, mice were randomized and treated every three days with saline or ONP-302 (1.0 mg/dose in 200 μL of saline) *via* i.v. injection. On Day 8 (prior to the first treatment), Day 14 (24 hours after the third treatment), and Day 20 (24 hours after the fifth treatment), spleens were collected to determine the percentage of various immune cell populations. The general lineage markers for CD45^hi^, CD4^+^ T cells, CD8^+^ T cells, NK cells, CD11b^+^ cells, Ly6C^-^/Ly6G^+^, Ly6C^+^/Ly6G^+^, and IL-15 cells are presented **(A, B)**. The specific effector phenotypes of the myeloid **(C)**, NK **(D)**, and CD8^+^ T cells **(E)** were determined by intracellular staining. The data are presented as the mean percentage of cells ± S.E.M. One representative experiment of two is presented. Asterisks (*, **, ***, ****) indicate a statistically significant difference as compared to saline treated mice, p < 0.05, < 0.01, < 0.001, and < 0.0001 respectively.

**Figure 6:**
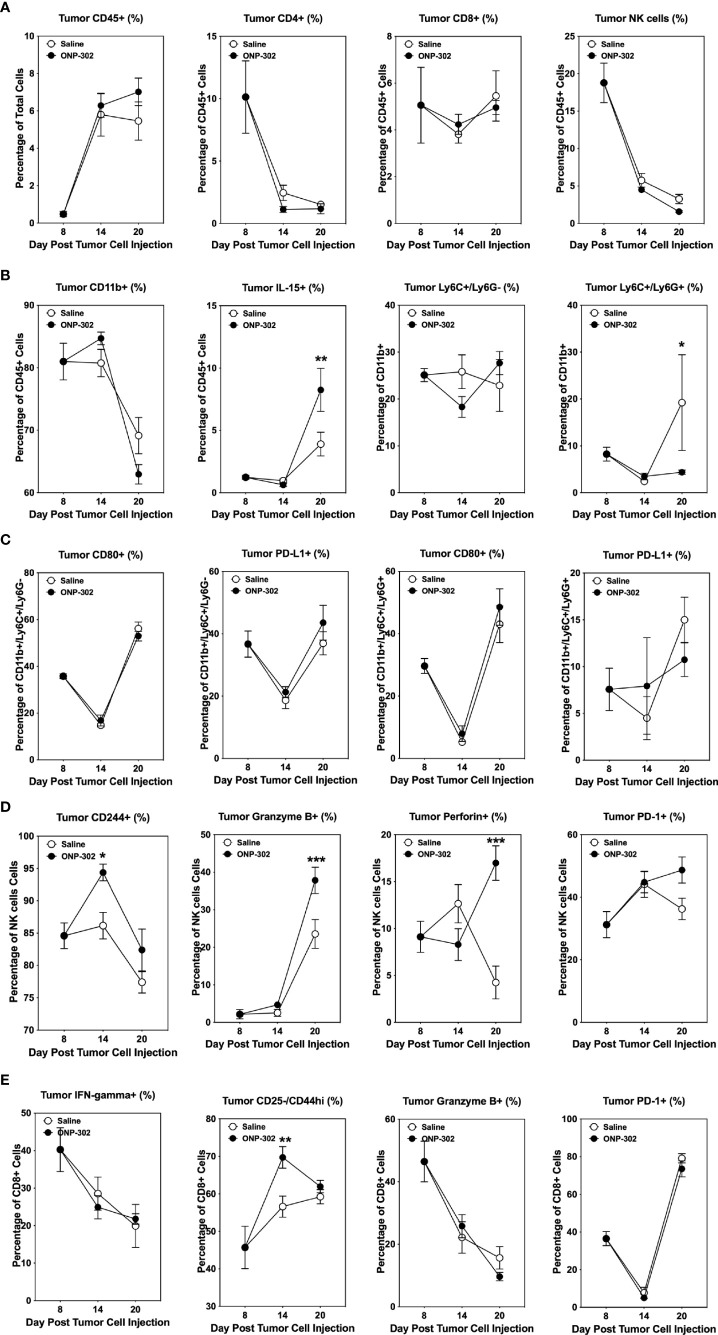
ONP-302 treatment alters immune cell populations within the tumor microenvironment (TME) of B16.F10 tumor-bearing mice over time. Naïve female C57BL/6 mice (n = 9 per treatment group) were injected s.c. with B16.F10 tumor cells. When the tumors were 50-100 mm^3^ in size, mice were randomized and treated every three days with saline or ONP-302 (1.0 mg/dose in 200 μL of saline) *via* i.v. injection. On Day 8 (prior to the first treatment), Day 14 (24 hours after the third treatment), and Day 20 (24 hours after the fifth treatment), tumors were collected to determine the percentage of various immune cell populations present. The general lineage markers for CD45^hi^, CD4^+^ T cells, CD8^+^ T cells, NK cells, CD11b^+^ cells, Ly6C^-^/Ly6G^+^, Ly6C^+^/Ly6G^+^, and IL-15 cells are presented **(A, B)**. The specific effector phenotypes of the myeloid **(C)**, NK **(D)**, and CD8^+^ T cells **(E)** were determined by intracellular staining. The data are presented as the mean percentage of cells ± S.E.M. One representative experiment of two is presented. Asterisks (*, **, ***) indicate a statistically significant difference as compared to saline treated mice, p < 0.05, < 0.01, and < 0.001 respectively.

The temporal effects of ONP-302 treatment on immune cell phenotypes within the spleen, blood, and tumor was assessed. B16.F10 tumor cells were implanted in C57BL/6 mice and on Day 8 the spleens, tumors, and PBMCs from representative mice were collected. The remainder of the mice received either saline or ONP-302 treatment every three days and tumors were excised either on Day 14 or Day 20 (representing 24 hours after the third and fifth doses, respectively). There was not a significant difference between the percentage of total CD45hi or CD4+ T cells within both the spleen and tumor over time in saline vs. ONP-302-treated mice (**Figure 5A**, **6A**). While ONP-302 treatment decreased the percentage of total CD8+ T cells and NK cells within the spleen, the percentages of total CD8+ T cells and NK cells within the tumor were not altered. In contrast, ONP-302 treatment increased the percentage of total myeloid cells (CD11b+) within the spleen. Of note, ONP-302 treatment increased the percentage of neutrophils present within the spleen (**Figure 5B**), while significantly decreasing the percentage of these cells present within the tumor (**Figure 6B**). Additionally, ONP-302 treatment significantly increased the percentage of CD80+ and PD-L1+ monocytes/macrophages (CD11b+/Ly6C+/Ly6G-) within the spleen (**Figure 5C**), and significantly increased the percentage of PD-L1+ neutrophils (CD11b+/Ly6C+/Ly6G+) within the spleen (**Figure 5C**). In contrast, the percentage of CD80+ or PD-L1+ monocytes/macrophages and neutrophils did not change within the tumor (**Figure 6C**). These findings suggest that ONP-302 decreased the percentage of PMN-MDSCs within the tumor. As a correlate to increased immune cell activation within the tumor following ONP-302 treatment, the percentage of IL-15+ cells increased within both the spleen and tumor (**Figures 5B**, **6B**) over time, and there was an increase in the percentage of activated NK cells (CD244+, granzyme B+, perforin+, and PD-1+) at both sites (**Figures 5D**, **6D**). Similarly, the percentage of activated CD44hi CD8+ T cells increased within both the spleen and tumor, while the percentage of IFN-ɣ+ and granzyme B+ CD8+ T cells increased within the spleen (**Figures 5E**, **6E**). The ONP-302-induced alterations in immune cell populations within the spleen and tumor are not limited to the B16.F10 tumor model, as similar alterations were found in ONP-302-treated MC38 tumor-bearing mice (**Supplementary Figures 3**, **4**). Additionally, the percentage of activated CD8+ T cells, as well as total and activated NK cells within the blood increased over time in both the B16.F10 and MC38 tumor-bearing mice (**Supplementary Figure 5**). These findings indicate that ONP-302 treatment activated and mobilized CD8+ T cells and NK cells in tumor-bearing mice. Collectively, these results support the hypothesis that ONP-302 treatment increases the percentage of myeloid cells within the spleen, while decreasing their percentage within the tumor. Secondly, these findings suggest that the same STING pathway-associated cellular mechanisms may be involved in ONP-302-induced modulation of tumor growth.

### Adaptive immune and NK cells, IL-15, and STING are required for ONP-302 function

**Figure 7:**
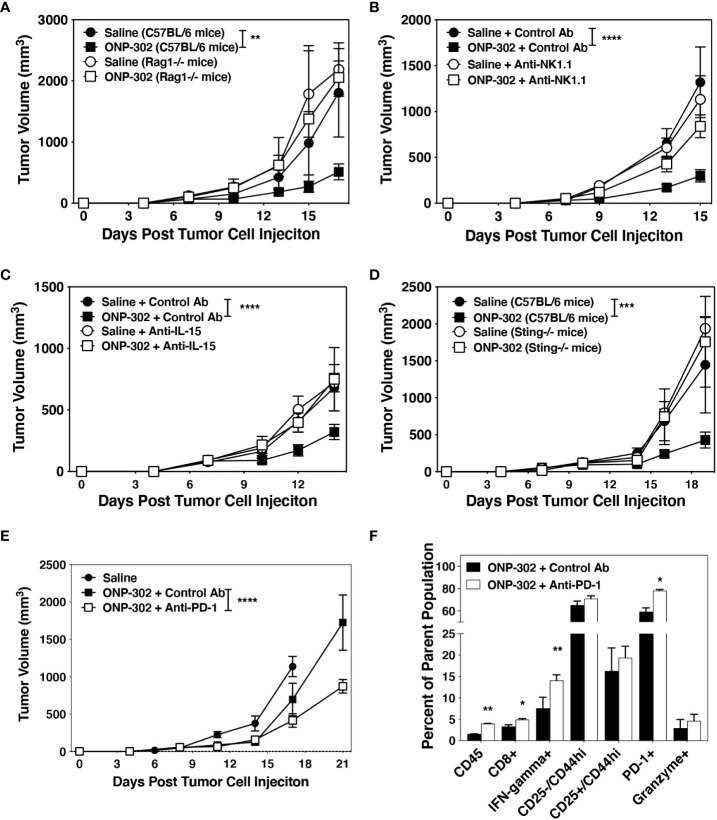
Effective ONP-302 treatment requires both adaptive immune and NK cells activated by STING and IL-15, and enhances tumor response to anti-PD-1 immunotherapy. Naïve female C57BL/6, Rag-/- **(A)**, or Sting-/- **(D)** mice were injected s.c. was B16.F10 tumor cells. When the tumors were 50-100 mm^3^ in size, mice were randomized and treated every three days with saline or ONP-302 (1.0 mg/dose in 200 μL of saline) *via* i.v. injection. To deplete NK cells, mice were treated with a species and isotype matched control antibody or anti-NK1.1 (100 μg/dose) one day prior to each saline of ONP-302 treatment (n = 5 per treatment group) **(B)**. To block IL-15, mice were treated with a species and isotype matched control antibody or anti-IL-15 (100 μg/dose) one day prior to each saline of ONP-302 treatment (n=5 per treatment group) **(C)**. After the fifth dose of ONP-302, mice received three doses of either a control antibody or anti-PD-1 (100 μg/dose) given every three days. The tumor volumes were measured on the indicted days, and the data **(E)** are presented as the mean tumor volume ± S.E.M. On Day 25 of the disease course, tumors were collected and the percentage of CD45^hi^ cells from the total cells, the percentage of CD8^+^ T cells from the CD45^hi^ cells, and the percentage of CD8^+^ T cells expressing IFN-γ, CD25, CD44, PD-1 and granzyme was assessed **(F)**. The data are presented as the mean percentage of the respective parent population of cells. One representative experiment of two is presented. Asterisks (*, **, ***, ****) indicate a statistically significant difference as compared to saline treated mice, p < 0.05, < 0.01, < 0.001, and < 0.0001 respectively.

The above findings suggested that the *in vivo* ONP-302-induced reduction of tumor growth may be dependent on the enhanced activation of NK cells and/or CD8+ T cells perhaps secondary to the induction of IL-15. To test this hypothesis, we first demonstrated that the protective effects of ONP-302 were dependent on a functional adaptive immune system as no delay in tumor growth was seen in treated Rag1-/- mice (**Figure 7A**). To examine the role of NK cells, wildtype C57BL/6 mice were implanted with B16.F10 or MC38 tumor cells and when the average tumor volume reached 50-100 mm3, mice were randomized into four groups and treated with saline or ONP-302 in the presence of either a species and isotype matched Control Ab or anti-NK1.1 monoclonal antibody. The present data show that NK cells are required for the ONP-302-induced decrease in B16.F10 growth as anti-NK1.1 treatment restored tumor growth to control levels (**Figure 7B** and **Supplementary Figure 6**). A crucial role for IL-15 was demonstrated by the ability of anti-IL-15 monoclonal antibody treatment to alleviate ONP-302-induced delay of tumor growth (**Figure 7C**). As IL-15 plays a critical role in the activation of both CD8+ T cells and NK cells, this result is consistent with a functional contribution of both the adaptive immune system (**Figure 7A**) and NK cells (**Figure 7C** and **Supplementary Figure 6**) in ONP-302-induced modulation of tumor growth. To test the potential requirement for the activation of the cGAS/STING pathway, which leads to IL-15 production in myeloid cells which have taken up the nanoparticles *in vitro* (**Figure 3**), in ONP-302-induced regulation of tumor growth, we examined B16.F10 tumor growth in STING-deficient C57BL/6 mice. ONP-302 treatment was unable to decrease tumor growth in STING-/- mice (**Figure 7D**). This data is thus consistent with the hypothesis that myeloid cell production of IL-15 secondary to cGAS/STING activation induced upon ingestion of ONP-302 promotes enhanced NK and CD8+ T cell function which regulate tumor growth.

### ONP-302 treatment of B16.F10 tumor-bearing mice enhances response to anti-PD-1 therapy

We have shown that ONP-302 treatment significantly decreased tumor growth while increasing the expression of PD-1/PD-L1 in the TME (**Figure 6** and **Supplementary Figure 4**). While the B16.F10 tumor model in C57BL/6 mice is not responsive to anti-PD-1 treatment (47), based on the temporal increase in PD-1 expression following ONP-302 treatment (**Figures 5**, **6**), we asked if prior ONP-302 treatment would enhance response to later anti-PD-1 immunotherapy in B16.F10 tumor-bearing mice. C57BL/6 mice bearing B16.F10 tumors were treated with either saline or ONP-302 (1.0 mg/dose; five doses given every three days). Twenty-four hours after the last dose, the ONP-302 treated mice were randomized into two treatments receiving either a species and isotype matched control antibody or anti-PD-1 (100 μg/dose injected i.p. every three days). Tumor volumes were measured on the indicated days, and 24 hours after the last Control Ab or anti-PD-1 treatment, the B16.F10 tumors were collected and the presence of CD8+ T cells analyzed by flow cytometry. ONP-302 pre-treatment led to a significant decrease in tumor growth in response to anti-PD-1 therapy (**Figure 7E**) concomitant with a significant increase in the percentage of CD8+ T cells that were IFN-ɣ+ and PD-1+ within the tumors (**Figure 7F**). These findings indicate that ONP-302 treatment may promote effective anti-PD-1 treatment in tumor types unresponsive to PD-1 monotherapy.

## Discussion

Nanoparticle-based cancer therapies have been under development for several decades with efforts primarily focused on the design of nanoparticle carrier systems for enhanced pharmacokinetics, improving the delivery of therapeutic agents to tumors, and reducing toxic side-effects of chemotherapeutics (48). The success of nanoformulated chemotherapeutics with reduced toxicity (Nab Paclitaxel) engendered the development of next-generation nanotherapeutics focused on drug delivery into tumors. The biocompatibility, tunable physiochemical properties, ease of functionalization, and ability to overcome biological barriers of biodegradable polymers made them an attractive option for use in the manufacture of these next generation nanocarrier delivery systems. Additionally, functionalization of nanocarrier surfaces with tumor targeting agents such as antibodies, proteins, and peptides have been described for tumor-specific delivery of anti-tumor agents. However, despite high initial promise, next generation nanocarrier systems have been largely unsuccessful in the clinic. For example, despite surface functionalization with tumor-targeting agents, only 2-5% of systemically administered nanocarriers make their way into tumors resulting in poor pharmacodynamics (48).

Here we demonstrate that i.v. infusion of small (400 to 500 nm), negatively charged, biodegradable carboxylated immune modifying poly(lactic-co-glycolic acid) (PLGA) nanoparticles (IMPs; named ONP-302) significantly reduces tumor growth in both checkpoint inhibitor-sensitive and -resistant tumor models. Furthermore, this control of tumor growth was mediated *via* a STING/IL-15/CD8/NK cell axis wherein the depletion of any of these elements rendered particle treatment ineffective. Prior to the present studies, the use of unloaded PLGA nanoparticles had focused on the anti-inflammatory function of treatment when mice received consecutive daily dosing. However, in light of the present findings it could be hypothesized that in addition to the anti-inflammatory function, the sterile immunity developed in the West Nile Virus (WNV) model may have resulted from an increase in activated NK cells and/or effector/memory WNV-specific CD8+ T cells (35). Further supporting this possibility, the present scRNASeq pathway analysis indicates that treatment of mice with three doses of ONP-302 increased the expression of transcripts associated with IFN-ɣ, IFN-⍺, the activation of the innate immune response, and cellular pathways associated with anti-viral immune response within myeloid cells (e.g., monocytes, macrophages, and neutrophils) (**Figure 1B, C**). In addition, flow cytometric analysis of leukocyte populations from ONP-302-treated mice showed the upregulation of MHC II and costimulatory molecules on various myeloid cell subsets, as well as increases in activation markers on NK cells and CD8+ T cells. (**Figure 2**). Taken together, these findings suggest that treatment with multiple doses of ONP-302 activates cellular pathways within the spleen that would be advantageous for immune cell-mediated control of tumor growth.

cGAS/STING signaling is a well-known pathway which recognizes cytoplasmic DNA and induces the production of IFNs and their downstream proinflammatory cytokines in myeloid cells. *In vitro* uptake of ONP-302 induced an increase in pro-inflammatory cytokines produced by the RAW264.7 macrophage cell line which was inhibited either by use of a STING inhibitor or genetic deletion of cGAS (**Figure 3**). Recent reports indicate a role for host oxidized DNA during cell death as a key trigger of cGAS/STING signaling (29), thus we tested the effects of a caspase inhibitor as PLGA nanoparticle treatment induced apoptosis within myeloid cell *in vivo* (35). Notably, the absence of caspase, STING, cGAS, and Il-15 activity all inhibited the ONP-302-induced increase in secreted cytokine/chemokines and ONP-302 *in vivo* function. IL-15 is a key cytokine for driving the activation of NK and CD8+ T cells (20) further suggesting that NK cells and CD8+ T cells play a key role in ONP-302-induced control of tumor growth as supported by our finding that treatment led to increased infiltrates of NK1.1+ cells and CD8+ T cells near areas of cellular necrosis (**Figure 4E**). Recently published data show that tumor cells survival and growth are not directly altered by ONP-302 (45). Therefore, the ONP-302-induced tumor growth is a consequence of ONP-302 altering myeloid (44, 45), and **Figures 1**–**3**), NK cell, CD8+ T cell (**Figures 4E**, **5**, **6**), and tumor associated fibroblast function (45).Similar to the present findings, treatment of LLC tumor-bearing mice with ONP-302 significantly decreased tumor growth. While the induced increase in the number of NK cells and CD8+ T cells within the tumors on a per gram basis was not statistically significant, there was a trend toward an increase in these cell populations following ONP-302 treatment. These cellular changes correlated with the changes in tumor growth in the absence and presence of anti-PD-1 treatment (45). These results show that ONP-302 is a potent treatment for solid tumors likely *via* stimulating NK cells and CD8+ T cells which use multiple direct and indirect effector mechanisms to regulate tumor growth (5).

We have previously shown that the present PLGA nanoparticles are selectively recognized and bound by inflammatory monocytes and macrophages *via* MARCO (35). MARCO, a type II glycoprotein scavenger receptor, binds anionic ligands (49) and plays an important role in innate defenses against microbial pathogens, uptake of apoptotic cells, and functions in persistent inflammation (50, 51). Upregulation of MARCO on inflammatory monocytes and macrophages is associated with increased phagocytic capacity, inflammatory cytokine production, and TGF-β secretion (52). Our data, considered in conjunction with a recent report demonstrating that MARCO contributes to efficient innate Adenovirus recognition through cGAS (53), strongly suggests that MARCO-mediated uptake of ONP-302 triggers the cGAS/STING cascade leading to IL-15 production and subsequent activation of anti-tumor NK cells and CD8+ T cells.

Translation of CNP-302 to the clinic for tumor immunotherapy due to its direct ability to activate NK cells and CD8+ T cells and ability to enhance the response to anti-PD-1 treatment is facilitated by the fact that it is easily GMP manufacturable and that i.v. administration of gliadin-encapsulating PLGA nanoparticles was proven safe in celiac disease patients (37). Farther studies to trace the pathway from particle uptake to STING signaling are thus of great interest to our group as is the potential role of STING signaling in immune tolerance induction using antigen-encapsulating PLGA nanoparticles (54). The specific mechanism by which CNP-302 internalization triggers the STING pathway remains unclear presently and this is currently under investigation.

Although treatment with ONP-302 resulted in effective tumor growth control and prolonged survival in mouse models of poorly immunogenic tumors, treated mice eventually succumbed to disease. PD-1 and PD-L1 expression are upregulated upon activation in both myeloid and lymphoid cells; however, it is soon followed by activation-induced downregulation of pro-inflammatory and effector functions. While ONP-302 treatment slowed B16.F10 tumor growth as compared to control treated mice, ONP-302 treatment resulted in increased percentages of PD-1+ CD4+ T cells, PD-1+ CD8+ T cells, PD-1+ NK cells, and PD-L1+ myeloid cells. PD-1+ NK cells were increased on day 14 (after three doses of ONP-302) coincident with the appearance of NK cells with an activated CD244+/Granzyme B+/Perforin+ phenotype. Nonetheless, this phenotype marks a systemic shift towards homeostasis after immune activation consistent with a normal immune function, but detrimental to treatment of poorly immunogenic, or ‘cold’, human solid tumors (e.g., pancreatic cancer, ovarian cancer, prostate cancer, and glioblastomas) (55). This finding is likely responsible for the eventual mortality in the active treatment group in the B16.F10 tumor model. In an attempt to therapeutically exploit the ability of ONP-302 to upregulate PD-1/PD-L1 expression on tumor-infiltrating leukocytes, we asked if initial nanoparticle treatment followed by anti-PD-1 checkpoint inhibitor blockade would lead to longer-lived inhibition of growth of B16.F10 melanoma, a ‘cold’ tumor which does not respond to anti-PD-1 monotherapy (47). While anti-PD-1 alone had no efficacy in B16.F10 tumor-bearing mice (45 and data not shown), anti-PD-1 treatment following five initial doses of ONP-302 showed a continued decrease in tumor growth, as compared to mice receiving ONP-302 treatment followed by control Ab treatment (**Figure 7E**). Additionally, ONP-302 plus anti-PD-1 co-treatment showed a trend towards an additive effect in LLC tumor-bearing mice (45). Furthermore, treatment with ONP-302 followed by anti-PD-1 induced a significant increase in the percentage of activated CD8+ and IFN-γ+ T cells within the tumor, hallmarks of effective anti-PD-1 treatment. These results indicate that pharmacologic re-programming of tumor-infiltrating myeloid cells using approaches such as ONP-302 has the capacity for eliciting more durable immune checkpoint-directed anti-tumor responses and improved survival, especially for the treatment of checkpoint blockade resistant immunologically ‘cold’ solid tumors.

## Data availability statement

The original contributions presented in the study are publicly available. This data can be found here: https://www.ebi.ac.uk/arrayexpress/E-MTAB-11584.

## Ethics statement

The animal study was reviewed and approved by Northwestern University Institutional Animal Care and Use Committee (IACUC).

## Author contributions

JP, AC, and SM formed the hypotheses, designed experiments, performed experiments, analyzed results, and prepared the manuscript. TM, AE, and MB helped design experiments, analyze results, and synthesized the ONP-302 nanoparticles. II, TN, and DX helped with data discussion. M-YC, VE, and YY helped to perform the *in vitro* and *in vivo* experiments. KM, SO, LS, and JM helped with performance and data analysis of scRNASeq. All authors contributed to the article and approved the submitted version.

## Acknowledgments

This research was supported by grants from onCour Pharma, Inc (to SM), the National Institutes of Health (R01 AI155678-01 to LS and SM), and the David and Amy Fulton Foundation and the Cramer Family Foundation (to SM). AC was supported by NIH Training Grant T32 AI-0007476. We gratefully acknowledge the Northwestern Mouse Histopathology & Phenotyping Core for their services. We acknowledge the support from all members of the Miller laboratory.

## Conflict of interest 

Authors JP, TM, AE, and MB are employed by Cour Pharmaceutical Development Company. SM and LS are co-founders of, members of the Scientific Advisory Board, grantees of, and hold stock options in Cour Pharmaceutical Development Company.

The remaining authors declare that the research was conducted in the absence of any commercial or financial relationships that could be construed as a potential conflict of interest.

This study received funding from onCOUR Pharm., Inc. The funder had the following involvement with the study formed the hypotheses, designed experiments, performed experiments, analyzed results, and prepared the manuscript. onCOUR Pharma, Inc. holds the patent for this technology. All authors declare no other competing interests.

## Publisher’s note

All claims expressed in this article are solely those of the authors and do not necessarily represent those of their affiliated organizations, or those of the publisher, the editors and the reviewers. Any product that may be evaluated in this article, or claim that may be made by its manufacturer, is not guaranteed or endorsed by the publisher.

## Supplementary material

Supplementary Figure 1 Single cell RNASeq Dot plot and Feature Plot. A representative **(A)** dot plot and **(B)** feature plot for the identification of splenocyte cell populations is presented.

Supplementary Figure 2 Differentiation of myeloid cells from bone marrow of STING^-/-^ mice does not differ from wildtype mice. Bone marrow cells from femurs of either female C57BL/6 mice or STING^-/-^ mice were harvested and cultured in RPMI-1640 with 25 ng/mL of GM-CSF for 7 days. On D7 cells were lifted from the plate and stained with antibodies to CD11b, CD11c, Ly6G and Ly6C. Representative staining of two experiments for C57BL/6 **(A)** and STING^-/-^ **(B)** mice.

Supplementary Figure 3 ONP-302 treatment alters the immune cell populations within the spleens of MC38 tumor-bearing mice over time. Naïve female C57BL/6 mice (n = 9 per treatment group) were injected s.c. with MC38 tumor cells. When the tumors were 50-100 mm^3^ in size, mice were randomized and treated every three days with saline or ONP-302 (1.0 mg/dose in 200 μL of saline) *via* i.v. injection. On Day 8 (prior to the first treatment), Day 14 (24 hours after the third treatment), and Day 20 (24 hours after the fifth treatment), spleens were collected to determine the percentage of various immune cell populations present within the spleen. The general lineage markers for CD45^hi^, CD4^+^ T cells, CD8^+^ T cells, NK cells, CD11b^+^ cells, Ly6C^-^/Ly6G^+^, Ly6C^+^/Ly6G^+^, and IL-15 cells are presented **(A, B)**. The specific effector phenotypes of the myeloid **(C)**, NK **(D)** and CD8^+^ T cells **(D)** were determined by intracellular staining. The data are presented as the mean percentage of cells ± S.E.M. One representative experiment of two is presented. Asterisks (*, **, ***, ****) indicates a statistically significant difference as compared to saline treated mice, p < 0.05, < 0.01, < 0.001, and < 0.0001 respectively.

Supplementary Figure 4 ONP-302 treatment alters the immune cell populations within the TME of MC38 tumor-bearing mice over time. Naïve female C57BL/6 mice (n = 9 per treatment group) were injected s.c. with MC38 tumor cells. When the tumors were 50-100 mm^3^ in size, mice were randomized and treated every three days with saline or ONP-302 (1.0 mg/dose in 200 μL of saline) *via* i.v. injection. On Day 8 (prior to the first treatment), Day 14 (24 hours after the third treatment), and Day 20 (24 hours after the fifth treatment), tumors were collected to determine the percentage of various immune cell populations present. The general lineage markers for CD45^hi^, CD4^+^ T cells, CD8^+^ T cells, NK cells, CD11b^+^ cells, Ly6C^-^/Ly6G^+^, Ly6C^+^/Ly6G^+^, and IL-15 cells are presented **(A, B**). The specific effector phenotypes of the myeloid **(C)**, NK **(D)**, and CD8^+^ T cells **(D)** were determined by intracellular staining. The data are presented as the mean percentage of cells ± S.E.M. One representative experiment of two is presented. Asterisks (*, **, ****) indicates a statistically significant difference as compared to saline treated mice, p < 0.05, < 0.01, and < 0.0001 respectively.

Supplementary Figure 5 ONP-302 treatment alters the CD8+ T cell and NK cell populations within the blood of B16.F10 and MC38 tumor-bearing mice over time. The phenotype of CD8+ T cells and NK cells within the blood of the mice analyzed in (**Figure 5**) and Supp. (**Figure 2**) were analyzed over the same time course. The general lineage markers for CD45^hi^, CD8^+^ T cells, NK cells, and IL-15 cells are presented. As well as the percentage of IFN-γ^+^ and CD25^-^/CD44^hi^ CD8^+^ T cells, and the percentage of granzyme B^+^ and perforin^+^ NK cells. The data for B16.F10 tumor-bearing mice **(A)**, and MC38 tumor-bearing mice **(B)** are presented. The data are presented as the mean percentage of cells ± S.E.M. One representative experiment of two is presented. Asterisks (*, **, ***, ****) indicates a statistically significant difference as compared to saline treated mice, p < 0.05, < 0.01, < 0.001, and < 0.0001 respectively.

Supplementary Figure 6 NK cells are required for ONP-302 anti-MC38 tumor function. Naïve female C57BL/6 mice were injected s.c. with MC38 tumor cells. When the tumors were 50-100 mm^3^ in size, mice were randomized and treated every three days with saline or ONP-302 (1.0 mg/dose in 200 μL of saline) *via* i.v. injection. To determine the role of NK cells in tumor resistance, mice were treated with a species and isotype matched control antibody or anti-NK1.1 (100 μg/dose) one day prior to each saline of ONP-302 treatment (n = 5 per treatment group). The tumor volumes were measure on the indicted days, and the data are presented as the mean tumor volume ± S.E.M. One representative experiment of two is presented. Asterisks (****) indicates a statistically significant difference as compared to saline treated mice, p < 0.0001 respectively.

Supplementary Table 1 Antibodies used for flow cytometric analysis.

## Abbreviations

APCs, antigen-presenting cells; CAR-Ts, chimeric antigen receptor T cells; cGAS, cyclic GMP-AMP synthase; IMP, immune modifying nanoparticle; IRF, interferon response factor; ISGs, interferon-stimulated genes; MARCO, macrophage receptor with collagenous structure; MDSCs, myeloid-derived suppressor cells; NF-kB, Nuclear Factor kappa B; NK cells, natural killer cells; PLGA, poly(lactic-co-glycolic acid); STING, stimulator of interion genes; TAMs, tumor-associated macrophages; TME, tumor microenvironment.
