# Supplementary figures and images for: Biodegradable nanoparticles induce cGAS/STING-dependent reprogramming of myeloid cells to promote tumor immunotherapy
Source: Front Immunol. 2022 Aug 18;13:887649. doi: 10.3389/fimmu.2022.887649 (PMC9433741; doi:10.3389/fimmu.2022.887649)

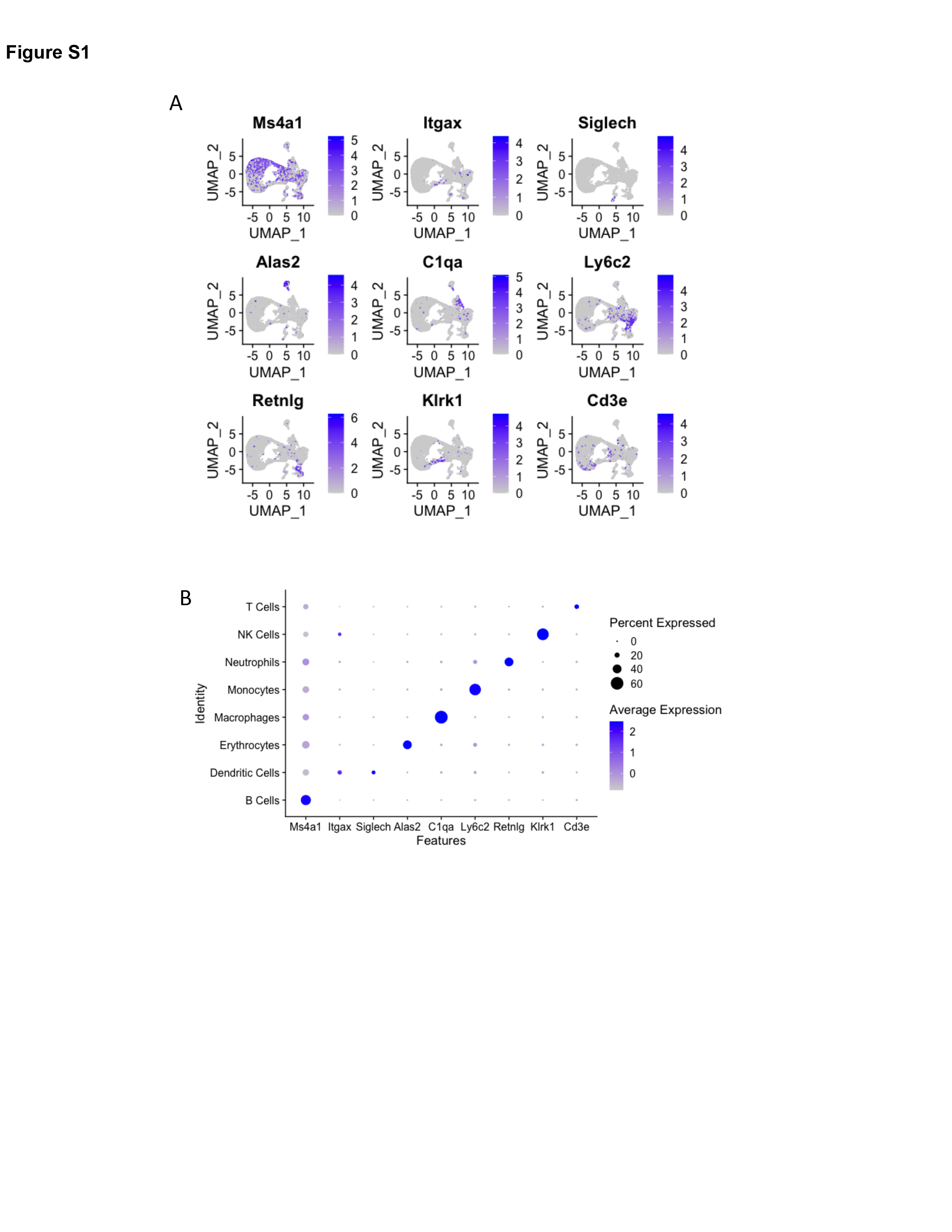

Supplement: Supplementary Figure 1 — Single cell RNASeq Dot plot and Feature Plot. A representative (A) dot plot and (B) feature plot for the identification of splenocyte cell populations is presented. [file Image_1.tif]

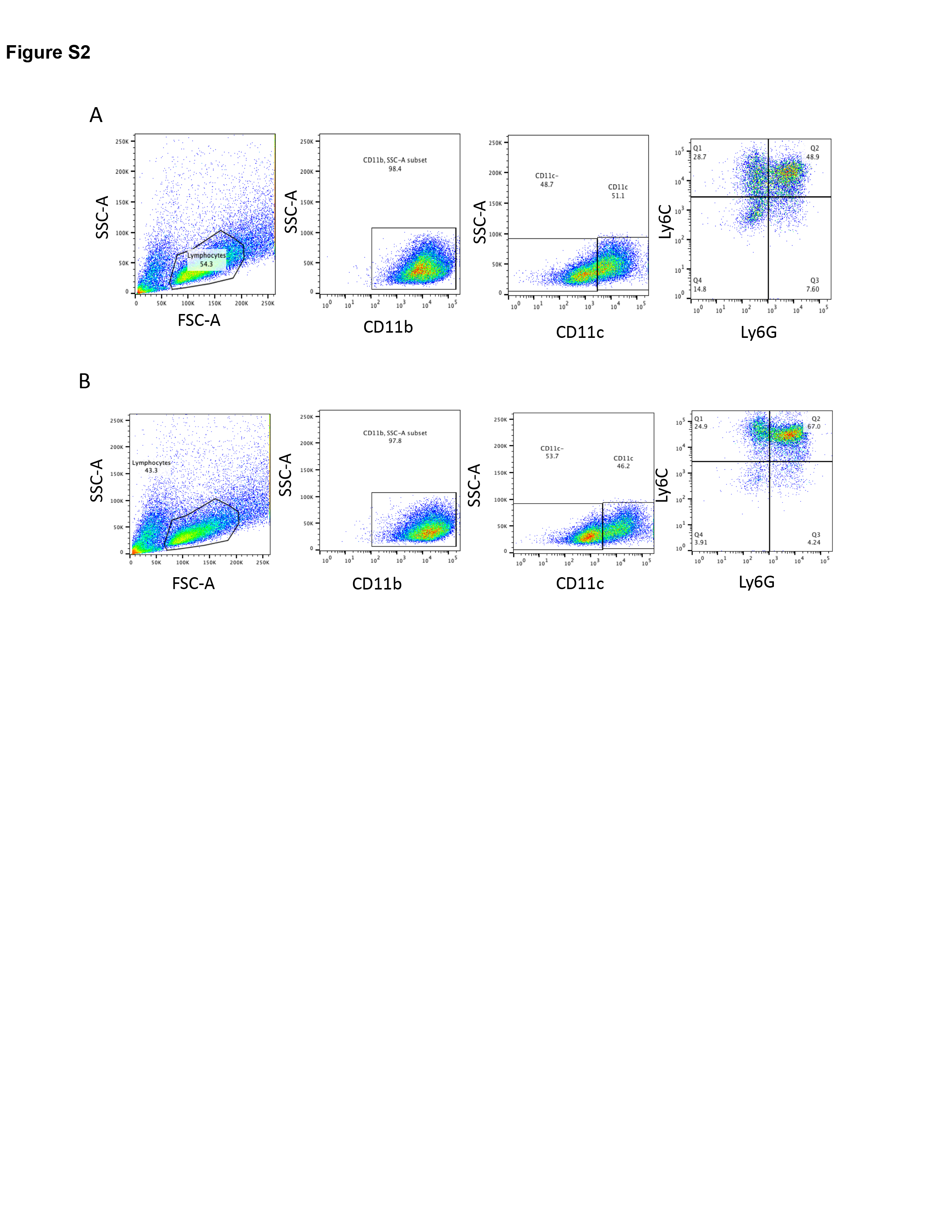

Supplement: Supplementary Figure 2 — Differentiation of myeloid cells from bone marrow of STING-/- mice does not differ from wildtype mice. Bone marrow cells from femurs of either female C57BL/6 mice or STING-/- mice were harvested and cultured in RPMI-1640 with 25 ng/mL of GM-CSF for 7 days. On D7 cells were lifted from the plate and stained with antibodies to CD11b, CD11c, Ly6G and Ly6C. Representative staining of two experiments for C57BL/6 (A) and STING-/- (B) mice. [file Image_2.tif]

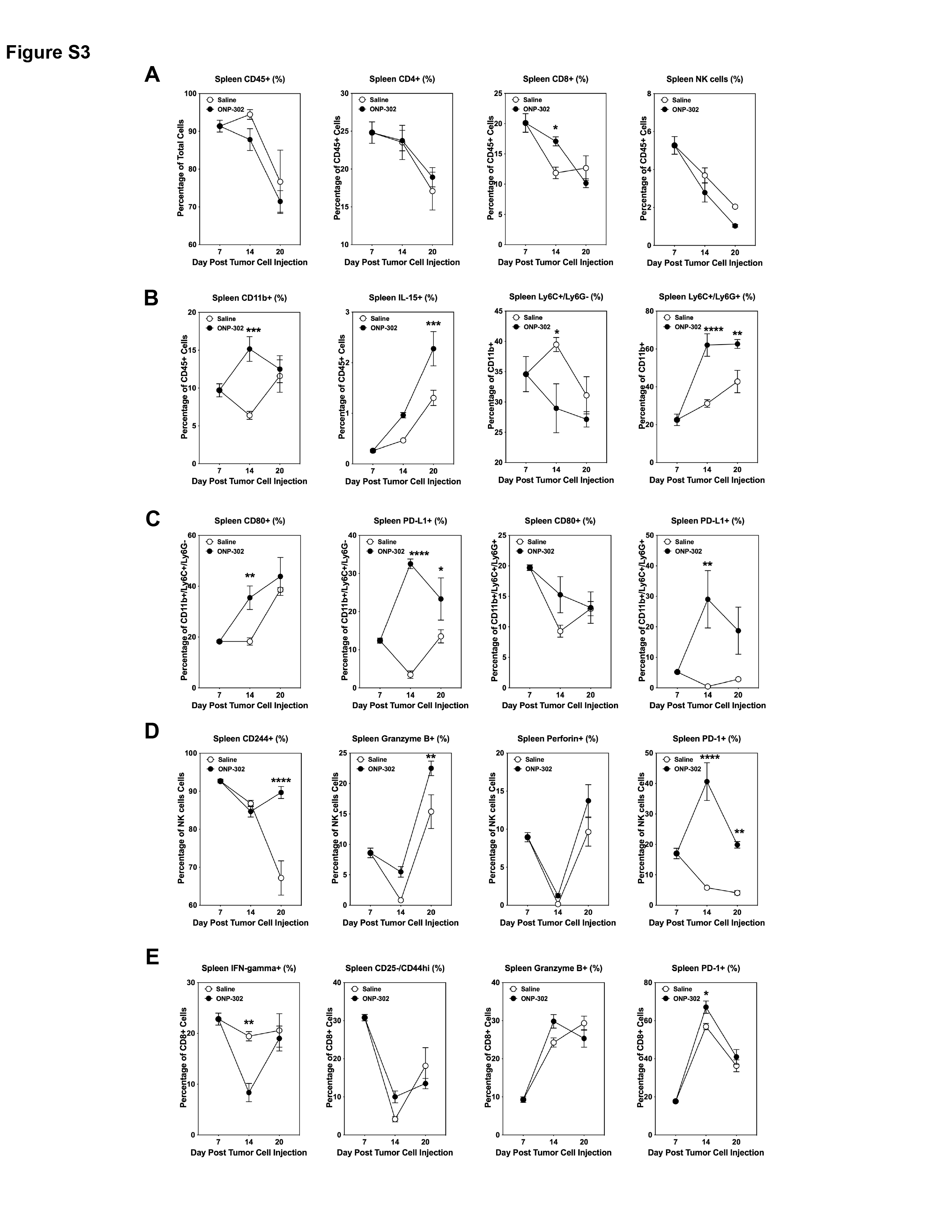

Supplement: Supplementary Figure 3 — ONP-302 treatment alters the immune cell populations within the spleens of MC38 tumor-bearing mice over time. Naïve female C57BL/6 mice (n = 9 per treatment group) were injected s.c. with MC38 tumor cells. When the tumors were 50-100 mm3 in size, mice were randomized and treated every three days with saline or ONP-302 (1.0 mg/dose in 200 μL of saline) via i.v. injection. On Day 8 (prior to the first treatment), Day 14 (24 hours after the third treatment), and Day 20 (24 hours after the fifth treatment), spleens were collected to determine the percentage of various immune cell populations present within the spleen. The general lineage markers for CD45hi, CD4+ T cells, CD8+ T cells, NK cells, CD11b+ cells, Ly6C-/Ly6G+, Ly6C+/Ly6G+, and IL-15 cells are presented (A, B). The specific effector phenotypes of the myeloid (C), NK (D) and CD8+ T cells (D) were determined by intracellular staining. The data are presented as the mean percentage of cells ± S.E.M. One representative experiment of two is presented. Asterisks (*, **, ***, ****) indicates a statistically significant difference as compared to saline treated mice, p < 0.05, < 0.01, < 0.001, and < 0.0001 respectively. [file Image_3.tif]

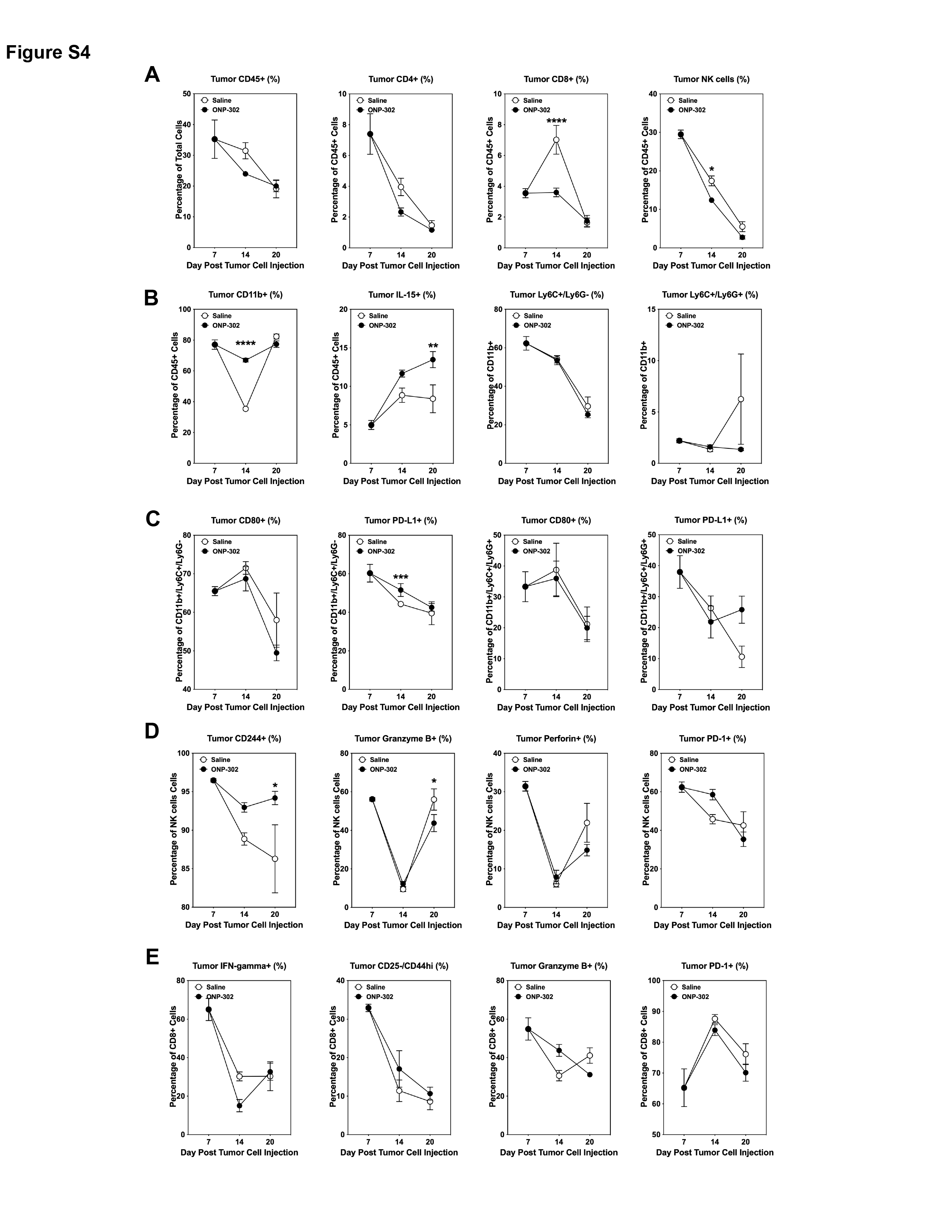

Supplement: Supplementary Figure 4 — ONP-302 treatment alters the immune cell populations within the TME of MC38 tumor-bearing mice over time. Naïve female C57BL/6 mice (n = 9 per treatment group) were injected s.c. with MC38 tumor cells. When the tumors were 50-100 mm3 in size, mice were randomized and treated every three days with saline or ONP-302 (1.0 mg/dose in 200 μL of saline) via i.v. injection. On Day 8 (prior to the first treatment), Day 14 (24 hours after the third treatment), and Day 20 (24 hours after the fifth treatment), tumors were collected to determine the percentage of various immune cell populations present. The general lineage markers for CD45hi, CD4+ T cells, CD8+ T cells, NK cells, CD11b+ cells, Ly6C-/Ly6G+, Ly6C+/Ly6G+, and IL-15 cells are presented (A, B). The specific effector phenotypes of the myeloid (C), NK (D), and CD8+ T cells (D) were determined by intracellular staining. The data are presented as the mean percentage of cells ± S.E.M. One representative experiment of two is presented. Asterisks (*, **, ****) indicates a statistically significant difference as compared to saline treated mice, p < 0.05, < 0.01, and < 0.0001 respectively. [file Image_4.tif]

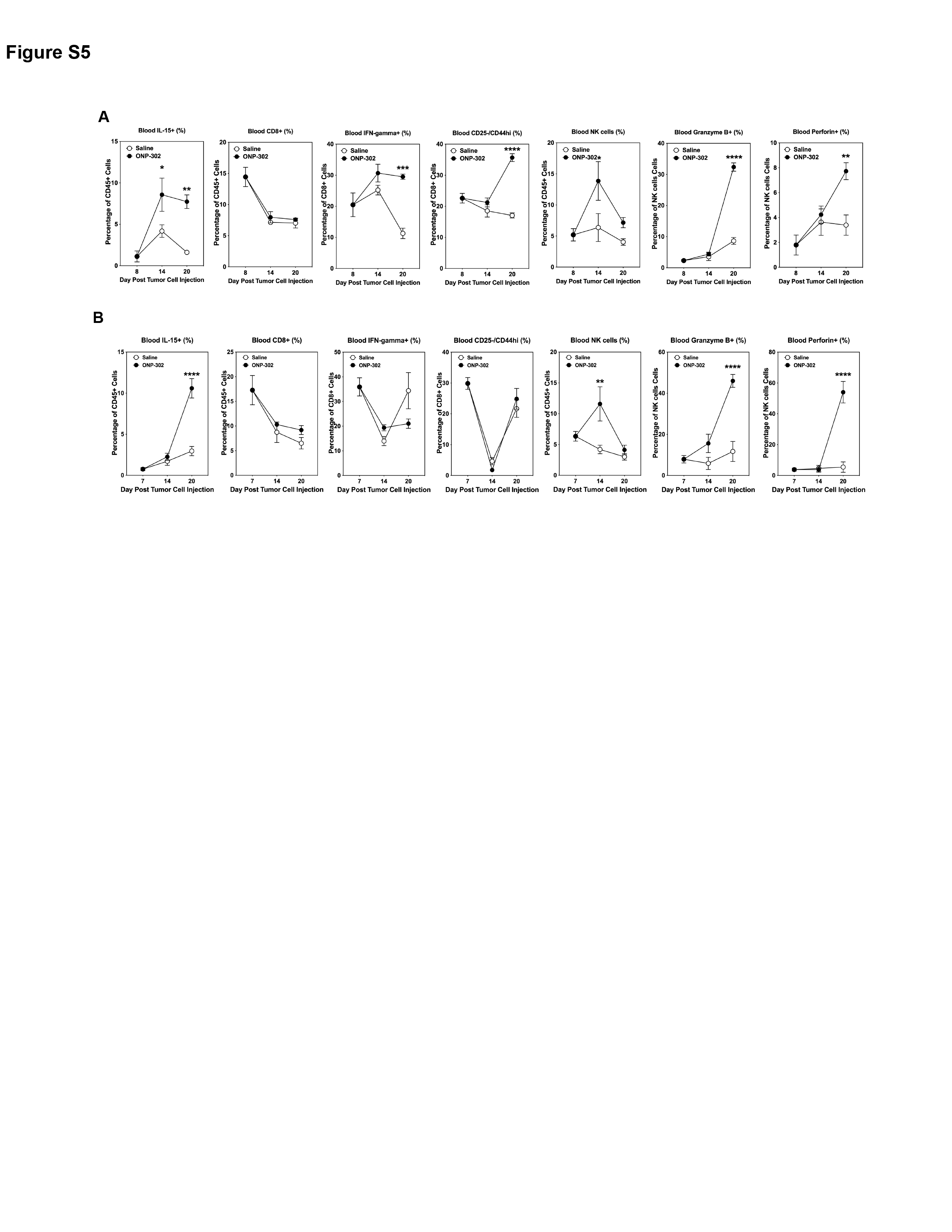

Supplement: Supplementary Figure 5 — ONP-302 treatment alters the CD8+ T cell and NK cell populations within the blood of B16.F10 and MC38 tumor-bearing mice over time. The phenotype of CD8+ T cells and NK cells within the blood of the mice analyzed in ( Figure 5 ) and Supp. ( Figure 2 ) were analyzed over the same time course. The general lineage markers for CD45hi, CD8+ T cells, NK cells, and IL-15 cells are presented. As well as the percentage of IFN-γ+ and CD25-/CD44hi CD8+ T cells, and the percentage of granzyme B+ and perforin+ NK cells. The data for B16.F10 tumor-bearing mice (A), and MC38 tumor-bearing mice (B) are presented. The data are presented as the mean percentage of cells ± S.E.M. One representative experiment of two is presented. Asterisks (*, **, ***, ****) indicates a statistically significant difference as compared to saline treated mice, p < 0.05, < 0.01, < 0.001, and < 0.0001 respectively. [file Image_5.tif]

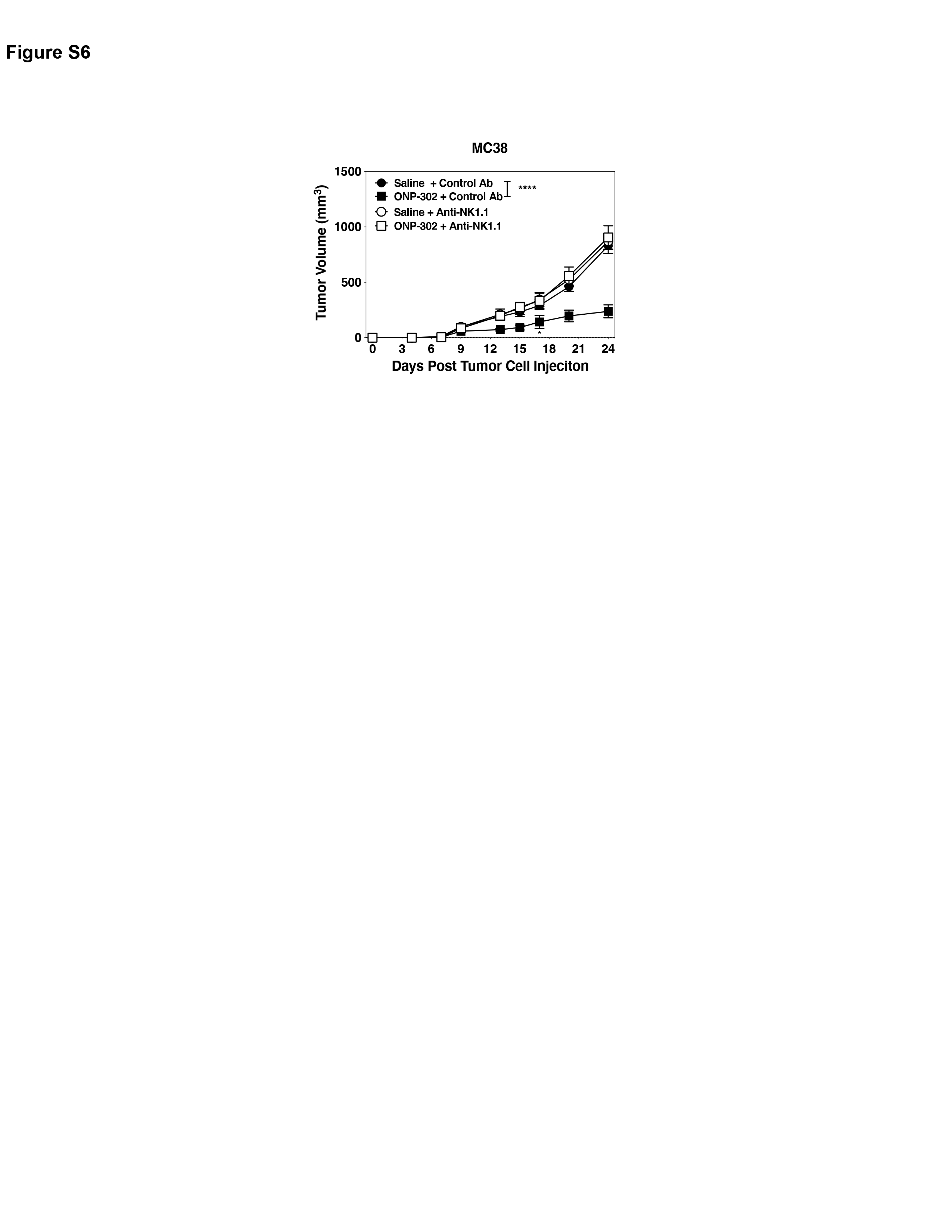

Supplement: Supplementary Figure 6 — NK cells are required for ONP-302 anti-MC38 tumor function. Naïve female C57BL/6 mice were injected s.c. with MC38 tumor cells. When the tumors were 50-100 mm3 in size, mice were randomized and treated every three days with saline or ONP-302 (1.0 mg/dose in 200 μL of saline) via i.v. injection. To determine the role of NK cells in tumor resistance, mice were treated with a species and isotype matched control antibody or anti-NK1.1 (100 μg/dose) one day prior to each saline of ONP-302 treatment (n = 5 per treatment group). The tumor volumes were measure on the indicted days, and the data are presented as the mean tumor volume ± S.E.M. One representative experiment of two is presented. Asterisks (****) indicates a statistically significant difference as compared to saline treated mice, p < 0.0001 respectively. [file Image_6.tif]
