# Supplementary material for: Biodegradable nanoparticles induce cGAS/STING-dependent reprogramming of myeloid cells to promote tumor immunotherapy
Source: Front Immunol. 2022 Aug 18;13:887649. doi: 10.3389/fimmu.2022.887649 (PMC9433741; doi:10.3389/fimmu.2022.887649)
Supplement: Supplementary Table 1 — Antibodies used for flow cytometric analysis. [file Table_1.pdf]

Supplemental Table 1. Antibodies used for flow cytometric analysis.

Cell marker	Color	Clone	Vendor
**iNOS**	PE	CXNFT	Invitrogen
**Perforin**	PE	eBioOMAK-D	eBioscience
**MHCII**	PE	NIMR-4	eBioscience
**IFN-γ**	PE	XMG1.2	Invitrogen
**IL-15**	PE	Polyclonal	AssayPro
**Il-10**	PE	JES5-16E3	eBioscience
**Foxp3**	PE	FJK-16S	Invitrogen
**CCL3**	PE	DNT3CC	Invitrogen
**Arginase**	PE-Cy7	A1exF5	eBioscience
**CD244**	PE-Cy7	eBio244F4	eBioscience
**CD40**	PE-Cy7	3/23	BioLegend
**CD25**	PE-Cy7	PC61.5	eBioscience
**OX-40**	Biotin	RM134L	BD
**Streptavidin**	PE-Cy7		eBioscience
**IL-1b**	PE-Cy7	NJTEN3	Invitrogen
**IL-10**	PE-Cy7	JES5-16E3	BioLegend
**IL-17A**	PE-Cy7	eBio17B7	Pharmingen
**F4/80**	FITC	BM8	eBioscience
**Granzyme B**	FITC	NG2B	eBioscience
**CD44**	FITC	IM7	BD
**VLA4**	FITC	R1-2	eBioscience
**Ly-6C**	PerCP-Cy5.5	AL-21	BD
**CD45**	PerCP-Cy5.5	30-F11	BD
**Lag3 (CD223)**	PerCP-Cy5.5	C9B7W	BD
**IFN-γ**	PerCP-Cy5.5	XMG1 .2	eBioscience
**CD206**	eFluor450	MR6F	eBioscience
**NKG2D (CD314)**	BV421	CX5	BD
**CD86**	PB/BV421	GL-1	BioLegend
**PD-1 (CD279)**	BV421	J43	BD
**PD-L2**	PB/BV421	TY25	BioLegend
**IL-12**	eFluor450	C17.8	eBioscience
**CTLA4 (CD152)**	PB/BV421	UC10-4B9	eBioscience
**CD45**	BV605	RM4-5	BD
**Ki67**	BV605	16A8	BioLegend
**CD8**	BV605	53-6.7	BD
**CD80**	BV650	16-10A1	BD
**PD-L1**	BV650	MIH5	BD
**TNFa**	BV650	MP6-XT22	BD
**CD4**	BV650	RM4-5	BD
**CD11c**	BV711	HL3	BD
**CD3**	BV711	145-2C11	BD
**CD11b**	BV786	M1/70	BD
**NK1.1**	BV786	PK136	BD
**CD44**	BV786	IM7	BD
**CD25**	BV786	PC61	BD
**Ly-6G**	PE-CF594	1A8	BD
**PD-1 (CD279)**	PE-CF594	J43	BD
**CD122**	PE-CF594	TM-b1	BD
