# Supplementary material for: Biodegradable nanoparticles induce cGAS/STING-dependent reprogramming of myeloid cells to promote tumor immunotherapy
Source: Front Immunol. 2022 Aug 18;13:887649. doi: 10.3389/fimmu.2022.887649 (PMC9433741; doi:10.3389/fimmu.2022.887649)
Supplement: Supplementary file 8 [file Table_2.pdf]

Supplemental Table 2. Signaling Pathway Analysis for All Myeloid Cells - 3 Consecutive Doses - ONP-302 vs. Saline

NAME	SIZE	ES	NES	NOM p-val	FDR q-val	FWER p-va	RANK AT M	LEADING E
HALLMARK_INTERFERON_GAMMA_RESPONSE	62	0.736	3.699	0	0	0	165	tags=81%,
HALLMARK_INTERFERON_ALPHA_RESPONSE	41	0.762	3.418	0	0	0	147	tags=80%,
GOBP_DEFENSE_RESPONSE_TO_VIRUS	40	0.709	3.315	0	0	0	170	tags=80%,
GOBP_DEFENSE_RESPONSE_TO_OTHER_ORGANISM	104	0.570	3.214	0	0	0	170	tags=58%,
GOBP_INNATE_IMMUNE_RESPONSE	92	0.589	3.214	0	0	0	165	tags=59%,
GOBP_RESPONSE_TO_VIRUS	49	0.653	3.207	0	0	0	170	tags=69%,
GOBP_REGULATION_OF_RESPONSE_TO_BIOTIC_STIMULUS	47	0.653	3.108	0	0	0	119	tags=60%,
REACTOME_INTERFERON_SIGNALING	34	0.729	3.104	0	0	0	87	tags=62%,
GOBP_RESPONSE_TO_TYPE_I_INTERFERON	26	0.729	2.927	0	0	0	85	tags=58%,
GOBP_REGULATION_OF_INNATE_IMMUNE_RESPONSE	40	0.635	2.914	0	0	0	119	tags=58%,
REACTOME_INTERFERON_ALPHA_BETA_SIGNALING	21	0.752	2.850	0	0	0	103	tags=67%,
GOBP_POSITIVE_REGULATION_OF_RESPONSE_TO_BIOTIC_STIMULUS	29	0.673	2.791	0	0	0	103	tags=55%,
GOBP_RESPONSE_TO_INTERFERON_GAMMA	31	0.670	2.790	0	0	0	152	tags=65%,
GOBP_RESPONSE_TO_BIOTIC_STIMULUS	140	0.471	2.786	0	0	0	170	tags=46%,
GOBP_INTERFERON_GAMMA_MEDIATED_SIGNALING_PATHWAY	19	0.734	2.728	0	0	0	152	tags=79%,
GOBP_DEFENSE_RESPONSE	141	0.450	2.659	0	0	0	170	tags=45%,
REACTOME_INTERFERON_GAMMA_SIGNALING	16	0.739	2.625	0	0	0	103	tags=69%,
GOBP_REGULATION_OF_IMMUNE_RESPONSE	88	0.474	2.602	0	0	0	166	tags=47%,
GOBP_NEGATIVE_REGULATION_OF_VIRAL_PROCESS	17	0.735	2.594	0	7.31E-05	0.002	165	tags=82%,
GOBP_REGULATION_OF_DEFENSE_RESPONSE	69	0.500	2.575	0	6.95E-05	0.002	133	tags=43%,
GOBP_RESPONSE_TO_CYTOKINE	118	0.442	2.551	0	9.90E-05	0.003	165	tags=42%,
GOBP_CYTOKINE_MEDIATED_SIGNALING_PATHWAY	87	0.458	2.522	0	1.90E-04	0.006	163	tags=44%,
GOBP_REGULATION_OF_VIRAL_LIFE_CYCLE	19	0.682	2.510	0	1.82E-04	0.006	165	tags=74%,
GOBP_REGULATION_OF_BIOLOGICAL_PROCESS_INVOLVED_IN_SYMBIOTIC_INTEF	22	0.657	2.484	0	2.04E-04	0.007	165	tags=68%,
GOBP_POSITIVE_REGULATION_OF_DEFENSE_RESPONSE	36	0.536	2.459	0.00147710	2.80E-04	0.01	131	tags=44%,
GOBP_POSITIVE_REGULATION_OF_IMMUNE_RESPONSE	58	0.484	2.431	0	4.60E-04	0.017	103	tags=34%,
REACTOME_CYTOKINE_SIGNALING_IN_IMMUNE_SYSTEM	82	0.446	2.402	0	5.72E-04	0.022	103	tags=34%,
GOBP_REGULATION_OF_IMMUNE_SYSTEM_PROCESS	127	0.416	2.392	0	7.03E-04	0.028	166	tags=42%,
GOBP_REGULATION_OF_RESPONSE_TO_EXTERNAL_STIMULUS	89	0.437	2.385	0	7.51E-04	0.03	138	tags=39%,
GOBP_REGULATION_OF_RESPONSE_TO_CYTOKINE_STIMULUS	29	0.579	2.382	0	7.26E-04	0.03	163	tags=62%,
HALLMARK_ALLOGRAFT_REJECTION	20	0.631	2.349	0	0.00133802	0.058	148	tags=65%,
GOBP_REGULATION_OF_RESPONSE_TO_STRESS	108	0.417	2.328	0	0.00186848	0.082	133	tags=36%,
GOBP_TYPE_I_INTERFERON_PRODUCTION	21	0.620	2.318	0	0.00198391	0.09	136	tags=57%,
GOBP_POSITIVE_REGULATION_OF_RESPONSE_TO_EXTERNAL_STIMULUS	49	0.483	2.293	0	0.00254954	0.118	147	tags=45%,
REACTOME_CLASS_I_MHC_MEDIATED_ANTIGEN_PROCESSING_PRESENTATION	32	0.527	2.270	0	0.00330334	0.154	97	tags=44%,
GOBP_ACTIVATION_OF_INNATE_IMMUNE_RESPONSE	17	0.648	2.261	0	0.00354530	0.165	97	tags=53%,
GOBP_BIOLOGICAL_PROCESS_INVOLVED_IN_SYMBIOTIC_INTERACTION	91	0.406	2.247	0	0.00398220	0.187	103	tags=30%,
GOBP_POSITIVE_REGULATION_OF_IMMUNE_SYSTEM_PROCESS	88	0.398	2.198	0	0.00651762	0.299	147	tags=38%,
GOBP_DEFENSE_RESPONSE_TO_BACTERIUM	17	0.621	2.175	0	0.00799618	0.356	90	tags=47%,
GOBP_RESPONSE_TO_INTERLEUKIN_1	20	0.567	2.171	0.00153846	0.00811647	0.366	101	tags=45%,
GOBP_PROTEIN_POLYUBIQUITINATION	26	0.535	2.170	0	0.00807262	0.375	136	tags=50%,
GOBP_PATTERN_RECOGNITION_RECEPTOR_SIGNALING_PATHWAY	26	0.529	2.147	0	0.00996306	0.45	159	tags=54%,
GOBP_CELLULAR_RESPONSE_TO_MOLECULE_OF_BACTERIAL_ORIGIN	18	0.587	2.141	0.00306278	0.01029035	0.471	35	tags=28%,
GOBP_RESPONSE_TO_BACTERIUM	51	0.443	2.140	0.00132450	0.01029785	0.475	90	tags=29%,
GOMF_HYDROLASE_ACTIVITY_ACTING_ON_ACID_ANHYDRIDES	35	0.478	2.122	0.00285306	0.01182450	0.534	75	tags=29%,
GOBP_VIRAL_GENOME_REPLICATION	15	0.632	2.121	0	0.01169177	0.538	79	tags=47%,
GOBP_VIRAL_LIFE_CYCLE	30	0.501	2.083	0	0.01673608	0.669	165	tags=53%,
REACTOME_ANTIGEN_PROCESSING_UBIQUITINATION_PROTEASOME_DEGRADAT	24	0.528	2.079	0	0.01685656	0.679	97	tags=46%,
GOBP_IMMUNE_EFFECTOR_PROCESS	131	0.355	2.061	0	0.01971482	0.735	170	tags=38%,
GOMF_GUANYL_NUCLEOTIDE_BINDING	15	0.604	2.045	0.00466563	0.02246962	0.788	24	tags=27%,
REACTOME_ANTIGEN_PROCESSING_CROSS_PRESENTATION	15	0.616	2.041	0.00326797	0.0228219	0.797	97	tags=53%,
GOBP_CELLULAR_RESPONSE_TO_BIOTIC_STIMULUS	21	0.518	1.999	0.00297176	0.03180976	0.888	35	tags=24%,
REACTOME_ADAPTIVE_IMMUNE_SYSTEM	56	0.403	1.995	0.00131061	0.03215635	0.897	246	tags=59%,
GOBP_ANTIGEN_PROCESSING_AND_PRESENTATION_OF_PEPTIDE_ANTIGEN_VIA_	17	0.558	1.981	0.00148588	0.03577868	0.926	97	tags=47%,
GOBP_ACTIVATION_OF_IMMUNE_RESPONSE	37	0.430	1.961	0.00538358	0.04152027	0.959	101	tags=32%,
GOBP_RESPONSE_TO_TUMOR_NECROSIS_FACTOR	29	0.470	1.957	0.00412087	0.04194235	0.967	119	tags=38%,
GOBP_NEGATIVE_REGULATION_OF_IMMUNE_SYSTEM_PROCESS	36	0.435	1.943	0.00141442	0.04653313	0.981	127	tags=39%,
HP_IMMUNODEFICIENCY	18	0.536	1.921	0.00456621	0.05417978	0.991	145	tags=56%,
GOBP_PROTEIN_MODIFICATION_BY_SMALL_PROTEIN_REMOVAL	25	0.477	1.907	0.00604229	0.05967084	0.995	256	tags=72%,
GOBP_PROTEIN_MODIFICATION_BY_SMALL_PROTEIN_CONJUGATION	62	0.375	1.898	0.00636942	0.0627111	0.996	146	tags=37%,
GOBP_TUMOR_NECROSIS_FACTOR_MEDIATED_SIGNALING_PATHWAY	21	0.492	1.890	0.01156069	0.06537387	0.997	163	tags=52%,
GOBP_BIOLOGICAL_PROCESS_INVOLVED_IN_INTERACTION_WITH_HOST	19	0.510	1.886	0.00928792	0.06617694	0.997	139	tags=42%,
REACTOME_HOST_INTERACTIONS_OF_HIV_FACTORS	17	0.520	1.842	0.01076923	0.08995806	0.999	97	tags=47%,
GOBP_PROTEIN_MODIFICATION_BY_SMALL_PROTEIN_CONJUGATION_OR_REMOV	73	0.353	1.836	0.00363196	0.09260645	1	146	tags=36%,
GOBP_ANTIGEN_PROCESSING_AND_PRESENTATION_OF_PEPTIDE_ANTIGEN	22	0.472	1.835	0.01508295	0.09172721	1	246	tags=68%,
GOCC_NUCLEOLUS	35	0.411	1.822	0.00404312	0.09908893	1	86	tags=29%,
GOCC_PHAGOCYTIC_VESICLE	15	0.528	1.820	0.01444622	0.09911799	1	124	tags=40%,
HP_RECURRENT_RESPIRATORY_INFECTIONS	31	0.420	1.819	0.01544943	0.09789981	1	119	tags=39%,
HALLMARK_INFLAMMATORY_RESPONSE	22	0.466	1.815	0.02040816	0.09930169	1	245	tags=64%,
REACTOME_HIV_INFECTION	21	0.463	1.790	0.01040118	0.11614539	1	247	tags=67%,
GOMF_UBIQUITIN_LIKE_PROTEIN_TRANSFERASE_ACTIVITY	30	0.418	1.764	0.02034883	0.13660637	1	164	tags=47%,
HP_ABNORMALITY_OF_THE_UPPER_RESPIRATORY_TRACT	26	0.426	1.762	0.01772525	0.13641715	1	112	tags=35%,
REACTOME_UB_SPECIFIC_PROCESSING_PROTEASES	19	0.471	1.757	0.02322206	0.13920824	1	120	tags=47%,
GOBP_NEGATIVE_REGULATION_OF_IMMUNE_RESPONSE	17	0.492	1.748	0.01848998	0.14518599	1	73	tags=35%,
GOMF_PROTEIN_HOMODIMERIZATION_ACTIVITY	37	0.389	1.728	0.01366120	0.16230261	1	87	tags=27%,
GOBP_IMMUNE_SYSTEM_DEVELOPMENT	85	0.317	1.724	0.00493827	0.16458398	1	145	tags=32%,
GOBP_CELLULAR_RESPONSE_TO_LIPID	33	0.396	1.719	0.02770083	0.16675664	1	35	tags=15%,
REACTOME_DDX58_IFIH1_MEDIATED_INDUCTION_OF_INTERFERON_ALPHA_BETA	15	0.496	1.715	0.02978056	0.1683836	1	256	tags=73%,

GOBP_INTRACELLULAR_RECEPTOR_SIGNALING_PATHWAY	24	0.440	1.715	0.02612481	0.16688512	1	271	tags=67%, l
GOMF_IDENTICAL_PROTEIN_BINDING	113	0.299	1.707	0.00931315	0.1726153	1	228	tags=45%, l
GOBP_RESPONSE_TO_MOLECULE_OF_BACTERIAL_ORIGIN	32	0.398	1.697	0.02691218	0.18203315	1	35	tags=16%, l
GOBP_ADAPTIVE_IMMUNE_RESPONSE	40	0.366	1.686	0.01869158	0.19223246	1	166	tags=40%, l
GOBP_TOLL_LIKE_RECEPTOR_SIGNALING_PATHWAY	21	0.439	1.677	0.04141104	0.20025407	1	149	tags=48%, l
GOBP_REGULATION_OF_PROTEIN_MODIFICATION_BY_SMALL_PROTEIN_CONJUGA	16	0.481	1.676	0.03656597	0.19855046	1	119	tags=44%, l
GOBP_REGULATION_OF_IMMUNE_EFFECTOR_PROCESS	34	0.389	1.676	0.02538787	0.19629896	1	143	tags=38%, l
GOBP_MONONUCLEAR_CELL_DIFFERENTIATION	31	0.390	1.668	0.03380282	0.20332408	1	145	tags=39%, l
GOBP_MOVEMENT_IN_HOST_ENVIRONMENT	15	0.480	1.659	0.02184087	0.2126436	1	139	tags=47%, l
REACTOME_DEUBIQUITINATION	25	0.412	1.657	0.02503682	0.21266164	1	256	tags=64%, l
GOCC_GOLGI_APPARATUS_SUBCOMPARTMENT	34	0.372	1.637	0.03945578	0.23633544	1	46	tags=12%, l
HALLMARK_TNFA_SIGNALING_VIA_NFKB	35	0.370	1.636	0.04219409	0.23423375	1	119	tags=34%, l
HP_UNUSUAL_INFECTION	50	0.337	1.622	0.02359108	0.25087368	1	119	tags=30%, l
GOBP_POSITIVE_REGULATION_OF_CYTOKINE_PRODUCTION	49	0.345	1.621	0.03866666	0.24854016	1	37	tags=16%, l
GOBP_REGULATION_OF_AUTOPHAGY	29	0.388	1.616	0.03168044	0.2526253	1	9	tags=10%, l
GOMF_TRANSFERASE_ACTIVITY_TRANSFERRING_GLYCOSYL_GROUPS	16	0.462	1.612	0.04573171	0.25521058	1	131	tags=44%, l
GOBP_NIK_NF_KAPPAB_SIGNALING	19	0.451	1.603	0.03933434	0.2665266	1	165	tags=53%, l
HP_ABNORMAL_LYMPHOCYTE_PHYSIOLOGY	22	0.407	1.599	0.05994152	0.26882958	1	145	tags=45%, l
GOBP_ANTIGEN_RECEPTOR_MEDIATED_SIGNALING_PATHWAY	23	0.410	1.596	0.05231689	0.27101907	1	97	tags=35%, l
GOBP_ANTIGEN_PROCESSING_AND_PRESENTATION	24	0.407	1.595	0.05385735	0.26964822	1	246	tags=63%, l
GOBP_CYTOKINE_PRODUCTION	77	0.301	1.593	0.03059975	0.2698443	1	117	tags=26%, l
GOBP_POST_TRANSLATIONAL_PROTEIN_MODIFICATION	21	0.419	1.577	0.04147465	0.29031727	1	97	tags=33%, l
GOBP_T_CELL_DIFFERENTIATION	17	0.442	1.573	0.06824925	0.29354438	1	127	tags=41%, l
HP_ABNORMAL_TRACHEOBRONCHIAL_MORPHOLOGY	16	0.458	1.570	0.06491499	0.295213	1	87	tags=31%, l
GOBP_REGULATION_OF_HEMOPOIESIS	32	0.363	1.551	0.06017192	0.321813	1	127	tags=34%, l
GOBP_FC_EPSILON_RECEPTOR_SIGNALING_PATHWAY	15	0.454	1.548	0.05965463	0.32479873	1	140	tags=47%, l
GOBP_NEGATIVE_REGULATION_OF_DEFENSE_RESPONSE	18	0.423	1.534	0.07230769	0.34610912	1	73	tags=33%, l
HP_ABNORMAL_LYMPHOCYTE_MORPHOLOGY	16	0.437	1.532	0.09567901	0.34647727	1	117	tags=38%, l
HP_LEUKOPENIA	19	0.404	1.526	0.08054711	0.3536089	1	145	tags=42%, l
GOBP_LYMPHOCYTE_MEDIATED_IMMUNITY	21	0.409	1.503	0.08411215	0.3913618	1	166	tags=48%, l
REACTOME_NEDDYLATION	15	0.437	1.501	0.07906977	0.3922424	1	105	tags=40%, l
GOBP_REGULATION_OF_ADAPTIVE_IMMUNE_RESPONSE	18	0.419	1.500	0.08270676	0.39034134	1	117	tags=33%, l
GOCC_ENDOCYTIC_VESICLE_MEMBRANE	16	0.434	1.486	0.10305958	0.41332626	1	151	tags=44%, l
GOMF_UBIQUITIN_LIKE_PROTEIN_LIGASE_ACTIVITY	17	0.407	1.473	0.07819549	0.43525097	1	164	tags=47%, l
REACTOME_SIGNALING_BY_NOTCH	21	0.389	1.471	0.07680945	0.43692735	1	113	tags=33%, l
HP_CONSTIPATION	19	0.403	1.455	0.11145510	0.46556228	1	294	tags=79%, l
GOBP_REGULATION_OF_CELL_CYCLE_G2_M_PHASE_TRANSITION	16	0.414	1.450	0.10697675	0.47233507	1	97	tags=38%, l
HP_RESPIRATORY_TRACT_INFECTION	39	0.323	1.441	0.08909574	0.48700732	1	135	tags=33%, l
GOBP_FC_RECEPTOR_SIGNALING_PATHWAY	20	0.390	1.437	0.12596007	0.49176878	1	166	tags=45%, l
HP_ABNORMAL_INFLAMMATORY_RESPONSE	60	0.286	1.427	0.07808564	0.51013833	1	145	tags=32%, l
GOBP_ADAPTIVE_IMMUNE_RESPONSE_BASED_ON_SOMATIC_RECOMBINATION_C	28	0.346	1.421	0.10085227	0.5190732	1	143	tags=36%, l
GOMF_PROTEIN_DIMERIZATION_ACTIVITY	51	0.291	1.419	0.11612903	0.51969546	1	87	tags=20%, l
REACTOME_SIGNALING_BY_HEDGEHOG	16	0.403	1.419	0.1244168	0.5155525	1	113	tags=38%, l
GOCC_GOLGI_APPARATUS	68	0.269	1.416	0.09741060	0.5174031	1	24	tags=7%, lis
REACTOME_PROGRAMMED_CELL_DEATH	29	0.335	1.414	0.11713031	0.51883817	1	119	tags=31%, l
GOBP_REGULATION_OF_LYMPHOCYTE_ACTIVATION	36	0.321	1.402	0.1184573	0.5405499	1	140	tags=36%, l
GOBP_LYMPHOCYTE_ACTIVATION	57	0.284	1.402	0.10657894	0.53673327	1	145	tags=33%, l
REACTOME_TRANSCRIPTIONAL_REGULATION_BY_RUNX1	16	0.401	1.396	0.13117284	0.54631996	1	183	tags=50%, l
GOBP_RESPONSE_TO_LIPID	47	0.293	1.391	0.12567568	0.5544069	1	35	tags=11%, l
GOBP_TISSUE_HOMEOSTASIS	20	0.364	1.378	0.12721893	0.57954854	1	173	tags=50%, l
GOBP_PROTEIN_LOCALIZATION_TO_NUCLEUS	16	0.397	1.369	0.15975423	0.59796304	1	54	tags=19%, l
GOBP_CELL_CYCLE_G2_M_PHASE_TRANSITION	17	0.387	1.368	0.14264037	0.5944616	1	97	tags=35%, l
GOMF_RIBONUCLEOTIDE_BINDING	92	0.245	1.368	0.11636364	0.59076405	1	168	tags=29%, l
HP_ABNORMALITY_OF_HUMORAL_IMMUNITY	22	0.358	1.364	0.15982404	0.59698814	1	90	tags=32%, l
HP_ABNORMAL_LEUKOCYTE_COUNT	27	0.335	1.359	0.14749263	0.6047667	1	145	tags=37%, l
GOBP_NEGATIVE_REGULATION_OF_RESPONSE_TO_EXTERNAL_STIMULUS	25	0.335	1.357	0.14040114	0.60337365	1	117	tags=32%, l
GOBP_PROTEIN_CONTAINING_COMPLEX_DISASSEMBLY	15	0.389	1.346	0.1710731	0.62726647	1	2	tags=7%, lis
REACTOME_INFECTIOUS_DISEASE	59	0.268	1.344	0.14285715	0.62616765	1	177	tags=34%, l
HP_ABNORMAL_INTESTINE_MORPHOLOGY	34	0.313	1.344	0.15841584	0.62262404	1	140	tags=26%, l
GOBP_PROCESS_UTILIZING_AUTOPHAGIC_MECHANISM	43	0.294	1.343	0.14285715	0.6201392	1	9	tags=7%, lis
GOBP_REGULATION_OF_PROTEIN_CONTAINING_COMPLEX_ASSEMBLY	28	0.323	1.343	0.16450216	0.61612326	1	68	tags=18%, l
REACTOME_TCF_DEPENDENT_SIGNALING_IN_RESPONSE_TO_WNT	15	0.387	1.327	0.16639742	0.65118444	1	97	tags=33%, l
GOBP_NUCLEOSIDE_PHOSPHATE_BIOSYNTHETIC_PROCESS	16	0.372	1.315	0.1770335	0.6784779	1	32	tags=19%, l
GOBP_ORGANELLE_ASSEMBLY	22	0.339	1.313	0.16193181	0.67944497	1	222	tags=50%, l
REACTOME_C_TYPE_LECTIN_RECEPTORS_CLRS	15	0.383	1.312	0.19085173	0.676346	1	97	tags=33%, l
GOMF_MOLECULAR_ADAPTOR_ACTIVITY	15	0.377	1.306	0.19936709	0.68736917	1	80	tags=27%, l
GOBP_CELLULAR_KETONE_METABOLIC_PROCESS	18	0.366	1.300	0.19097744	0.6968323	1	127	tags=39%, l
GOBP_CELLULAR_RESPONSE_TO_DNA_DAMAGE_STIMULUS	53	0.264	1.296	0.16380449	0.70280176	1	155	tags=30%, l
GOBP_HOMEOSTASIS_OF_NUMBER_OF_CELLS	22	0.331	1.289	0.19545455	0.71760374	1	127	tags=32%, l
GOCC_ORGANELLE_SUBCOMPARTMENT	77	0.245	1.284	0.1702396	0.72605973	1	24	tags=6%, lis
GOBP_POSITIVE_REGULATION_OF_SIGNALING	113	0.223	1.276	0.1786543	0.7444606	1	165	tags=28%, l
REACTOME_TRANSCRIPTIONAL_REGULATION_BY_RUNX3	15	0.372	1.275	0.18873668	0.7416845	1	97	tags=33%, l
HP_FUNCTIONAL_ABNORMALITY_OF_THE_GASTROINTESTINAL_TRACT	47	0.268	1.273	0.21017402	0.74082184	1	151	tags=28%, l
GOBP_REGULATION_OF_MYELOID_CELL_DIFFERENTIATION	22	0.336	1.273	0.19672132	0.73602676	1	127	tags=32%, l
REACTOME_MITOTIC_G2_G2_M_PHASES	15	0.370	1.273	0.21091445	0.73280954	1	97	tags=33%, l
REACTOME_MITOTIC_G1_PHASE_AND_G1_S_TRANSITION	15	0.373	1.267	0.20183486	0.7422865	1	97	tags=33%, l
HP_FEVER	24	0.316	1.264	0.21052632	0.7476609	1	292	tags=63%, l
GOMF_PROTEIN_MACROMOLECULE_ADAPTOR_ACTIVITY	15	0.377	1.264	0.21405229	0.74296767	1	80	tags=27%, l
GOBP_NEGATIVE_REGULATION_OF_CELL_POPULATION_PROLIFERATION	48	0.265	1.263	0.2055703	0.74013084	1	37	tags=13%, l
GOBP_LEUKOCYTE_DIFFERENTIATION	45	0.273	1.262	0.21842106	0.73647535	1	127	tags=27%, l
REACTOME_METABOLISM_OF_AMINO_ACIDS_AND_DERIVATIVES	15	0.362	1.254	0.23600605	0.7533072	1	97	tags=33%, l

GOBP_NEGATIVE_REGULATION_OF_PROTEIN_MODIFICATION_PROCESS	39	0.274	1.246	0.20965517	0.7709161	1	184	tags=38%, l
GOMF_ATPASE_ACTIVITY	23	0.322	1.242	0.22334294	0.77754325	1	106	tags=30%, l
GOBP_REGULATION_OF_LEUKOCYTE_DIFFERENTIATION	23	0.315	1.240	0.23580787	0.77763945	1	127	tags=35%, l
GOBP_IMMUNE_RESPONSE_REGULATING_SIGNALING_PATHWAY	35	0.281	1.239	0.19482289	0.77467215	1	101	tags=26%, l
GOBP_REGULATION_OF_PROTEIN_STABILITY	16	0.354	1.236	0.2449923	0.77886736	1	52	tags=13%, l
REACTOME_FC_EPSILON_RECEPTOR_FCERI_SIGNALING	16	0.367	1.235	0.23274478	0.77612823	1	140	tags=38%, l
GOBP_REGULATION_OF_DNA_BINDING_TRANSCRIPTION_FACTOR_ACTIVITY	29	0.290	1.235	0.22720695	0.7720275	1	165	tags=34%, l
HP_ABNORMAL_THROMBOCYTE_MORPHOLOGY	29	0.299	1.234	0.23426062	0.7703363	1	117	tags=31%, l
GOBP_T_CELL_ACTIVATION	37	0.280	1.231	0.225	0.77458125	1	140	tags=32%, l
GOBP_REGULATION_OF_CELL_ACTIVATION	47	0.255	1.230	0.22654155	0.7725081	1	149	tags=34%, l
REACTOME_SIGNALING_BY_THE_B_CELL_RECEPTOR_BCR	16	0.354	1.229	0.23493975	0.7683729	1	133	tags=38%, l
HP_ABNORMALITY_OF_THE_CEREBRAL_VASCULATURE	19	0.333	1.229	0.2336874	0.764463	1	226	tags=58%, l
GOBP_I_KAPPAB_KINASE_NF_KAPPAB_SIGNALING	28	0.294	1.229	0.24823695	0.7602028	1	228	tags=46%, l
REACTOME_APOPTOSIS	25	0.315	1.226	0.26637554	0.7640281	1	97	tags=28%, l
GOBP_LEUKOCYTE_PROLIFERATION	30	0.293	1.224	0.24397163	0.7653928	1	127	tags=30%, l
GOBP_MORPHOGENESIS_OF_AN_EPITHELIUM	27	0.297	1.213	0.23657474	0.7884382	1	97	tags=26%, l
GOBP_REGULATION_OF_CYSTEINE_TYPE_ENDOPEPTIDASE_ACTIVITY	25	0.302	1.212	0.24602026	0.7862694	1	68	tags=20%, l
GOMF_KINASE_REGULATOR_ACTIVITY	21	0.322	1.212	0.25825825	0.78411365	1	148	tags=29%, l
GOBP_REGULATION_OF_PEPTIDASE_ACTIVITY	36	0.274	1.211	0.23595506	0.78155434	1	77	tags=22%, l
REACTOME_SARS_COV_INFECTIONS	18	0.330	1.209	0.27076924	0.78236	1	29	tags=17%, l
GOBP_NEGATIVE_REGULATION_OF_CELL_CELL_ADHESION	16	0.356	1.207	0.2519685	0.78390944	1	138	tags=38%, l
GOBP_NEGATIVE_REGULATION_OF_RESPONSE_TO_STIMULUS	99	0.220	1.205	0.24170616	0.7847075	1	162	tags=29%, l
GOBP_MODIFICATION_DEPENDENT_MACROMOLECULE_CATABOLIC_PROCESS	52	0.248	1.204	0.24779874	0.7831164	1	117	tags=25%, l
GOBP_NEGATIVE_REGULATION_OF_CELL_CYCLE_PROCESS	21	0.316	1.202	0.2764977	0.7824633	1	97	tags=29%, l
GOBP_NEGATIVE_REGULATION_OF_CELL_ACTIVATION	21	0.320	1.197	0.25426358	0.79253083	1	149	tags=38%, l
GOBP_REGULATION_OF_MACROAUTOPHAGY	18	0.335	1.195	0.26636904	0.792078	1	2	tags=6%, lis
GOBP_POSITIVE_REGULATION_OF_PROTEIN_SERINE_THREONINE_KINASE_ACTIV	21	0.311	1.194	0.26666668	0.792254	1	2	tags=5%, lis
GOBP_PROTEIN_PHOSPHOPANTETHEINYLATION	16	0.348	1.193	0.27142859	0.7903606	1	174	tags=50%, l
GOBP_HEMATOPOIETIC_PROGENITOR_CELL_DIFFERENTIATION	20	0.319	1.191	0.28115502	0.7911141	1	165	tags=40%, l
GOMF_ZINC_ION_BINDING	33	0.276	1.191	0.28029197	0.7875345	1	167	tags=36%, l
GOBP_T_CELL_PROLIFERATION	16	0.344	1.188	0.2835131	0.78959113	1	112	tags=31%, l
GOBP_REGULATION_OF_LEUKOCYTE_PROLIFERATION	27	0.297	1.183	0.27738264	0.79980046	1	127	tags=30%, l
GOBP_POSITIVE_REGULATION_OF_GENE_EXPRESSION	78	0.224	1.181	0.26674786	0.80075204	1	37	tags=10%, l
GOBP_NEGATIVE_REGULATION_OF_SIGNALING	85	0.223	1.180	0.24630542	0.79733473	1	162	tags=31%, l
GOBP_REGULATION_OF_CELLULAR_COMPONENT_BIOGENESIS	50	0.246	1.178	0.28552458	0.79851353	1	226	tags=42%, l
GOBP_SECOND_MESSENGER_MEDIATED_SIGNALING	19	0.312	1.177	0.2661654	0.79921556	1	2	tags=5%, lis
GOBP_PEPTIDYL_SERINE_MODIFICATION	23	0.305	1.173	0.2795389	0.80407816	1	2	tags=4%, lis
GOMF_UBIQUITIN_LIKE_PROTEIN_LIGASE_BINDING	30	0.281	1.172	0.30397728	0.8037637	1	136	tags=30%, l
GOBP_REGULATION_OF_CELLULAR_CATABOLIC_PROCESS	73	0.220	1.169	0.27941176	0.8050812	1	120	tags=19%, l
REACTOME_INNATE_IMMUNE_SYSTEM	111	0.208	1.168	0.27020785	0.8038603	1	143	tags=26%, l
HP_ABNORMALITY_OF_TEMPERATURE_REGULATION	29	0.283	1.167	0.29619566	0.80227965	1	292	tags=62%, l
HP_ABNORMALITY_OF_DIGESTIVE_SYSTEM_MORPHOLOGY	39	0.259	1.164	0.29271522	0.80787444	1	140	tags=23%, l
GOBP_NEGATIVE_REGULATION_OF_MULTICELLULAR_ORGANISMAL_PROCESS	64	0.228	1.158	0.2784974	0.81880534	1	136	tags=27%, l
GOMF_ENZYME_INHIBITOR_ACTIVITY	21	0.314	1.156	0.29777777	0.81874204	1	222	tags=52%, l
GOCC_RIBONUCLEOPROTEIN_GRANULE	22	0.304	1.155	0.31432748	0.81788456	1	209	tags=45%, l
GOBP_T_CELL_RECEPTOR_SIGNALING_PATHWAY	18	0.315	1.154	0.31268883	0.8174712	1	97	tags=28%, l
HP_PHENOTYPIC_VARIABILITY	22	0.301	1.153	0.2974589	0.8143797	1	107	tags=27%, l
HALLMARK_APOPTOSIS	25	0.289	1.150	0.30583215	0.81885666	1	174	tags=36%, l
GOBP_NEGATIVE_REGULATION_OF_CYTOKINE_PRODUCTION	27	0.280	1.149	0.29883382	0.8177957	1	75	tags=22%, l
REACTOME_NERVOUS_SYSTEM_DEVELOPMENT	22	0.295	1.147	0.307578	0.81941324	1	157	tags=36%, l
HP_ABNORMALITY_OF_THE_LYMPH_NODES	23	0.297	1.146	0.31746033	0.81659454	1	145	tags=35%, l
GOBP_NEGATIVE_REGULATION_OF_MITOTIC_CELL_CYCLE	17	0.322	1.146	0.31458968	0.81414455	1	97	tags=29%, l
GOBP_NUCLEOBASE_CONTAINING_SMALL_MOLECULE_METABOLIC_PROCESS	40	0.252	1.141	0.3031496	0.82242835	1	176	tags=35%, l
HP_ABNORMAL_HEART_VALVE_PHYSIOLOGY	16	0.329	1.137	0.3323077	0.8284719	1	322	tags=75%, l
GOBP_REGULATION_OF_T_CELL_ACTIVATION	27	0.274	1.134	0.32554516	0.83313143	1	140	tags=33%, l
GOBP_REGULATION_OF_CATABOLIC_PROCESS	83	0.211	1.133	0.30806845	0.83108455	1	120	tags=19%, l
GOBP_DNA_REPAIR	27	0.283	1.133	0.34117648	0.8285203	1	150	tags=33%, l
GOMF_SIGNALING_RECEPTOR_BINDING	77	0.214	1.132	0.32164448	0.82526165	1	90	tags=18%, l
GOBP_POSITIVE_REGULATION_OF_CELL_ADHESION	31	0.268	1.130	0.31266847	0.8266784	1	203	tags=48%, l
GOBP_POSITIVE_REGULATION_OF_MULTICELLULAR_ORGANISMAL_PROCESS	92	0.208	1.128	0.3188406	0.82729393	1	112	tags=20%, l
GOCC_SIDE_OF_MEMBRANE	33	0.263	1.125	0.33002833	0.8312477	1	173	tags=36%, l
GOBP_PRODUCTION_OF_MOLECULAR_MEDIATOR_OF_IMMUNE_RESPONSE	22	0.298	1.125	0.32440478	0.82938045	1	138	tags=32%, l
GOBP_REGULATION_OF_CELL_CELL_ADHESION	33	0.261	1.123	0.32309747	0.83037573	1	140	tags=33%, l
GOBP_RESPONSE_TO_TEMPERATURE_STIMULUS	15	0.328	1.120	0.33384854	0.83306515	1	256	tags=60%, l
GOBP_POSITIVE_REGULATION_OF_LEUKOCYTE_CELL_CELL_ADHESION	21	0.288	1.116	0.3452566	0.84029067	1	140	tags=38%, l
GOBP_POSITIVE_REGULATION_OF_PEPTIDASE_ACTIVITY	22	0.292	1.115	0.33383232	0.8385209	1	77	tags=23%, l
HP_ABNORMAL_IMMUNE_SYSTEM_MORPHOLOGY	41	0.249	1.113	0.32333767	0.83941704	1	145	tags=32%, l
GOBP_CELLULAR_RESPONSE_TO_ABIOTIC_STIMULUS	22	0.283	1.111	0.3548387	0.8398801	1	140	tags=27%, l
GOBP_INFLAMMATORY_RESPONSE	61	0.217	1.110	0.32418302	0.83981144	1	35	tags=8%, lis
GOMF_ENZYME_ACTIVATOR_ACTIVITY	28	0.275	1.107	0.35568514	0.8428395	1	82	tags=18%, l
GOBP_NEGATIVE_REGULATION_OF_GENE_EXPRESSION	95	0.201	1.105	0.3487242	0.8441944	1	209	tags=36%, l
REACTOME_METABOLISM_OF_RNA	33	0.256	1.104	0.34647888	0.84344774	1	116	tags=27%, l
HP_ABNORMAL_CELLULAR_PHENOTYPE	32	0.263	1.104	0.35783634	0.84039664	1	145	tags=34%, l
GOCC_VESICLE_MEMBRANE	56	0.223	1.097	0.35038364	0.8524492	1	246	tags=45%, l
HP_ABNORMALITY_OF_FACIAL_SOFT_TISSUE	21	0.291	1.093	0.33742332	0.8586827	1	140	tags=38%, l
GOBP_REGULATION_OF_B_CELL_ACTIVATION	15	0.320	1.091	0.35168195	0.8601472	1	127	tags=33%, l
GOBP_MYELOID_CELL_DIFFERENTIATION	37	0.244	1.091	0.36760563	0.8568379	1	103	tags=19%, l
REACTOME_SEPARATION_OF_SISTER_CHROMATIDS	16	0.314	1.089	0.36263737	0.8570608	1	135	tags=38%, l
GOCC_NUCLEAR_OUTER_MEMBRANE_ENDOPLASMIC_RETICULUM_MEMBRANE_N	55	0.225	1.089	0.37252313	0.85519725	1	13	tags=5%, lis
GOBP_CELLULAR_RESPONSE_TO_EXTERNAL_STIMULUS	18	0.294	1.083	0.36923078	0.8658799	1	18	tags=11%, l
GOBP_TISSUE_MORPHOGENESIS	30	0.258	1.082	0.3619186	0.8630557	1	97	tags=23%, l

REACTOME_S_PHASE	16	0.310	1.082	0.36176935	0.8599614	1	135	tags=38%, l
HP_VARIABLE_EXPRESSIVITY	18	0.301	1.079	0.36163983	0.8646001	1	280	tags=61%, l
GOBP_REGULATION_OF_MRNA_CATABOLIC_PROCESS	25	0.272	1.077	0.40285715	0.8650232	1	118	tags=28%, l
GOBP_REGULATION_OF_CELLULAR_RESPONSE_TO_STRESS	44	0.227	1.074	0.3643617	0.8697025	1	337	tags=66%, l
GOMF_TRANSCRIPTION_COACTIVATOR_ACTIVITY	18	0.308	1.073	0.3827893	0.8685765	1	291	tags=61%, l
GOBP_CELLULAR_AMINO_ACID_METABOLIC_PROCESS	18	0.296	1.072	0.37750384	0.86670184	1	97	tags=28%, l
GOBP_REGULATION_OF_CELL_CYCLE_PHASE_TRANSITION	26	0.270	1.072	0.37931034	0.8634857	1	97	tags=27%, l
GOCC_ENDOCYTIC_VESICLE	25	0.267	1.069	0.38616714	0.8661489	1	127	tags=28%, l
GOBP_POSITIVE_REGULATION_OF_LEUKOCYTE_PROLIFERATION	17	0.299	1.069	0.37770897	0.86407137	1	274	tags=59%, l
HP_ABNORMAL_RESPIRATORY_SYSTEM_MORPHOLOGY	66	0.206	1.069	0.3660606	0.86091715	1	119	tags=23%, l
GOBP_REGULATION_OF_INFLAMMATORY_RESPONSE	35	0.243	1.067	0.37142858	0.8611987	1	260	tags=54%, l
GOBP_NEGATIVE_REGULATION_OF_CELL_ADHESION	18	0.296	1.066	0.37947884	0.86013246	1	138	tags=33%, l
HP_ABNORMAL_LUNG_MORPHOLOGY	57	0.210	1.064	0.38587642	0.86059016	1	305	tags=56%, l
REACTOME_MAPK_FAMILY_SIGNALING_CASCADES	21	0.282	1.064	0.39734122	0.85761005	1	97	tags=29%, l
GOBP_POSITIVE_REGULATION_OF_I_KAPPAB_KINASE_NF_KAPPAB_SIGNALING	19	0.296	1.064	0.40031645	0.85468596	1	220	tags=47%, l
GOMF_RNA_BINDING	100	0.192	1.063	0.36941177	0.85334855	1	167	tags=27%, l
GOBP_POSITIVE_REGULATION_OF_CELL_CELL_ADHESION	22	0.276	1.060	0.35735735	0.85699654	1	140	tags=36%, l
GOBP_POSITIVE_REGULATION_OF_CYSTEINE_TYPE_ENDOPEPTIDASE_ACTIVITY	17	0.301	1.060	0.38394162	0.85414076	1	62	tags=18%, l
GOCC_EXTERNAL_SIDE_OF_PLASMA_MEMBRANE	22	0.277	1.059	0.37790698	0.8537168	1	173	tags=36%, l
HP_THICKENED_SKIN	18	0.296	1.058	0.42523363	0.8510848	1	254	tags=56%, l
HP_ABNORMALITY_OF_SKIN_PHYSIOLOGY	37	0.235	1.056	0.3900415	0.85207206	1	145	tags=27%, l
GOBP_LEUKOCYTE_CELL_CELL_ADHESION	34	0.239	1.056	0.4089636	0.85084015	1	140	tags=32%, l
GOBP_POSITIVE_REGULATION_OF_CELL_ACTIVATION	31	0.248	1.050	0.41354465	0.8597304	1	140	tags=32%, l
GOCC_MITOCHONDRION	78	0.197	1.050	0.4091456	0.858084	1	35	tags=6%, lis
REACTOME_POST_TRANSLATIONAL_PROTEIN_MODIFICATION	66	0.202	1.045	0.39974937	0.8647579	1	120	tags=26%, l
HP_AUTOIMMUNITY	16	0.297	1.045	0.40791476	0.862125	1	305	tags=63%, l
GOBP_PHAGOCYTOSIS	25	0.259	1.045	0.41063514	0.85976064	1	166	tags=44%, l
GOBP_MACROAUTOPHAGY	27	0.254	1.037	0.41007194	0.8746694	1	2	tags=4%, lis
HP_WEAKNESS_DUE_TO_UPPER_MOTOR_NEURON_DYSFUNCTION	16	0.291	1.036	0.3897281	0.8731582	1	151	tags=44%, l
REACTOME_CELL_CYCLE_CHECKPOINTS	18	0.280	1.035	0.4168013	0.8723311	1	97	tags=28%, l
HP_ABNORMALITY_OF_THE_LYMPHATIC_SYSTEM	48	0.215	1.033	0.41699347	0.8745156	1	145	tags=29%, l
GOBP_NEGATIVE_REGULATION_OF_CELL_DIFFERENTIATION	36	0.233	1.031	0.44170097	0.87455106	1	259	tags=47%, l
GOCC_TRANSFERASE_COMPLEX_TRANSFERRING_PHOSPHORUS_CONTAINING_(	15	0.305	1.029	0.43548387	0.87734807	1	251	tags=60%, l
HP_ABNORMAL_ENZYME_COENZYME_ACTIVITY	19	0.276	1.027	0.44144145	0.8789133	1	73	tags=21%, l
GOBP_NEGATIVE_REGULATION_OF_MOLECULAR_FUNCTION	63	0.203	1.024	0.4362245	0.8815055	1	92	tags=19%, l
GOBP_PROTEIN_LOCALIZATION_TO_ORGANELLE	45	0.220	1.023	0.4216366	0.88031477	1	62	tags=11%, l
HP_HEMATOLOGICAL_NEOPLASM	23	0.260	1.016	0.42481753	0.8920054	1	144	tags=35%, l
HALLMARK_UV_RESPONSE_UP	19	0.275	1.015	0.43909773	0.892568	1	62	tags=16%, l
GOBP_POSITIVE_REGULATION_OF_MOLECULAR_FUNCTION	97	0.182	1.014	0.44852072	0.89146596	1	87	tags=14%, l
HP_WEIGHT_LOSS	22	0.260	1.013	0.44478527	0.88921773	1	226	tags=50%, l
GOMF_CIS_REGULATORY_REGION_SEQUENCE_SPECIFIC_DNA_BINDING	42	0.218	1.012	0.43951613	0.88871384	1	41	tags=10%, l
GOBP_VESICLE_ORGANIZATION	17	0.290	1.012	0.46	0.8869077	1	249	tags=53%, l
GOBP_CELLULAR_COMPONENT_DISASSEMBLY	28	0.248	1.010	0.44680852	0.88674694	1	2	tags=4%, lis
HP_DIARRHEA	18	0.279	1.010	0.44608566	0.88425225	1	90	tags=22%, l
HALLMARK_IL2_STAT5_SIGNALING	20	0.271	1.004	0.45534408	0.8935555	1	180	tags=45%, l
GOBP_NEGATIVE_REGULATION_OF_PROTEIN_METABOLIC_PROCESS	70	0.196	1.003	0.46503067	0.8926225	1	222	tags=39%, l
GOMF_MOLECULAR_TRANSDUCER_ACTIVITY	32	0.230	1.002	0.44846797	0.8923333	1	181	tags=34%, l
GOCC_INTRACELLULAR_PROTEIN_CONTAINING_COMPLEX	51	0.204	0.995	0.4677207	0.9052581	1	275	tags=51%, l
GOCC_PERINUCLEAR_REGION_OF_CYTOPLASM	50	0.207	0.995	0.4794702	0.90216005	1	79	tags=12%, l
GOBP_POSITIVE_REGULATION_OF_NUCLEOBASE_CONTAINING_COMPOUND_MET	105	0.174	0.994	0.46969697	0.90132964	1	305	tags=50%, l
GOBP_POSITIVE_REGULATION_OF_CATABOLIC_PROCESS	44	0.214	0.992	0.46505377	0.9021802	1	9	tags=5%, lis
GOMF_TRANSCRIPTION_COREGULATOR_ACTIVITY	42	0.211	0.982	0.4854369	0.92059976	1	276	tags=48%, l
GOCC_ENDOPLASMIC_RETICULUM	88	0.179	0.982	0.4799514	0.91798913	1	130	tags=20%, l
REACTOME_SIGNALING_BY_WNT	21	0.256	0.980	0.4679878	0.9185362	1	97	tags=24%, l
GOBP_NEGATIVE_REGULATION_OF_WNT_SIGNALING_PATHWAY	22	0.259	0.977	0.49253732	0.92296183	1	97	tags=27%, l
HP_DEVELOPMENTAL_REGRESSION	15	0.291	0.975	0.48181817	0.9251225	1	385	tags=80%, l
HP_ABNORMALITY_OF_MOUTH_SIZE	16	0.283	0.974	0.49068323	0.9232228	1	417	tags=88%, l
GOBP_CELL_CELL_ADHESION	45	0.206	0.974	0.5	0.920355	1	144	tags=31%, l
GOBP_INTRINSIC_APOPTOTIC_SIGNALING_PATHWAY_IN_RESPONSE_TO_DNA_DA	15	0.283	0.972	0.49001536	0.9218982	1	301	tags=60%, l
GOMF_TRANSCRIPTION_REGULATOR_ACTIVITY	83	0.181	0.971	0.5048662	0.91965044	1	29	tags=7%, lis
GOBP_CELLULAR_RESPONSE_TO_OXYGEN_CONTAINING_COMPOUND	77	0.184	0.971	0.49874687	0.9167896	1	47	tags=8%, lis
GOMF_KINASE_BINDING	48	0.204	0.970	0.5171053	0.9168689	1	9	tags=4%, lis
GOBP_REGULATION_OF_CELL_DEATH	101	0.171	0.969	0.50714284	0.9164789	1	174	tags=28%, l
GOBP_POSTTRANSCRIPTIONAL_REGULATION_OF_GENE_EXPRESSION	48	0.204	0.968	0.49736148	0.91458	1	209	tags=38%, l
GOBP_REGULATION_OF_CELL_ADHESION	47	0.208	0.965	0.50476193	0.9183986	1	223	tags=45%, l
GOBP_CELL_CYCLE_PHASE_TRANSITION	30	0.227	0.957	0.4971591	0.93271804	1	97	tags=23%, l
GOCC_ENDOSOME_MEMBRANE	38	0.212	0.954	0.509589	0.9349781	1	247	tags=47%, l
GOMF_DNA_BINDING_TRANSCRIPTION_FACTOR_ACTIVITY	43	0.207	0.953	0.5263158	0.9340974	1	58	tags=12%, l
GOBP_REGULATION_OF_CYTOSKELETON_ORGANIZATION	32	0.217	0.948	0.5118881	0.9420038	1	226	tags=50%, l
GOBP_TUMOR_NECROSIS_FACTOR_SUPERFAMILY_CYTOKINE_PRODUCTION	15	0.272	0.948	0.5169082	0.9400567	1	117	tags=27%, l
REACTOME_M_PHASE	24	0.242	0.947	0.5277383	0.9393331	1	97	tags=25%, l
GOBP_ANIMAL_ORGAN_MORPHOGENESIS	43	0.203	0.945	0.5158184	0.93920106	1	126	tags=23%, l
GOBP_REGULATION_OF_ACTIN_FILAMENT_ORGANIZATION	15	0.277	0.945	0.52821314	0.93651295	1	217	tags=53%, l
GOBP_INTRINSIC_APOPTOTIC_SIGNALING_PATHWAY	32	0.216	0.945	0.51622003	0.93428177	1	170	tags=31%, l
GOBP_NEGATIVE_REGULATION_OF_TRANSFERASE_ACTIVITY	23	0.244	0.945	0.5366569	0.9316589	1	117	tags=30%, l
GOBP_NEGATIVE_REGULATION_OF_ORGANELLE_ORGANIZATION	17	0.263	0.944	0.5360983	0.9308842	1	268	tags=59%, l
REACTOME_INTRACELLULAR_SIGNALING_BY_SECOND_MESSENGERS	24	0.242	0.942	0.5092199	0.931493	1	260	tags=50%, l
GOBP_NEGATIVE_REGULATION_OF_CELL_DEATH	57	0.190	0.940	0.5278481	0.932895	1	162	tags=30%, l
GOBP_NEGATIVE_REGULATION_OF_CANONICAL_WNT_SIGNALING_PATHWAY	20	0.248	0.936	0.516129	0.93754035	1	97	tags=25%, l
HP_ABNORMALITY_OF_THE_BLADDER	22	0.236	0.933	0.5286533	0.9413468	1	256	tags=50%, l
GOBP_POSITIVE_REGULATION_OF_DNA_BINDING_TRANSCRIPTION_FACTOR_ACT	17	0.260	0.932	0.5297619	0.94034165	1	221	tags=41%, l

GOBP_REGULATION_OF_GTPASE_ACTIVITY	16	0.267	0.932	0.5296875	0.9378591	1	477	tags=94%, l
HP_INFLAMMATORY_ABNORMALITY_OF_THE_EYE	18	0.258	0.928	0.5367647	0.9439083	1	119	tags=33%, l
REACTOME_MITOTIC_METAPHASE_AND_ANAPHASE	19	0.254	0.927	0.5102639	0.9425477	1	97	tags=26%, l
GOBP_REGULATION_OF_INTRINSIC_APOPTOTIC_SIGNALING_PATHWAY	19	0.250	0.922	0.54531944	0.95068204	1	162	tags=37%, l
GOBP_POSITIVE_REGULATION_OF_PROTEIN_CONTAINING_COMPLEX_ASSEMBLY	16	0.267	0.919	0.5224551	0.9533374	1	62	tags=19%, l
GOBP_POSITIVE_REGULATION_OF_PROTEIN_KINASE_ACTIVITY	33	0.209	0.918	0.5405028	0.95213723	1	2	tags=3%, lis
GOBP_REGULATION_OF_PROTEOLYSIS	48	0.191	0.917	0.58289474	0.95148367	1	77	tags=17%, l
PID_P53_DOWNSTREAM_PATHWAY	15	0.267	0.916	0.5199362	0.9509372	1	305	tags=60%, l
GOBP_REGULATION_OF_PRODUCTION_OF_MOLECULAR_MEDIATOR_OF_IMMUNE	16	0.267	0.915	0.55079365	0.95081055	1	127	tags=25%, l
GOBP_NUCLEOBASE_CONTAINING_COMPOUND_TRANSPORT	16	0.269	0.914	0.54858	0.94849485	1	116	tags=31%, l
GOBP_RESPONSE_TO_EXTRACELLULAR_STIMULUS	18	0.256	0.914	0.549483	0.9457298	1	213	tags=50%, l
GOBP_POSITIVE_REGULATION_OF_CATALYTIC_ACTIVITY	79	0.170	0.913	0.5770171	0.9460216	1	82	tags=13%, l
GOCC_VACUOLE	62	0.178	0.911	0.5534591	0.94682467	1	233	tags=39%, l
GOBP_POSITIVE_REGULATION_OF_BIOSYNTHETIC_PROCESS	108	0.160	0.910	0.6044341	0.9458343	1	305	tags=49%, l
GOBP_REGULATION_OF_PROTEIN_SERINE_THREONINE_KINASE_ACTIVITY	32	0.212	0.908	0.5781711	0.94652045	1	2	tags=3%, lis
GOCC_VACUOLAR_MEMBRANE	37	0.208	0.906	0.5685752	0.9490224	1	233	tags=38%, l
GOBP_MULTICELLULAR_ORGANISMAL_HOMEOSTASIS	37	0.205	0.905	0.5751724	0.9467453	1	222	tags=43%, l
GOBP_STEM_CELL_DIFFERENTIATION	19	0.250	0.904	0.55151516	0.94688284	1	184	tags=42%, l
GOBP_NEGATIVE_REGULATION_OF_CELL_CYCLE	39	0.203	0.902	0.58068967	0.9481946	1	117	tags=23%, l
GOBP_REGULATION_OF_SMALL_MOLECULE_METABOLIC_PROCESS	29	0.218	0.902	0.587234	0.94606066	1	172	tags=34%, l
HP_THIN_UPPER_LIP_VERMILION	17	0.253	0.899	0.5494012	0.948116	1	383	tags=82%, l
GOBP_MONONUCLEAR_CELL_MIGRATION	17	0.254	0.898	0.56414217	0.9484496	1	173	tags=41%, l
GOCC_INTRINSIC_COMPONENT_OF_ORGANELLE_MEMBRANE	22	0.235	0.894	0.59198815	0.9519467	1	254	tags=45%, l
GOBP_POSITIVE_REGULATION_OF_TRANSFERASE_ACTIVITY	38	0.201	0.894	0.5964674	0.9494736	1	47	tags=8%, lis
GOBP_POSITIVE_REGULATION_OF_IMMUNE_EFFECTOR_PROCESS	22	0.228	0.886	0.5950653	0.9632337	1	143	tags=27%, l
GOBP_REGULATION_OF_PROTEIN_MODIFICATION_PROCESS	97	0.160	0.883	0.6200717	0.9661598	1	140	tags=21%, l
HP_ABNORMALITY_OF_THE_SPLEEN	39	0.195	0.879	0.61148196	0.97103494	1	89	tags=21%, l
GOBP_POSITIVE_REGULATION_OF_PROTEIN_PHOSPHORYLATION	44	0.186	0.879	0.60638297	0.96858007	1	47	tags=7%, lis
GOBP_REGULATION_OF_HYDROLASE_ACTIVITY	72	0.168	0.878	0.6093943	0.96821404	1	86	tags=17%, l
GOMF_CYTOKINE_RECEPTOR_BINDING	16	0.254	0.877	0.6270677	0.9661627	1	78	tags=19%, l
GOBP_REGULATION_OF_CELL_DIFFERENTIATION	98	0.157	0.876	0.6359338	0.9655617	1	112	tags=18%, l
GOMF_TRANSCRIPTION_FACTOR_BINDING	43	0.188	0.875	0.64777327	0.9649067	1	256	tags=42%, l
GOBP_POSITIVE_REGULATION_OF_TRANSCRIPTION_BY_RNA_POLYMERASE_II	67	0.165	0.874	0.61012655	0.96426004	1	41	tags=7%, lis
GOBP_CELL_POPULATION_PROLIFERATION	116	0.151	0.874	0.65172416	0.96225446	1	127	tags=19%, l
GOBP_CHROMATIN_ORGANIZATION	40	0.190	0.870	0.6117166	0.9659333	1	401	tags=70%, l
GOBP_POSITIVE_REGULATION_OF_HEMOPOIESIS	15	0.257	0.867	0.5839874	0.96929294	1	127	tags=33%, l
GOBP_CELLULAR_PROTEIN_CATABOLIC_PROCESS	64	0.170	0.867	0.64450127	0.9667626	1	77	tags=16%, l
GOMF_ENDOPEPTIDASE_ACTIVITY	18	0.241	0.866	0.612462	0.96620333	1	97	tags=22%, l
GOBP_ANATOMICAL_STRUCTURE_HOMEOSTASIS	30	0.210	0.865	0.64295876	0.9646356	1	173	tags=37%, l
HP_ABNORMALITY_OF_THE_VASCULATURE_OF_THE_EYE	22	0.225	0.860	0.5994195	0.9711626	1	106	tags=23%, l
GOBP_NEGATIVE_REGULATION_OF_DEVELOPMENTAL_PROCESS	47	0.182	0.860	0.65171504	0.9694406	1	285	tags=49%, l
GOMF_NUCLEOSIDE_TRIPHOSPHATASE_REGULATOR_ACTIVITY	15	0.259	0.860	0.614897	0.96710634	1	477	tags=93%, l
HP_GLUCOSE_INTOLERANCE	21	0.225	0.858	0.62995595	0.9685168	1	86	tags=19%, l
HP_DYSPHAGIA	19	0.229	0.852	0.61737806	0.97653	1	305	tags=63%, l
GOMF_RNA_POLYMERASE_II_SPECIFIC_DNA_BINDING_TRANSCRIPTION_FACTOR	23	0.223	0.851	0.6276276	0.97422385	1	256	tags=48%, l
GOBP_ORGANOPHOSPHATE_BIOSYNTHETIC_PROCESS	28	0.207	0.850	0.6368638	0.97394085	1	32	tags=11%, l
GOBP_POSITIVE_REGULATION_OF_PHOSPHORUS_METABOLIC_PROCESS	50	0.178	0.850	0.65075034	0.9713118	1	179	tags=26%, l
GOCC_CELL_SURFACE	40	0.182	0.849	0.65147454	0.9708968	1	101	tags=18%, l
REACTOME_DISEASES_OF_SIGNAL_TRANSDUCTION_BY_GROWTH_FACTOR_REC	37	0.191	0.847	0.6414566	0.97167706	1	140	tags=24%, l
GOMF_PEPTIDASE_ACTIVITY	23	0.213	0.846	0.6318052	0.97046113	1	163	tags=30%, l
GOBP_RESPONSE_TO_ORGANIC_CYCLIC_COMPOUND	48	0.177	0.846	0.6496644	0.9688394	1	99	tags=17%, l
GOBP_RESPONSE_TO_ABIOTIC_STIMULUS	78	0.159	0.842	0.68875	0.973359	1	99	tags=17%, l
GOBP_NEGATIVE_REGULATION_OF_NUCLEOBASE_CONTAINING_COMPOUND_ME	78	0.160	0.841	0.6814815	0.97266597	1	29	tags=6%, lis
HP_ABNORMAL_MYELOID_LEUKOCYTE_MORPHOLOGY	19	0.234	0.840	0.6415929	0.97106117	1	145	tags=32%, l
GOBP_PROTEOLYSIS	103	0.149	0.835	0.6861144	0.97824675	1	77	tags=14%, l
HP_ABNORMALITY_OF_REFRACTION	18	0.236	0.835	0.62910795	0.97618574	1	343	tags=72%, l
GOBP_MULTI_ORGANISM_PROCESS	37	0.184	0.834	0.66895604	0.9758596	1	254	tags=43%, l
GOCC_TRANSPORT_VESICLE	19	0.225	0.833	0.6567164	0.97420615	1	539	tags=95%, l
GOBP_CELLULAR_RESPONSE_TO_ORGANIC_CYCLIC_COMPOUND	31	0.190	0.831	0.65229887	0.97486395	1	99	tags=19%, l
GOBP_NEGATIVE_REGULATION_OF_PHOSPHORUS_METABOLIC_PROCESS	35	0.189	0.830	0.66993004	0.97386044	1	179	tags=34%, l
GOBP_DNA_METABOLIC_PROCESS	47	0.173	0.825	0.7043364	0.98153764	1	150	tags=26%, l
HP_INTELLECTUAL_DISABILITY_MILD	18	0.224	0.818	0.6625578	0.98982465	1	294	tags=61%, l
GOBP_APOPTOTIC_PROCESS	135	0.139	0.818	0.7288136	0.9880962	1	162	tags=22%, l
GOBP_BIOLOGICAL_ADHESION	67	0.158	0.817	0.71428573	0.98669666	1	182	tags=33%, l
GOBP_ORGANONITROGEN_COMPOUND_BIOSYNTHETIC_PROCESS	75	0.157	0.815	0.697995	0.9878744	1	32	tags=5%, lis
GOBP_NEGATIVE_REGULATION_OF_INTRACELLULAR_SIGNAL_TRANSDUCTION	38	0.180	0.815	0.68363136	0.9855622	1	170	tags=32%, l
HP_AUTISM	15	0.233	0.812	0.65384614	0.98820394	1	268	tags=60%, l
GOBP_REGULATION_OF_PHOSPHORUS_METABOLIC_PROCESS	88	0.148	0.811	0.73741007	0.9871297	1	223	tags=35%, l
GOMF_ENZYME_REGULATOR_ACTIVITY	63	0.160	0.811	0.7008872	0.9848842	1	148	tags=22%, l
GOBP_REGULATION_OF_ACTIN_FILAMENT_BASED_PROCESS	25	0.204	0.811	0.658606	0.98260295	1	223	tags=48%, l
GOBP_POSITIVE_REGULATION_OF_CELLULAR_COMPONENT_BIOGENESIS	25	0.203	0.809	0.6745213	0.9835218	1	140	tags=28%, l
GOCC_OUTER_MEMBRANE	15	0.229	0.807	0.66820985	0.98482037	1	62	tags=13%, l
HP_PUBERTY_AND_GONADAL_DISORDERS	21	0.213	0.801	0.6868829	0.9910507	1	304	tags=57%, l
GOBP_NEGATIVE_REGULATION_OF_APOPTOTIC_SIGNALING_PATHWAY	17	0.229	0.801	0.66566265	0.988725	1	327	tags=65%, l
GOBP_SIGNAL_TRANSDUCTION_BY_P53_CLASS_MEDIATOR	19	0.222	0.801	0.68237084	0.9870193	1	337	tags=63%, l
GOBP_EPITHELIUM_DEVELOPMENT	45	0.171	0.798	0.71834624	0.99002016	1	194	tags=31%, l
GOBP_SMALL_MOLECULE_METABOLIC_PROCESS	85	0.147	0.797	0.76634383	0.98817915	1	176	tags=27%, l
GOBP_LEUKOCYTE_MIGRATION	42	0.169	0.796	0.7201087	0.9871955	1	177	tags=36%, l
HP_ABNORMALITY_OF_THE_MIDDLE_EAR	25	0.195	0.791	0.69929075	0.9928046	1	46	tags=12%, l
GOMF_TRANSCRIPTION_COREPRESSOR_ACTIVITY	20	0.211	0.789	0.6883309	0.99376535	1	29	tags=10%, l
HP_INCREASED_HEAD_CIRCUMFERENCE	17	0.222	0.789	0.71257484	0.9916575	1	232	tags=53%, l

PID_ERBB1_DOWNSTREAM_PATHWAY	15	0.228	0.789	0.68225807	0.9892038	1	10	tags=7%, lis
HP_LEUKEMIA	16	0.224	0.789	0.70878273	0.9867656	1	144	tags=31%, l
GOMF_PEPTIDE_BINDING	20	0.212	0.786	0.7057143	0.98933554	1	54	tags=15%, l
GOBP_REGULATION_OF_TRANSFERASE_ACTIVITY	62	0.156	0.785	0.7311688	0.98807925	1	165	tags=24%, l
HP_ABNORMALITY_OF_THE_THYROID_GLAND	24	0.195	0.783	0.7254623	0.98846453	1	140	tags=29%, l
GOBP_POSITIVE_REGULATION_OF_PROTEOLYSIS	32	0.180	0.782	0.72649574	0.9880969	1	77	tags=16%, l
GOBP_REGULATION_OF_CELL_CYCLE_PROCESS	38	0.172	0.781	0.73297733	0.98737913	1	151	tags=26%, l
GOBP_NEGATIVE_REGULATION_OF_BIOSYNTHETIC_PROCESS	83	0.144	0.776	0.7652068	0.99302155	1	29	tags=6%, lis
GOBP_NEGATIVE_REGULATION_OF_KINASE_ACTIVITY	21	0.204	0.776	0.7209653	0.9913049	1	117	tags=29%, l
GOBP_NEGATIVE_REGULATION_OF_PHOSPHORYLATION	32	0.179	0.771	0.71954674	0.9958129	1	130	tags=25%, l
GOBP_REGULATION_OF_MITOTIC_CELL_CYCLE	30	0.185	0.771	0.7428161	0.99443656	1	97	tags=20%, l
GOBP_CELLULAR_RESPONSE_TO_OXYGEN_LEVELS	25	0.195	0.769	0.7404255	0.99385303	1	97	tags=20%, l
GOBP_REGULATION_OF_MRNA_METABOLIC_PROCESS	33	0.180	0.768	0.7221439	0.9933346	1	118	tags=21%, l
GOMF_PROTEIN_DOMAIN_SPECIFIC_BINDING	50	0.159	0.768	0.7619048	0.99127275	1	2	tags=2%, lis
GOBP_REGULATION_OF_INTRACELLULAR_TRANSPORT	28	0.185	0.766	0.7245763	0.9912929	1	578	tags=96%, l
GOBP_POSITIVE_REGULATION_OF_ORGANELLE_ORGANIZATION	46	0.162	0.766	0.7479893	0.99006534	1	2	tags=2%, lis
GOBP_RESPONSE_TO_LIGHT_STIMULUS	18	0.217	0.764	0.7462006	0.99078095	1	535	tags=94%, l
GOBP_NEGATIVE_REGULATION_OF_TRANSCRIPTION_BY_RNA_POLYMERASE_II	43	0.162	0.760	0.7584856	0.99365747	1	10	tags=5%, lis
GOBP_ORGANIC_CYCLIC_COMPOUND_CATABOLIC_PROCESS	50	0.157	0.760	0.7526178	0.99241555	1	118	tags=22%, l
GOBP_PEPTIDYL_TYROSINE_MODIFICATION	26	0.193	0.759	0.74101794	0.9911434	1	274	tags=50%, l
REACTOME_SIGNALING_BY_INTERLEUKINS	49	0.161	0.758	0.77453583	0.990212	1	78	tags=14%, l
GOBP_NUCLEOTIDE_PHOSPHORYLATION	18	0.209	0.756	0.7214815	0.9904801	1	172	tags=33%, l
HP_ABNORMALITY_OF_THE_NASAL_TIP	16	0.215	0.750	0.75185734	0.998306	1	380	tags=75%, l
GOBP_NUCLEOSIDE_DIPHOSPHATE_METABOLIC_PROCESS	18	0.209	0.749	0.75346684	0.99693245	1	172	tags=33%, l
GOBP_PROTEIN_CATABOLIC_PROCESS	75	0.143	0.749	0.79327524	0.9948304	1	117	tags=19%, l
GOBP_NEGATIVE_REGULATION_OF_PROTEOLYSIS	15	0.219	0.747	0.75526744	0.9946376	1	8	tags=7%, lis
HP_ABSENT_SPEECH	16	0.217	0.747	0.7540029	0.9927716	1	380	tags=81%, l
GOBP_POSITIVE_REGULATION_OF_PROTEIN_MODIFICATION_PROCESS	61	0.150	0.741	0.8010204	0.99915737	1	148	tags=20%, l
GOBP_NUCLEAR_EXPORT	15	0.216	0.739	0.76236045	0.9998566	1	161	tags=33%, l
GOBP_PROTEASOMAL_PROTEIN_CATABOLIC_PROCESS	33	0.173	0.739	0.75	0.9976696	1	113	tags=21%, l
GOCC_RECEPTOR_COMPLEX	16	0.210	0.737	0.7248428	0.99863183	1	213	tags=44%, l
GOCC_NUCLEAR_SPECK	34	0.172	0.736	0.7656033	0.9968253	1	22	tags=6%, lis
KEGG_PATHWAYS_IN_CANCER	19	0.199	0.735	0.7616893	0.996337	1	68	tags=16%, l
HP_ABNORMAL_EMOTION_AFFECT_BEHAVIOR	27	0.177	0.735	0.7740113	0.99456435	1	268	tags=52%, l
GOBP_ORGANONITROGEN_COMPOUND_CATABOLIC_PROCESS	91	0.133	0.735	0.8115055	0.99230134	1	117	tags=19%, l
GOBP_RNA_CATABOLIC_PROCESS	37	0.164	0.734	0.77119786	0.9908706	1	118	tags=22%, l
GOBP_POSITIVE_REGULATION_OF_PROTEIN_METABOLIC_PROCESS	110	0.128	0.733	0.8278689	0.9906443	1	165	tags=23%, l
HALLMARK_P53_PATHWAY	22	0.192	0.729	0.7540741	0.9928536	1	203	tags=36%, l
GOBP_PROTEIN_CONTAINING_COMPLEX_SUBUNIT_ORGANIZATION	98	0.129	0.727	0.83042973	0.9933603	1	87	tags=12%, l
HP_MYOPIA	16	0.212	0.727	0.7468553	0.99231815	1	453	tags=88%, l
HP_OPTIC_ATROPHY	23	0.188	0.726	0.76435935	0.99048877	1	421	tags=78%, l
GOBP_POSITIVE_REGULATION_OF_HYDROLASE_ACTIVITY	43	0.156	0.724	0.78629035	0.9910199	1	82	tags=14%, l
GOMF_SEQUENCE_SPECIFIC_DNA_BINDING	61	0.143	0.724	0.82586426	0.9892458	1	18	tags=5%, lis
GOBP_REGULATION_OF_LEUKOCYTE_MEDIATED_IMMUNITY	20	0.194	0.722	0.77678573	0.98917127	1	87	tags=15%, l
GOBP_POSITIVE_REGULATION_OF_CELL_POPULATION_PROLIFERATION	60	0.140	0.722	0.8190955	0.98717487	1	127	tags=20%, l
GOBP_PROTEIN_TARGETING	19	0.194	0.722	0.76788324	0.9857486	1	558	tags=95%, l
GOBP_APOPTOTIC_MITOCHONDRIAL_CHANGES	20	0.193	0.717	0.782344	0.99047494	1	62	tags=15%, l
HP_FEEDING_DIFFICULTIES_IN_INFANCY	24	0.177	0.715	0.78971255	0.98985034	1	453	tags=83%, l
GOMF_DNA_BINDING_TRANSCRIPTION_FACTOR_BINDING	27	0.175	0.715	0.7977685	0.9877133	1	86	tags=15%, l
GOCC_RIBONUCLEOPROTEIN_COMPLEX	29	0.172	0.715	0.79275364	0.98648936	1	209	tags=41%, l
GOBP_MYELOID_LEUKOCYTE_DIFFERENTIATION	24	0.180	0.714	0.80030257	0.98557967	1	127	tags=21%, l
HP_NEOPLASM	48	0.148	0.708	0.80052495	0.99050987	1	194	tags=31%, l
GOBP_B_CELL_ACTIVATION	28	0.170	0.707	0.7879656	0.99020463	1	145	tags=25%, l
REACTOME_CELL_CYCLE	38	0.157	0.705	0.82118297	0.99065316	1	97	tags=18%, l
GOBP_PEPTIDYL_LYSINE_MODIFICATION	26	0.175	0.699	0.80171186	0.99561435	1	549	tags=92%, l
GOBP_REGULATION_OF_REACTIVE_OXYGEN_SPECIES_METABOLIC_PROCESS	19	0.192	0.696	0.820208	0.9974832	1	223	tags=42%, l
GOBP_REGULATION_OF_CELL_CYCLE	68	0.134	0.695	0.8329208	0.9962747	1	161	tags=25%, l
GOBP_POSITIVE_REGULATION_OF_CELL_DEATH	53	0.142	0.689	0.8472585	1	1	62	tags=11%, l
GOMF_CELL_ADHESION_MOLECULE_BINDING	35	0.154	0.684	0.86103153	1	1	14	tags=6%, lis
GOMF_ENZYME_BINDING	130	0.118	0.679	0.90102386	1	1	14	tags=3%, lis
GOBP_NEGATIVE_REGULATION_OF_CATALYTIC_ACTIVITY	42	0.146	0.678	0.84965515	1	1	131	tags=21%, l
GOBP_DNA_CONFORMATION_CHANGE	21	0.183	0.675	0.8338323	1	1	106	tags=19%, l
GOBP_REGULATION_OF_PROTEIN_KINASE_ACTIVITY	52	0.138	0.675	0.85045516	1	1	2	tags=2%, lis
GOBP_RNA_LOCALIZATION	16	0.197	0.675	0.82039577	1	1	116	tags=25%, l
GOBP_REGULATION_OF_INTRACELLULAR_PROTEIN_TRANSPORT	23	0.173	0.672	0.8278932	1	1	578	tags=96%, l
GOBP_HEAD_DEVELOPMENT	31	0.160	0.672	0.8268956	1	1	301	tags=58%, l
HP_ABNORMAL_GLUCOSE_HOMEOSTASIS	28	0.161	0.667	0.8443804	1	1	86	tags=14%, l
PID_PDGFRB_PATHWAY	17	0.188	0.662	0.85714287	1	1	10	tags=6%, lis
GOBP_MEMBRANE_ORGANIZATION	56	0.135	0.660	0.87690353	1	1	62	tags=11%, l
HP_ABNORMAL_CARDIAC_ATRIUM_MORPHOLOGY	19	0.184	0.656	0.845706	1	1	10	tags=5%, lis
GOBP_NUCLEAR_TRANSPORT	22	0.170	0.652	0.85692066	1	1	306	tags=55%, l
GOCC_CHROMATIN	49	0.134	0.647	0.87614083	1	1	310	tags=51%, l
GOBP_RESPONSE_TO_OXYGEN_LEVELS	37	0.147	0.646	0.86280054	1	1	170	tags=27%, l
GOBP_APOPTOTIC_SIGNALING_PATHWAY	54	0.130	0.642	0.88073397	1	1	327	tags=52%, l
HP_ABDOMINAL_PAIN	16	0.188	0.638	0.85692066	1	1	222	tags=44%, l
GOCC_INTRINSIC_COMPONENT_OF_PLASMA_MEMBRANE	43	0.137	0.637	0.8667582	1	1	188	tags=30%, l
GOCC_NUCLEAR_PROTEIN_CONTAINING_COMPLEX	50	0.136	0.636	0.8761651	1	1	256	tags=42%, l
GOBP_LEUKOCYTE_MEDIATED_IMMUNITY	83	0.121	0.635	0.91314834	1	1	143	tags=22%, l
GOMF_CATALYTIC_ACTIVITY_ACTING_ON_RNA	15	0.184	0.632	0.85365856	1	1	75	tags=20%, l
HP_ABNORMALITY_OF_BONE_MARROW_CELL_MORPHOLOGY	15	0.183	0.631	0.87761676	1	1	445	tags=80%, l
GOBP_REGULATION_OF_DNA_METABOLIC_PROCESS	22	0.163	0.628	0.87334317	1	1	276	tags=50%, l

GOCC_CENTROSOME	24	0.160	0.628	0.86008835	1	1	104	tags=17%, l
GOBP_PROTEIN_DEPHOSPHORYLATION	16	0.180	0.627	0.8640625	1	1	186	tags=38%, l
GOBP_RESPONSE_TO_PEPTIDE_HORMONE	26	0.154	0.627	0.88728327	1	1	47	tags=8%, lis
HP_ABNORMALITY_OF_UPPER_LIP_VERMILLION	21	0.164	0.626	0.8827786	1	1	294	tags=57%, l
GOBP_REGULATION_OF_PEPTIDYL_TYROSINE_PHOSPHORYLATION	22	0.165	0.625	0.8757143	1	1	132	tags=23%, l
GOBP_REGULATION_OF_ORGANELLE_ORGANIZATION	79	0.116	0.621	0.9041262	1	1	268	tags=41%, l
GOBP_CELLULAR_MACROMOLECULE_CATABOLIC_PROCESS	92	0.114	0.619	0.9357907	1	1	118	tags=17%, l
GOBP_DEPHOSPHORYLATION	19	0.168	0.618	0.88529414	1	1	186	tags=37%, l
HP_ABNORMALITY_OF_THE_BILIARY_SYSTEM	15	0.180	0.615	0.884984	1	1	87	tags=20%, l
GOBP_MYELOID_LEUKOCYTE_MIGRATION	20	0.167	0.615	0.8810642	1	1	138	tags=30%, l
GOBP_POSITIVE_REGULATION_OF_INTRACELLULAR_PROTEIN_TRANSPORT	20	0.162	0.610	0.90332323	1	1	578	tags=95%, l
GOBP_ESTABLISHMENT_OF_PROTEIN_LOCALIZATION_TO_MEMBRANE	21	0.165	0.609	0.8947368	1	1	74	tags=14%, l
GOBP_CELLULAR_MACROMOLECULE_LOCALIZATION	96	0.109	0.608	0.9310345	1	1	86	tags=10%, l
HP_ABNORMALITY_OF_THE_DENTITION	39	0.134	0.603	0.8888889	1	1	439	tags=77%, l
GOBP_REGULATION_OF_APOPTOTIC_SIGNALING_PATHWAY	28	0.149	0.602	0.9154728	1	1	162	tags=29%, l
GOBP_POSITIVE_REGULATION_OF_INTRACELLULAR_TRANSPORT	20	0.162	0.599	0.88971686	1	1	578	tags=95%, l
GOBP_ESTABLISHMENT_OF_PROTEIN_LOCALIZATION_TO_ORGANELLE	30	0.142	0.598	0.88345325	1	1	550	tags=90%, l
GOBP_COVALENT_CHROMATIN_MODIFICATION	29	0.143	0.598	0.8851064	1	1	473	tags=79%, l
GOCC_CELL_LEADING_EDGE	20	0.158	0.596	0.89751554	1	1	166	tags=30%, l
HP_DYSARTHRIA	15	0.174	0.596	0.87304074	1	1	165	tags=33%, l
HP_ABNORMALITY_OF_THE_INNER_EAR	42	0.130	0.596	0.9185083	1	1	445	tags=76%, l
GOBP_REGULATION_OF_PROTEIN_LOCALIZATION	54	0.119	0.593	0.92268044	1	1	11	tags=4%, lis
HP_ABNORMAL_NUMBER_OF_TEETH	20	0.158	0.588	0.9308271	1	1	439	tags=80%, l
GOBP_RESPONSE_TO_MECHANICAL_STIMULUS	15	0.169	0.586	0.8861538	1	1	18	tags=7%, lis
GOBP_PEPTIDYL_AMINO_ACID_MODIFICATION	75	0.113	0.585	0.9281364	1	1	276	tags=39%, l
HP_EXTERNAL_GENITAL_HYPOPLASIA	18	0.157	0.582	0.9040881	1	1	471	tags=83%, l
HP_DISINHIBITION	15	0.170	0.580	0.9128368	1	1	404	tags=73%, l
HP_INFANTILE_ONSET	22	0.150	0.576	0.93118596	1	1	387	tags=68%, l
REACTOME_RNA_POLYMERASE_II_TRANSCRIPTION	70	0.112	0.576	0.94625	1	1	97	tags=14%, l
GOBP_RESPONSE_TO_DRUG	16	0.161	0.571	0.9100775	1	1	224	tags=44%, l
HP_HYPOPLASIA_OF_PENIS	18	0.157	0.565	0.9159159	1	1	471	tags=83%, l
HP_ABNORMAL_PYRAMIDAL_SIGN	15	0.165	0.562	0.922956	1	1	140	tags=27%, l
GOBP_INTRACELLULAR_TRANSPORT	87	0.102	0.562	0.947619	1	1	267	tags=39%, l
HP_ABNORMAL_CARDIAC_SEPTUM_MORPHOLOGY	29	0.136	0.560	0.9366906	1	1	436	tags=76%, l
GOBP_MITOCHONDRIAL_TRANSPORT	23	0.142	0.555	0.9306785	1	1	601	tags=96%, l
GOCC_AXON	23	0.141	0.554	0.94056463	1	1	10	tags=4%, lis
GOBP_NEGATIVE_REGULATION_OF_CELLULAR_COMPONENT_ORGANIZATION	38	0.126	0.553	0.9230769	1	1	268	tags=47%, l
GOBP_ACTIN_FILAMENT_ORGANIZATION	26	0.137	0.551	0.93140244	1	1	217	tags=38%, l
GOBP_ORGANIC_ACID_METABOLIC_PROCESS	47	0.118	0.550	0.9346939	1	1	172	tags=26%, l
HP_TREMOR	33	0.125	0.538	0.9385475	1	1	278	tags=45%, l
GOBP_RESPONSE_TO_HORMONE	46	0.113	0.536	0.95	1	1	47	tags=7%, lis
GOBP_LEUKOCYTE_CHEMOTAXIS	17	0.152	0.530	0.94526625	1	1	229	tags=41%, l
GOBP_TRANSMEMBRANE_TRANSPORT	68	0.101	0.528	0.96050954	1	1	124	tags=16%, l
GOBP_MACROMOLECULE_CATABOLIC_PROCESS	108	0.092	0.527	0.9780347	1	1	120	tags=17%, l
HP_ABNORMAL_SYSTEMIC_ARTERIAL_MORPHOLOGY	28	0.129	0.527	0.9381295	1	1	73	tags=11%, l
GOBP_MRNA_METABOLIC_PROCESS	55	0.104	0.525	0.9518856	1	1	251	tags=36%, l
GOCC_CATALYTIC_COMPLEX	89	0.097	0.523	0.97472924	1	1	275	tags=40%, l
GOBP_RESPONSE_TO_METAL_ION	19	0.142	0.515	0.9488722	1	1	211	tags=37%, l
HP_ABNORMALITY_OF_HINDBRAIN_MORPHOLOGY	26	0.125	0.512	0.9554235	1	1	315	tags=54%, l
GOCC_NUCLEAR_BODY	53	0.104	0.509	0.9715026	1	1	22	tags=4%, lis
HP_ABNORMALITY_OF_THE_LENS	36	0.115	0.509	0.9542936	1	1	362	tags=58%, l
GOBP_ENDOSOMAL_TRANSPORT	18	0.137	0.502	0.9670165	1	1	249	tags=44%, l
GOMF_ION_TRANSMEMBRANE_TRANSPORTER_ACTIVITY	27	0.123	0.501	0.9612069	1	1	46	tags=7%, lis
GOCC_SECRETORY_GRANULE_MEMBRANE	27	0.120	0.490	0.96081275	1	1	245	tags=41%, l
GOBP_PURINE_CONTAINING_COMPOUND_METABOLIC_PROCESS	32	0.114	0.486	0.9597122	1	1	176	tags=28%, l
GOCC_ANCHORING_JUNCTION	38	0.107	0.485	0.963365	1	1	151	tags=24%, l
GOCC_CELL_CELL_JUNCTION	18	0.132	0.484	0.9694657	1	1	594	tags=94%, l
HP_ABNORMALITY_OF_THE_CEREBRAL_SUBCORTEX	42	0.104	0.479	0.98687667	1	1	268	tags=40%, l
GOCC_MICROTUBULE_ORGANIZING_CENTER	30	0.114	0.478	0.97350067	1	1	41	tags=7%, lis
HP_PNEUMONIA	16	0.139	0.476	0.9586524	1	1	135	tags=25%, l
GOBP_REGULATION_OF_MITOCHONDRION_ORGANIZATION	22	0.122	0.465	0.9652677	1	1	321	tags=55%, l
GOBP_OSSIFICATION	24	0.116	0.458	0.97333336	1	1	14	tags=4%, lis
GOBP_AGING	16	0.132	0.455	0.9767442	1	1	508	tags=88%, l
HP_ABNORMALITY_OF_MUSCLE_SIZE	29	0.108	0.453	0.980663	1	1	302	tags=48%, l
GOBP_PYRUVATE_METABOLIC_PROCESS	17	0.127	0.450	0.9878604	1	1	172	tags=29%, l
HP_ABNORMAL_BONE_STRUCTURE	33	0.103	0.448	0.97550434	1	1	10	tags=3%, lis
HP_ABNORMAL_ANTERIOR_EYE_SEGMENT_MORPHOLOGY	53	0.090	0.431	0.98275864	1	1	87	tags=11%, l
REACTOME_PROCESSING_OF_CAPPED_INTRON_CONTAINING_PRE_MRNA	15	0.131	0.430	0.990566	1	1	116	tags=20%, l
GOBP_INTRACELLULAR_PROTEIN_TRANSPORT	63	0.084	0.424	0.9873578	1	1	671	tags=98%, l
GOBP_VACUOLAR_TRANSPORT	16	0.121	0.423	0.9921753	0.9999449	1	265	tags=44%, l
GOCC_TRANSFERASE_COMPLEX	51	0.085	0.415	0.992126	0.9996515	1	328	tags=51%, l
GOBP_POSITIVE_REGULATION_OF_MITOCHONDRION_ORGANIZATION	17	0.118	0.406	0.99244714	0.99943656	1	62	tags=12%, l
HP_VENTRICULOMEGALY	24	0.102	0.405	0.9927536	0.9977512	1	322	tags=54%, l
GOBP_CHROMOSOME_ORGANIZATION	59	0.078	0.394	0.99749684	0.99761254	1	307	tags=46%, l
REACTOME_DEVELOPMENTAL_BIOLOGY	42	0.082	0.380	0.99210525	0.99749506	1	182	tags=24%, l
GOBP_ESTABLISHMENT_OF_PROTEIN_LOCALIZATION	103	0.063	0.356	1	0.99775565	1	86	tags=9%, lis
