# Supplementary material for: Biodegradable nanoparticles induce cGAS/STING-dependent reprogramming of myeloid cells to promote tumor immunotherapy
Source: Front Immunol. 2022 Aug 18;13:887649. doi: 10.3389/fimmu.2022.887649 (PMC9433741; doi:10.3389/fimmu.2022.887649)
Supplement: Supplementary file 9 [file Table_3.pdf]

Supplemental Table 3. Signaling Pathway Analysis for All Myeloid Cells - 3 Doses Once Every 3 Days - ONP-302 vs. Saline

NAME	SIZE	ES	NES	NOM p-val	FDR q-val	FWER p-va	RANK AT M	LEADING E
HALLMARK_MYC_TARGETS_V1	40	0.664	3.229	0	0	0	320	tags=83%,
GOCC_INNER_MITOCHONDRIAL_MEMBRANE_PROTEIN_COMPLEX	34	0.639	3.027	0	0	0	378	tags=88%,
KEGG_PARKINSONS_DISEASE	35	0.638	3.019	0	0	0	463	tags=100%
KEGG_OXIDATIVE_PHOSPHORYLATION	34	0.639	3.008	0	0	0	378	tags=88%,
REACTOME_RESPIRATORY_ELECTRON_TRANSPORT_ATP_SYNTHESIS_BY_CHEMI	36	0.644	3.007	0	0	0	378	tags=89%,
GOBP_OXIDATIVE_PHOSPHORYLATION	40	0.614	3.003	0	0	0	378	tags=85%,
KEGG_HUNTINGTONS_DISEASE	44	0.590	2.969	0	0	0	463	tags=93%,
GOCC_MITOCHONDRIAL_PROTEIN_CONTAINING_COMPLEX	42	0.590	2.952	0	0	0	378	tags=81%,
HALLMARK_OXIDATIVE_PHOSPHORYLATION	43	0.555	2.811	0	0	0	470	tags=91%,
REACTOME_SIGNALING_BY_THE_B_CELL_RECEPTOR_BCR	26	0.645	2.792	0	0	0	111	tags=50%,
KEGG_ANTIGEN_PROCESSING_AND_PRESENTATION	16	0.747	2.776	0	0	0	212	tags=81%,
REACTOME_THE_CITRIC_ACID_TCA_CYCLE_AND_RESPIRATORY_ELECTRON_TRA	40	0.557	2.724	0	8.68E-05	0.001	463	tags=93%,
REACTOME_AUF1_HNRNP_D0_BINDS_AND_DESTABILIZES_MRNA	17	0.710	2.713	0	8.02E-05	0.001	332	tags=94%,
GOCC_RESPIRASOME	26	0.633	2.690	0	7.44E-05	0.001	463	tags=100%
GOBP_ANTIGEN_PROCESSING_AND_PRESENTATION_OF_PEPTIDE_ANTIGEN	39	0.556	2.686	0	6.95E-05	0.001	332	tags=79%,
GOBP_ANTIGEN_RECEPTOR_MEDIATED_SIGNALING_PATHWAY	42	0.535	2.672	0	6.51E-05	0.001	111	tags=43%,
REACTOME_THE_ROLE_OF_GTSE1_IN_G2_M_PROGRESSION_AFTER_G2_CHECKI	17	0.709	2.668	0	6.13E-05	0.001	332	tags=94%,
GOCC_RESPIRATORY_CHAIN_COMPLEX	24	0.632	2.667	0	5.79E-05	0.001	463	tags=100%
GOBP_ATP_SYNTHESIS_COUPLED_ELECTRON_TRANSPORT	29	0.599	2.658	0	1.08E-04	0.002	463	tags=97%,
REACTOME_ABC_FAMILY_PROTEINS_MEDIATED_TRANSPORT	18	0.685	2.646	0	1.02E-04	0.002	332	tags=89%,
GOBP_ANTIGEN_PROCESSING_AND_PRESENTATION	43	0.527	2.645	0	9.76E-05	0.002	332	tags=77%,
GOBP_RESPIRATORY_ELECTRON_TRANSPORT_CHAIN	29	0.599	2.636	0	9.31E-05	0.002	463	tags=97%,
REACTOME_MITOTIC_G2_G2_M_PHASES	25	0.609	2.631	0	8.91E-05	0.002	342	tags=80%,
GOBP_ESTABLISHMENT_OF_TISSUE_POLARITY	17	0.694	2.612	0	8.54E-05	0.002	332	tags=94%,
REACTOME_RESPIRATORY_ELECTRON_TRANSPORT	26	0.633	2.611	0	8.20E-05	0.002	463	tags=100%
KEGG_PROTEASOME	15	0.736	2.611	0	7.88E-05	0.002	332	tags=100%
GOBP_ATP_METABOLIC_PROCESS	51	0.500	2.594	0	1.15E-04	0.003	463	tags=84%,
REACTOME_REGULATION_OF_RUNX2_EXPRESSION_AND_ACTIVITY	17	0.678	2.593	0	1.11E-04	0.003	332	tags=88%,
REACTOME_ORC1_REMOVAL_FROM_CHROMATIN	15	0.705	2.591	0	1.07E-04	0.003	332	tags=93%,
GOBP_CELLULAR_RESPIRATION	36	0.546	2.576	0	1.38E-04	0.004	478	tags=92%,
GOMF_PROTON_TRANSMEMBRANE_TRANSPORTER_ACTIVITY	20	0.655	2.575	0	1.34E-04	0.004	340	tags=90%,
GOBP_B_CELL_RECEPTOR_SIGNALING_PATHWAY	18	0.668	2.573	0	1.30E-04	0.004	100	tags=50%,
REACTOME_NEGATIVE_REGULATION_OF_NOTCH4_SIGNALING	15	0.705	2.573	0	1.26E-04	0.004	332	tags=93%,
REACTOME_DNA_REPLICATION	17	0.670	2.570	0	1.22E-04	0.004	332	tags=88%,
GOBP_TUMOR_NECROSIS_FACTOR_MEDIATED_SIGNALING_PATHWAY	20	0.650	2.562	0	1.18E-04	0.004	332	tags=85%,
GOBP_PROTON_TRANSMEMBRANE_TRANSPORT	23	0.622	2.560	0	1.15E-04	0.004	340	tags=83%,
GOBP_MORPHOGENESIS_OF_A_POLARIZED_EPITHELIUM	20	0.644	2.558	0	1.12E-04	0.004	332	tags=85%,
REACTOME_SWITCHING_OF_ORIGINS_TO_A_POST_REPLICATIVE_STATE	15	0.705	2.557	0	1.09E-04	0.004	332	tags=93%,
REACTOME_TRANSLATION	26	0.584	2.557	0	1.06E-04	0.004	387	tags=81%,
GOBP_NEGATIVE_REGULATION_OF_CELL_CYCLE_G2_M_PHASE_TRANSITION	18	0.685	2.557	0	1.04E-04	0.004	332	tags=89%,
REACTOME_ASSEMBLY_OF_THE_PRE_REPLICATIVE_COMPLEX	15	0.705	2.556	0	1.01E-04	0.004	332	tags=93%,
REACTOME_METABOLISM_OF_RNA	59	0.470	2.543	0	1.24E-04	0.005	486	tags=85%,
REACTOME_HEDGEHOG_LIGAND_BIOGENESIS	17	0.681	2.543	0	1.21E-04	0.005	332	tags=88%,
REACTOME_DEGRADATION_OF_DVL	15	0.705	2.543	0	1.19E-04	0.005	332	tags=93%,
GOBP_REGULATION_OF_CELL_CYCLE_G2_M_PHASE_TRANSITION	24	0.599	2.540	0	1.16E-04	0.005	332	tags=79%,
REACTOME_APC_C_MEDIATED_DEGRADATION_OF_CELL_CYCLE_PROTEINS	15	0.705	2.537	0	1.37E-04	0.006	332	tags=93%,
REACTOME_S_PHASE	21	0.613	2.537	0	1.34E-04	0.006	342	tags=81%,
REACTOME_MHC_CLASS_II_ANTIGEN_PRESENTATION	16	0.673	2.537	0	1.31E-04	0.006	221	tags=81%,
REACTOME_TRANSCRIPTIONAL_REGULATION_BY_RUNX2	20	0.625	2.535	0	1.28E-04	0.006	332	tags=80%,
REACTOME_APC_C_CDH1_MEDIATED_DEGRADATION_OF_CDC20_AND_OTHER_AI	15	0.705	2.535	0	1.26E-04	0.006	332	tags=93%,
REACTOME_ASYMMETRIC_LOCALIZATION_OF_PCP_PROTEINS	15	0.705	2.535	0	1.23E-04	0.006	332	tags=93%,
GOMF_UNFOLDED_PROTEIN_BINDING	17	0.658	2.514	0	1.61E-04	0.008	214	tags=71%,
REACTOME_G2_M_CHECKPOINTS	20	0.626	2.511	0	1.58E-04	0.008	396	tags=90%,
REACTOME_REGULATION_OF_RUNX3_EXPRESSION_AND_ACTIVITY	16	0.677	2.508	0	1.55E-04	0.008	332	tags=88%,
REACTOME_PCP_CE_PATHWAY	20	0.635	2.501	0	1.72E-04	0.009	332	tags=85%,
REACTOME_DECTIN_1_MEDIATED_NONCANONICAL_NF_KB_SIGNALING	15	0.705	2.499	0	1.69E-04	0.009	332	tags=93%,
REACTOME_DEGRADATION_OF_AXIN	15	0.705	2.497	0	1.66E-04	0.009	332	tags=93%,
GOBP_NUCLEOSIDE_TRIPHOSPHATE_METABOLIC_PROCESS	16	0.690	2.496	0	1.63E-04	0.009	304	tags=88%,
REACTOME_DNA_REPLICATION_PRE_INITIATION	15	0.705	2.495	0	1.60E-04	0.009	332	tags=93%,
GOBP_ANTIGEN_PROCESSING_AND_PRESENTATION_OF_PEPTIDE_ANTIGEN_VIA_	26	0.565	2.492	0	1.57E-04	0.009	332	tags=81%,
REACTOME_SCF_SKP2_MEDIATED_DEGRADATION_OF_P27_P21	16	0.678	2.491	0	1.55E-04	0.009	332	tags=88%,
REACTOME_DEGRADATION_OF_GLI1_BY_THE_PROTEASOME	16	0.666	2.478	0	1.86E-04	0.011	332	tags=88%,
GOCC_RIBONUCLEOPROTEIN_COMPLEX	61	0.443	2.474	0	1.83E-04	0.011	486	tags=77%,
REACTOME_FCERI_MEDIATED_NF_KB_ACTIVATION	17	0.652	2.470	0	1.80E-04	0.011	332	tags=88%,
REACTOME_REGULATION_OF_RAS_BY_GAPS	16	0.678	2.468	0	1.77E-04	0.011	332	tags=88%,
GOBP_MITOCHONDRIAL_TRANSMEMBRANE_TRANSPORT	18	0.650	2.465	0	1.74E-04	0.011	304	tags=83%,
REACTOME_STABILIZATION_OF_P53	17	0.647	2.460	0	1.88E-04	0.012	332	tags=82%,
GOCC_ENDOPEPTIDASE_COMPLEX	16	0.675	2.458	0	1.85E-04	0.012	332	tags=88%,
REACTOME_CYCLIN_A_CDK2_ASSOCIATED_EVENTS_AT_S_PHASE_ENTRY	18	0.637	2.458	0	1.82E-04	0.012	342	tags=83%,
REACTOME_SIGNALING_BY_HEDGEHOG	22	0.577	2.458	0	1.80E-04	0.012	332	tags=73%,
REACTOME_ABC_TRANSPORTER_DISORDERS	16	0.678	2.455	0	1.77E-04	0.012	332	tags=88%,
GOCC_PEPTIDASE_COMPLEX	17	0.655	2.451	0	1.75E-04	0.012	399	tags=94%,
GOCC_MITOCHONDRIAL_ENVELOPE	75	0.427	2.444	0	2.01E-04	0.014	483	tags=76%,
REACTOME_DOWNSTREAM_SIGNALING_EVENTS_OF_B_CELL_RECEPTOR_BCR	19	0.621	2.440	0	1.99E-04	0.014	332	tags=79%,
REACTOME_CELLULAR_RESPONSE_TO_HYPOXIA	17	0.651	2.440	0	1.96E-04	0.014	332	tags=82%,
GOCC_ENDOPLASMIC_RETICULUM_PROTEIN_CONTAINING_COMPLEX	22	0.589	2.440	0	1.93E-04	0.014	478	tags=95%,
REACTOME_METABOLISM_OF_POLYAMINES	16	0.655	2.431	0	2.05E-04	0.015	332	tags=88%,
GOBP_CELL_CYCLE_G2_M_PHASE_TRANSITION	28	0.554	2.428	0	2.16E-04	0.016	332	tags=71%,

REACTOME_DEFECTIVE_CFTR_CAUSES_CYSTIC_FIBROSIS	16	0.678	2.426	0	2.13E-04	0.016	332	tags=88%,
HALLMARK_INTERFERON_GAMMA_RESPONSE	48	0.467	2.424	0	2.10E-04	0.016	235	tags=52%,
REACTOME_MITOTIC_G1_PHASE_AND_G1_S_TRANSITION	22	0.595	2.423	0	2.20E-04	0.017	342	tags=77%,
GOBP_MITOCHONDRIAL_MEMBRANE_ORGANIZATION	23	0.587	2.421	0	2.30E-04	0.018	411	tags=87%,
REACTOME_REGULATION_OF_PTEN_STABILITY_AND_ACTIVITY	16	0.652	2.420	0	2.27E-04	0.018	332	tags=88%,
REACTOME_G1_S_DNA_DAMAGE_CHECKPOINTS	18	0.624	2.410	0	2.87E-04	0.023	332	tags=78%,
REACTOME_TNFR2_NON_CANONICAL_NF_KB_PATHWAY	16	0.663	2.405	0	3.08E-04	0.025	332	tags=88%,
GOBP_PURINE_CONTAINING_COMPOUND_BIOSYNTHETIC_PROCESS	17	0.640	2.404	0	3.04E-04	0.025	304	tags=76%,
GOBP_ANTIGEN_PROCESSING_AND_PRESENTATION_OF_EXOGENOUS_PEPTIDE_	25	0.559	2.404	0	3.01E-04	0.025	332	tags=80%,
REACTOME_HOST_INTERACTIONS_OF_HIV_FACTORS	27	0.559	2.403	0	2.98E-04	0.025	332	tags=67%,
GOBP_ANAPHASE_PROMOTING_COMPLEX_DEPENDENT_CATABOLIC_PROCESS	16	0.653	2.401	0.00239808	3.18E-04	0.027	332	tags=88%,
REACTOME_SEPARATION_OF_SISTER_CHROMATIDS	19	0.604	2.394	0	3.14E-04	0.027	341	tags=84%,
REACTOME_CYTOPROTECTION_BY_HMOX1	34	0.506	2.388	0	3.34E-04	0.029	340	tags=71%,
GOBP_REGULATION_OF_HEMATOPOIETIC_STEM_CELL_DIFFERENTIATION	16	0.645	2.386	0	3.30E-04	0.029	332	tags=88%,
REACTOME_UCH_PROTEINASES	19	0.606	2.384	0	3.38E-04	0.03	332	tags=79%,
REACTOME_HEDGEHOG_ON_STATE	17	0.625	2.384	0	3.34E-04	0.03	332	tags=82%,
REACTOME_HEDGEHOG_OFF_STATE	19	0.625	2.378	0	3.52E-04	0.032	332	tags=79%,
REACTOME_RUNX1_REGULATES_TRANSCRIPTION_OF_GENES_INVOLVED_IN_DIF	16	0.664	2.375	0	3.70E-04	0.034	332	tags=88%,
GOBP_POSITIVE_REGULATION_OF_CANONICAL_WNT_SIGNALING_PATHWAY	18	0.615	2.367	0	3.88E-04	0.036	332	tags=78%,
GOBP_B_CELL_MEDIATED_IMMUNITY	17	0.616	2.364	0	3.84E-04	0.036	53	tags=35%,
REACTOME_REGULATION_OF_EXPRESSION_OF_SLITS_AND_ROBOS	17	0.646	2.362	0	4.01E-04	0.038	332	tags=82%,
GOBP_HEMATOPOIETIC_STEM_CELL_DIFFERENTIATION	16	0.645	2.346	0	4.60E-04	0.044	332	tags=88%,
REACTOME_CELLULAR_RESPONSE_TO_CHEMICAL_STRESS	46	0.455	2.329	0	6.02E-04	0.056	340	tags=65%,
GOMF_RNA_BINDING	166	0.343	2.322	0	6.68E-04	0.063	326	tags=46%,
REACTOME_HIV_INFECTION	31	0.502	2.314	0	7.34E-04	0.069	332	tags=65%,
GOBP_NON_CANONICAL_WNT_SIGNALING_PATHWAY	21	0.578	2.314	0	7.27E-04	0.069	365	tags=81%,
GOCC_ORGANELLE_INNER_MEMBRANE	58	0.429	2.303	0	7.49E-04	0.072	483	tags=81%,
GOBP_FC_EPSILON_RECEPTOR_SIGNALING_PATHWAY	23	0.548	2.291	0	8.40E-04	0.082	111	tags=43%,
REACTOME_DEGRADATION_OF_BETA_CATENIN_BY_THE_DESTRUCTION_COMPLE	19	0.598	2.288	0	8.52E-04	0.084	339	tags=79%,
REACTOME_MITOTIC_METAPHASE_AND_ANAPHASE	20	0.573	2.280	0	9.02E-04	0.09	341	tags=80%,
REACTOME_TCF_DEPENDENT_SIGNALING_IN_RESPONSE_TO_WNT	21	0.564	2.274	0	9.42E-04	0.095	332	tags=71%,
REACTOME_SIGNALING_BY_NOTCH4	18	0.597	2.269	0	0.00101849	0.104	332	tags=78%,
GOCC_SPLICEOSOMAL_COMPLEX	22	0.547	2.247	0	0.00132910	0.132	486	tags=91%,
GOBP_REGULATION_OF_B_CELL_ACTIVATION	19	0.573	2.243	0.00220264	0.00138314	0.138	53	tags=32%,
GOBP_POSITIVE_REGULATION_OF_WNT_SIGNALING_PATHWAY	20	0.566	2.239	0.00223214	0.00140790	0.141	332	tags=70%,
GOBP_ELECTRON_TRANSPORT_CHAIN	36	0.477	2.239	0	0.00145037	0.146	463	tags=86%,
REACTOME_DISORDERS_OF_TRANSMEMBRANE_TRANSPORTERS	18	0.563	2.224	0	0.00165473	0.167	332	tags=78%,
REACTOME_BETA_CATENIN_INDEPENDENT_WNT_SIGNALING	28	0.498	2.220	0	0.00177612	0.181	332	tags=64%,
GOBP_ENERGY_DERIVATION_BY_OXIDATION_OF_ORGANIC_COMPOUNDS	45	0.447	2.217	0	0.00183305	0.187	480	tags=84%,
GOCC_VACUOLAR_LUMEN	32	0.477	2.216	0	0.00183455	0.189	113	tags=31%,
REACTOME_REGULATION_OF_MRNA_STABILITY_BY_PROTEINS_THAT_BIND_AU_R	23	0.530	2.210	0	0.00195092	0.202	332	tags=74%,
GOBP_REGULATION_OF_HEMATOPOIETIC_PROGENITOR_CELL_DIFFERENTIATION	18	0.569	2.180	0	0.00274697	0.274	332	tags=78%,
REACTOME_REGULATION_OF_HMOX1_EXPRESSION_AND_ACTIVITY	19	0.571	2.179	0	0.00274150	0.276	332	tags=74%,
GOBP_HEMATOPOIETIC_PROGENITOR_CELL_DIFFERENTIATION	24	0.521	2.178	0	0.00273612	0.278	332	tags=71%,
GOBP_MITOCHONDRIAL_TRANSPORT	30	0.500	2.170	0	0.00302036	0.303	304	tags=63%,
GOCC_BLOOD_MICROPARTICLE	15	0.611	2.163	0	0.00319061	0.322	94	tags=47%,
GOBP_SCF_DEPENDENT_PROTEASOMAL_UBIQUITIN_DEPENDENT_PROTEIN_CAT	18	0.583	2.162	0.00444444	0.00317322	0.323	332	tags=78%,
REACTOME_PROCESSING_OF_CAPPED_INTRON_CONTAINING_PRE_MRNA	26	0.499	2.147	0	0.00363889	0.368	486	tags=88%,
GOBP_TRANSLATIONAL_INITIATION	22	0.510	2.139	0.00232018	0.00381805	0.382	461	tags=82%,
GOBP_B_CELL_ACTIVATION	37	0.445	2.138	0.00235849	0.00383792	0.383	62	tags=24%,
REACTOME_ORGANELLE_BIOGENESIS_AND_MAINTENANCE	23	0.509	2.133	0	0.00392996	0.394	304	tags=65%,
GOBP_ACTIVATION_OF_IMMUNE_RESPONSE	73	0.373	2.133	0	0.00390820	0.395	111	tags=29%,
GOMF_ELECTRON_TRANSFER_ACTIVITY	31	0.475	2.129	0.00220750	0.00410348	0.415	463	tags=87%,
GOBP_PROTEIN_FOLDING	25	0.485	2.128	0	0.00410434	0.418	478	tags=80%,
REACTOME_MRNA_SPLICING	25	0.496	2.126	0.00212765	0.00416868	0.424	486	tags=88%,
REACTOME_UB_SPECIFIC_PROCESSING_PROTEASES	24	0.501	2.126	0.00224719	0.00413757	0.424	332	tags=67%,
GOMF_RIBONUCLEOPROTEIN_COMPLEX_BINDING	16	0.566	2.124	0	0.00419127	0.427	282	tags=69%,
GOBP_NUCLEOSIDE_PHOSPHATE_BIOSYNTHETIC_PROCESS	20	0.528	2.114	0	0.00445423	0.452	304	tags=75%,
GOBP_RIBOSE_PHOSPHATE_BIOSYNTHETIC_PROCESS	18	0.560	2.113	0	0.00448363	0.457	304	tags=72%,
GOMF_TRANSLATION_REGULATOR_ACTIVITY_NUCLEIC_ACID_BINDING	20	0.528	2.103	0.00211416	0.00496626	0.485	326	tags=70%,
REACTOME_CELL_CYCLE_CHECKPOINTS	25	0.488	2.102	0	0.00496869	0.49	332	tags=64%,
GOMF_TRANSLATION_FACTOR_ACTIVITY_RNA_BINDING	20	0.528	2.094	0.00231481	0.00535323	0.52	326	tags=70%,
GOBP_REGULATION_OF_ANIMAL_ORGAN_MORPHOGENESIS	21	0.513	2.092	0.00460829	0.00542685	0.527	332	tags=76%,
REACTOME_M_PHASE	29	0.467	2.082	0	0.00582332	0.554	341	tags=66%,
GOMF_TRANSLATION_REGULATOR_ACTIVITY	23	0.498	2.075	0.00225733	0.00620008	0.581	326	tags=65%,
REACTOME_SIGNALING_BY_ROBO_RECEPTORS	23	0.498	2.070	0.00227790	0.00641961	0.597	332	tags=65%,
GOBP_AEROBIC_RESPIRATION	20	0.524	2.069	0.00216919	0.00643301	0.598	400	tags=80%,
REACTOME_MAPK6_MAPK4_SIGNALING	20	0.518	2.067	0.00227272	0.00648878	0.603	332	tags=70%,
GOBP_FC_RECEPTOR_SIGNALING_PATHWAY	33	0.442	2.066	0.00229357	0.00646563	0.604	332	tags=58%,
GOBP_ACTIVATION_OF_INNATE_IMMUNE_RESPONSE	33	0.459	2.057	0	0.00701763	0.637	111	tags=36%,
REACTOME_ANTIGEN_PROCESSING_CROSS_PRESENTATION	31	0.462	2.053	0	0.00723027	0.65	332	tags=68%,
GOBP_REGULATION_OF_STEM_CELL_DIFFERENTIATION	19	0.535	2.040	0	0.00797630	0.684	332	tags=74%,
REACTOME_CELL_CYCLE_MITOTIC	41	0.412	2.035	0	0.00820921	0.692	342	tags=59%,
REACTOME_TCR_SIGNALING	25	0.479	2.033	0.00219780	0.00826535	0.697	332	tags=64%,
GOBP_GENERATION_OF_PRECURSOR_METABOLITES_AND_ENERGY	73	0.356	2.033	0	0.00825948	0.702	480	tags=74%,
REACTOME_TRANSCRIPTIONAL_REGULATION_BY_RUNX1	29	0.469	2.027	0.00253164	0.00859373	0.723	335	tags=62%,
GOBP_REGULATION_OF_CELLULAR_AMINO_ACID_METABOLIC_PROCESS	17	0.552	2.025	0	0.00867353	0.731	332	tags=82%,
GOBP_CELL_RECOGNITION	15	0.557	2.023	0	0.00881402	0.737	214	tags=53%,
GOBP_POSITIVE_REGULATION_OF_RESPONSE_TO_BIOTIC_STIMULUS	44	0.394	2.018	0	0.00908466	0.752	124	tags=32%,
GOBP_POST_TRANSLATIONAL_PROTEIN_MODIFICATION	36	0.413	2.015	0	0.00926836	0.762	337	tags=64%,
HP_ABNORMAL_PARANASAL_SINUS_MORPHOLOGY	23	0.486	2.011	0	0.00953314	0.774	218	tags=52%,

KEGG_ALZHEIMERS_DISEASE	39	0.420	2.002	0	0.01010288	0.791	378	tags=74%,
GOCC_AZUROPHIL_GRANULE_LUMEN	22	0.496	1.996	0.00221729	0.0105204	0.806	113	tags=32%,
REACTOME_MITOCHONDRIAL_BIOGENESIS	15	0.547	1.992	0.00444444	0.01071390	0.816	304	tags=73%,
KEGG_CARDIAC_MUSCLE_CONTRACTION	15	0.548	1.984	0.00209205	0.01145426	0.84	378	tags=80%,
REACTOME_SIGNALING_BY_WNT	36	0.417	1.979	0.00233100	0.01190011	0.854	339	tags=56%,
GOMF_CATION_CHANNEL_ACTIVITY	15	0.560	1.971	0	0.01271471	0.872	304	tags=73%,
HALLMARK_INTERFERON_ALPHA_RESPONSE	25	0.461	1.970	0.00227272	0.01280234	0.877	225	tags=56%,
GOCC_OXIDOREDUCTASE_COMPLEX	24	0.477	1.966	0.00446428	0.01313423	0.886	463	tags=88%,
REACTOME_TRANSCRIPTIONAL_REGULATION_BY_RUNX3	24	0.460	1.966	0.00248756	0.01306212	0.886	332	tags=58%,
GOBP_RESPONSE_TO_TUMOR_NECROSIS_FACTOR	36	0.410	1.960	0.00476190	0.01368335	0.898	111	tags=33%,
GOCC_PIGMENT_GRANULE	22	0.476	1.959	0.00223713	0.01368266	0.902	358	tags=68%,
REACTOME_CELL_CYCLE	52	0.375	1.948	0.00707547	0.01475880	0.916	342	tags=54%,
KEGG_SPLICEOSOME	17	0.514	1.947	0.00476190	0.01481221	0.918	473	tags=88%,
GOBP_BIOLOGICAL_PROCESS_INVOLVED_IN_SYMBIOTIC_INTERACTION	115	0.308	1.940	0	0.01559245	0.928	273	tags=40%,
REACTOME_FC_EPSILON_RECEPTOR_FCERI_SIGNALING	26	0.452	1.935	0.00691244	0.01608772	0.931	111	tags=35%,
GOBP_STEM_CELL_DIFFERENTIATION	26	0.448	1.935	0.00466200	0.01600801	0.931	332	tags=62%,
GOBP_REGULATION_OF_CELLULAR_AMINE_METABOLIC_PROCESS	19	0.492	1.920	0.00930232	0.01767913	0.949	332	tags=74%,
HP_ABNORMALITY_OF_THE_MITOCHONDRION	17	0.508	1.918	0.00828157	0.01776730	0.95	478	tags=88%,
GOBP_POSITIVE_REGULATION_OF_IMMUNE_RESPONSE	98	0.322	1.918	0	0.01769685	0.95	111	tags=23%,
GOCC_MEMBRANE_PROTEIN_COMPLEX	131	0.297	1.914	0	0.01821838	0.953	480	tags=63%,
REACTOME_ASPARAGINE_N_LINKED_GLYCOSYLATION	29	0.438	1.913	0.00913242	0.01816370	0.953	481	tags=79%,
REACTOME_TRANSPORT_TO_THE_GOLGI_AND_SUBSEQUENT_MODIFICATION	16	0.517	1.910	0.00443458	0.01857899	0.958	595	tags=100%
GOMF_PASSIVE_TRANSMEMBRANE_TRANSPORTER_ACTIVITY	19	0.493	1.909	0.00455580	0.01860965	0.958	253	tags=58%,
HP_ABNORMALITY_OF_ACID_BASE_HOMEOSTASIS	26	0.440	1.899	0.00456621	0.02003304	0.968	463	tags=81%,
GOBP_PROTEIN_MODIFICATION_BY_SMALL_PROTEIN_REMOVAL	34	0.404	1.898	0.00240963	0.02007783	0.968	332	tags=56%,
GOBP_POSTTRANSCRIPTIONAL_REGULATION_OF_GENE_EXPRESSION	72	0.335	1.896	0	0.02024547	0.97	303	tags=47%,
HP_ABNORMAL_CNS_MYELINATION	22	0.459	1.892	0.00909090	0.02070365	0.971	475	tags=82%,
HP_ABNORMAL_PHARYNX_MORPHOLOGY	24	0.444	1.885	0.00439560	0.02184770	0.978	98	tags=33%,
HP_ABNORMALITY_OF_THE_PHARYNX	28	0.423	1.884	0.00854700	0.02184371	0.979	98	tags=32%,
REACTOME_CELLULAR_RESPONSES_TO_EXTERNAL_STIMULI	80	0.323	1.880	0	0.02241493	0.981	342	tags=50%,
HP_COUGH	17	0.501	1.879	0.00214592	0.02238948	0.981	98	tags=35%,
REACTOME_NEDDYLATION	24	0.443	1.874	0.00439560	0.02308968	0.983	332	tags=63%,
GOBP_RIBONUCLEOPROTEIN_COMPLEX_SUBUNIT_ORGANIZATION	27	0.429	1.870	0.01565995	0.02374711	0.984	326	tags=59%,
GOBP_RIBONUCLEOPROTEIN_COMPLEX_BIOGENESIS	36	0.394	1.867	0.00227790	0.02406749	0.984	326	tags=56%,
HP_RECURRENT_UPPER_RESPIRATORY_TRACT_INFECTIONS	24	0.444	1.844	0.00245098	0.0282563	0.993	98	tags=33%,
GOBP_DEFENSE_RESPONSE_TO_BACTERIUM	35	0.390	1.842	0.00928074	0.02833642	0.994	102	tags=29%,
HP_ABNORMALITY_OF_THE_UPPER_RESPIRATORY_TRACT	46	0.359	1.839	0.00496277	0.02890999	0.996	218	tags=37%,
REACTOME_CLEC7A_DECTIN_1_SIGNALING	21	0.451	1.828	0.00865800	0.03116713	0.996	332	tags=71%,
HP_ABNORMAL_LYMPHOCYTE_PHYSIOLOGY	37	0.381	1.810	0.00461893	0.03510756	0.999	164	tags=32%,
GOBP_IMMUNE_RESPONSE_REGULATING_SIGNALING_PATHWAY	72	0.322	1.806	0	0.03570443	0.999	111	tags=25%,
HP_ABNORMAL_TRACHEOBRONCHIAL_MORPHOLOGY	30	0.401	1.806	0.01079913	0.03566353	0.999	246	tags=47%,
GOBP_MITOCHONDRION_ORGANIZATION	56	0.335	1.799	0.00222222	0.037152	0.999	475	tags=73%,
REACTOME_TP53_REGULATES_METABOLIC_GENES	24	0.419	1.791	0.01094091	0.03911794	0.999	405	tags=71%,
HP_LYMPHOPENIA	18	0.477	1.786	0.01492537	0.04047644	0.999	96	tags=39%,
GOBP_ADAPTIVE_IMMUNE_RESPONSE	61	0.325	1.779	0	0.04214635	0.999	98	tags=23%,
GOBP_REGULATION_OF_INNATE_IMMUNE_RESPONSE	55	0.330	1.778	0	0.04208335	0.999	111	tags=25%,
REACTOME_POST_TRANSLATIONAL_PROTEIN_MODIFICATION	106	0.283	1.775	0.00484261	0.04296997	0.999	352	tags=47%,
GOMF_CHAPERONE_BINDING	15	0.480	1.772	0.01991150	0.04351255	0.999	511	tags=87%,
GOBP_POSITIVE_REGULATION_OF_TYPE_I_INTERFERON_PRODUCTION	17	0.467	1.768	0.00938967	0.04464090	0.999	39	tags=18%,
HP_ABNORMAL_BRONCHUS_MORPHOLOGY	19	0.441	1.767	0.01666666	0.04495195	0.999	218	tags=47%,
GOBP_T_CELL_RECEPTOR_SIGNALING_PATHWAY	29	0.393	1.764	0.01830663	0.04551230	0.999	141	tags=38%,
GOBP_REGULATION_OF_MRNA_CATABOLIC_PROCESS	37	0.360	1.760	0.00917431	0.04637620	0.999	332	tags=54%,
GOMF_CATION_TRANSMEMBRANE_TRANSPORTER_ACTIVITY	32	0.384	1.749	0.01339285	0.04987563	1	340	tags=59%,
GOBP_REGULATION_OF_CELL_CYCLE_PHASE_TRANSITION	45	0.344	1.746	0.00488997	0.05079135	1	118	tags=31%,
REACTOME_ADAPTIVE_IMMUNE_SYSTEM	102	0.285	1.745	0	0.05084216	1	236	tags=35%,
REACTOME_DEUBIQUITINATION	30	0.397	1.738	0.01190476	0.0529016	1	332	tags=57%,
GOMF_GUANYL_NUCLEOTIDE_BINDING	28	0.396	1.736	0.01955990	0.05345102	1	127	tags=29%,
HP_ABNORMALITY_OF_THE_CEREBROSPINAL_FLUID	34	0.366	1.732	0.01647058	0.05484448	1	463	tags=71%,
REACTOME_APOPTOSIS	34	0.377	1.720	0.01213592	0.05882055	1	332	tags=53%,
GOBP_REGULATION_OF_TRANSCRIPTION_FROM_RNA_POLYMERASE_II_PROMOT	22	0.420	1.706	0.02517162	0.06415567	1	332	tags=64%,
GOCC_ENVELOPE	109	0.274	1.703	0.00519480	0.06510538	1	340	tags=47%,
GOBP_POSITIVE_REGULATION_OF_IMMUNE_SYSTEM_PROCESS	134	0.263	1.701	0.00269541	0.06550673	1	111	tags=20%,
HP_ABNORMALITY_OF_HUMORAL_IMMUNITY	36	0.359	1.693	0.01149425	0.06866595	1	164	tags=31%,
GOBP_TYPE_I_INTERFERON_PRODUCTION	20	0.424	1.688	0.02494331	0.07053802	1	77	tags=20%,
GOBP_LYMPHOCYTE_MEDIATED_IMMUNITY	33	0.363	1.683	0.02619047	0.07247922	1	53	tags=21%,
GOBP_RNA_SPLICING_VIA_TRANSESTERIFICATION_REACTIONS	38	0.351	1.682	0.02137767	0.07264088	1	486	tags=71%,
HP_NEOPLASM_OF_THE_SKIN	17	0.443	1.680	0.02267573	0.0734258	1	98	tags=29%,
HP_ABNORMAL_CELLULAR_PHENOTYPE	61	0.304	1.679	0.01295336	0.07320827	1	303	tags=41%,
REACTOME_ANTIGEN_PROCESSING_UBIQUITINATION_PROTEASOME_DEGRADAT	31	0.364	1.679	0.00997506	0.07299276	1	111	tags=29%,
GOBP_PROTEIN_POLYUBIQUITINATION	30	0.371	1.678	0.01978022	0.07297117	1	111	tags=33%,
HP_LEUKOPENIA	28	0.380	1.675	0.02914798	0.07441519	1	96	tags=29%,
HP_PNEUMONIA	24	0.388	1.673	0.03571428	0.07497594	1	218	tags=42%,
HP_IMMUNODEFICIENCY	31	0.374	1.669	0.02455357	0.07638556	1	218	tags=39%,
GOBP_MRNA_METABOLIC_PROCESS	82	0.281	1.659	0.00530503	0.08069118	1	486	tags=65%,
HP_RECURRENT_RESPIRATORY_INFECTIONS	50	0.310	1.646	0.02347418	0.08727603	1	98	tags=22%,
HP_RECURRENT_SKIN_INFECTIONS	19	0.419	1.644	0.03030303	0.08810972	1	148	tags=37%,
GOBP_REGULATION_OF_RESPONSE_TO_BIOTIC_STIMULUS	67	0.293	1.643	0.00779220	0.08839282	1	280	tags=42%,
GOBP_DEFENSE_RESPONSE_TO_OTHER_ORGANISM	150	0.244	1.635	0.00795756	0.09214599	1	164	tags=23%,
GOCC_ENDOPLASMIC_RETICULUM_GOLGI_INTERMEDIATE_COMPARTMENT	16	0.465	1.633	0.02068965	0.09300075	1	412	tags=75%,
GOCC_MITOCHONDRION	122	0.253	1.631	0.00485436	0.09370649	1	311	tags=40%,
GOBP_INNATE_IMMUNE_RESPONSE	132	0.252	1.630	0.00544959	0.09418892	1	113	tags=20%,

HALLMARK_E2F_TARGETS	16	0.444	1.627	0.04273504	0.09516835	1	312	tags=56%,
HP_RED_EYE	15	0.455	1.623	0.03944315	0.0975209	1	98	tags=40%,
HP_RECURRENT_BACTERIAL_INFECTIONS	25	0.378	1.621	0.02262443	0.09808154	1	98	tags=28%,
GOBP_MEMBRANE_ORGANIZATION	99	0.262	1.621	0.01052631	0.09782959	1	253	tags=32%,
GOBP_PURINE_CONTAINING_COMPOUND_METABOLIC_PROCESS	33	0.342	1.620	0.03004291	0.09782625	1	147	tags=30%,
GOBP_INTERFERON_GAMMA_MEDIATED_SIGNALING_PATHWAY	18	0.421	1.614	0.03189066	0.1009289	1	39	tags=28%,
GOMF_HISTONE_BINDING	19	0.418	1.614	0.04608295	0.10052448	1	78	tags=26%,
GOBP_RNA_CATABOLIC_PROCESS	48	0.305	1.611	0.02637889	0.10174995	1	139	tags=27%,
GOBP_ADAPTIVE_IMMUNE_RESPONSE_BASED_ON_SOMATIC_RECOMBINATION_C	38	0.335	1.611	0.01882352	0.10166065	1	53	tags=18%,
GOMF_ENDOPEPTIDASE_ACTIVITY	32	0.340	1.591	0.03712297	0.11347059	1	332	tags=59%,
HALLMARK_ALLOGRAFT_REJECTION	47	0.313	1.591	0.02624671	0.11303117	1	219	tags=38%,
REACTOME_CLASS_I_MHC_MEDIATED_ANTIGEN_PROCESSING_PRESENTATION	48	0.307	1.591	0.03731343	0.11259103	1	236	tags=38%,
GOBP_RESPONSE_TO_INTERLEUKIN_1	35	0.337	1.590	0.03472222	0.11286067	1	111	tags=31%,
HP_RESPIRATORY_TRACT_INFECTION	58	0.294	1.590	0.02153110	0.11243269	1	218	tags=31%,
HP_ABNORMAL_NEUTROPHIL_COUNT	25	0.367	1.585	0.05630630	0.11532453	1	98	tags=28%,
GOBP_PEPTIDE_BIOSYNTHETIC_PROCESS	59	0.297	1.582	0.00982801	0.11688629	1	306	tags=46%,
GOBP_RNA_LOCALIZATION	22	0.399	1.581	0.03311258	0.11686785	1	415	tags=68%,
GOBP_POSITIVE_REGULATION_OF_CELLULAR_AMIDE_METABOLIC_PROCESS	18	0.411	1.577	0.04139433	0.11895207	1	303	tags=56%,
GOBP_CELLULAR_PROTEIN_CONTAINING_COMPLEX_ASSEMBLY	103	0.257	1.575	0.01278772	0.12022757	1	357	tags=46%,
GOBP_CELL_CYCLE_PHASE_TRANSITION	54	0.297	1.572	0.02870813	0.12142222	1	118	tags=26%,
REACTOME_PTEN_REGULATION	24	0.383	1.563	0.04978355	0.12691462	1	342	tags=63%,
GOBP_RESPONSE_TO_VIRUS	40	0.315	1.560	0.03837472	0.12878095	1	189	tags=35%,
GOBP_RNA_SPLICING	45	0.303	1.557	0.03631961	0.13046594	1	486	tags=64%,
GOBP_INNATE_IMMUNE_RESPONSE_ACTIVATING_SIGNAL_TRANSDUCTION	25	0.370	1.555	0.02466367	0.13112943	1	111	tags=32%,
HP_ABNORMAL_THROMBOCYTE_MORPHOLOGY	49	0.301	1.555	0.03349282	0.13097036	1	164	tags=29%,
GOBP_REGULATION_OF_MRNA_METABOLIC_PROCESS	54	0.285	1.553	0.04295942	0.13185407	1	332	tags=44%,
HP_ECTOPIC_CALCIFICATION	17	0.412	1.547	0.05708245	0.13599683	1	246	tags=41%,
GOBP_ANATOMICAL_STRUCTURE_HOMEOSTASIS	34	0.323	1.545	0.02898550	0.1369015	1	214	tags=38%,
GOBP_MRNA_PROCESSING	46	0.296	1.545	0.03271028	0.13658349	1	486	tags=65%,
GOBP_PROTEIN_LOCALIZATION_TO_MITOCHONDRION	15	0.422	1.541	0.05714285	0.13900177	1	475	tags=80%,
GOBP_NEGATIVE_REGULATION_OF_WNT_SIGNALING_PATHWAY	28	0.343	1.539	0.06132075	0.14000827	1	111	tags=32%,
GOMF_SINGLE_STRANDED_DNA_BINDING	16	0.416	1.538	0.06008583	0.13966535	1	178	tags=38%,
KEGG_LYSOSOME	20	0.388	1.537	0.06415929	0.13983937	1	419	tags=70%,
GOBP_ORGANONITROGEN_COMPOUND_BIOSYNTHETIC_PROCESS	130	0.237	1.537	0.00514138	0.13980588	1	306	tags=42%,
GOBP_NEGATIVE_REGULATION_OF_CELL_CYCLE_PROCESS	44	0.301	1.531	0.02988505	0.14366235	1	342	tags=50%,
GOBP_HUMORAL_IMMUNE_RESPONSE	32	0.335	1.526	0.04600484	0.14674799	1	164	tags=38%,
GOBP_RESPONSE_TO_TOPOLOGICALLY_INCORRECT_PROTEIN	18	0.390	1.526	0.05011933	0.14624071	1	579	tags=83%,
GOMF_CADHERIN_BINDING	51	0.291	1.526	0.03588516	0.14583853	1	322	tags=43%,
HP_OTITIS_MEDIA	30	0.326	1.525	0.06024096	0.14590506	1	98	tags=23%,
GOCC_CATALYTIC_COMPLEX	139	0.233	1.525	0.01612903	0.14586681	1	502	tags=63%,
REACTOME_SIGNALING_BY_NOTCH	34	0.321	1.521	0.03874092	0.14869519	1	111	tags=26%,
HP_ABNORMAL_INFLAMMATORY_RESPONSE	94	0.250	1.517	0.04381443	0.15084082	1	110	tags=20%,
GOBP_RESPONSE_TO_BIOTIC_STIMULUS	191	0.221	1.516	0.01146131	0.15122357	1	164	tags=23%,
REACTOME_INFECTIOUS_DISEASE	96	0.251	1.510	0.02020202	0.15591976	1	332	tags=42%,
GOBP_CELLULAR_PROTEIN_CATABOLIC_PROCESS	79	0.260	1.510	0.02522935	0.155604	1	320	tags=42%,
GOBP_INTERLEUKIN_1_MEDIATED_SIGNALING_PATHWAY	24	0.355	1.507	0.07855626	0.15717615	1	111	tags=38%,
HP_INCREASED_SERUM_LACTATE	15	0.418	1.506	0.06741573	0.15707754	1	300	tags=60%,
GOBP_NEGATIVE_REGULATION_OF_CELL_CYCLE_PHASE_TRANSITION	33	0.326	1.505	0.06067961	0.15725614	1	332	tags=55%,
HP_HIGH_FOREHEAD	17	0.400	1.505	0.06526806	0.15689728	1	184	tags=29%,
GOCC_ENDOCYTIC_VESICLE_MEMBRANE	22	0.368	1.500	0.06651376	0.16090044	1	221	tags=45%,
HP_UNUSUAL_INFECTION	81	0.259	1.499	0.02179176	0.16119172	1	98	tags=19%,
REACTOME_INTERLEUKIN_1_SIGNALING	23	0.359	1.497	0.06823529	0.16298069	1	111	tags=35%,
GOBP_REGULATION_OF_DNA_TEMPLATED_TRANSCRIPTION_IN_RESPONSE_TO_S	26	0.349	1.495	0.06308411	0.16401881	1	111	tags=31%,
GOCC_TRANS_GOLGI_NETWORK	19	0.383	1.494	0.07439825	0.16393106	1	268	tags=47%,
REACTOME_C_TYPE_LECTIN_RECEPTORS_CLRS	28	0.337	1.481	0.06206896	0.17504935	1	111	tags=32%,
GOCC_INTRINSIC_COMPONENT_OF_ENDOPLASMIC_RETICULUM_MEMBRANE	17	0.396	1.481	0.07159353	0.1746762	1	238	tags=41%,
GOBP_POSITIVE_REGULATION_OF_I_KAPPAB_KINASE_NF_KAPPAB_SIGNALING	15	0.412	1.481	0.07865169	0.1741127	1	189	tags=40%,
HP_ABNORMAL_LEUKOCYTE_COUNT	49	0.286	1.476	0.05238095	0.17817903	1	98	tags=22%,
HP_SKIN_RASH	20	0.367	1.475	0.08371041	0.17804208	1	153	tags=40%,
GOBP_AMINE_METABOLIC_PROCESS	23	0.353	1.473	0.05643341	0.17951024	1	351	tags=65%,
GOMF_PROTEIN_HOMODIMERIZATION_ACTIVITY	50	0.279	1.473	0.05422993	0.17926778	1	88	tags=20%,
GOCC_NUCLEOLUS	78	0.257	1.472	0.03807106	0.17926006	1	317	tags=37%,
REACTOME_METABOLISM_OF_AMINO_ACIDS_AND_DERIVATIVES	26	0.335	1.470	0.09610984	0.18102254	1	332	tags=58%,
GOBP_CELLULAR_RESPONSE_TO_DNA_DAMAGE_STIMULUS	60	0.268	1.465	0.04901961	0.18471202	1	107	tags=15%,
HP_ABNORMALITY_OF_BRAINSTEM_MORPHOLOGY	15	0.403	1.465	0.07918552	0.18464631	1	463	tags=80%,
GOBP_REGULATION_OF_IMMUNE_RESPONSE	136	0.224	1.463	0.02419354	0.18595655	1	111	tags=18%,
REACTOME_INTERLEUKIN_12_FAMILY_SIGNALING	17	0.394	1.456	0.07882883	0.19174999	1	211	tags=41%,
GOBP_POSITIVE_REGULATION_OF_CELL_ACTIVATION	52	0.272	1.455	0.05700712	0.19222179	1	84	tags=19%,
HP_FAILURE_TO_THRIVE	78	0.249	1.452	0.02590673	0.19434175	1	98	tags=18%,
GOBP_NUCLEOBASE_CONTAINING_SMALL_MOLECULE_METABOLIC_PROCESS	40	0.297	1.451	0.06997742	0.19470805	1	304	tags=48%,
GOBP_TRANSMEMBRANE_TRANSPORT	119	0.229	1.449	0.03005464	0.19608873	1	296	tags=39%,
GOBP_PROTEASOMAL_PROTEIN_CATABOLIC_PROCESS	47	0.284	1.448	0.07159904	0.1963799	1	332	tags=47%,
HP_ABNORMAL_LYMPHOCYTE_MORPHOLOGY	26	0.339	1.447	0.09367681	0.1969193	1	96	tags=27%,
GOCC_PERINUCLEAR_REGION_OF_CYTOPLASM	61	0.269	1.445	0.05287356	0.1985766	1	281	tags=36%,
GOBP_POSITIVE_REGULATION_OF_DEFENSE_RESPONSE	61	0.265	1.444	0.06636155	0.19922277	1	111	tags=23%,
HP_INFLAMMATORY_ABNORMALITY_OF_THE_EYE	25	0.347	1.442	0.09324009	0.19983086	1	156	tags=36%,
GOBP_INTRINSIC_APOPTOTIC_SIGNALING_PATHWAY	39	0.300	1.441	0.07572383	0.20095558	1	107	tags=21%,
HP_ABNORMAL_CONJUNCTIVA_MORPHOLOGY	22	0.355	1.440	0.09932279	0.20082082	1	98	tags=27%,
HP_MALABSORPTION	21	0.356	1.440	0.08313539	0.20052208	1	164	tags=33%,
GOMF_ION_TRANSMEMBRANE_TRANSPORTER_ACTIVITY	56	0.271	1.437	0.077951	0.20322101	1	304	tags=45%,
GOBP_CELLULAR_RESPONSE_TO_TOPOLOGICALLY_INCORRECT_PROTEIN	17	0.381	1.435	0.0989011	0.20441924	1	579	tags=82%,

HP_POOR_HEAD_CONTROL	17	0.376	1.434	0.10043668	0.20457953	1	549	tags=88%,
GOBP_PROTEIN_CONTAINING_COMPLEX_SUBUNIT_ORGANIZATION	164	0.213	1.433	0.02638522	0.20501134	1	331	tags=38%,
HP_RECURRENT_OTITIS_MEDIA	15	0.391	1.432	0.07061503	0.20497261	1	98	tags=27%,
GOBP_REGULATION_OF_PROTEIN_STABILITY	36	0.306	1.432	0.07568807	0.2046988	1	271	tags=42%,
GOBP_PEPTIDE_METABOLIC_PROCESS	72	0.252	1.431	0.04466501	0.20480804	1	387	tags=54%,
GOBP_RESPONSE_TO_INTERFERON_GAMMA	29	0.325	1.428	0.08571429	0.20712101	1	39	tags=21%,
HP_ABNORMAL_IMMUNE_SYSTEM_MORPHOLOGY	73	0.246	1.418	0.06281407	0.21706894	1	98	tags=19%,
GOBP_PROTEIN_STABILIZATION	23	0.345	1.418	0.09955752	0.21690899	1	271	tags=43%,
GOBP_INTRACELLULAR_TRANSPORT	145	0.214	1.417	0.02203856	0.21739963	1	417	tags=48%,
GOBP_ESTABLISHMENT_OF_PROTEIN_LOCALIZATION_TO_ORGANELLE	43	0.283	1.413	0.08411215	0.2213474	1	475	tags=67%,
GOCC_MITOCHONDRIAL_MATRIX	25	0.323	1.409	0.09198113	0.22448906	1	253	tags=40%,
GOBP_ESTABLISHMENT_OF_RNA_LOCALIZATION	18	0.365	1.406	0.10849056	0.22729774	1	415	tags=67%,
GOCC_AZUROPHIL_GRANULE	36	0.297	1.400	0.11604938	0.233069	1	113	tags=19%,
HP_ABNORMAL_GRANULOCYTE_COUNT	30	0.303	1.395	0.10690423	0.23834343	1	98	tags=23%,
GOBP_AMIDE_BIOSYNTHETIC_PROCESS	70	0.242	1.394	0.05249343	0.23890549	1	387	tags=50%,
GOCC_EXTERNAL_SIDE_OF_PLASMA_MEMBRANE	41	0.282	1.393	0.08333333	0.23937099	1	163	tags=27%,
GOBP_NEGATIVE_REGULATION_OF_CANONICAL_WNT_SIGNALING_PATHWAY	25	0.332	1.383	0.12356979	0.25039828	1	339	tags=60%,
GOMF_PEPTIDASE_ACTIVITY	41	0.279	1.382	0.08877285	0.2504069	1	135	tags=29%,
GOMF_IDENTICAL_PROTEIN_BINDING	160	0.206	1.382	0.02732240	0.25013834	1	143	tags=19%,
GOMF_OXIDOREDUCTASE_ACTIVITY_ACTING_ON_NAD_P_H	19	0.353	1.379	0.13390929	0.25243694	1	463	tags=79%,
GOBP_POSITIVE_REGULATION_OF_RESPONSE_TO_EXTERNAL_STIMULUS	78	0.234	1.378	0.05660377	0.25271448	1	163	tags=26%,
GOBP_ORGANIC_CYCLIC_COMPOUND_CATABOLIC_PROCESS	62	0.249	1.377	0.06542056	0.2531668	1	159	tags=26%,
GOBP_POSITIVE_REGULATION_OF_PEPTIDASE_ACTIVITY	29	0.315	1.376	0.12641084	0.25338194	1	114	tags=24%,
HP_DISPLACEMENT_OF_THE_URETHRAL_MEATUS	23	0.326	1.370	0.10786517	0.2606564	1	232	tags=30%,
GOBP_REGULATION_OF_BIOLOGICAL_PROCESS_INVOLVED_IN_SYMBIOTIC_INTEF	23	0.332	1.369	0.12171838	0.26111192	1	70	tags=26%,
GOMF_PROTEIN_DIMERIZATION_ACTIVITY	67	0.242	1.367	0.08018868	0.26239076	1	196	tags=27%,
GOBP_REGULATION_OF_RESPONSE_TO_CYTOKINE_STIMULUS	26	0.311	1.367	0.12679426	0.26213834	1	77	tags=23%,
HP_ABNORMAL_LIVER_MORPHOLOGY	79	0.234	1.366	0.09456265	0.2623777	1	164	tags=23%,
GOBP_MORPHOGENESIS_OF_AN_EPITHELIUM	45	0.267	1.366	0.07816092	0.26199132	1	280	tags=40%,
HP_ABNORMALITY_OF_THE_CEREBRAL_VENTRICLES	49	0.262	1.361	0.10579345	0.26767406	1	509	tags=63%,
REACTOME_PROGRAMMED_CELL_DEATH	39	0.282	1.358	0.1097852	0.27063784	1	118	tags=26%,
GOBP_REGULATION_OF_VIRAL_LIFE_CYCLE	17	0.361	1.357	0.14442013	0.27062577	1	70	tags=29%,
GOCC_CLATHRIN_COATED_VESICLE_MEMBRANE	15	0.380	1.352	0.14495799	0.27672628	1	278	tags=53%,
GOBP_CELLULAR_PROTEIN_COMPLEX_DISASSEMBLY	16	0.371	1.349	0.1255814	0.27911893	1	324	tags=50%,
HP_ABSENT_SPEECH	19	0.343	1.349	0.14977974	0.27876645	1	805	tags=100%
HP_DIARRHEA	33	0.288	1.346	0.11650485	0.28265545	1	98	tags=18%,
GOBP_CELLULAR_AMIDE_METABOLIC_PROCESS	89	0.226	1.344	0.07360406	0.28386325	1	387	tags=51%,
GOBP_REGULATION_OF_LYMPHOCYTE_ACTIVATION	61	0.243	1.343	0.09624413	0.28398764	1	84	tags=16%,
GOBP_RESPONSE_TO_INTERLEUKIN_12	15	0.369	1.343	0.14193548	0.28357655	1	323	tags=53%,
GOCC_ENDOPLASMIC_RETICULUM_LUMEN	23	0.330	1.341	0.13126491	0.2852676	1	325	tags=61%,
HP_ENCEPHALOPATHY	16	0.363	1.339	0.15124154	0.2875698	1	508	tags=75%,
GOBP_NIK_NF_KAPPAB_SIGNALING	28	0.307	1.338	0.12362031	0.28797927	1	280	tags=50%,
GOBP_REGULATION_OF_WNT_SIGNALING_PATHWAY	36	0.284	1.334	0.11510792	0.29216403	1	111	tags=25%,
REACTOME_RNA_POLYMERASE_II_TRANSCRIPTION	108	0.216	1.333	0.1	0.292259	1	342	tags=41%,
HP_ABNORMALITY_OF_NEUTROPHILS	33	0.285	1.332	0.13856813	0.29389665	1	156	tags=27%,
GOMF_PROTEIN_C_TERMINUS_BINDING	15	0.368	1.326	0.16775599	0.30125722	1	317	tags=60%,
GOBP_CELLULAR_RESPONSE_TO_OXYGEN_LEVELS	36	0.283	1.326	0.12444445	0.300578	1	332	tags=53%,
HALLMARK_MTORC1_SIGNALING	33	0.284	1.326	0.12080537	0.29978618	1	240	tags=39%,
PID_MYC_ACTIV_PATHWAY	16	0.362	1.325	0.14482759	0.2995169	1	177	tags=38%,
REACTOME_CELL_SURFACE_INTERACTIONS_AT_THE_VASCULAR_WALL	25	0.311	1.324	0.13258427	0.29985467	1	58	tags=20%,
HP_ABNORMALITY_OF_FACIAL_SKELETON	82	0.225	1.321	0.10440835	0.303251	1	246	tags=28%,
GOBP_REGULATION_OF_CELLULAR_CATABOLIC_PROCESS	96	0.220	1.313	0.10473815	0.31341583	1	151	tags=22%,
GOCC_VACUOLE	103	0.214	1.312	0.10459184	0.31376955	1	271	tags=31%,
HP_ABNORMALITY_OF_THE_LIVER	91	0.216	1.311	0.09162304	0.3154838	1	164	tags=21%,
REACTOME_NERVOUS_SYSTEM_DEVELOPMENT	52	0.252	1.309	0.10952381	0.31717327	1	332	tags=44%,
GOBP_SIGNAL_TRANSDUCTION_BY_P53_CLASS_MEDIATOR	23	0.313	1.308	0.13478261	0.31800023	1	91	tags=17%,
GOBP_POSITIVE_REGULATION_OF_CYSTEINE_TYPE_ENDOPEPTIDASE_ACTIVITY	20	0.324	1.299	0.15513127	0.3299303	1	114	tags=25%,
HP_DECREASED_CIRCULATING_ANTIBODY_LEVEL	22	0.311	1.298	0.15952381	0.32952932	1	98	tags=23%,
GOBP_NEGATIVE_REGULATION_OF_MITOTIC_CELL_CYCLE	38	0.265	1.298	0.16461916	0.3299946	1	332	tags=47%,
HALLMARK_PROTEIN_SECRETION	17	0.341	1.297	0.18791947	0.330227	1	480	tags=76%,
HP_ABNORMAL_ERYTHROCYTE_MORPHOLOGY	62	0.238	1.295	0.13580246	0.33283865	1	97	tags=18%,
GOMF_PEPTIDE_BINDING	26	0.301	1.290	0.1557562	0.33834258	1	241	tags=42%,
GOBP_REGULATION_OF_MITOTIC_CELL_CYCLE	53	0.246	1.283	0.13921113	0.34797832	1	118	tags=25%,
GOBP_CELLULAR_AMINO_ACID_METABOLIC_PROCESS	25	0.307	1.283	0.189011	0.3470854	1	332	tags=60%,
GOBP_TISSUE_HOMEOSTASIS	21	0.320	1.279	0.18340611	0.3528337	1	211	tags=38%,
GOBP_LEUKOCYTE_PROLIFERATION	40	0.263	1.278	0.12781955	0.35277998	1	200	tags=30%,
HP_ABNORMAL_RESPIRATORY_SYSTEM_MORPHOLOGY	102	0.205	1.274	0.12345679	0.35752273	1	104	tags=16%,
GOCC_LATE_ENDOSOME	33	0.275	1.274	0.16385542	0.3568241	1	192	tags=27%,
GOBP_REGULATION_OF_DNA_BINDING	16	0.345	1.274	0.20229885	0.35649154	1	17	tags=13%,
GOMF_ION_CHANNEL_BINDING	15	0.343	1.272	0.19654427	0.35917312	1	118	tags=27%,
HP_SKIN_ULCER	17	0.338	1.267	0.1938326	0.3646509	1	212	tags=41%,
GOBP_LYMPHOCYTE_ACTIVATION	98	0.206	1.266	0.11627907	0.36509022	1	111	tags=16%,
REACTOME_SARS_COV_INFECTIONS	27	0.291	1.265	0.16210045	0.3663046	1	441	tags=59%,
GOBP_BIOLOGICAL_PROCESS_INVOLVED_IN_INTERACTION_WITH_HOST	18	0.325	1.264	0.19205298	0.36722782	1	167	tags=39%,
HP_ABNORMALITY_OF_THE_URETHRA	25	0.294	1.263	0.20581655	0.3673138	1	232	tags=28%,
GOBP_CELLULAR_RESPONSE_TO_RADIATION	16	0.343	1.262	0.20568928	0.36802545	1	26	tags=19%,
GOBP_DEFENSE_RESPONSE_TO_VIRUS	27	0.297	1.259	0.19025522	0.37265795	1	189	tags=33%,
GOCC_VESICLE_LUMEN	67	0.227	1.257	0.15223098	0.37481824	1	113	tags=19%,
GOBP_REGULATION_OF_CELL_CYCLE_PROCESS	66	0.222	1.254	0.1459854	0.378596	1	118	tags=21%,
GOBP_ANIMAL_ORGAN_MORPHOGENESIS	71	0.219	1.252	0.13978495	0.38012806	1	280	tags=34%,
HP_ABNORMAL_LUNG_MORPHOLOGY	90	0.206	1.250	0.13695091	0.38362446	1	104	tags=16%,

GOMF_GTPASE_ACTIVITY	24	0.297	1.248	0.18322296	0.38496903	1	113	tags=21%,
GOBP_REGULATION_OF_AUTOPHAGY	20	0.305	1.248	0.2	0.3844244	1	129	tags=20%,
PID_MTOR_4PATHWAY	15	0.358	1.247	0.19501133	0.38446146	1	262	tags=47%,
GOBP_CELLULAR_MACROMOLECULE_CATABOLIC_PROCESS	115	0.200	1.245	0.13559322	0.3880772	1	320	tags=36%,
GOBP_VIRAL_LIFE_CYCLE	37	0.262	1.239	0.20366132	0.39695084	1	189	tags=32%,
GOBP_GOLGI_VESICLE_TRANSPORT	28	0.283	1.237	0.21412803	0.39862856	1	527	tags=79%,
HP_ABNORMALITY_OF_THE_VASCULATURE_OF_THE_EYE	30	0.271	1.235	0.19863014	0.400484	1	98	tags=20%,
GOBP_VIRAL_GENOME_REPLICATION	16	0.345	1.234	0.20227273	0.4012992	1	189	tags=44%,
REACTOME_TRANSPORT_OF_SMALL_MOLECULES	65	0.221	1.234	0.17283951	0.4019084	1	286	tags=35%,
GOCC_VACUOLAR_MEMBRANE	51	0.237	1.233	0.19483568	0.40235656	1	271	tags=31%,
REACTOME_VESICLE_MEDIATED_TRANSPORT	65	0.217	1.233	0.15441176	0.4016236	1	440	tags=52%,
GOBP_NUCLEOBASE_CONTAINING_COMPOUND_TRANSPORT	23	0.301	1.229	0.22737306	0.40582275	1	415	tags=61%,
GOBP_PROTEIN_TARGETING	33	0.269	1.225	0.19761905	0.4127124	1	475	tags=67%,
GOMF_PROTEIN_CONTAINING_COMPLEX_BINDING	124	0.191	1.223	0.14673913	0.415665	1	328	tags=39%,
HP_HYPERTELORISM	44	0.244	1.220	0.20413436	0.41996267	1	118	tags=16%,
GOMF_AMIDE_BINDING	32	0.261	1.220	0.21140142	0.4191061	1	58	tags=19%,
REACTOME_INTERLEUKIN_1_FAMILY_SIGNALING	29	0.272	1.219	0.20581655	0.41888285	1	111	tags=28%,
REACTOME_INTERFERON_SIGNALING	24	0.290	1.213	0.24178404	0.42781526	1	47	tags=21%,
GOCC_GOLGI_APPARATUS_SUBCOMPARTMENT	63	0.217	1.206	0.1875	0.44035697	1	31	tags=10%,
GOBP_REGULATION_OF_CATABOLIC_PROCESS	108	0.195	1.205	0.16537468	0.44101417	1	139	tags=19%,
REACTOME_NEURONAL_SYSTEM	16	0.325	1.204	0.25	0.44116867	1	573	tags=81%,
GOBP_CARBOHYDRATE_DERIVATIVE_BIOSYNTHETIC_PROCESS	47	0.236	1.203	0.20990565	0.44132933	1	313	tags=45%,
GOMF_HYDROLASE_ACTIVITY_ACTING_ON_ACID_ANHYDRIDES	58	0.228	1.202	0.22413793	0.44203183	1	265	tags=31%,
GOBP_TISSUE_MORPHOGENESIS	53	0.230	1.199	0.21463415	0.44717357	1	280	tags=36%,
HP_ABNORMALITY_OF_DENTAL_STRUCTURE	19	0.304	1.197	0.2413793	0.44895718	1	288	tags=42%,
HP_DECREASED_BODY_WEIGHT	113	0.191	1.194	0.1922078	0.4533442	1	98	tags=14%,
GOBP_CANONICAL_WNT_SIGNALING_PATHWAY	34	0.254	1.194	0.2189781	0.45247874	1	111	tags=24%,
HP_ABNORMAL_MYELINATION	40	0.241	1.193	0.21226415	0.45415804	1	475	tags=60%,
HP_AUTOIMMUNITY	21	0.297	1.191	0.24390244	0.45555568	1	62	tags=19%,
HP_ABNORMAL_MYELOID_LEUKOCYTE_MORPHOLOGY	40	0.250	1.189	0.23349057	0.45929438	1	156	tags=25%,
GOCC_SIDE_OF_MEMBRANE	66	0.213	1.188	0.21428572	0.45956087	1	163	tags=21%,
GOBP_CATION_TRANSMEMBRANE_TRANSPORT	54	0.226	1.186	0.2452381	0.4623233	1	304	tags=41%,
HP_ABNORMALITY_OF_SKIN_PHYSIOLOGY	52	0.229	1.184	0.21582733	0.4644064	1	102	tags=21%,
HP_MIDFACE_RETRUSION	22	0.292	1.183	0.27433628	0.46633396	1	794	tags=95%,
GOCC_COATED_VESICLE_MEMBRANE	20	0.303	1.182	0.26773456	0.46712142	1	227	tags=35%,
GOCC_FICOLIN_1_RICH_GRANULE_LUMEN	33	0.251	1.179	0.2448037	0.47093663	1	278	tags=42%,
HP_GONOSOMAL_INHERITANCE	17	0.317	1.179	0.2786177	0.47047374	1	237	tags=41%,
GOMF_OXIDOREDUCTASE_ACTIVITY	66	0.210	1.176	0.24630542	0.47412747	1	378	tags=50%,
HP_ABNORMALITY_OF_THE_MIDFACE	42	0.233	1.175	0.22222222	0.4757418	1	794	tags=90%,
HP_ABNORMALITY_OF_THE_PERIORBITAL_REGION	18	0.308	1.174	0.27586207	0.47570315	1	153	tags=28%,
GOBP_NEGATIVE_REGULATION_OF_BINDING	21	0.293	1.174	0.26593408	0.4748537	1	51	tags=14%,
GOBP_INORGANIC_ION_TRANSMEMBRANE_TRANSPORT	49	0.232	1.173	0.25183374	0.47554165	1	340	tags=43%,
HP_ELEVATED_HEPATIC_TRANSAMINASE	20	0.293	1.170	0.2564706	0.48049372	1	96	tags=25%,
GOBP_NEGATIVE_REGULATION_OF_IMMUNE_SYSTEM_PROCESS	50	0.227	1.169	0.23600973	0.47983056	1	225	tags=30%,
HP_HYDROCEPHALUS	21	0.291	1.166	0.25770926	0.48535806	1	425	tags=57%,
HP_ANORECTAL_ANOMALY	22	0.282	1.165	0.28738317	0.48681518	1	246	tags=36%,
GOMF_MRNA_BINDING	35	0.246	1.162	0.2574032	0.49024555	1	487	tags=69%,
GOBP_MODIFICATION_DEPENDENT_MACROMOLECULE_CATABOLIC_PROCESS	62	0.212	1.160	0.23960881	0.49346632	1	332	tags=39%,
HP_EPICANTHUS	35	0.244	1.155	0.27559054	0.5019335	1	118	tags=17%,
REACTOME_INTRACELLULAR_SIGNALING_BY_SECOND_MESSENGERS	37	0.239	1.153	0.27944574	0.5038585	1	111	tags=22%,
HP_ABNORMAL_INTESTINE_MORPHOLOGY	56	0.216	1.152	0.24155845	0.50548613	1	206	tags=23%,
GOMF_SIGNALING_RECEPTOR_BINDING	135	0.176	1.149	0.19512194	0.5099914	1	167	tags=20%,
GOBP_RESPONSE_TO_OXYGEN_LEVELS	44	0.227	1.149	0.27083334	0.5093825	1	332	tags=45%,
HP_VENTRICULOMEGALY	30	0.253	1.149	0.2800926	0.50832516	1	509	tags=67%,
GOBP_REGULATION_OF_CYSTEINE_TYPE_ENDOPEPTIDASE_ACTIVITY	30	0.255	1.146	0.2832244	0.5122735	1	118	tags=23%,
GOMF_ATPASE_ACTIVITY	32	0.254	1.144	0.28947368	0.5160007	1	365	tags=44%,
GOBP_PROTEIN_CATABOLIC_PROCESS	94	0.189	1.138	0.25735295	0.5248839	1	320	tags=37%,
HP_APLASIA_HYPOPLASIA_OF_THE_SKIN	17	0.296	1.131	0.31637168	0.5381901	1	206	tags=29%,
HP_ABNORMALITY_OF_THE_OUTER_EAR	66	0.198	1.131	0.2584541	0.5374241	1	259	tags=27%,
GOBP_PROTEIN_LOCALIZATION_TO_ORGANELLE	72	0.201	1.130	0.26096997	0.53877485	1	481	tags=60%,
GOBP_DNA_METABOLIC_PROCESS	65	0.203	1.129	0.2735632	0.5390896	1	312	tags=37%,
HP_ABNORMALITY_OF_THE_ABDOMINAL_ORGANS	105	0.183	1.128	0.26717559	0.53962034	1	164	tags=19%,
HP_ABNORMALITY_OF_THE_SPLEEN	65	0.202	1.122	0.2884161	0.5515602	1	102	tags=17%,
GOMF_HYDROLASE_ACTIVITY_ACTING_ON_GLYCOSYL_BONDS	16	0.297	1.119	0.32045454	0.55579305	1	397	tags=56%,
HP_ABNORMAL_ENZYME_COENZYME_ACTIVITY	36	0.237	1.113	0.33966747	0.5657916	1	163	tags=25%,
GOMF_TRANSPORTER_ACTIVITY	68	0.201	1.113	0.30697674	0.56521755	1	304	tags=38%,
GOBP_REGULATION_OF_MRNA_SPLICING_VIA_SPLICEOSOME	15	0.311	1.113	0.3197279	0.5644331	1	464	tags=67%,
GOBP_PROTEIN_CONTAINING_COMPLEX_DISASSEMBLY	26	0.255	1.110	0.32372504	0.5691149	1	190	tags=27%,
GOBP_INTRACELLULAR_PROTEIN_TRANSPORT	93	0.184	1.110	0.28282827	0.5688355	1	481	tags=57%,
GOBP_ESTABLISHMENT_OF_PROTEIN_LOCALIZATION_TO_MEMBRANE	27	0.252	1.108	0.32175925	0.5703043	1	470	tags=59%,
REACTOME_MAPK_FAMILY_SIGNALING_CASCADES	43	0.225	1.108	0.30963302	0.5701008	1	111	tags=21%,
GOMF_UBIQUITIN_LIKE_PROTEIN_LIGASE_BINDING	30	0.252	1.106	0.33014354	0.5723482	1	163	tags=27%,
HP_CONGESTIVE_HEART_FAILURE	28	0.251	1.105	0.3135392	0.57250756	1	113	tags=18%,
GOBP_CELL_AGING	17	0.294	1.104	0.3311111	0.5752316	1	251	tags=41%,
GOBP_CELL_CELL_SIGNALING_BY_WNT	49	0.209	1.103	0.33004925	0.57513976	1	236	tags=29%,
HP_DYSPHAGIA	31	0.233	1.100	0.33786848	0.5804802	1	516	tags=65%,
HP_CHOLESTASIS	23	0.269	1.098	0.34553775	0.58257085	1	725	tags=91%,
GOBP_B_CELL_DIFFERENTIATION	17	0.298	1.098	0.3268817	0.58157974	1	98	tags=24%,
HP_ABNORMAL_THROMBOSIS	19	0.285	1.097	0.33258426	0.58229077	1	191	tags=32%,
GOMF_CELL_ADHESION_MOLECULE_BINDING	75	0.193	1.096	0.32828283	0.58240014	1	322	tags=37%,
HP_ABNORMALITY_OF_THE_LOWER_URINARY_TRACT	49	0.208	1.090	0.3181818	0.59379953	1	113	tags=14%,

HP_ABNORMALITY_OF_MOUTH_SHAPE	18	0.280	1.088	0.37089202	0.5980914	1	794	tags=94%,
GOBP_CELLULAR_RESPONSE_TO_TOXIC_SUBSTANCE	20	0.277	1.085	0.3355856	0.60257846	1	241	tags=45%,
HP_HEPATOMEGALY	60	0.195	1.083	0.34529147	0.6055899	1	218	tags=27%,
GOBP_ENTRY_INTO_HOST	17	0.279	1.077	0.33482143	0.6172981	1	167	tags=35%,
GOBP_REGULATION_OF_DNA_METABOLIC_PROCESS	33	0.238	1.077	0.37416482	0.61708295	1	214	tags=27%,
GOBP_PROTEIN_COMPLEX_OLIGOMERIZATION	16	0.292	1.075	0.35746607	0.6189844	1	77	tags=19%,
GOCC_ENDOCYTIC_VESICLE	45	0.214	1.075	0.37327188	0.6178415	1	221	tags=29%,
GOBP_DETOXIFICATION	20	0.277	1.071	0.367713	0.6241322	1	241	tags=45%,
GOBP_CELL_SURFACE_RECEPTOR_SIGNALING_PATHWAY_INVOLVED_IN_CELL_CI	52	0.201	1.071	0.3566265	0.6230909	1	280	tags=33%,
GOCC_PLASMA_MEMBRANE_PROTEIN_COMPLEX	50	0.204	1.071	0.35307518	0.62199616	1	221	tags=24%,
HP_SPLENOMEGALY	54	0.198	1.070	0.38173303	0.6236553	1	163	tags=22%,
GOCC_TRANSPORT_VESICLE_MEMBRANE	15	0.292	1.069	0.38222224	0.6230092	1	31	tags=13%,
HP_LYMPHOMA	17	0.287	1.066	0.36136365	0.6289781	1	259	tags=35%,
HP_ABNORMALITY_OF_THE_MIDDLE_EAR	48	0.206	1.062	0.38139534	0.6356695	1	98	tags=15%,
HP_CONSTIPATION	26	0.243	1.061	0.3711584	0.6380717	1	925	tags=100%,
GOBP_ENDOCYTOSIS	78	0.182	1.060	0.39007092	0.6372651	1	227	tags=23%,
HP_ABNORMAL_ESOPHAGUS_MORPHOLOGY	16	0.293	1.059	0.3812636	0.63938856	1	196	tags=31%,
HP_REDUCED_CONSCIOUSNESS_CONFUSION	21	0.273	1.059	0.41150442	0.6384903	1	576	tags=86%,
GOBP_PROTEIN_DNA_COMPLEX_SUBUNIT_ORGANIZATION	16	0.304	1.056	0.39101124	0.6436146	1	78	tags=19%,
GOCC_ENDOPLASMIC_RETICULUM	133	0.164	1.052	0.37292817	0.6503764	1	314	tags=33%,
HP_ABNORMAL_LARGE_INTESTINE_MORPHOLOGY	26	0.248	1.051	0.41371158	0.6501399	1	285	tags=35%,
GOCC_CLATHRIN_COATED_VESICLE	21	0.262	1.050	0.4004193	0.65230554	1	227	tags=33%,
GOCC_INTRINSIC_COMPONENT_OF_ORGANELLE_MEMBRANE	33	0.231	1.048	0.37871286	0.6542381	1	256	tags=30%,
GOBP_POSITIVE_REGULATION_OF_LEUKOCYTE_PROLIFERATION	19	0.270	1.048	0.3963039	0.65478903	1	189	tags=32%,
HP_ABNORMALITY_OF_THE_LYMPHATIC_SYSTEM	78	0.179	1.045	0.38071066	0.65855217	1	201	tags=23%,
REACTOME_ANTI_INFLAMMATORY_RESPONSE_FAVOURING_LEISHMANIA_PARASI	15	0.293	1.044	0.4214876	0.65950674	1	3	tags=7%, lis
GOBP_APOPTOTIC_SIGNALING_PATHWAY	76	0.182	1.044	0.3826291	0.65938365	1	162	tags=18%,
HP_ABNORMALITY_OF_PULMONARY_CIRCULATION	19	0.270	1.043	0.385567	0.6589555	1	255	tags=37%,
GOBP_MOVEMENT_IN_HOST_ENVIRONMENT	17	0.279	1.042	0.39461884	0.66004187	1	167	tags=35%,
GOBP_NEGATIVE_REGULATION_OF_IMMUNE_RESPONSE	19	0.266	1.042	0.38913044	0.6601017	1	268	tags=42%,
HP_ABNORMALITY_OF_THE_MENSTRUAL_CYCLE	17	0.275	1.041	0.41555557	0.6601651	1	94	tags=18%,
GOBP_RNA_PROCESSING	66	0.190	1.041	0.40102828	0.6593591	1	486	tags=55%,
GOBP_ORGANOPHOSPHATE_BIOSYNTHETIC_PROCESS	48	0.203	1.040	0.40632603	0.65862954	1	253	tags=31%,
REACTOME_DISEASES_OF_SIGNAL_TRANSDUCTION_BY_GROWTH_FACTOR_REC	55	0.202	1.036	0.3941606	0.6675306	1	111	tags=16%,
GOBP_PROTEIN_LOCALIZATION_TO_NUCLEUS	22	0.248	1.034	0.41469195	0.67089844	1	214	tags=32%,
HP_FEVER	47	0.201	1.033	0.4205379	0.6708662	1	163	tags=21%,
HP_FUNCTIONAL_MOTOR_DEFICIT	28	0.237	1.033	0.42592594	0.6698505	1	516	tags=64%,
HP_APNEA	18	0.277	1.033	0.4037123	0.66937214	1	475	tags=67%,
GOBP_MACROPHAGE_ACTIVATION	19	0.261	1.028	0.4232558	0.67870784	1	84	tags=16%,
HP_VOMITING	17	0.272	1.027	0.4375	0.6797074	1	555	tags=76%,
GOBP_REGULATION_OF_RESPONSE_TO_DNA_DAMAGE_STIMULUS	18	0.266	1.020	0.40929204	0.6925186	1	107	tags=17%,
REACTOME_MEMBRANE_TRAFFICKING	59	0.185	1.016	0.44730678	0.700165	1	537	tags=66%,
HP_HIGH_PALATE	42	0.202	1.016	0.45498782	0.69953084	1	787	tags=90%,
HP_ABNORMAL_SIZE_OF_THE_PALPEBRAL_FISSURES	15	0.286	1.014	0.43326038	0.7025362	1	680	tags=87%,
GOBP_CELL_KILLING	20	0.252	1.013	0.43811882	0.7027241	1	173	tags=30%,
GOBP_REGULATION_OF_CELLULAR_AMIDE_METABOLIC_PROCESS	47	0.201	1.008	0.44417477	0.71256655	1	303	tags=40%,
HP_ABNORMALITY_OF_BLOOD_AND_BLOOD_FORMING_TISSUES	124	0.158	1.007	0.42610836	0.7138104	1	164	tags=18%,
GOCC_MEMBRANE_MICRODOMAIN	44	0.198	1.006	0.44691357	0.7134257	1	189	tags=23%,
HP_PARAPLEGIA_PARAPARESIS	20	0.247	1.000	0.4543379	0.7268199	1	300	tags=40%,
HP_INCREASED_CIRCULATING_ANTIBODY_LEVEL	17	0.261	0.999	0.4321267	0.7265065	1	259	tags=35%,
HP_ABNORMAL_ABDOMEN_MORPHOLOGY	84	0.167	0.995	0.47921762	0.7335322	1	218	tags=24%,
HP_ABNORMAL_HAIR_PATTERN	24	0.233	0.992	0.4416476	0.7387863	1	789	tags=96%,
HP_HYPERREFLEXIA	38	0.205	0.987	0.4784689	0.74917805	1	300	tags=37%,
GOBP_MITOTIC_CELL_CYCLE	81	0.167	0.985	0.49447513	0.7527933	1	118	tags=17%,
HP_ABNORMALITY_OF_ESOPHAGUS_PHYSIOLOGY	46	0.194	0.984	0.48456058	0.7520659	1	549	tags=65%,
GOBP_NEGATIVE_REGULATION_OF_CELL_CYCLE	67	0.178	0.983	0.4917647	0.75437015	1	111	tags=16%,
GOBP_REGULATION_OF_ACTIN_FILAMENT_ORGANIZATION	29	0.224	0.981	0.48701298	0.7567791	1	324	tags=41%,
HP_ABNORMALITY_OF_DIGESTIVE_SYSTEM_MORPHOLOGY	67	0.173	0.977	0.47381547	0.7644211	1	98	tags=13%,
GOBP_DNA_BIOSYNTHETIC_PROCESS	17	0.261	0.977	0.4923077	0.76362157	1	312	tags=47%,
HP_INFANTILE_ONSET	39	0.196	0.976	0.5048309	0.7643593	1	110	tags=15%,
GOCC_MICROTUBULE_ORGANIZING_CENTER	46	0.185	0.975	0.50629723	0.7641915	1	127	tags=15%,
HP_ABNORMAL_RESPIRATORY_SYSTEM_PHYSIOLOGY	101	0.158	0.975	0.49874687	0.7632414	1	98	tags=13%,
HP_PROMINENT_FOREHEAD	17	0.258	0.973	0.49889624	0.76607776	1	784	tags=94%,
HP_ABNORMALITY_OF_THE_ZYGOMATIC_BONE	17	0.259	0.969	0.48723897	0.7729632	1	425	tags=53%,
GOCC_COATED_VESICLE	32	0.214	0.967	0.4917647	0.7763424	1	227	tags=25%,
GOBP_PROCESS_UTILIZING_AUTOPHAGIC_MECHANISM	36	0.202	0.966	0.49746192	0.7770416	1	201	tags=22%,
GOBP_REGULATION_OF_LEUKOCYTE_PROLIFERATION	33	0.209	0.964	0.48314607	0.7801325	1	84	tags=15%,
GOBP_POSITIVE_REGULATION_OF_PROTEIN_CONTAINING_COMPLEX_ASSEMBLY	17	0.260	0.963	0.49889135	0.78101605	1	307	tags=41%,
GOMF_CATALYTIC_ACTIVITY_ACTING_ON_RNA	15	0.274	0.962	0.5113636	0.7828217	1	886	tags=100%,
GOBP_PROTEIN_MODIFICATION_BY_SMALL_PROTEIN_CONJUGATION	67	0.170	0.959	0.5418719	0.78734994	1	111	tags=16%,
GOCC_RIBONUCLEOPROTEIN_GRANULE	31	0.215	0.958	0.52705884	0.78760463	1	299	tags=42%,
HP_GASTROESOPHAGEAL_REFLUX	24	0.231	0.958	0.5272727	0.78671825	1	607	tags=75%,
GOBP_AGING	34	0.205	0.955	0.51927435	0.7907954	1	251	tags=32%,
GOBP_CARBOHYDRATE_DERIVATIVE_METABOLIC_PROCESS	79	0.165	0.955	0.5393795	0.79051614	1	415	tags=49%,
HP_ABNORMALITY_OF_THE_LYMPH_NODES	44	0.193	0.954	0.5452436	0.7910207	1	163	tags=23%,
HP_ABNORMAL_VASCULAR_PHYSIOLOGY	25	0.223	0.951	0.50672644	0.7972315	1	377	tags=48%,
HP_HYPOPIGMENTATION_OF_THE_SKIN	17	0.258	0.949	0.5112613	0.7992226	1	66	tags=18%,
GOBP_RESPONSE_TO_ENDOPLASMIC_RETICULUM_STRESS	27	0.219	0.949	0.5102975	0.7978647	1	548	tags=70%,
GOBP_CELLULAR_RESPONSE_TO_EXTRACELLULAR_STIMULUS	20	0.235	0.949	0.5509259	0.79644877	1	2	tags=5%, lis
HP_ABNORMAL_URINE_CYTOLOGY	19	0.245	0.948	0.53691274	0.7967704	1	102	tags=16%,
HP_ABNORMAL_PENIS_MORPHOLOGY	41	0.195	0.945	0.52505964	0.8029808	1	259	tags=27%,

REACTOME_SUMOYLATION	15	0.265	0.944	0.5264368	0.8043833	1	632	tags=80%, l
GOBP_RECEPTOR_MEDIATED_ENDOCYTOSIS	44	0.190	0.942	0.52816904	0.80658674	1	15	tags=7%, lis
GOBP_REGULATION_OF_CELL_CYCLE_G1_S_PHASE_TRANSITION	18	0.250	0.942	0.5558036	0.80533755	1	99	tags=22%, l
HP_FATIGUE	45	0.187	0.940	0.57544756	0.80675215	1	26	tags=9%, lis
GOMF_ANTIOXIDANT_ACTIVITY	16	0.257	0.940	0.53139013	0.8057253	1	241	tags=44%, l
GOBP_POSITIVE_REGULATION_OF_CYTOSKELETON_ORGANIZATION	21	0.232	0.934	0.5321508	0.8176547	1	307	tags=38%, l
GOBP_VESICLE_ORGANIZATION	26	0.214	0.930	0.56626505	0.8256979	1	37	tags=12%, l
GOCC_MICROTUBULE_CYTOSKELETON	79	0.163	0.929	0.5753086	0.8262438	1	127	tags=16%, l
HP_ABNORMALITY_OF_THE_CURVATURE_OF_THE_VERTEBRAL_COLUMN	73	0.159	0.927	0.59753084	0.8298211	1	1007	tags=99%, l
HP_ABNORMAL_FINGERNAIL_MORPHOLOGY	16	0.259	0.924	0.5358744	0.83330053	1	26	tags=13%, l
HP_ABNORMALITY_OF_THE_AMNIOTIC_FLUID	16	0.251	0.923	0.5474614	0.83394325	1	118	tags=19%, l
REACTOME_POTENTIAL_THERAPEUTICS_FOR_SARS	18	0.239	0.923	0.54767185	0.83267826	1	271	tags=33%, l
HP_SPARSE_HAIR	26	0.211	0.919	0.56289977	0.83916086	1	47	tags=12%, l
HP_CONSTITUTIONAL_SYMPTOM	84	0.155	0.919	0.6331776	0.83959043	1	98	tags=12%, l
HP_CEREBELLAR_MALFORMATION	18	0.237	0.918	0.5472973	0.83841693	1	753	tags=89%, l
HP_ABNORMALITY_OF_TEMPERATURE_REGULATION	52	0.174	0.916	0.6074074	0.8411199	1	163	tags=19%, l
HP_ABNORMALITY_OF_COAGULATION	20	0.230	0.913	0.5651214	0.8473821	1	654	tags=80%, l
GOBP_PROTEIN_MODIFICATION_BY_SMALL_PROTEIN_CONJUGATION_OR_REMOV	79	0.158	0.912	0.6081731	0.84707373	1	111	tags=15%, l
GOBP_CATION_TRANSPORT	75	0.158	0.912	0.6425	0.8472145	1	304	tags=35%, l
HP_ABNORMAL_NUMBER_OF_TEETH	23	0.218	0.909	0.5816092	0.85177135	1	196	tags=22%, l
GOBP_CELLULAR_OXIDANT_DETOXIFICATION	17	0.243	0.907	0.5717439	0.8541801	1	241	tags=41%, l
GOBP_REGULATION_OF_CELLULAR_PROTEIN_CATABOLIC_PROCESS	27	0.211	0.907	0.62560385	0.8527759	1	109	tags=19%, l
GOBP_REGULATION_OF_MRNA_PROCESSING	19	0.229	0.905	0.59359604	0.85465753	1	464	tags=58%, l
HP_MUSCLE_WEAKNESS	71	0.157	0.904	0.63844395	0.857127	1	516	tags=58%, l
GOMF_DNA_BINDING_TRANSCRIPTION_REPRESSOR_ACTIVITY	20	0.234	0.903	0.6	0.8570748	1	2	tags=5%, lis
HP_HEMATOLOGICAL_NEOPLASM	31	0.198	0.902	0.5883721	0.85798705	1	163	tags=19%, l
HP_ABNORMALITY_OF_GLOBE_LOCATION	63	0.166	0.901	0.6315789	0.85724604	1	118	tags=13%, l
REACTOME_TRANS_GOLGI_NETWORK_VESICLE_BUDDING	15	0.253	0.899	0.58651686	0.85977805	1	227	tags=40%, l
HP_POSTERIORLY_ROTATED_EARS	19	0.232	0.898	0.5922551	0.8608432	1	288	tags=32%, l
HP_CLINICAL_COURSE	112	0.145	0.898	0.66917294	0.85946816	1	113	tags=13%, l
HP_ABNORMALITY_OF_THE_CERVICAL_SPINE	25	0.214	0.897	0.61173815	0.86034405	1	118	tags=16%, l
HP_ONSET	91	0.149	0.892	0.65829146	0.86836	1	113	tags=14%, l
GOBP_POSITIVE_REGULATION_OF_CELLULAR_PROTEIN_LOCALIZATION	30	0.196	0.890	0.59142214	0.8712367	1	415	tags=53%, l
HP_WEAKNESS_DUE_TO_UPPER_MOTOR_NEURON_DYSFUNCTION	34	0.194	0.890	0.6311111	0.8710607	1	110	tags=18%, l
HP_BRUISING_SUSCEPTIBILITY	23	0.219	0.890	0.57596374	0.8696345	1	94	tags=13%, l
HP_NEOPLASM	76	0.152	0.889	0.6567164	0.8698204	1	113	tags=13%, l
HP_ABNORMAL_PATTERN_OF_RESPIRATION	23	0.217	0.889	0.6270784	0.8687535	1	575	tags=74%, l
HP_DOWNSLANTED_PALPEBRAL_FISSURES	37	0.191	0.888	0.61678004	0.86797094	1	616	tags=73%, l
GOBP_NUCLEAR_TRANSPORT	30	0.197	0.887	0.6215054	0.86942697	1	385	tags=50%, l
HP_LOWER_LIMB_SPASTICITY	17	0.235	0.886	0.5955056	0.87007797	1	300	tags=41%, l
REACTOME_TRANSCRIPTIONAL_REGULATION_BY_TP53	48	0.174	0.886	0.6354167	0.8693572	1	342	tags=40%, l
HP_LEUKOENCEPHALOPATHY	19	0.227	0.886	0.5852535	0.86795056	1	463	tags=63%, l
HP_PEDIATRIC_ONSET	49	0.169	0.885	0.6453089	0.86841786	1	110	tags=14%, l
GOCC_CELL_BODY	35	0.186	0.884	0.6164383	0.8690059	1	278	tags=34%, l
GOBP_DNA_CONFORMATION_CHANGE	24	0.210	0.879	0.61981565	0.8765471	1	516	tags=63%, l
GOBP_NUCLEAR_EXPORT	17	0.235	0.878	0.60674155	0.87791514	1	228	tags=35%, l
GOBP_MACROMOLECULE_CATABOLIC_PROCESS	142	0.134	0.878	0.6984536	0.87663615	1	300	tags=30%, l
HP_ABNORMALITY_OF_THE_CURVATURE_OF_THE_CORNEA	15	0.249	0.877	0.59292036	0.8770189	1	916	tags=100%,
GOBP_ACTIN_FILAMENT_BUNDLE_ORGANIZATION	18	0.230	0.875	0.63656884	0.8782651	1	264	tags=33%, l
GOBP_SPHINGOLIPID_METABOLIC_PROCESS	15	0.242	0.875	0.60491073	0.87707365	1	386	tags=53%, l
HP_THICK_VERMILION_BORDER	21	0.219	0.872	0.5925926	0.881609	1	616	tags=76%, l
HP_ABNORMALITY_OF_THE_GINGIVA	25	0.203	0.872	0.62910795	0.88026816	1	156	tags=20%, l
GOBP_CELL_REDOX_HOMEOSTASIS	15	0.247	0.872	0.63053095	0.88000906	1	200	tags=33%, l
HALLMARK_COMPLEMENT	33	0.190	0.868	0.6481481	0.8851208	1	252	tags=30%, l
GOBP_NEGATIVE_REGULATION_OF_GENE_EXPRESSION	120	0.136	0.867	0.7102564	0.88600975	1	163	tags=18%, l
GOCC_INTRACELLULAR_PROTEIN_CONTAINING_COMPLEX	62	0.159	0.867	0.6940874	0.88502884	1	114	tags=15%, l
HP_BABINSKI_SIGN	20	0.218	0.867	0.625	0.8842002	1	293	tags=35%, l
HP_SHORT_NOSE	18	0.234	0.866	0.66588235	0.88378245	1	771	tags=89%, l
GOBP_NEGATIVE_REGULATION_OF_LYMPHOCYTE_ACTIVATION	25	0.208	0.865	0.6380091	0.8840472	1	11	tags=8%, lis
GOBP_REGULATION_OF_RNA_SPLICING	23	0.207	0.863	0.6395349	0.8870482	1	820	tags=91%, l
HALLMARK_G2M_CHECKPOINT	17	0.235	0.862	0.63539445	0.8868396	1	179	tags=29%, l
HP_ABNORMALITY_OF_THE_PINNA	47	0.172	0.860	0.69285715	0.8889892	1	447	tags=47%, l
GOBP_INTEGRIN_MEDIATED_SIGNALING_PATHWAY	18	0.227	0.852	0.6600877	0.9029021	1	943	tags=100%,
HP_ABNORMAL_INVOLUNTARY_EYE_MOVEMENTS	54	0.162	0.852	0.71004564	0.9026019	1	916	tags=94%, l
HP_JAUNDICE	17	0.224	0.848	0.63238513	0.907363	1	725	tags=88%, l
GOBP_REGULATION_OF_ACTIN_FILAMENT_LENGTH	20	0.218	0.848	0.65759635	0.90593946	1	324	tags=45%, l
GOCC_LAMELLIPODIUM	28	0.195	0.846	0.6839729	0.90815824	1	323	tags=39%, l
HP_ABNORMALITY_OF_THE_PITUITARY_GLAND	16	0.230	0.841	0.66741574	0.9172176	1	511	tags=69%, l
HP_HIRSUTISM	16	0.228	0.841	0.6530612	0.9161957	1	590	tags=75%, l
HP_ABNORMALITY_OF_THE_HYPOTHALAMUS_PITUITARY_AXIS	16	0.230	0.840	0.65410197	0.9152621	1	511	tags=69%, l
GOBP_NEGATIVE_REGULATION_OF_GROWTH	20	0.211	0.840	0.6883721	0.91463363	1	51	tags=10%, l
HP_FUNCTIONAL_ABNORMALITY_OF_THE_MIDDLE_EAR	23	0.201	0.840	0.7123288	0.91319895	1	975	tags=100%,
GOMF_ENZYME_ACTIVATOR_ACTIVITY	60	0.157	0.837	0.6868009	0.91719586	1	85	tags=12%, l
HP_BONE_PAIN	17	0.230	0.837	0.6652174	0.9160342	1	47	tags=12%, l
GOBP_CELL_CYCLE_G1_S_PHASE_TRANSITION	22	0.207	0.833	0.6792453	0.92172	1	99	tags=18%, l
GOBP_CARBOHYDRATE_DERIVATIVE_CATABOLIC_PROCESS	17	0.221	0.833	0.6315789	0.92050976	1	249	tags=35%, l
HP_ABNORMALITY_OF_FLUID_REGULATION	57	0.156	0.831	0.74396133	0.9216695	1	113	tags=14%, l
GOCC_POLYMERIC_CYTOSKELETAL_FIBER	49	0.162	0.831	0.7223529	0.92123735	1	127	tags=16%, l
GOBP_ACTIN_POLYMERIZATION_OR_DEPOLYMERIZATION	27	0.187	0.828	0.70842826	0.92447126	1	324	tags=41%, l
GOCC_ORGANELLE_SUBCOMPARTMENT	125	0.130	0.826	0.77970296	0.9267564	1	284	tags=26%, l
HP_PANCYTOPENIA	19	0.212	0.824	0.7078652	0.92855096	1	163	tags=21%, l

HP_ABNORMAL_SHAPE_OF_THE_FRONTAL_REGION	27	0.190	0.820	0.7155963	0.93465406	1	749	tags=85%, l
REACTOME_CLATHRIN_MEDIATED_ENDOCYTOSIS	19	0.211	0.820	0.70095694	0.9335594	1	601	tags=74%, l
HP_ABNORMAL_EYELID_MORPHOLOGY	73	0.146	0.819	0.74623114	0.93359876	1	298	tags=27%, l
GOMF_ACTIN_FILAMENT_BINDING	23	0.198	0.819	0.7126697	0.9324453	1	324	tags=43%, l
HP_RETROGNATHIA	17	0.218	0.809	0.6768559	0.94899595	1	206	tags=24%, l
GOBP_SUPEROXIDE_METABOLIC_PROCESS	17	0.216	0.806	0.67180616	0.95258844	1	211	tags=29%, l
HP_ABNORMAL_THYROID_MORPHOLOGY	16	0.217	0.805	0.70669746	0.9518938	1	259	tags=31%, l
HP_ABNORMAL_LOCATION_OF_EARS	44	0.158	0.799	0.74820143	0.9610376	1	118	tags=14%, l
GOBP_NEGATIVE_REGULATION_OF_CYTOSKELETON_ORGANIZATION	15	0.225	0.798	0.7061311	0.9609812	1	190	tags=27%, l
HP_PTOSIS	52	0.150	0.796	0.74584323	0.9622373	1	1040	tags=100%
GOCC_CENTROSOME	36	0.170	0.796	0.72283816	0.96170235	1	127	tags=14%, l
HP_ABNORMAL_FEMALE_REPRODUCTIVE_SYSTEM_PHYSIOLOGY	23	0.193	0.794	0.7617977	0.9636752	1	94	tags=13%, l
HP_MYOPIA	23	0.190	0.793	0.7470726	0.9635224	1	534	tags=65%, l
GOBP_RESPONSE_TO_TEMPERATURE_STIMULUS	22	0.198	0.792	0.7327189	0.96427864	1	293	tags=32%, l
HP_BILATERAL_TONIC_CLONIC_SEIZURE	19	0.204	0.789	0.7307692	0.9666037	1	607	tags=74%, l
HP_LIMB_UNDERGROWTH	15	0.218	0.787	0.7161017	0.9684054	1	469	tags=60%, l
GOBP_REGULATION_OF_ACTIN_FILAMENT_BASED_PROCESS	41	0.159	0.787	0.79097384	0.96741265	1	324	tags=37%, l
GOBP_SULFUR_COMPOUND_METABOLIC_PROCESS	25	0.184	0.786	0.7505774	0.9670131	1	277	tags=36%, l
GOBP_CELL_CYCLE_PROCESS	105	0.128	0.786	0.81407034	0.9660465	1	118	tags=13%, l
GOBP_REGULATION_OF_PROTEIN_CONTAINING_COMPLEX_ASSEMBLY	39	0.163	0.785	0.75961536	0.9660956	1	141	tags=18%, l
HP_LEUKEMIA	16	0.216	0.784	0.72911966	0.96611863	1	163	tags=19%, l
GOBP_DNA_RECOMBINATION	17	0.210	0.782	0.7301927	0.96782035	1	866	tags=94%, l
HP_APLASIA_HYPOPLASIA_OF_THE_CEREBELLUM	15	0.218	0.779	0.726087	0.9712873	1	509	tags=67%, l
GOBP_NCRNA_METABOLIC_PROCESS	19	0.200	0.777	0.73277664	0.972766	1	975	tags=100%
HP_HYPERTROPHIC_CARDIOMYOPATHY	24	0.188	0.776	0.74449337	0.9731517	1	463	tags=63%, l
GOBP_REGULATION_OF_ANATOMICAL_STRUCTURE_MORPHOGENESIS	93	0.128	0.774	0.86649877	0.9743784	1	332	tags=34%, l
HP_CONGENITAL_ONSET	27	0.176	0.773	0.75114155	0.9758195	1	298	tags=37%, l
HP_ABNORMAL_CARDIOVASCULAR_SYSTEM_PHYSIOLOGY	97	0.126	0.765	0.874092	0.98616016	1	113	tags=11%, l
GOMF_KINASE_REGULATOR_ACTIVITY	30	0.169	0.764	0.77367204	0.9858915	1	51	tags=10%, l
HP_DYSTONIA	30	0.170	0.763	0.7708333	0.9855329	1	300	tags=33%, l
HP_ABNORMALITY_OF_THE_HAIRLINE	18	0.194	0.757	0.78959274	0.9936946	1	789	tags=94%, l
HP_FUNCTIONAL_ABNORMALITY_OF_THE_GASTROINTESTINAL_TRACT	81	0.130	0.757	0.8630807	0.99249023	1	94	tags=10%, l
HP_ABNORMALITY_OF_GLOBE_SIZE	20	0.190	0.756	0.7860577	0.9911639	1	206	tags=25%, l
HP_SUBCUTANEOUS_HEMORRHAGE	34	0.162	0.755	0.8116592	0.9918001	1	94	tags=12%, l
HP_IRREGULAR_HYPERPIGMENTATION	19	0.196	0.755	0.753915	0.99048984	1	206	tags=26%, l
GOBP_NCRNA_PROCESSING	18	0.200	0.755	0.76082003	0.98923284	1	975	tags=100%
HP_INTELLECTUAL_DISABILITY_MILD	29	0.171	0.753	0.7832168	0.99094725	1	437	tags=48%, l
GOCC_GOLGI_APPARATUS	124	0.115	0.752	0.9195122	0.99035335	1	41	tags=6%, lis
GOBP_REGULATION_OF_PROTEIN_CONTAINING_COMPLEX_DISASSEMBLY	15	0.210	0.752	0.76748973	0.9890855	1	190	tags=27%, l
GOBP_MEMBRANE_LIPID_METABOLIC_PROCESS	18	0.197	0.751	0.77272725	0.98832583	1	390	tags=50%, l
GOCC_DENDRITIC_TREE	38	0.158	0.748	0.7914692	0.9914849	1	384	tags=39%, l
GOBP_CYTOSOLIC_CALCIUM_ION_TRANSPORT	15	0.206	0.747	0.7802198	0.9919347	1	149	tags=20%, l
GOCC_NUCLEAR_PROTEIN_CONTAINING_COMPLEX	92	0.128	0.747	0.8967391	0.9906832	1	501	tags=54%, l
HP_INCOORDINATION	22	0.184	0.743	0.7923251	0.9945564	1	91	tags=14%, l
HP_OSTEOPOROSIS	21	0.187	0.741	0.7772727	0.99535704	1	39	tags=10%, l
HP_NEOPLASM_OF_THE_NERVOUS_SYSTEM	18	0.192	0.741	0.7809111	0.9944797	1	984	tags=100%
GOCC_TRANSPORT_VESICLE	33	0.161	0.740	0.8329356	0.99454373	1	654	tags=76%, l
HP_INTRAUTERINE_GROWTH_RETARDATION	31	0.163	0.739	0.8257757	0.9942676	1	463	tags=55%, l
HP_ABNORMAL_JOINT_MORPHOLOGY	76	0.131	0.738	0.87764704	0.994847	1	118	tags=13%, l
HP_ABNORMALITY_OF_THE_PALPEBRAL_FISSURES	48	0.142	0.734	0.85121953	0.9979344	1	734	tags=81%, l
HP_AUTISM	16	0.192	0.734	0.7821101	0.9966454	1	984	tags=100%
HP_ABNORMALITY_OF_COORDINATION	75	0.126	0.731	0.86292136	0.99965703	1	463	tags=49%, l
HP_PURPURA	19	0.191	0.730	0.7987152	0.99866337	1	94	tags=16%, l
GOBP_REGULATION_OF_SUPRAMOLECULAR_FIBER_ORGANIZATION	37	0.155	0.729	0.84598213	0.9986812	1	324	tags=35%, l
GOMF_TRANSCRIPTION_COACTIVATOR_ACTIVITY	23	0.175	0.728	0.8156682	0.99962074	1	28	tags=9%, lis
HP_ABNORMALITY_OF_THE_CHEEK	18	0.187	0.727	0.8325792	0.9987748	1	790	tags=89%, l
HP_HYPERMELANOTIC_MACULE	15	0.200	0.726	0.7652812	0.99825466	1	156	tags=20%, l
HP_SPECIFIC_LEARNING_DISABILITY	19	0.183	0.726	0.82758623	0.99765027	1	734	tags=84%, l
HP_LOW_SET_POSTERIORLY_ROTATED_EARS	15	0.200	0.724	0.7760532	0.9984355	1	113	tags=13%, l
HP_APLASIA_HYPOPLASIA_OF_TOE	15	0.203	0.724	0.8249453	0.9978127	1	15	tags=7%, lis
HP_PES_PLANUS	23	0.174	0.721	0.8186275	1	1	1007	tags=100%
HP_ABNORMALITY_OF_THE_FOREHEAD	58	0.135	0.720	0.87470996	0.9993292	1	794	tags=86%, l
GOBP_PROTEIN_PHOSPHOPANTETHEINYLATION	18	0.193	0.719	0.81333333	0.9994857	1	109	tags=17%, l
HP_GENERALIZED_ONSET_MOTOR_SEIZURE	15	0.203	0.712	0.81113803	1	1	800	tags=93%, l
HP_MISALIGNMENT_OF_TEETH	22	0.174	0.710	0.8211009	1	1	1007	tags=100%
HP_ABNORMALITY_OF_THE_CEREBRAL_SUBCORTEX	61	0.131	0.710	0.89548695	1	1	509	tags=57%, l
GOCC_SUPRAMOLECULAR_COMPLEX	83	0.122	0.708	0.9113924	1	1	280	tags=27%, l
HP_RECURRENT_FRACTURES	18	0.186	0.708	0.8340807	1	1	305	tags=33%, l
HP_MACROTIA	22	0.171	0.702	0.8618421	1	1	47	tags=9%, lis
GOCC_SUPRAMOLECULAR_POLYMER	56	0.134	0.701	0.8708861	1	1	127	tags=14%, l
GOCC_MICROTUBULE	28	0.159	0.696	0.8402626	1	1	117	tags=18%, l
HP_POOR_SPEECH	23	0.168	0.696	0.82539684	1	1	492	tags=61%, l
HP_ABNORMAL_TENDON_MORPHOLOGY	48	0.134	0.695	0.9014778	1	1	96	tags=10%, l
HP_ABNORMAL_PALATE_MORPHOLOGY	58	0.127	0.692	0.8752941	1	1	118	tags=12%, l
HP_ABNORMAL_BLOOD_GLUCOSE_CONCENTRATION	18	0.180	0.691	0.8430107	1	1	590	tags=78%, l
HP_ABNORMAL_FORM_OF_THE_VERTEBRAL_BODIES	21	0.174	0.686	0.8278867	1	1	1007	tags=100%
GOMF_RNA_POLYMERASE_II_SPECIFIC_DNA_BINDING_TRANSCRIPTION_FACTOR	30	0.157	0.686	0.8862559	1	1	99	tags=13%, l
HP_ABNORMAL_5TH_FINGER_MORPHOLOGY	29	0.157	0.686	0.8684211	1	1	1028	tags=100%
HP_ABNORMAL_EYEBROW_MORPHOLOGY	36	0.148	0.685	0.87857145	1	1	66	tags=8%, lis
HP_FINGER_CLINODACTYLY	26	0.157	0.682	0.84976524	1	1	1028	tags=100%
GOCC_ENDOSOME	100	0.112	0.681	0.92269325	1	1	227	tags=20%, l

GOBP_CELLULAR_MACROMOLECULE_LOCALIZATION	152	0.103	0.680	0.9650873	1	1	271	tags=24%, l
GOMF_CHROMATIN_BINDING	55	0.126	0.679	0.9014423	1	1	67	tags=7%, lis
HP_EPISTAXIS	16	0.180	0.677	0.8622222	1	1	94	tags=13%, l
GOMF_RIBONUCLEOTIDE_BINDING	121	0.104	0.675	0.95135134	1	1	113	tags=10%, l
HP_ABNORMALITY_OF_THE_BILIARY_SYSTEM	29	0.150	0.671	0.8744395	1	1	725	tags=83%, l
HP_GAIT_DISTURBANCE	67	0.120	0.670	0.93240094	1	1	516	tags=57%, l
HP_FEEDING_DIFFICULTIES_IN_INFANCY	32	0.146	0.669	0.87764704	1	1	1041	tags=100%,
GOBP_MACROAUTOPHAGY	18	0.176	0.669	0.8512931	1	1	263	tags=28%, l
GOCC_NUCLEAR_SPECK	36	0.142	0.669	0.88248336	1	1	2	tags=3%, lis
GOBP_PROTEIN_IMPORT	16	0.183	0.665	0.86098653	1	1	271	tags=38%, l
GOBP_POSITIVE_REGULATION_OF_DNA_METABOLIC_PROCESS	21	0.166	0.663	0.85314685	1	1	312	tags=33%, l
GOCC_CHROMOSOMAL_REGION	20	0.169	0.661	0.8856502	1	1	841	tags=90%, l
HP_APLASIA_HYPOPLASIA_AFFECTING_BONES_OF_THE_AXIAL_SKELETON	48	0.128	0.661	0.9146608	1	1	790	tags=83%, l
HALLMARK_ADIPOGENESIS	26	0.153	0.660	0.90553	1	1	450	tags=58%, l
GOCC_CILIUM	16	0.178	0.660	0.85547787	1	1	882	tags=94%, l
HP_FEEDING_DIFFICULTIES	60	0.121	0.660	0.9130435	1	1	1075	tags=100%,
HP_GENERALIZED_HYPOTONIA	67	0.121	0.659	0.9340909	1	1	1075	tags=100%,
HP_ABNORMAL_SYSTEMIC_BLOOD_PRESSURE	33	0.141	0.658	0.9032258	1	1	1048	tags=100%,
GOBP_POSITIVE_REGULATION_OF_CELLULAR_COMPONENT_BIOGENESIS	37	0.138	0.654	0.88435376	1	1	219	tags=22%, l
GOBP_CHROMATIN_REMODELING	18	0.171	0.648	0.876652	1	1	444	tags=56%, l
HP_ABNORMALITY_OF_FACIAL_SOFT_TISSUE	25	0.154	0.646	0.9297052	1	1	153	tags=16%, l
GOBP_ESTABLISHMENT_OF_PROTEIN_LOCALIZATION	151	0.099	0.646	0.97720796	1	1	249	tags=23%, l
HP_NARROW_MOUTH	20	0.163	0.645	0.90611356	1	1	435	tags=55%, l
GOBP_PROTEIN_LOCALIZATION_TO_PLASMA_MEMBRANE	24	0.152	0.642	0.8901345	1	1	51	tags=8%, lis
HP_CONTRACTURES_OF_THE_JOINTS_OF_THE_LOWER_LIMBS	16	0.179	0.641	0.89400923	1	1	91	tags=13%, l
GOMF_PROTEIN_DOMAIN_SPECIFIC_BINDING	73	0.112	0.638	0.9334917	1	1	113	tags=11%, l
GOBP_REGULATION_OF_CYTOSKELETON_ORGANIZATION	52	0.121	0.636	0.9373494	1	1	324	tags=33%, l
HP_SHORT_LONG_BONE	17	0.173	0.635	0.8964758	1	1	1007	tags=100%,
HP_ABNORMALITY_OF_MOUTH_SIZE	30	0.141	0.631	0.91460675	1	1	437	tags=53%, l
HP_SPASTICITY	52	0.117	0.629	0.9308756	1	1	543	tags=62%, l
GOBP_PROTEIN_LOCALIZATION_TO_CELL_PERIPHERY	25	0.147	0.628	0.902439	1	1	51	tags=8%, lis
GOBP_ESTABLISHMENT_OF_ORGANELLE_LOCALIZATION	32	0.135	0.626	0.9086651	1	1	88	tags=9%, lis
HP_INCREASED_BLOOD_PRESSURE	29	0.140	0.625	0.930131	1	1	1048	tags=100%,
HP_ABNORMALITY_OF_THE_CEREBRAL_VASCULATURE	26	0.149	0.623	0.8997494	1	1	94	tags=12%, l
HP_ABNORMAL_SOCIAL_BEHAVIOR	15	0.174	0.620	0.9130435	1	1	590	tags=80%, l
HP_INCREASED_SUSCEPTIBILITY_TO_FRACTURES	27	0.140	0.619	0.92743766	1	1	1048	tags=100%,
GOBP_MICROTUBULE_BASED_PROCESS	40	0.128	0.619	0.921875	1	1	117	tags=13%, l
HP_TALIPES_EQUINOVARUS	18	0.162	0.616	0.9162996	1	1	104	tags=11%, l
HP_ABNORMALITY_OF_THE_PERIPHERAL_NERVOUS_SYSTEM	16	0.169	0.615	0.9194915	1	1	1012	tags=100%,
HP_DELAYED_GROSS_MOTOR_DEVELOPMENT	20	0.154	0.613	0.9309577	1	1	549	tags=65%, l
HP_WEIGHT_LOSS	30	0.136	0.612	0.9370629	1	1	31	tags=7%, lis
HP_ABNORMAL_HEART_VALVE_MORPHOLOGY	22	0.147	0.609	0.9504505	1	1	1039	tags=100%,
HP_HYPERHIDROSIS	17	0.164	0.608	0.92954546	1	1	156	tags=18%, l
HP_ABNORMALITY_OF_THE_DENTITION	58	0.111	0.603	0.9487805	1	1	288	tags=26%, l
GOBP_ORGANELLE_LOCALIZATION	47	0.116	0.602	0.9390519	1	1	118	tags=11%, l
GOBP_HOMEOSTASIS_OF_NUMBER_OF_CELLS	33	0.131	0.599	0.92957747	1	1	39	tags=6%, lis
GOBP_PROTEIN_LOCALIZATION_TO_MEMBRANE	52	0.115	0.598	0.95555556	1	1	184	tags=17%, l
HP_ABNORMAL_JAW_MORPHOLOGY	58	0.112	0.596	0.9638243	1	1	790	tags=83%, l
GOBP_MICROTUBULE_CYTOSKELETON_ORGANIZATION	31	0.131	0.595	0.94239634	1	1	117	tags=13%, l
GOCC_SOMATODENDRITIC_COMPARTMENT	50	0.114	0.592	0.95642203	1	1	1023	tags=98%, l
HP_ABNORMALITY_OF_FINGER	69	0.105	0.590	0.962963	1	1	118	tags=12%, l
HP_ABNORMALITY_OF_THE_BLADDER	32	0.129	0.590	0.9277389	1	1	90	tags=9%, lis
HP_ABNORMALITY_OF_THE_BREAST	36	0.124	0.586	0.9277389	1	1	794	tags=86%, l
HP_COLOBOMA	15	0.154	0.577	0.95010394	1	1	1029	tags=100%,
HP_CRYPTORCHIDISM	42	0.119	0.576	0.9336493	1	1	1075	tags=100%,
HP_ABNORMALITY_OF_CIRCULATING_ENZYME_LEVEL	17	0.145	0.572	0.94930875	1	1	1040	tags=100%,
HP_ABNORMALITY_OF_THE_ELBOW	19	0.146	0.570	0.938914	1	1	1040	tags=100%,
HP_ABNORMAL_HAIR_MORPHOLOGY	77	0.096	0.570	0.98345155	1	1	66	tags=6%, lis
HP_ABNORMAL_CORPUS_CALLOSUM_MORPHOLOGY	45	0.113	0.567	0.9592326	1	1	509	tags=56%, l
GOMF_DNA_BINDING_TRANSCRIPTION_FACTOR_BINDING	39	0.115	0.566	0.97669	1	1	99	tags=10%, l
HP_DECREASED_HEAD_CIRCUMFERENCE	73	0.098	0.561	0.9814385	1	1	113	tags=10%, l
GOCC_NUCLEAR_OUTER_MEMBRANE_ENDOPLASMIC_RETICULUM_MEMBRANE_N	84	0.095	0.561	0.9832536	1	1	37	tags=5%, lis
GOMF_STRUCTURAL_MOLECULE_ACTIVITY	28	0.122	0.559	0.9544419	1	1	440	tags=50%, l
HP_ABNORMALITY_OF_URINE_HOMEOSTASIS	42	0.113	0.556	0.9560185	1	1	102	tags=10%, l
GOBP_GLAND_DEVELOPMENT	33	0.120	0.555	0.969555	1	1	399	tags=39%, l
GOMF_PROTEIN_HETERODIMERIZATION_ACTIVITY	18	0.147	0.553	0.9498861	1	1	291	tags=33%, l
HP_ABNORMAL_ATRIOVENTRICULAR_VALVE_PHYSIOLOGY	16	0.151	0.551	0.9284065	1	1	449	tags=56%, l
HP_ABNORMALITY_OF_BONE_MARROW_CELL_MORPHOLOGY	25	0.131	0.549	0.960739	1	1	163	tags=16%, l
HP_EXTERNAL_GENITAL_HYPOPLASIA	28	0.122	0.546	0.962963	1	1	259	tags=25%, l
HP_ABNORMAL_RENAL_PHYSIOLOGY	49	0.104	0.545	0.96713614	1	1	39	tags=6%, lis
HP_CEREBRAL_CORTICAL_ATROPHY	16	0.146	0.545	0.96255505	1	1	509	tags=63%, l
HP_ABNORMALITY_OF_HINDBRAIN_MORPHOLOGY	50	0.103	0.540	0.9809069	1	1	795	tags=82%, l
GOBP_ORGANELLE_ASSEMBLY	40	0.107	0.530	0.9738717	1	1	1039	tags=98%, l
HP_APLASIA_HYPOPLASIA_INVOLVING_BONES_OF_THE_FEET	22	0.129	0.527	0.9589041	1	1	91	tags=9%, lis
GOBP_DNA_REPAIR	29	0.118	0.523	0.97396964	1	1	266	tags=24%, l
HP_ABNORMALITY_OF_PRENATAL_DEVELOPMENT_OR_BIRTH	51	0.099	0.522	0.9896104	1	1	268	tags=22%, l
HP_ABNORMALITY_OF_TOE	46	0.102	0.520	0.9830508	1	1	66	tags=7%, lis
HP_ABNORMAL_THORAX_MORPHOLOGY	54	0.097	0.511	0.9728507	1	1	113	tags=9%, lis
HP_INVOLUNTARY_MOVEMENTS	71	0.089	0.510	0.9809069	1	1	110	tags=10%, l
GOBP_PROTEIN_CONTAINING_COMPLEX_LOCALIZATION	17	0.137	0.509	0.9769392	1	1	496	tags=59%, l
HP_DERMATOLOGICAL_MANIFESTATIONS_OF_SYSTEMIC_DISORDERS	20	0.128	0.507	0.9766355	1	1	725	tags=80%, l

HP_PAIN	67	0.090	0.505	0.99761903	1	1	96	tags=9%, lis
HP_HYPOPLASIA_OF_PENIS	24	0.121	0.504	0.98249453	1	1	259	tags=25%, l
HP_INTELLECTUAL_DISABILITY_SEVERE	26	0.116	0.501	0.9771167	1	1	607	tags=69%, l
HP_LARGE_FACE	16	0.133	0.500	0.9698376	1	1	680	tags=75%, l
HP_AREFLEXIA	17	0.135	0.497	0.9806452	1	1	897	tags=94%, l
KEGG_NEUROTROPHIN_SIGNALING_PATHWAY	20	0.123	0.493	0.9866667	1	1	784	tags=85%, l
GOBP_REGULATION_OF_PROTEIN_LOCALIZATION_TO_MEMBRANE	21	0.121	0.488	0.9742389	1	1	184	tags=19%, l
GOCC_NUCLEAR_BODY	63	0.089	0.487	0.9930233	1	1	2	tags=2%, lis
HP_STEREOTYPY	18	0.123	0.482	0.9886105	1	1	1067	tags=100%,
GOCC_ACTIN_FILAMENT	16	0.132	0.481	0.9804772	1	1	917	tags=94%, l
HP_SLOW_PROGRESSION	16	0.128	0.478	0.98630136	1	1	333	tags=38%, l
HP_ABNORMALITY_OF_THE_CEREBRAL_CORTEX	22	0.113	0.459	0.9912088	1	1	184	tags=18%, l
GOCC_ENDOSOME_MEMBRANE	51	0.087	0.456	0.9887387	1	1	221	tags=18%, l
HP_ABNORMAL_REFLEX	70	0.080	0.454	0.9884259	1	1	300	tags=26%, l
HP_ABNORMALITY_OF_THE_THYROID_GLAND	33	0.099	0.452	0.99775785	1	1	590	tags=67%, l
HP_WIDE_NASAL_BRIDGE	32	0.097	0.450	0.99546486	1	1	66	tags=6%, lis
HP_ABNORMAL_ORAL_PHYSIOLOGY	23	0.110	0.448	0.99040765	1	1	94	tags=9%, lis
HP_PACE_OF_PROGRESSION	36	0.095	0.446	1	1	1	549	tags=58%, l
HP_ABNORMAL_URINE_METABOLITE_LEVEL	30	0.103	0.443	0.99058825	1	1	102	tags=10%, l
HP_DYSPNEA	32	0.097	0.438	0.98675495	1	1	790	tags=81%, l
HP_ABNORMAL_TESTIS_MORPHOLOGY	56	0.082	0.434	0.9976798	1	1	26	tags=4%, lis
HP_SKELETAL_MUSCLE_ATROPHY	33	0.091	0.433	0.9926108	1	1	480	tags=48%, l
HP_TREMOR	30	0.095	0.429	0.9910314	1	1	61	tags=7%, lis
GOCC_DISTAL_AXON	18	0.112	0.427	0.99368423	1	1	599	tags=67%, l
HP_ABNORMALITY_OF_NEURONAL_MIGRATION	23	0.103	0.421	0.99770117	1	1	118	tags=13%, l
GOBP_REGULATION_OF_NEURON_DIFFERENTIATION	16	0.116	0.418	0.9955157	1	1	1075	tags=100%,
HP_ABNORMAL_SYSTEMIC_ARTERIAL_MORPHOLOGY	39	0.085	0.412	1	1	1	86	tags=8%, lis
HP_ABNORMAL_STERNUM_MORPHOLOGY	33	0.088	0.408	0.9954853	1	1	15	tags=3%, lis
HP_ABNORMAL_PYRAMIDAL_SIGN	29	0.091	0.402	0.99770117	1	1	300	tags=28%, l
HP_ABNORMALITY_OF_UPPER_LIMB_JOINT	35	0.084	0.399	0.9977375	1	1	1040	tags=97%, l
HP_ABNORMALITY_OF_LOWER_LIMB_JOINT	32	0.086	0.396	0.9905437	1	1	399	tags=38%, l
HP_MICROPENIS	16	0.105	0.384	1	1	1	259	tags=25%, l
HP_ABNORMALITY_OF_THE_PHILTRUM	41	0.073	0.358	1	1	1	725	tags=76%, l
GOCC_LEADING_EDGE_MEMBRANE	18	0.092	0.349	1	1	1	969	tags=94%, l
HP_ABNORMALITY_OF_THE_CALVARIA	65	0.061	0.349	1	0.9996114	1	1039	tags=95%, l
