# Supplementary material for: Biodegradable nanoparticles induce cGAS/STING-dependent reprogramming of myeloid cells to promote tumor immunotherapy
Source: Front Immunol. 2022 Aug 18;13:887649. doi: 10.3389/fimmu.2022.887649 (PMC9433741; doi:10.3389/fimmu.2022.887649)
Supplement: Supplementary file 10 [file Table_4.pdf]

Supplemental Table 4. Signaling Pathway Analysis for Macrophages - 3 Consecutive Doses - ONP-302 vs. Saline

NAME	SIZE	ES	NES	NOM p-val	FDR q-val	FWER p-val	RANK AT MA	LEADING EDG
HALLMARK_INTERFERON_GAMMA_RESPONSE	82	0.505	3.345	0	0	0	708	tags=66%, lis
HALLMARK_INTERFERON_ALPHA_RESPONSE	43	0.533	2.876	0	0	0	592	tags=63%, lis
REACTOME_INTERFERON_SIGNALING	46	0.469	2.624	0	0.001437942	0.005	534	tags=57%, lis
GOBP_NEGATIVE_REGULATION_OF_VIRAL_PROCESS	17	0.648	2.561	0	0.002143980	0.01	319	tags=59%, lis
REACTOME_ANTIVIRAL_MECHANISM_BY_IFN_STIMULATED_GENES	17	0.612	2.407	0	0.010650609	0.06	501	tags=71%, lis
REACTOME_ABC_FAMILY_PROTEINS_MEDIATED_TRANSPORT	26	0.483	2.274	0	0.042183474	0.261	964	tags=85%, lis
REACTOME_INTERFERON_ALPHA_BETA_SIGNALING	16	0.576	2.261	0	0.041074134	0.29	592	tags=69%, lis
KEGG_PROTEASOME	17	0.557	2.229	0	0.04944133	0.375	958	tags=94%, lis
GOBP_POSITIVE_REGULATION_OF_IMMUNE_RESPONSE	138	0.296	2.224	0	0.046413265	0.392	592	tags=41%, lis
GOBP_REGULATION_OF_VIRAL_LIFE_CYCLE	27	0.471	2.208	0.003838771	0.047181442	0.435	319	tags=44%, lis
GOBP_RESPONSE_TO_TYPE_I_INTERFERON	28	0.465	2.190	0	0.051314022	0.487	708	tags=61%, lis
REACTOME_INTERFERON_GAMMA_SIGNALING	22	0.493	2.187	0	0.048394162	0.492	264	tags=45%, lis
REACTOME_AUF1_HNRNP_D0_BINDS_AND_DESTABILIZES_MRNA	22	0.493	2.176	0	0.049824696	0.526	958	tags=86%, lis
GOBP_REGULATION_OF_INNATE_IMMUNE_RESPONSE	84	0.321	2.162	0	0.053195775	0.578	592	tags=44%, lis
GOBP_REGULATION_OF_IMMUNE_RESPONSE	189	0.276	2.156	0	0.053376902	0.601	592	tags=40%, lis
GOBP_POSITIVE_REGULATION_OF_RESPONSE_TO_CYTOKINE_STIMULUS	16	0.543	2.151	0.003968254	0.052136432	0.617	364	tags=56%, lis
REACTOME_ASSEMBLY_OF_THE_PRE_REPLICATIVE_COMPLEX	22	0.491	2.143	0.001919385	0.05260747	0.641	958	tags=86%, lis
GOBP_DEFENSE_RESPONSE_TO_VIRUS	63	0.353	2.139	0	0.05202321	0.657	1036	tags=71%, lis
GOBP_INTERFERON_GAMMA_MEDIATED_SIGNALING_PATHWAY	28	0.447	2.136	0	0.05063519	0.669	264	tags=39%, lis
GOBP_REGULATION_OF_BIOLOGICAL_PROCESS_INVOLVED_IN_SYMBIOTIC_INTERACTION	40	0.397	2.126	0	0.05223055	0.697	607	tags=50%, lis
REACTOME_SCF_SKP2_MEDIATED_DEGRADATION_OF_P27_P21	21	0.487	2.118	0	0.052682623	0.718	958	tags=86%, lis
GOBP_RESPONSE_TO_VIRUS	83	0.318	2.111	0	0.054227687	0.75	912	tags=59%, lis
REACTOME_CROSS_PRESENTATION_OF_SOLUBLE_EXOGENOUS_ANTIGENS_ENDOSOMES	19	0.506	2.105	0	0.054114334	0.758	958	tags=89%, lis
REACTOME_ABC_TRANSPORTER_DISORDERS	23	0.464	2.102	0	0.053039674	0.767	964	tags=83%, lis
REACTOME_ORC1_REMOVAL_FROM_CHROMATIN	23	0.471	2.092	0.003780718	0.05582417	0.797	958	tags=83%, lis
REACTOME_DEFECTIVE_CFTR_CAUSES_CYSTIC_FIBROSIS	23	0.464	2.078	0.001996008	0.060907513	0.841	964	tags=83%, lis
GOBP_ACTIVATION_OF_IMMUNE_RESPONSE	102	0.291	2.048	0	0.07480308	0.907	592	tags=41%, lis
REACTOME_DNA_REPLICATION_PRE_INITIATION	23	0.463	2.044	0	0.07443096	0.911	958	tags=83%, lis
GOBP_POSITIVE_REGULATION_OF_RESPONSE_TO_BIOTIC_STIMULUS	67	0.326	2.030	0	0.08064776	0.929	592	tags=45%, lis
GOBP_TUMOR_NECROSIS_FACTOR_MEDIATED_SIGNALING_PATHWAY	48	0.359	2.030	0.001858736	0.07798814	0.929	592	tags=50%, lis
GOBP_RESPONSE_TO_INTERFERON_GAMMA	45	0.371	2.016	0.003676470	0.085041	0.953	264	tags=33%, lis
REACTOME_HEDGEHOG_LIGAND_BIOGENESIS	22	0.462	2.014	0.003759398	0.08375219	0.955	958	tags=82%, lis
REACTOME_DECTIN_1_MEDIATED_NONCANONICAL_NF_KB_SIGNALING	22	0.452	2.010	0.001876172	0.08409938	0.962	958	tags=82%, lis
GOBP_RESPONSE_TO_INTERLEUKIN_1	48	0.351	1.976	0	0.10640093	0.986	617	tags=50%, lis
REACTOME_DEGRADATION_OF_DVL	21	0.462	1.974	0.007518797	0.10536907	0.986	958	tags=81%, lis
GOBP_REGULATION_OF_RESPONSE_TO_BIOTIC_STIMULUS	105	0.281	1.968	0	0.10732447	0.987	592	tags=40%, lis
GOBP_POSITIVE_REGULATION_OF_DEFENSE_RESPONSE	87	0.296	1.953	0	0.11674748	0.992	592	tags=41%, lis
REACTOME_ASYMMETRIC_LOCALIZATION_OF_PCP_PROTEINS	19	0.473	1.948	0.009225092	0.118168496	0.994	958	tags=84%, lis
REACTOME_REGULATION_OF_MRNA_STABILITY_BY_PROTEINS_THAT_BIND_AU_RICH_ELEMENTS	28	0.413	1.947	0	0.11575853	0.994	958	tags=75%, lis
REACTOME_REGULATION_OF_PTEN_STABILITY_AND_ACTIVITY	21	0.447	1.947	0.001851851	0.11339873	0.994	958	tags=81%, lis
GOCC_ENDOPEPTIDASE_COMPLEX	23	0.425	1.916	0.014814815	0.14068386	0.998	1005	tags=83%, lis
REACTOME_DISORDERS_OF_TRANSMEMBRANE_TRANSPORTERS	31	0.385	1.908	0.001798561	0.14629863	0.998	592	tags=52%, lis
GOCC_FICOLIN_1_RICH_GRANULE	50	0.335	1.897	0.005405405	0.15484615	0.998	558	tags=42%, lis
GOBP_REGULATION_OF_CELLULAR_AMINE_METABOLIC_PROCESS	27	0.397	1.888	0.003780718	0.16133873	0.998	667	tags=59%, lis
REACTOME_DEGRADATION_OF_GLI1_BY_THE_PROTEASOME	23	0.416	1.878	0.007575758	0.16962339	0.999	990	tags=78%, lis
REACTOME_DEGRADATION_OF_AXIN	20	0.441	1.868	0.005545286	0.1794344	0.999	958	tags=80%, lis
GOBP_REGULATION_OF_IMMUNE_SYSTEM_PROCESS	284	0.217	1.867	0	0.17780834	0.999	536	tags=32%, lis
REACTOME_REGULATION_OF_RUNX2_EXPRESSION_AND_ACTIVITY	26	0.405	1.866	0.003683241	0.17501758	0.999	671	tags=58%, lis
REACTOME_TNFR2_NON_CANONICAL_NF_KB_PATHWAY	27	0.402	1.865	0.007575758	0.17251003	0.999	958	tags=74%, lis
REACTOME_TCR_SIGNALING	32	0.372	1.861	0.010291595	0.1738612	0.999	592	tags=53%, lis
GOBP_ANTIGEN_PROCESSING_AND_PRESENTATION_OF_PEPTIDE_ANTIGEN	66	0.296	1.855	0.005639097	0.1772574	0.999	609	tags=42%, lis
GOBP_REGULATION_OF_MRNA_CATABOLIC_PROCESS	64	0.304	1.855	0.003610108	0.17477353	0.999	620	tags=47%, lis
REACTOME_DISEASES_OF_METABOLISM	18	0.453	1.853	0.005660377	0.17418058	0.999	434	tags=50%, lis
REACTOME_CELLULAR_RESPONSE_TO_CHEMICAL_STRESS	57	0.305	1.850	0.003527336	0.17459275	0.999	788	tags=53%, lis
GOBP_REGULATION_OF_CELLULAR_AMINO_ACID_METABOLIC_PROCESS	23	0.398	1.845	0.007339449	0.17734355	0.999	667	tags=61%, lis
HP_DEVELOPMENTAL_REGRESSION	52	0.313	1.845	0.010507881	0.17463614	1	980	tags=65%, lis
GOBP_ANTIGEN_RECEPTOR_MEDIATED_SIGNALING_PATHWAY	66	0.294	1.841	0.003546099	0.17652695	1	592	tags=44%, lis
GOBP_INTERLEUKIN_1_MEDIATED_SIGNALING_PATHWAY	33	0.365	1.836	0.003838771	0.1795414	1	667	tags=55%, lis
HP_ABNORMAL_ENZYME_COENZYME_ACTIVITY	74	0.283	1.825	0.003649635	0.19064201	1	505	tags=39%, lis
GOBP_REGULATION_OF_VIRAL_GENOME_REPLICATION	15	0.481	1.825	0.016304348	0.1879645	1	319	tags=47%, lis
REACTOME_MITOTIC_G1_PHASE_AND_G1_S_TRANSITION	37	0.347	1.817	0.005504587	0.19494753	1	788	tags=59%, lis
GOBP_ACTIVATION_OF_INNATE_IMMUNE_RESPONSE	42	0.336	1.815	0.013333334	0.19486405	1	958	tags=67%, lis
REACTOME_NEGATIVE_REGULATION_OF_NOTCH4_SIGNALING	21	0.409	1.813	0.009140768	0.19425203	1	958	tags=76%, lis
REACTOME_CYCLIN_A_CDK2_ASSOCIATED_EVENTS_AT_S_PHASE_ENTRY	25	0.401	1.811	0.015817223	0.19373403	1	667	tags=60%, lis
GOBP_POSITIVE_REGULATION_OF_IMMUNE_SYSTEM_PROCESS	187	0.232	1.809	0.001694915	0.1940646	1	645	tags=38%, lis
REACTOME_REGULATION_OF_RUNX3_EXPRESSION_AND_ACTIVITY	22	0.406	1.807	0.010810811	0.19387971	1	958	tags=73%, lis
GOBP_IMMUNE_EFFECTOR_PROCESS	275	0.210	1.806	0.001642036	0.19177872	1	537	tags=31%, lis
GOBP_AMINE_METABOLIC_PROCESS	33	0.363	1.798	0.003795066	0.19926882	1	667	tags=55%, lis
REACTOME_METABOLISM_OF_POLYAMINES	23	0.406	1.795	0.010830325	0.20108515	1	958	tags=78%, lis
HP_THORACIC_HYPOPLASIA	21	0.410	1.793	0.015873017	0.20037927	1	803	tags=67%, lis
GOBP_ANTIGEN_PROCESSING_AND_PRESENTATION	73	0.282	1.790	0.003539823	0.20124261	1	609	tags=41%, lis
GOBP_RESPONSE_TO_TUMOR_NECROSIS_FACTOR	67	0.288	1.787	0	0.20299084	1	592	tags=42%, lis
GOCC_PEPTIDASE_COMPLEX	26	0.378	1.786	0.012844036	0.20095181	1	1005	tags=77%, lis
REACTOME_G2_M_CHECKPOINTS	32	0.355	1.784	0.016949153	0.2007303	1	788	tags=66%, lis
HP_IRRITABILITY	39	0.331	1.780	0.011320755	0.20367819	1	991	tags=69%, lis
REACTOME_REGULATION_OF_EXPRESSION_OF_SLITS_AND_ROBOS	25	0.388	1.775	0.019305019	0.20918947	1	958	tags=72%, lis

GOBP_DEFENSE_RESPONSE_TO_OTHER_ORGANISM	216	0.218	1.774	0.001680672	0.20745344	1	617	tags=36%, lis
GOBP_CELLULAR_KETONE_METABOLIC_PROCESS	38	0.329	1.767	0.007142857	0.21467969	1	667	tags=55%, lis
REACTOME_REGULATION_OF_HMOX1_EXPRESSION_AND_ACTIVITY	25	0.385	1.764	0.013358778	0.2165069	1	667	tags=56%, lis
GOBP_IMMUNE_RESPONSE_REGULATING_SIGNALING_PATHWAY	97	0.255	1.763	0	0.21529014	1	592	tags=39%, lis
GOBP_ANTIGEN_PROCESSING_AND_PRESENTATION_OF_EXOGENOUS_PEPTIDE_ANTIGEN_VIA_MH	37	0.332	1.762	0.011049724	0.21440123	1	609	tags=49%, lis
REACTOME_THE_ROLE_OF_GTSE1_IN_G2_M_PROGRESSION_AFTER_G2_CHECKPOINT	23	0.393	1.757	0.010889292	0.21825314	1	958	tags=74%, lis
GOBP_DEFENSE_RESPONSE	289	0.204	1.755	0	0.21817571	1	562	tags=33%, lis
GOBP_ANTIGEN_PROCESSING_AND_PRESENTATION_OF_PEPTIDE_ANTIGEN_VIA_MHC_CLASS_I	43	0.316	1.751	0.01119403	0.22153133	1	609	tags=47%, lis
GOBP_REGULATION_OF_DEFENSE_RESPONSE	145	0.231	1.748	0.001792114	0.22213802	1	562	tags=35%, lis
GOBP_INNATE_IMMUNE_RESPONSE	182	0.220	1.747	0.001686340	0.22077431	1	627	tags=36%, lis
REACTOME_G1_S_DNA_DAMAGE_CHECKPOINTS	23	0.389	1.741	0.016245488	0.22770879	1	958	tags=74%, lis
REACTOME_TRANSCRIPTIONAL_REGULATION_BY_RUNX2	33	0.349	1.734	0.013207547	0.23520969	1	671	tags=55%, lis
REACTOME_SWITCHING_OF_ORIGINS_TO_A_POST_REPLICATIVE_STATE	27	0.367	1.725	0.019880716	0.24667166	1	958	tags=70%, lis
GOBP_NEGATIVE_REGULATION_OF_INNATE_IMMUNE_RESPONSE	16	0.435	1.719	0.011029412	0.25217843	1	729	tags=63%, lis
GOBP_REGULATION_OF_RESPONSE_TO_EXTERNAL_STIMULUS	189	0.212	1.712	0.005093378	0.2611942	1	562	tags=33%, lis
REACTOME_NEUTROPHIL_DEGRANULATION	125	0.236	1.707	0.005328597	0.2676577	1	486	tags=32%, lis
GOBP_POSITIVE_REGULATION_OF_RESPONSE_TO_EXTERNAL_STIMULUS	109	0.245	1.706	0.012152778	0.26623845	1	610	tags=38%, lis
GOBP_REGULATION_OF_CELLULAR_KETONE_METABOLIC_PROCESS	33	0.344	1.704	0.011257036	0.26656407	1	667	tags=58%, lis
GOBP_DETOXIFICATION	23	0.380	1.704	0.008960574	0.26465192	1	371	tags=39%, lis
GOCC_TERTIARY_GRANULE	40	0.321	1.703	0.011516315	0.2628147	1	558	tags=43%, lis
GOCC_MITOTIC_SPINDLE	18	0.411	1.702	0.032319393	0.2615026	1	211	tags=33%, lis
REACTOME_INTERLEUKIN_1_FAMILY_SIGNALING	36	0.327	1.701	0.017985612	0.26033965	1	667	tags=53%, lis
GOBP_CYTOKINE_MEDIATED_SIGNALING_PATHWAY	161	0.222	1.700	0.004991680	0.26016006	1	562	tags=34%, lis
REACTOME_REGULATION_OF_RAS_BY_GAPS	24	0.379	1.695	0.039033458	0.26550862	1	667	tags=58%, lis
REACTOME_METABOLISM_OF_AMINO_ACIDS_AND_DERIVATIVES	44	0.303	1.693	0.017857144	0.26592225	1	974	tags=68%, lis
GOBP_RESPONSE_TO_BIOTIC_STIMULUS	297	0.195	1.690	0	0.26753867	1	562	tags=32%, lis
REACTOME_FCERI_MEDIATED_NF_KB_ACTIVATION	30	0.349	1.690	0.026217228	0.26543942	1	958	tags=70%, lis
KEGG_CELL_ADHESION_MOLECULES_CAMS	15	0.445	1.689	0.02846975	0.26527864	1	500	tags=53%, lis
HP_FAILURE_TO_THRIVE	137	0.228	1.683	0.009009009	0.2714063	1	832	tags=49%, lis
REACTOME_STABILIZATION_OF_P53	22	0.380	1.678	0.032258064	0.2771837	1	958	tags=73%, lis
GOMF_SH3_DOMAIN_BINDING	15	0.440	1.677	0.029126214	0.27776647	1	867	tags=73%, lis
REACTOME_SIGNALING_BY_ROBO_RECEPTORS	32	0.334	1.673	0.020257827	0.28082734	1	958	tags=66%, lis
GOBP_RESPONSE_TO_CYTOKINE	235	0.204	1.673	0.001669449	0.27846414	1	503	tags=29%, lis
GOCC_FICOLIN_1_RICH_GRANULE_LUMEN	30	0.343	1.667	0.014869888	0.28763446	1	667	tags=47%, lis
HP_METABOLIC_ACIDOSIS	17	0.416	1.662	0.03047619	0.2930562	1	577	tags=59%, lis
REACTOME_RRNA_PROCESSING	15	0.438	1.661	0.034734916	0.292102	1	1076	tags=87%, lis
REACTOME_HEDGEHOG_OFF_STATE	28	0.337	1.656	0.018214935	0.2987266	1	990	tags=68%, lis
GOBP_ANAPHASE_PROMOTING_COMPLEX_DEPENDENT_CATABOLIC_PROCESS	24	0.370	1.653	0.02919708	0.3008571	1	958	tags=71%, lis
GOBP_REGULATION_OF_RESPONSE_TO_CYTOKINE_STIMULUS	54	0.281	1.652	0.01908397	0.30024093	1	562	tags=39%, lis
GOBP_PROTEIN_HOMOOLIGOMERIZATION	22	0.372	1.651	0.026415095	0.2998792	1	561	tags=45%, lis
GOMF_NUCLEOTIDYLTRANSFERASE_ACTIVITY	16	0.417	1.651	0.034136545	0.29789007	1	591	tags=50%, lis
GOBP_ESTABLISHMENT_OF_TISSUE_POLARITY	23	0.369	1.644	0.026819924	0.30666018	1	958	tags=74%, lis
GOBP_RRNA_METABOLIC_PROCESS	27	0.347	1.642	0.031135531	0.30913365	1	1076	tags=74%, lis
GOBP_NEGATIVE_REGULATION_OF_IMMUNE_SYSTEM_PROCESS	80	0.248	1.636	0.020942409	0.31838334	1	499	tags=31%, lis
GOBP_INNATE_IMMUNE_RESPONSE_ACTIVATING_SIGNAL_TRANSDUCTION	35	0.314	1.633	0.023131672	0.32053244	1	958	tags=69%, lis
REACTOME_CYTOPROTECTION_BY_HMOX1	42	0.297	1.622	0.03142329	0.3400383	1	788	tags=52%, lis
HP_POOR_HEAD_CONTROL	29	0.339	1.622	0.03130435	0.33749664	1	646	tags=48%, lis
GOBP_MONOCARBOXYLIC_ACID_BIOSYNTHETIC_PROCESS	21	0.374	1.619	0.034068137	0.3417488	1	143	tags=29%, lis
HP_LACTIC_ACIDOSIS	24	0.354	1.616	0.026768642	0.343837	1	899	tags=63%, lis
GOBP_T_CELL_RECEPTOR_SIGNALING_PATHWAY	46	0.285	1.611	0.033568904	0.3516309	1	592	tags=43%, lis
HP_TREMOR	89	0.239	1.607	0.019264448	0.35708	1	495	tags=33%, lis
REACTOME_SIGNALING_BY_HEDGEHOG	37	0.304	1.602	0.038391225	0.3639971	1	788	tags=51%, lis
GOCC_SECRETORY_GRANULE_MEMBRANE	75	0.253	1.597	0.014084507	0.3720615	1	479	tags=35%, lis
REACTOME_CYTOSOLIC_SENSORS_OF_PATHOGEN_ASSOCIATED_DNA	17	0.399	1.592	0.04868914	0.37921003	1	697	tags=53%, lis
GOBP_REGULATION_OF_HEMATOPOIETIC_PROGENITOR_CELL_DIFFERENTIATION	33	0.321	1.592	0.030245747	0.37768897	1	1000	tags=70%, lis
REACTOME_PCP_CE_PATHWAY	24	0.352	1.589	0.04725898	0.3797514	1	958	tags=71%, lis
REACTOME_RUNX1_REGULATES_TRANSCRIPTION_OF_GENES_INVOLVED_IN_DIFFERENTIATION_OF	25	0.340	1.589	0.0455408	0.37750864	1	958	tags=72%, lis
GOBP_HEMATOPOIETIC_PROGENITOR_CELL_DIFFERENTIATION	40	0.289	1.579	0.036750484	0.3957924	1	667	tags=48%, lis
REACTOME_ANTIGEN_PROCESSING_CROSS_PRESENTATION	40	0.295	1.577	0.053113554	0.39868692	1	667	tags=48%, lis
KEGG_EPITHELIAL_CELL_SIGNALING_IN_HELICOBACTER_PYLORI_INFECTION	17	0.392	1.577	0.056497175	0.39640418	1	720	tags=59%, lis
REACTOME_HEDGEHOG_ON_STATE	29	0.321	1.575	0.04779412	0.39627022	1	667	tags=48%, lis
GOMF_PROTEIN_HOMODIMERIZATION_ACTIVITY	95	0.230	1.573	0.033391915	0.39952892	1	887	tags=53%, lis
HP_RESTING_TREMOR	15	0.406	1.570	0.0483559	0.40226942	1	970	tags=73%, lis
GOMF_ENDOPEPTIDASE_ACTIVITY	51	0.279	1.569	0.032490976	0.4007147	1	712	tags=53%, lis
GOBP_CELLULAR_RESPONSE_TO_TOXIC_SUBSTANCE	22	0.357	1.567	0.03954802	0.4047432	1	371	tags=36%, lis
REACTOME_HIV_INFECTION	57	0.264	1.563	0.03314917	0.41052634	1	592	tags=40%, lis
REACTOME_RESPIRATORY_ELECTRON_TRANSPORT_ATP_SYNTHESIS_BY_CHEMIOSMOTIC_COUPLIN	43	0.280	1.561	0.051526718	0.41275728	1	1257	tags=77%, lis
REACTOME_IMMUNOREGULATORY_INTERACTIONS_BETWEEN_A_LYMPHOID_AND_A_NON_LYMPH	20	0.364	1.560	0.058303885	0.4101357	1	500	tags=45%, lis
GOBP_VIRAL_LIFE_CYCLE	76	0.242	1.560	0.017094018	0.40795878	1	561	tags=37%, lis
GOBP_MIDBRAIN_DEVELOPMENT	15	0.410	1.558	0.05162524	0.4105648	1	691	tags=60%, lis
HP_INTERICTAL_EEG_ABNORMALITY	39	0.291	1.556	0.05263158	0.4128736	1	620	tags=46%, lis
GOBP_RESPONSE_TO_OXYGEN_LEVELS	88	0.233	1.555	0.03152364	0.41068205	1	958	tags=55%, lis
HP_OPTIC_NEUROPATHY	15	0.406	1.554	0.0655106	0.41105977	1	577	tags=47%, lis
GOCC_PROTON_TRANSPORTING_TWO_SECTOR_ATPASE_COMPLEX	16	0.398	1.553	0.055350553	0.41048712	1	753	tags=63%, lis
HP_HALLUCINATIONS	25	0.337	1.552	0.04743833	0.41000256	1	293	tags=32%, lis
REACTOME_CYTOKINE_SIGNALING_IN_IMMUNE_SYSTEM	151	0.203	1.552	0.017123288	0.407678	1	501	tags=31%, lis
HALLMARK_REACTIVE_OXYGEN_SPECIES_PATHWAY	22	0.362	1.551	0.052532833	0.40639812	1	567	tags=50%, lis
REACTOME_INNATE_IMMUNE_SYSTEM	248	0.186	1.549	0.014010508	0.40834576	1	562	tags=31%, lis
GOBP_ENTRY_INTO_HOST	34	0.294	1.547	0.033898305	0.410181	1	537	tags=41%, lis

GOBP_POSITIVE_REGULATION_OF_TYPE_I_INTERFERON_PRODUCTION	26	0.334	1.547	0.06130268	0.408986	1	617	tags=46%, lis
GOBP_RESPONSE_TO_TOXIC_SUBSTANCE	39	0.290	1.545	0.034883723	0.40965226	1	371	tags=33%, lis
GOCC_VACUOLAR_MEMBRANE	108	0.221	1.543	0.019927537	0.41165212	1	569	tags=35%, lis
HP_ACTION_TREMOR	26	0.337	1.542	0.058303885	0.41071913	1	487	tags=42%, lis
REACTOME_INTERLEUKIN_1_SIGNALING	34	0.297	1.542	0.04710145	0.40817338	1	667	tags=50%, lis
REACTOME_HOST_INTERACTIONS_OF_HIV_FACTORS	40	0.292	1.541	0.039772727	0.40973976	1	592	tags=43%, lis
GOCC_FICOLIN_1_RICH_GRANULE_MEMBRANE	20	0.360	1.536	0.05321101	0.41820508	1	308	tags=35%, lis
HP_ABNORMALITY_OF_THE_DIENCEPHALON	15	0.399	1.533	0.058601134	0.4221882	1	807	tags=67%, lis
REACTOME_CELLULAR_RESPONSE_TO_STARVATION	20	0.360	1.531	0.06130268	0.4228952	1	1086	tags=75%, lis
GOBP_RIBOSOME_BIOGENESIS	31	0.310	1.529	0.06032907	0.42605796	1	1076	tags=71%, lis
GOBP_MYELOID_LEUKOCYTE_ACTIVATION	158	0.199	1.528	0.017513135	0.42505097	1	486	tags=30%, lis
GOBP_NIK_NF_KAPPAB_SIGNALING	47	0.273	1.524	0.042031523	0.4314333	1	592	tags=45%, lis
HALLMARK_ALLOGRAFT_REJECTION	37	0.288	1.519	0.06398538	0.441324	1	534	tags=35%, lis
HP_ABNORMAL_MYOCARDIUM_MORPHOLOGY	64	0.247	1.518	0.044483986	0.4412159	1	991	tags=56%, lis
REACTOME_SEPARATION_OF_SISTER_CHROMATIDS	38	0.287	1.518	0.044	0.4389897	1	592	tags=42%, lis
GOBP_OXIDATIVE_PHOSPHORYLATION	46	0.269	1.512	0.040145986	0.452125	1	1257	tags=76%, lis
HP_LETHARGY	27	0.319	1.511	0.05313093	0.45013946	1	714	tags=48%, lis
GOBP_POSTREPLICATION_REPAIR	16	0.382	1.509	0.056776557	0.452863	1	597	tags=50%, lis
HP_ABNORMAL_ENCHONDRAL_OSSIFICATION	18	0.372	1.508	0.07777778	0.45263195	1	803	tags=61%, lis
HP_ABNORMALITY_OF_ACID_BASE_HOMEOSTASIS	52	0.258	1.506	0.050847456	0.45624807	1	899	tags=54%, lis
HP_APNEA	47	0.269	1.505	0.042201836	0.45475265	1	933	tags=57%, lis
GOBP_MYELOID_LEUKOCYTE_MEDIATED_IMMUNITY	143	0.196	1.505	0.027777778	0.45234805	1	486	tags=29%, lis
GOBP_PROTEIN_LOCALIZATION_TO_VACUOLE	20	0.353	1.504	0.074285716	0.45434937	1	683	tags=55%, lis
GOCC_MITOCHONDRIAL_MATRIX	70	0.237	1.503	0.035906643	0.45249432	1	1043	tags=64%, lis
GOBP_REGULATION_OF_TOLL_LIKE_RECEPTOR_SIGNALING_PATHWAY	23	0.339	1.503	0.09140768	0.451351	1	742	tags=57%, lis
REACTOME_INFLUENZA_INFECTION	16	0.379	1.499	0.051376145	0.4566701	1	411	tags=44%, lis
HP_ABNORMALITY_OF_THE_METAPHYSIS	32	0.304	1.498	0.07358491	0.4563444	1	300	tags=31%, lis
GOBP_MORPHOGENESIS_OF_A_POLARIZED_EPITHELIUM	26	0.319	1.496	0.07514451	0.45908043	1	958	tags=69%, lis
GOMF_ANTIOXIDANT_ACTIVITY	18	0.365	1.496	0.06727273	0.45681006	1	371	tags=39%, lis
KEGG_OXIDATIVE_PHOSPHORYLATION	45	0.272	1.494	0.05035971	0.45890838	1	1088	tags=64%, lis
REACTOME_APOPTOSIS	47	0.267	1.493	0.058270678	0.45961356	1	958	tags=64%, lis
REACTOME_MITOTIC_METAPHASE_AND_ANAPHASE	47	0.266	1.491	0.045045044	0.4615305	1	611	tags=40%, lis
HP_ABNORMALITY_OF_THE_OVARY	28	0.307	1.490	0.075187966	0.46213222	1	864	tags=57%, lis
HP_ABNORMAL_PATTERN_OF_RESPIRATION	56	0.254	1.487	0.05956679	0.46754718	1	317	tags=25%, lis
GOBP_INTRINSIC_APOPTOTIC_SIGNALING_PATHWAY_BY_P53_CLASS_MEDIATOR	17	0.378	1.483	0.08550186	0.4738655	1	189	tags=24%, lis
HP_METAPHYSEAL_WIDENING	16	0.379	1.481	0.057761732	0.47697255	1	863	tags=63%, lis
GOBP_SCF_DEPENDENT_PROTEASOMAL_UBIQUITIN_DEPENDENT_PROTEIN_CATABOLIC_PROCESS	28	0.312	1.478	0.055456173	0.48194614	1	667	tags=50%, lis
REACTOME_APC_C_CDH1_MEDIATED_DEGRADATION_OF_CDC20_AND_OTHER_APC_C_CDH1_TAR	24	0.324	1.477	0.06591337	0.48197332	1	958	tags=67%, lis
GOBP_ESTABLISHMENT_OF_PROTEIN_LOCALIZATION_TO_VACUOLE	16	0.367	1.477	0.069767445	0.48008573	1	804	tags=63%, lis
HP_APLASIA_HYPOPLASIA_OF_THE_RIBS	15	0.384	1.477	0.07421875	0.47793624	1	803	tags=60%, lis
REACTOME_MAPK6_MAPK4_SIGNALING	30	0.303	1.475	0.06754221	0.48046273	1	958	tags=67%, lis
HP_STATUS_EPILEPTICUS	29	0.316	1.474	0.08015267	0.4803042	1	714	tags=52%, lis
GOBP_REGULATION_OF_STEM_CELL_DIFFERENTIATION	36	0.277	1.472	0.048327137	0.48361477	1	603	tags=44%, lis
HP_PALLOR	25	0.313	1.471	0.06884058	0.48221534	1	495	tags=40%, lis
REACTOME_UB_SPECIFIC_PROCESSING_PROTEASES	52	0.251	1.471	0.05283019	0.4800403	1	667	tags=46%, lis
GOBP_NEGATIVE_REGULATION_OF_RESPONSE_TO_ENDOPLASMIC_RETICULUM_STRESS	17	0.368	1.467	0.08394161	0.48835263	1	431	tags=41%, lis
HP_ABNORMALITY_OF_PULMONARY_CIRCULATION	21	0.345	1.466	0.07692308	0.48739448	1	314	tags=29%, lis
GOBP_DEFENSE_RESPONSE_TO_BACTERIUM	31	0.296	1.464	0.07879925	0.49069178	1	627	tags=48%, lis
GOCC_VESICLE_MEMBRANE	161	0.188	1.464	0.044802867	0.4895185	1	479	tags=29%, lis
GOBP_VIRAL_GENOME_REPLICATION	29	0.306	1.464	0.07485604	0.4871722	1	340	tags=31%, lis
HP_HYPSARRHYTHMIA	31	0.294	1.464	0.06751825	0.48511592	1	524	tags=42%, lis
GOBP_REGULATION_OF_DNA_TEMPLATED_TRANSCRIPTION_IN_RESPONSE_TO_STRESS	38	0.282	1.461	0.06457564	0.4908057	1	667	tags=47%, lis
GOBP_TYPE_I_INTERFERON_PRODUCTION	41	0.270	1.456	0.056074765	0.5007975	1	697	tags=44%, lis
KEGG_LEUKOCYTE_TRANSENDOTHELIAL_MIGRATION	22	0.342	1.456	0.09601449	0.4994083	1	635	tags=50%, lis
REACTOME_RESPIRATORY_ELECTRON_TRANSPORT	37	0.277	1.454	0.08775137	0.50060195	1	1419	tags=86%, lis
GOBP_CELL_ACTIVATION_INVOLVED_IN_IMMUNE_RESPONSE	180	0.185	1.454	0.028268551	0.49915668	1	506	tags=29%, lis
GOBP_RETROGRADE_VESICLE_MEDIATED_TRANSPORT_GOLGI_TO_ENDOPLASMIC_RETICULUM	22	0.331	1.453	0.08302583	0.49899414	1	297	tags=32%, lis
GOCC_SECRETORY_GRANULE	166	0.185	1.452	0.033101045	0.49778593	1	486	tags=29%, lis
GOBP_PROTEIN_MODIFICATION_BY_SMALL_PROTEIN_REMOVAL	83	0.220	1.450	0.07007576	0.5017432	1	683	tags=43%, lis
HP_ABNORMAL_NEURAL_TUBE_MORPHOLOGY	25	0.318	1.450	0.06934307	0.50015193	1	224	tags=28%, lis
GOCC_MICROBODY	22	0.330	1.446	0.09330986	0.5084856	1	917	tags=68%, lis
HP_PLATYSPONDYLY	16	0.361	1.444	0.0917603	0.51016426	1	89	tags=25%, lis
HP_ABNORMALITY_OF_THE_BASAL_GANGLIA	48	0.259	1.443	0.07760141	0.5097586	1	999	tags=60%, lis
GOBP_FATTY_ACID_BIOSYNTHETIC_PROCESS	19	0.341	1.443	0.08639706	0.5087193	1	143	tags=26%, lis
GOBP_POSITIVE_REGULATION_OF_CANONICAL_WNT_SIGNALING_PATHWAY	35	0.280	1.441	0.089090906	0.5096795	1	958	tags=63%, lis
HP_RIGIDITY	38	0.270	1.441	0.091778204	0.5086655	1	461	tags=34%, lis
REACTOME_APC_C_MEDIATED_DEGRADATION_OF_CELL_CYCLE_PROTEINS	24	0.324	1.441	0.08971962	0.5075294	1	958	tags=67%, lis
GOBP_NEGATIVE_REGULATION_OF_CELL_CYCLE_G2_M_PHASE_TRANSITION	27	0.302	1.437	0.07992565	0.51314145	1	667	tags=52%, lis
HP_BULBOUS_NOSE	24	0.330	1.435	0.08872458	0.51843446	1	675	tags=50%, lis
HP_MENTAL_DETERIORATION	66	0.229	1.434	0.083769634	0.5163206	1	461	tags=30%, lis
HP_LEUKODYSTROPHY	29	0.298	1.433	0.083636366	0.51686877	1	1281	tags=83%, lis
REACTOME_THE_CITRIC_ACID_TCA_CYCLE_AND_RESPIRATORY_ELECTRON_TRANSPORT	48	0.251	1.432	0.07400379	0.5172075	1	1257	tags=75%, lis
GOBP_FC_RECEPTOR_SIGNALING_PATHWAY	53	0.244	1.432	0.07885305	0.5166283	1	667	tags=43%, lis
GOBP_HEMATOPOIETIC_STEM_CELL_DIFFERENTIATION	31	0.289	1.432	0.071969695	0.51467896	1	958	tags=65%, lis
GOCC_CLATHRIN_COATED_VESICLE_MEMBRANE	29	0.292	1.432	0.088495575	0.5127579	1	569	tags=45%, lis
REACTOME_CELLULAR_RESPONSE_TO_HYPOXIA	24	0.317	1.429	0.07650273	0.51694375	1	958	tags=67%, lis
KEGG_ANTIGEN_PROCESSING_AND_PRESENTATION	19	0.347	1.426	0.099047616	0.5231481	1	340	tags=37%, lis
HP_PNEUMONIA	42	0.261	1.426	0.088607594	0.5209952	1	476	tags=33%, lis
GOMF_GUANYL_NUCLEOTIDE_BINDING	55	0.238	1.424	0.09157509	0.52181894	1	905	tags=51%, lis

REACTOME_MITOTIC_G2_G2_M_PHASES	37	0.269	1.419	0.08495575	0.53319824	1	682	tags=46%, lis
HP_PRE_CAPILLARY_PULMONARY_HYPERTENSION	18	0.347	1.419	0.09864604	0.5313536	1	314	tags=28%, lis
GOBP_PROTEIN_DEPOLYMERIZATION	17	0.357	1.417	0.08364312	0.5330735	1	504	tags=41%, lis
HP_COGNITIVE_IMPAIRMENT	100	0.199	1.417	0.044198897	0.5310533	1	864	tags=48%, lis
REACTOME_SIGNALING_BY_NOTCH4	27	0.299	1.414	0.09461967	0.53667265	1	667	tags=48%, lis
REACTOME_DOWNSTREAM_SIGNALING_EVENTS_OF_B_CELL_RECEPTOR_BCR	28	0.294	1.414	0.08679928	0.53450453	1	667	tags=50%, lis
GOBP_LEUKOCYTE_MEDIATED_IMMUNITY	182	0.180	1.413	0.034722224	0.5359105	1	558	tags=31%, lis
REACTOME_CELL_CYCLE_CHECKPOINTS	50	0.247	1.412	0.090405904	0.5356474	1	788	tags=52%, lis
GOCC_RUFFLE_MEMBRANE	21	0.325	1.410	0.12387792	0.5376069	1	473	tags=38%, lis
REACTOME_PROGRAMMED_CELL_DEATH	56	0.239	1.408	0.08448276	0.5433367	1	926	tags=59%, lis
GOMF_MOLECULAR_TRANSDUCER_ACTIVITY	88	0.213	1.406	0.06797853	0.5452634	1	586	tags=34%, lis
HP_DENTAL_MALOCCLUSION	21	0.321	1.405	0.11363637	0.54581815	1	138	tags=24%, lis
HP_GONOSOMAL_INHERITANCE	37	0.266	1.402	0.1059246	0.5513087	1	382	tags=30%, lis
REACTOME_REGULATION_OF_TNFR1_SIGNALING	18	0.337	1.401	0.110486895	0.55164975	1	901	tags=61%, lis
GOBP_NEGATIVE_REGULATION_OF_SIGNALING	225	0.170	1.398	0.037162162	0.5576695	1	496	tags=28%, lis
GOBP_REGULATION_OF_ANIMAL_ORGAN_MORPHOGENESIS	25	0.307	1.397	0.12264151	0.55727524	1	667	tags=52%, lis
GOBP_REGULATION_OF_ANTIGEN_RECEPTOR_MEDIATED_SIGNALING_PATHWAY	17	0.352	1.396	0.0953271	0.5589691	1	824	tags=59%, lis
GOBP_REGULATION_OF_HEMATOPOIETIC_STEM_CELL_DIFFERENTIATION	28	0.289	1.395	0.09796673	0.5585934	1	958	tags=64%, lis
GOMF_PROTEIN_FOLDING_CHAPERONE	15	0.368	1.395	0.12316176	0.55639905	1	637	tags=53%, lis
REACTOME_S_PHASE	42	0.255	1.393	0.093639575	0.55964583	1	788	tags=50%, lis
GOBP_ATP_SYNTHESIS_COUPLED_ELECTRON_TRANSPORT	39	0.257	1.389	0.110320285	0.5691104	1	1257	tags=74%, lis
GOBP_POSTTRANSCRIPTIONAL_REGULATION_OF_GENE_EXPRESSION	140	0.188	1.388	0.05940594	0.5709385	1	924	tags=51%, lis
REACTOME_DNA_REPLICATION	32	0.278	1.387	0.10873441	0.5701354	1	958	tags=63%, lis
GOBP_B_CELL_RECEPTOR_SIGNALING_PATHWAY	24	0.308	1.387	0.11190053	0.5679586	1	535	tags=42%, lis
GOBP_CELLULAR_OXIDANT_DETOXIFICATION	20	0.333	1.386	0.113553114	0.5693942	1	371	tags=35%, lis
GOBP_CELL_CYCLE_G2_M_PHASE_TRANSITION	49	0.241	1.384	0.09671533	0.5722027	1	682	tags=45%, lis
GOBP_REGULATION_OF_TRANSCRIPTION_FROM_RNA_POLYMERASE_II_PROMOTER_IN_RESPONSE	27	0.300	1.383	0.12361623	0.570997	1	667	tags=48%, lis
GOBP_PROTEIN_LOCALIZATION_TO_NUCLEUS	56	0.235	1.382	0.09689214	0.5719221	1	450	tags=30%, lis
GOCC_RESPIRATORY_CHAIN_COMPLEX	30	0.283	1.382	0.13076924	0.5701162	1	1257	tags=77%, lis
REACTOME_ADAPTIVE_IMMUNE_SYSTEM	181	0.176	1.382	0.05175292	0.56876844	1	503	tags=28%, lis
GOBP_NEGATIVE_REGULATION_OF_RESPONSE_TO_BIOTIC_STIMULUS	23	0.316	1.379	0.10412926	0.5737459	1	963	tags=61%, lis
HP_ABNORMAL_BRONCHUS_MORPHOLOGY	20	0.326	1.376	0.11376147	0.5800133	1	300	tags=30%, lis
HP_DECREASED_BODY_WEIGHT	210	0.169	1.376	0.043333333	0.5791397	1	864	tags=45%, lis
HP_RESPIRATORY_INSUFFICIENCY	76	0.210	1.375	0.0953271	0.57942665	1	807	tags=47%, lis
GOBP_MOVEMENT_IN_HOST_ENVIRONMENT	40	0.257	1.373	0.10486891	0.5837588	1	561	tags=40%, lis
REACTOME_INTERLEUKIN_12_FAMILY_SIGNALING	18	0.329	1.369	0.12927757	0.59130263	1	130	tags=22%, lis
HP_HYDROCEPHALUS	43	0.255	1.369	0.12546125	0.5899743	1	314	tags=28%, lis
HP_HYPERTROPHIC_CARDIOMYOPATHY	38	0.257	1.369	0.119544595	0.58819914	1	980	tags=58%, lis
HP_ABNORMAL_ACTIVITY_OF_MITOCHONDRIAL_RESPIRATORY_CHAIN	22	0.315	1.367	0.10375671	0.590046	1	1419	tags=91%, lis
HP_LYMPHOPENIA	26	0.294	1.365	0.10810811	0.59454316	1	489	tags=38%, lis
GOCC_ENDOPLASMIC_RETICULUM_GOLGI_INTERMEDIATE_COMPARTMENT	32	0.268	1.365	0.12382739	0.5929676	1	516	tags=38%, lis
HP_BRADYKINESIA	23	0.308	1.363	0.121673	0.5960883	1	1074	tags=70%, lis
GOBP_NEGATIVE_REGULATION_OF_RESPONSE_TO_STIMULUS	263	0.163	1.360	0.051903114	0.60110974	1	496	tags=27%, lis
BIOCARTA_MAPK_PATHWAY	23	0.309	1.360	0.11342155	0.59976256	1	450	tags=39%, lis
GOBP_RESPIRATORY_ELECTRON_TRANSPORT_CHAIN	43	0.249	1.359	0.096958175	0.6012068	1	1257	tags=74%, lis
GOCC_ENDOPLASMIC_RETICULUM_GOLGI_INTERMEDIATE_COMPARTMENT_MEMBRANE	21	0.312	1.358	0.13218391	0.5992124	1	250	tags=29%, lis
GOMF_PEPTIDASE_ACTIVITY	77	0.203	1.357	0.10469314	0.6023647	1	712	tags=44%, lis
HP_ABNORMAL_BONE_STRUCTURE	79	0.207	1.356	0.09479554	0.6028146	1	300	tags=22%, lis
GOBP_RESPONSE_TO_OXIDATIVE_STRESS	94	0.198	1.354	0.07491289	0.604115	1	854	tags=49%, lis
HP_ABNORMALITY_OF_BRAINSTEM_MORPHOLOGY	43	0.245	1.354	0.11545293	0.60415334	1	612	tags=40%, lis
GOBP_ACTIN_POLYMERIZATION_OR_DEPOLYMERIZATION	39	0.252	1.353	0.1163227	0.60383487	1	504	tags=33%, lis
GOBP_BIOLOGICAL_PROCESS_INVOLVED_IN_INTERACTION_WITH_HOST	51	0.232	1.348	0.097928435	0.61632454	1	561	tags=37%, lis
GOBP_CELLULAR_RESPIRATION	58	0.223	1.347	0.111310594	0.61692894	1	899	tags=50%, lis
GOCC_SPINDLE_POLE	22	0.302	1.346	0.12713473	0.616141	1	128	tags=23%, lis
GOCC_INNER_MITOCHONDRIAL_MEMBRANE_PROTEIN_COMPLEX	44	0.250	1.346	0.12932605	0.61446387	1	1270	tags=75%, lis
REACTOME_MHC_CLASS_II_ANTIGEN_PRESENTATION	25	0.294	1.346	0.12987013	0.61324954	1	248	tags=24%, lis
KEGG_PARKINSONS_DISEASE	48	0.234	1.344	0.11908932	0.6156582	1	771	tags=44%, lis
HP_REDUCED_CONSCIOUSNESS_CONFUSION	47	0.235	1.339	0.115523465	0.62877065	1	718	tags=43%, lis
GOBP_TRANSCRIPTION_INITIATION_FROM_RNA_POLYMERASE_II_PROMOTER	29	0.277	1.337	0.122486286	0.6314422	1	671	tags=45%, lis
GOCC_AZUROPHIL_GRANULE_MEMBRANE	21	0.318	1.337	0.1311787	0.62973475	1	442	tags=38%, lis
HP_DEMENTIA	43	0.243	1.336	0.1376673	0.629776	1	461	tags=33%, lis
GOBP_CELLULAR_AMINO_ACID_METABOLIC_PROCESS	51	0.230	1.336	0.12007169	0.62838876	1	974	tags=61%, lis
GOCC_EARLY_ENDOSOME_MEMBRANE	25	0.293	1.336	0.1438849	0.62669104	1	229	tags=24%, lis
HP_JOINT_LAXITY	26	0.288	1.335	0.13508442	0.6278706	1	523	tags=38%, lis
GOCC_INTRINSIC_COMPONENT_OF_PLASMA_MEMBRANE	131	0.183	1.334	0.09632224	0.62653536	1	569	tags=33%, lis
GOBP_RESPONSE_TO_BACTERIUM	110	0.189	1.334	0.10076046	0.62480384	1	562	tags=34%, lis
GOBP_CYTOKINESIS	25	0.285	1.332	0.15229358	0.62819576	1	1024	tags=68%, lis
REACTOME_MRNA_SPLICING_MINOR_PATHWAY	15	0.356	1.331	0.16481481	0.62938213	1	605	tags=47%, lis
GOBP_NEUTROPHIL_MIGRATION	19	0.316	1.330	0.14869888	0.62967604	1	617	tags=47%, lis
REACTOME_DETOXIFICATION_OF_REACTIVE_OXYGEN_SPECIES	15	0.351	1.329	0.15913557	0.6323124	1	422	tags=40%, lis
GOBP_LEUKOCYTE_MEDIATED_CYTOTOXICITY	16	0.344	1.328	0.16699801	0.63164073	1	1084	tags=75%, lis
GOBP_POSITIVE_REGULATION_OF_CYTOKINE_PRODUCTION	90	0.195	1.326	0.13089006	0.63569343	1	568	tags=32%, lis
HP_DISINHIBITION	39	0.254	1.326	0.13148148	0.6356688	1	217	tags=23%, lis
GOBP_REGULATION_OF_TUMOR_NECROSIS_FACTOR_MEDIATED_SIGNALING_PATHWAY	21	0.299	1.325	0.1402214	0.63532084	1	528	tags=43%, lis
GOBP_CELLULAR_PROTEIN_COMPLEX_DISASSEMBLY	32	0.261	1.322	0.13812155	0.6417918	1	527	tags=38%, lis
GOBP_EXOCYTOSIS	195	0.164	1.321	0.08050089	0.64205587	1	561	tags=31%, lis
GOBP_POSITIVE_REGULATION_OF_AUTOPHAGY	21	0.304	1.320	0.140255	0.6430831	1	619	tags=48%, lis
REACTOME_SIGNALING_BY_NOTCH	50	0.234	1.320	0.14411248	0.64169854	1	489	tags=34%, lis
GOBP_POSITIVE_REGULATION_OF_NF_KAPPAB_TRANSCRIPTION_FACTOR_ACTIVITY	36	0.249	1.319	0.12903225	0.64294076	1	617	tags=42%, lis

HP_MYOCLONUS	54	0.229	1.318	0.14074074	0.6431107	1	714	tags=43%, lis
GOCC_SECRETORY_VESICLE	184	0.166	1.318	0.09306261	0.6415179	1	638	tags=34%, lis
GOMF_PEPTIDE_BINDING	45	0.235	1.317	0.12181818	0.6425658	1	450	tags=33%, lis
GOMF_RIBONUCLEOPROTEIN_COMPLEX_BINDING	33	0.261	1.314	0.14234875	0.6478541	1	540	tags=36%, lis
GOBP_PROTEIN_CONTAINING_COMPLEX_DISASSEMBLY	57	0.218	1.312	0.12891985	0.6508088	1	562	tags=35%, lis
HP_VISUAL_LOSS	18	0.320	1.312	0.15839694	0.6498225	1	293	tags=33%, lis
HP_ABNORMAL_SHAPE_OF_THE_FRONTAL_REGION	47	0.231	1.312	0.14689265	0.6487947	1	89	tags=17%, lis
HP_FULL_CHEEKS	16	0.331	1.311	0.15264188	0.64763695	1	224	tags=25%, lis
GOBP_CELL_DIVISION	97	0.192	1.309	0.1097561	0.65113634	1	835	tags=45%, lis
GOCC_NUCLEAR_PERIPHERY	27	0.277	1.307	0.15300547	0.6552219	1	197	tags=22%, lis
GOBP_MYELOID_LEUKOCYTE_MIGRATION	34	0.254	1.307	0.15270019	0.65398896	1	245	tags=24%, lis
HP_ABNORMALITY_OF_CIRCULATING_ENZYME_LEVEL	35	0.260	1.306	0.15026833	0.65456355	1	879	tags=60%, lis
GOBP_MITOTIC_NUCLEAR_DIVISION	41	0.238	1.305	0.15047619	0.65721875	1	411	tags=29%, lis
HP_ABNORMALITY_OF_THE_CEREBROSPINAL_FLUID	76	0.201	1.304	0.13644524	0.6574795	1	902	tags=50%, lis
REACTOME_TNF_SIGNALING	19	0.311	1.303	0.17408124	0.6575721	1	683	tags=47%, lis
REACTOME_UCH_PROTEINASES	29	0.268	1.301	0.15869981	0.66234785	1	667	tags=48%, lis
GOBP_REGULATION_OF_MRNA_METABOLIC_PROCESS	91	0.192	1.300	0.12908778	0.66152495	1	620	tags=35%, lis
HP_SMALL_FACE	27	0.271	1.299	0.1534091	0.6632779	1	807	tags=56%, lis
GOBP_I_KAPPAB_KINASE_NF_KAPPAB_SIGNALING	70	0.204	1.299	0.13661203	0.6621848	1	581	tags=34%, lis
HP_ABNORMAL_PHARYNX_MORPHOLOGY	28	0.275	1.299	0.16078432	0.66041017	1	476	tags=32%, lis
GOCC_RESPIRASOME	34	0.261	1.298	0.1575985	0.6598202	1	1419	tags=85%, lis
HP_HYPOREFLEXIA	55	0.219	1.297	0.14206642	0.6626652	1	820	tags=49%, lis
GOBP_CD4_POSITIVE_ALPHA_BETA_T_CELL_ACTIVATION	21	0.303	1.291	0.15732369	0.67573303	1	293	tags=29%, lis
GOMF_UBIQUITIN_BINDING	22	0.289	1.288	0.18023255	0.68263894	1	704	tags=50%, lis
HP_MEMORY_IMPAIRMENT	23	0.290	1.286	0.16141002	0.68677104	1	864	tags=65%, lis
GOMF_AMIDE_BINDING	50	0.224	1.286	0.14661655	0.68652356	1	487	tags=34%, lis
GOBP_NEGATIVE_REGULATION_OF_TYPE_I_INTERFERON_PRODUCTION	15	0.333	1.285	0.18773234	0.6872174	1	1007	tags=73%, lis
REACTOME_COPI_DEPENDENT_GOLGI_TO_ER_RETROGRADE_TRAFFIC	19	0.310	1.285	0.1712204	0.6864321	1	905	tags=58%, lis
HP_CONGESTIVE_HEART_FAILURE	36	0.247	1.284	0.16171004	0.6866484	1	812	tags=53%, lis
GOBP_T_CELL_ACTIVATION_INVOLVED_IN_IMMUNE_RESPONSE	21	0.304	1.283	0.1754386	0.6875828	1	558	tags=43%, lis
HP_PARAPARESIS	19	0.311	1.283	0.17120622	0.68660206	1	1167	tags=79%, lis
HP_MISALIGNMENT_OF_TEETH	41	0.240	1.282	0.16077739	0.6854005	1	221	tags=22%, lis
HP_X_LINKED_RECESSIVE_INHERITANCE	30	0.259	1.281	0.16984732	0.6870353	1	382	tags=30%, lis
GOCC_MITOCHONDRION	296	0.150	1.277	0.07308970	0.69581807	1	832	tags=42%, lis
GOBP_CELLULAR_RESPONSE_TO_OXYGEN_LEVELS	57	0.216	1.274	0.14756945	0.7024429	1	958	tags=54%, lis
HP_DILATED_FOURTH_VENTRICLE	23	0.287	1.274	0.17509025	0.7010822	1	604	tags=43%, lis
GOBP_CHAPERONE_COFACTOR_DEPENDENT_PROTEIN_REFOLDING	15	0.337	1.274	0.2016129	0.7012373	1	637	tags=47%, lis
HP_INCREASED_CSF_LACTATE	19	0.308	1.271	0.19136961	0.70652425	1	1419	tags=89%, lis
GOBP_LEUKOCYTE_CHEMOTAXIS	34	0.247	1.269	0.17657992	0.71150345	1	878	tags=53%, lis
HP_FAILURE_TO_THRIVE_IN_INFANCY	16	0.327	1.268	0.18819188	0.71279573	1	574	tags=44%, lis
GOBP_MEIOTIC_CELL_CYCLE_PROCESS	15	0.330	1.267	0.19577736	0.71408546	1	909	tags=67%, lis
GOBP_NON_CANONICAL_WNT_SIGNALING_PATHWAY	33	0.251	1.267	0.16396396	0.7122936	1	592	tags=42%, lis
KEGG_RIG_I_LIKE_RECEPTOR_SIGNALING_PATHWAY	15	0.325	1.266	0.20114942	0.7133936	1	858	tags=60%, lis
REACTOME_HIV_LIFE_CYCLE	26	0.269	1.265	0.19266056	0.7129692	1	439	tags=35%, lis
GOCC_LATE_ENDOSOME	59	0.208	1.265	0.17290552	0.71187335	1	905	tags=51%, lis
HP_ABNORMALITY_OF_THE_PERIPHERAL_NERVOUS_SYSTEM	31	0.255	1.264	0.16698292	0.7119534	1	80	tags=16%, lis
GOBP_NEGATIVE_REGULATION_OF_RNA_CATABOLIC_PROCESS	17	0.314	1.261	0.20113853	0.7182065	1	617	tags=53%, lis
REACTOME_TRANSCRIPTIONAL_REGULATION_BY_RUNX3	35	0.244	1.260	0.17962962	0.7190157	1	667	tags=43%, lis
GOBP_LYSOSOMAL_TRANSPORT	35	0.245	1.259	0.18928571	0.72090745	1	1084	tags=66%, lis
GOBP_REGULATION_OF_CELL_CYCLE_G2_M_PHASE_TRANSITION	42	0.232	1.259	0.18491921	0.7209804	1	682	tags=45%, lis
GOBP_NEGATIVE_REGULATION_OF_INTRINSIC_APOPTOTIC_SIGNALING_PATHWAY	18	0.303	1.258	0.20669292	0.72069544	1	312	tags=28%, lis
GOBP_REGULATION_OF_DEFENSE_RESPONSE_TO_VIRUS	15	0.331	1.256	0.20260224	0.72523355	1	1198	tags=80%, lis
REACTOME_UNFOLDED_PROTEIN_RESPONSE_UPR	28	0.266	1.255	0.18478261	0.7249071	1	938	tags=61%, lis
REACTOME_GENE_SILENCING_BY_RNA	15	0.324	1.255	0.19274808	0.7230787	1	411	tags=40%, lis
HP_UNSTEADY_GAIT	24	0.279	1.254	0.21715328	0.7264796	1	861	tags=54%, lis
HP_INCREASED_SERUM_LACTATE	32	0.248	1.252	0.19188192	0.72994167	1	1097	tags=63%, lis
GOBP_NEGATIVE_REGULATION_OF_GROWTH	37	0.243	1.250	0.18978103	0.7327794	1	644	tags=41%, lis
GOCC_MITOCHONDRIAL_PROTEIN_CONTAINING_COMPLEX	58	0.207	1.250	0.16964285	0.73243374	1	899	tags=52%, lis
HP_SPINAL_DYSRAPHISM	23	0.279	1.250	0.19202898	0.7306441	1	224	tags=26%, lis
HP_CONSTIPATION	59	0.209	1.249	0.18566176	0.72959024	1	719	tags=39%, lis
GOBP_MITOTIC_SISTER_CHROMATID_SEGREGATION	21	0.288	1.249	0.19619048	0.7277638	1	561	tags=43%, lis
GOBP_ANTIGEN_PROCESSING_AND_PRESENTATION_OF_PEPTIDE_OR_POLYSACCHARIDE_ANTIGEN	27	0.265	1.248	0.20113853	0.72795165	1	248	tags=22%, lis
KEGG_PURINE_METABOLISM	20	0.288	1.248	0.21153846	0.7270108	1	865	tags=55%, lis
GOBP_PROTEIN_POLYUBIQUITINATION	93	0.184	1.247	0.159132	0.7289563	1	704	tags=38%, lis
GOBP_REGULATION_OF_LEUKOCYTE_CHEMOTAXIS	17	0.313	1.247	0.19604316	0.7273614	1	188	tags=24%, lis
GOCC_LEADING_EDGE_MEMBRANE	29	0.260	1.247	0.20864661	0.725655	1	645	tags=41%, lis
HP_OSTEOPOROSIS	26	0.271	1.247	0.18441065	0.72388566	1	812	tags=54%, lis
GOBP_POSITIVE_REGULATION_OF_DNA_BINDING_TRANSCRIPTION_FACTOR_ACTIVITY	52	0.219	1.246	0.18829982	0.72481567	1	568	tags=35%, lis
HP_ABNORMAL_CSF_METABOLITE_LEVEL	24	0.273	1.245	0.19005328	0.7241451	1	1419	tags=88%, lis
GOBP_NEGATIVE_REGULATION_OF_RESPONSE_TO_EXTERNAL_STIMULUS	63	0.200	1.245	0.17522123	0.7228138	1	963	tags=56%, lis
GOCC_VACUOLE	180	0.158	1.243	0.1358885	0.7262401	1	619	tags=32%, lis
GOBP_REGULATION_OF_ERBB_SIGNALING_PATHWAY	19	0.298	1.242	0.21335807	0.72785664	1	848	tags=63%, lis
GOBP_RNA_CATABOLIC_PROCESS	96	0.181	1.240	0.17957747	0.7308418	1	620	tags=35%, lis
PID_BCR_5PATHWAY	23	0.278	1.240	0.1846435	0.72992367	1	470	tags=39%, lis
GOCC_SPINDLE	54	0.210	1.239	0.19459459	0.73269904	1	211	tags=19%, lis
GOBP_NCRNA_PROCESSING	43	0.227	1.238	0.20253165	0.7320024	1	1076	tags=67%, lis
HP_ABNORMAL_FORM_OF_THE_VERTEBRAL_BODIES	45	0.222	1.238	0.19860627	0.73190534	1	89	tags=16%, lis
HP_ELEVATED_HEPATIC_TRANSAMINASE	46	0.222	1.237	0.1959707	0.73124725	1	714	tags=46%, lis
GOBP_ORGANELLE_FISSION	58	0.205	1.237	0.19855596	0.7307648	1	667	tags=38%, lis

GOBP_REGULATION_OF_CATABOLIC_PROCESS	217	0.151	1.236	0.12815884	0.73240095	1	620	tags=33%, lis
GOBP_INTERLEUKIN_1_BETA_PRODUCTION	18	0.304	1.235	0.20604914	0.7319115	1	4	tags=11%, lis
HP_ABNORMALITY_OF_THE_PHARYNX	30	0.251	1.235	0.1875	0.7305248	1	476	tags=30%, lis
HP_ABNORMALITY_OF_THE_METACARPAL_BONES	34	0.246	1.234	0.20895523	0.7330037	1	89	tags=18%, lis
GOBP_REGULATION_OF_RESPONSE_TO_STRESS	267	0.146	1.233	0.13213703	0.733896	1	592	tags=29%, lis
GOBP_TISSUE_HOMEOSTASIS	35	0.243	1.232	0.21797323	0.7337468	1	314	tags=26%, lis
HP_GLIOSIS	17	0.305	1.231	0.20733945	0.7340154	1	1200	tags=82%, lis
GOBP_PEPTIDE_SECRETION	58	0.203	1.231	0.18065694	0.73459744	1	930	tags=55%, lis
GOBP_ATP_METABOLIC_PROCESS	78	0.191	1.230	0.19611308	0.7336345	1	771	tags=42%, lis
GOBP_REGULATION_OF_PROTEIN_STABILITY	67	0.197	1.230	0.22180451	0.73399866	1	442	tags=27%, lis
HP_INFANTILE_MUSCULAR_HYPOTONIA	23	0.276	1.229	0.2148289	0.73291343	1	524	tags=43%, lis
GOCC_ORGANELLE_INNER_MEMBRANE	104	0.174	1.227	0.18971631	0.7365125	1	910	tags=48%, lis
HP_GENERALIZED_HYPOTONIA	178	0.157	1.222	0.1590106	0.7503217	1	812	tags=43%, lis
REACTOME_BETA_CATENIN_INDEPENDENT_WNT_SIGNALING	38	0.228	1.222	0.2063197	0.7502361	1	592	tags=39%, lis
GOBP_REGULATION_OF_GENERATION_OF_PRECURSOR_METABOLITES_AND_ENERGY	30	0.253	1.221	0.2172352	0.75124586	1	506	tags=37%, lis
HP_PIGMENTARY_RETINOPATHY	21	0.283	1.219	0.2204301	0.7551192	1	1257	tags=81%, lis
GOBP_INTRINSIC_APOPTOTIC_SIGNALING_PATHWAY_IN_RESPONSE_TO_DNA_DAMAGE	24	0.271	1.219	0.23247233	0.7545236	1	37	tags=13%, lis
HP_DYSARTHRIA	83	0.184	1.218	0.18848167	0.7535164	1	870	tags=46%, lis
GOBP_NEGATIVE_REGULATION_OF_IMMUNE_RESPONSE	27	0.262	1.217	0.21771218	0.75505376	1	729	tags=48%, lis
GOBP_POSITIVE_REGULATION_OF_CHEMOTAXIS	17	0.306	1.217	0.20955883	0.7533566	1	188	tags=24%, lis
GOBP_CELL_RECOGNITION	19	0.298	1.216	0.22686026	0.7542949	1	419	tags=42%, lis
GOBP_PROTEIN_COMPLEX_OLIGOMERIZATION	34	0.241	1.215	0.23134328	0.7551824	1	476	tags=32%, lis
GOBP_POSITIVE_REGULATION_OF_PHAGOCYTOSIS	16	0.311	1.213	0.23047619	0.7596642	1	635	tags=50%, lis
HP_ABNORMAL_CRANIAL_NERVE_MORPHOLOGY	33	0.239	1.213	0.23508772	0.75804853	1	807	tags=55%, lis
HP_ABNORMAL_VASCULAR_PHYSIOLOGY	38	0.231	1.213	0.24118738	0.75763404	1	314	tags=21%, lis
HP_JUVENILE_ONSET	24	0.268	1.212	0.2457786	0.7573816	1	1045	tags=67%, lis
GOBP_VESICLE_BUDDING_FROM_MEMBRANE	26	0.258	1.212	0.22568807	0.7571326	1	479	tags=35%, lis
GOBP_POSITIVE_REGULATION_OF_MRNA_METABOLIC_PROCESS	33	0.240	1.211	0.24482109	0.75623196	1	398	tags=30%, lis
GOBP_POSITIVE_REGULATION_OF_INFLAMMATORY_RESPONSE	29	0.248	1.211	0.2311828	0.7549028	1	832	tags=52%, lis
GOBP_POSITIVE_REGULATION_OF_PROTEIN_KINASE_B_SIGNALING	29	0.253	1.211	0.2086331	0.75442964	1	543	tags=38%, lis
GOBP_VACUOLAR_TRANSPORT	50	0.214	1.210	0.21568628	0.75359553	1	940	tags=56%, lis
REACTOME_FC_EPSILON_RECEPTOR_FCERI_SIGNALING	44	0.218	1.210	0.19963032	0.75387394	1	592	tags=39%, lis
GOBP_POSITIVE_REGULATION_OF_I_KAPPAB_KINASE_NF_KAPPAB_SIGNALING	47	0.208	1.209	0.20255475	0.7540299	1	550	tags=34%, lis
GOBP_FEMALE_SEX_DIFFERENTIATION	21	0.281	1.209	0.21937843	0.752723	1	1067	tags=67%, lis
GOBP_POSITIVE_REGULATION_OF_WNT_SIGNALING_PATHWAY	40	0.227	1.209	0.22994652	0.75235337	1	958	tags=58%, lis
GOMF_OXIDOREDUCTASE_ACTIVITY_ACTING_ON_NAD_P_H	34	0.233	1.208	0.22486289	0.7513	1	1541	tags=88%, lis
GOMF_GTPASE_ACTIVITY	46	0.219	1.207	0.22380106	0.7541895	1	635	tags=35%, lis
GOBP_FC_EPSILON_RECEPTOR_SIGNALING_PATHWAY	36	0.236	1.207	0.2228261	0.7530756	1	592	tags=42%, lis
HP_ABNORMAL_LYMPHOCYTE_MORPHOLOGY	37	0.231	1.205	0.2228164	0.75503653	1	489	tags=32%, lis
HP_ABNORMALITY_OF_THE_MITOCHONDRION	37	0.232	1.205	0.21484375	0.7551275	1	899	tags=51%, lis
HP_ABNORMALITY_OF_EPIPHYSIS_MORPHOLOGY	42	0.222	1.203	0.24021353	0.7590881	1	275	tags=21%, lis
HP_VASCULAR_NEOPLASM	23	0.266	1.202	0.25373134	0.7608753	1	396	tags=35%, lis
HP_ABNORMAL_BLOOD_GLUCOSE_CONCENTRATION	27	0.255	1.202	0.20879121	0.75967693	1	944	tags=59%, lis
REACTOME_TP53_REGULATES_METABOLIC_GENES	26	0.254	1.201	0.25141776	0.7593388	1	471	tags=35%, lis
GOMF_IDENTICAL_PROTEIN_BINDING	297	0.138	1.200	0.15270936	0.7599623	1	765	tags=37%, lis
GOMF_PROTEIN_DIMERIZATION_ACTIVITY	136	0.161	1.200	0.17171717	0.76024115	1	782	tags=41%, lis
GOBP_SECRETION	259	0.143	1.200	0.15224913	0.75858945	1	569	tags=30%, lis
GOCC_EXTERNAL_SIDE_OF_PLASMA_MEMBRANE	39	0.225	1.199	0.2358156	0.75870204	1	568	tags=36%, lis
REACTOME_M_PHASE	67	0.192	1.199	0.22867514	0.7570262	1	682	tags=39%, lis
HP_OPTIC_ATROPHY	86	0.177	1.197	0.21801803	0.76038235	1	902	tags=50%, lis
REACTOME_TNFR1_INDUCED_NFKAPPAB_SIGNALING_PATHWAY	15	0.317	1.197	0.24236642	0.76020247	1	901	tags=60%, lis
HP_ENLARGED_POSTERIOR_FOSSA	24	0.266	1.196	0.2513661	0.761516	1	604	tags=42%, lis
HP_DEPRESSIVITY	60	0.196	1.195	0.21328671	0.76200914	1	1008	tags=57%, lis
GOBP_INSULIN_SECRETION	33	0.239	1.194	0.2421875	0.7624839	1	635	tags=42%, lis
GOBP_VESICLE_TARGETING_TO_FROM_OR_WITHIN_GOLGI	19	0.279	1.194	0.26654065	0.76140964	1	474	tags=37%, lis
HP_ABNORMAL_BLOOD_INORGANIC_CATION_CONCENTRATION	23	0.264	1.194	0.21104537	0.7598577	1	807	tags=52%, lis
HP_ABNORMALITY_OF_EXTRAPYRAMIDAL_MOTOR_FUNCTION	53	0.205	1.193	0.24270073	0.7605255	1	1082	tags=60%, lis
GOBP_NEGATIVE_REGULATION_OF_TRANSPORT	75	0.184	1.193	0.21352313	0.7588527	1	547	tags=32%, lis
GOMF_IMMUNE_RECEPTOR_ACTIVITY	18	0.285	1.193	0.22813688	0.7572928	1	313	tags=28%, lis
GOBP_MORPHOGENESIS_OF_AN_EPITHELIUM	62	0.192	1.193	0.21518987	0.7558832	1	750	tags=44%, lis
GOBP_LEUKOCYTE_HOMEOSTASIS	17	0.291	1.192	0.23047619	0.7565331	1	720	tags=53%, lis
GOBP_MODIFICATION_DEPENDENT_MACROMOLECULE_CATABOLIC_PROCESS	158	0.158	1.190	0.18739353	0.76134664	1	712	tags=37%, lis
GOBP_PROTON_TRANSMEMBRANE_TRANSPORT	32	0.237	1.189	0.2434457	0.7620935	1	753	tags=50%, lis
GOCC_CENTRIOLAR_SATELLITE	18	0.291	1.189	0.26213592	0.7618111	1	64	tags=17%, lis
HP_ABNORMALITY_OF_HAIR_TEXTURE	28	0.250	1.189	0.24282983	0.7611561	1	678	tags=50%, lis
REACTOME_RHOBTB_GTPASE_CYCLE	18	0.293	1.189	0.2518797	0.75964564	1	316	tags=33%, lis
GOMF_P53_BINDING	15	0.312	1.188	0.26545453	0.75899285	1	433	tags=33%, lis
GOCC_ENDOSOME	177	0.151	1.187	0.18505943	0.76091796	1	569	tags=31%, lis
GOBP_TISSUE_MORPHOGENESIS	72	0.185	1.185	0.21481481	0.76377857	1	983	tags=57%, lis
GOBP_STEM_CELL_DIFFERENTIATION	49	0.207	1.185	0.25044405	0.76382715	1	603	tags=41%, lis
REACTOME_RESOLUTION_OF_SISTER_CHROMATID_COHESION	15	0.313	1.183	0.26306307	0.7670265	1	211	tags=27%, lis
HP_ABNORMAL_CRANIAL_NERVE_PHYSIOLOGY	35	0.226	1.183	0.22719142	0.7657854	1	807	tags=51%, lis
GOCC_GOLGI_APPARATUS_SUBCOMPARTMENT	170	0.154	1.181	0.20176992	0.77017343	1	522	tags=26%, lis
HP_SKIN_ULCER	20	0.281	1.181	0.23562153	0.769192	1	662	tags=45%, lis
GOBP_GRANULOCYTE_CHEMOTAXIS	16	0.303	1.181	0.25703564	0.7681133	1	558	tags=44%, lis
HP_ABNORMALITY_OF_COAGULATION	29	0.242	1.180	0.26873857	0.76798135	1	694	tags=48%, lis
GOBP_SULFUR_COMPOUND_METABOLIC_PROCESS	57	0.197	1.180	0.23134328	0.76710755	1	660	tags=35%, lis
HP_LEUKOPENIA	35	0.226	1.179	0.23507462	0.7675647	1	489	tags=31%, lis
GOCC_MITOCHONDRIAL_ENVELOPE	147	0.156	1.179	0.20683761	0.7664189	1	910	tags=46%, lis

REACTOME_GOLGI_ASSOCIATED_VESICLE_BIOGENESIS	20	0.273	1.179	0.26142597	0.76485693	1	905	tags=55%, lis
REACTOME_DEUBIQUITINATION	71	0.190	1.179	0.24196598	0.76340866	1	683	tags=41%, lis
HP_APLASIA_HYPOPLASIA_OF_THE_EYEBROW	20	0.274	1.179	0.27567568	0.7628971	1	477	tags=40%, lis
REACTOME_POST_TRANSLATIONAL_PROTEIN_MODIFICATION	269	0.139	1.178	0.16523236	0.7641025	1	495	tags=26%, lis
GOBP_TAXIS	75	0.178	1.178	0.2297794	0.76251173	1	342	tags=21%, lis
GOCC_LAMELLIPODIUM	39	0.222	1.177	0.2556391	0.761502	1	589	tags=38%, lis
GOBP_REGULATION_OF_CHEMOTAXIS	28	0.250	1.177	0.23091976	0.7602075	1	314	tags=25%, lis
GOCC_SIDE_OF_MEMBRANE	81	0.178	1.176	0.228223	0.7622062	1	568	tags=33%, lis
HALLMARK_E2F_TARGETS	43	0.213	1.174	0.24021353	0.7665893	1	865	tags=51%, lis
GOBP_REGULATION_OF_B_CELL_PROLIFERATION	18	0.282	1.174	0.2548638	0.76575106	1	298	tags=28%, lis
GOBP_NEGATIVE_REGULATION_OF_CELLULAR_AMIDE_METABOLIC_PROCESS	50	0.201	1.172	0.24460432	0.7700217	1	327	tags=24%, lis
HP_APLASIA_HYPOPLASIA_INVOLVING_BONES_OF_THE_THORAX	21	0.271	1.172	0.29056603	0.7686354	1	803	tags=52%, lis
GOBP_NEGATIVE_REGULATION_OF_CYSTEINE_TYPE_ENDOPEPTIDASE_ACTIVITY	16	0.295	1.171	0.26550388	0.77051604	1	24	tags=13%, lis
GOBP_SPINDLE_ASSEMBLY	19	0.286	1.170	0.27165353	0.7699664	1	621	tags=42%, lis
REACTOME_DEGRADATION_OF_BETA_CATENIN_BY_THE_DESTRUCTION_COMPLEX	30	0.235	1.167	0.26275992	0.7783794	1	592	tags=40%, lis
GOMF_UBIQUITIN_LIKE_PROTEIN_LIGASE_BINDING	83	0.174	1.167	0.24682395	0.77684635	1	644	tags=35%, lis
REACTOME_RHO_GTPASE_EFFECTORS	42	0.214	1.165	0.26978418	0.77991575	1	438	tags=29%, lis
GOBP_REGULATION_OF_CELLULAR_CATABOLIC_PROCESS	178	0.149	1.164	0.22203098	0.7824684	1	620	tags=33%, lis
REACTOME_GOLGI_TO_ER_RETROGRADE_TRANSPORT	26	0.251	1.164	0.256705	0.7811975	1	297	tags=23%, lis
HP_POLYMICROGYRIA	37	0.227	1.163	0.26003492	0.7812721	1	694	tags=43%, lis
GOBP_PROTEASOMAL_PROTEIN_CATABOLIC_PROCESS	119	0.165	1.163	0.22463769	0.7798782	1	518	tags=29%, lis
GOBP_ENDOSOME_ORGANIZATION	20	0.276	1.163	0.3045045	0.7791578	1	607	tags=40%, lis
GOBP_RESPONSE_TO_IONIZING_RADIATION	27	0.252	1.163	0.28486055	0.7778119	1	704	tags=44%, lis
HP_SLEEP_DISTURBANCE	85	0.174	1.162	0.21390374	0.77734125	1	724	tags=39%, lis
PID_TNF_PATHWAY	17	0.288	1.162	0.2783883	0.7761135	1	518	tags=35%, lis
HP_ABNORMALITY_OF_THE_ABDOMINAL_ORGANS	168	0.150	1.161	0.20677362	0.7787367	1	644	tags=33%, lis
HALLMARK_TNFA_SIGNALING_VIA_NFKB	61	0.193	1.160	0.26497278	0.7806712	1	814	tags=44%, lis
GOMF_HYDROLASE_ACTIVITY_ACTING_ON_CARBON_NITROGEN_BUT_NOT_PEPTIDE_BONDS	15	0.300	1.159	0.24157304	0.78086776	1	860	tags=67%, lis
GOBP_EPIDERMAL_GROWTH_FACTOR_RECEPTOR_SIGNALING_PATHWAY	22	0.263	1.159	0.25884545	0.7799984	1	848	tags=59%, lis
HP_ABNORMAL_CARDIOVASCULAR_SYSTEM_PHYSIOLOGY	166	0.148	1.158	0.2303665	0.78018874	1	314	tags=18%, lis
HP_ABNORMALITY_OF_THE_HYPOTHALAMUS_PITUITARY_AXIS	33	0.226	1.158	0.28153566	0.7803093	1	293	tags=24%, lis
REACTOME_FATTY_ACID_METABOLISM	23	0.259	1.157	0.28489482	0.7803798	1	1301	tags=83%, lis
HP_ABNORMAL_NUMBER_OF_TEETH	45	0.207	1.157	0.28284672	0.7794511	1	194	tags=18%, lis
HP_ABNORMAL_CIRCULATING_AMINO_ACID_CONCENTRATION	22	0.267	1.157	0.2802303	0.7784444	1	554	tags=41%, lis
GOBP_INTERLEUKIN_1_PRODUCTION	21	0.266	1.157	0.26848248	0.7771981	1	557	tags=33%, lis
HP_REDUCED_TENDON_REFLEXES	74	0.180	1.155	0.26788992	0.78095675	1	820	tags=47%, lis
HP_INVOLUNTARY_MOVEMENTS	187	0.144	1.154	0.21516755	0.7806441	1	642	tags=32%, lis
HP_APLASIA_HYPOPLASIA_OF_THE_EAR	22	0.259	1.154	0.29074073	0.7803373	1	394	tags=36%, lis
GOBP_REGULATION_OF_RESPONSE_TO_ENDOPLASMIC_RETICULUM_STRESS	26	0.244	1.153	0.2810219	0.78113616	1	431	tags=31%, lis
HP_DISTAL_AMYOTROPHY	28	0.241	1.153	0.2816092	0.78053445	1	1045	tags=64%, lis
HP_GASTROESOPHAGEAL_REFLUX	60	0.190	1.153	0.25905797	0.7793665	1	215	tags=18%, lis
GOBP_RESPONSE_TO_UV	29	0.237	1.150	0.24647887	0.7872159	1	849	tags=55%, lis
GOBP_SISTER_CHROMATID_SEGREGATION	28	0.240	1.147	0.3041145	0.7939609	1	211	tags=21%, lis
GOBP_MITOCHONDRIAL_ELECTRON_TRANSPORT_NADH_TO_UBIQUINONE	21	0.261	1.146	0.2857143	0.7936415	1	899	tags=52%, lis
GOBP_PROTEIN_LOCALIZATION_TO_ENDOPLASMIC_RETICULUM	19	0.279	1.146	0.28697184	0.79299474	1	445	tags=37%, lis
HALLMARK_OXIDATIVE_PHOSPHORYLATION	63	0.187	1.146	0.24956064	0.7915222	1	899	tags=46%, lis
GOCC_ENVELOPE	235	0.139	1.146	0.23166667	0.7904572	1	757	tags=37%, lis
HP_SPARSE_EYEBROW	15	0.300	1.146	0.29020333	0.78943264	1	477	tags=47%, lis
REACTOME_DEATH_RECEPTOR_SIGNALLING	39	0.214	1.146	0.2962963	0.78818166	1	901	tags=51%, lis
HP_INCREASED_HEAD_CIRCUMFERENCE	76	0.179	1.145	0.27008548	0.78879464	1	724	tags=41%, lis
GOBP_POSITIVE_REGULATION_OF_LEUKOCYTE_MIGRATION	20	0.270	1.145	0.29101562	0.78754187	1	188	tags=20%, lis
HP_NASOGASTRIC_TUBE_FEEDING	18	0.283	1.144	0.30374753	0.7876746	1	675	tags=50%, lis
GOBP_INTRINSIC_APOPTOTIC_SIGNALING_PATHWAY_IN_RESPONSE_TO_ENDOPLASMIC_RETICULU	20	0.274	1.144	0.26779026	0.7863038	1	1165	tags=75%, lis
GOBP_ORGANIC_ACID_BIOSYNTHETIC_PROCESS	36	0.220	1.144	0.2982143	0.7853174	1	198	tags=19%, lis
HALLMARK_G2M_CHECKPOINT	31	0.235	1.144	0.2854512	0.78433377	1	359	tags=29%, lis
GOBP_POSITIVE_REGULATION_OF_CELLULAR_PROTEIN_LOCALIZATION	70	0.179	1.143	0.2788104	0.78615546	1	437	tags=27%, lis
GOBP_REGULATION_OF_CYTOSOLIC_CALCIUM_ION_CONCENTRATION	46	0.199	1.142	0.28956833	0.78799266	1	76	tags=13%, lis
GOBP_POSITIVE_REGULATION_OF_LIPID_METABOLIC_PROCESS	22	0.261	1.142	0.2928709	0.78677195	1	531	tags=41%, lis
GOBP_ERBB_SIGNALING_PATHWAY	27	0.246	1.142	0.29143897	0.78535724	1	689	tags=48%, lis
HP_ECZEMA	25	0.250	1.140	0.30492425	0.7871047	1	503	tags=32%, lis
GOCC_AUTOPHAGOSOME	33	0.228	1.140	0.2893773	0.78809303	1	952	tags=58%, lis
HP_ABNORMAL_CARDIAC_VENTRICLE_MORPHOLOGY	73	0.179	1.138	0.27333334	0.78990847	1	959	tags=53%, lis
GOBP_PALLIUM_DEVELOPMENT	18	0.277	1.138	0.29582578	0.78974247	1	199	tags=22%, lis
HP_ABNORMALITY_OF_CARDIOVASCULAR_SYSTEM_ELECTROPHYSIOLOGY	60	0.186	1.138	0.2759227	0.78961116	1	793	tags=45%, lis
REACTOME_INTRINSIC_PATHWAY_FOR_APOPTOSIS	15	0.302	1.137	0.2938856	0.78871286	1	698	tags=53%, lis
GOBP_POSITIVE_REGULATION_OF_PEPTIDYL_TYROSINE_PHOSPHORYLATION	30	0.234	1.137	0.28947368	0.78854465	1	237	tags=23%, lis
GOMF_METAL_CLUSTER_BINDING	16	0.289	1.137	0.3078394	0.78738517	1	1220	tags=81%, lis
GOCC_NEURON_SPINE	20	0.268	1.136	0.30258304	0.7872986	1	284	tags=30%, lis
GOBP_INTERFERON_BETA_PRODUCTION	15	0.294	1.136	0.28769842	0.7872325	1	1007	tags=67%, lis
GOBP_GRANULOCYTE_MIGRATION	21	0.262	1.133	0.28349516	0.7930303	1	617	tags=43%, lis
HP_ABNORMAL_RESPIRATORY_SYSTEM_PHYSIOLOGY	180	0.145	1.133	0.23956443	0.7919061	1	863	tags=42%, lis
GOMF_PROTON_TRANSMEMBRANE_TRANSPORTER_ACTIVITY	25	0.243	1.133	0.26691043	0.7914047	1	753	tags=52%, lis
GOBP_ANATOMICAL_STRUCTURE_HOMEOSTASIS	70	0.180	1.133	0.2813067	0.79090804	1	427	tags=26%, lis
GOBP_NEUTROPHIL_CHEMOTAXIS	16	0.303	1.133	0.3011811	0.789658	1	558	tags=44%, lis
GOBP_REGULATION_OF_PROTEIN_CONTAINING_COMPLEX_DISASSEMBLY	22	0.256	1.132	0.3118081	0.78976417	1	432	tags=32%, lis
REACTOME_ANTIGEN_PROCESSING_UBIQUITINATION_PROTEASOME_DEGRADATION	81	0.171	1.132	0.28295255	0.7897621	1	503	tags=28%, lis
GOMF_EXOGENOUS_PROTEIN_BINDING	16	0.292	1.131	0.30827066	0.7887027	1	500	tags=44%, lis
GOBP_REGULATION_OF_PEPTIDYL_SERINE_PHOSPHORYLATION	24	0.251	1.131	0.28519857	0.7878563	1	371	tags=29%, lis
HP_ABSENT_SPEECH	49	0.200	1.128	0.3122807	0.794407	1	612	tags=37%, lis

GOBP_REGULATION_OF_ACTIN_FILAMENT_LENGTH	33	0.222	1.127	0.31444243	0.79635173	1	496	tags=30%, lis
HP_CEREBELLAR_HYPOPLASIA	30	0.234	1.127	0.32022473	0.7967567	1	611	tags=37%, lis
KEGG_LYSOSOME	27	0.241	1.126	0.29310346	0.79628086	1	618	tags=41%, lis
GOBP_REGULATION_OF_TOR_SIGNALING	17	0.282	1.126	0.2911153	0.7960038	1	411	tags=29%, lis
GOBP_RNA_DEPENDENT_DNA_BIOSYNTHETIC_PROCESS	16	0.289	1.126	0.30097088	0.79470915	1	427	tags=31%, lis
GOBP_INFLAMMATORY_RESPONSE	120	0.157	1.125	0.27758008	0.79443717	1	557	tags=29%, lis
HP_SENSORY_NEUROPATHY	23	0.245	1.125	0.31332082	0.79336184	1	557	tags=39%, lis
GOBP_CELLULAR_PROTEIN_CATABOLIC_PROCESS	189	0.142	1.125	0.26101694	0.79370826	1	615	tags=31%, lis
HP_MUSCLE_WEAKNESS	141	0.150	1.124	0.26881722	0.7930852	1	812	tags=41%, lis
GOBP_ORGANIC_CYCLIC_COMPOUND_CATABOLIC_PROCESS	117	0.156	1.124	0.27272728	0.7923169	1	620	tags=33%, lis
REACTOME_ASPARAGINE_N_LINKED_GLYCOSYLATION	65	0.180	1.124	0.3021978	0.79291457	1	474	tags=28%, lis
HP_NARROW_PALATE	21	0.260	1.123	0.33274022	0.79210645	1	167	tags=24%, lis
REACTOME_TRANS_GOLGI_NETWORK_VESICLE_BUDDING	27	0.236	1.123	0.2935943	0.7921121	1	905	tags=56%, lis
GOMF_HYDROLASE_ACTIVITY_ACTING_ON_ACID_ANHYDRIDES	133	0.149	1.122	0.25900516	0.7917962	1	909	tags=47%, lis
REACTOME_C_TYPE_LECTIN_RECEPTORS_CLRS	46	0.202	1.122	0.3430127	0.79066396	1	918	tags=54%, lis
HP_ABNORMALITY_OF_THE_SEPTUM_PELLUCIDUM	20	0.260	1.122	0.3061594	0.7894574	1	224	tags=25%, lis
GOBP_ANIMAL_ORGAN_MORPHOGENESIS	99	0.163	1.122	0.28825623	0.7890167	1	499	tags=29%, lis
PID_RAC1_PATHWAY	15	0.294	1.122	0.31978798	0.78766567	1	438	tags=33%, lis
GOCC_PHAGOCYTIC_VESICLE_MEMBRANE	25	0.244	1.121	0.30755064	0.78856015	1	549	tags=32%, lis
HP_ABNORMALITY_OF_LIMB_EPIPHYSIS_MORPHOLOGY	19	0.278	1.121	0.32068312	0.78764886	1	89	tags=21%, lis
GOBP_GENERATION_OF_PRECURSOR_METABOLITES_AND_ENERGY	123	0.153	1.121	0.28288287	0.78696656	1	835	tags=41%, lis
HP_ABNORMAL_PULMONARY_VALVE_MORPHOLOGY	17	0.285	1.120	0.3126177	0.78800166	1	314	tags=29%, lis
GOBP_PEPTIDE_HORMONE_SECRETION	35	0.214	1.120	0.30091742	0.78749484	1	635	tags=40%, lis
REACTOME_HCMV_INFECTION	21	0.257	1.120	0.32287824	0.7862852	1	849	tags=52%, lis
HP_NEUROLOGICAL_SPEECH_IMPAIRMENT	196	0.136	1.119	0.27538726	0.7874476	1	870	tags=43%, lis
GOBP_RESPONSE_TO_TOPOLOGICALLY_INCORRECT_PROTEIN	61	0.186	1.119	0.30645162	0.7861412	1	445	tags=26%, lis
GOBP_NEGATIVE_REGULATION_OF_WNT_SIGNALING_PATHWAY	46	0.194	1.119	0.3099631	0.78482324	1	489	tags=33%, lis
GOBP_OSTEOCLAST_DIFFERENTIATION	16	0.285	1.117	0.3139746	0.78741974	1	275	tags=31%, lis
GOBP_PROTEIN_MATURATION	39	0.207	1.117	0.33027524	0.7880096	1	352	tags=21%, lis
GOBP_VACUOLE_ORGANIZATION	40	0.207	1.116	0.3429603	0.78742164	1	508	tags=30%, lis
GOBP_ERAD_PATHWAY	24	0.248	1.116	0.31015038	0.7876611	1	295	tags=25%, lis
HP_ABNORMALITY_OF_BONE_MINERAL_DENSITY	56	0.188	1.115	0.3015873	0.78711796	1	812	tags=45%, lis
HP_OPTIC_DISC_PALLOR	21	0.262	1.115	0.3262136	0.7862361	1	642	tags=43%, lis
GOBP_LAMELLIPODIUM_ASSEMBLY	15	0.292	1.115	0.31861803	0.78534395	1	224	tags=27%, lis
HP_HEMIPLEGIA_HEMIPARESIS	29	0.232	1.115	0.3294347	0.7844211	1	980	tags=59%, lis
HP_ABNORMAL_VERTEBRAL_MORPHOLOGY	53	0.190	1.115	0.30198914	0.78324646	1	89	tags=13%, lis
GOBP_ACTIN_FILAMENT_ORGANIZATION	73	0.176	1.115	0.31272084	0.78194004	1	504	tags=29%, lis
GOCC_OXIDOREDUCTASE_COMPLEX	37	0.210	1.115	0.3253012	0.78104	1	1088	tags=59%, lis
HP_ABNORMALITY_OF_THE_CEREBRAL_CORTEX	62	0.181	1.114	0.32637572	0.7822527	1	736	tags=40%, lis
HP_ABNORMAL_CNS_MYELINATION	59	0.188	1.114	0.3063063	0.7813249	1	1281	tags=73%, lis
GOBP_NEGATIVE_REGULATION_OF_PROTEIN_CONTAINING_COMPLEX_ASSEMBLY	23	0.251	1.113	0.33517495	0.78123486	1	637	tags=43%, lis
GOBP_SELECTIVE_AUTOPHAGY	18	0.271	1.113	0.30075186	0.78029746	1	912	tags=61%, lis
HP_ABNORMAL_EMOTION_AFFECT_BEHAVIOR	97	0.159	1.113	0.3068783	0.77995855	1	1008	tags=53%, lis
GOBP_MONOVALENT_INORGANIC_CATION_HOMEOSTASIS	24	0.243	1.113	0.31471136	0.7787913	1	729	tags=46%, lis
GOMF_LIGASE_ACTIVITY	30	0.225	1.112	0.3537285	0.7790653	1	1202	tags=70%, lis
REACTOME_CLEC7A_DECTIN_1_SIGNALING	36	0.211	1.112	0.33510637	0.778418	1	592	tags=39%, lis
GOBP_PROCESS_UTILIZING_AUTOPHAGIC_MECHANISM	107	0.159	1.111	0.28928572	0.7781828	1	561	tags=29%, lis
GOCC_INTRACELLULAR_PROTEIN_CONTAINING_COMPLEX	177	0.141	1.110	0.29913044	0.7803335	1	510	tags=27%, lis
GOMF_TRANSLATION_REGULATOR_ACTIVITY	29	0.228	1.110	0.31499052	0.77936745	1	256	tags=21%, lis
GOBP_AEROBIC_RESPIRATION	24	0.240	1.110	0.30975142	0.77817726	1	744	tags=46%, lis
GOBP_MITOCHONDRIAL_RESPIRATORY_CHAIN_COMPLEX_ASSEMBLY	32	0.224	1.110	0.3012939	0.77759767	1	899	tags=50%, lis
GOBP_RESPONSE_TO_ENDOPLASMIC_RETICULUM_STRESS	83	0.167	1.110	0.28947368	0.776376	1	312	tags=20%, lis
HP_POOR_SPEECH	51	0.190	1.110	0.32562277	0.77546877	1	729	tags=39%, lis
GOBP_REGULATION_OF_ACTIN_FILAMENT_ORGANIZATION	52	0.194	1.110	0.3309481	0.7743611	1	290	tags=21%, lis
HP_HETEROGENEOUS	24	0.244	1.110	0.3032015	0.77315354	1	310	tags=25%, lis
HP_OSTEOPENIA	27	0.228	1.109	0.32971016	0.77444065	1	300	tags=22%, lis
GOCC_COATED_VESICLE_MEMBRANE	45	0.198	1.107	0.3201581	0.7765597	1	441	tags=29%, lis
GOBP_RESPONSE_TO_DRUG	50	0.189	1.107	0.31878558	0.7766536	1	744	tags=44%, lis
HP_PEDIATRIC_ONSET	101	0.162	1.107	0.30664396	0.775772	1	474	tags=26%, lis
GOBP_POSITIVE_REGULATION_OF_ESTABLISHMENT_OF_PROTEIN_LOCALIZATION_TO_MITOCHONI	17	0.278	1.106	0.34296724	0.7765724	1	437	tags=35%, lis
HP_FEEDING_DIFFICULTIES	167	0.142	1.106	0.2872154	0.7754785	1	629	tags=32%, lis
HP_ABNORMAL_SACCADIC_EYE_MOVEMENTS	15	0.284	1.106	0.3275194	0.77543265	1	770	tags=53%, lis
GOBP_ENERGY_DERIVATION_BY_OXIDATION_OF_ORGANIC_COMPOUNDS	73	0.173	1.105	0.32667875	0.7742318	1	899	tags=45%, lis
HP_ABNORMALITY_OF_THE_UVULA	18	0.270	1.104	0.3393502	0.77577364	1	619	tags=39%, lis
HP_CEREBELLAR_CYST	19	0.264	1.104	0.33396947	0.7754165	1	604	tags=42%, lis
REACTOME_RHO_GTPASES_ACTIVATE_FORMINS	15	0.285	1.104	0.3125	0.7749684	1	211	tags=27%, lis
HP_OTITIS_MEDIA	48	0.195	1.103	0.3345521	0.7753979	1	422	tags=27%, lis
GOBP_GLYCOPROTEIN_METABOLIC_PROCESS	65	0.179	1.103	0.33274338	0.7759242	1	486	tags=26%, lis
GOBP_DNA_TEMPLATED_TRANSCRIPTION_TERMINATION	15	0.283	1.102	0.3251418	0.7755056	1	911	tags=60%, lis
GOCC_ENDOCYTIC_VESICLE_MEMBRANE	42	0.204	1.101	0.3181818	0.77699673	1	479	tags=29%, lis
GOBP_POSITIVE_REGULATION_OF_SIGNALING	307	0.126	1.101	0.2760943	0.7771892	1	550	tags=27%, lis
HP_RECURRENT_BACTERIAL_INFECTIONS	27	0.229	1.101	0.34705883	0.7762375	1	245	tags=19%, lis
HP_ABNORMALITY_OF_THE_OPTIC_NERVE	122	0.152	1.100	0.3142361	0.775624	1	854	tags=44%, lis
GOBP_PHAGOCYTOSIS	65	0.175	1.100	0.33041957	0.77514446	1	645	tags=35%, lis
GOBP_PROTEIN_MODIFICATION_BY_SMALL_PROTEIN_CONJUGATION	205	0.134	1.100	0.30071175	0.7739619	1	418	tags=21%, lis
GOBP_RESPONSE_TO_EXTRACELLULAR_STIMULUS	72	0.175	1.099	0.32566372	0.77621144	1	644	tags=36%, lis
HP_PROPTOSIS	37	0.208	1.099	0.33020636	0.7756998	1	224	tags=22%, lis
HP_BIFID_UVULA	18	0.270	1.098	0.3258427	0.7766494	1	619	tags=39%, lis
GOBP_REACTIVE_OXYGEN_SPECIES_BIOSYNTHETIC_PROCESS	23	0.250	1.097	0.35218978	0.77621	1	506	tags=35%, lis

GOBP_CELLULAR_MACROMOLECULE_CATABOLIC_PROCESS	264	0.129	1.097	0.27966103	0.7751825	1	620	tags=30%, lis
HP_BLINDNESS	36	0.208	1.097	0.31784385	0.774523	1	618	tags=39%, lis
HP_ABNORMALITY_OF_THE_LYMPHATIC_SYSTEM	104	0.155	1.097	0.33972126	0.7735324	1	644	tags=35%, lis
GOBP_LYTIC_VACUOLE_ORGANIZATION	21	0.253	1.094	0.3276515	0.78112876	1	618	tags=38%, lis
GOBP_RESPONSE_TO_ETHANOL	19	0.260	1.093	0.32789856	0.7819696	1	1043	tags=63%, lis
HP_MELANOMA	15	0.285	1.093	0.35728157	0.78127146	1	345	tags=33%, lis
GOBP_CELL_ACTIVATION	292	0.128	1.092	0.3004695	0.7825614	1	506	tags=25%, lis
GOBP_MITOTIC_SPINDLE_ORGANIZATION	21	0.252	1.092	0.330855	0.78137314	1	312	tags=24%, lis
GOMF_ACTIN_FILAMENT_BINDING	28	0.223	1.091	0.35571688	0.7837175	1	1158	tags=68%, lis
REACTOME_RAC3_GTPASE_CYCLE	29	0.226	1.090	0.34501845	0.784185	1	91	tags=14%, lis
GOBP_POSITIVE_REGULATION_OF_PEPTIDYL_SERINE_PHOSPHORYLATION	15	0.289	1.090	0.35907337	0.7838588	1	371	tags=33%, lis
HP_ABNORMAL_RECTUM_MORPHOLOGY	19	0.258	1.089	0.34839925	0.78344536	1	1386	tags=84%, lis
GOCC_EARLY_ENDOSOME	73	0.172	1.089	0.3505338	0.7827723	1	251	tags=19%, lis
HP_ABNORMAL_TRACHEOBRONCHIAL_MORPHOLOGY	36	0.213	1.088	0.3432836	0.78549135	1	1132	tags=64%, lis
HALLMARK_HEME_METABOLISM	36	0.211	1.088	0.35185185	0.78436077	1	1097	tags=61%, lis
GOBP_MACROPHAGE_ACTIVATION	20	0.255	1.088	0.34313726	0.78342116	1	897	tags=60%, lis
GOCC_SPECIFIC_GRANULE	36	0.209	1.087	0.3504587	0.7835321	1	486	tags=31%, lis
GOBP_ACTOMYOSIN_STRUCTURE_ORGANIZATION	27	0.234	1.087	0.34339622	0.7832885	1	294	tags=26%, lis
HP_ABNORMAL_CARDIAC_SEPTUM_MORPHOLOGY	86	0.165	1.087	0.3478261	0.7823167	1	736	tags=37%, lis
REACTOME_CELL_CYCLE	112	0.152	1.086	0.3409894	0.7831457	1	720	tags=38%, lis
HP_INCREASED_INTRACRANIAL_PRESSURE	15	0.282	1.084	0.33771107	0.7864437	1	293	tags=33%, lis
HP_LARGE_FACE	39	0.207	1.084	0.36481482	0.785667	1	793	tags=46%, lis
PID_IL6_7_PATHWAY	19	0.258	1.084	0.3564728	0.78449607	1	359	tags=32%, lis
GOMF_G_PROTEIN_COUPLED_RECEPTOR_ACTIVITY	18	0.266	1.084	0.33697632	0.78376114	1	506	tags=33%, lis
HP_ABNORMALITY_OF_THE_LIVER	152	0.141	1.084	0.3257443	0.7836846	1	642	tags=33%, lis
GOBP_REGULATION_OF_PATTERN_RECOGNITION_RECEPTOR_SIGNALING_PATHWAY	35	0.212	1.083	0.34352517	0.78397274	1	1068	tags=66%, lis
HP_ABNORMAL_SCAPULA_MORPHOLOGY	20	0.254	1.082	0.35120147	0.7861221	1	803	tags=55%, lis
GOBP_CELLULAR_RESPONSE_TO_REACTIVE_OXYGEN_SPECIES	31	0.220	1.082	0.3418647	0.78503036	1	683	tags=39%, lis
HP_ABNORMAL_IMMUNE_SYSTEM_MORPHOLOGY	106	0.151	1.081	0.35924006	0.78468734	1	495	tags=26%, lis
GOBP_RESPONSE_TO_NITROGEN_COMPOUND	195	0.135	1.081	0.31083483	0.78563684	1	644	tags=32%, lis
GOBP_NEGATIVE_REGULATION_OF_CELL_GROWTH	31	0.221	1.080	0.35795453	0.78563243	1	644	tags=39%, lis
KEGG_PANCREATIC_CANCER	20	0.251	1.079	0.3581818	0.78665197	1	345	tags=30%, lis
GOBP_NEGATIVE_REGULATION_OF_CANONICAL_WNT_SIGNALING_PATHWAY	43	0.197	1.078	0.3482309	0.7877421	1	489	tags=33%, lis
HP_INCOORDINATION	28	0.223	1.078	0.37269372	0.78723276	1	461	tags=32%, lis
GOBP_CELL_CHEMOTAXIS	42	0.199	1.077	0.34709194	0.7891273	1	878	tags=48%, lis
GOBP_PROTEIN_MODIFICATION_BY_SMALL_PROTEIN_CONJUGATION_OR_REMOVAL	252	0.131	1.077	0.32692307	0.7880731	1	715	tags=34%, lis
GOBP_ELECTRON_TRANSPORT_CHAIN	55	0.182	1.077	0.35674676	0.78829694	1	1257	tags=69%, lis
HALLMARK_ADIPOGENESIS	53	0.181	1.076	0.35283688	0.78914386	1	1088	tags=58%, lis
HP_ABNORMALITY_OF_VISION	161	0.140	1.075	0.32879046	0.7904496	1	731	tags=35%, lis
GOCC_CYTOPLASMIC_STRESS_GRANULE	27	0.224	1.075	0.3538175	0.78963673	1	879	tags=52%, lis
GOBP_STRESS_FIBER_ASSEMBLY	21	0.255	1.074	0.38973385	0.7899189	1	294	tags=29%, lis
HP_FREQUENT_FALLS	16	0.276	1.074	0.3654224	0.78947175	1	725	tags=50%, lis
GOBP_VESICLE_TARGETING	25	0.239	1.074	0.36920223	0.78863287	1	474	tags=32%, lis
GOBP_PROTEIN_STABILIZATION	42	0.195	1.072	0.35828876	0.79316825	1	433	tags=26%, lis
GOBP_RESPONSE_TO_ABIOTIC_STIMULUS	222	0.132	1.071	0.31949458	0.7928607	1	617	tags=30%, lis
GOBP_REGULATION_OF_PH	19	0.262	1.070	0.36923078	0.7941559	1	729	tags=47%, lis
HP_ABNORMAL_GLUCOSE_HOMEOSTASIS	70	0.173	1.070	0.37272727	0.79382324	1	956	tags=53%, lis
GOBP_CELLULAR_RESPONSE_TO_VIRUS	22	0.241	1.070	0.35673624	0.79355234	1	536	tags=36%, lis
REACTOME_SIGNALING_BY_VEGF	27	0.227	1.069	0.38376385	0.79321873	1	438	tags=30%, lis
GOBP_NOTCH_SIGNALING_PATHWAY	29	0.220	1.069	0.36802974	0.79220116	1	450	tags=28%, lis
GOBP_REGULATION_OF_PEPTIDE_HORMONE_SECRETION	29	0.218	1.069	0.37200737	0.7914509	1	635	tags=41%, lis
GOBP_ENDOPLASMIC_RETICULUM_UNFOLDED_PROTEIN_RESPONSE	40	0.197	1.068	0.3764045	0.7928217	1	445	tags=28%, lis
HP_HIGH_FOREHEAD	51	0.182	1.068	0.36923078	0.79184407	1	337	tags=24%, lis
GOBP_POSITIVE_REGULATION_OF_LEUKOCYTE_PROLIFERATION	26	0.234	1.068	0.3880597	0.7918268	1	720	tags=46%, lis
GOBP_CANONICAL_WNT_SIGNALING_PATHWAY	65	0.172	1.067	0.36823106	0.79220426	1	592	tags=34%, lis
HP_CLEFT_SOFT_PALATE	19	0.256	1.067	0.3752345	0.7919186	1	217	tags=21%, lis
HP_DUPLICATION_OF_HAND_BONES	20	0.251	1.065	0.3759542	0.79543394	1	951	tags=60%, lis
GOBP_POSITIVE_REGULATION_OF_PLASMA_MEMBRANE_BOUNDED_CELL_PROJECTION_ASSEMBL	22	0.244	1.064	0.38931298	0.7955279	1	224	tags=23%, lis
GOBP_DNA_BIOSYNTHETIC_PROCESS	38	0.202	1.063	0.38059703	0.79855424	1	461	tags=29%, lis
HP_CHRONIC_CONSTIPATION	15	0.276	1.062	0.37695312	0.79852307	1	719	tags=47%, lis
GOBP_EPITHELIUM_DEVELOPMENT	130	0.145	1.062	0.33568904	0.7973856	1	983	tags=52%, lis
GOCC_TERTIARY_GRANULE_MEMBRANE	20	0.251	1.061	0.3864469	0.80027574	1	558	tags=35%, lis
GOBP_PROTEIN_EXIT_FROM_ENDOPLASMIC_RETICULUM	15	0.275	1.060	0.37430167	0.80016345	1	237	tags=27%, lis
GOBP_NUCLEAR_CHROMOSOME_SEGREGATION	31	0.216	1.060	0.38112524	0.7997482	1	649	tags=39%, lis
HP_CHILDHOOD_ONSET	17	0.261	1.060	0.37383178	0.8001109	1	292	tags=29%, lis
HP_SPECIFIC_LEARNING_DISABILITY	30	0.213	1.059	0.392	0.80152136	1	300	tags=23%, lis
REACTOME_INFECTIOUS_DISEASE	160	0.139	1.058	0.35304055	0.8007838	1	619	tags=31%, lis
GOBP_MULTICELLULAR_ORGANISM_GROWTH	28	0.221	1.058	0.37918872	0.8007231	1	420	tags=32%, lis
GOBP_ERYTHROCYTE_HOMEOSTASIS	32	0.209	1.058	0.38745388	0.7997501	1	982	tags=56%, lis
REACTOME_HCMV_EARLY_EVENTS	15	0.275	1.058	0.38262478	0.7997452	1	411	tags=33%, lis
KEGG_LEISHMANIA_INFECTION	24	0.233	1.057	0.36555362	0.7990352	1	617	tags=38%, lis
GOBP_NEGATIVE_REGULATION_OF_CELLULAR_CATABOLIC_PROCESS	38	0.200	1.057	0.39037433	0.8002086	1	617	tags=39%, lis
HP_KYPHOSCOLIOSIS	20	0.244	1.056	0.36074767	0.7994128	1	542	tags=40%, lis
HP_ABNORMALITY_OF_CENTRAL_NERVOUS_SYSTEM_ELECTROPHYSIOLOGY	75	0.165	1.056	0.35636362	0.80025834	1	694	tags=39%, lis
GOBP_INTRINSIC_APOPTOTIC_SIGNALING_PATHWAY	74	0.164	1.056	0.37985864	0.79912156	1	213	tags=14%, lis
GOBP_PROTEIN_CATABOLIC_PROCESS	217	0.130	1.056	0.36408567	0.79834193	1	618	tags=31%, lis
HP_ABNORMAL_GLIAL_CELL_MORPHOLOGY	27	0.220	1.055	0.38838476	0.7981501	1	893	tags=56%, lis
HALLMARK_UNFOLDED_PROTEIN_RESPONSE	27	0.226	1.055	0.3850365	0.7979033	1	838	tags=48%, lis
GOBP_NCRNA_METABOLIC_PROCESS	53	0.182	1.053	0.36445242	0.8004215	1	963	tags=57%, lis

REACTOME_CELL_CYCLE_MITOTIC	90	0.158	1.053	0.37683824	0.80091715	1	720	tags=38%, lis
GOBP_REGULATION_OF_CELL_DIVISION	22	0.239	1.053	0.38709676	0.8005717	1	804	tags=50%, lis
GOBP_CELLULAR_RESPONSE_TO_STARVATION	31	0.210	1.052	0.37964776	0.8015042	1	411	tags=26%, lis
HP_CORTICAL_DYSPLASIA	16	0.267	1.052	0.3988658	0.8004097	1	999	tags=69%, lis
HP_RECURRENT_UPPER_RESPIRATORY_TRACT_INFECTIONS	24	0.235	1.051	0.39285713	0.80102694	1	476	tags=29%, lis
GOBP_REACTIVE_OXYGEN_SPECIES_METABOLIC_PROCESS	61	0.170	1.051	0.39114392	0.8001525	1	195	tags=15%, lis
GOBP_REGULATION_OF_CELLULAR_RESPONSE_TO_HEAT	16	0.269	1.051	0.3841912	0.8001165	1	411	tags=31%, lis
HALLMARK_MTORC1_SIGNALING	55	0.180	1.049	0.37545127	0.803569	1	686	tags=40%, lis
GOBP_SIGNAL_TRANSDUCTION_IN_ABSENCE_OF_LIGAND	15	0.281	1.049	0.3937729	0.80258334	1	698	tags=47%, lis
HP_ABNORMALITY_OF_THE_RIBS	35	0.202	1.049	0.3820034	0.8015631	1	300	tags=23%, lis
REACTOME_PI3K_AKT_SIGNALING_IN_CANCER	20	0.246	1.049	0.3897059	0.80102277	1	283	tags=25%, lis
GOBP_REGULATION_OF_REACTIVE_OXYGEN_SPECIES_METABOLIC_PROCESS	40	0.199	1.048	0.38181818	0.80111617	1	662	tags=38%, lis
GOBP_DEVELOPMENT_OF_PRIMARY_FEMALE_SEXUAL_CHARACTERISTICS	19	0.253	1.048	0.39439252	0.8000704	1	1067	tags=63%, lis
HP_EMOTIONAL_LABILITY	27	0.219	1.047	0.3846154	0.8027629	1	1257	tags=74%, lis
GOBP_CELLULAR_MONOVALENT_INORGANIC_CATION_HOMEOSTASIS	22	0.240	1.046	0.38560885	0.8043359	1	729	tags=45%, lis
GOBP_RESPONSE_TO_TEMPERATURE_STIMULUS	43	0.194	1.046	0.38754326	0.8033123	1	481	tags=30%, lis
HP_BRACHYDACTYLY	54	0.177	1.045	0.38324422	0.8032443	1	807	tags=48%, lis
HP_ABNORMALITY_OF_THE_CEREBRAL_VASCULATURE	49	0.181	1.045	0.39888683	0.8035947	1	286	tags=20%, lis
GOBP_CELLULAR_RESPONSE_TO_TOPOLOGICALLY_INCORRECT_PROTEIN	53	0.182	1.044	0.39826086	0.8035253	1	445	tags=26%, lis
GOBP_POSITIVE_REGULATION_OF_VIRAL_PROCESS	22	0.230	1.044	0.41621622	0.8029481	1	561	tags=36%, lis
GOBP_PROTEIN_PROCESSING	25	0.225	1.044	0.40636703	0.8030642	1	9	tags=8%, list-
GOCC_UBIQUITIN_LIGASE_COMPLEX	73	0.164	1.043	0.40145984	0.80282766	1	404	tags=23%, lis
PID_CXCR4_PATHWAY	26	0.221	1.042	0.40515652	0.8058819	1	224	tags=19%, lis
GOBP_SULFUR_COMPOUND_BIOSYNTHETIC_PROCESS	36	0.201	1.042	0.41058394	0.80534714	1	660	tags=36%, lis
GOBP_UBIQUITIN_DEPENDENT_ERAD_PATHWAY	20	0.245	1.041	0.40712947	0.80459446	1	637	tags=40%, lis
GOCC_TRANS_GOLGI_NETWORK	53	0.179	1.041	0.39181286	0.80475324	1	520	tags=28%, lis
GOBP_REGULATION_OF_REACTIVE_OXYGEN_SPECIES_BIOSYNTHETIC_PROCESS	19	0.256	1.041	0.40402195	0.8043403	1	506	tags=37%, lis
HP_ABNORMAL_VASCULAR_MORPHOLOGY	112	0.146	1.039	0.4128114	0.8081817	1	817	tags=42%, lis
GOCC_CELL_SURFACE	87	0.156	1.039	0.39963832	0.80738425	1	568	tags=33%, lis
REACTOME_CELLULAR_RESPONSES_TO_EXTERNAL_STIMULI	143	0.137	1.038	0.40535715	0.8077434	1	499	tags=24%, lis
GOBP_PROTEIN_LOCALIZATION_TO_ORGANELLE	185	0.131	1.038	0.39688042	0.80674845	1	487	tags=24%, lis
HP_ABNORMAL_CIRCULATING_METABOLITE_CONCENTRATION	178	0.132	1.037	0.40036902	0.8082934	1	902	tags=46%, lis
HP_DECREASED_FACIAL_EXPRESSION	26	0.223	1.035	0.41604477	0.81364286	1	523	tags=35%, lis
HP_URINARY_INCONTINENCE	19	0.252	1.034	0.42992425	0.81510323	1	224	tags=26%, lis
HP_DIMINISHED_MOTIVATION	21	0.238	1.033	0.41219962	0.8158514	1	434	tags=29%, lis
HP_RESPIRATORY_TRACT_INFECTION	99	0.147	1.032	0.39718804	0.8173024	1	476	tags=23%, lis
REACTOME_FACTORS_INVOLVED_IN_MEGAKARYOCYTE_DEVELOPMENT_AND_PLATELET_PRODUCT	23	0.232	1.032	0.4227941	0.8166199	1	258	tags=22%, lis
GOBP_UNSATURATED_FATTY_ACID_METABOLIC_PROCESS	16	0.260	1.032	0.39332098	0.81566644	1	92	tags=19%, lis
HP_ABNORMAL_INVOLUNTARY_EYE_MOVEMENTS	144	0.138	1.032	0.42358804	0.8146121	1	831	tags=43%, lis
HP_ABNORMAL_METACARPAL_MORPHOLOGY	27	0.215	1.031	0.40223464	0.8140006	1	89	tags=15%, lis
GOBP_POSITIVE_REGULATION_OF_ACTIN_FILAMENT_POLYMERIZATION	18	0.251	1.031	0.4126394	0.813128	1	1072	tags=67%, lis
GOBP_CELLULAR_RESPONSE_TO_DNA_DAMAGE_STIMULUS	157	0.134	1.031	0.40140846	0.81232166	1	189	tags=12%, lis
GOBP_LEUKOCYTE_MIGRATION	74	0.161	1.031	0.40373832	0.8119935	1	290	tags=19%, lis
REACTOME_CLASS_I_MHC_MEDIATED_ANTIGEN_PROCESSING_PRESENTATION	107	0.147	1.030	0.4283088	0.81438327	1	503	tags=26%, lis
PID_ERBB1_DOWNSTREAM_PATHWAY	29	0.211	1.028	0.4270073	0.81694925	1	224	tags=21%, lis
HP_ABNORMAL_THORAX_MORPHOLOGY	123	0.142	1.027	0.42300886	0.8200571	1	828	tags=44%, lis
GOBP_RESPONSE_TO_ORGANIC_CYCLIC_COMPOUND	135	0.139	1.025	0.44049734	0.82426244	1	786	tags=40%, lis
HP_DEPRESSED_NASAL_RIDGE	15	0.275	1.024	0.44545454	0.8252126	1	138	tags=20%, lis
HP_NEVUS	15	0.270	1.023	0.4015444	0.8268355	1	345	tags=33%, lis
REACTOME_METABOLISM_OF_RNA	133	0.138	1.023	0.42611682	0.8260456	1	975	tags=50%, lis
HP_HEMOLYTIC_ANEMIA	27	0.218	1.022	0.42560554	0.82773393	1	521	tags=33%, lis
GOMF_UBIQUITIN_LIKE_PROTEIN_BINDING	28	0.216	1.021	0.4227941	0.8280561	1	1053	tags=61%, lis
GOMF_AMYLOID_BETA_BINDING	17	0.251	1.021	0.42857143	0.827661	1	420	tags=35%, lis
GOCC_LYSOSOMAL_LUMEN	17	0.258	1.020	0.42992425	0.8285006	1	709	tags=47%, lis
HP_ABNORMAL_MITRAL_VALVE_PHYSIOLOGY	18	0.244	1.020	0.41869158	0.82805896	1	557	tags=39%, lis
HP_OSTEOMYELITIS	19	0.245	1.019	0.42230347	0.8289203	1	662	tags=42%, lis
GOBP_B_CELL_MEDIATED_IMMUNITY	21	0.237	1.018	0.41712204	0.8296526	1	237	tags=24%, lis
HP_REDUCED_VISUAL_ACUITY	60	0.171	1.018	0.44878957	0.8287319	1	618	tags=33%, lis
GOBP_ACTIN_FILAMENT_BUNDLE_ORGANIZATION	33	0.203	1.018	0.4283019	0.8285038	1	504	tags=30%, lis
GOMF_ACTIVE_TRANSMEMBRANE_TRANSPORTER_ACTIVITY	22	0.231	1.018	0.45421246	0.8279465	1	381	tags=27%, lis
GOBP_ALPHA_BETA_T_CELL_ACTIVATION	31	0.204	1.017	0.44285715	0.82769066	1	293	tags=23%, lis
HP_SLEEP_APNEA	18	0.246	1.017	0.43843284	0.8278079	1	1064	tags=72%, lis
REACTOME_NEDDYLATION	58	0.169	1.016	0.4368231	0.8280868	1	615	tags=34%, lis
HP_APLASIA_HYPOPLASIA_OF_THE_CEREBELLUM	45	0.182	1.016	0.4519774	0.82819486	1	224	tags=18%, lis
HP_DEPRESSED_NASAL_BRIDGE	70	0.158	1.016	0.42201835	0.82820266	1	724	tags=39%, lis
PID_P75_NTR_PATHWAY	18	0.249	1.015	0.40252706	0.8274779	1	901	tags=61%, lis
GOBP_LEUKOCYTE_APOPTOTIC_PROCESS	20	0.239	1.013	0.42572463	0.83198977	1	720	tags=50%, lis
GOBP_POSITIVE_REGULATION_OF_PATTERN_RECOGNITION_RECEPTOR_SIGNALING_PATHWAY	15	0.269	1.013	0.4446565	0.8310757	1	568	tags=47%, lis
GOCC_CELL_LEADING_EDGE	72	0.161	1.013	0.4364641	0.83071584	1	589	tags=32%, lis
GOBP_ACTIVATION_OF_CYSTEINE_TYPE_ENDOPEPTIDASE_ACTIVITY_INVOLVED_IN_APOPTOTIC_PR	18	0.247	1.012	0.44741875	0.8316544	1	1089	tags=67%, lis
GOBP_REGULATION_OF_PEPTIDYL_TYROSINE_PHOSPHORYLATION	46	0.182	1.012	0.44241315	0.8309726	1	237	tags=20%, lis
GOBP_REGULATION_OF_DENDRITE_DEVELOPMENT	15	0.266	1.012	0.4456929	0.8303593	1	301	tags=27%, lis
GOCC_ENDOSOME_MEMBRANE	95	0.147	1.012	0.42805755	0.8297562	1	1046	tags=56%, lis
REACTOME_REGULATED_NECROSIS	19	0.245	1.011	0.4173077	0.82974106	1	901	tags=58%, lis
GOBP_SUPEROXIDE_METABOLIC_PROCESS	16	0.252	1.011	0.45864663	0.8298006	1	619	tags=38%, lis
GOBP_POSITIVE_REGULATION_OF_MRNA_CATABOLIC_PROCESS	24	0.225	1.011	0.42829075	0.8290369	1	398	tags=29%, lis
GOBP_PROTEIN_DEPHOSPHORYLATION	60	0.172	1.011	0.44464946	0.8283288	1	473	tags=27%, lis
GOBP_CELLULAR_RESPONSE_TO_KETONE	19	0.243	1.010	0.44954127	0.82779413	1	540	tags=42%, lis
GOBP_HORMONE_TRANSPORT	43	0.182	1.010	0.43603605	0.8274251	1	761	tags=44%, lis

HP_ABNORMALITY_OF_THE_LYMPH_NODES	52	0.171	1.010	0.45272726	0.8264877	1	617	tags=35%, lis
HP_ABNORMAL_MITRAL_VALVE_MORPHOLOGY	18	0.244	1.010	0.45242718	0.8256401	1	461	tags=33%, lis
GOBP_POSITIVE_REGULATION_OF_RESPONSE_TO_DNA_DAMAGE_STIMULUS	16	0.256	1.009	0.43664718	0.8264381	1	359	tags=31%, lis
HP_ECTOPIC_CALCIFICATION	28	0.208	1.009	0.44186047	0.82602745	1	314	tags=21%, lis
HP_PHENOTYPIC_VARIABILITY	51	0.179	1.009	0.43872115	0.825006	1	523	tags=31%, lis
GOBP_RESPONSE_TO_ACID_CHEMICAL	24	0.223	1.008	0.45351043	0.8272273	1	499	tags=33%, lis
HP_ABNORMAL_AUTONOMIC_NERVOUS_SYSTEM_PHYSIOLOGY	20	0.241	1.007	0.4351852	0.82772994	1	80	tags=15%, lis
HP_GAIT_DISTURBANCE	173	0.128	1.006	0.44901314	0.8280761	1	864	tags=43%, lis
GOBP_AUTOPHAGOSOME_ORGANIZATION	20	0.235	1.006	0.433526	0.82789606	1	33	tags=15%, lis
GOCC_CELL_PROJECTION_MEMBRANE	41	0.184	1.006	0.4470135	0.8278976	1	490	tags=29%, lis
GOBP_REGULATION_OF_SUPRAMOLECULAR_FIBER_ORGANIZATION	74	0.158	1.005	0.46605504	0.82834744	1	340	tags=22%, lis
HP_ABSENT_SEPTUM_PELLUCIDUM	18	0.250	1.005	0.4678899	0.8282836	1	675	tags=44%, lis
GOBP_NEPHRON_DEVELOPMENT	15	0.264	1.005	0.45564517	0.82743186	1	359	tags=33%, lis
GOBP_REGULATION_OF_ACTIN_FILAMENT_BUNDLE_ASSEMBLY	20	0.240	1.005	0.46138996	0.82641387	1	290	tags=25%, lis
GOCC_AZUROPHIL_GRANULE	48	0.174	1.005	0.45210728	0.8256822	1	442	tags=25%, lis
HP_ABNORMALITY_OF_COORDINATION	156	0.132	1.004	0.4658494	0.82508355	1	899	tags=44%, lis
REACTOME_RAC2_GTPASE_CYCLE	31	0.202	1.003	0.4466403	0.8263542	1	91	tags=13%, lis
GOBP_NEGATIVE_REGULATION_OF_DEFENSE_RESPONSE	43	0.184	1.003	0.44894028	0.8269202	1	562	tags=33%, lis
GOBP_LAMELLIPODIUM_ORGANIZATION	17	0.247	1.002	0.44970414	0.82836586	1	224	tags=24%, lis
HP_BRUISING_SUSCEPTIBILITY	24	0.223	1.001	0.44869214	0.8293519	1	138	tags=17%, lis
GOCC_ACTIN_CYTOSKELETON	65	0.162	1.001	0.4502814	0.8287053	1	686	tags=35%, lis
GOBP_NEGATIVE_REGULATION_OF_DEPHOSPHORYLATION	25	0.219	1.001	0.45557657	0.8283776	1	462	tags=32%, lis
REACTOME_INTERLEUKIN_12_SIGNALING	16	0.257	1.001	0.46252286	0.82738155	1	130	tags=19%, lis
KEGG_TOLL_LIKE_RECEPTOR_SIGNALING_PATHWAY	19	0.234	1.000	0.44878957	0.8276205	1	617	tags=37%, lis
GOBP_MYELOID_LEUKOCYTE_DIFFERENTIATION	43	0.185	0.999	0.47297296	0.82807773	1	275	tags=19%, lis
HP_ABNORMALITY_OF_THE_PITUITARY_GLAND	31	0.200	0.999	0.47513813	0.8273497	1	293	tags=23%, lis
GOBP_ORGANONITROGEN_COMPOUND_CATABOLIC_PROCESS	252	0.119	0.998	0.46678025	0.82942986	1	618	tags=30%, lis
GOBP_SKIN_DEVELOPMENT	21	0.229	0.998	0.46168223	0.82848895	1	957	tags=57%, lis
GOBP_CYTOSKELETON_DEPENDENT_CYTOKINESIS	15	0.268	0.997	0.46629214	0.82917964	1	1024	tags=67%, lis
HP_ABNORMALITY_OF_FACIAL_SOFT_TISSUE	65	0.161	0.997	0.46551725	0.8302019	1	725	tags=38%, lis
HP_ABNORMALITY_OF_THE_SKULL_BASE	29	0.205	0.997	0.4504673	0.829251	1	670	tags=38%, lis
GOBP_POSITIVE_REGULATION_OF_EPITHELIAL_CELL_PROLIFERATION	20	0.228	0.994	0.4384058	0.8334724	1	729	tags=50%, lis
HP_TUBE_FEEDING	40	0.184	0.993	0.45864663	0.83498406	1	620	tags=35%, lis
HALLMARK_APOPTOSIS	49	0.171	0.993	0.4446461	0.8339976	1	335	tags=22%, lis
HP_POLYDACTYLY	24	0.226	0.993	0.45525292	0.833914	1	933	tags=54%, lis
GOBP_NEURAL_PRECURSOR_CELL_PROLIFERATION	15	0.264	0.992	0.48168498	0.8341367	1	1215	tags=73%, lis
GOBP_REGULATION_OF_LEUKOCYTE_APOPTOTIC_PROCESS	15	0.260	0.992	0.4517454	0.83319503	1	534	tags=40%, lis
HP_VISUAL_IMPAIRMENT	138	0.134	0.992	0.51365185	0.8322859	1	731	tags=36%, lis
GOBP_REGULATION_OF_HORMONE_LEVELS	53	0.168	0.992	0.47803164	0.83134115	1	763	tags=43%, lis
GOBP_ESTABLISHMENT_OF_PROTEIN_LOCALIZATION_TO_ORGANELLE	110	0.143	0.992	0.46061644	0.832518	1	744	tags=38%, lis
GOBP_MACROAUTOPHAGY	69	0.155	0.991	0.47897622	0.8329993	1	964	tags=52%, lis
GOBP_DEPHOSPHORYLATION	79	0.151	0.989	0.4733096	0.83591	1	473	tags=25%, lis
GOBP_VESICLE_LOCALIZATION	38	0.191	0.988	0.4883721	0.8378388	1	474	tags=26%, lis
HP_ABNORMAL_ADIPOSE_TISSUE_MORPHOLOGY	33	0.196	0.988	0.47522935	0.83763707	1	710	tags=48%, lis
GOBP_CELL_CELL_ADHESION	112	0.139	0.987	0.4831261	0.8376056	1	500	tags=26%, lis
GOBP_POSITIVE_REGULATION_OF_SMALL_GTPASE_MEDIATED_SIGNAL_TRANSDUCTION	16	0.255	0.987	0.4831014	0.8373226	1	720	tags=44%, lis
GOBP_TOLL_LIKE_RECEPTOR_SIGNALING_PATHWAY	45	0.178	0.987	0.47894737	0.8365182	1	910	tags=49%, lis
GOBP_HOMEOSTASIS_OF_NUMBER_OF_CELLS	55	0.166	0.986	0.48154658	0.8389299	1	984	tags=51%, lis
HP_PANCYTOPENIA	23	0.228	0.985	0.4738806	0.83921856	1	1871	tags=100%, l
HP_ABNORMALITY_OF_LATERAL_VENTRICLE	19	0.230	0.985	0.46930692	0.8394666	1	279	tags=21%, lis
GOBP_VESICLE_ORGANIZATION	69	0.158	0.983	0.4755245	0.8417292	1	619	tags=32%, lis
REACTOME_SENSORY_PERCEPTION	16	0.246	0.983	0.49716446	0.8409502	1	251	tags=25%, lis
GOBP_POSITIVE_REGULATION_OF_ERK1_AND_ERK2_CASCADE	25	0.211	0.982	0.51263535	0.84332377	1	201	tags=20%, lis
GOBP_PROTEIN_TARGETING	75	0.155	0.981	0.5109489	0.8433144	1	1079	tags=56%, lis
GOBP_MYELOID_CELL_HOMEOSTASIS	37	0.191	0.981	0.4805915	0.8430528	1	927	tags=51%, lis
GOBP_CHAPERONE_MEDIATED_PROTEIN_FOLDING	20	0.231	0.980	0.4814815	0.8438966	1	637	tags=40%, lis
HALLMARK_INFLAMMATORY_RESPONSE	44	0.181	0.980	0.48833033	0.8429577	1	644	tags=39%, lis
GOBP_POSITIVE_REGULATION_OF_MITOTIC_CELL_CYCLE	21	0.228	0.978	0.46743295	0.84653527	1	674	tags=43%, lis
HALLMARK_UV_RESPONSE_UP	36	0.187	0.978	0.4848485	0.8466776	1	796	tags=50%, lis
GOBP_REGULATION_OF_CELL_PROJECTION_ASSEMBLY	32	0.198	0.978	0.46125463	0.84638584	1	224	tags=19%, lis
HP_ADULT_ONSET	39	0.182	0.977	0.47743055	0.8459906	1	303	tags=21%, lis
HP_PROTRUDING_EAR	27	0.208	0.977	0.47663552	0.8451384	1	523	tags=33%, lis
GOCC_ORGANELLE_ENVELOPE_LUMEN	26	0.207	0.975	0.503876	0.8490633	1	744	tags=42%, lis
GOBP_MICROTUBULE_CYTOSKELETON_ORGANIZATION_INVOLVED_IN_MITOSIS	25	0.214	0.975	0.4725898	0.84931	1	567	tags=32%, lis
GOBP_MITOCHONDRIAL_GENE_EXPRESSION	15	0.258	0.975	0.47519085	0.848594	1	1174	tags=80%, lis
HP_HAND_POLYDACTYLY	17	0.242	0.974	0.475	0.84940946	1	933	tags=59%, lis
HP_HYPOTELORISM	19	0.234	0.973	0.48880598	0.8499476	1	711	tags=47%, lis
HP_ABNORMAL_GROWTH_HORMONE_LEVEL	19	0.235	0.973	0.46906188	0.8490085	1	245	tags=21%, lis
HP_ONSET	183	0.124	0.973	0.5280136	0.84902954	1	793	tags=38%, lis
GOBP_NEGATIVE_REGULATION_OF_CELL_ADHESION	54	0.165	0.973	0.5115864	0.8483109	1	258	tags=17%, lis
GOBP_REGULATION_OF_SMALL_MOLECULE_METABOLIC_PROCESS	74	0.154	0.972	0.4912892	0.8488488	1	670	tags=38%, lis
HP_ABNORMALITY_OF_THE_ADRENAL_GLANDS	23	0.216	0.972	0.5074627	0.8481022	1	807	tags=52%, lis
GOBP_ENDOTHELIUM_DEVELOPMENT	20	0.229	0.971	0.509542	0.8497451	1	635	tags=40%, lis
GOBP_NEGATIVE_REGULATION_OF_CELL_ACTIVATION	44	0.174	0.971	0.49818182	0.84994966	1	258	tags=18%, lis
GOBP_NEGATIVE_REGULATION_OF_PEPTIDYL_TYROSINE_PHOSPHORYLATION	16	0.252	0.970	0.4820416	0.85044277	1	227	tags=25%, lis
HP_SPASTIC_TETRAPLEGIA	17	0.245	0.969	0.4888889	0.8512192	1	292	tags=24%, lis
HP_APLASIA_HYPOPLASIA_OF_THE_CEREBELLAR_VERMIS	30	0.194	0.969	0.49737304	0.850579	1	612	tags=37%, lis
GOBP_REGULATION_OF_LIPID_METABOLIC_PROCESS	58	0.164	0.969	0.5062389	0.8506021	1	531	tags=29%, lis
GOBP_NEGATIVE_REGULATION_OF_INTRACELLULAR_SIGNAL_TRANSDUCTION	99	0.141	0.968	0.5149385	0.8513832	1	213	tags=14%, lis

GOBP_RESPONSE_TO_RADIATION	72	0.153	0.966	0.4652908	0.85409564	1	849	tags=44%, lis
GOBP_FC_RECEPTOR_MEDIATED_STIMULATORY_SIGNALING_PATHWAY	23	0.212	0.966	0.51224107	0.85440457	1	720	tags=43%, lis
GOMF_PHOSPHATIDYLINOSITOL_PHOSPHATE_BINDING	27	0.206	0.965	0.49818182	0.8548859	1	589	tags=37%, lis
HP_SKELETAL_DYSPLASIA	25	0.210	0.964	0.49909255	0.8565331	1	245	tags=20%, lis
GOBP_RESPONSE_TO_NUTRIENT	20	0.226	0.963	0.47328246	0.85773057	1	610	tags=40%, lis
GOBP_REGULATION_OF_HORMONE_SECRETION	34	0.191	0.963	0.48632812	0.8568043	1	728	tags=44%, lis
GOBP_CELL_CYCLE_PHASE_TRANSITION	119	0.133	0.962	0.5143885	0.85880524	1	682	tags=35%, lis
GOBP_ENDOPLASMIC_RETICULUM_TO_GOLGI_VESICLE_MEDIATED_TRANSPORT	49	0.170	0.962	0.50729924	0.85878843	1	516	tags=29%, lis
GOBP_BIOMINERALIZATION	18	0.232	0.962	0.5045872	0.8579677	1	499	tags=39%, lis
GOCC_SPECIFIC_GRANULE_MEMBRANE	24	0.208	0.960	0.49803922	0.8612635	1	139	tags=17%, lis
GOBP_GENE_SILENCING	50	0.169	0.959	0.5072727	0.8613057	1	415	tags=26%, lis
GOBP_ESTABLISHMENT_OR_MAINTENANCE_OF_CELL_POLARITY	34	0.189	0.959	0.49910232	0.8611953	1	294	tags=21%, lis
HP_ABNORMAL_MYELINATION	97	0.138	0.959	0.525	0.86100394	1	991	tags=51%, lis
GOBP_PATTERN_RECOGNITION_RECEPTOR_SIGNALING_PATHWAY	69	0.151	0.958	0.53954303	0.86105376	1	568	tags=30%, lis
GOBP_PROTEIN_KINASE_B_SIGNALING	41	0.179	0.958	0.5067437	0.8605895	1	473	tags=29%, lis
GOBP_GOLGI_VESICLE_TRANSPORT	87	0.142	0.958	0.5246801	0.859819	1	520	tags=25%, lis
GOBP_CELLULAR_RESPONSE_TO_EXTERNAL_STIMULUS	52	0.165	0.958	0.52327746	0.8594341	1	844	tags=46%, lis
GOBP_NEGATIVE_REGULATION_OF_PROTEIN_MODIFICATION_BY_SMALL_PROTEIN_CONJUGATION	23	0.217	0.957	0.5185185	0.8607847	1	683	tags=39%, lis
GOMF_RIBONUCLEOTIDE_BINDING	311	0.110	0.956	0.553018	0.86153215	1	393	tags=18%, lis
HP_SMALL_FOR_GESTATIONAL_AGE	34	0.189	0.956	0.5053381	0.8614063	1	477	tags=29%, lis
GOBP_VASCULAR_ENDOTHELIAL_GROWTH_FACTOR_RECEPTOR_SIGNALING_PATHWAY	20	0.232	0.954	0.4906367	0.8632093	1	609	tags=40%, lis
HP_PSYCHOSIS	17	0.236	0.954	0.51445085	0.8627796	1	779	tags=47%, lis
HP_ABNORMAL_CIRCULATING_CARBOXYLIC_ACID_CONCENTRATION	35	0.187	0.954	0.518797	0.8628721	1	855	tags=46%, lis
HP_NEURODEGENERATION	21	0.227	0.953	0.51724136	0.86313516	1	1874	tags=100%, l
GOBP_POSITIVE_REGULATION_OF_PROTEIN_LOCALIZATION_TO_NUCLEUS	19	0.227	0.952	0.49717513	0.86490506	1	392	tags=26%, lis
GOBP_SIGNAL_TRANSDUCTION_BY_P53_CLASS_MEDIATOR	45	0.167	0.951	0.532567	0.86546266	1	327	tags=18%, lis
KEGG_HUNTINGTONS_DISEASE	60	0.158	0.951	0.5368421	0.8648708	1	1257	tags=65%, lis
HP_DELAYED_ERUPTION_OF_TEETH	30	0.191	0.951	0.51291513	0.8639639	1	79	tags=13%, lis
HP_ABNORMAL_LEUKOCYTE_COUNT	67	0.152	0.950	0.5313059	0.8652073	1	489	tags=25%, lis
GOCC_ACTIN_FILAMENT	22	0.216	0.950	0.522686	0.8650621	1	294	tags=23%, lis
GOBP_REGULATION_OF_DNA_BINDING	27	0.194	0.950	0.5262206	0.8648361	1	450	tags=26%, lis
REACTOME_G_ALPHA_Q_SIGNALLING_EVENTS	20	0.226	0.950	0.51341	0.86397433	1	224	tags=20%, lis
GOBP_PEPTIDE_METABOLIC_PROCESS	133	0.130	0.949	0.55793226	0.8643605	1	540	tags=26%, lis
GOBP_CELLULAR_RESPONSE_TO_EXTRACELLULAR_STIMULUS	39	0.179	0.947	0.56746763	0.86718553	1	644	tags=36%, lis
GOBP_NECROTIC_CELL_DEATH	16	0.242	0.947	0.53246754	0.8679584	1	518	tags=38%, lis
GOBP_MEIOTIC_CELL_CYCLE	23	0.212	0.946	0.5303327	0.86761725	1	909	tags=57%, lis
HP_INABILITY_TO_WALK	38	0.180	0.946	0.52733684	0.8668027	1	554	tags=32%, lis
HP_ABNORMALITY_OF_NEURONAL_MIGRATION	67	0.152	0.946	0.56607145	0.8658694	1	933	tags=48%, lis
GOBP_REGULATION_OF_METAL_ION_TRANSPORT	29	0.200	0.946	0.51476014	0.8664292	1	897	tags=55%, lis
GOBP_CELLULAR_RESPONSE_TO_ABIOTIC_STIMULUS	54	0.160	0.945	0.5441696	0.8679542	1	359	tags=20%, lis
GOBP_CELLULAR_RESPONSE_TO_RADIATION	28	0.199	0.944	0.55675673	0.86751115	1	359	tags=21%, lis
GOMF_ELECTRON_TRANSFER_ACTIVITY	44	0.173	0.944	0.5444646	0.86679494	1	1419	tags=80%, lis
HP_ABNORMALITY_OF_THE_SENSE_OF_SMELL	16	0.236	0.944	0.5240741	0.8673716	1	893	tags=56%, lis
GOBP_TOR_SIGNALING	23	0.214	0.943	0.520354	0.86766446	1	411	tags=26%, lis
GOBP_GLIAL_CELL_DIFFERENTIATION	18	0.230	0.943	0.5192308	0.86738837	1	314	tags=28%, lis
HP_ABNORMALITY_OF_THE_COAGULATION_CASCADE	19	0.225	0.941	0.50823045	0.87036264	1	901	tags=58%, lis
REACTOME_NERVOUS_SYSTEM_DEVELOPMENT	72	0.147	0.941	0.5434381	0.8703914	1	489	tags=25%, lis
GOBP_GOLGI_ORGANIZATION	31	0.189	0.940	0.54626334	0.8704753	1	424	tags=29%, lis
HP_ABNORMAL_ERYTHROCYTE_MORPHOLOGY	113	0.134	0.940	0.5565371	0.87063897	1	854	tags=42%, lis
HP_GAIT_ATAXIA	29	0.195	0.939	0.55258125	0.8719285	1	461	tags=28%, lis
GOBP_FOREBRAIN_DEVELOPMENT	41	0.174	0.939	0.56294966	0.87119806	1	314	tags=20%, lis
HP_ABNORMALITY_OF_THE_SYNOVIA	35	0.180	0.937	0.54856116	0.8733335	1	138	tags=14%, lis
KEGG_ENDOCYTOSIS	30	0.192	0.937	0.56	0.8724364	1	607	tags=37%, lis
GOBP_REGULATION_OF_DEPHOSPHORYLATION	44	0.172	0.937	0.55315316	0.87251556	1	462	tags=27%, lis
GOMF_REPRESSING_TRANSCRIPTION_FACTOR_BINDING	15	0.245	0.935	0.5276753	0.8758989	1	24	tags=13%, lis
HP_CUTIS_LAXA	15	0.244	0.934	0.5304183	0.8775198	1	539	tags=40%, lis
GOBP_MITOTIC_CELL_CYCLE	181	0.118	0.933	0.57118356	0.87796575	1	682	tags=34%, lis
GOBP_REGULATION_OF_ANATOMICAL_STRUCTURE_SIZE	73	0.145	0.933	0.5650624	0.8775447	1	432	tags=23%, lis
GOBP_CELLULAR_MODIFIED_AMINO_ACID_METABOLIC_PROCESS	24	0.207	0.933	0.53271025	0.8779473	1	395	tags=29%, lis
REACTOME_AUTOPHAGY	27	0.197	0.932	0.5531136	0.8780916	1	561	tags=33%, lis
GOBP_NEURAL_TUBE_DEVELOPMENT	15	0.245	0.932	0.52327746	0.87783366	1	979	tags=60%, lis
HP_ABNORMAL_TONGUE_MORPHOLOGY	36	0.178	0.932	0.5478424	0.8773886	1	789	tags=44%, lis
GOBP_RESPONSE_TO_INTERLEUKIN_12	17	0.237	0.931	0.528827	0.87717164	1	130	tags=18%, lis
HP_ABNORMAL_SOCIAL_BEHAVIOR	36	0.178	0.931	0.5536723	0.8773302	1	595	tags=33%, lis
HP_PARKINSONISM	31	0.188	0.931	0.5537037	0.876772	1	80	tags=13%, lis
HP_POLYHYDRAMNIOS	20	0.216	0.931	0.5359116	0.87596965	1	807	tags=50%, lis
GOBP_ANIMAL_ORGAN_REGENERATION	15	0.243	0.930	0.53488374	0.87586176	1	705	tags=47%, lis
HP_MOTOR_SEIZURE	43	0.168	0.930	0.53577983	0.8752729	1	409	tags=26%, lis
HP_APLASIA_HYPOPLASIA_INVOLVING_THE_METACARPAL_BONES	23	0.204	0.930	0.5460993	0.8752853	1	79	tags=13%, lis
GOBP_POSITIVE_REGULATION_OF_TRANSPORT	148	0.122	0.929	0.59652174	0.87542504	1	638	tags=32%, lis
HP_GASTROINTESTINAL_INFLAMMATION	17	0.233	0.929	0.5559633	0.8752336	1	33	tags=12%, lis
GOCC_MEMBRANE_PROTEIN_COMPLEX	213	0.117	0.929	0.60271645	0.8744513	1	922	tags=45%, lis
HP_SHORT_ATTENTION_SPAN	69	0.150	0.928	0.55871886	0.8748628	1	245	tags=16%, lis
HP_SMOOTH_PHILTRUM	35	0.181	0.928	0.5490566	0.87492275	1	828	tags=49%, lis
REACTOME_CLATHRIN_MEDIATED_ENDOCYTOSIS	33	0.183	0.928	0.56989247	0.874023	1	569	tags=33%, lis
GOCC_TRANSFERASE_COMPLEX_TRANSFERRING_PHOSPHORUS_CONTAINING_GROUPS	48	0.159	0.928	0.57194245	0.8735702	1	510	tags=31%, lis
HP_DYSKINESIA	46	0.166	0.927	0.5825427	0.87323165	1	706	tags=37%, lis
GOBP_RESPONSE_TO_REACTIVE_OXYGEN_SPECIES	45	0.173	0.927	0.5576923	0.87326956	1	786	tags=42%, lis
GOBP_NEGATIVE_REGULATION_OF_SUPRAMOLECULAR_FIBER_ORGANIZATION	29	0.194	0.927	0.5441176	0.87287223	1	340	tags=24%, lis

GOBP_REGULATION_OF_ACTIN_FILAMENT_BASED_PROCESS	72	0.144	0.926	0.5516014	0.874327	1	290	tags=18%, lis
HP_ABNORMAL_FEMALE_REPRODUCTIVE_SYSTEM_PHYSIOLOGY	31	0.190	0.925	0.53406996	0.87402576	1	849	tags=52%, lis
HP_STROKE	21	0.216	0.925	0.5542857	0.8735892	1	198	tags=19%, lis
REACTOME_CELL_SURFACE_INTERACTIONS_AT_THE_VASCULAR_WALL	23	0.208	0.925	0.5584416	0.87278575	1	224	tags=22%, lis
HP_UNUSUAL_INFECTION	122	0.129	0.925	0.59930915	0.87227756	1	503	tags=24%, lis
KEGG_B_CELL_RECEPTOR_SIGNALING_PATHWAY	25	0.200	0.925	0.5410448	0.8714304	1	283	tags=24%, lis
HP_CEREBRAL_VISUAL_IMPAIRMENT	37	0.176	0.924	0.56653994	0.87201023	1	714	tags=38%, lis
HP_ABNORMALITY_OF_FACIAL_SKELETON	162	0.121	0.924	0.6135593	0.871782	1	816	tags=40%, lis
HALLMARK_PEROXISOME	19	0.220	0.922	0.55985266	0.87401056	1	917	tags=63%, lis
GOBP_RESPONSE_TO_LIGHT_STIMULUS	53	0.155	0.922	0.57410884	0.87389374	1	881	tags=49%, lis
GOBP_INTEGRIN_MEDIATED_SIGNALING_PATHWAY	19	0.224	0.922	0.56640625	0.8732091	1	500	tags=37%, lis
GOCC_CLATHRIN_COATED_VESICLE	40	0.169	0.921	0.5833333	0.87537766	1	441	tags=28%, lis
GOBP_NUCLEOSIDE_TRIPHOSPHATE_BIOSYNTHETIC_PROCESS	17	0.230	0.919	0.56962025	0.8769387	1	865	tags=59%, lis
PID_MYC_ACTIV_PATHWAY	25	0.199	0.918	0.55140185	0.8784662	1	924	tags=56%, lis
GOBP_CELLULAR_COMPONENT_DISASSEMBLY	99	0.133	0.918	0.58523726	0.87915784	1	568	tags=28%, lis
HP_MITOCHONDRIAL_MYOPATHY	16	0.231	0.917	0.5534351	0.8790113	1	899	tags=56%, lis
KEGG_NATURAL_KILLER_CELL_MEDIATED_CYTOTOXICITY	26	0.195	0.916	0.5708955	0.87982583	1	224	tags=19%, lis
HP_UNUSUAL_INFECTION_BY_ANATOMICAL_SITE	16	0.235	0.915	0.56296295	0.88122064	1	15	tags=13%, lis
GOBP_BIOLOGICAL_PROCESS_INVOLVED_IN_SYMBIOTIC_INTERACTION	239	0.109	0.915	0.6305085	0.8804966	1	540	tags=24%, lis
HP_DRY_SKIN	20	0.211	0.915	0.56949806	0.8795993	1	1181	tags=75%, lis
HP_GLUCOSE_INTOLERANCE	55	0.154	0.915	0.56183743	0.87899184	1	592	tags=33%, lis
HP_CRANIOSYNOSTOSIS	24	0.201	0.915	0.56537753	0.8791142	1	477	tags=33%, lis
HP_ABNORMAL_NEUTROPHIL_COUNT	36	0.173	0.915	0.58093524	0.8785107	1	281	tags=17%, lis
GOBP_RECOMBINATIONAL_REPAIR	15	0.240	0.914	0.5697446	0.8776759	1	764	tags=53%, lis
GOBP_REGULATION_OF_AUTOPHAGY	58	0.153	0.914	0.57404023	0.87688446	1	557	tags=28%, lis
GOBP_REGULATION_OF_CYCLIN_DEPENDENT_PROTEIN_KINASE_ACTIVITY	18	0.224	0.914	0.5721831	0.87614137	1	751	tags=50%, lis
HP_POSTNATAL_MICROCEPHALY	39	0.169	0.913	0.58455884	0.8783485	1	618	tags=33%, lis
HP_AUTOIMMUNE_ANTIBODY_POSITIVITY	18	0.225	0.912	0.5623836	0.87874264	1	313	tags=22%, lis
HALLMARK_GLYCOLYSIS	24	0.205	0.912	0.5878136	0.87785447	1	1088	tags=63%, lis
GOBP_NADH_DEHYDROGENASE_COMPLEX_ASSEMBLY	20	0.219	0.912	0.5777778	0.87794185	1	899	tags=50%, lis
PID_P53_DOWNSTREAM_PATHWAY	28	0.192	0.911	0.5559633	0.8794344	1	189	tags=14%, lis
REACTOME_COMPLEX_I_BIOGENESIS	20	0.219	0.910	0.59029126	0.8812898	1	899	tags=50%, lis
GOBP_RESPONSE_TO_MOLECULE_OF_BACTERIAL_ORIGIN	74	0.143	0.909	0.5988806	0.88238275	1	562	tags=30%, lis
GOBP_ADAPTIVE_IMMUNE_RESPONSE	77	0.141	0.908	0.5784832	0.88313806	1	311	tags=18%, lis
GOCC_GOLGI_APPARATUS	270	0.108	0.907	0.6395939	0.8841815	1	522	tags=23%, lis
GOCC_MICROTUBULE_ASSOCIATED_COMPLEX	16	0.232	0.906	0.5700576	0.8844926	1	770	tags=44%, lis
HP_ABNORMAL_SOFT_PALATE_MORPHOLOGY	23	0.204	0.906	0.58992803	0.8836571	1	619	tags=35%, lis
GOBP_PROTEIN_K63_LINKED_UBIQUITINATION	17	0.226	0.905	0.57471263	0.88564503	1	704	tags=41%, lis
GOBP_IMMUNE_SYSTEM_DEVELOPMENT	210	0.109	0.905	0.6579407	0.8853086	1	704	tags=32%, lis
GOBP_TELENCEPHALON_DEVELOPMENT	24	0.198	0.904	0.5857143	0.8851979	1	199	tags=17%, lis
HP_EPICANTHUS	74	0.145	0.903	0.601476	0.8864524	1	505	tags=26%, lis
REACTOME_FCGAMMA_RECEPTOR_FCGR_DEPENDENT_PHAGOCYTOSIS	20	0.208	0.903	0.58135283	0.8872124	1	224	tags=20%, lis
HP_RECURRENT_OTITIS_MEDIA	21	0.210	0.903	0.5659051	0.8863544	1	403	tags=29%, lis
REACTOME_METABOLISM_OF_WATER_SOLUBLE_VITAMINS_AND_COFACTORS	17	0.225	0.900	0.5685185	0.89139014	1	329	tags=24%, lis
HP_DYSTONIA	94	0.132	0.900	0.6372007	0.8912165	1	400	tags=19%, lis
GOBP_TISSUE_REMODELING	26	0.193	0.899	0.5939716	0.8924791	1	290	tags=23%, lis
GOBP_POSITIVE_REGULATION_OF_EXOCYTOSIS	16	0.229	0.898	0.59149724	0.89214176	1	607	tags=44%, lis
GOCC_COPII_COATED_ER_TO_GOLGI_TRANSPORT_VESICLE	22	0.201	0.898	0.5664207	0.8912503	1	516	tags=32%, lis
GOCC_CELL_BODY	73	0.141	0.898	0.61717355	0.8909263	1	398	tags=22%, lis
REACTOME_COPII_MEDIATED_VESICLE_TRANSPORT	19	0.215	0.898	0.61814743	0.89036334	1	474	tags=32%, lis
GOBP_REGULATION_OF_CELL_SUBSTRATE_JUNCTION_ORGANIZATION	16	0.228	0.898	0.5891182	0.8903639	1	290	tags=25%, lis
HP_ABNORMAL_CARDIAC_TEST	15	0.234	0.897	0.58925146	0.8896566	1	714	tags=47%, lis
GOBP_PROTEIN_AUTOUBIQUITINATION	18	0.217	0.897	0.5874263	0.8899474	1	311	tags=22%, lis
HP_RECURRENT_SKIN_INFECTIONS	19	0.214	0.897	0.5873016	0.8892087	1	28	tags=11%, lis
GOCC_NUCLEAR_MEMBRANE	58	0.151	0.897	0.6259124	0.88859606	1	443	tags=26%, lis
GOBP_REGULATION_OF_GENE_SILENCING_BY_RNA	20	0.205	0.896	0.59683096	0.8889759	1	411	tags=30%, lis
HP_SPASTICITY	153	0.116	0.896	0.6898305	0.88892275	1	1008	tags=48%, lis
GOBP_HEAD_DEVELOPMENT	90	0.134	0.896	0.63829786	0.88835585	1	990	tags=50%, lis
HP_ABNORMAL_THYROID_MORPHOLOGY	21	0.207	0.895	0.5763359	0.88874763	1	864	tags=52%, lis
GOBP_NEGATIVE_REGULATION_OF_BINDING	27	0.190	0.895	0.61714286	0.8881358	1	450	tags=26%, lis
GOBP_ORGANIC_ACID_CATABOLIC_PROCESS	21	0.208	0.895	0.58472997	0.88729614	1	917	tags=57%, lis
GOBP_SPINDLE_ORGANIZATION	32	0.182	0.894	0.5874525	0.8876072	1	621	tags=34%, lis
GOBP_REGULATION_OF_PEPTIDE_SECRETION	44	0.164	0.894	0.60254085	0.88762313	1	961	tags=55%, lis
HP_ABNORMALITY_OF_THE_SPLEEN	90	0.133	0.894	0.6533575	0.88680536	1	642	tags=33%, lis
HP_ABNORMAL_FACIAL_EXPRESSION	29	0.183	0.893	0.59322035	0.8882703	1	523	tags=31%, lis
HP_DYSPHAGIA	75	0.137	0.892	0.625	0.8881964	1	899	tags=43%, lis
HP_LIMB_HYPERTONIA	20	0.207	0.892	0.57751936	0.8889969	1	317	tags=25%, lis
HP_HYPOPITUITARISM	19	0.211	0.891	0.5936255	0.8882846	1	245	tags=21%, lis
HP_ABNORMAL_HEART_VALVE_MORPHOLOGY	41	0.166	0.891	0.60215056	0.8886451	1	817	tags=44%, lis
GOBP_NEGATIVE_REGULATION_OF_CELLULAR_COMPONENT_ORGANIZATION	125	0.120	0.889	0.6594595	0.89078903	1	518	tags=26%, lis
REACTOME_SIGNALING_BY_THE_B_CELL_RECEPTOR_BCR	41	0.166	0.889	0.60465115	0.8899594	1	592	tags=34%, lis
GOMF_PHOSPHOPROTEIN_PHOSPHATASE_ACTIVITY	27	0.189	0.889	0.60795456	0.89056796	1	374	tags=26%, lis
HP_CONTRACTURES_OF_THE_JOINTS_OF_THE_LOWER_LIMBS	26	0.190	0.888	0.60688406	0.8908925	1	1249	tags=73%, lis
GOBP_REGULATION_OF_MEMBRANE_PERMEABILITY	20	0.205	0.888	0.5744681	0.89101875	1	698	tags=40%, lis
HP_INFANTILE_ONSET	76	0.136	0.888	0.6316726	0.8902946	1	725	tags=36%, lis
HP_CEREBELLAR_ATROPHY	62	0.146	0.887	0.60394263	0.89088404	1	317	tags=19%, lis
REACTOME_PARASITE_INFECTION	15	0.227	0.887	0.59655833	0.8906359	1	188	tags=20%, lis
HP_DELAYED_ABILITY_TO_WALK	27	0.189	0.886	0.60761905	0.89058226	1	403	tags=22%, lis
HP_ABNORMALITY_OF_THE_GINGIVA	29	0.183	0.886	0.6315789	0.889935	1	1089	tags=59%, lis

HP_PRIMITIVE_NEUROECTODERMAL_TUMOR	15	0.230	0.886	0.5984405	0.8900841	1	293	tags=27%, lis
GOBP_SMALL_MOLECULE_METABOLIC_PROCESS	258	0.105	0.885	0.7190227	0.89138436	1	835	tags=39%, lis
GOCC_VACUOLAR_LUMEN	39	0.167	0.883	0.62147886	0.8942866	1	747	tags=38%, lis
GOBP_INTRACELLULAR_TRANSPORT	328	0.102	0.881	0.71428573	0.8965803	1	609	tags=27%, lis
HP_ABNORMAL_MUSCLE_FIBER_MORPHOLOGY	24	0.196	0.881	0.6040146	0.8964135	1	879	tags=54%, lis
GOCC_CHROMOSOME_CENTROMERIC_REGION	36	0.168	0.881	0.61130744	0.89587235	1	152	tags=14%, lis
HP_ABNORMAL_NERVOUS_SYSTEM_ELECTROPHYSIOLOGY	95	0.127	0.881	0.65313655	0.89510167	1	694	tags=36%, lis
HP_WEAKNESS_DUE_TO_UPPER_MOTOR_NEURON_DYSFUNCTION	71	0.140	0.880	0.65745854	0.8956626	1	864	tags=44%, lis
GOBP_REGULATION_OF_DNA_BINDING_TRANSCRIPTION_FACTOR_ACTIVITY	84	0.131	0.879	0.6886447	0.8962275	1	617	tags=30%, lis
REACTOME_GPCR_LIGAND_BINDING	19	0.214	0.879	0.60747665	0.8954143	1	280	tags=21%, lis
REACTOME_PTEN_REGULATION	42	0.165	0.879	0.6208791	0.8946101	1	908	tags=48%, lis
HP_UPPER_MOTOR_NEURON_DYSFUNCTION	198	0.109	0.879	0.6937394	0.89540106	1	870	tags=41%, lis
GOBP_LOCOMOTORY_BEHAVIOR	21	0.199	0.877	0.62350595	0.89727485	1	95	tags=14%, lis
HP_VARIABLE_EXPRESSIVITY	39	0.164	0.877	0.606947	0.89673567	1	523	tags=31%, lis
HP_ABNORMAL_ATRIOVENTRICULAR_VALVE_MORPHOLOGY	20	0.210	0.876	0.61904764	0.89734393	1	461	tags=30%, lis
GOBP_CELLULAR_RESPONSE_TO_OXYGEN_CONTAINING_COMPOUND	194	0.112	0.875	0.70826304	0.898401	1	549	tags=26%, lis
REACTOME_ER_TO_GOLGI_ANTEROGRADE_TRANSPORT	34	0.176	0.875	0.61966604	0.8978679	1	474	tags=26%, lis
HP_VASCULAR_DILATATION	34	0.169	0.873	0.62900186	0.9026252	1	992	tags=56%, lis
HP_ABNORMALITY_OF_THE_MENSTRUAL_CYCLE	25	0.194	0.872	0.62068963	0.90249056	1	849	tags=56%, lis
HP_ABNORMALITY_OF_THE_PULMONARY_ARTERY	22	0.200	0.872	0.6207547	0.90230435	1	314	tags=23%, lis
GOBP_CELL_REDOX_HOMEOSTASIS	15	0.231	0.871	0.6089494	0.90240836	1	1563	tags=93%, lis
REACTOME_TCF_DEPENDENT_SIGNALING_IN_RESPONSE_TO_WNT	39	0.164	0.871	0.6397059	0.9025666	1	592	tags=33%, lis
GOBP_NEGATIVE_REGULATION_OF_CELL_CELL_ADHESION	39	0.164	0.870	0.6351607	0.903054	1	419	tags=23%, lis
HP_HEPATOSPLENOMEGALY	29	0.181	0.870	0.64116573	0.90250117	1	50	tags=10%, lis
HP_ABNORMALITY_OF_PRENATAL_DEVELOPMENT_OR_BIRTH	96	0.127	0.869	0.6919014	0.9033744	1	807	tags=41%, lis
GOBP_POSITIVE_REGULATION_OF_CELL_CYCLE_PHASE_TRANSITION	16	0.228	0.869	0.635514	0.9033093	1	674	tags=44%, lis
HP_MACULE	23	0.196	0.869	0.6406534	0.90260965	1	779	tags=48%, lis
GOMF_PASSIVE_TRANSMEMBRANE_TRANSPORTER_ACTIVITY	23	0.190	0.868	0.639313	0.90248793	1	207	tags=17%, lis
GOBP_POSITIVE_REGULATION_OF_ORGANELLE_ORGANIZATION	117	0.124	0.868	0.68566495	0.90168786	1	425	tags=22%, lis
GOBP_MEMBRANE_INVAGINATION	19	0.202	0.868	0.6045198	0.9014242	1	905	tags=53%, lis
GOBP_REGULATION_OF_CELLULAR_AMIDE_METABOLIC_PROCESS	96	0.127	0.868	0.6913124	0.9007048	1	511	tags=25%, lis
HP_ABNORMALITY_OF_HINDBRAIN_MORPHOLOGY	136	0.119	0.867	0.70212764	0.901344	1	317	tags=17%, lis
HP_ABNORMAL_LIVER_MORPHOLOGY	123	0.120	0.867	0.6820604	0.90058	1	642	tags=31%, lis
GOBP_MITOCHONDRION_ORGANIZATION	129	0.120	0.867	0.6870629	0.8998631	1	771	tags=36%, lis
GOBP_TRANSITION_METAL_ION_TRANSPORT	26	0.184	0.867	0.64492756	0.8994445	1	679	tags=38%, lis
GOBP_MYELOID_CELL_DIFFERENTIATION	93	0.129	0.867	0.676951	0.8990524	1	312	tags=16%, lis
GOBP_NEGATIVE_REGULATION_OF_PROTEIN_DEPHOSPHORYLATION	16	0.224	0.866	0.607767	0.8987073	1	567	tags=38%, lis
GOBP_POSITIVE_REGULATION_OF_REACTIVE_OXYGEN_SPECIES_METABOLIC_PROCESS	22	0.198	0.866	0.6320755	0.89792967	1	111	tags=14%, lis
HP_DISPROPORTIONATE_SHORT_STATURE	17	0.215	0.866	0.6137566	0.8980224	1	1901	tags=100%, l
GOMF_GDP_BINDING	17	0.215	0.865	0.59855336	0.89922005	1	989	tags=59%, lis
GOBP_REGULATION_OF_RNA_SPLICING	38	0.164	0.865	0.6475096	0.8984428	1	340	tags=21%, lis
GOBP_REGULATION_OF_GENE_EXPRESSION_EPIGENETIC	40	0.160	0.864	0.67346936	0.8981948	1	118	tags=10%, lis
HP_APLASIA_HYPOPLASIA_OF_THE_PHALANGES_OF_THE_HAND	36	0.167	0.864	0.6305506	0.89777595	1	803	tags=44%, lis
GOBP_POST_EMBRYONIC_DEVELOPMENT	16	0.223	0.863	0.6339623	0.8996519	1	157	tags=19%, lis
GOBP_REGULATION_OF_MITOCHONDRIAL_MEMBRANE_PERMEABILITY	20	0.205	0.862	0.62406015	0.90051574	1	698	tags=40%, lis
REACTOME_METABOLISM_OF_VITAMINS_AND_COFACTORS	26	0.183	0.862	0.62618595	0.89985603	1	329	tags=23%, lis
REACTOME_MAPK_FAMILY_SIGNALING_CASCADES	68	0.138	0.861	0.65377533	0.901717	1	667	tags=35%, lis
PID_MET_PATHWAY	19	0.209	0.860	0.6433692	0.90166974	1	635	tags=42%, lis
GOMF_CYTOKINE_BINDING	18	0.213	0.860	0.6276803	0.9010102	1	37	tags=11%, lis
GOBP_RIBONUCLEOSIDE_TRIPHOSPHATE_METABOLIC_PROCESS	18	0.209	0.860	0.64084506	0.90046054	1	865	tags=56%, lis
HP_APLASIA_HYPOPLASIA_INVOLVING_BONES_OF_THE_UPPER_LIMBS	78	0.132	0.859	0.68058074	0.9007808	1	505	tags=26%, lis
GOMF_UBIQUITIN_LIKE_PROTEIN_TRANSFERASE_ACTIVITY	96	0.126	0.859	0.6940171	0.90049326	1	383	tags=20%, lis
GOBP_NEGATIVE_REGULATION_OF_CYTOSKELETON_ORGANIZATION	26	0.186	0.859	0.6387833	0.9002234	1	284	tags=19%, lis
HP_INCREASED_SUSCEPTIBILITY_TO_FRACTURES	23	0.194	0.858	0.630713	0.90068734	1	807	tags=52%, lis
HP_LEUKOCYTOSIS	17	0.211	0.858	0.65413535	0.90125626	1	901	tags=53%, lis
GOCC_MICROTUBULE_ORGANIZING_CENTER	121	0.119	0.854	0.7188612	0.9066806	1	458	tags=22%, lis
HP_ATTENTION_DEFICIT_HYPERACTIVITY_DISORDER	45	0.156	0.854	0.6879562	0.90587133	1	224	tags=16%, lis
HP_WEAKNESS_OF_FACIAL_MUSCULATURE	25	0.184	0.853	0.6329588	0.9080564	1	807	tags=48%, lis
GOMF_MRNA_BINDING	68	0.136	0.851	0.7061856	0.9103763	1	334	tags=18%, lis
HP_INTELLECTUAL_DISABILITY_MODERATE	39	0.159	0.851	0.66847825	0.9099272	1	724	tags=38%, lis
GOMF_CIS_REGULATORY_REGION_SEQUENCE_SPECIFIC_DNA_BINDING	121	0.118	0.851	0.72064054	0.9092321	1	671	tags=32%, lis
GOBP_RESPONSE_TO_HYDROGEN_PEROXIDE	32	0.174	0.850	0.66853935	0.9093686	1	767	tags=41%, lis
HP_HYPERTONIA	174	0.109	0.850	0.7376761	0.90907204	1	719	tags=33%, lis
HP_PROPORTIONATE_SHORT_STATURE	21	0.198	0.850	0.6516008	0.90837985	1	803	tags=52%, lis
GOBP_POSITIVE_REGULATION_OF_CELLULAR_AMIDE_METABOLIC_PROCESS	34	0.166	0.850	0.668616	0.9079963	1	540	tags=29%, lis
GOBP_NEGATIVE_REGULATION_OF_CELL_CYCLE_PHASE_TRANSITION	56	0.141	0.849	0.7103825	0.90925896	1	919	tags=48%, lis
HP_DYSMETRIA	25	0.188	0.848	0.6625	0.9101081	1	461	tags=28%, lis
GOCC_ER_TO_GOLGI_TRANSPORT_VESICLE_MEMBRANE	15	0.222	0.848	0.62799263	0.90963274	1	215	tags=20%, lis
GOBP_RESPIRATORY_BURST	17	0.210	0.847	0.6539197	0.91003424	1	1567	tags=88%, lis
GOBP_POSITIVE_REGULATION_OF_TRANSLATION	25	0.181	0.847	0.6548507	0.91003555	1	540	tags=32%, lis
GOBP_FOCAL_ADHESION_ASSEMBLY	17	0.211	0.846	0.6433692	0.909497	1	290	tags=24%, lis
PID_PDGFRB_PATHWAY	38	0.160	0.846	0.67657995	0.9089832	1	473	tags=26%, lis
GOBP_ORGANIC_HYDROXY_COMPOUND_BIOSYNTHETIC_PROCESS	28	0.177	0.846	0.6908397	0.90854937	1	506	tags=29%, lis
HP_IMPAIRED_SOCIAL_INTERACTIONS	31	0.173	0.846	0.67155963	0.90833074	1	577	tags=32%, lis
HP_COARSE_FACIAL_FEATURES	34	0.170	0.846	0.662963	0.90751606	1	505	tags=29%, lis
GOBP_RESPONSE_TO_CORTICOSTEROID	20	0.202	0.845	0.6852207	0.9083241	1	1116	tags=65%, lis
HP_ABNORMALITY_OF_THE_CEREBRAL_VENTRICLES	127	0.116	0.844	0.7364747	0.9082004	1	317	tags=17%, lis
GOBP_POSITIVE_REGULATION_OF_INTRINSIC_APOPTOTIC_SIGNALING_PATHWAY	18	0.204	0.844	0.6702899	0.9092096	1	157	tags=17%, lis
HP_ABNORMALITY_OF_THE_CHEEK	28	0.172	0.843	0.6697588	0.9086974	1	67	tags=11%, lis

HP_PROGRESSIVE_MICROCEPHALY	22	0.193	0.843	0.640625	0.90888214	1	980	tags=55%, lis
REACTOME_TRANSCRIPTIONAL_REGULATION_BY_RUNX1	53	0.145	0.843	0.6956522	0.90813303	1	958	tags=53%, lis
REACTOME_RAC1_GTPASE_CYCLE	52	0.145	0.843	0.7015707	0.90766317	1	479	tags=25%, lis
GOBP_MORPHOGENESIS_OF_A_BRANCHING_STRUCTURE	16	0.214	0.842	0.66972476	0.9082139	1	849	tags=56%, lis
HP_DYSPHONIA	21	0.196	0.841	0.6229508	0.9088982	1	805	tags=48%, lis
REACTOME_DEVELOPMENTAL_BIOLOGY	126	0.115	0.841	0.7614213	0.9090305	1	499	tags=25%, lis
HP_EEG_WITH_FOCAL_EPILEPTIFORM_DISCHARGES	19	0.196	0.840	0.67597765	0.9098152	1	675	tags=42%, lis
GOBP_LYMPHOCYTE_MEDIATED_IMMUNITY	48	0.146	0.840	0.6796296	0.9090705	1	617	tags=33%, lis
BIOCARTA_HIVNEF_PATHWAY	22	0.187	0.840	0.63925236	0.90842664	1	1589	tags=86%, lis
GOBP_CELLULAR_RESPONSE_TO_HYDROGEN_PEROXIDE	16	0.211	0.839	0.6457565	0.9085568	1	508	tags=31%, lis
HP_HYPOPLASIA_OF_THE_CORPUS_CALLOSUM	88	0.126	0.839	0.7274306	0.9080103	1	684	tags=33%, lis
REACTOME_NEGATIVE_REGULATION_OF_THE_PI3K_AKT_NETWORK	15	0.212	0.838	0.67712176	0.90890104	1	224	tags=20%, lis
HP_ABNORMAL_ATRIOVENTRICULAR_VALVE_PHYSIOLOGY	23	0.189	0.837	0.6721311	0.90915513	1	557	tags=35%, lis
HP_CARDIAC_CONDUCTION_ABNORMALITY	18	0.213	0.837	0.6587591	0.908911	1	793	tags=50%, lis
HP_TELANGIECTASIA	23	0.186	0.837	0.6923077	0.90892243	1	300	tags=22%, lis
HP_SPLENOMEGALY	72	0.132	0.836	0.7023593	0.90926385	1	642	tags=33%, lis
HP_PROMINENT_NASAL_BRIDGE	25	0.182	0.835	0.6711281	0.91028607	1	789	tags=44%, lis
GOBP_TRANSLATIONAL_INITIATION	32	0.167	0.835	0.6911197	0.9098516	1	1072	tags=56%, lis
GOBP_CELL_CELL_JUNCTION_ASSEMBLY	16	0.217	0.834	0.70229006	0.90981406	1	543	tags=38%, lis
HP_EPILEPTIC_SPASM	29	0.171	0.834	0.6941839	0.90988433	1	675	tags=38%, lis
KEGG_APOPTOSIS	31	0.169	0.834	0.693578	0.9093039	1	901	tags=52%, lis
GOBP_COPII_COATED_VESICLE_BUDDING	20	0.198	0.833	0.6616541	0.9091931	1	474	tags=30%, lis
HP_PROGRESSIVE_SPASTIC_PARAPLEGIA	17	0.215	0.833	0.66346157	0.90844434	1	1257	tags=76%, lis
KEGG_P53_SIGNALING_PATHWAY	17	0.213	0.833	0.67518246	0.90800893	1	1396	tags=82%, lis
GOBP_MAINTENANCE_OF_PROTEIN_LOCATION	21	0.194	0.831	0.6710775	0.9104741	1	450	tags=29%, lis
GOMF_OXIDOREDUCTASE_ACTIVITY	118	0.116	0.831	0.73472947	0.91013825	1	828	tags=39%, lis
HP_ABNORMALITY_OF_THE_ANKLES	15	0.220	0.831	0.69034606	0.9098274	1	1296	tags=80%, lis
HP_HEPATOMEGALY	90	0.120	0.830	0.7385965	0.90977603	1	832	tags=41%, lis
HP_MOTOR_DELAY	110	0.117	0.830	0.75	0.91060543	1	430	tags=21%, lis
GOBP_POSITIVE_REGULATION_OF_TELOMERE_MAINTENANCE	15	0.221	0.828	0.65498155	0.9133593	1	953	tags=60%, lis
HP_RESPIRATORY_DISTRESS	19	0.197	0.827	0.66988415	0.91301095	1	803	tags=47%, lis
HP_ABNORMAL_VISUAL_ELECTROPHYSIOLOGY	22	0.187	0.826	0.6764706	0.91396946	1	305	tags=23%, lis
GOBP_ESTABLISHMENT_OF_PROTEIN_LOCALIZATION	371	0.093	0.825	0.83505154	0.91508377	1	647	tags=28%, lis
HP_LEUKOENCEPHALOPATHY	45	0.149	0.825	0.7172285	0.91455454	1	581	tags=33%, lis
GOBP_REGULATION_OF_PROTEIN_DEPHOSPHORYLATION	32	0.161	0.825	0.69031143	0.9137995	1	462	tags=25%, lis
GOBP_REGULATION_OF_PEPTIDE_TRANSPORT	115	0.116	0.824	0.76792455	0.91444534	1	533	tags=25%, lis
HP_ABNORMAL_CELLULAR_PHENOTYPE	97	0.119	0.824	0.7309645	0.914373	1	646	tags=29%, lis
GOBP_NUCLEOSIDE_PHOSPHATE_BIOSYNTHETIC_PROCESS	46	0.152	0.822	0.70699435	0.9161029	1	1214	tags=67%, lis
HALLMARK_ESTROGEN_RESPONSE_EARLY	29	0.171	0.822	0.69158876	0.9153148	1	628	tags=38%, lis
HP_ABNORMAL_CEREBRAL_ARTERY_MORPHOLOGY	15	0.211	0.822	0.6685499	0.9154987	1	251	tags=20%, lis
HP_PARAPLEGIA_PARAPARESIS	45	0.145	0.821	0.712204	0.9153804	1	1281	tags=71%, lis
REACTOME_MITOTIC_PROMETAPHASE	24	0.180	0.821	0.6808118	0.91497415	1	211	tags=17%, lis
GOBP_REGULATION_OF_PROTEIN_LOCALIZATION_TO_NUCLEUS	29	0.171	0.821	0.7117117	0.9146644	1	438	tags=24%, lis
GOBP_ENDOCYTOSIS	109	0.116	0.820	0.79046	0.9150323	1	645	tags=31%, lis
GOBP_NEGATIVE_REGULATION_OF_HEMOPOIESIS	23	0.181	0.820	0.68650794	0.91558176	1	359	tags=22%, lis
GOBP_CELLULAR_RESPONSE_TO_CHEMICAL_STRESS	80	0.125	0.818	0.7403509	0.9166267	1	385	tags=20%, lis
GOMF_CADHERIN_BINDING	75	0.125	0.818	0.73938227	0.9162164	1	438	tags=21%, lis
GOCC_POLYSOME	15	0.211	0.817	0.6895874	0.91680324	1	80	tags=13%, lis
GOMF_PHOSPHATASE_ACTIVITY	35	0.160	0.817	0.7064057	0.9170136	1	571	tags=31%, lis
GOCC_NUCLEAR_ENVELOPE	89	0.121	0.817	0.765625	0.9162548	1	652	tags=33%, lis
GOBP_NUCLEOSIDE_TRIPHOSPHATE_METABOLIC_PROCESS	23	0.183	0.815	0.7029126	0.9194108	1	1015	tags=61%, lis
GOBP_REGULATION_OF_CELLULAR_COMPONENT_SIZE	62	0.132	0.814	0.731569	0.92039186	1	432	tags=23%, lis
GOBP_INORGANIC_ION_TRANSMEMBRANE_TRANSPORT	94	0.120	0.813	0.7478261	0.9198764	1	813	tags=39%, lis
HP_BENIGN_NEOPLASM_OF_THE_CENTRAL_NERVOUS_SYSTEM	19	0.198	0.813	0.6925859	0.9198755	1	864	tags=53%, lis
GOBP_POSITIVE_REGULATION_OF_PROTEIN_PHOSPHORYLATION	133	0.110	0.813	0.7902098	0.9191519	1	497	tags=23%, lis
HP_BLEPHAROPHIMOSIS	25	0.175	0.812	0.72284645	0.9192031	1	724	tags=40%, lis
HP_ABNORMAL_INFLAMMATORY_RESPONSE	152	0.106	0.812	0.80316347	0.91895515	1	503	tags=22%, lis
GOMF_CATION_TRANSMEMBRANE_TRANSPORTER_ACTIVITY	53	0.139	0.812	0.7432188	0.918833	1	753	tags=40%, lis
GOBP_POSITIVE_REGULATION_OF_MITOCHONDRION_ORGANIZATION	22	0.182	0.811	0.7045045	0.919562	1	1010	tags=55%, lis
HP_ABNORMAL_GRANULOCYTE_COUNT	38	0.157	0.811	0.73406196	0.9189669	1	281	tags=16%, lis
GOBP_GLIOGENESIS	32	0.163	0.811	0.7152778	0.91843826	1	419	tags=25%, lis
GOCC_SYNAPSE	142	0.108	0.811	0.7926421	0.917812	1	638	tags=30%, lis
GOBP_POSITIVE_REGULATION_OF_T_CELL_PROLIFERATION	16	0.205	0.809	0.7013233	0.91902757	1	448	tags=31%, lis
GOMF_PHOSPHOPROTEIN_BINDING	21	0.184	0.809	0.6990476	0.9194617	1	138	tags=14%, lis
HP_ABNORMALITY_OF_ADRENAL_MORPHOLOGY	16	0.205	0.808	0.6965889	0.9190363	1	779	tags=50%, lis
GOMF_CARBOHYDRATE_BINDING	29	0.167	0.808	0.7129456	0.91896415	1	144	tags=14%, lis
REACTOME_FOXO_MEDIATED_TRANSCRIPTION	19	0.196	0.808	0.716763	0.9184829	1	425	tags=26%, lis
HP_FUNCTIONAL_ABNORMALITY_OF_THE_GASTROINTESTINAL_TRACT	159	0.105	0.808	0.8051282	0.91809016	1	461	tags=20%, lis
GOMF_LIPID_BINDING	110	0.114	0.808	0.7810858	0.91746813	1	641	tags=32%, lis
GOMF_UNFOLDED_PROTEIN_BINDING	35	0.158	0.806	0.73540145	0.918593	1	392	tags=23%, lis
GOBP_RESPONSE_TO_STARVATION	38	0.152	0.804	0.732852	0.92124015	1	644	tags=32%, lis
GOBP_EPITHELIAL_TUBE_MORPHOGENESIS	25	0.172	0.804	0.73333335	0.92129993	1	359	tags=24%, lis
GOCC_PERIKARYON	15	0.208	0.803	0.7011719	0.9213593	1	398	tags=27%, lis
HP_ABNORMALITY_OF_THE_BREAST	62	0.130	0.802	0.7562724	0.9226328	1	864	tags=45%, lis
KEGG_CHEMOKINE_SIGNALING_PATHWAY	34	0.157	0.802	0.7356322	0.9218763	1	290	tags=18%, lis
REACTOME_PROTEIN_UBIQUITINATION	17	0.196	0.800	0.6957364	0.9238415	1	328	tags=24%, lis
HP_ABNORMALITY_OF_THE_SMALL_INTESTINE	24	0.180	0.799	0.7378277	0.92550194	1	13	tags=8%, list
HP_ABNORMALITY_OF_BLOOD_AND_BLOOD_FORMING_TISSUES	213	0.098	0.799	0.8438061	0.92473596	1	495	tags=23%, lis
HP_AUTOIMMUNITY	40	0.149	0.799	0.7324955	0.9245857	1	476	tags=25%, lis

HP_ABNORMALITY_OF_THE_PULMONARY_VASCULATURE	24	0.176	0.799	0.71482176	0.9238192	1	314	tags=21%, lis
GOMF_NAD_P_H_DEHYDROGENASE_QUINONE_ACTIVITY	19	0.189	0.798	0.7271028	0.92368823	1	899	tags=47%, lis
GOMF_OXIDOREDUCTASE_ACTIVITY_ACTING_ON_NAD_P_H_QUINONE_OR_SIMILAR_COMPOUND	19	0.189	0.797	0.68582374	0.92438287	1	899	tags=47%, lis
HP_ABNORMAL_TRACHEA_MORPHOLOGY	17	0.200	0.796	0.6896552	0.92627054	1	1104	tags=65%, lis
HP_ABNORMALITY_OF_FACIAL_MUSCULATURE	46	0.141	0.795	0.7504621	0.92568487	1	725	tags=37%, lis
HP_MYALGIA	22	0.178	0.795	0.7260788	0.9263182	1	902	tags=55%, lis
HP_ABNORMAL_MYELOID_LEUKOCYTE_MORPHOLOGY	55	0.136	0.794	0.7669173	0.9261682	1	642	tags=31%, lis
GOBP_EPITHELIAL_CELL_DEVELOPMENT	33	0.158	0.794	0.73653847	0.9257862	1	314	tags=21%, lis
HP_MULTIFOCAL_EPILEPTIFORM_DISCHARGES	16	0.202	0.793	0.71910113	0.92593765	1	999	tags=63%, lis
GOBP_RAS_PROTEIN_SIGNAL_TRANSDUCTION	74	0.126	0.793	0.765324	0.9253176	1	314	tags=18%, lis
GOBP_REGULATION_OF_PHAGOCYTOSIS	23	0.180	0.789	0.7405303	0.9306391	1	635	tags=35%, lis
GOBP_NUCLEUS_ORGANIZATION	26	0.167	0.788	0.7314815	0.9322795	1	197	tags=15%, lis
GOBP_PROTEIN_POLYMERIZATION	55	0.132	0.788	0.76459855	0.93158793	1	202	tags=15%, lis
HP_MUSCLE_ABNORMALITY_RELATED_TO_MITOCHONDRIAL_DYSFUNCTION	17	0.198	0.788	0.72168905	0.9311963	1	899	tags=53%, lis
GOCC_CELL_SUBSTRATE_JUNCTION	81	0.120	0.787	0.7889734	0.9309027	1	500	tags=25%, lis
HP_ABNORMALITY_OF_THE_FOREARM	31	0.160	0.787	0.7612782	0.9303085	1	138	tags=13%, lis
GOBP_CENTRAL_NERVOUS_SYSTEM_DEVELOPMENT	115	0.113	0.786	0.7948276	0.9309726	1	1012	tags=49%, lis
HP_ABNORMALITY_OF_EARLOBE	17	0.198	0.784	0.7210145	0.9340815	1	356	tags=24%, lis
HP_FUNCTIONAL_MOTOR_DEFICIT	66	0.126	0.784	0.79636365	0.9333197	1	409	tags=23%, lis
GOBP_NUCLEOBASE_CONTAINING_SMALL_MOLECULE_METABOLIC_PROCESS	95	0.116	0.783	0.78938055	0.93376964	1	865	tags=42%, lis
GOBP_REGULATION_OF_CELL_DEVELOPMENT	74	0.121	0.783	0.7838828	0.9335276	1	314	tags=18%, lis
GOCC_RIBONUCLEOPROTEIN_GRANULE	66	0.124	0.782	0.7816901	0.9335885	1	333	tags=18%, lis
REACTOME_INTRACELLULAR_SIGNALING_BY_SECOND_MESSENGERS	76	0.122	0.782	0.7927757	0.9334322	1	908	tags=42%, lis
HP_VOMITING	38	0.150	0.782	0.75	0.9328802	1	828	tags=39%, lis
HP_ABNORMALITY_OF_THE_CEREBRAL_SUBCORTEX	176	0.099	0.780	0.86135185	0.93507415	1	729	tags=33%, lis
GOBP_ANTERIOR_POSTERIOR_PATTERN_SPECIFICATION	17	0.193	0.779	0.75339806	0.93530357	1	314	tags=24%, lis
HP_RECURRENT_RESPIRATORY_INFECTIONS	79	0.121	0.778	0.8007181	0.93593615	1	476	tags=22%, lis
KEGG_JAK_STAT_SIGNALING_PATHWAY	26	0.169	0.778	0.75	0.9362647	1	443	tags=27%, lis
GOBP_CELLULAR_RESPONSE_TO_MOLECULE_OF_BACTERIAL_ORIGIN	45	0.141	0.777	0.7743119	0.9363543	1	543	tags=29%, lis
GOCC_NADH_DEHYDROGENASE_COMPLEX	19	0.189	0.777	0.74025977	0.9361377	1	899	tags=47%, lis
HP_ABNORMAL_PYRAMIDAL_SIGN	62	0.126	0.776	0.80415946	0.93559486	1	980	tags=50%, lis
REACTOME_MITOTIC_SPINDLE_CHECKPOINT	15	0.202	0.775	0.75457877	0.93639463	1	211	tags=20%, lis
REACTOME_TRANSPORT_TO_THE_GOLGI_AND_SUBSEQUENT_MODIFICATION	40	0.143	0.775	0.7941176	0.9361757	1	474	tags=25%, lis
GOBP_POSITIVE_REGULATION_OF_ESTABLISHMENT_OF_PROTEIN_LOCALIZATION	69	0.123	0.775	0.8125	0.93623376	1	445	tags=23%, lis
HP_GASTROSTOMY_TUBE_FEEDING_IN_INFANCY	21	0.183	0.772	0.7462121	0.93913615	1	19	tags=10%, lis
GOBP_CARBOHYDRATE_DERIVATIVE_METABOLIC_PROCESS	170	0.099	0.772	0.84731543	0.93867505	1	700	tags=31%, lis
GOBP_POSITIVE_REGULATION_OF_PROTEIN_MODIFICATION_PROCESS	184	0.098	0.772	0.86130136	0.93792427	1	518	tags=23%, lis
HP_APLASIA_HYPOPLASIA_OF_THE_EXTREMITIES	102	0.111	0.772	0.8123894	0.9372014	1	863	tags=42%, lis
HP_FOCAL_ONSET_SEIZURE	41	0.144	0.772	0.79591835	0.93659675	1	504	tags=27%, lis
HP_ABNORMALITY_OF_DENTAL_MORPHOLOGY	34	0.151	0.771	0.76014763	0.93755496	1	50	tags=9%, list=
GOBP_PHOSPHATIDYLINOSITOL_METABOLIC_PROCESS	31	0.155	0.770	0.7900763	0.937416	1	395	tags=23%, lis
HP_CONGENITAL_ONSET	44	0.137	0.770	0.76953125	0.9372028	1	793	tags=41%, lis
REACTOME_DISEASES_OF_SIGNAL_TRANSDUCTION_BY_GROWTH_FACTOR_RECEPTORS_AND_SEC	97	0.113	0.770	0.8174061	0.936509	1	489	tags=23%, lis
GOBP_NEGATIVE_REGULATION_OF_CELL_DEVELOPMENT	25	0.170	0.769	0.7719928	0.9367862	1	537	tags=32%, lis
HP_ABNORMAL_ABDOMEN_MORPHOLOGY	129	0.105	0.769	0.8534483	0.93666303	1	812	tags=38%, lis
HP_ACUTE_LEUKEMIA	19	0.180	0.768	0.79924953	0.93687457	1	557	tags=32%, lis
GOBP_CHROMOSOME_SEGREGATION	44	0.140	0.768	0.77697843	0.9366538	1	211	tags=14%, lis
GOBP_CARBOHYDRATE_DERIVATIVE_BIOSYNTHETIC_PROCESS	102	0.109	0.767	0.83304346	0.93645144	1	753	tags=34%, lis
GOBP_POSITIVE_REGULATION_OF_CELLULAR_COMPONENT_BIOGENESIS	98	0.112	0.767	0.8062827	0.93681836	1	568	tags=27%, lis
GOBP_MRNA_TRANSPORT	39	0.143	0.766	0.7787934	0.9362768	1	648	tags=36%, lis
PID_TCR_PATHWAY	16	0.196	0.766	0.74531835	0.93614596	1	283	tags=19%, lis
GOBP_CELLULAR_AMIDE_METABOLIC_PROCESS	184	0.097	0.766	0.8670121	0.93577147	1	540	tags=24%, lis
GOBP_REGULATION_OF_RESPONSE_TO_OXIDATIVE_STRESS	15	0.204	0.765	0.7633136	0.93639183	1	111	tags=13%, lis
HP_ABNORMALITY_OF_BONE_MARROW_CELL_MORPHOLOGY	34	0.150	0.765	0.7874763	0.93585855	1	1871	tags=94%, lis
GOCC_NUCLEOLUS	175	0.098	0.764	0.8509532	0.9359471	1	444	tags=19%, lis
GOMF_KINASE_ACTIVATOR_ACTIVITY	21	0.180	0.763	0.75842696	0.9364791	1	644	tags=33%, lis
HP_MYELODYSPLASIA	17	0.191	0.763	0.7622505	0.93662685	1	245	tags=18%, lis
GOMF_TRANSLATION_REGULATOR_ACTIVITY_NUCLEIC_ACID_BINDING	21	0.174	0.762	0.7598499	0.93728197	1	540	tags=29%, lis
GOBP_CYTOSOLIC_TRANSPORT	38	0.143	0.761	0.79566	0.93670744	1	123	tags=11%, lis
GOBP_REGULATION_OF_MRNA_SPLICING_VIA_SPLICEOSOME	25	0.167	0.761	0.7807692	0.93621516	1	98	tags=12%, lis
HP_THICK_VERMILION_BORDER	41	0.142	0.761	0.8053435	0.9360597	1	167	tags=12%, lis
GOCC_POLYMERIC_CYTOSKELETAL_FIBER	89	0.112	0.761	0.82871974	0.93557954	1	645	tags=29%, lis
GOBP_NEGATIVE_REGULATION_OF_LYMPHOCYTE_ACTIVATION	33	0.151	0.761	0.7616488	0.9349879	1	258	tags=15%, lis
GOBP_CELL_CELL_JUNCTION_ORGANIZATION	22	0.173	0.760	0.7557252	0.9351365	1	543	tags=32%, lis
HP_ABNORMALITY_OF_THE_UPPER_RESPIRATORY_TRACT	73	0.118	0.758	0.818662	0.9367849	1	1104	tags=55%, lis
GOBP_CELL_CYCLE_PROCESS	228	0.092	0.758	0.8894472	0.9369026	1	689	tags=31%, lis
GOBP_DE_NOVO_PROTEIN_FOLDING	20	0.183	0.754	0.76994437	0.94171053	1	380	tags=25%, lis
HP_NEOPLASM_OF_THE_PANCREAS	16	0.190	0.753	0.7519084	0.94140756	1	864	tags=50%, lis
GOBP_RESPONSE_TO_CADMIUM_ION	15	0.196	0.753	0.7684825	0.94083256	1	1104	tags=67%, lis
HP_ENCEPHALOPATHY	45	0.136	0.752	0.8178634	0.94216824	1	577	tags=29%, lis
GOCC_CATALYTIC_COMPLEX	327	0.086	0.751	0.9244663	0.9430926	1	646	tags=28%, lis
GOBP_REGULATION_OF_MITOCHONDRIAL_MEMBRANE_POTENTIAL	16	0.191	0.750	0.77717394	0.94257355	1	131	tags=13%, lis
HP_LOWER_LIMB_SPASTICITY	42	0.138	0.750	0.7781818	0.9422772	1	1257	tags=69%, lis
GOBP_PROTEIN_N_LINKED_GLYCOSYLATION	16	0.189	0.749	0.7480315	0.94251686	1	442	tags=31%, lis
HP_ABNORMALITY_OF_ESOPHAGUS_PHYSIOLOGY	111	0.105	0.749	0.8605852	0.9420269	1	215	tags=12%, lis
GOBP_NEGATIVE_REGULATION_OF_CELL_DIFFERENTIATION	97	0.109	0.749	0.8367347	0.9413007	1	537	tags=25%, lis
GOCC_NEURON_PROJECTION_CYTOPLASM	15	0.199	0.749	0.7643678	0.9407403	1	1940	tags=100%, l
HP_ABNORMAL_SIZE_OF_THE_PALPEBRAL_FISSURES	39	0.139	0.749	0.80755395	0.9400133	1	724	tags=38%, lis
GOBP_RIBOSE_PHOSPHATE_BIOSYNTHETIC_PROCESS	37	0.142	0.748	0.7823741	0.94004697	1	865	tags=46%, lis

GOCC_VESICLE_LUMEN	65	0.121	0.748	0.8493648	0.93993306	1	747	tags=35%, lis
GOBP_ORGANOPHOSPHATE_METABOLIC_PROCESS	165	0.097	0.748	0.89446366	0.93928355	1	826	tags=38%, lis
GOMF_DNA_BINDING_TRANSCRIPTION_REPRESSOR_ACTIVITY	34	0.145	0.748	0.8197026	0.93884474	1	590	tags=29%, lis
HP_PAIN	102	0.108	0.747	0.8454546	0.93874496	1	503	tags=25%, lis
HP_OBSESSIVE_COMPULSIVE_BEHAVIOR	17	0.191	0.746	0.7619048	0.93948805	1	80	tags=12%, lis
GOBP_DNA_TEMPLATED_TRANSCRIPTION_INITIATION	38	0.143	0.746	0.8211091	0.9387947	1	671	tags=34%, lis
PID_MYC_REPRESS_PATHWAY	16	0.190	0.745	0.80221814	0.93940896	1	1137	tags=69%, lis
HP_ABNORMALITY_OF_FEMALE_EXTERNAL_GENITALIA	15	0.192	0.744	0.76937985	0.9397876	1	807	tags=53%, lis
HP_ABNORMALITY_OF_THE_FEMALE_GENITALIA	56	0.127	0.744	0.80225986	0.9390816	1	864	tags=43%, lis
REACTOME_TRANSCRIPTIONAL_REGULATION_BY_TP53	86	0.114	0.744	0.8427562	0.9384727	1	864	tags=38%, lis
GOBP_DNA_MODIFICATION	20	0.176	0.744	0.7893738	0.9377773	1	359	tags=25%, lis
HP_PROGRESSIVE_CEREBELLAR_ATAXIA	21	0.168	0.744	0.76545453	0.937265	1	1257	tags=71%, lis
GOBP_NUCLEAR_TRANSCRIBED_MRNA_POLY_A_TAIL_SHORTENING	15	0.196	0.744	0.79379565	0.93661004	1	427	tags=27%, lis
GOBP_REGULATION_OF_PROTEIN_TARGETING	20	0.175	0.744	0.793621	0.9360927	1	437	tags=25%, lis
GOMF_MRNA_3_UTR_BINDING	21	0.167	0.743	0.7528517	0.93563074	1	392	tags=24%, lis
GOBP_REGULATION_OF_RAS_PROTEIN_SIGNAL_TRANSDUCTION	42	0.137	0.743	0.81305116	0.93550164	1	750	tags=38%, lis
HP_PECTUS_EXCAVATUM	44	0.134	0.741	0.81386864	0.93798065	1	710	tags=36%, lis
GOMF_TRANSLATION_FACTOR_ACTIVITY_RNA_BINDING	19	0.177	0.740	0.7909091	0.93756276	1	501	tags=26%, lis
GOBP_NEGATIVE_REGULATION_OF_NF_KAPPAB_TRANSCRIPTION_FACTOR_ACTIVITY	25	0.165	0.740	0.7882798	0.9372862	1	562	tags=32%, lis
REACTOME_TRANSLATION	38	0.141	0.739	0.8221831	0.93783987	1	1020	tags=55%, lis
HP_NEOPLASM_OF_THE_STOMACH	16	0.191	0.739	0.8022388	0.9372721	1	473	tags=31%, lis
GOBP_NEGATIVE_REGULATION_OF_APOPTOTIC_SIGNALING_PATHWAY	47	0.131	0.738	0.81508076	0.9374239	1	344	tags=17%, lis
GOBP_INTRACELLULAR_PROTEIN_TRANSPORT	223	0.089	0.737	0.90192646	0.9379734	1	507	tags=22%, lis
GOBP_TRNA_METABOLIC_PROCESS	19	0.173	0.737	0.79766536	0.9372549	1	1013	tags=63%, lis
HP_ABNORMALITY_OF_GLOBE_SIZE	28	0.155	0.737	0.78909093	0.9373445	1	807	tags=43%, lis
GOBP_NEGATIVE_REGULATION_OF_NERVOUS_SYSTEM_DEVELOPMENT	23	0.165	0.736	0.7789855	0.93689424	1	314	tags=22%, lis
GOBP_REGULATION_OF_INTRACELLULAR_PROTEIN_TRANSPORT	65	0.121	0.735	0.8361582	0.9376614	1	445	tags=22%, lis
REACTOME_DDX58_IFIH1_MEDIATED_INDUCTION_OF_INTERFERON_ALPHA_BETA	28	0.158	0.734	0.8233083	0.9389099	1	858	tags=46%, lis
HP_ABNORMALITY_OF_NEUTROPHILS	46	0.131	0.733	0.83864915	0.93943787	1	617	tags=28%, lis
KEGG_CARDIAC_MUSCLE_CONTRACTION	15	0.190	0.732	0.7863894	0.94015664	1	254	tags=20%, lis
GOMF_RNA_BINDING	366	0.082	0.730	0.9443535	0.94138104	1	543	tags=22%, lis
HP_AUTISM	33	0.144	0.730	0.82560295	0.94081116	1	286	tags=18%, lis
HP_ABNORMAL_RETINAL_MORPHOLOGY	114	0.103	0.728	0.8790036	0.94249415	1	902	tags=43%, lis
GOBP_CYTOPLASMIC_TRANSLATION	17	0.181	0.728	0.8146067	0.94199634	1	1072	tags=59%, lis
GOBP_POSITIVE_REGULATION_OF_CYTOSKELETON_ORGANIZATION	39	0.141	0.727	0.8100358	0.9426639	1	496	tags=26%, lis
GOCC_MEMBRANE_COAT	19	0.175	0.725	0.8203267	0.943825	1	863	tags=47%, lis
REACTOME_MEMBRANE_TRAFFICKING	133	0.098	0.723	0.8998331	0.94619995	1	887	tags=40%, lis
GOBP_ESTABLISHMENT_OF_CELL_POLARITY	25	0.158	0.722	0.8136882	0.94637245	1	123	tags=12%, lis
GOCC_CENTRIOLE	16	0.188	0.720	0.8103448	0.94795954	1	370	tags=25%, lis
GOMF_TRANSFERASE_ACTIVITY_TRANSFERRING_HEXOSYL_GROUPS	27	0.150	0.720	0.8371212	0.94782996	1	434	tags=22%, lis
HP_INCREASED_BLOOD_PRESSURE	49	0.126	0.719	0.8725314	0.94756913	1	314	tags=16%, lis
HP_ABNORMAL_REFLEX	170	0.093	0.719	0.9162393	0.9471098	1	861	tags=39%, lis
PID_ERA_GENOMIC_PATHWAY	16	0.183	0.718	0.80035025	0.9471387	1	1778	tags=94%, lis
HP_DIARRHEA	45	0.129	0.716	0.842803	0.9489224	1	28	tags=7%, list-
GOBP_MULTICELLULAR_ORGANISMAL_HOMEOSTASIS	78	0.111	0.716	0.8705674	0.9486148	1	224	tags=13%, lis
HP_ABNORMALITY_OF_THE_CEREBELLAR_VERMIS	39	0.138	0.716	0.83365947	0.9481152	1	807	tags=41%, lis
REACTOME_CILIUM_ASSEMBLY	25	0.153	0.716	0.82894737	0.94741225	1	1231	tags=68%, lis
REACTOME_SIGNALING_BY_INTERLEUKINS	101	0.103	0.715	0.88164663	0.94778496	1	496	tags=24%, lis
REACTOME_INTRA_GOLGI_AND_RETROGRADE_GOLGI_TO_ER_TRAFFIC	46	0.130	0.715	0.8512241	0.94740725	1	863	tags=41%, lis
GOBP_CELLULAR_RESPONSE_TO_BIOTIC_STIMULUS	54	0.121	0.714	0.845614	0.9471539	1	543	tags=28%, lis
HP_SEVERE_SHORT_STATURE	15	0.189	0.714	0.83815026	0.9466144	1	495	tags=33%, lis
HP_ARTERIAL_STENOSIS	16	0.185	0.713	0.8095238	0.9468556	1	396	tags=25%, lis
HP_HYPERREFLEXIA	112	0.100	0.713	0.8935018	0.94625276	1	1296	tags=66%, lis
GOCC_RIBOSOME	24	0.160	0.712	0.82954544	0.9463435	1	623	tags=33%, lis
GOBP_TELOMERE_MAINTENANCE_VIA_TELOMERE_LENGTHENING	18	0.173	0.712	0.81800765	0.946432	1	427	tags=28%, lis
REACTOME_TP53_REGULATES_TRANSCRIPTION_OF_DNA_REPAIR_GENES	16	0.183	0.710	0.8358209	0.9475388	1	439	tags=25%, lis
HP_NEOPLASM_OF_THE_BREAST	20	0.168	0.710	0.8303748	0.9469521	1	864	tags=45%, lis
HALLMARK_DNA_REPAIR	40	0.134	0.710	0.86630034	0.94638616	1	542	tags=28%, lis
GOBP_POLYOL_METABOLIC_PROCESS	18	0.173	0.710	0.8194175	0.9460443	1	666	tags=33%, lis
HP_CEREBELLAR_MALFORMATION	49	0.124	0.709	0.8592322	0.94580114	1	711	tags=35%, lis
HP_MYOPATHY	34	0.139	0.707	0.8342441	0.9470877	1	793	tags=44%, lis
HP_SLOPING_FOREHEAD	20	0.162	0.707	0.8238748	0.94743806	1	67	tags=10%, lis
HP_VESTIBULAR_DYSFUNCTION	24	0.159	0.705	0.85265225	0.94815004	1	849	tags=46%, lis
KEGG_CELL_CYCLE	24	0.153	0.705	0.83114445	0.94832915	1	780	tags=38%, lis
GOBP_REGULATION_OF_DNA_REPAIR	17	0.174	0.704	0.8342967	0.9479842	1	1061	tags=59%, lis
KEGG_REGULATION_OF_ACTIN_CYTOSKELETON	32	0.142	0.703	0.83576643	0.948941	1	290	tags=19%, lis
GOBP_REGULATION_OF_TRANSLATIONAL_INITIATION	24	0.152	0.702	0.8219697	0.94850093	1	1305	tags=71%, lis
GOBP_ESTABLISHMENT_OF_ORGANELLE_LOCALIZATION	75	0.111	0.702	0.89084506	0.9484838	1	589	tags=27%, lis
HP_PARALYSIS	23	0.159	0.700	0.8296296	0.9498552	1	770	tags=39%, lis
HALLMARK_MYC_TARGETS_V1	63	0.115	0.698	0.8584559	0.9515477	1	501	tags=25%, lis
GOBP_MITOCHONDRIAL_TRANSMEMBRANE_TRANSPORT	20	0.162	0.698	0.8265896	0.95111805	1	753	tags=45%, lis
GOBP_MONONUCLEAR_CELL_MIGRATION	27	0.148	0.697	0.83450705	0.9511535	1	720	tags=37%, lis
GOBP_MITOCHONDRIAL_TRANSPORT	58	0.115	0.695	0.8804348	0.9530363	1	716	tags=34%, lis
GOBP_ORGANONITROGEN_COMPOUND_BIOSYNTHETIC_PROCESS	276	0.081	0.694	0.9550173	0.9523907	1	548	tags=22%, lis
REACTOME_REGULATION_OF_HSF1_MEDIATED_HEAT_SHOCK_RESPONSE	21	0.158	0.694	0.8538012	0.9517105	1	411	tags=24%, lis
GOBP_ALCOHOL_METABOLIC_PROCESS	47	0.123	0.693	0.84335154	0.9524961	1	251	tags=15%, lis
HP_VASCULAR_SKIN_ABNORMALITY	88	0.103	0.692	0.8814433	0.95269847	1	617	tags=28%, lis
GOMF_UBIQUITIN_LIKE_PROTEIN_LIGASE_ACTIVITY	59	0.117	0.692	0.8943662	0.9519997	1	337	tags=17%, lis
REACTOME_G_ALPHA_I_SIGNALLING_EVENTS	29	0.146	0.689	0.85444236	0.9545828	1	881	tags=45%, lis

GOBP_NEGATIVE_REGULATION_OF_GENE_EXPRESSION_EPIGENETIC	18	0.167	0.688	0.86538464	0.9545546	1	198	tags=17%, lis
REACTOME_SUMOYLATION	45	0.124	0.687	0.8782288	0.95534605	1	36	tags=7%, list
HP_MALABSORPTION	27	0.144	0.684	0.86168224	0.9573994	1	694	tags=37%, lis
GOBP_NEGATIVE_REGULATION_OF_ORGANELLE_ORGANIZATION	49	0.118	0.683	0.87061	0.957821	1	508	tags=24%, lis
GOMF_N_ACETYLTRANSFERASE_ACTIVITY	15	0.180	0.683	0.8606403	0.957538	1	1746	tags=93%, lis
HP_SKELETAL_MUSCLE_ATROPHY	75	0.107	0.683	0.9108734	0.9569595	1	879	tags=43%, lis
HP_ABNORMAL_LARYNX_MORPHOLOGY	21	0.161	0.682	0.8742747	0.9566401	1	933	tags=52%, lis
GOBP_CELL_DEATH_IN_RESPONSE_TO_OXIDATIVE_STRESS	18	0.167	0.682	0.8460145	0.9562239	1	111	tags=11%, lis
GOBP_ER_NUCLEUS_SIGNALING_PATHWAY	22	0.155	0.682	0.86105675	0.9560756	1	278	tags=18%, lis
GOBP_REGULATION_OF_PROTEIN_MODIFICATION_BY_SMALL_PROTEIN_CONJUGATION_OR_REMO	63	0.113	0.681	0.89806676	0.95569503	1	683	tags=32%, lis
GOBP_MICROTUBULE_BASED_MOVEMENT	33	0.134	0.681	0.8599641	0.95502234	1	89	tags=9%, list
HP_ACUTE_PHASE_RESPONSE	15	0.178	0.681	0.84909093	0.95471966	1	617	tags=40%, lis
GOBP_GLYCOPROTEIN_BIOSYNTHETIC_PROCESS	49	0.120	0.679	0.8814433	0.955687	1	486	tags=22%, lis
GOBP_IRE1_MEDIATED_UNFOLDED_PROTEIN_RESPONSE	19	0.162	0.678	0.86972475	0.95601696	1	725	tags=42%, lis
REACTOME_P75_NTR_RECEPTOR_MEDIATED_SIGNALLING	22	0.155	0.677	0.867031	0.95606124	1	188	tags=14%, lis
HP_ABDOMINAL_PAIN	43	0.123	0.672	0.8852459	0.9604448	1	495	tags=26%, lis
HP_ABNORMAL_AORTIC_VALVE_MORPHOLOGY	16	0.171	0.670	0.87295824	0.9620624	1	902	tags=50%, lis
GOBP_ORGANOPHOSPHATE_CATABOLIC_PROCESS	17	0.166	0.670	0.87732345	0.9614554	1	991	tags=53%, lis
GOBP_PROTEIN_TARGETING_TO_MEMBRANE	23	0.150	0.669	0.86168224	0.9615789	1	1881	tags=96%, lis
GOBP_VIRAL_GENE_EXPRESSION	23	0.150	0.668	0.8702011	0.96129376	1	439	tags=26%, lis
REACTOME_IRE1ALPHA_ACTIVATES_CHAPERONES	17	0.168	0.666	0.8659218	0.96272564	1	445	tags=29%, lis
HP_GENERALIZED_ONSET_SEIZURE	46	0.119	0.666	0.886076	0.9620675	1	679	tags=33%, lis
HP_DILATION_OF_LATERAL_VENTRICLES	15	0.177	0.665	0.8707865	0.9622435	1	279	tags=20%, lis
GOBP_MITOTIC_G1_S_TRANSITION_CHECKPOINT	15	0.171	0.665	0.8530466	0.9615399	1	1769	tags=93%, lis
HP_DELAYED_GROSS_MOTOR_DEVELOPMENT	47	0.116	0.665	0.8982143	0.9611068	1	50	tags=6%, list
GOMF_MAGNESIUM_ION_BINDING	37	0.125	0.662	0.9048474	0.963556	1	386	tags=22%, lis
GOBP_REGULATION_OF_ALPHA_BETA_T_CELL_ACTIVATION	23	0.149	0.660	0.86531365	0.9644172	1	258	tags=17%, lis
GOMF_N_ACYLTRANSFERASE_ACTIVITY	21	0.154	0.660	0.88539743	0.9638144	1	1746	tags=90%, lis
GOBP_TRANSCRIPTION_ELONGATION_FROM_RNA_POLYMERASE_II_PROMOTER	19	0.158	0.656	0.87077534	0.96626186	1	1706	tags=89%, lis
HP_BILATERAL_TONIC_CLONIC_SEIZURE	27	0.139	0.655	0.8812616	0.96638316	1	729	tags=37%, lis
GOCC_PHAGOCYTIC_VESICLE	40	0.120	0.654	0.8961039	0.9670043	1	635	tags=30%, lis
HP_ABNORMALITY_OF_THE_HELIX	24	0.145	0.653	0.87142855	0.96754783	1	629	tags=33%, lis
GOCC_PRECATALYTIC_SPLICEOSOME	18	0.159	0.651	0.88530463	0.9681928	1	1094	tags=61%, lis
GOBP_AMINOGLYCAN_BIOSYNTHETIC_PROCESS	16	0.168	0.650	0.8636364	0.96835977	1	434	tags=25%, lis
HP_AORTIC_ANEURYSM	27	0.137	0.649	0.8848263	0.9686954	1	992	tags=56%, lis
GOBP_EPIDERMAL_CELL_DIFFERENTIATION	19	0.155	0.646	0.90352505	0.9704027	1	409	tags=26%, lis
HP_ABNORMAL_PARANASAL_SINUS_MORPHOLOGY	30	0.133	0.646	0.90267175	0.9696986	1	671	tags=33%, lis
GOCC_RIBONUCLEOPROTEIN_COMPLEX	136	0.086	0.645	0.9492754	0.96962607	1	975	tags=44%, lis
HP_ABNORMALITY_OF_THE_ZYGOMATIC_BONE	36	0.124	0.645	0.9161905	0.96940243	1	828	tags=42%, lis
GOBP_TRANSLATIONAL_ELONGATION	19	0.155	0.643	0.89013034	0.97005993	1	1207	tags=68%, lis
GOMF_TRANSFERASE_ACTIVITY_TRANSFERRING_GLYCOSYL_GROUPS	43	0.117	0.642	0.9229323	0.97017753	1	348	tags=16%, lis
HP_FEMALE_REPRODUCTIVE_SYSTEM_NEOPLASM	16	0.162	0.640	0.8994614	0.9709473	1	944	tags=50%, lis
GOMF_ION_CHANNEL_BINDING	29	0.134	0.638	0.92249525	0.97162426	1	784	tags=41%, lis
GOBP_DEVELOPMENT_OF_PRIMARY_SEXUAL_CHARACTERISTICS	32	0.127	0.636	0.91590494	0.9727812	1	688	tags=34%, lis
GOBP_PEPTIDE_BIOSYNTHETIC_PROCESS	112	0.088	0.633	0.94655174	0.9745937	1	540	tags=23%, lis
GOBP_ORGANELLE_ASSEMBLY	103	0.090	0.632	0.94545454	0.9747154	1	70	tags=6%, list
HP_NAUSEA_AND_VOMITING	69	0.101	0.632	0.9373882	0.97412044	1	577	tags=25%, lis
HALLMARK_IL6_JAK_STAT3_SIGNALING	24	0.143	0.632	0.8959538	0.9734793	1	726	tags=38%, lis
GOBP_PURINE_CONTAINING_COMPOUND_BIOSYNTHETIC_PROCESS	36	0.122	0.631	0.91696113	0.97301644	1	969	tags=50%, lis
GOBP_CYTOSKELETON_DEPENDENT_INTRACELLULAR_TRANSPORT	30	0.127	0.628	0.91516244	0.9752784	1	1940	tags=97%, lis
PID_TXA2PATHWAY	15	0.163	0.627	0.9168207	0.97493774	1	550	tags=33%, lis
GOCC_CATALYTIC_STEP_2_SPLICEOSOME	25	0.138	0.626	0.905838	0.9746824	1	530	tags=28%, lis
GOBP_MITOCHONDRIAL_OUTER_MEMBRANE_PERMEABILIZATION	17	0.154	0.626	0.9118705	0.9742777	1	698	tags=35%, lis
GOBP_REGULATION_OF_INTRACELLULAR_TRANSPORT	80	0.096	0.626	0.94755876	0.97391695	1	487	tags=21%, lis
GOBP_RNA_DESTABILIZATION	17	0.159	0.625	0.8976234	0.97342724	1	398	tags=24%, lis
HP_THORACIC_AORTIC_ANEURYSM	20	0.146	0.624	0.9070248	0.97371906	1	807	tags=45%, lis
GOBP_CARBOHYDRATE_METABOLIC_PROCESS	93	0.092	0.622	0.93406594	0.97432745	1	700	tags=31%, lis
GOBP_GLAND_MORPHOGENESIS	15	0.161	0.621	0.89943075	0.97453046	1	683	tags=40%, lis
GOBP_NEGATIVE_REGULATION_OF_EXTRINSIC_APOPTOTIC_SIGNALING_PATHWAY	24	0.135	0.620	0.9198543	0.97432303	1	581	tags=29%, lis
GOBP_NUCLEOTIDE_PHOSPHORYLATION	22	0.140	0.620	0.9	0.9739638	1	670	tags=36%, lis
REACTOME_BIOLOGICAL_OXIDATIONS	19	0.145	0.618	0.92060494	0.97468257	1	92	tags=11%, lis
HP_ABNORMALITY_OF_THE_ELBOW	35	0.121	0.618	0.92	0.97402096	1	793	tags=40%, lis
GOBP_APOPTOTIC_MITOCHONDRIAL_CHANGES	30	0.126	0.616	0.9171171	0.97485256	1	1010	tags=50%, lis
HP_GASTROINTESTINAL_HEMORRHAGE	27	0.129	0.615	0.90555555	0.97502893	1	644	tags=33%, lis
GOMF_SINGLE_STRANDED_DNA_BINDING	20	0.147	0.614	0.9230769	0.9751037	1	1005	tags=55%, lis
GOCC_LATE_ENDOSOME_MEMBRANE	32	0.119	0.612	0.9027027	0.9754394	1	607	tags=28%, lis
GOBP_CELLULAR_CARBOHYDRATE_METABOLIC_PROCESS	49	0.110	0.612	0.943958	0.97486	1	400	tags=18%, lis
GOBP_CELLULAR_RESPONSE_TO_HEAT	26	0.130	0.608	0.9279113	0.97658926	1	411	tags=23%, lis
HALLMARK_PI3K_AKT_MTOR_SIGNALING	29	0.125	0.606	0.93333334	0.97755384	1	644	tags=31%, lis
HP_ROUND_FACE	18	0.148	0.606	0.9203187	0.97693413	1	619	tags=33%, lis
GOCC_AXON	77	0.094	0.605	0.945993	0.976463	1	347	tags=16%, lis
GOBP_DNA_DAMAGE_RESPONSE_SIGNAL_TRANSDUCTION_BY_P53_CLASS_MEDIATOR	24	0.133	0.605	0.93358636	0.9758506	1	186	tags=13%, lis
HP_DELAYED_MYELINATION	30	0.124	0.603	0.93050194	0.97676224	1	612	tags=30%, lis
HP_ABNORMAL_HEART_VALVE_PHYSIOLOGY	37	0.115	0.601	0.94280446	0.97734964	1	557	tags=27%, lis
GOBP_REGULATION_OF_PHOSPHOPROTEIN_PHOSPHATASE_ACTIVITY	30	0.123	0.599	0.9279113	0.9777972	1	462	tags=23%, lis
GOBP_REGULATION_OF_WOUND_HEALING	16	0.157	0.599	0.9322034	0.97739536	1	719	tags=38%, lis
HP_ABNORMAL_SYSTEMIC_BLOOD_PRESSURE	57	0.099	0.596	0.96240604	0.9780634	1	398	tags=18%, lis
HP_RENAL_AGENESIS	17	0.150	0.590	0.9299611	0.9810294	1	249	tags=18%, lis
HP_ABNORMALITY_OF_HEPATOBILIARY_SYSTEM_PHYSIOLOGY	28	0.123	0.590	0.94	0.98067236	1	1281	tags=71%, lis

HP_VERTIGO	21	0.139	0.590	0.94095236	0.9800507	1	1089	tags=57%, lis
HP_TACHYCARDIA	17	0.145	0.586	0.9116541	0.9813114	1	871	tags=47%, lis
HP_ABNORMAL_LARGE_INTESTINE_MORPHOLOGY	40	0.110	0.586	0.94671404	0.98067164	1	901	tags=43%, lis
HP_ABNORMAL_ORAL_PHYSIOLOGY	25	0.129	0.586	0.9378428	0.98011607	1	1249	tags=68%, lis
HP_ABNORMAL_ORAL_MUCOSA_MORPHOLOGY	38	0.111	0.584	0.93333334	0.98076016	1	849	tags=39%, lis
REACTOME_CELLULAR_RESPONSE_TO_HEAT_STRESS	24	0.129	0.581	0.9348231	0.98143506	1	411	tags=21%, lis
GOBP_ACTIN_CYTOSKELETON_REORGANIZATION	19	0.139	0.580	0.9340866	0.9812079	1	423	tags=21%, lis
GOBP_CELL_PROJECTION_ASSEMBLY	71	0.091	0.580	0.95977014	0.98070836	1	314	tags=14%, lis
HP_ABNORMAL_CLAVICLE_MORPHOLOGY	15	0.153	0.580	0.92804426	0.9801139	1	1786	tags=93%, lis
HALLMARK_PROTEIN_SECRETION	36	0.111	0.578	0.9577735	0.98049676	1	905	tags=44%, lis
HP_MUSCULAR_HYPOTONIA_OF_THE_TRUNK	34	0.112	0.577	0.94485295	0.98012227	1	48	tags=6%, list
GOBP_ORGANELLE_LOCALIZATION	107	0.081	0.574	0.97513324	0.98129267	1	864	tags=37%, lis
HP_ASCITES	19	0.137	0.569	0.95211786	0.98297244	1	1281	tags=68%, lis
GOMF_ACETYLTRANSFERASE_ACTIVITY	19	0.137	0.569	0.94332725	0.9823172	1	1746	tags=89%, lis
HP_SOFT_TISSUE_SARCOMA	24	0.123	0.567	0.9424861	0.9825551	1	286	tags=17%, lis
HP_POOR_EYE_CONTACT	24	0.128	0.567	0.94676805	0.982017	1	577	tags=29%, lis
HP_IMMUNODEFICIENCY	47	0.101	0.564	0.9444444	0.9826475	1	617	tags=28%, lis
GOBP_CELL_KILLING	22	0.127	0.559	0.9546314	0.9845746	1	1735	tags=91%, lis
GOBP_POSITIVE_REGULATION_OF_MEMBRANE_PERMEABILITY	18	0.138	0.558	0.96183205	0.9845414	1	425	tags=22%, lis
GOBP_NUCLEOSIDE_DIPHOSPHATE_METABOLIC_PROCESS	23	0.125	0.555	0.9456522	0.9849485	1	670	tags=35%, lis
HP_NEOPLASM_OF_THE_LARGE_INTESTINE	18	0.134	0.555	0.9599237	0.98448676	1	1311	tags=72%, lis
GOBP_AMIDE_BIOSYNTHETIC_PROCESS	146	0.075	0.553	0.9916388	0.9847594	1	1032	tags=47%, lis
GOBP_AGING	47	0.100	0.549	0.9802867	0.985708	1	508	tags=23%, lis
REACTOME_METABOLISM_OF_CARBOHYDRATES	44	0.098	0.548	0.9668616	0.985501	1	434	tags=20%, lis
HP_LOWER_EXTREMITY_JOINT_DISLOCATION	37	0.104	0.546	0.9601518	0.9854574	1	905	tags=43%, lis
GOBP_LIPOSACCHARIDE_METABOLIC_PROCESS	18	0.134	0.542	0.95535713	0.9867117	1	746	tags=39%, lis
GOMF_ACTIVE_ION_TRANSMEMBRANE_TRANSPORTER_ACTIVITY	15	0.140	0.540	0.9588015	0.9865697	1	522	tags=27%, lis
HP_CUTANEOUS_PHOTOSENSITIVITY	17	0.135	0.539	0.9677419	0.9864649	1	1869	tags=94%, lis
GOMF_ENDOPEPTIDASE_REGULATOR_ACTIVITY	18	0.132	0.536	0.97781885	0.98665106	1	640	tags=33%, lis
GOBP_REGULATION_OF_MITOCHONDRION_ORGANIZATION	37	0.101	0.532	0.9669118	0.98743314	1	337	tags=16%, lis
HP_GENITAL_NEOPLASM	27	0.109	0.527	0.97402596	0.98886865	1	341	tags=19%, lis
REACTOME_GLYCOSAMINOGLYCAN_METABOLISM	17	0.131	0.515	0.9808061	0.99199826	1	434	tags=24%, lis
GOBP_MITOCHONDRIAL_MEMBRANE_ORGANIZATION	39	0.096	0.507	0.98674244	0.9936356	1	753	tags=33%, lis
HP_ABNORMAL_AORTIC_VALVE_PHYSIOLOGY	19	0.120	0.504	0.97884613	0.9939824	1	668	tags=32%, lis
HP_ABNORMALITY_OF_SALIVATION	18	0.120	0.498	0.9758813	0.99486685	1	1874	tags=94%, lis
HP_LEUKEMIA	35	0.098	0.497	0.9828897	0.9943735	1	304	tags=14%, lis
REACTOME_COPI_MEDIATED_ANTEROGRADE_TRANSPORT	22	0.112	0.497	0.9827916	0.9938326	1	123	tags=9%, list
HP_BREAST_CARCINOMA	18	0.122	0.494	0.9828897	0.99392104	1	710	tags=33%, lis
GOCC_DISTAL_AXON	44	0.090	0.493	0.99261993	0.9933536	1	340	tags=16%, lis
GOCC_PIGMENT_GRANULE	31	0.096	0.489	0.9782609	0.993802	1	637	tags=29%, lis
HP_ABNORMAL_UTERUS_MORPHOLOGY	18	0.117	0.483	0.990942	0.99434304	1	293	tags=17%, lis
GOCC_NEURON_TO_NEURON_SYNAPSE	28	0.104	0.482	0.9942639	0.9938637	1	284	tags=14%, lis
HP_LISSENCEPHALY	19	0.114	0.467	0.986692	0.9963374	1	828	tags=37%, lis
REACTOME_SARS_COV_2_INFECTION	20	0.108	0.460	0.99429655	0.9968044	1	833	tags=40%, lis
GOBP_LIPOPOLYSACCHARIDE_MEDIATED_SIGNALING_PATHWAY	18	0.112	0.459	0.9926605	0.99632627	1	720	tags=33%, lis
HP_RECURRENT_LOWER_RESPIRATORY_TRACT_INFECTIONS	17	0.112	0.444	0.9961977	0.9976626	1	1940	tags=94%, lis
HP_DIALEPTIC_SEIZURE	23	0.097	0.435	0.991274	0.9980151	1	675	tags=30%, lis
REACTOME_TRANSLOCATION_OF_SLC2A4_GLUT4_TO_THE_PLASMA_MEMBRANE	18	0.105	0.433	0.9961759	0.9975592	1	188	tags=11%, lis
GOBP_RESPONSE_TO_HEAT	31	0.086	0.427	0.99446493	0.9974604	1	411	tags=19%, lis
HP_ABNORMAL_PATELLA_MORPHOLOGY	21	0.097	0.422	0.9926874	0.9971867	1	905	tags=43%, lis
GOBP_MICROTUBULE_ORGANIZING_CENTER_ORGANIZATION	18	0.100	0.413	0.99445474	0.99721885	1	1024	tags=50%, lis
