# Supplementary material for: Biodegradable nanoparticles induce cGAS/STING-dependent reprogramming of myeloid cells to promote tumor immunotherapy
Source: Front Immunol. 2022 Aug 18;13:887649. doi: 10.3389/fimmu.2022.887649 (PMC9433741; doi:10.3389/fimmu.2022.887649)
Supplement: Supplementary file 11 [file Table_5.pdf]

Supplemental Table 5. Signaling Pathway Analysis for Macrophages - 3 Doses Once Every 3 Days - ONP-302 vs. Saline

NAME	SIZE	ES	NES	NOM p-val	FDR q-val	FWER p-val	RANK AT MA	LEADING ED
HALLMARK_INTERFERON_GAMMA_RESPONSE	79	0.507	3.098	0	0	0	351	tags=48%, lis
REACTOME_CELLULAR_RESPONSE_TO_CHEMICAL_STRESS	65	0.488	2.880	0	0	0	431	tags=51%, lis
REACTOME_AUF1_HNRNP_D0_BINDS_AND_DESTABILIZES_MRNA	27	0.626	2.848	0	0	0	406	tags=67%, lis
KEGG_OXIDATIVE_PHOSPHORYLATION	45	0.535	2.820	0	4.22E-04	0.002	741	tags=80%, lis
GOBP_POSITIVE_REGULATION_OF_RESPONSE_TO_BIOTIC_STIMULUS	65	0.479	2.789	0	3.37E-04	0.002	353	tags=45%, lis
REACTOME_SCF_SKP2_MEDIATED_DEGRADATION_OF_P27_P21	24	0.648	2.779	0	2.81E-04	0.002	406	tags=71%, lis
REACTOME_RESPIRATORY_ELECTRON_TRANSPORT_ATP_SYNTHESIS_BY_CHEMIOSMOTIC_COUPLIN	41	0.546	2.773	0	2.41E-04	0.002	647	tags=76%, lis
GOBP_OXIDATIVE_PHOSPHORYLATION	44	0.538	2.770	0	2.11E-04	0.002	841	tags=91%, lis
KEGG_PROTEASOME	22	0.672	2.755	0	1.87E-04	0.002	253	tags=64%, lis
KEGG_HUNTINGTONS_DISEASE	49	0.508	2.752	0	1.69E-04	0.002	741	tags=80%, lis
REACTOME_DECTIN_1_MEDIATED_NONCANONICAL_NF_KB_SIGNALING	27	0.600	2.716	0	1.53E-04	0.002	406	tags=63%, lis
REACTOME_NEGATIVE_REGULATION_OF_NOTCH4_SIGNALING	24	0.623	2.708	0	1.41E-04	0.002	406	tags=67%, lis
REACTOME_REGULATION_OF_RUNX2_EXPRESSION_AND_ACTIVITY	27	0.599	2.699	0	1.96E-04	0.003	406	tags=63%, lis
REACTOME_CROSS_PRESENTATION_OF_SOLUBLE_EXOGENOUS_ANTIGENS_ENDOSOMES	22	0.639	2.696	0	1.82E-04	0.003	253	tags=59%, lis
KEGG_PARKINSONS_DISEASE	43	0.509	2.683	0	1.70E-04	0.003	741	tags=79%, lis
GOCC_INNER_MITOCHONDRIAL_MEMBRANE_PROTEIN_COMPLEX	36	0.539	2.670	0	1.59E-04	0.003	841	tags=89%, lis
GOBP_TUMOR_NECROSIS_FACTOR_MEDIATED_SIGNALING_PATHWAY	46	0.501	2.668	0	1.50E-04	0.003	255	tags=46%, lis
REACTOME_CYCLIN_A_CDK2_ASSOCIATED_EVENTS_AT_S_PHASE_ENTRY	28	0.578	2.661	0	1.41E-04	0.003	406	tags=61%, lis
REACTOME_TNFR2_NON_CANONICAL_NF_KB_PATHWAY	30	0.570	2.654	0	1.34E-04	0.003	406	tags=60%, lis
REACTOME_ABC_FAMILY_PROTEINS_MEDIATED_TRANSPORT	26	0.591	2.644	0	1.70E-04	0.004	563	tags=73%, lis
GOCC_RESPIRASOME	29	0.572	2.641	0	2.02E-04	0.005	841	tags=93%, lis
GOCC_MITOCHONDRIAL_PROTEIN_CONTAINING_COMPLEX	54	0.466	2.634	0	1.93E-04	0.005	647	tags=70%, lis
REACTOME_DEGRADATION_OF_DVL	23	0.614	2.624	0	1.84E-04	0.005	406	tags=65%, lis
REACTOME_REGULATION_OF_HMOX1_EXPRESSION_AND_ACTIVITY	29	0.583	2.621	0	1.77E-04	0.005	406	tags=62%, lis
GOCC_ORGANELLE_INNER_MEMBRANE	89	0.413	2.618	0	1.70E-04	0.005	655	tags=64%, lis
REACTOME_ASYMMETRIC_LOCALIZATION_OF_PCP_PROTEINS	23	0.614	2.614	0	1.63E-04	0.005	406	tags=65%, lis
REACTOME_ORC1_REMOVAL_FROM_CHROMATIN	24	0.623	2.614	0	1.57E-04	0.005	406	tags=67%, lis
REACTOME_REGULATION_OF_RUNX3_EXPRESSION_AND_ACTIVITY	23	0.607	2.584	0	2.43E-04	0.008	406	tags=65%, lis
REACTOME_ASSEMBLY_OF_THE_PRE_REPLICATIVE_COMPLEX	24	0.594	2.576	0	2.93E-04	0.01	406	tags=63%, lis
REACTOME_DEFECTIVE_CFTR_CAUSES_CYSTIC_FIBROSIS	24	0.590	2.575	0	2.83E-04	0.01	406	tags=63%, lis
REACTOME_DNA_REPLICATION_PRE_INITIATION	25	0.579	2.574	0	3.01E-04	0.011	406	tags=60%, lis
REACTOME_REGULATION_OF_MRNA_STABILITY_BY_PROTEINS_THAT_BIND_AU_RICH_ELEMENTS	35	0.530	2.571	0	2.92E-04	0.011	344	tags=51%, lis
REACTOME_METABOLISM_OF_POLYAMINES	25	0.578	2.571	0	2.83E-04	0.011	299	tags=56%, lis
REACTOME_THE_CITRIC_ACID_TCA_CYCLE_AND_RESPIRATORY_ELECTRON_TRANSPORT	47	0.473	2.566	0	2.75E-04	0.011	647	tags=72%, lis
REACTOME_SWITCHING_OF_ORIGINS_TO_A_POST_REPLICATIVE_STATE	27	0.570	2.563	0	2.67E-04	0.011	406	tags=59%, lis
REACTOME_ABC_TRANSPORTER_DISORDERS	24	0.590	2.552	0	2.83E-04	0.012	406	tags=63%, lis
REACTOME_FCERI_MEDIATED_NF_KB_ACTIVATION	38	0.506	2.551	0	2.75E-04	0.012	283	tags=47%, lis
GOBP_REGULATION_OF_INNATE_IMMUNE_RESPONSE	77	0.422	2.549	0	2.91E-04	0.013	353	tags=43%, lis
REACTOME_REGULATION_OF_PTEN_STABILITY_AND_ACTIVITY	28	0.574	2.537	0	3.27E-04	0.015	253	tags=54%, lis
GOBP_ACTIVATION_OF_INNATE_IMMUNE_RESPONSE	43	0.488	2.534	0	3.40E-04	0.016	413	tags=51%, lis
REACTOME_RESPIRATORY_ELECTRON_TRANSPORT	32	0.521	2.534	0	3.31E-04	0.016	841	tags=88%, lis
GOBP_INTERLEUKIN_1_MEDIATED_SIGNALING_PATHWAY	40	0.485	2.528	0	3.43E-04	0.016	347	tags=50%, lis
GOBP_POSITIVE_REGULATION_OF_DEFENSE_RESPONSE	86	0.411	2.522	0	3.55E-04	0.017	413	tags=43%, lis
REACTOME_REGULATION_OF_RAS_BY_GAPS	24	0.592	2.513	0	3.88E-04	0.019	406	tags=63%, lis
REACTOME_CYTOPROTECTION_BY_HMOX1	54	0.451	2.511	0	3.79E-04	0.019	431	tags=48%, lis
GOBP_REGULATION_OF_RESPONSE_TO_BIOTIC_STIMULUS	91	0.394	2.507	0	3.90E-04	0.02	363	tags=41%, lis
GOBP_INNATE_IMMUNE_RESPONSE_ACTIVATING_SIGNAL_TRANSDUCTION	36	0.504	2.506	0	3.81E-04	0.02	283	tags=47%, lis
REACTOME_SIGNALING_BY_NOTCH4	28	0.555	2.503	0	4.44E-04	0.024	406	tags=61%, lis
REACTOME_DEGRADATION_OF_GLI1_BY_THE_PROTEASOME	26	0.576	2.495	0	4.70E-04	0.026	406	tags=62%, lis
GOCC_PEPTIDASE_COMPLEX	29	0.542	2.492	0	4.61E-04	0.026	253	tags=48%, lis
GOBP_ANAPHASE_PROMOTING_COMPLEX_DEPENDENT_CATABOLIC_PROCESS	27	0.546	2.490	0	4.51E-04	0.026	253	tags=48%, lis
REACTOME_THE_ROLE_OF_GTSE1_IN_G2_M_PROGRESSION_AFTER_G2_CHECKPOINT	27	0.563	2.488	0	4.59E-04	0.027	253	tags=52%, lis
REACTOME_DEGRADATION_OF_BETA_CATENIN_BY_THE_DESTRUCTION_COMPLEX	28	0.540	2.482	0	5.00E-04	0.03	406	tags=57%, lis
GOCC_ENDOPEPTIDASE_COMPLEX	25	0.567	2.477	0	5.06E-04	0.031	253	tags=52%, lis
GOCC_MITOCHONDRIAL_ENVELOPE	130	0.362	2.477	0	4.97E-04	0.031	655	tags=57%, lis
REACTOME_REGULATION_OF_EXPRESSION_OF_SLITS_AND_ROBOS	31	0.516	2.475	0	4.88E-04	0.031	559	tags=65%, lis
REACTOME_G1_S_DNA_DAMAGE_CHECKPOINTS	26	0.552	2.471	0	4.95E-04	0.032	253	tags=54%, lis
REACTOME_HEDGEHOG_LIGAND_BIOGENESIS	25	0.567	2.464	0	5.30E-04	0.035	253	tags=52%, lis
REACTOME_PCP_CE_PATHWAY	29	0.531	2.457	0	5.67E-04	0.038	548	tags=66%, lis
GOCC_RESPIRATORY_CHAIN_COMPLEX	25	0.566	2.457	0.00200400	5.57E-04	0.038	841	tags=92%, lis
REACTOME_MITOTIC_G1_PHASE_AND_G1_S_TRANSITION	34	0.507	2.446	0.00193050	5.76E-04	0.04	283	tags=47%, lis
REACTOME_INTERLEUKIN_1_SIGNALING	40	0.475	2.446	0	5.67E-04	0.04	344	tags=48%, lis
GOBP_REGULATION_OF_CELLULAR_AMINE_METABOLIC_PROCESS	25	0.555	2.441	0	5.71E-04	0.041	253	tags=52%, lis
REACTOME_CELLULAR_RESPONSE_TO_HYPOXIA	28	0.538	2.438	0	6.29E-04	0.046	406	tags=57%, lis
GOBP_MITOCHONDRIAL_TRANSMEMBRANE_TRANSPORT	19	0.597	2.437	0	6.32E-04	0.047	603	tags=84%, lis
REACTOME_TRANSCRIPTIONAL_REGULATION_BY_RUNX2	34	0.508	2.437	0	6.35E-04	0.048	283	tags=47%, lis
REACTOME_APC_C_CDH1_MEDIATED_DEGRADATION_OF_CDC20_AND_OTHER_APC_C_CDH1_TAR	27	0.550	2.430	0	7.03E-04	0.054	253	tags=48%, lis
GOBP_REGULATION_OF_CELLULAR_AMINO_ACID_METABOLIC_PROCESS	24	0.573	2.427	0	7.42E-04	0.058	253	tags=54%, lis
GOBP_DETOXIFICATION	20	0.594	2.425	0	7.57E-04	0.06	288	tags=50%, lis
GOBP_RESPONSE_TO_INTERLEUKIN_1	52	0.441	2.422	0	8.20E-04	0.066	413	tags=48%, lis
GOBP_DEFENSE_RESPONSE_TO_OTHER_ORGANISM	200	0.324	2.418	0	8.32E-04	0.068	363	tags=33%, lis
REACTOME_TCR_SIGNALING	44	0.459	2.411	0	8.91E-04	0.073	406	tags=50%, lis
REACTOME_APC_C_MEDIATED_DEGRADATION_OF_CELL_CYCLE_PROTEINS	29	0.542	2.407	0	8.79E-04	0.073	406	tags=55%, lis
HALLMARK_REACTIVE_OXYGEN_SPECIES_PATHWAY	16	0.627	2.398	0	9.60E-04	0.081	355	tags=63%, lis
GOBP_DEFENSE_RESPONSE	275	0.302	2.396	0	0.00101687	0.087	273	tags=26%, lis
GOBP_ATP_SYNTHESIS_COUPLED_ELECTRON_TRANSPORT	32	0.502	2.385	0	0.00111725	0.097	841	tags=88%, lis

GOBP_DEFENSE_RESPONSE_TO_BACTERIUM	30	0.504	2.383	0	0.001113954	0.098	198	tags=40%, lis
GOBP_AMINE_METABOLIC_PROCESS	29	0.516	2.379	0	0.001143483	0.102	299	tags=52%, lis
REACTOME_ANTIGEN_PROCESSING_CROSS_PRESENTATION	42	0.462	2.378	0	0.001129008	0.102	344	tags=45%, lis
GOBP_RESPIRATORY_ELECTRON_TRANSPORT_CHAIN	35	0.485	2.374	0	0.001265346	0.112	841	tags=86%, lis
REACTOME_DEGRADATION_OF_AXIN	25	0.554	2.371	0	0.001292276	0.116	253	tags=52%, lis
GOBP_RESPONSE_TO_TUMOR_NECROSIS_FACTOR	65	0.404	2.370	0	0.001307257	0.119	255	tags=37%, lis
REACTOME_STABILIZATION_OF_P53	25	0.532	2.364	0	0.001394130	0.128	253	tags=52%, lis
GOBP_ANTIGEN_PROCESSING_AND_PRESENTATION_OF_EXOGENOUS_PEPTIDE_ANTIGEN_VIA_MH	38	0.477	2.361	0	0.001418537	0.132	253	tags=45%, lis
GOBP_PROTEASOMAL_UBIQUITIN_INDEPENDENT_PROTEIN_CATABOLIC_PROCESS	15	0.641	2.356	0	0.001482309	0.14	253	tags=60%, lis
REACTOME_HEDGEHOG_ON_STATE	27	0.521	2.355	0.001941747	0.001494645	0.143	253	tags=48%, lis
GOBP_SCF_DEPENDENT_PROTEASOMAL_UBIQUITIN_DEPENDENT_PROTEIN_CATABOLIC_PROCESS	29	0.505	2.339	0	0.001861212	0.177	283	tags=48%, lis
GOBP_CELLULAR_OXIDANT_DETOXIFICATION	17	0.606	2.337	0	0.001888253	0.182	288	tags=53%, lis
GOBP_CELLULAR_RESPONSE_TO_TOXIC_SUBSTANCE	19	0.581	2.327	0	0.001972026	0.191	288	tags=47%, lis
REACTOME_DNA_REPLICATION	31	0.489	2.320	0	0.002208140	0.212	445	tags=55%, lis
GOBP_MORPHOGENESIS_OF_A_POLARIZED_EPITHELIUM	33	0.486	2.319	0	0.002183875	0.212	576	tags=64%, lis
REACTOME_HEDGEHOG_OFF_STATE	31	0.488	2.317	0	0.002197855	0.215	406	tags=52%, lis
GOBP_NEGATIVE_REGULATION_OF_CELL_CYCLE_G2_M_PHASE_TRANSITION	34	0.471	2.313	0	0.002247665	0.222	406	tags=53%, lis
GOCC_PROTON_TRANSPORTING_TWO_SECTOR_ATPASE_COMPLEX	19	0.584	2.312	0	0.002251452	0.224	603	tags=79%, lis
GOBP_INNATE_IMMUNE_RESPONSE	169	0.318	2.309	0	0.002263789	0.227	353	tags=33%, lis
GOBP_ESTABLISHMENT_OF_TISSUE_POLARITY	29	0.497	2.296	0.001937984	0.002525936	0.249	547	tags=62%, lis
GOBP_REGULATION_OF_DEFENSE_RESPONSE	132	0.339	2.289	0	0.002649085	0.261	353	tags=36%, lis
HP_HEMOLYTIC_ANEMIA	32	0.480	2.287	0	0.002639646	0.261	254	tags=38%, lis
GOBP_POSITIVE_REGULATION_OF_RESPONSE_TO_EXTERNAL_STIMULUS	109	0.348	2.284	0	0.002682686	0.264	283	tags=31%, lis
GOBP_CYTOKINE_MEDIATED_SIGNALING_PATHWAY	166	0.317	2.268	0	0.003074646	0.3	349	tags=32%, lis
REACTOME_SEPARATION_OF_SISTER_CHROMATIDS	36	0.452	2.265	0	0.003222114	0.314	253	tags=39%, lis
REACTOME_S_PHASE	39	0.450	2.264	0	0.003232680	0.317	283	tags=41%, lis
GOBP_ANTIGEN_PROCESSING_AND_PRESENTATION_OF_PEPTIDE_ANTIGEN_VIA_MHC_CLASS_I	44	0.434	2.261	0	0.003300614	0.324	331	tags=45%, lis
GOBP_ANTIGEN_PROCESSING_AND_PRESENTATION_OF_PEPTIDE_ANTIGEN	64	0.387	2.260	0	0.003293509	0.327	437	tags=45%, lis
REACTOME_RUNX1_REGULATES_TRANSCRIPTION_OF_GENES_INVOLVED_IN_DIFFERENTIATION_OF	28	0.495	2.255	0	0.003376615	0.336	447	tags=57%, lis
GOBP_ACTIVATION_OF_IMMUNE_RESPONSE	106	0.342	2.254	0	0.003352830	0.336	417	tags=39%, lis
GOBP_ANTIGEN_RECEPTOR_MEDIATED_SIGNALING_PATHWAY	67	0.371	2.243	0	0.003658107	0.361	547	tags=49%, lis
GOBP_RESPONSE_TO_TOXIC_SUBSTANCE	33	0.462	2.239	0	0.003727696	0.369	288	tags=36%, lis
REACTOME_INTERLEUKIN_1_FAMILY_SIGNALING	44	0.422	2.237	0	0.003748247	0.374	295	tags=41%, lis
HALLMARK_OXIDATIVE_PHOSPHORYLATION	53	0.416	2.236	0	0.003799054	0.379	653	tags=66%, lis
GOBP_REGULATION_OF_TRANSCRIPTION_FROM_RNA_POLYMERASE_II_PROMOTER_IN_RESPONSE	29	0.484	2.228	0	0.004011559	0.4	406	tags=55%, lis
GOBP_POSITIVE_REGULATION_OF_IMMUNE_RESPONSE	145	0.320	2.225	0	0.004053305	0.408	417	tags=36%, lis
REACTOME_TCF_DEPENDENT_SIGNALING_IN_RESPONSE_TO_WNT	37	0.455	2.217	0	0.004344885	0.434	253	tags=41%, lis
GOBP_RESPONSE_TO_BIOTIC_STIMULUS	266	0.283	2.214	0	0.004396458	0.443	273	tags=25%, lis
HALLMARK_INTERFERON_ALPHA_RESPONSE	32	0.487	2.214	0	0.004387852	0.445	374	tags=44%, lis
GOBP_NEUTROPHIL_CHEMOTAXIS	16	0.589	2.210	0	0.004506022	0.459	116	tags=38%, lis
GOBP_REGULATION_OF_HEMATOPOIETIC_PROGENITOR_CELL_DIFFERENTIATION	34	0.451	2.210	0	0.004497044	0.462	253	tags=41%, lis
GOCC_RIBOSOMAL_SUBUNIT	18	0.545	2.191	0	0.005330295	0.526	646	tags=78%, lis
HALLMARK_GLYCOLYSIS	22	0.525	2.190	0	0.005357583	0.53	373	tags=55%, lis
GOBP_ANTIGEN_PROCESSING_AND_PRESENTATION	73	0.356	2.176	0	0.006048787	0.573	437	tags=42%, lis
GOBP_NIK_NF_KAPPAB_SIGNALING	47	0.412	2.175	0	0.006069636	0.582	283	tags=38%, lis
GOBP_MITOCHONDRIAL_TRANSPORT	52	0.399	2.168	0	0.006398700	0.604	603	tags=62%, lis
GOBP_CELLULAR_KETONE_METABOLIC_PROCESS	41	0.417	2.167	0	0.006395750	0.606	253	tags=37%, lis
REACTOME_TRANSCRIPTIONAL_REGULATION_BY_RUNX3	31	0.478	2.163	0	0.006580075	0.617	253	tags=45%, lis
REACTOME_SIGNALING_BY_ROBO_RECEPTORS	40	0.426	2.160	0	0.006768962	0.628	559	tags=55%, lis
GOBP_REGULATION_OF_HEMATOPOIETIC_STEM_CELL_DIFFERENTIATION	31	0.459	2.158	0	0.006843664	0.636	253	tags=42%, lis
GOBP_REGULATION_OF_ANIMAL_ORGAN_MORPHOGENESIS	31	0.448	2.151	0.002044989	0.007243379	0.654	303	tags=45%, lis
GOBP_HUMORAL_IMMUNE_RESPONSE	31	0.457	2.147	0.001923076	0.007407963	0.662	249	tags=42%, lis
GOBP_POSITIVE_REGULATION_OF_CANONICAL_WNT_SIGNALING_PATHWAY	37	0.428	2.145	0	0.007476199	0.67	406	tags=49%, lis
REACTOME_SIGNALING_BY_THE_B_CELL_RECEPTOR_BCR	49	0.395	2.142	0	0.007556676	0.678	353	tags=45%, lis
GOCC_RIBOSOME	24	0.501	2.139	0.001984127	0.007728290	0.69	646	tags=71%, lis
GOBP_LEUKOCYTE_CHEMOTAXIS	34	0.438	2.133	0.001956947	0.008020668	0.702	123	tags=26%, lis
GOBP_RESPONSE_TO_BACTERIUM	100	0.332	2.129	0	0.008335267	0.718	220	tags=26%, lis
GOBP_GRANULOCYTE_CHEMOTAXIS	17	0.567	2.123	0	0.008630603	0.731	116	tags=35%, lis
REACTOME_MITOTIC_G2_G2_M_PHASES	45	0.398	2.113	0	0.009354193	0.77	410	tags=44%, lis
GOBP_REGULATION_OF_CELLULAR_KETONE_METABOLIC_PROCESS	36	0.433	2.109	0.001945525	0.00955019	0.775	253	tags=39%, lis
HALLMARK_MYC_TARGETS_V1	51	0.393	2.097	0.001912045	0.010513369	0.802	412	tags=45%, lis
GOMF_PROTON_TRANSMEMBRANE_TRANSPORTER_ACTIVITY	31	0.446	2.090	0	0.011022341	0.817	641	tags=68%, lis
REACTOME_DOWNSTREAM_SIGNALING_EVENTS_OF_B_CELL_RECEPTOR_BCR	37	0.420	2.087	0	0.011153261	0.82	283	tags=43%, lis
GOCC_BLOOD_MICROPARTICLE	20	0.509	2.081	0.003968254	0.011685085	0.84	198	tags=45%, lis
REACTOME_G2_M_CHECKPOINTS	35	0.421	2.081	0.001912045	0.01162008	0.841	344	tags=46%, lis
REACTOME_DISORDERS_OF_TRANSMEMBRANE_TRANSPORTERS	32	0.430	2.065	0	0.013179595	0.881	253	tags=41%, lis
GOBP_ATP_METABOLIC_PROCESS	75	0.341	2.063	0	0.013292149	0.884	647	tags=59%, lis
REACTOME_SIGNALING_BY_HEDGEHOG	35	0.425	2.051	0.001953125	0.014696130	0.909	283	tags=40%, lis
GOBP_POSITIVE_REGULATION_OF_WNT_SIGNALING_PATHWAY	43	0.395	2.051	0	0.014683287	0.909	253	tags=37%, lis
GOBP_REGULATION_OF_DNA_TEMPLATED_TRANSCRIPTION_IN_RESPONSE_TO_STRESS	38	0.411	2.050	0	0.014723976	0.91	344	tags=45%, lis
REACTOME_METABOLISM_OF_AMINO_ACIDS_AND_DERIVATIVES	38	0.411	2.048	0	0.014805349	0.912	406	tags=50%, lis
GOBP_NUCLEOSIDE_TRIPHOSPHATE_BIOSYNTHETIC_PROCESS	23	0.474	2.041	0.003960396	0.015538000	0.926	603	tags=74%, lis
GOBP_FC_EPSILON_RECEPTOR_SIGNALING_PATHWAY	47	0.375	2.041	0	0.015468384	0.928	283	tags=38%, lis
GOBP_B_CELL_MEDIATED_IMMUNITY	28	0.438	2.034	0	0.016105792	0.936	198	tags=36%, lis
GOBP_NEUTROPHIL_MIGRATION	20	0.505	2.032	0	0.016384715	0.939	116	tags=30%, lis
GOBP_MYELOID_LEUKOCYTE_MIGRATION	35	0.418	2.028	0.004115226	0.016801601	0.943	123	tags=26%, lis
GOMF_OXIDOREDUCTASE_ACTIVITY	99	0.312	2.026	0	0.016971571	0.943	355	tags=30%, lis
GOBP_IMMUNE_EFFECTOR_PROCESS	274	0.254	2.016	0	0.018290885	0.962	417	tags=30%, lis
REACTOME_UCH_PROTEINASES	36	0.406	2.016	0	0.018206134	0.962	406	tags=47%, lis

REACTOME_CLEC7A_DECTIN_1_SIGNALING	46	0.381	2.003	0.00207039З	0.02018967	0.977	413	tags=46%, lis
GOBP_REGULATION_OF_CELL_CYCLE_G2_M_PHASE_TRANSITION	49	0.365	2.002	0.001988071	0.02011595	0.977	410	tags=43%, lis
GOBP_FC_RECEPTOR_SIGNALING_PATHWAY	70	0.334	1.999	0.001818181	0.02061812	0.979	417	tags=41%, lis
GOBP_REGULATION_OF_IMMUNE_RESPONSE	189	0.270	1.997	0	0.02075204	0.98	417	tags=33%, lis
GOBP_HEMATOPOIETIC_STEM_CELL_DIFFERENTIATION	33	0.419	1.990	0.00395256	0.02173109	0.986	253	tags=39%, lis
REACTOME_MAPK6_MAPK4_SIGNALING	33	0.415	1.985	0.00404040	0.02258035	0.987	253	tags=39%, lis
GOBP_POSITIVE_REGULATION_OF_IMMUNE_SYSTEM_PROCESS	191	0.264	1.981	0	0.02314244	0.99	417	tags=31%, lis
GOBP_T_CELL_RECEPTOR_SIGNALING_PATHWAY	51	0.356	1.975	0	0.02410867	0.991	283	tags=33%, lis
REACTOME_C_TYPE_LECTIN_RECEPTORS_CLRS	49	0.382	1.974	0.00579150	0.02415469	0.991	413	tags=45%, lis
GOBP_REGULATION_OF_STEM_CELL_DIFFERENTIATION	36	0.401	1.968	0	0.02518354	0.993	290	tags=39%, lis
KEGG_ANTIGEN_PROCESSING_AND_PRESENTATION	19	0.499	1.967	0.00598802	0.025109341	0.993	163	tags=37%, lis
GOBP_HEMATOPOIETIC_PROGENITOR_CELL_DIFFERENTIATION	43	0.383	1.961	0.00404858	0.02609654	0.994	253	tags=35%, lis
GOBP_GRANULOCYTE_MIGRATION	23	0.462	1.956	0.00387596	0.02726307	0.995	116	tags=26%, lis
GOMF_ELECTRON_TRANSFER_ACTIVITY	36	0.393	1.949	0.00385356	0.02842530	0.995	835	tags=75%, lis
GOBP_REGULATION_OF_RESPONSE_TO_EXTERNAL_STIMULUS	173	0.268	1.941	0	0.030376311	0.996	413	tags=32%, lis
GOMF_OXIDOREDUCTASE_ACTIVITY_ACTING_ON_NAD_P_H	17	0.509	1.933	0.00405679	0.03190431	0.999	809	tags=82%, lis
GOBP_IMMUNE_RESPONSE_REGULATING_SIGNALING_PATHWAY	104	0.295	1.932	0	0.03193276	0.999	417	tags=36%, lis
HALLMARK_ALLOGRAFT_REJECTION	47	0.367	1.932	0	0.03180793	0.999	188	tags=26%, lis
GOBP_REGULATION_OF_MRNA_CATABOLIC_PROCESS	62	0.330	1.928	0.00909090	0.03244974	0.999	409	tags=40%, lis
GOCC_RIBONUCLEOPROTEIN_COMPLEX	113	0.283	1.925	0	0.03296349	0.999	656	tags=49%, lis
GOBP_CELLULAR_RESPIRATION	44	0.372	1.924	0.00408163	0.03303564	0.999	647	tags=64%, lis
GOBP_ELECTRON_TRANSPORT_CHAIN	46	0.365	1.924	0.00192307	0.03296982	0.999	841	tags=74%, lis
GOCC_MICROBODY	15	0.522	1.921	0.00578034	0.03361947	0.999	181	tags=47%, lis
GOBP_RESPONSE_TO_CYTOKINE	233	0.247	1.915	0	0.03485682	1	349	tags=27%, lis
REACTOME_FC_EPSILON_RECEPTOR_FCERI_SIGNALING	55	0.342	1.910	0.00196078	0.03574423	1	353	tags=38%, lis
HALLMARK_PEROXISOME	15	0.521	1.910	0.018556701	0.03560818	1	388	tags=60%, lis
GOMF_LIGASE_ACTIVITY	24	0.438	1.905	0.01014198	0.03681265	1	606	tags=67%, lis
GOBP_CELL_KILLING	20	0.472	1.905	0.01257861	0.03674787	1	1052	tags=95%, lis
GOBP_PROTON_TRANSMEMBRANE_TRANSPORT	35	0.387	1.892	0.00610997	0.03990717	1	653	tags=60%, lis
GOBP_CELLULAR_RESPONSE_TO_OXYGEN_LEVELS	53	0.340	1.885	0.010245901	0.04193195	1	502	tags=47%, lis
REACTOME_HOST_INTERACTIONS_OF_HIV_FACTORS	42	0.367	1.882	0.00798403	0.04249818	1	437	tags=45%, lis
REACTOME_BETA_CATENIN_INDEPENDENT_WNT_SIGNALING	43	0.357	1.871	0.00963391	0.04557207	1	253	tags=35%, lis
REACTOME_INNATE_IMMUNE_SYSTEM	258	0.238	1.861	0	0.04872188	1	417	tags=29%, lis
GOBP_INFLAMMATORY_RESPONSE	128	0.266	1.854	0.00199203	0.0509521	1	267	tags=24%, lis
REACTOME_NEDDYLATION	50	0.339	1.850	0.00401606	0.05179156	1	406	tags=40%, lis
REACTOME_UB_SPECIFIC_PROCESSING_PROTEASES	48	0.342	1.848	0.00919117	0.05234677	1	406	tags=42%, lis
GOBP_LEUKOCYTE_MEDIATED_CYTOTOXICITY	18	0.469	1.836	0.01252609	0.05642138	1	1052	tags=94%, lis
HP_INCREASED_SERUM_LACTATE	26	0.420	1.834	0.02096436	0.05683095	1	647	tags=62%, lis
GOBP_NUCLEOSIDE_TRIPHOSPHATE_METABOLIC_PROCESS	26	0.414	1.833	0.01224489	0.05691464	1	603	tags=65%, lis
GOBP_RESPONSE_TO_VIRUS	72	0.307	1.831	0.00371747	0.05737387	1	426	tags=35%, lis
GOBP_RESPONSE_TO_INTERFERON_GAMMA	40	0.353	1.822	0.00785854	0.06054534	1	318	tags=33%, lis
GOBP_RIBONUCLEOSIDE_TRIPHOSPHATE_BIOSYNTHETIC_PROCESS	20	0.441	1.820	0.02419354	0.06118426	1	603	tags=70%, lis
GOBP_MORPHOGENESIS_OF_AN_EPITHELIUM	65	0.308	1.815	0.01100917	0.06284048	1	266	tags=31%, lis
GOBP_CELL_CYCLE_G2_M_PHASE_TRANSITION	53	0.330	1.811	0.005725191	0.06415688	1	410	tags=40%, lis
GOMF_HYDROLASE_ACTIVITY_ACTING_ON_ACID_ANHYDRIDES	115	0.264	1.811	0	0.06389196	1	576	tags=43%, lis
GOBP_LEUKOCYTE_MEDIATED_IMMUNITY	188	0.244	1.803	0	0.06679924	1	416	tags=29%, lis
KEGG_CARDIAC_MUSCLE_CONTRACTION	17	0.481	1.803	0.01691331	0.06662939	1	741	tags=76%, lis
GOBP_NEGATIVE_REGULATION_OF_CANONICAL_WNT_SIGNALING_PATHWAY	39	0.365	1.796	0.00408997	0.06951097	1	300	tags=38%, lis
GOCC_FICOLIN_1_RICH_GRANULE_LUMEN	33	0.360	1.795	0.01188118	0.06957336	1	326	tags=33%, lis
REACTOME_CELLULAR_RESPONSES_TO_EXTERNAL_STIMULI	142	0.258	1.792	0.00181488	0.0704258	1	406	tags=30%, lis
GOBP_CELL_CHEMOTAXIS	43	0.345	1.792	0.011673151	0.07024976	1	123	tags=21%, lis
GOBP_CELLULAR_AMINO_ACID_METABOLIC_PROCESS	44	0.338	1.789	0.00787401	0.07111437	1	299	tags=36%, lis
GOCC_MITOCHONDRION	232	0.235	1.788	0	0.07120534	1	655	tags=46%, lis
REACTOME_CELL_CYCLE_CHECKPOINTS	45	0.343	1.788	0.01174168	0.07126216	1	344	tags=38%, lis
REACTOME_SIGNALING_BY_WNT	59	0.317	1.776	0.007797271	0.07637041	1	283	tags=32%, lis
REACTOME_IMMUNOREGULATORY_INTERACTIONS_BETWEEN_A_LYMPHOID_AND_A_NON_LYMPH	17	0.452	1.773	0.01348747	0.07785107	1	203	tags=35%, lis
GOBP_ATP_BIOSYNTHETIC_PROCESS	18	0.445	1.771	0.02777777	0.0781708	1	603	tags=72%, lis
REACTOME_MITOTIC_METAPHASE_AND_ANAPHASE	48	0.335	1.764	0.00760456	0.08132762	1	253	tags=31%, lis
GOBP_B_CELL_RECEPTOR_SIGNALING_PATHWAY	21	0.414	1.752	0.02390438	0.08724971	1	587	tags=57%, lis
GOBP_PROTEIN_TARGETING_TO_MITOCHONDRION	18	0.446	1.751	0.01960784	0.08747049	1	548	tags=61%, lis
GOCC_VACUOLAR_LUMEN	32	0.374	1.748	0.01829268	0.08890423	1	128	tags=22%, lis
GOBP_ANTIGEN_PROCESSING_AND_PRESENTATION_OF_PEPTIDE_OR_POLYSACCHARIDE_ANTIGEN	23	0.401	1.747	0.01377952	0.08921802	1	738	tags=65%, lis
GOBP_NEGATIVE_REGULATION_OF_WNT_SIGNALING_PATHWAY	44	0.336	1.746	0.01737451	0.08918588	1	300	tags=36%, lis
REACTOME_GOLGI_ASSOCIATED_VESICLE_BIOGENESIS	16	0.454	1.733	0.01458333	0.09647243	1	672	tags=69%, lis
GOBP_REGULATION_OF_IMMUNE_SYSTEM_PROCESS	270	0.221	1.731	0	0.0970682	1	417	tags=29%, lis
REACTOME_CLASS_A_1_RHODOPSIN_LIKE_RECEPTORS	16	0.467	1.727	0.02169197	0.09885843	1	116	tags=25%, lis
REACTOME_PTEN_REGULATION	47	0.323	1.719	0.01649484	0.10373093	1	278	tags=34%, lis
GOCC_MEMBRANE_PROTEIN_COMPLEX	197	0.231	1.716	0	0.10467925	1	625	tags=44%, lis
HP_ABNORMALITY_OF_THE_PERIORBITAL_REGION	29	0.370	1.711	0.01984127	0.10772072	1	348	tags=31%, lis
GOBP_MYELOID_LEUKOCYTE_ACTIVATION	155	0.241	1.709	0.00183486	0.10829274	1	416	tags=29%, lis
REACTOME_APOPTOSIS	55	0.308	1.706	0.00739371	0.10967155	1	409	tags=40%, lis
GOCC_ENVELOPE	210	0.225	1.706	0.00173913	0.10920354	1	655	tags=45%, lis
REACTOME_SIGNALING_BY_NOTCH	48	0.313	1.706	0.00968992	0.1087881	1	344	tags=35%, lis
GOBP_REGULATION_OF_MRNA_METABOLIC_PROCESS	90	0.268	1.705	0.00389105	0.10863723	1	419	tags=36%, lis
GOBP_NON_CANONICAL_WNT_SIGNALING_PATHWAY	40	0.339	1.703	0.01646090	0.10977621	1	253	tags=33%, lis
GOBP_NEGATIVE_REGULATION_OF_DEVELOPMENTAL_GROWTH	16	0.439	1.699	0.02839756	0.11166391	1	386	tags=44%, lis
GOBP_RESPONSE_TO_OXYGEN_LEVELS	72	0.281	1.692	0.0056926	0.1165167	1	376	tags=35%, lis
GOBP_MAINTENANCE_OF_LOCATION_IN_CELL	32	0.356	1.686	0.02169625	0.11992106	1	187	tags=28%, lis
GOBP_RIBONUCLEOSIDE_TRIPHOSPHATE_METABOLIC_PROCESS	24	0.391	1.682	0.03745318	0.12228651	1	603	tags=63%, lis

GOBP_REGULATION_OF_RESPONSE_TO_CYTOKINE_STIMULUS	39	0.341	1.670	0.020242915	0.1296777	1	413	tags=38%, lis
GOBP_DEFENSE_RESPONSE_TO_VIRUS	52	0.307	1.669	0.017441861	0.12992448	1	413	tags=33%, lis
REACTOME_ADAPTIVE_IMMUNE_SYSTEM	172	0.228	1.669	0.007662835	0.12949209	1	284	tags=23%, lis
REACTOME_NERVOUS_SYSTEM_DEVELOPMENT	88	0.267	1.668	0.009823183	0.13006565	1	559	tags=43%, lis
REACTOME_PROGRAMMED_CELL_DEATH	62	0.292	1.666	0.011538462	0.13087095	1	409	tags=39%, lis
HALLMARK_TNFA_SIGNALING_VIA_NFKB	72	0.281	1.665	0.009652509	0.13168693	1	243	tags=25%, lis
REACTOME_TRANSCRIPTIONAL_REGULATION_BY_RUNX1	50	0.306	1.663	0.026369167	0.13212518	1	253	tags=32%, lis
GOBP_TRANSLATIONAL_ELONGATION	19	0.408	1.658	0.036217302	0.13593331	1	648	tags=68%, lis
HP_POLYHYDRAMNIOS	18	0.415	1.652	0.03168317	0.1400809	1	317	tags=33%, lis
GOBP_INNER_MITOCHONDRIAL_MEMBRANE_ORGANIZATION	16	0.443	1.651	0.046908315	0.14051707	1	603	tags=69%, lis
GOBP_POSITIVE_REGULATION_OF_LEUKOCYTE_CHEMOTAXIS	15	0.454	1.650	0.048523206	0.14089184	1	123	tags=33%, lis
KEGG_ALZHEIMERS_DISEASE	55	0.299	1.646	0.018292682	0.14351812	1	785	tags=67%, lis
GOBP_ORGANIC_CYCLIC_COMPOUND_CATABOLIC_PROCESS	113	0.252	1.641	0.013084112	0.14677137	1	359	tags=32%, lis
GOCC_MITOCHONDRIAL_MATRIX	50	0.299	1.640	0.03168317	0.14686859	1	646	tags=56%, lis
GOBP_MITOCHONDRION_ORGANIZATION	103	0.251	1.639	0.015384615	0.14765802	1	548	tags=44%, lis
GOBP_TRANSMEMBRANE_TRANSPORT	186	0.221	1.636	0.003831417	0.1489723	1	611	tags=42%, lis
GOCC_EXTERNAL_SIDE_OF_PLASMA_MEMBRANE	51	0.299	1.636	0.024096385	0.14868528	1	262	tags=29%, lis
GOMF_RECEPTOR_REGULATOR_ACTIVITY	25	0.378	1.635	0.02584493	0.1489633	1	116	tags=20%, lis
HP_EPICANTHUS	62	0.286	1.635	0.013916501	0.1483918	1	385	tags=31%, lis
GOBP_INTERFERON_GAMMA_MEDIATED_SIGNALING_PATHWAY	24	0.374	1.623	0.04527559	0.15844586	1	84	tags=21%, lis
HP_CONGESTIVE_HEART_FAILURE	32	0.342	1.618	0.037190083	0.16208003	1	128	tags=19%, lis
GOBP_LYMPHOCYTE_MEDIATED_IMMUNITY	56	0.285	1.618	0.02131783	0.16144691	1	198	tags=23%, lis
REACTOME_PROTEIN_LOCALIZATION	26	0.363	1.615	0.031311154	0.1637819	1	532	tags=50%, lis
GOBP_MYELOID_LEUKOCYTE_MEDIATED_IMMUNITY	147	0.229	1.612	0.011009174	0.16559236	1	416	tags=29%, lis
GOMF_ENDOPEPTIDASE_ACTIVITY	46	0.304	1.610	0.03373016	0.16620864	1	253	tags=28%, lis
HP_PRE_CAPILLARY_PULMONARY_HYPERTENSION	19	0.408	1.604	0.043396227	0.17162411	1	731	tags=74%, lis
HP_SPLENOMEGALY	80	0.261	1.603	0.014981274	0.17194942	1	284	tags=24%, lis
HP_LACTIC_ACIDOSIS	19	0.404	1.602	0.05811623	0.17180282	1	458	tags=53%, lis
GOMF_GTPASE_ACTIVITY	40	0.316	1.596	0.04191617	0.17748208	1	576	tags=43%, lis
GOBP_INTRINSIC_APOPTOTIC_SIGNALING_PATHWAY_IN_RESPONSE_TO_DNA_DAMAGE	22	0.379	1.588	0.046747968	0.1846278	1	426	tags=41%, lis
GOBP_NEGATIVE_REGULATION_OF_CELL_CYCLE_PHASE_TRANSITION	52	0.285	1.587	0.023166023	0.18561387	1	363	tags=37%, lis
GOBP_INTRINSIC_APOPTOTIC_SIGNALING_PATHWAY	68	0.271	1.586	0.023076924	0.18562774	1	440	tags=34%, lis
REACTOME_HIV_INFECTION	57	0.282	1.586	0.033009708	0.18499045	1	502	tags=40%, lis
GOBP_B_CELL_PROLIFERATION	20	0.396	1.584	0.042471044	0.1859769	1	153	tags=30%, lis
GOBP_NUCLEOSIDE_PHOSPHATE_BIOSYNTHETIC_PROCESS	46	0.296	1.584	0.026923077	0.18536572	1	624	tags=54%, lis
GOBP_CELL_ACTIVATION_INVOLVED_IN_IMMUNE_RESPONSE	181	0.216	1.580	0.00907441	0.18903379	1	416	tags=28%, lis
HP_BULBOUS_NOSE	18	0.399	1.579	0.036144577	0.18945237	1	358	tags=50%, lis
REACTOME_MHC_CLASS_II_ANTIGEN_PRESENTATION	24	0.364	1.577	0.05544933	0.19084509	1	757	tags=63%, lis
GOMF_PROTEIN_HOMODIMERIZATION_ACTIVITY	91	0.244	1.572	0.024482109	0.19526967	1	308	tags=23%, lis
GOBP_REGULATION_OF_LEUKOCYTE_CHEMOTAXIS	18	0.402	1.568	0.06012024	0.19803736	1	123	tags=28%, lis
GOBP_RESPONSE_TO_REACTIVE_OXYGEN_SPECIES	45	0.296	1.568	0.03952569	0.1980027	1	280	tags=24%, lis
GOBP_RESPONSE_TO_HYDROGEN_PEROXIDE	30	0.335	1.568	0.05284553	0.1973908	1	357	tags=30%, lis
HP_ABNORMALITY_OF_FLUID_REGULATION	76	0.260	1.566	0.03538175	0.19837525	1	353	tags=29%, lis
GOCC_AZUROPHIL_GRANULE_LUMEN	22	0.374	1.563	0.0655106	0.20064317	1	128	tags=23%, lis
GOCC_VESICLE_LUMEN	64	0.269	1.559	0.031434186	0.2047819	1	413	tags=31%, lis
REACTOME_NEUTROPHIL_DEGRANULATION	130	0.227	1.558	0.018726591	0.20494358	1	416	tags=28%, lis
GOBP_RIBOSE_PHOSPHATE_BIOSYNTHETIC_PROCESS	41	0.306	1.558	0.036511157	0.2047331	1	624	tags=56%, lis
GOCC_OUTER_MEMBRANE	29	0.335	1.556	0.058935363	0.20570295	1	571	tags=45%, lis
GOBP_PATTERN_RECOGNITION_RECEPTOR_SIGNALING_PATHWAY	51	0.286	1.555	0.04375	0.2060436	1	374	tags=33%, lis
REACTOME_TRANSPORT_OF_SMALL_MOLECULES	101	0.238	1.553	0.013232514	0.20777921	1	580	tags=42%, lis
GOBP_GENERATION_OF_PRECURSOR_METABOLITES_AND_ENERGY	102	0.237	1.551	0.018	0.20861487	1	653	tags=49%, lis
GOBP_POSITIVE_REGULATION_OF_B_CELL_ACTIVATION	19	0.392	1.551	0.073267326	0.20794277	1	198	tags=32%, lis
HP_SKIN_RASH	33	0.324	1.550	0.053892214	0.20821595	1	315	tags=33%, lis
REACTOME_GPCR_LIGAND_BINDING	20	0.377	1.545	0.06507592	0.2140496	1	206	tags=25%, lis
GOBP_FC_RECEPTOR_MEDIATED_STIMULATORY_SIGNALING_PATHWAY	31	0.332	1.544	0.05	0.21403593	1	500	tags=48%, lis
GOMF_AMIDE_BINDING	49	0.286	1.542	0.024856597	0.2156376	1	188	tags=24%, lis
REACTOME_SIGNALING_BY_INTERLEUKINS	117	0.231	1.541	0.027075812	0.21558307	1	349	tags=28%, lis
REACTOME_M_PHASE	76	0.254	1.539	0.03468208	0.21694979	1	219	tags=24%, lis
GOBP_TISSUE_MORPHOGENESIS	72	0.261	1.536	0.036437247	0.21966615	1	266	tags=28%, lis
HP_ABNORMALITY_OF_PULMONARY_CIRCULATION	23	0.360	1.532	0.051587302	0.2240311	1	431	tags=48%, lis
GOBP_CANONICAL_WNT_SIGNALING_PATHWAY	59	0.270	1.532	0.067729086	0.22334988	1	253	tags=29%, lis
HALLMARK_P53_PATHWAY	48	0.279	1.530	0.05019305	0.22516613	1	255	tags=29%, lis
GOMF_PASSIVE_TRANSMEMBRANE_TRANSPORTER_ACTIVITY	31	0.323	1.527	0.056277055	0.22792195	1	603	tags=52%, lis
GOBP_PROTEIN_TARGETING	51	0.280	1.524	0.04743083	0.23011878	1	548	tags=45%, lis
GOCC_SIDE_OF_MEMBRANE	89	0.241	1.521	0.023715414	0.23304334	1	267	tags=25%, lis
GOMF_IRON_ION_BINDING	18	0.384	1.521	0.080078125	0.23238097	1	47	tags=11%, lis
GOBP_RESPONSE_TO_ABIOTIC_STIMULUS	193	0.205	1.515	0.012727273	0.23967366	1	411	tags=30%, lis
REACTOME_CLASS_I_MHC_MEDIATED_ANTIGEN_PROCESSING_PRESENTATION	95	0.234	1.514	0.02259887	0.24001804	1	547	tags=38%, lis
HP_UNUSUAL_INFECTION_BY_ANATOMICAL_SITE	22	0.357	1.509	0.074152544	0.2449752	1	155	tags=27%, lis
GOBP_RESPONSE_TO_TEMPERATURE_STIMULUS	38	0.300	1.508	0.049115915	0.24535047	1	192	tags=24%, lis
GOBP_MUSCLE_CELL_DIFFERENTIATION	42	0.292	1.508	0.042168673	0.24484123	1	83	tags=14%, lis
GOCC_OXIDOREDUCTASE_COMPLEX	26	0.342	1.501	0.06175299	0.25235057	1	841	tags=77%, lis
HP_PNEUMONIA	43	0.287	1.494	0.0591716	0.26035538	1	587	tags=44%, lis
HP_DISPLACEMENT_OF_THE_URETHRAL_MEATUS	38	0.292	1.494	0.07581967	0.2598948	1	364	tags=29%, lis
GOBP_MITOCHONDRIAL_RESPIRATORY_CHAIN_COMPLEX_ASSEMBLY	23	0.353	1.493	0.087128714	0.2602217	1	723	tags=65%, lis
GOCC_FICOLIN_1_RICH_GRANULE	52	0.271	1.493	0.05232558	0.2596416	1	406	tags=31%, lis
REACTOME_TRANS_GOLGI_NETWORK_VESICLE_BUDDING	20	0.374	1.493	0.068359375	0.2589037	1	672	tags=60%, lis
GOBP_REGULATION_OF_WNT_SIGNALING_PATHWAY	73	0.245	1.492	0.0513834	0.25876838	1	255	tags=26%, lis
HALLMARK_APOPTOSIS	45	0.291	1.490	0.08213552	0.26124898	1	409	tags=38%, lis

HP_PIGMENTARY_RETINOPATHY	15	0.400	1.486	0.089361705	0.2650853	1	647	tags=60%, lis
HP_ABNORMALITY_OF_HUMORAL_IMMUNITY	70	0.248	1.485	0.062992126	0.26544398	1	374	tags=30%, lis
GOBP_POSITIVE_REGULATION_OF_CATION_TRANSMEMBRANE_TRANSPORT	15	0.403	1.482	0.093877554	0.2694157	1	353	tags=33%, lis
REACTOME_CYTOKINE_SIGNALING_IN_IMMUNE_SYSTEM	166	0.207	1.481	0.025547445	0.26992634	1	318	tags=23%, lis
GOBP_MITOCHONDRIAL_MEMBRANE_ORGANIZATION	36	0.309	1.479	0.07471264	0.27099726	1	503	tags=47%, lis
REACTOME_DEUBIQUITINATION	72	0.255	1.478	0.058027077	0.27189857	1	253	tags=26%, lis
GOBP_CELL_ACTIVATION	274	0.189	1.477	0.010948905	0.27222097	1	416	tags=27%, lis
REACTOME_TRANSLATION	38	0.297	1.477	0.057471264	0.2717307	1	648	tags=50%, lis
GOBP_PROTEIN_MODIFICATION_BY_SMALL_PROTEIN_REMOVAL	80	0.247	1.477	0.048076924	0.2713149	1	253	tags=26%, lis
HP_INCREASED_CSF_LACTATE	18	0.374	1.476	0.08285164	0.27065566	1	841	tags=72%, lis
REACTOME_ANTIGEN_PROCESSING_UBIQUITINATION_PROTEASOME_DEGRADATION	71	0.247	1.476	0.036053132	0.26984954	1	547	tags=39%, lis
GOBP_RESPONSE_TO_OXIDATIVE_STRESS	80	0.242	1.472	0.044061303	0.27485177	1	357	tags=25%, lis
GOBP_PROTEIN_HOMOOLIGOMERIZATION	21	0.347	1.471	0.09338521	0.27611664	1	448	tags=38%, lis
GOBP_RNA_CATABOLIC_PROCESS	88	0.234	1.468	0.04770642	0.278459	1	359	tags=30%, lis
HP_BRONCHITIS	17	0.378	1.468	0.064386316	0.27854592	1	163	tags=29%, lis
HP_ABNORMAL_ERYTHROCYTE_MORPHOLOGY	108	0.222	1.467	0.05	0.2782398	1	290	tags=22%, lis
GOMF_CYTOKINE_RECEPTOR_BINDING	27	0.325	1.466	0.06534653	0.2789712	1	116	tags=22%, lis
HP_FAILURE_TO_THRIVE	130	0.211	1.461	0.02268431	0.2851055	1	316	tags=22%, lis
GOCC_ORGANELLE_ENVELOPE_LUMEN	22	0.347	1.459	0.092337914	0.2867826	1	285	tags=32%, lis
HP_ABNORMALITY_OF_THE_LYMPHATIC_SYSTEM	117	0.218	1.458	0.028625954	0.28710702	1	284	tags=21%, lis
GOMF_PEPTIDE_BINDING	39	0.282	1.456	0.077071294	0.28966388	1	188	tags=23%, lis
GOBP_RESPONSE_TO_TYPE_I_INTERFERON	24	0.335	1.455	0.076771654	0.28971925	1	149	tags=21%, lis
GOBP_INTRINSIC_APOPTOTIC_SIGNALING_PATHWAY_BY_P53_CLASS_MEDIATOR	18	0.370	1.454	0.099029124	0.28994036	1	413	tags=44%, lis
REACTOME_INTERFERON_GAMMA_SIGNALING	23	0.348	1.454	0.085192695	0.28918195	1	318	tags=30%, lis
GOBP_CELLULAR_TRANSITION_METAL_ION_HOMEOSTASIS	23	0.333	1.450	0.086065575	0.2938102	1	378	tags=39%, lis
GOMF_ION_TRANSMEMBRANE_TRANSPORTER_ACTIVITY	85	0.232	1.446	0.057971016	0.29844052	1	641	tags=46%, lis
GOBP_REGULATION_OF_RESPONSE_TO_STRESS	252	0.188	1.446	0.014519056	0.29813313	1	371	tags=24%, lis
HP_ABNORMALITY_OF_ACID_BASE_HOMEOSTASIS	44	0.289	1.446	0.07920792	0.2972789	1	458	tags=41%, lis
HP_ABNORMAL_INFLAMMATORY_RESPONSE	180	0.196	1.444	0.031496063	0.29899102	1	358	tags=24%, lis
REACTOME_MAPK_FAMILY_SIGNALING_CASCADES	67	0.242	1.439	0.06122449	0.3063822	1	253	tags=25%, lis
HP_ABNORMAL_CIRCULATING_CARBOXYLIC_ACID_CONCENTRATION	26	0.321	1.438	0.086444005	0.30683914	1	316	tags=35%, lis
GOBP_REACTIVE_OXYGEN_SPECIES_METABOLIC_PROCESS	47	0.268	1.436	0.07495069	0.30767134	1	388	tags=28%, lis
HP_SHORT_TOE	15	0.398	1.435	0.1	0.30891126	1	2	tags=7%, list-
GOBP_POSTTRANSCRIPTIONAL_REGULATION_OF_GENE_EXPRESSION	128	0.212	1.434	0.040733196	0.3098414	1	409	tags=30%, lis
HP_VASCULITIS	19	0.357	1.432	0.09406953	0.31048772	1	405	tags=42%, lis
GOBP_TOLL_LIKE_RECEPTOR_SIGNALING_PATHWAY	36	0.290	1.431	0.07602339	0.31184366	1	344	tags=33%, lis
HP_PALLOR	19	0.360	1.426	0.124497995	0.3179576	1	302	tags=32%, lis
REACTOME_IRON_UPTAKE_AND_TRANSPORT	20	0.344	1.425	0.09504132	0.31932253	1	566	tags=55%, lis
HP_ABNORMALITY_OF_THE_SPLEEN	95	0.223	1.424	0.052930057	0.31923467	1	284	tags=21%, lis
REACTOME_METABOLISM_OF_RNA	122	0.210	1.422	0.07444668	0.3213429	1	366	tags=26%, lis
GOBP_TRANSITION_METAL_ION_HOMEOSTASIS	27	0.324	1.422	0.09533469	0.3207663	1	378	tags=37%, lis
HP_NEOPLASM_OF_THE_GASTROINTESTINAL_TRACT	41	0.275	1.421	0.0952381	0.32173124	1	153	tags=20%, lis
HP_ABNORMAL_AORTIC_VALVE_MORPHOLOGY	18	0.366	1.420	0.11111111	0.32204422	1	374	tags=44%, lis
GOBP_CALCIUM_ION_TRANSPORT_INTO_CYTOSOL	15	0.378	1.418	0.11134454	0.3247075	1	357	tags=40%, lis
REACTOME_CELL_CYCLE_MITOTIC	92	0.222	1.417	0.07450981	0.32440904	1	253	tags=23%, lis
GOBP_PROTEIN_LOCALIZATION_TO_MITOCHONDRION	28	0.316	1.414	0.11500975	0.32883763	1	548	tags=50%, lis
REACTOME_G_ALPHA_I_SIGNALLING_EVENTS	32	0.296	1.413	0.1064257	0.3298148	1	206	tags=22%, lis
GOBP_CELL_CELL_SIGNALING	176	0.194	1.412	0.037383176	0.32933578	1	255	tags=20%, lis
GOBP_POSITIVE_REGULATION_OF_CHEMOTAXIS	22	0.334	1.412	0.11608961	0.32867506	1	123	tags=23%, lis
HP_LETHARGY	16	0.378	1.406	0.12704918	0.33636117	1	458	tags=50%, lis
GOBP_SUPEROXIDE_METABOLIC_PROCESS	16	0.368	1.403	0.11623246	0.3407025	1	315	tags=31%, lis
REACTOME_INTRACELLULAR_SIGNALING_BY_SECOND_MESSENGERS	74	0.234	1.402	0.069767445	0.3406931	1	278	tags=27%, lis
GOBP_REGULATION_OF_INFLAMMATORY_RESPONSE	64	0.238	1.401	0.08806262	0.3414902	1	267	tags=25%, lis
HALLMARK_ADIPOGENESIS	49	0.258	1.401	0.08134921	0.34148428	1	352	tags=33%, lis
HALLMARK_XENOBIOTIC_METABOLISM	27	0.316	1.401	0.11632653	0.3406312	1	391	tags=41%, lis
GOBP_POSITIVE_REGULATION_OF_TRANSLATION	21	0.338	1.400	0.11673152	0.34162685	1	559	tags=52%, lis
GOMF_GUANYL_NUCLEOTIDE_BINDING	49	0.263	1.400	0.078947365	0.34073114	1	632	tags=43%, lis
HP_ABNORMALITY_OF_SKIN_PHYSIOLOGY	95	0.216	1.399	0.070500925	0.33993283	1	397	tags=29%, lis
GOBP_CYTOPLASMIC_TRANSLATION	16	0.378	1.399	0.12577319	0.3399432	1	562	tags=50%, lis
GOMF_ATPASE_ACTIVITY	69	0.236	1.396	0.1021611	0.3426584	1	571	tags=43%, lis
HP_MENINGITIS	19	0.356	1.396	0.13569938	0.34208348	1	155	tags=26%, lis
GOBP_ENERGY_DERIVATION_BY_OXIDATION_OF_ORGANIC_COMPOUNDS	59	0.251	1.395	0.084684685	0.34354088	1	647	tags=51%, lis
REACTOME_HEME_SIGNALING	15	0.380	1.394	0.1242485	0.34446368	1	2	tags=7%, list-
GOBP_CELL_RECOGNITION	22	0.332	1.394	0.119284295	0.34362274	1	323	tags=36%, lis
HP_ARTHRALGIA	37	0.281	1.389	0.11089109	0.34919816	1	271	tags=30%, lis
HP_ACUTE_PHASE_RESPONSE	21	0.334	1.389	0.12403101	0.34855986	1	253	tags=33%, lis
GOBP_REGULATION_OF_CELL_CYCLE_PHASE_TRANSITION	82	0.226	1.388	0.08171206	0.34891054	1	410	tags=32%, lis
GOBP_NEGATIVE_REGULATION_OF_IMMUNE_SYSTEM_PROCESS	60	0.244	1.387	0.09486166	0.3496282	1	412	tags=32%, lis
HP_ABNORMAL_ABDOMEN_MORPHOLOGY	121	0.205	1.386	0.0754717	0.35102117	1	397	tags=26%, lis
GOBP_REGULATION_OF_IMMUNE_EFFECTOR_PROCESS	76	0.228	1.386	0.07874016	0.3502681	1	318	tags=22%, lis
HP_PECTUS_CARINATUM	15	0.379	1.386	0.13402061	0.34950903	1	79	tags=13%, lis
HP_FAILURE_TO_THRIVE_IN_INFANCY	21	0.341	1.385	0.09623431	0.34964743	1	235	tags=29%, lis
HP_ANEMIA_OF_INADEQUATE_PRODUCTION	29	0.289	1.384	0.09504951	0.34997723	1	107	tags=14%, lis
HP_LEUKODYSTROPHY	17	0.355	1.384	0.13598326	0.34912607	1	466	tags=47%, lis
GOBP_POSITIVE_REGULATION_OF_INFLAMMATORY_RESPONSE	28	0.304	1.383	0.13535354	0.35003456	1	161	tags=25%, lis
GOMF_ENDOPEPTIDASE_REGULATOR_ACTIVITY	21	0.321	1.382	0.11623246	0.35050985	1	549	tags=43%, lis
GOBP_NEGATIVE_REGULATION_OF_MITOTIC_CELL_CYCLE	59	0.243	1.382	0.09475806	0.35039362	1	363	tags=32%, lis
GOBP_ADENYLATE_CYCLASE_MODULATING_G_PROTEIN_COUPLED_RECEPTOR_SIGNALING_PATHW	22	0.335	1.382	0.15866388	0.3497586	1	83	tags=14%, lis
HP_ABNORMALITY_OF_THE_AMNIOTIC_FLUID	29	0.305	1.380	0.12524462	0.3518951	1	317	tags=28%, lis

GOCC_ACTIN_CYTOSKELETON	75	0.227	1.377	0.09580839	0.35573193	1	420	tags=32%, lis
GOBP_PURINE_CONTAINING_COMPOUND_BIOSYNTHETIC_PROCESS	40	0.271	1.376	0.11704312	0.35660923	1	624	tags=52%, lis
HP_ABNORMAL_CONJUNCTIVA_MORPHOLOGY	29	0.299	1.374	0.14313726	0.3581269	1	155	tags=24%, lis
HP_ABNORMALITY_OF_THE_MITOCHONDRION	23	0.324	1.373	0.1509804	0.35972568	1	841	tags=78%, lis
GOBP_CELLULAR_ION_HOMEOSTASIS	92	0.218	1.372	0.08935361	0.36014235	1	173	tags=16%, lis
GOBP_ORGANOPHOSPHATE_BIOSYNTHETIC_PROCESS	94	0.209	1.371	0.07129095	0.35975835	1	624	tags=45%, lis
GOBP_STEM_CELL_DIFFERENTIATION	51	0.249	1.371	0.08686869	0.3597537	1	476	tags=37%, lis
HP_WEAKNESS_DUE_TO_UPPER_MOTOR_NEURON_DYSFUNCTION	57	0.245	1.369	0.09719626	0.36234075	1	393	tags=30%, lis
GOCC_VACUOLAR_MEMBRANE	103	0.208	1.367	0.069767445	0.36464494	1	616	tags=41%, lis
GOBP_MIDBRAIN_DEVELOPMENT	15	0.371	1.361	0.13241106	0.37236387	1	503	tags=47%, lis
HP_RECURRENT_BACTERIAL_INFECTIONS	43	0.264	1.361	0.11967546	0.37245643	1	155	tags=19%, lis
GOBP_RESPONSE_TO_ETHANOL	17	0.360	1.360	0.1262525	0.37209028	1	225	tags=35%, lis
REACTOME_INTERLEUKIN_3_INTERLEUKIN_5_AND_GM_CSF_SIGNALING	15	0.359	1.356	0.14705883	0.37842515	1	593	tags=60%, lis
HP_ABNORMAL_ERYTHROCYTE_SEDIMENTATION_RATE	18	0.339	1.356	0.12548262	0.37762773	1	253	tags=33%, lis
HP_AUTOIMMUNITY	50	0.253	1.354	0.123203285	0.3805249	1	397	tags=32%, lis
HP_FIBROUS_TISSUE_NEOPLASM	16	0.365	1.354	0.14375	0.37965336	1	107	tags=25%, lis
HP_ABNORMALITY_OF_THE_ABDOMINAL_ORGANS	160	0.189	1.354	0.05982906	0.37893698	1	316	tags=21%, lis
GOBP_REGULATION_OF_CYTOSOLIC_CALCIUM_ION_CONCENTRATION	36	0.268	1.351	0.10982659	0.38263696	1	173	tags=19%, lis
HP_ABNORMALITY_OF_THE_LIVER	139	0.192	1.350	0.07857143	0.38247564	1	316	tags=22%, lis
GOBP_CELLULAR_RESPONSE_TO_REACTIVE_OXYGEN_SPECIES	34	0.275	1.350	0.1300813	0.38251033	1	280	tags=26%, lis
GOBP_POST_TRANSLATIONAL_PROTEIN_MODIFICATION	64	0.230	1.349	0.102661595	0.38331088	1	283	tags=25%, lis
REACTOME_INTERFERON_ALPHA_BETA_SIGNALING	19	0.341	1.349	0.13953489	0.38292745	1	149	tags=21%, lis
GOBP_POSITIVE_REGULATION_OF_TYPE_I_INTERFERON_PRODUCTION	19	0.333	1.348	0.1376673	0.3821614	1	476	tags=42%, lis
GOCC_CELL_SURFACE	97	0.208	1.345	0.071428575	0.3864687	1	262	tags=22%, lis
HP_IRREGULAR_HYPERPIGMENTATION	26	0.307	1.344	0.12547529	0.38767013	1	128	tags=19%, lis
GOBP_CELLULAR_HOMEOSTASIS	134	0.196	1.344	0.077651516	0.38717133	1	445	tags=29%, lis
GOBP_FIBROBLAST_PROLIFERATION	16	0.356	1.343	0.15789473	0.3876636	1	81	tags=25%, lis
GOBP_TYPE_I_INTERFERON_PRODUCTION	28	0.298	1.341	0.13957936	0.3904491	1	476	tags=39%, lis
HP_ABNORMALITY_OF_THE_BASAL_GANGLIA	29	0.299	1.335	0.13721414	0.39964476	1	691	tags=55%, lis
GOCC_LYSOSOMAL_LUMEN	15	0.358	1.331	0.1493776	0.40637937	1	89	tags=20%, lis
GOBP_CELLULAR_PROTEIN_COMPLEX_DISASSEMBLY	32	0.283	1.329	0.14059407	0.4083349	1	674	tags=53%, lis
HP_ABNORMALITY_OF_THE_CERVICAL_SPINE	43	0.258	1.327	0.1290944	0.41197953	1	284	tags=21%, lis
GOBP_NEGATIVE_REGULATION_OF_BINDING	26	0.301	1.325	0.14117648	0.4129135	1	255	tags=31%, lis
GOBP_REGULATION_OF_PATTERN_RECOGNITION_RECEPTOR_SIGNALING_PATHWAY	21	0.314	1.322	0.15221988	0.41767874	1	363	tags=38%, lis
HP_ABNORMALITY_OF_THE_URETHRA	41	0.255	1.322	0.12598425	0.4176281	1	364	tags=27%, lis
GOMF_PEPTIDASE_ACTIVITY	75	0.217	1.314	0.12655602	0.43121547	1	253	tags=23%, lis
GOBP_POSITIVE_REGULATION_OF_LEUKOCYTE_MIGRATION	24	0.301	1.313	0.15120968	0.43148914	1	123	tags=21%, lis
GOCC_MEMBRANE_COAT	21	0.321	1.313	0.17460318	0.43069348	1	931	tags=76%, lis
REACTOME_FCGAMMA_RECEPTOR_FCGR_DEPENDENT_PHAGOCYTOSIS	29	0.291	1.311	0.17764471	0.432634	1	500	tags=48%, lis
GOBP_PEPTIDE_BIOSYNTHETIC_PROCESS	105	0.200	1.311	0.11299435	0.43207988	1	685	tags=46%, lis
GOMF_CATION_CHANNEL_ACTIVITY	22	0.314	1.311	0.16833667	0.43115363	1	603	tags=55%, lis
GOBP_PALLIUM_DEVELOPMENT	17	0.339	1.310	0.188	0.4329858	1	412	tags=35%, lis
GOCC_TERTIARY_GRANULE	43	0.254	1.308	0.1670061	0.43476734	1	416	tags=33%, lis
HALLMARK_KRAS_SIGNALING_UP	35	0.264	1.306	0.1670061	0.43722376	1	306	tags=29%, lis
GOMF_G_PROTEIN_COUPLED_RECEPTOR_BINDING	25	0.297	1.306	0.15127702	0.43688658	1	351	tags=28%, lis
GOBP_METAL_ION_HOMEOSTASIS	86	0.212	1.305	0.11222445	0.43690807	1	173	tags=16%, lis
GOBP_RESPONSE_TO_MOLECULE_OF_BACTERIAL_ORIGIN	62	0.227	1.304	0.14931238	0.4375103	1	137	tags=15%, lis
HP_ABNORMAL_NEURAL_TUBE_MORPHOLOGY	21	0.319	1.303	0.16411379	0.43870693	1	358	tags=43%, lis
GOBP_POSITIVE_REGULATION_OF_CYTOKINE_PRODUCTION	85	0.210	1.303	0.1359404	0.4391111	1	374	tags=27%, lis
GOBP_ION_HOMEOSTASIS	104	0.201	1.302	0.12525667	0.43880224	1	399	tags=27%, lis
GOBP_LEUKOCYTE_MIGRATION	82	0.212	1.302	0.13773584	0.43859375	1	203	tags=17%, lis
GOBP_PROTEIN_POLYUBIQUITINATION	78	0.216	1.298	0.11111111	0.44495738	1	283	tags=23%, lis
HP_SENSORY_NEUROPATHY	24	0.286	1.295	0.16839917	0.4484426	1	229	tags=25%, lis
REACTOME_FATTY_ACID_METABOLISM	20	0.321	1.295	0.16179337	0.44769236	1	936	tags=80%, lis
GOBP_AUTOPHAGOSOME_ORGANIZATION	19	0.323	1.295	0.16071428	0.44771123	1	420	tags=42%, lis
PID_P53_DOWNSTREAM_PATHWAY	25	0.289	1.292	0.18181819	0.45177418	1	163	tags=16%, lis
GOBP_INORGANIC_ION_TRANSMEMBRANE_TRANSPORT	89	0.206	1.292	0.14466546	0.45150745	1	653	tags=45%, lis
HP_RED_EYE	24	0.297	1.290	0.17850287	0.4530678	1	155	tags=25%, lis
GOBP_MAINTENANCE_OF_LOCATION	56	0.230	1.289	0.14876033	0.45486823	1	187	tags=18%, lis
GOBP_ADAPTIVE_IMMUNE_RESPONSE_BASED_ON_SOMATIC_RECOMBINATION_OF_IMMUNE_RE(	60	0.222	1.288	0.1574642	0.45544475	1	198	tags=20%, lis
HP_ABNORMALITY_OF_THE_CEREBROSPINAL_FLUID	66	0.218	1.286	0.14035088	0.45759705	1	458	tags=30%, lis
HP_APLASIA_HYPOPLASIA_OF_THE_EYEBROW	23	0.302	1.285	0.16221374	0.45970234	1	86	tags=9%, list-
HP_LOWER_LIMB_SPASTICITY	33	0.268	1.282	0.16565657	0.46315068	1	442	tags=36%, lis
HP_TUBE_FEEDING	38	0.250	1.282	0.16571428	0.4623462	1	358	tags=34%, lis
HP_ABNORMAL_CIRCULATING_METABOLITE_CONCENTRATION	155	0.182	1.279	0.115311906	0.46710017	1	290	tags=23%, lis
REACTOME_PARASITE_INFECTION	21	0.314	1.278	0.20816326	0.46858147	1	460	tags=48%, lis
GOCC_VESICLE_COAT	18	0.321	1.278	0.18421052	0.46803573	1	931	tags=78%, lis
HP_POOR_SPEECH	44	0.246	1.277	0.16833667	0.46911433	1	580	tags=41%, lis
GOBP_TAXIS	77	0.211	1.277	0.14365672	0.4685079	1	142	tags=14%, lis
HP_NEUROLOGICAL_SPEECH_IMPAIRMENT	159	0.177	1.273	0.110091746	0.47482666	1	374	tags=23%, lis
GOBP_POSITIVE_REGULATION_OF_CYSTEINE_TYPE_ENDOPEPTIDASE_ACTIVITY	25	0.284	1.273	0.186	0.47388262	1	485	tags=48%, lis
GOBP_DIVALENT_INORGANIC_CATION_HOMEOSTASIS	58	0.222	1.269	0.16988418	0.47956356	1	173	tags=17%, lis
GOMF_IDENTICAL_PROTEIN_BINDING	273	0.159	1.268	0.09057301	0.48145387	1	312	tags=19%, lis
GOBP_REGULATION_OF_TOLL_LIKE_RECEPTOR_SIGNALING_PATHWAY	16	0.348	1.268	0.2034632	0.48104343	1	315	tags=38%, lis
REACTOME_INFECTIOUS_DISEASE	165	0.177	1.266	0.119373776	0.4826346	1	460	tags=30%, lis
HP_ABNORMAL_BLOOD_GLUCOSE_CONCENTRATION	25	0.287	1.266	0.19038077	0.48255318	1	458	tags=40%, lis
GOBP_CELLULAR_RESPONSE_TO_MOLECULE_OF_BACTERIAL_ORIGIN	39	0.256	1.265	0.18736842	0.48263475	1	137	tags=15%, lis
HP_RECURRENT_UPPER_RESPIRATORY_TRACT_INFECTIONS	33	0.266	1.265	0.20634921	0.48174968	1	163	tags=24%, lis
GOBP_EXOCYTOSIS	180	0.175	1.263	0.11254612	0.48497817	1	416	tags=26%, lis

GOBP_RESPONSE_TO_INTERLEUKIN_12	15	0.343	1.259	0.20450282	0.49179044	1	869	tags=73%, lis
HP_ABNORMAL_LYMPHOCYTE_MORPHOLOGY	44	0.243	1.258	0.17165668	0.4929118	1	405	tags=34%, lis
HP_ABNORMAL_CSF_METABOLITE_LEVEL	22	0.301	1.258	0.21255061	0.4927547	1	543	tags=45%, lis
GOCC_AZUROPHIL_GRANULE	47	0.236	1.257	0.172	0.49204594	1	297	tags=21%, lis
GOBP_NEGATIVE_REGULATION_OF_PROTEIN_BINDING	16	0.332	1.256	0.21443298	0.49395475	1	255	tags=31%, lis
GOBP_NEGATIVE_REGULATION_OF_CELL_CYCLE_PROCESS	66	0.217	1.255	0.17213115	0.49452943	1	363	tags=30%, lis
GOBP_PHAGOCYTOSIS	71	0.208	1.255	0.17087379	0.49375325	1	593	tags=41%, lis
GOCC_CATALYTIC_STEP_2_SPLICEOSOME	16	0.333	1.253	0.20550847	0.49702945	1	618	tags=56%, lis
HP_ABNORMAL_PATTERN_OF_RESPIRATION	37	0.251	1.253	0.18518518	0.49654397	1	809	tags=59%, lis
GOBP_CYTOPLASMIC_PATTERN_RECOGNITION_RECEPTOR_SIGNALING_PATHWAY	20	0.314	1.251	0.21235521	0.49931595	1	374	tags=35%, lis
REACTOME_PROTEIN_FOLDING	20	0.304	1.250	0.21912351	0.49946958	1	258	tags=30%, lis
GOBP_REGULATION_OF_ACTIN_FILAMENT_LENGTH	39	0.249	1.250	0.20281124	0.49892256	1	640	tags=51%, lis
HALLMARK_IL6_JAK_STAT3_SIGNALING	33	0.260	1.249	0.20194174	0.49906623	1	273	tags=27%, lis
GOBP_PROTEIN_COMPLEX_OLIGOMERIZATION	30	0.274	1.249	0.19488189	0.49874184	1	172	tags=20%, lis
HP_EMOTIONAL_LABILITY	19	0.308	1.249	0.21384929	0.49785352	1	247	tags=26%, lis
REACTOME_DISEASES_OF_SIGNAL_TRANSDUCTION_BY_GROWTH_FACTOR_RECEPTORS_AND_SEC	81	0.202	1.248	0.15257353	0.4987636	1	253	tags=22%, lis
HP_DEPRESSIVITY	56	0.225	1.246	0.18897638	0.50131786	1	585	tags=45%, lis
REACTOME_INTERLEUKIN_12_SIGNALING	15	0.343	1.245	0.20961538	0.5026251	1	869	tags=73%, lis
HP_HYPOPIGMENTED_SKIN_PATCHES	20	0.312	1.245	0.21443298	0.5022877	1	855	tags=65%, lis
HP_HEPATOMEGALY	79	0.206	1.243	0.18304431	0.5044966	1	414	tags=25%, lis
GOBP_PEPTIDE_METABOLIC_PROCESS	127	0.183	1.240	0.15162455	0.5090525	1	685	tags=45%, lis
HP_LYMPHOPENIA	36	0.260	1.239	0.21294363	0.5107746	1	279	tags=28%, lis
KEGG_TOLL_LIKE_RECEPTOR_SIGNALING_PATHWAY	24	0.295	1.239	0.204	0.5104949	1	202	tags=21%, lis
GOBP_TISSUE_HOMEOSTASIS	24	0.286	1.234	0.2310757	0.5183554	1	592	tags=46%, lis
REACTOME_TP53_REGULATES_METABOLIC_GENES	33	0.254	1.232	0.2102161	0.5232867	1	854	tags=64%, lis
GOBP_REGULATION_OF_LEUKOCYTE_MIGRATION	36	0.248	1.228	0.19642857	0.52908486	1	123	tags=17%, lis
GOBP_REGULATION_OF_BIOLOGICAL_PROCESS_INVOLVED_IN_SYMBIOTIC_INTERACTION	34	0.250	1.227	0.20231214	0.5308414	1	274	tags=24%, lis
REACTOME_DEATH_RECEPTOR_SIGNALLING	26	0.277	1.227	0.23238096	0.53031486	1	628	tags=50%, lis
REACTOME_INTERFERON_SIGNALING	46	0.228	1.226	0.20233463	0.5294559	1	344	tags=24%, lis
HP_INFLAMMATION_OF_THE_LARGE_INTESTINE	21	0.294	1.225	0.2177264	0.53097045	1	124	tags=19%, lis
HP_ECTOPIC_CALCIFICATION	25	0.272	1.225	0.22857143	0.5309531	1	690	tags=52%, lis
GOBP_REGULATION_OF_PHAGOCYTOSIS	30	0.265	1.224	0.21694215	0.5313614	1	484	tags=40%, lis
GOMF_PHOSPHOPROTEIN_BINDING	16	0.323	1.221	0.23175965	0.53607345	1	593	tags=56%, lis
GOMF_PEPTIDASE_REGULATOR_ACTIVITY	29	0.268	1.221	0.22072937	0.53521097	1	413	tags=34%, lis
GOBP_POSITIVE_REGULATION_OF_ESTABLISHMENT_OF_PROTEIN_LOCALIZATION_TO_MITOCHONI	17	0.310	1.221	0.22594142	0.534859	1	548	tags=53%, lis
PID_BCR_5PATHWAY	27	0.274	1.221	0.24593496	0.5339594	1	500	tags=37%, lis
HP_SPINAL_DYSRAPHISM	19	0.307	1.220	0.2193676	0.53530335	1	358	tags=42%, lis
KEGG_CELL_CYCLE	22	0.292	1.219	0.21714285	0.5351908	1	503	tags=41%, lis
GOBP_REGULATION_OF_RESPONSE_TO_OXIDATIVE_STRESS	16	0.325	1.219	0.25203252	0.5355678	1	255	tags=25%, lis
GOBP_CELLULAR_RESPONSE_TO_CHEMICAL_STRESS	68	0.206	1.217	0.19778189	0.53796965	1	525	tags=35%, lis
GOBP_POSITIVE_REGULATION_OF_LEUKOCYTE_PROLIFERATION	22	0.290	1.217	0.24285714	0.5371087	1	188	tags=23%, lis
REACTOME_EPH_EPHRIN_SIGNALING	18	0.314	1.216	0.24248497	0.53761667	1	745	tags=67%, lis
GOMF_PROTEIN_DIMERIZATION_ACTIVITY	129	0.180	1.216	0.16962525	0.5367438	1	315	tags=19%, lis
GOBP_POSITIVE_REGULATION_OF_PEPTIDYL_SERINE_PHOSPHORYLATION	17	0.312	1.216	0.23352166	0.5357365	1	15	tags=12%, lis
HP_APLASIA_HYPOPLASIA_OF_TOE	20	0.302	1.216	0.22178988	0.534763	1	2	tags=5%, list-
GOBP_CELLULAR_RESPONSE_TO_BIOTIC_STIMULUS	44	0.227	1.215	0.20502092	0.53504837	1	137	tags=14%, lis
GOBP_POSITIVE_REGULATION_OF_TRANSPORT	137	0.172	1.215	0.15934066	0.5345666	1	520	tags=33%, lis
GOBP_PROTEIN_FOLDING	46	0.227	1.212	0.20934579	0.54014254	1	586	tags=41%, lis
HP_IMMUNODEFICIENCY	61	0.209	1.211	0.20643939	0.5400177	1	315	tags=25%, lis
GOBP_AEROBIC_RESPIRATION	24	0.282	1.211	0.23046093	0.5392142	1	641	tags=58%, lis
GOMF_TRANSLATION_REGULATOR_ACTIVITY_NUCLEIC_ACID_BINDING	25	0.274	1.211	0.22515213	0.5381829	1	753	tags=56%, lis
GOBP_REGULATION_OF_ESTABLISHMENT_OF_PROTEIN_LOCALIZATION_TO_MITOCHONDRION	20	0.297	1.211	0.22310758	0.5380086	1	548	tags=50%, lis
HP_ABNORMAL_PHARYNX_MORPHOLOGY	36	0.241	1.209	0.2264529	0.5405885	1	163	tags=22%, lis
GOBP_MAINTENANCE_OF_PROTEIN_LOCATION	20	0.303	1.209	0.22648752	0.54101735	1	388	tags=35%, lis
GOMF_CATALYTIC_ACTIVITY_ACTING_ON_DNA	23	0.279	1.205	0.22950819	0.5484618	1	476	tags=43%, lis
HP_MACULE	25	0.276	1.204	0.27383366	0.547764	1	79	tags=16%, lis
GOBP_ADAPTIVE_IMMUNE_RESPONSE	85	0.194	1.204	0.20342205	0.5484876	1	198	tags=19%, lis
HP_RECURRENT_OTITIS_MEDIA	29	0.259	1.203	0.21899225	0.5477945	1	358	tags=34%, lis
HP_APNEA	31	0.249	1.203	0.24760076	0.54771715	1	809	tags=58%, lis
GOBP_RESPONSE_TO_INORGANIC_SUBSTANCE	88	0.191	1.203	0.20454545	0.5469654	1	136	tags=11%, lis
KEGG_LEISHMANIA_INFECTION	24	0.280	1.203	0.22153209	0.5465257	1	361	tags=33%, lis
PID_P75_NTR_PATHWAY	15	0.325	1.201	0.24894515	0.5477015	1	544	tags=53%, lis
GOBP_CELL_CELL_SIGNALING_BY_WNT	97	0.193	1.201	0.18574108	0.54674804	1	364	tags=28%, lis
GOCC_SECRETORY_GRANULE	166	0.165	1.201	0.18378378	0.54745895	1	416	tags=25%, lis
HP_ABNORMAL_ADIPOSE_TISSUE_MORPHOLOGY	40	0.235	1.200	0.21756487	0.546918	1	253	tags=23%, lis
GOCC_ENDOCYTIC_VESICLE	69	0.201	1.198	0.19881889	0.55122346	1	19	tags=6%, list-
GOBP_REGULATION_OF_CELLULAR_PROTEIN_CATABOLIC_PROCESS	59	0.212	1.197	0.22700587	0.5522697	1	363	tags=29%, lis
GOBP_ESTABLISHMENT_OR_MAINTENANCE_OF_CELL_POLARITY	34	0.243	1.197	0.25	0.5517532	1	632	tags=47%, lis
HP_CONSTITUTIONAL_SYMPTOM	148	0.172	1.196	0.17829457	0.55273765	1	284	tags=19%, lis
GOCC_COPII_COATED_ER_TO_GOLGI_TRANSPORT_VESICLE	18	0.303	1.195	0.25308642	0.55324614	1	19	tags=11%, lis
GOCC_TERTIARY_GRANULE_MEMBRANE	19	0.300	1.195	0.24524716	0.5526821	1	416	tags=37%, lis
GOBP_NEGATIVE_REGULATION_OF_INTRINSIC_APOPTOTIC_SIGNALING_PATHWAY	20	0.294	1.189	0.253493	0.56415904	1	440	tags=35%, lis
HP_REDUCED_CONSCIOUSNESS_CONFUSION	38	0.236	1.188	0.23838384	0.5655589	1	500	tags=37%, lis
HP_ABNORMAL_VASCULAR_PHYSIOLOGY	39	0.233	1.187	0.26043737	0.5656469	1	448	tags=36%, lis
HP_ABNORMALITY_OF_THE_HELIX	17	0.307	1.186	0.2576336	0.5671477	1	358	tags=35%, lis
GOBP_REGULATION_OF_CELLULAR_CATABOLIC_PROCESS	187	0.161	1.186	0.16635859	0.5665293	1	363	tags=23%, lis
REACTOME_ORGANELLE_BIOGENESIS_AND_MAINTENANCE	49	0.221	1.185	0.23320158	0.56700087	1	503	tags=37%, lis
GOBP_REGULATION_OF_BLOOD_PRESSURE	18	0.302	1.185	0.2751004	0.5663084	1	364	tags=33%, lis
HP_ABNORMAL_CNS_MYELINATION	45	0.227	1.183	0.23846154	0.56974113	1	550	tags=38%, lis

HP_VISUAL_IMPAIRMENT	121	0.175	1.181	0.20715632	0.57225806	1	535	tags=35%, lis
HP_BROAD_FOREHEAD	25	0.267	1.181	0.25646123	0.5714874	1	500	tags=32%, lis
GOBP_NEGATIVE_REGULATION_OF_CELL_CELL_ADHESION	27	0.261	1.180	0.26760563	0.5728965	1	123	tags=19%, lis
GOBP_REGULATION_OF_B_CELL_ACTIVATION	32	0.251	1.180	0.24744377	0.5721133	1	198	tags=22%, lis
GOBP_ADENYLATE_CYCLASE_ACTIVATING_G_PROTEIN_COUPLED_RECEPTOR_SIGNALING_PATHWA	15	0.324	1.179	0.25301206	0.5733827	1	83	tags=13%, lis
GOBP_REGULATION_OF_PROTEIN_TARGETING	16	0.311	1.178	0.27875245	0.5750255	1	548	tags=50%, lis
GOMF_TRANSLATION_INITIATION_FACTOR_ACTIVITY	17	0.305	1.177	0.24259259	0.5755261	1	562	tags=47%, lis
GOBP_BIOLOGICAL_PROCESS_INVOLVED_IN_INTERACTION_WITH_HOST	43	0.225	1.176	0.23904383	0.5759756	1	203	tags=19%, lis
HP_UNUSUAL_INFECTION	156	0.166	1.176	0.21268657	0.57544714	1	405	tags=26%, lis
HP_REDUCED_VISUAL_ACUITY	58	0.208	1.176	0.22988506	0.5746107	1	535	tags=38%, lis
GOBP_CELL_SURFACE_RECEPTOR_SIGNALING_PATHWAY_INVOLVED_IN_CELL_CELL_SIGNALING	103	0.178	1.174	0.21298175	0.57762134	1	255	tags=21%, lis
HP_INCREASED_CIRCULATING_ANTIBODY_LEVEL	27	0.259	1.174	0.26254827	0.57771856	1	253	tags=30%, lis
GOBP_HOMEOSTATIC_PROCESS	255	0.151	1.173	0.17247707	0.5779733	1	399	tags=23%, lis
GOMF_TRANSLATION_FACTOR_ACTIVITY_RNA_BINDING	21	0.282	1.173	0.28781512	0.5780657	1	753	tags=57%, lis
HP_SOFT_TISSUE_SARCOMA	21	0.284	1.172	0.2784314	0.5770471	1	107	tags=19%, lis
HP_LIPODYSTROPHY	21	0.277	1.172	0.276	0.57750344	1	253	tags=29%, lis
HP_IMMUNOLOGIC_HYPERSENSITIVITY	31	0.248	1.172	0.25048923	0.5767312	1	353	tags=32%, lis
GOMF_TRANSLATION_REGULATOR_ACTIVITY	31	0.244	1.171	0.2936508	0.5774215	1	753	tags=55%, lis
GOBP_NEGATIVE_REGULATION_OF_DNA_BINDING_TRANSCRIPTION_FACTOR_ACTIVITY	27	0.259	1.170	0.25690022	0.57883996	1	295	tags=30%, lis
GOBP_INTERLEUKIN_1_PRODUCTION	17	0.299	1.169	0.28657314	0.5788242	1	91	tags=18%, lis
GOMF_PEPTIDASE_ACTIVATOR_ACTIVITY	15	0.325	1.168	0.28884462	0.5812867	1	413	tags=47%, lis
HP_ARTHRITIS	34	0.240	1.167	0.26953125	0.58235747	1	284	tags=32%, lis
GOBP_AGING	49	0.213	1.166	0.25	0.5835508	1	127	tags=10%, lis
HP_ABNORMALITY_OF_THE_LOWER_URINARY_TRACT	83	0.187	1.166	0.2164751	0.58271253	1	385	tags=24%, lis
GOBP_I_KAPPAB_KINASE_NF_KAPPAB_SIGNALING	53	0.213	1.166	0.27238095	0.5820583	1	476	tags=34%, lis
HP_FULL_CHEEKS	19	0.298	1.165	0.27474746	0.5819008	1	688	tags=58%, lis
HP_AIRWAY_OBSTRUCTION	16	0.305	1.165	0.2854251	0.5813425	1	315	tags=38%, lis
REACTOME_AUTOPHAGY	29	0.253	1.164	0.26692456	0.5822034	1	498	tags=38%, lis
GOBP_NEGATIVE_REGULATION_OF_GROWTH	37	0.235	1.161	0.2825279	0.58752686	1	436	tags=30%, lis
GOBP_REGULATION_OF_DNA_BINDING	27	0.260	1.160	0.28290766	0.58836347	1	254	tags=26%, lis
GOBP_CYTOSOLIC_CALCIUM_ION_TRANSPORT	20	0.284	1.160	0.26638478	0.5883331	1	173	tags=20%, lis
GOBP_STRIATED_MUSCLE_CELL_DIFFERENTIATION	28	0.257	1.159	0.28	0.58878005	1	225	tags=18%, lis
GOBP_POSITIVE_REGULATION_OF_ION_TRANSPORT	93	0.181	1.159	0.2472119	0.58824	1	689	tags=45%, lis
GOBP_PROTEASOMAL_PROTEIN_CATABOLIC_PROCESS	112	0.177	1.159	0.22718808	0.5882044	1	379	tags=26%, lis
KEGG_FC_GAMMA_R_MEDIATED_PHAGOCYTOSIS	28	0.254	1.158	0.268714	0.58901757	1	689	tags=54%, lis
HP_ABNORMAL_LYMPHOCYTE_PHYSIOLOGY	71	0.192	1.158	0.27238095	0.58809257	1	374	tags=28%, lis
HP_ABNORMAL_CARDIAC_VENTRICLE_MORPHOLOGY	71	0.194	1.157	0.25409836	0.5876336	1	862	tags=61%, lis
HP_HEMIPLEGIA_HEMIPARESIS	20	0.283	1.156	0.27852997	0.5887312	1	374	tags=30%, lis
HP_GASTROINTESTINAL_INFLAMMATION	26	0.258	1.155	0.275	0.59005326	1	155	tags=19%, lis
HP_IRRITABILITY	25	0.264	1.152	0.2843327	0.5963593	1	107	tags=20%, lis
GOBP_RECEPTOR_MEDIATED_ENDOCYTOSIS	50	0.212	1.151	0.27952754	0.59828466	1	55	tags=8%, list
HP_HYDROCEPHALUS	39	0.225	1.150	0.26654065	0.599047	1	290	tags=21%, lis
HP_HYPERPIGMENTATION_OF_THE_SKIN	46	0.217	1.150	0.28514057	0.5980529	1	315	tags=24%, lis
GOBP_PROTEIN_CONTAINING_COMPLEX_DISASSEMBLY	57	0.206	1.149	0.2611336	0.598094	1	683	tags=46%, lis
REACTOME_CELL_CYCLE	108	0.176	1.148	0.25714287	0.59935486	1	344	tags=24%, lis
HP_MACROGLOSSIA	17	0.297	1.148	0.31120333	0.59908396	1	98	tags=24%, lis
HP_BLINDNESS	32	0.240	1.148	0.27592954	0.59865075	1	500	tags=41%, lis
HP_DECREASED_BODY_WEIGHT	193	0.156	1.146	0.22222222	0.6007016	1	324	tags=20%, lis
GOBP_CELLULAR_MODIFIED_AMINO_ACID_METABOLIC_PROCESS	16	0.305	1.145	0.29352227	0.6033528	1	225	tags=31%, lis
HP_ABNORMAL_LIVER_MORPHOLOGY	110	0.173	1.144	0.2651072	0.6046473	1	418	tags=25%, lis
REACTOME_MITOCHONDRIAL_BIOGENESIS	27	0.252	1.143	0.28294572	0.6039624	1	621	tags=48%, lis
GOBP_MACROPHAGE_ACTIVATION	17	0.300	1.142	0.3015873	0.60533184	1	22	tags=12%, lis
GOMF_SIGNALING_RECEPTOR_BINDING	185	0.156	1.142	0.23963964	0.6050116	1	422	tags=26%, lis
GOBP_SECRETION	229	0.150	1.141	0.2192029	0.60570824	1	416	tags=24%, lis
GOBP_REGULATION_OF_MRNA_PROCESSING	36	0.232	1.141	0.28735632	0.60591924	1	348	tags=31%, lis
HP_HALLUCINATIONS	20	0.277	1.138	0.3190184	0.60985035	1	664	tags=50%, lis
HP_CEREBRAL_VISUAL_IMPAIRMENT	35	0.238	1.138	0.28486055	0.60960084	1	698	tags=51%, lis
GOBP_NEGATIVE_REGULATION_OF_RESPONSE_TO_ENDOPLASMIC_RETICULUM_STRESS	15	0.307	1.137	0.29218107	0.61106706	1	1468	tags=100%, l
GOBP_REGULATION_OF_CELL_DEATH	253	0.148	1.133	0.2173913	0.61759895	1	440	tags=24%, lis
GOBP_CELL_DEATH_IN_RESPONSE_TO_OXIDATIVE_STRESS	16	0.295	1.133	0.29761904	0.6169208	1	255	tags=25%, lis
PID_MYC_ACTIV_PATHWAY	19	0.279	1.132	0.30538923	0.61839485	1	449	tags=37%, lis
GOBP_CELL_POPULATION_PROLIFERATION	238	0.146	1.132	0.2400722	0.61812997	1	118	tags=10%, lis
HP_RESPIRATORY_TRACT_INFECTION	108	0.170	1.131	0.2825279	0.6181211	1	397	tags=27%, lis
GOCC_VACUOLE	166	0.158	1.127	0.2659176	0.6256518	1	692	tags=40%, lis
GOBP_B_CELL_ACTIVATION	58	0.201	1.126	0.28	0.62758553	1	198	tags=19%, lis
GOBP_REGULATION_OF_MUSCLE_CELL_DIFFERENTIATION	21	0.277	1.125	0.31536925	0.63009024	1	42	tags=14%, lis
GOBP_SMOOTH_MUSCLE_CELL_PROLIFERATION	22	0.272	1.124	0.32115385	0.62955374	1	70	tags=18%, lis
GOBP_MAPK_CASCADE	160	0.157	1.123	0.26542056	0.63242763	1	220	tags=16%, lis
HP_CEREBRAL_CALCIFICATION	20	0.279	1.123	0.3079848	0.6314367	1	690	tags=55%, lis
HP_ABNORMALITY_OF_BLOOD_AND_BLOOD_FORMING_TISSUES	218	0.147	1.121	0.24953096	0.63401055	1	405	tags=23%, lis
GOBP_REGULATION_OF_BINDING	74	0.187	1.120	0.2755102	0.634065	1	255	tags=22%, lis
GOBP_PH_REDUCTION	15	0.306	1.119	0.32040817	0.6353447	1	664	tags=53%, lis
HP_ABNORMALITY_OF_SKIN_PIGMENTATION	79	0.182	1.118	0.29961088	0.636673	1	368	tags=25%, lis
HP_HYPERTROPHIC_CARDIOMYOPATHY	33	0.235	1.118	0.31779662	0.6361591	1	691	tags=48%, lis
GOMF_ANION_TRANSMEMBRANE_TRANSPORTER_ACTIVITY	33	0.235	1.118	0.31337327	0.63613176	1	611	tags=42%, lis
GOMF_SERINE_HYDROLASE_ACTIVITY	15	0.307	1.117	0.3184466	0.63616186	1	34	tags=13%, lis
GOBP_REGULATION_OF_MRNA_SPLICING_VIA_SPLICEOSOME	26	0.253	1.117	0.3257732	0.6355199	1	419	tags=35%, lis
HP_INCOORDINATION	31	0.238	1.117	0.33088234	0.6355401	1	364	tags=29%, lis
GOBP_REGULATION_OF_INTRINSIC_APOPTOTIC_SIGNALING_PATHWAY	39	0.220	1.115	0.3131313	0.6370794	1	440	tags=33%, lis

GOBP_REGULATION_OF_MITOCHONDRIAL_MEMBRANE_POTENTIAL	15	0.302	1.114	0.31673306	0.638095	1	388	tags=47%, lis
GOBP_CELL_CYCLE_PHASE_TRANSITION	106	0.170	1.114	0.2737864	0.6375376	1	337	tags=24%, lis
GOBP_POSITIVE_REGULATION_OF_LIPID_METABOLIC_PROCESS	18	0.281	1.112	0.33460075	0.6406903	1	45	tags=17%, lis
HP_ABNORMAL_HEART_VALVE_MORPHOLOGY	36	0.228	1.111	0.32553193	0.6420659	1	397	tags=33%, lis
GOBP_APOPTOTIC_SIGNALING_PATHWAY	121	0.161	1.111	0.28820115	0.6415721	1	470	tags=27%, lis
GOBP_REGULATION_OF_CHEMOTAXIS	30	0.244	1.109	0.296	0.6450784	1	123	tags=17%, lis
GOBP_NEGATIVE_REGULATION_OF_NF_KAPPAB_TRANSCRIPTION_FACTOR_ACTIVITY	17	0.283	1.109	0.30844793	0.64408034	1	295	tags=35%, lis
GOBP_CELL_CELL_ADHESION	109	0.168	1.109	0.28544775	0.6442321	1	246	tags=18%, lis
REACTOME_INTERLEUKIN_12_FAMILY_SIGNALING	20	0.277	1.108	0.3360825	0.64366436	1	127	tags=20%, lis
HP_ABSENT_SEPTUM_PELLUCIDUM	16	0.294	1.107	0.31380752	0.6452684	1	520	tags=44%, lis
GOBP_NEGATIVE_REGULATION_OF_RESPONSE_TO_STIMULUS	240	0.145	1.106	0.2587156	0.6460534	1	255	tags=17%, lis
HP_CHRONIC_OTITIS_MEDIA	27	0.245	1.104	0.32149532	0.6492553	1	284	tags=30%, lis
GOBP_REGULATION_OF_CALCIUM_ION_TRANSMEMBRANE_TRANSPORT	15	0.299	1.104	0.34475806	0.64837855	1	549	tags=47%, lis
GOMF_RIBONUCLEOPROTEIN_COMPLEX_BINDING	27	0.246	1.104	0.32985386	0.64864504	1	559	tags=41%, lis
HP_ABNORMALITY_OF_THE_ZYGOMATIC_BONE	26	0.251	1.103	0.34756097	0.64883304	1	86	tags=8%, list
GOMF_UNFOLDED_PROTEIN_BINDING	27	0.243	1.103	0.31558937	0.6481762	1	574	tags=41%, lis
GOBP_MODIFICATION_DEPENDENT_MACROMOLECULE_CATABOLIC_PROCESS	140	0.157	1.101	0.30018762	0.6516341	1	453	tags=26%, lis
KEGG_CELL_ADHESION_MOLECULES_CAMS	18	0.275	1.101	0.33996025	0.6507264	1	203	tags=22%, lis
KEGG_PATHOGENIC_ESCHERICHIA_COLI_INFECTION	15	0.297	1.101	0.35744682	0.6507507	1	417	tags=40%, lis
HP_ABNORMALITY_OF_THE_LYMPH_NODES	60	0.197	1.099	0.30722892	0.6525688	1	405	tags=30%, lis
GOCC_PLASMA_MEMBRANE_PROTEIN_COMPLEX	59	0.190	1.098	0.32282004	0.65472823	1	220	tags=20%, lis
GOBP_POSITIVE_REGULATION_OF_CELL_DEATH	111	0.167	1.097	0.30740038	0.65565616	1	413	tags=23%, lis
HP_ABNORMAL_CIRCULATING_PROTEIN_CONCENTRATION	72	0.183	1.096	0.32596686	0.65721005	1	290	tags=22%, lis
HP_POLYDACTYLY	20	0.268	1.095	0.35146442	0.65866	1	284	tags=30%, lis
HP_OSTEOMYELITIS	22	0.258	1.094	0.33742332	0.6599842	1	587	tags=45%, lis
GOBP_PROTEIN_LOCALIZATION_TO_ORGANELLE	158	0.155	1.094	0.30929792	0.6590957	1	410	tags=25%, lis
HP_ABNORMAL_THYROID_MORPHOLOGY	21	0.262	1.094	0.35443038	0.6581752	1	107	tags=19%, lis
GOCC_TRANSPORT_VESICLE_MEMBRANE	22	0.264	1.093	0.34710744	0.6577226	1	284	tags=23%, lis
GOBP_VACUOLE_ORGANIZATION	32	0.227	1.093	0.3713733	0.65867406	1	466	tags=34%, lis
GOBP_REGULATION_OF_MITOTIC_CELL_CYCLE	100	0.170	1.092	0.3181818	0.658437	1	438	tags=28%, lis
GOBP_RRNA_METABOLIC_PROCESS	23	0.259	1.092	0.36820924	0.6589832	1	656	tags=48%, lis
HP_FREQUENT_FALLS	18	0.268	1.091	0.33935744	0.6584284	1	364	tags=33%, lis
HP_HEPATITIS	22	0.258	1.091	0.346856	0.65766907	1	249	tags=27%, lis
GOBP_IMMUNE_SYSTEM_DEVELOPMENT	194	0.146	1.091	0.3057554	0.65678316	1	253	tags=18%, lis
HP_UNSTEADY_GAIT	16	0.285	1.091	0.34631148	0.6558623	1	1042	tags=88%, lis
KEGG_TIGHT_JUNCTION	16	0.292	1.091	0.34285715	0.65510815	1	227	tags=25%, lis
HP_ABNORMAL_BRONCHUS_MORPHOLOGY	29	0.236	1.090	0.34319526	0.65530866	1	163	tags=21%, lis
GOBP_REGULATION_OF_TRANSPORT	236	0.142	1.090	0.29834256	0.6550673	1	413	tags=24%, lis
GOBP_CELLULAR_PROTEIN_CATABOLIC_PROCESS	169	0.152	1.090	0.31003585	0.654713	1	379	tags=23%, lis
GOBP_MUSCLE_STRUCTURE_DEVELOPMENT	72	0.184	1.089	0.3372093	0.65607643	1	83	tags=10%, lis
HP_NEVUS	17	0.278	1.087	0.34764826	0.6600095	1	776	tags=59%, lis
HP_ABNORMAL_EYEBROW_MORPHOLOGY	71	0.180	1.086	0.3313253	0.66090703	1	350	tags=21%, lis
GOBP_POSITIVE_REGULATION_OF_CELLULAR_AMIDE_METABOLIC_PROCESS	28	0.238	1.086	0.3503055	0.6602619	1	559	tags=43%, lis
GOBP_ANIMAL_ORGAN_MORPHOGENESIS	100	0.167	1.085	0.33333334	0.6601029	1	320	tags=22%, lis
GOCC_SPLICEOSOMAL_COMPLEX	41	0.215	1.084	0.375	0.66180414	1	559	tags=39%, lis
HP_OTITIS_MEDIA	63	0.191	1.084	0.3326572	0.6608465	1	358	tags=29%, lis
HP_PAIN	113	0.163	1.084	0.32677165	0.66003114	1	284	tags=20%, lis
GOBP_REGULATION_OF_PH	18	0.275	1.083	0.35020244	0.6599617	1	664	tags=50%, lis
KEGG_FC_EPSILON_RI_SIGNALING_PATHWAY	15	0.300	1.083	0.36105478	0.65976375	1	602	tags=53%, lis
REACTOME_GOLGI_TO_ER_RETROGRADE_TRANSPORT	23	0.257	1.083	0.34455445	0.6596231	1	1244	tags=87%, lis
HP_ABNORMALITY_OF_VISION	148	0.152	1.081	0.3408663	0.662293	1	535	tags=32%, lis
GOBP_TELENCEPHALON_DEVELOPMENT	20	0.264	1.081	0.3809524	0.66175884	1	412	tags=30%, lis
PID_IL12_2PATHWAY	16	0.287	1.080	0.3442623	0.66329694	1	175	tags=25%, lis
HALLMARK_MTORC1_SIGNALING	57	0.193	1.079	0.35685483	0.6651589	1	280	tags=21%, lis
HP_RECURRENT_VIRAL_INFECTIONS	16	0.290	1.078	0.35341364	0.6649838	1	561	tags=44%, lis
HALLMARK_FATTY_ACID_METABOLISM	26	0.239	1.078	0.36938775	0.66407067	1	302	tags=31%, lis
GOMF_CATION_TRANSMEMBRANE_TRANSPORTER_ACTIVITY	56	0.193	1.077	0.3783784	0.6665346	1	641	tags=46%, lis
GOBP_CELLULAR_PROTEIN_CONTAINING_COMPLEX_ASSEMBLY	191	0.146	1.075	0.33941606	0.6693239	1	486	tags=30%, lis
GOBP_REGULATION_OF_MYELOID_LEUKOCYTE_MEDIATED_IMMUNITY	17	0.282	1.074	0.3585746	0.6698785	1	548	tags=41%, lis
GOCC_AUTOPHAGOSOME	24	0.251	1.074	0.37698412	0.67063916	1	420	tags=33%, lis
GOBP_REGULATION_OF_CELL_ACTIVATION	94	0.171	1.073	0.35452795	0.67050564	1	273	tags=20%, lis
GOBP_REGULATION_OF_PROTEASOMAL_PROTEIN_CATABOLIC_PROCESS	46	0.205	1.073	0.37125748	0.6700673	1	532	tags=39%, lis
GOBP_REGULATION_OF_PROTEIN_TYROSINE_KINASE_ACTIVITY	16	0.293	1.072	0.385567	0.6712082	1	493	tags=38%, lis
GOCC_CLATHRIN_COATED_VESICLE_MEMBRANE	20	0.262	1.072	0.38178295	0.6705887	1	257	tags=25%, lis
GOBP_TRANSITION_METAL_ION_TRANSPORT	21	0.256	1.071	0.37236086	0.67064184	1	60	tags=14%, lis
GOBP_REGULATION_OF_VIRAL_LIFE_CYCLE	24	0.250	1.071	0.36456212	0.6700822	1	274	tags=25%, lis
GOBP_ENTRY_INTO_HOST	22	0.258	1.071	0.3562753	0.6693083	1	203	tags=23%, lis
GOCC_ENDOSOME_MEMBRANE	110	0.161	1.069	0.36131388	0.6737903	1	632	tags=39%, lis
REACTOME_LEISHMANIA_INFECTION	50	0.200	1.069	0.36758894	0.6730815	1	417	tags=32%, lis
KEGG_LEUKOCYTE_TRANSENDOTHELIAL_MIGRATION	27	0.237	1.068	0.36268345	0.6724331	1	689	tags=52%, lis
HP_ABNORMALITY_OF_THE_PHARYNX	42	0.206	1.067	0.38508064	0.675007	1	163	tags=19%, lis
GOBP_CATION_TRANSPORT	125	0.157	1.066	0.34935305	0.6755684	1	641	tags=40%, lis
GOBP_TRANSLATIONAL_INITIATION	28	0.233	1.066	0.34911242	0.6749725	1	1035	tags=75%, lis
HP_TELECANTHUS	19	0.267	1.066	0.38381743	0.6743888	1	290	tags=32%, lis
HP_MYALGIA	23	0.251	1.065	0.371134	0.6755339	1	284	tags=30%, lis
HP_METABOLIC_ACIDOSIS	18	0.269	1.064	0.3916501	0.67581236	1	841	tags=67%, lis
HP_ABNORMALITY_OF_FACIAL_SOFT_TISSUE	48	0.196	1.064	0.36015326	0.6752365	1	585	tags=42%, lis
HP_DISTAL_AMYOTROPHY	18	0.272	1.064	0.3830645	0.6742973	1	454	tags=39%, lis
GOBP_CELLULAR_MACROMOLECULE_CATABOLIC_PROCESS	237	0.138	1.064	0.32553956	0.673709	1	379	tags=22%, lis

GOCC_RECYCLING_ENDOSOME_MEMBRANE	18	0.267	1.064	0.3821138	0.6734272	1	965	tags=72%, lis
GOBP_T_CELL_ACTIVATION_INVOLVED_IN_IMMUNE_RESPONSE	25	0.238	1.063	0.39009902	0.67413056	1	187	tags=20%, lis
GOCC_LATE_ENDOSOME	54	0.193	1.062	0.38771594	0.6750409	1	622	tags=41%, lis
GOBP_APOPTOTIC_PROCESS	317	0.131	1.062	0.32922536	0.6751895	1	440	tags=25%, lis
HP_ABNORMALITY_OF_THE_TONGUE	35	0.218	1.060	0.35077518	0.6780233	1	107	tags=17%, lis
GOBP_POSITIVE_REGULATION_OF_TRANSMEMBRANE_TRANSPORT	32	0.217	1.060	0.38878143	0.6778596	1	353	tags=22%, lis
HP_CEREBELLAR_HYPOPLASIA	25	0.236	1.059	0.37574553	0.67879856	1	535	tags=40%, lis
GOBP_NEGATIVE_REGULATION_OF_CELL_GROWTH	32	0.222	1.058	0.40874526	0.6782821	1	436	tags=31%, lis
GOBP_POSITIVE_REGULATION_OF_SIGNALING	259	0.134	1.054	0.3694853	0.6867548	1	255	tags=16%, lis
GOBP_REGULATION_OF_CATABOLIC_PROCESS	214	0.140	1.054	0.3584559	0.68609685	1	363	tags=22%, lis
HP_LOW_SET_POSTERIORLY_ROTATED_EARS	34	0.213	1.054	0.37575758	0.6863117	1	128	tags=9%, list-
HALLMARK_HYPOXIA	39	0.206	1.052	0.39534885	0.6883803	1	104	tags=13%, lis
GOMF_CHAPERONE_BINDING	18	0.262	1.052	0.36511156	0.6881015	1	369	tags=33%, lis
HP_COUGH	25	0.240	1.051	0.38636363	0.6886169	1	220	tags=20%, lis
HP_ABNORMAL_TONGUE_MORPHOLOGY	28	0.235	1.051	0.4012474	0.6891829	1	98	tags=18%, lis
HP_ABNORMAL_CELLULAR_PHENOTYPE	103	0.161	1.050	0.3869159	0.6900184	1	460	tags=30%, lis
HP_PARAPLEGIA_PARAPARESIS	35	0.215	1.047	0.39591837	0.6946511	1	442	tags=31%, lis
GOBP_CHEMICAL_HOMEOSTASIS	152	0.147	1.047	0.3745583	0.6941778	1	399	tags=22%, lis
HP_CLEFT_LIP	17	0.269	1.047	0.39503816	0.6940646	1	86	tags=18%, lis
GOBP_PROTEIN_CONTAINING_COMPLEX_SUBUNIT_ORGANIZATION	314	0.130	1.047	0.3957219	0.69326913	1	502	tags=28%, lis
GOBP_POSITIVE_REGULATION_OF_CELL_POPULATION_PROLIFERATION	113	0.157	1.046	0.39963168	0.6939123	1	267	tags=18%, lis
GOBP_MYELOID_CELL_HOMEOSTASIS	30	0.226	1.044	0.3752418	0.69675714	1	357	tags=30%, lis
GOBP_NEGATIVE_REGULATION_OF_LYMPHOCYTE_ACTIVATION	27	0.230	1.043	0.4	0.69796467	1	273	tags=22%, lis
GOBP_POSITIVE_REGULATION_OF_INTERLEUKIN_6_PRODUCTION	19	0.262	1.042	0.4039604	0.7000557	1	413	tags=37%, lis
HP_POLYMICROGYRIA	32	0.221	1.041	0.39883268	0.70030326	1	301	tags=25%, lis
GOBP_POSITIVE_REGULATION_OF_PHAGOCYTOSIS	23	0.246	1.041	0.39558232	0.6999843	1	484	tags=39%, lis
HP_ABNORMAL_CIRCULATING_IGA_LEVEL	15	0.282	1.040	0.3726708	0.70080173	1	481	tags=40%, lis
GOBP_APOPTOTIC_MITOCHONDRIAL_CHANGES	28	0.225	1.039	0.40792078	0.70136315	1	503	tags=39%, lis
GOMF_CARBOXYLIC_ACID_BINDING	18	0.268	1.038	0.40796965	0.70362145	1	256	tags=28%, lis
HP_CONSTIPATION	48	0.193	1.038	0.41883767	0.70302397	1	368	tags=27%, lis
GOBP_HOMEOSTASIS_OF_NUMBER_OF_CELLS	47	0.194	1.037	0.4169884	0.7044097	1	397	tags=30%, lis
GOBP_REGULATION_OF_ANATOMICAL_STRUCTURE_MORPHOGENESIS	149	0.145	1.035	0.3992095	0.7067628	1	267	tags=19%, lis
GOBP_NEGATIVE_REGULATION_OF_CELL_DEATH	142	0.148	1.035	0.39423078	0.7076628	1	353	tags=20%, lis
GOBP_REGULATION_OF_LYMPHOCYTE_ACTIVATION	78	0.169	1.034	0.42352942	0.7070439	1	273	tags=19%, lis
GOBP_MACROAUTOPHAGY	67	0.175	1.034	0.40307102	0.7074651	1	524	tags=31%, lis
GOBP_CELLULAR_MONOVALENT_INORGANIC_CATION_HOMEOSTASIS	19	0.259	1.032	0.4157088	0.7108633	1	664	tags=47%, lis
HALLMARK_UV_RESPONSE_UP	38	0.209	1.031	0.41104296	0.71093756	1	440	tags=34%, lis
GOBP_INTERLEUKIN_1_BETA_PRODUCTION	15	0.277	1.030	0.414	0.7120891	1	33	tags=13%, lis
GOBP_NEGATIVE_REGULATION_OF_CELL_ADHESION	39	0.198	1.030	0.40836653	0.71150637	1	399	tags=28%, lis
GOBP_REGULATION_OF_CYSTEINE_TYPE_ENDOPEPTIDASE_ACTIVITY	38	0.207	1.028	0.41015625	0.71485925	1	503	tags=39%, lis
REACTOME_RRNA_PROCESSING	16	0.269	1.025	0.4319066	0.7218976	1	656	tags=50%, lis
HP_ABNORMALITY_OF_THE_NECK	73	0.169	1.024	0.40518963	0.7217475	1	330	tags=18%, lis
PID_P73PATHWAY	18	0.264	1.023	0.442	0.72343117	1	763	tags=61%, lis
HP_ABNORMAL_RECTUM_MORPHOLOGY	22	0.250	1.022	0.42528737	0.7257997	1	841	tags=64%, lis
HP_SARCOMA	26	0.228	1.021	0.42553192	0.72546864	1	107	tags=15%, lis
HP_COLITIS	15	0.274	1.020	0.41825095	0.7274391	1	124	tags=20%, lis
HP_ABNORMAL_VENOUS_MORPHOLOGY	16	0.274	1.020	0.4522293	0.72656065	1	1354	tags=94%, lis
GOBP_POSITIVE_REGULATION_OF_I_KAPPAB_KINASE_NF_KAPPAB_SIGNALING	29	0.225	1.020	0.46218488	0.7256885	1	763	tags=52%, lis
GOBP_NEGATIVE_REGULATION_OF_CELL_ACTIVATION	34	0.209	1.020	0.43856332	0.725453	1	273	tags=24%, lis
GOBP_EPITHELIUM_DEVELOPMENT	137	0.148	1.018	0.43939394	0.72774297	1	266	tags=18%, lis
HP_DECREASED_FACIAL_EXPRESSION	17	0.253	1.018	0.43333334	0.7276606	1	364	tags=29%, lis
GOMF_CELL_ADHESION_MOLECULE_BINDING	93	0.160	1.018	0.41325536	0.72697	1	326	tags=23%, lis
KEGG_CHEMOKINE_SIGNALING_PATHWAY	46	0.189	1.017	0.43040293	0.7270362	1	206	tags=15%, lis
GOBP_POSITIVE_REGULATION_OF_ACTIN_FILAMENT_POLYMERIZATION	22	0.245	1.017	0.4248497	0.7270105	1	632	tags=55%, lis
HP_ABNORMAL_PENIS_MORPHOLOGY	75	0.170	1.016	0.43106797	0.7283377	1	556	tags=32%, lis
GOBP_NEGATIVE_REGULATION_OF_IMMUNE_RESPONSE	24	0.232	1.015	0.4372549	0.7298992	1	349	tags=29%, lis
HP_ABNORMALITY_OF_THE_SEPTUM_PELLUCIDUM	22	0.242	1.011	0.43326885	0.736464	1	520	tags=41%, lis
HALLMARK_ESTROGEN_RESPONSE_LATE	28	0.221	1.010	0.43495935	0.73838055	1	139	tags=18%, lis
GOBP_NEGATIVE_REGULATION_OF_MRNA_METABOLIC_PROCESS	18	0.256	1.010	0.45364892	0.73782736	1	452	tags=39%, lis
HP_DUPLICATION_OF_HAND_BONES	16	0.269	1.010	0.41104296	0.7371118	1	284	tags=31%, lis
HP_ABNORMAL_GLUCOSE_HOMEOSTASIS	60	0.176	1.009	0.46628132	0.7381391	1	654	tags=43%, lis
HP_DISINHIBITION	38	0.203	1.007	0.4665392	0.7403372	1	585	tags=42%, lis
HP_POOR_HEAD_CONTROL	24	0.242	1.007	0.43486974	0.74069816	1	979	tags=79%, lis
GOBP_REGULATION_OF_CELLULAR_LOCALIZATION	134	0.147	1.006	0.46307385	0.74017584	1	520	tags=30%, lis
HP_ABNORMALITY_OF_THE_CEREBRAL_VENTRICLES	117	0.150	1.006	0.44337812	0.73988223	1	690	tags=42%, lis
GOBP_LYSOSOME_LOCALIZATION	16	0.264	1.004	0.4387755	0.7441535	1	548	tags=44%, lis
GOBP_AMIDE_BIOSYNTHETIC_PROCESS	127	0.147	1.002	0.41379312	0.7468823	1	753	tags=46%, lis
GOBP_EPHRIN_RECEPTOR_SIGNALING_PATHWAY	16	0.266	1.002	0.42524272	0.74641645	1	544	tags=50%, lis
GOBP_ORGANIC_ANION_TRANSPORT	27	0.228	1.001	0.43312103	0.747406	1	2	tags=4%, list-
GOBP_REGULATION_OF_ION_TRANSPORT	164	0.138	1.001	0.4664179	0.7467824	1	403	tags=24%, lis
GOCC_SPINDLE_POLE	23	0.236	1.000	0.46169773	0.7477451	1	426	tags=35%, lis
HP_MOTOR_SEIZURE	38	0.203	0.999	0.45686275	0.7490359	1	364	tags=29%, lis
GOMF_UBIQUITIN_LIKE_PROTEIN_LIGASE_BINDING	70	0.169	0.996	0.46911198	0.7539875	1	524	tags=33%, lis
GOCC_RIBONUCLEOPROTEIN_GRANULE	58	0.176	0.994	0.46755725	0.7576634	1	334	tags=24%, lis
GOBP_MRNA_METABOLIC_PROCESS	164	0.137	0.994	0.47113594	0.7573038	1	359	tags=22%, lis
GOBP_MOVEMENT_IN_HOST_ENVIRONMENT	31	0.213	0.994	0.4639376	0.756498	1	326	tags=26%, lis
HP_RESPIRATORY_DISTRESS	21	0.237	0.994	0.46464646	0.7558127	1	220	tags=19%, lis
GOBP_REGULATION_OF_LIPID_METABOLIC_PROCESS	58	0.176	0.994	0.46489564	0.7553174	1	69	tags=10%, lis
GOBP_ORGANONITROGEN_COMPOUND_BIOSYNTHETIC_PROCESS	244	0.129	0.994	0.49340865	0.75490814	1	753	tags=44%, lis

GOCC_LATE_ENDOSOME_MEMBRANE	36	0.199	0.993	0.4741036	0.7561306	1	687	tags=47%, lis
GOBP_REGULATION_OF_PROTEIN_CATABOLIC_PROCESS	84	0.160	0.992	0.49196786	0.75679034	1	425	tags=29%, lis
HP_GENERALIZED_HYPOTONIA	149	0.142	0.988	0.48737863	0.7633147	1	550	tags=33%, lis
GOBP_ORGANIC_ACID_METABOLIC_PROCESS	124	0.148	0.986	0.5069307	0.7671142	1	302	tags=19%, lis
GOBP_EPITHELIAL_TUBE_MORPHOGENESIS	22	0.234	0.986	0.47022587	0.76679206	1	136	tags=18%, lis
HP_ABNORMAL_MYOCARDIUM_MORPHOLOGY	53	0.181	0.985	0.46934867	0.7672523	1	691	tags=43%, lis
GOBP_NEGATIVE_REGULATION_OF_SIGNALING	203	0.132	0.985	0.5	0.7676304	1	255	tags=16%, lis
GOBP_POSITIVE_REGULATION_OF_REACTIVE_OXYGEN_SPECIES_METABOLIC_PROCESS	24	0.229	0.983	0.47524753	0.7701193	1	127	tags=17%, lis
GOBP_POSITIVE_REGULATION_OF_MRNA_METABOLIC_PROCESS	24	0.224	0.981	0.45773196	0.77352345	1	528	tags=42%, lis
GOBP_REGULATION_OF_PEPTIDYL_SERINE_PHOSPHORYLATION	25	0.219	0.981	0.47686118	0.77334064	1	15	tags=8%, list-
HALLMARK_DNA_REPAIR	28	0.220	0.981	0.46052632	0.77271587	1	888	tags=68%, lis
GOBP_LEUKOCYTE_HOMEOSTASIS	19	0.247	0.981	0.4748491	0.77195156	1	575	tags=47%, lis
GOBP_ESTABLISHMENT_OF_CELL_POLARITY	23	0.228	0.980	0.48015872	0.7721259	1	689	tags=52%, lis
GOMF_MRNA_3_UTR_BINDING	17	0.260	0.979	0.46625766	0.7741491	1	476	tags=41%, lis
GOBP_HORMONE_TRANSPORT	26	0.219	0.979	0.47165993	0.7735416	1	213	tags=23%, lis
HP_HYPERTRICHOSIS	41	0.189	0.978	0.502008	0.7727923	1	350	tags=27%, lis
GOBP_PROTEIN_CATABOLIC_PROCESS	195	0.131	0.978	0.50455374	0.77187485	1	409	tags=23%, lis
GOBP_CELLULAR_IRON_ION_HOMEOSTASIS	20	0.242	0.978	0.4789357	0.7712793	1	378	tags=35%, lis
GOBP_REGULATION_OF_REGULATED_SECRETORY_PATHWAY	16	0.256	0.978	0.46966732	0.77073526	1	318	tags=31%, lis
GOBP_POSITIVE_REGULATION_OF_ANION_TRANSPORT	73	0.164	0.978	0.4895238	0.7701066	1	689	tags=47%, lis
HP_TALIPES_EQUINOVARUS	33	0.208	0.978	0.5029821	0.76947737	1	2	tags=3%, list-
HP_ABNORMAL_CIRCULATING_NITROGEN_COMPOUND_CONCENTRATION	17	0.252	0.977	0.4834308	0.7702304	1	130	tags=18%, lis
GOBP_ORGANONITROGEN_COMPOUND_CATABOLIC_PROCESS	228	0.128	0.976	0.52355075	0.77197593	1	379	tags=22%, lis
GOBP_PHOSPHATIDYLINOSITOL_BIOSYNTHETIC_PROCESS	17	0.256	0.974	0.4894027	0.77468145	1	698	tags=59%, lis
GOBP_INTRACELLULAR_PROTEIN_TRANSPORT	196	0.132	0.973	0.5055147	0.77625793	1	563	tags=32%, lis
HP_THICKENED_SKIN	50	0.184	0.972	0.4733728	0.77659994	1	691	tags=44%, lis
HP_APLASIA_HYPOPLASIA_INVOLVING_BONES_OF_THE_FEET	33	0.202	0.972	0.4893204	0.7764117	1	2	tags=3%, list-
GOBP_TUMOR_NECROSIS_FACTOR_SUPERFAMILY_CYTOKINE_PRODUCTION	28	0.214	0.972	0.48262548	0.7762472	1	413	tags=32%, lis
HP_ABNORMALITY_OF_EXTRAPYRAMIDAL_MOTOR_FUNCTION	39	0.193	0.971	0.5252525	0.7759284	1	490	tags=33%, lis
GOBP_REGULATION_OF_METAL_ION_TRANSPORT	27	0.213	0.970	0.50951374	0.7780363	1	549	tags=37%, lis
GOMF_KINASE_REGULATOR_ACTIVITY	44	0.188	0.970	0.51167315	0.7771228	1	255	tags=18%, lis
GOBP_PROTEIN_LOCALIZATION_TO_NUCLEUS	52	0.176	0.970	0.502	0.77632564	1	187	tags=13%, lis
GOBP_ERYTHROCYTE_HOMEOSTASIS	24	0.223	0.970	0.48288974	0.7757076	1	334	tags=29%, lis
GOBP_CELLULAR_MACROMOLECULE_LOCALIZATION	327	0.118	0.966	0.5377532	0.7825378	1	515	tags=28%, lis
GOCC_SECRETORY_VESICLE	185	0.131	0.966	0.5283019	0.7816377	1	416	tags=22%, lis
GOMF_CADHERIN_BINDING	73	0.164	0.966	0.5104762	0.7814974	1	326	tags=22%, lis
HP_ABNORMALITY_OF_CIRCULATING_ENZYME_LEVEL	27	0.209	0.966	0.5	0.78077024	1	290	tags=26%, lis
GOBP_INTRINSIC_APOPTOTIC_SIGNALING_PATHWAY_IN_RESPONSE_TO_ENDOPLASMIC_RETICULU	17	0.251	0.965	0.47638604	0.7804553	1	440	tags=35%, lis
GOCC_ENDOCYTIC_VESICLE_MEMBRANE	36	0.194	0.965	0.4979424	0.77981806	1	19	tags=8%, list-
GOCC_INTRACELLULAR_PROTEIN_CONTAINING_COMPLEX	150	0.137	0.965	0.5047801	0.7795163	1	283	tags=19%, lis
GOBP_CYTOKINESIS	22	0.229	0.964	0.49689442	0.7811408	1	231	tags=23%, lis
GOBP_SMALL_MOLECULE_METABOLIC_PROCESS	226	0.127	0.963	0.5626204	0.7814409	1	318	tags=19%, lis
GOBP_CELLULAR_RESPONSE_TO_EXTERNAL_STIMULUS	54	0.173	0.963	0.489011	0.78093034	1	420	tags=30%, lis
REACTOME_SIGNALING_BY_VEGF	31	0.204	0.961	0.515625	0.78362036	1	500	tags=35%, lis
HALLMARK_PROTEIN_SECRETION	30	0.206	0.961	0.50630254	0.7828202	1	697	tags=50%, lis
GOBP_CELL_DIVISION	70	0.162	0.958	0.5242537	0.788994	1	300	tags=20%, lis
GOBP_CELL_AGING	18	0.246	0.957	0.49593496	0.789761	1	127	tags=17%, lis
GOBP_REGULATION_OF_PROTEIN_SERINE_THREONINE_KINASE_ACTIVITY	81	0.156	0.956	0.540856	0.7923599	1	127	tags=11%, lis
GOBP_POSITIVE_REGULATION_OF_MEMBRANE_PERMEABILITY	19	0.238	0.955	0.50209206	0.79337746	1	503	tags=42%, lis
REACTOME_L1CAM_INTERACTIONS	17	0.249	0.953	0.494	0.7966908	1	92	tags=18%, lis
HALLMARK_COMPLEMENT	47	0.181	0.953	0.5188679	0.79598475	1	273	tags=19%, lis
GOCC_PHAGOCYTIC_VESICLE_MEMBRANE	23	0.227	0.952	0.4950495	0.79580414	1	576	tags=39%, lis
HP_RETROGNATHIA	36	0.197	0.952	0.515748	0.7949798	1	79	tags=6%, list-
GOBP_RESPONSE_TO_METAL_ION	53	0.179	0.951	0.5372849	0.7961601	1	254	tags=19%, lis
GOBP_NEGATIVE_REGULATION_OF_PROTEIN_CONTAINING_COMPLEX_ASSEMBLY	20	0.230	0.951	0.52208835	0.795282	1	1629	tags=100%, l
GOBP_REGULATION_OF_MITOCHONDRIAL_MEMBRANE_PERMEABILITY	19	0.238	0.951	0.49905124	0.7944636	1	503	tags=42%, lis
KEGG_P53_SIGNALING_PATHWAY	17	0.251	0.950	0.5247148	0.7956651	1	375	tags=35%, lis
GOBP_ACTIN_POLYMERIZATION_OR_DEPOLYMERIZATION	46	0.179	0.950	0.5551181	0.7955775	1	674	tags=46%, lis
HP_DYSTONIA	75	0.158	0.949	0.52224374	0.79613405	1	324	tags=23%, lis
REACTOME_ANTI_INFLAMMATORY_RESPONSE_FAVOURING_LEISHMANIA_PARASITE_INFECTION	15	0.259	0.948	0.5362319	0.79675066	1	353	tags=33%, lis
HP_AUTOIMMUNE_ANTIBODY_POSITIVITY	22	0.223	0.948	0.512987	0.79649746	1	397	tags=32%, lis
HP_ABNORMALITY_OF_PRENATAL_DEVELOPMENT_OR_BIRTH	83	0.154	0.948	0.57251906	0.79652494	1	358	tags=20%, lis
REACTOME_CELLULAR_RESPONSE_TO_STARVATION	19	0.241	0.947	0.5224171	0.79626137	1	685	tags=47%, lis
GOBP_MITOCHONDRIAL_OUTER_MEMBRANE_PERMEABILIZATION	17	0.244	0.947	0.52277225	0.796057	1	360	tags=35%, lis
GOBP_PROCESS_UTILIZING_AUTOPHAGIC_MECHANISM	110	0.144	0.946	0.5336976	0.7980132	1	524	tags=29%, lis
GOBP_REGULATION_OF_MEMBRANE_PERMEABILITY	19	0.238	0.945	0.5123457	0.7991778	1	503	tags=42%, lis
HP_DECREASED_HEAD_CIRCUMFERENCE	174	0.130	0.944	0.54971856	0.7988756	1	317	tags=18%, lis
HP_SPASTIC_PARAPLEGIA	20	0.230	0.944	0.54059404	0.7991484	1	520	tags=40%, lis
HP_DYSKINESIA	40	0.187	0.944	0.52556235	0.7982378	1	585	tags=40%, lis
GOBP_ANION_TRANSMEMBRANE_TRANSPORT	54	0.169	0.942	0.5717054	0.80112094	1	323	tags=22%, lis
HP_THORACIC_HYPOPLASIA	15	0.257	0.940	0.524	0.80342525	1	1016	tags=80%, lis
GOBP_MONOVALENT_INORGANIC_CATION_HOMEOSTASIS	22	0.231	0.940	0.5383104	0.80259275	1	664	tags=45%, lis
GOBP_POSITIVE_REGULATION_OF_VIRAL_PROCESS	18	0.237	0.938	0.53488374	0.8068892	1	274	tags=22%, lis
GOBP_PRODUCTION_OF_MOLECULAR_MEDIATOR_OF_IMMUNE_RESPONSE	37	0.187	0.938	0.5188679	0.8059837	1	295	tags=19%, lis
GOBP_REGULATION_OF_PROTEASOMAL_UBIQUITIN_DEPENDENT_PROTEIN_CATABOLIC_PROCESS	35	0.195	0.937	0.5705263	0.80605304	1	532	tags=37%, lis
GOBP_REGULATION_OF_CELLULAR_AMIDE_METABOLIC_PROCESS	82	0.152	0.937	0.548	0.8058334	1	564	tags=37%, lis
HP_UNUSUAL_FUNGAL_INFECTION	17	0.245	0.937	0.52929294	0.8060287	1	481	tags=41%, lis
GOBP_NEGATIVE_REGULATION_OF_PROTEIN_MODIFICATION_BY_SMALL_PROTEIN_CONJUGATION	15	0.254	0.936	0.53488374	0.8060502	1	1381	tags=93%, lis
GOCC_TRANS_GOLGI_NETWORK_MEMBRANE	21	0.222	0.935	0.5302419	0.8084254	1	19	tags=10%, lis

GOBP_RESPONSE_TO_RADIATION	67	0.160	0.933	0.56772906	0.8107335	1	391	tags=24%, lis
GOBP_RESPONSE_TO_HEAT	27	0.205	0.933	0.5338491	0.80989254	1	618	tags=41%, lis
HP_HIGH_FOREHEAD	49	0.173	0.932	0.57831323	0.8108917	1	79	tags=6%, list-
GOBP_POSITIVE_REGULATION_OF_PROTEIN_PHOSPHORYLATION	127	0.137	0.932	0.5685185	0.8102973	1	127	tags=9%, list-
GOMF_SNARE_BINDING	15	0.248	0.931	0.5252525	0.8103204	1	336	tags=33%, lis
GOBP_VESICLE_TARGETING_TO_FROM_OR_WITHIN_GOLGI	17	0.235	0.931	0.52310926	0.8096569	1	1454	tags=94%, lis
HP_DYSPHAGIA	65	0.163	0.931	0.55212355	0.8088006	1	344	tags=22%, lis
GOBP_POSITIVE_REGULATION_OF_PROTEIN_KINASE_ACTIVITY	81	0.150	0.928	0.5542636	0.8139047	1	127	tags=10%, lis
GOBP_ORGANIC_ACID_CATABOLIC_PROCESS	15	0.256	0.928	0.5402062	0.81462383	1	174	tags=27%, lis
GOBP_ALTERNATIVE_MRNA_SPLICING_VIA_SPLICEOSOME	18	0.233	0.925	0.5397149	0.81969255	1	348	tags=28%, lis
HP_ONSET	163	0.130	0.923	0.6093458	0.8222874	1	364	tags=21%, lis
REACTOME_TRANSCRIPTIONAL_REGULATION_BY_TP53	83	0.149	0.923	0.61078995	0.82161814	1	896	tags=58%, lis
HP_ABNORMAL_TRACHEOBRONCHIAL_MORPHOLOGY	43	0.174	0.922	0.5694716	0.8228282	1	633	tags=42%, lis
GOCC_COATED_VESICLE_MEMBRANE	35	0.190	0.922	0.5686654	0.82244176	1	284	tags=20%, lis
GOBP_ENDOCYTOSIS	103	0.141	0.921	0.5881226	0.8229624	1	205	tags=12%, lis
GOBP_REGULATION_OF_T_CELL_DIFFERENTIATION	24	0.214	0.921	0.57057655	0.8223171	1	273	tags=21%, lis
HP_ABNORMALITY_OF_GLOBE_SIZE	32	0.197	0.921	0.5285996	0.8222376	1	1005	tags=72%, lis
HP_WIDE_NASAL_BRIDGE	65	0.157	0.920	0.5694165	0.8220609	1	368	tags=20%, lis
GOBP_MALE_SEX_DIFFERENTIATION	18	0.229	0.919	0.550495	0.8225385	1	712	tags=56%, lis
HP_ABNORMALITY_ON_PULMONARY_FUNCTION_TESTING	20	0.229	0.919	0.5598377	0.8220017	1	220	tags=20%, lis
HP_GENERALIZED_ONSET_SEIZURE	45	0.176	0.919	0.560396	0.8211689	1	672	tags=42%, lis
HP_ABNORMALITY_OF_THE_PERIPHERAL_NERVOUS_SYSTEM	30	0.200	0.918	0.54990214	0.8224426	1	324	tags=27%, lis
HP_ABNORMALITY_OF_THE_ANUS	29	0.197	0.918	0.56122446	0.8223641	1	290	tags=24%, lis
HP_ABNORMALITY_OF_THE_PALPEBRAL_FISSURES	103	0.142	0.915	0.5857418	0.827712	1	358	tags=19%, lis
GOMF_GDP_BINDING	17	0.236	0.915	0.552	0.826964	1	632	tags=47%, lis
HP_OPTIC_ATROPHY	74	0.155	0.914	0.624031	0.8270314	1	698	tags=47%, lis
GOBP_PHOSPHATIDYLINOSITOL_METABOLIC_PROCESS	27	0.203	0.914	0.5714286	0.8270287	1	735	tags=52%, lis
HP_CLEFT_SOFT_PALATE	18	0.231	0.914	0.5574713	0.8266699	1	344	tags=28%, lis
BIOCARTA_HIVNEF_PATHWAY	15	0.243	0.913	0.55531454	0.8262453	1	275	tags=27%, lis
HP_CELLULITIS	15	0.245	0.913	0.5625	0.82587504	1	587	tags=53%, lis
HP_ABNORMALITY_OF_THE_UVULA	18	0.231	0.913	0.54043394	0.8252985	1	344	tags=28%, lis
GOBP_PEPTIDYL_TYROSINE_DEPHOSPHORYLATION	15	0.244	0.912	0.5469729	0.8250651	1	69	tags=13%, lis
GOBP_POSITIVE_REGULATION_OF_PEPTIDYL_TYROSINE_PHOSPHORYLATION	26	0.209	0.912	0.5594542	0.82568014	1	55	tags=12%, lis
GOBP_ORGANELLE_ASSEMBLY	90	0.143	0.910	0.6272727	0.82784045	1	503	tags=30%, lis
GOBP_REGULATION_OF_UBIQUITIN_DEPENDENT_PROTEIN_CATABOLIC_PROCESS	36	0.185	0.910	0.5854167	0.8277536	1	582	tags=39%, lis
HP_GASTROESOPHAGEAL_REFLUX	51	0.162	0.908	0.57894737	0.8303215	1	337	tags=24%, lis
PID_TCR_PATHWAY	17	0.237	0.906	0.54545456	0.83447176	1	1028	tags=76%, lis
HP_ABNORMALITY_OF_RETINAL_PIGMENTATION	31	0.199	0.906	0.588694	0.83362997	1	499	tags=32%, lis
HP_INTELLECTUAL_DISABILITY_MILD	63	0.158	0.905	0.61714286	0.83332586	1	691	tags=43%, lis
GOBP_REGULATION_OF_PROTEIN_CONTAINING_COMPLEX_ASSEMBLY	82	0.147	0.905	0.6403162	0.83241093	1	424	tags=27%, lis
HP_SPASTICITY	125	0.133	0.904	0.6516854	0.833695	1	374	tags=21%, lis
REACTOME_COPI_DEPENDENT_GOLGI_TO_ER_RETROGRADE_TRAFFIC	18	0.228	0.904	0.55666006	0.8332933	1	1633	tags=100%, l
HP_HYPERREFLEXIA	77	0.150	0.903	0.6230769	0.8335339	1	566	tags=35%, lis
REACTOME_SIGNALING_BY_GPCR	61	0.157	0.903	0.5870842	0.83322096	1	206	tags=13%, lis
GOBP_REGULATION_OF_ANION_TRANSPORT	120	0.133	0.903	0.62818	0.83331	1	403	tags=24%, lis
GOBP_PROTEIN_MODIFICATION_BY_SMALL_PROTEIN_CONJUGATION	162	0.125	0.902	0.63770795	0.8327227	1	453	tags=25%, lis
GOCC_CATALYTIC_COMPLEX	267	0.114	0.902	0.6606822	0.8324712	1	595	tags=33%, lis
GOBP_REGULATION_OF_SIGNAL_TRANSDUCTION_BY_P53_CLASS_MEDIATOR	32	0.189	0.902	0.589942	0.8326923	1	385	tags=28%, lis
HP_COGNITIVE_IMPAIRMENT	79	0.146	0.901	0.5947467	0.83204347	1	691	tags=42%, lis
GOBP_POSITIVE_REGULATION_OF_CELL_ACTIVATION	61	0.160	0.897	0.6037736	0.83951956	1	257	tags=18%, lis
HP_CLINICAL_COURSE	206	0.121	0.896	0.6571429	0.84186184	1	364	tags=21%, lis
GOBP_ENDOTHELIUM_DEVELOPMENT	25	0.205	0.895	0.5697674	0.8431431	1	136	tags=16%, lis
GOBP_PROTEIN_DEPOLYMERIZATION	17	0.233	0.895	0.591716	0.84228355	1	674	tags=47%, lis
GOBP_CATION_TRANSMEMBRANE_TRANSPORT	94	0.141	0.894	0.6247544	0.84189653	1	653	tags=40%, lis
HP_ABNORMAL_BLOOD_INORGANIC_CATION_CONCENTRATION	21	0.216	0.894	0.57749075	0.8423869	1	471	tags=43%, lis
REACTOME_G_ALPHA_Q_SIGNALLING_EVENTS	22	0.210	0.893	0.56725144	0.84238017	1	527	tags=36%, lis
HP_RECURRENT_SKIN_INFECTIONS	26	0.198	0.893	0.57171315	0.8422036	1	481	tags=38%, lis
GOBP_REGULATION_OF_PROTEIN_STABILITY	58	0.161	0.892	0.60192305	0.8420721	1	236	tags=16%, lis
HP_ASTHMA	18	0.229	0.892	0.5762712	0.84136295	1	353	tags=33%, lis
HP_ABNORMAL_PULMONARY_INTERSTITIAL_MORPHOLOGY	21	0.215	0.892	0.5741127	0.84073824	1	107	tags=14%, lis
KEGG_EPITHELIAL_CELL_SIGNALING_IN_HELICOBACTER_PYLORI_INFECTION	17	0.228	0.892	0.5731225	0.83998686	1	534	tags=41%, lis
REACTOME_CILIUM_ASSEMBLY	22	0.213	0.892	0.5711575	0.8391011	1	503	tags=36%, lis
GOMF_PROTEIN_TYROSINE_PHOSPHATASE_ACTIVITY	15	0.244	0.891	0.5746888	0.8407275	1	69	tags=13%, lis
GOBP_POSITIVE_REGULATION_OF_SECRETION	23	0.210	0.890	0.58677685	0.84110904	1	377	tags=30%, lis
HP_INABILITY_TO_WALK	25	0.204	0.889	0.59807694	0.843087	1	393	tags=32%, lis
HP_BIFID_UVULA	18	0.231	0.888	0.610998	0.84450424	1	344	tags=28%, lis
HP_FEEDING_DIFFICULTIES	138	0.128	0.887	0.65849054	0.8443148	1	580	tags=34%, lis
GOMF_LIPID_BINDING	113	0.134	0.887	0.662768	0.84361553	1	256	tags=17%, lis
GOMF_ION_CHANNEL_REGULATOR_ACTIVITY	15	0.240	0.886	0.5693277	0.84411854	1	255	tags=27%, lis
PID_ERA_GENOMIC_PATHWAY	15	0.240	0.886	0.59	0.8435069	1	446	tags=40%, lis
GOBP_POSITIVE_REGULATION_OF_PRODUCTION_OF_MOLECULAR_MEDIATOR_OF_IMMUNE_RESP	19	0.222	0.885	0.5857988	0.8441675	1	45	tags=11%, lis
GOBP_REGULATION_OF_CELL_CYCLE_PROCESS	125	0.132	0.885	0.6585821	0.8436558	1	337	tags=21%, lis
GOBP_T_CELL_DIFFERENTIATION_IN_THYMUS	16	0.240	0.885	0.5627615	0.84300464	1	334	tags=25%, lis
GOBP_CELLULAR_AMIDE_METABOLIC_PROCESS	166	0.122	0.883	0.7083333	0.84579647	1	763	tags=45%, lis
GOBP_LYSOSOMAL_TRANSPORT	23	0.205	0.883	0.5995935	0.8461391	1	549	tags=39%, lis
HP_TRIANGULAR_SHAPED_PHALANGES_OF_THE_HAND	19	0.223	0.882	0.610101	0.84561	1	339	tags=26%, lis
REACTOME_DEVELOPMENTAL_BIOLOGY	148	0.125	0.882	0.6859345	0.8459595	1	502	tags=28%, lis
GOBP_VESICLE_TARGETING	23	0.208	0.881	0.5860735	0.8466175	1	1676	tags=100%, l
GOBP_IRON_ION_HOMEOSTASIS	23	0.208	0.881	0.60721445	0.846433	1	378	tags=30%, lis

HP_INTERICTAL_EEG_ABNORMALITY	33	0.181	0.880	0.63249516	0.8465042	1	404	tags=30%, lis
HP_PARAPLEGIA	21	0.212	0.880	0.6146045	0.84567446	1	520	tags=38%, lis
HP_ANAL_ATRESIA	16	0.231	0.880	0.59166664	0.8450194	1	284	tags=25%, lis
HP_ABNORMALITY_OF_THE_OPTIC_NERVE	97	0.137	0.880	0.67178506	0.84457785	1	698	tags=44%, lis
GOCC_SPECIFIC_GRANULE	39	0.176	0.879	0.6082677	0.8455446	1	416	tags=28%, lis
REACTOME_MITOTIC_PROMETAPHASE	25	0.201	0.878	0.610338	0.8463164	1	128	tags=16%, lis
GOBP_EXTRINSIC_APOPTOTIC_SIGNALING_PATHWAY	47	0.169	0.878	0.63095236	0.84608954	1	420	tags=23%, lis
GOMF_HEAT_SHOCK_PROTEIN_BINDING	22	0.208	0.878	0.6233766	0.8452446	1	369	tags=32%, lis
GOBP_RESPONSE_TO_NUTRIENT	19	0.223	0.877	0.5807128	0.8451349	1	551	tags=37%, lis
GOBP_REGULATION_OF_PROTEIN_KINASE_ACTIVITY	135	0.126	0.877	0.6949459	0.8444071	1	96	tags=8%, list-
REACTOME_RHO_GTPASES_ACTIVATE_WASPS_AND_WAVES	15	0.235	0.877	0.6041237	0.84400815	1	460	tags=40%, lis
HP_LEUKOPENIA	51	0.163	0.877	0.62708336	0.8433546	1	279	tags=20%, lis
HP_ABNORMAL_EMOTION_AFFECT_BEHAVIOR	72	0.149	0.877	0.6641075	0.84284604	1	107	tags=11%, lis
HP_ABNORMALITY_OF_ESOPHAGUS_PHYSIOLOGY	94	0.138	0.876	0.65471697	0.8422265	1	344	tags=21%, lis
PID_ECADHERIN_NASCENT_AJ_PATHWAY	17	0.227	0.872	0.6213592	0.8489541	1	689	tags=47%, lis
GOBP_EMBRYONIC_ORGAN_MORPHOGENESIS	19	0.220	0.872	0.6145833	0.8482786	1	127	tags=16%, lis
GOBP_MITOTIC_CELL_CYCLE	172	0.120	0.871	0.71374047	0.84967947	1	337	tags=20%, lis
GOBP_POSITIVE_REGULATION_OF_PROTEIN_SERINE_THREONINE_KINASE_ACTIVITY	49	0.163	0.870	0.6308594	0.8511656	1	127	tags=10%, lis
HP_INVOLUNTARY_MOVEMENTS	149	0.122	0.869	0.7255639	0.85132396	1	585	tags=33%, lis
HP_PALMOPLANTAR_KERATODERMA	15	0.242	0.867	0.5988024	0.85443455	1	830	tags=60%, lis
HP_ABNORMAL_FACIAL_EXPRESSION	19	0.214	0.864	0.6226804	0.85943437	1	364	tags=26%, lis
GOCC_PERINUCLEAR_REGION_OF_CYTOPLASM	122	0.127	0.863	0.70018977	0.86021835	1	187	tags=11%, lis
GOBP_NEGATIVE_REGULATION_OF_CELLULAR_AMIDE_METABOLIC_PROCESS	41	0.171	0.861	0.65708417	0.86355907	1	726	tags=54%, lis
HP_BRADYKINESIA	15	0.229	0.859	0.61157024	0.8663465	1	585	tags=47%, lis
HP_ABSENT_SPEECH	41	0.172	0.857	0.6432866	0.8688573	1	580	tags=41%, lis
HP_FOCAL_ONSET_SEIZURE	33	0.181	0.857	0.63469386	0.86876833	1	520	tags=33%, lis
GOBP_T_CELL_DIFFERENTIATION	43	0.167	0.856	0.6513944	0.86883086	1	397	tags=26%, lis
HP_NEOPLASM_OF_THE_SKIN	35	0.178	0.856	0.6458333	0.8689463	1	155	tags=14%, lis
GOBP_LEUKOCYTE_APOPTOTIC_PROCESS	21	0.203	0.853	0.6354776	0.87348783	1	494	tags=33%, lis
HP_ABNORMALITY_OF_THE_NASAL_TIP	49	0.153	0.852	0.697446	0.8737974	1	358	tags=24%, lis
GOBP_MACROMOLECULE_CATABOLIC_PROCESS	271	0.108	0.852	0.77205884	0.8732211	1	379	tags=20%, lis
GOBP_REGULATION_OF_HORMONE_LEVELS	39	0.169	0.852	0.6645833	0.87342155	1	525	tags=36%, lis
GOBP_POSITIVE_REGULATION_OF_ERK1_AND_ERK2_CASCADE	24	0.195	0.851	0.65645516	0.87437695	1	116	tags=13%, lis
GOBP_CYTOKINE_PRODUCTION_INVOLVED_IN_IMMUNE_RESPONSE	20	0.211	0.849	0.6368821	0.8762715	1	476	tags=30%, lis
HP_ABNORMAL_RESPIRATORY_SYSTEM_PHYSIOLOGY	165	0.116	0.849	0.7416357	0.87586963	1	316	tags=18%, lis
GOCC_EARLY_ENDOSOME_MEMBRANE	29	0.187	0.848	0.64534885	0.8758603	1	632	tags=45%, lis
HP_APLASIA_HYPOPLASIA_OF_THE_CEREBELLUM	35	0.171	0.848	0.6640159	0.87572604	1	535	tags=34%, lis
REACTOME_PLATELET_ACTIVATION_SIGNALING_AND_AGGREGATION	52	0.154	0.847	0.67622954	0.8776098	1	602	tags=38%, lis
GOMF_CARBOHYDRATE_BINDING	17	0.222	0.846	0.62815124	0.8774934	1	228	tags=24%, lis
GOBP_MEMBRANE_INVAGINATION	18	0.221	0.846	0.646729	0.8766716	1	198	tags=22%, lis
GOBP_NEGATIVE_REGULATION_OF_CELL_DIFFERENTIATION	75	0.141	0.845	0.7053232	0.8776632	1	152	tags=12%, lis
HP_ABNORMAL_GROWTH_HORMONE_LEVEL	20	0.207	0.845	0.6486486	0.8776056	1	561	tags=40%, lis
GOCC_ACTIN_BASED_CELL_PROJECTION	29	0.184	0.844	0.6321839	0.87807804	1	403	tags=24%, lis
GOBP_POSITIVE_REGULATION_OF_MITOCHONDRION_ORGANIZATION	20	0.211	0.844	0.624498	0.8772371	1	503	tags=40%, lis
GOBP_PROTEIN_STABILIZATION	38	0.166	0.843	0.6647174	0.8773597	1	369	tags=21%, lis
HP_CONGENITAL_ONSET	44	0.163	0.843	0.7032755	0.8771023	1	691	tags=45%, lis
HP_NEURODEGENERATION	17	0.220	0.843	0.6378987	0.8766701	1	1649	tags=100%, l
GOCC_PIGMENT_GRANULE	24	0.192	0.842	0.6673307	0.87743413	1	280	tags=21%, lis
GOBP_AUTOPHAGY_OF_MITOCHONDRION	15	0.223	0.841	0.64444447	0.8772927	1	474	tags=40%, lis
HP_MEMORY_IMPAIRMENT	16	0.226	0.840	0.6529774	0.87893534	1	500	tags=44%, lis
HP_APHASIA	15	0.226	0.840	0.65553236	0.8786774	1	79	tags=13%, lis
GOCC_TRANS_GOLGI_NETWORK	49	0.155	0.839	0.6805293	0.87856656	1	21	tags=6%, list-
HP_ELEVATED_HEPATIC_TRANSAMINASE	32	0.173	0.839	0.67184466	0.8777411	1	153	tags=16%, lis
GOBP_REGULATION_OF_CELL_SHAPE	36	0.171	0.839	0.6487524	0.87686425	1	387	tags=31%, lis
GOMF_RNA_BINDING	308	0.103	0.839	0.8054054	0.8761724	1	673	tags=37%, lis
GOCC_AZUROPHIL_GRANULE_MEMBRANE	24	0.192	0.838	0.64257026	0.8776296	1	961	tags=67%, lis
GOBP_REGULATION_OF_ACTIN_FILAMENT_ORGANIZATION	55	0.149	0.838	0.694332	0.87698764	1	689	tags=45%, lis
HP_PEDIATRIC_ONSET	97	0.130	0.837	0.7413793	0.87730205	1	364	tags=22%, lis
GOBP_ORGANELLE_LOCALIZATION	88	0.132	0.833	0.72952384	0.8840715	1	552	tags=33%, lis
GOBP_REGULATION_OF_PROTEIN_CONTAINING_COMPLEX_DISASSEMBLY	23	0.197	0.831	0.69138277	0.88554966	1	123	tags=13%, lis
HP_ABNORMAL_B_CELL_MORPHOLOGY	16	0.222	0.831	0.6623656	0.8847275	1	155	tags=19%, lis
HP_TELANGIECTASIA	21	0.200	0.831	0.6851064	0.8840253	1	830	tags=57%, lis
GOBP_INTRACELLULAR_TRANSPORT	290	0.105	0.830	0.841637	0.88553834	1	563	tags=30%, lis
GOBP_POSITIVE_REGULATION_OF_PROTEOLYSIS_INVOLVED_IN_CELLULAR_PROTEIN_CATABOLIC_P	31	0.171	0.827	0.6646943	0.88931155	1	851	tags=58%, lis
GOBP_CELLULAR_RESPONSE_TO_HEAT	24	0.197	0.826	0.65900385	0.89095974	1	618	tags=42%, lis
REACTOME_COPII_MEDIATED_VESICLE_TRANSPORT	17	0.212	0.824	0.6826347	0.8932262	1	1342	tags=88%, lis
HP_ABNORMAL_LOCATION_OF_EARS	100	0.126	0.824	0.749522	0.8931839	1	385	tags=20%, lis
HP_DEVELOPMENTAL_REGRESSION	40	0.160	0.821	0.70408165	0.89657575	1	1155	tags=75%, lis
GOBP_CELLULAR_RESPONSE_TO_STARVATION	29	0.183	0.820	0.68846154	0.897147	1	420	tags=31%, lis
GOBP_HEPATICOBILIARY_SYSTEM_DEVELOPMENT	18	0.208	0.820	0.6686747	0.89768016	1	763	tags=56%, lis
HP_DEMENTIA	34	0.168	0.819	0.71428573	0.89758104	1	324	tags=21%, lis
GOMF_CHROMATIN_DNA_BINDING	24	0.189	0.818	0.6916836	0.8985883	1	278	tags=25%, lis
GOBP_PROTEIN_MODIFICATION_BY_SMALL_PROTEIN_CONJUGATION_OR_REMOVAL	206	0.108	0.817	0.7964912	0.89914095	1	283	tags=17%, lis
HP_RECURRENT_PNEUMONIA	18	0.213	0.816	0.6770833	0.8997001	1	587	tags=44%, lis
GOBP_REGULATION_OF_PEPTIDYL_TYROSINE_PHOSPHORYLATION	41	0.156	0.816	0.7029703	0.9002359	1	416	tags=24%, lis
GOBP_REGULATION_OF_MULTICELLULAR_ORGANISM_GROWTH	15	0.224	0.815	0.6918367	0.89977026	1	127	tags=13%, lis
HP_SLEEP_DISTURBANCE	70	0.137	0.815	0.7356322	0.89915097	1	330	tags=21%, lis
GOBP_EPITHELIAL_CELL_APOPTOTIC_PROCESS	18	0.206	0.815	0.67469877	0.8983968	1	1032	tags=72%, lis
HP_ABNORMAL_SOFT_PALATE_MORPHOLOGY	20	0.197	0.815	0.70871985	0.8980153	1	344	tags=25%, lis

HP_FUNCTIONAL_ABNORMALITY_OF_THE_GASTROINTESTINAL_TRACT	148	0.117	0.814	0.7842401	0.89815545	1	353	tags=20%, lis
GOBP_RNA_SPLICING_VIA_TRANSESTERIFICATION_REACTIONS	73	0.136	0.814	0.73308957	0.8973316	1	559	tags=32%, lis
HALLMARK_HEME_METABOLISM	34	0.166	0.814	0.69902915	0.8974473	1	1593	tags=94%, lis
GOBP_MONONUCLEAR_CELL_DIFFERENTIATION	70	0.137	0.811	0.77394634	0.9012549	1	397	tags=24%, lis
REACTOME_DDX58_IFIH1_MEDIATED_INDUCTION_OF_INTERFERON_ALPHA_BETA	23	0.188	0.808	0.6851852	0.9044226	1	374	tags=26%, lis
GOCC_GOLGI_APPARATUS_SUBCOMPARTMENT	154	0.113	0.808	0.8070501	0.9039357	1	40	tags=5%, list-
HP_ABNORMAL_MYELINATION	72	0.133	0.808	0.7448015	0.90329397	1	550	tags=32%, lis
HP_ABNORMAL_LARGE_INTESTINE_MORPHOLOGY	50	0.145	0.807	0.7318436	0.90337396	1	124	tags=10%, lis
GOBP_REGULATION_OF_LYMPHOCYTE_DIFFERENTIATION	29	0.172	0.807	0.6936759	0.9031862	1	334	tags=21%, lis
GOBP_REGULATION_OF_ORGANELLE_ASSEMBLY	24	0.179	0.806	0.7110656	0.90328723	1	326	tags=25%, lis
GOCC_ACTIN_FILAMENT	22	0.188	0.805	0.6843137	0.90525293	1	548	tags=36%, lis
GOBP_POSITIVE_REGULATION_OF_MAP_KINASE_ACTIVITY	35	0.166	0.804	0.7062257	0.90573525	1	127	tags=11%, lis
HP_ABNORMAL_VENTRICULAR_SEPTUM_MORPHOLOGY	58	0.143	0.803	0.7446808	0.9070943	1	862	tags=57%, lis
GOBP_REGULATION_OF_ACTIN_FILAMENT_BASED_PROCESS	71	0.135	0.802	0.7234042	0.9071922	1	549	tags=35%, lis
GOMF_ACTIN_FILAMENT_BINDING	34	0.160	0.802	0.71252567	0.9069151	1	417	tags=26%, lis
GOMF_NUCLEOSOME_BINDING	17	0.206	0.800	0.6814516	0.90800846	1	579	tags=41%, lis
GOBP_NEGATIVE_REGULATION_OF_HEMOPOIESIS	18	0.202	0.799	0.7055336	0.909085	1	74	tags=11%, lis
GOMF_PHOSPHATASE_REGULATOR_ACTIVITY	15	0.214	0.799	0.6818182	0.9087279	1	573	tags=40%, lis
HP_MALIGNANT_NEOPLASM_OF_THE_CENTRAL_NERVOUS_SYSTEM	18	0.209	0.799	0.69411767	0.907907	1	128	tags=17%, lis
HP_EPILEPTIC_SPASM	25	0.182	0.795	0.72141373	0.91358703	1	317	tags=24%, lis
HP_SKELETAL_MUSCLE_ATROPHY	54	0.143	0.793	0.74953616	0.91629815	1	263	tags=19%, lis
GOBP_ESTABLISHMENT_OF_PROTEIN_LOCALIZATION_TO_ORGANELLE	85	0.128	0.792	0.7844203	0.91599643	1	576	tags=33%, lis
GOCC_ADHERENS_JUNCTION	17	0.208	0.792	0.71457905	0.9151902	1	607	tags=47%, lis
GOBP_GLAND_DEVELOPMENT	46	0.150	0.792	0.76620823	0.915198	1	500	tags=30%, lis
KEGG_SPLICEOSOME	34	0.163	0.792	0.75	0.91451454	1	989	tags=65%, lis
REACTOME_RHO_GTPASE_EFFECTORS	47	0.153	0.791	0.7598425	0.9142853	1	832	tags=53%, lis
GOBP_REGULATION_OF_VIRAL_GENOME_REPLICATION	16	0.208	0.789	0.7302505	0.91712904	1	274	tags=25%, lis
REACTOME_P75_NTR_RECEPTOR_MEDIATED_SIGNALLING	17	0.208	0.789	0.72709554	0.91631657	1	628	tags=47%, lis
GOCC_PERIKARYON	15	0.213	0.788	0.7145749	0.9172578	1	546	tags=40%, lis
GOBP_DETECTION_OF_STIMULUS	21	0.191	0.787	0.7281746	0.91680896	1	445	tags=33%, lis
HP_NASAL_SPEECH	15	0.214	0.785	0.7014315	0.918975	1	442	tags=40%, lis
GOBP_NEGATIVE_REGULATION_OF_DNA_METABOLIC_PROCESS	18	0.206	0.785	0.7184874	0.91813725	1	150	tags=17%, lis
REACTOME_RAB_REGULATION_OF_TRAFFICKING	32	0.165	0.784	0.7509881	0.9201425	1	1543	tags=94%, lis
HP_CRYPTORCHIDISM	81	0.126	0.783	0.8292683	0.91951686	1	305	tags=15%, lis
HP_HYPERKERATOSIS	29	0.174	0.781	0.7411765	0.9224398	1	683	tags=45%, lis
HP_PROMINENT_NASAL_BRIDGE	22	0.186	0.779	0.7443763	0.9247044	1	358	tags=27%, lis
GOBP_POSITIVE_REGULATION_OF_MACROAUTOPHAGY	15	0.208	0.778	0.7197581	0.92452204	1	10	tags=7%, list-
HP_ABNORMAL_CONNECTION_OF_THE_CARDIAC_SEGMENTS	22	0.184	0.778	0.73214287	0.92410886	1	385	tags=32%, lis
HP_ABNORMALITY_OF_THE_HALLUX	22	0.184	0.776	0.7403101	0.9268069	1	299	tags=23%, lis
GOMF_MRNA_BINDING	55	0.136	0.770	0.80544746	0.93528086	1	376	tags=24%, lis
GOCC_RUFFLE_MEMBRANE	20	0.187	0.770	0.75482625	0.9344173	1	810	tags=55%, lis
GOBP_RIBOSOME_BIOGENESIS	28	0.171	0.770	0.75303644	0.9336026	1	656	tags=39%, lis
GOBP_POSITIVE_REGULATION_OF_T_CELL_PROLIFERATION	16	0.201	0.769	0.7153996	0.93408614	1	188	tags=19%, lis
HP_PROGRESSIVE_MICROCEPHALY	20	0.190	0.768	0.74757284	0.9340426	1	689	tags=45%, lis
GOBP_SPINDLE_ASSEMBLY	21	0.187	0.768	0.7423935	0.93347126	1	410	tags=29%, lis
REACTOME_MRNA_SPLICING	44	0.147	0.768	0.76226413	0.9329337	1	348	tags=20%, lis
HP_STATUS_EPILEPTICUS	23	0.177	0.767	0.7356322	0.9333533	1	208	tags=17%, lis
HP_MENTAL_DETERIORATION	57	0.135	0.767	0.8011472	0.9327969	1	337	tags=19%, lis
HP_DELAYED_ABILITY_TO_WALK	21	0.183	0.766	0.753507	0.9332156	1	780	tags=48%, lis
GOMF_ENZYME_INHIBITOR_ACTIVITY	42	0.147	0.765	0.7955912	0.9329521	1	117	tags=10%, lis
HP_UPPER_MOTOR_NEURON_DYSFUNCTION	168	0.106	0.764	0.87007874	0.9346096	1	374	tags=18%, lis
GOBP_INTEGRIN_MEDIATED_SIGNALING_PATHWAY	17	0.201	0.763	0.76953906	0.93414503	1	267	tags=24%, lis
PID_CXCR4_PATHWAY	28	0.170	0.763	0.78723407	0.93446416	1	500	tags=32%, lis
GOBP_INTRACELLULAR_RECEPTOR_SIGNALING_PATHWAY	43	0.148	0.762	0.7878788	0.9349048	1	396	tags=26%, lis
REACTOME_PI3K_AKT_SIGNALING_IN_CANCER	16	0.205	0.761	0.71686745	0.93535733	1	593	tags=44%, lis
GOBP_MONOSACCHARIDE_BIOSYNTHETIC_PROCESS	16	0.201	0.760	0.73939395	0.9356058	1	225	tags=19%, lis
GOMF_CALCIUM_ION_BINDING	65	0.131	0.759	0.79657793	0.93577796	1	256	tags=15%, lis
GOCC_CLATHRIN_COATED_VESICLE	33	0.159	0.758	0.799591	0.9362383	1	161	tags=12%, lis
HP_ANORECTAL_ANOMALY	47	0.144	0.758	0.79611653	0.93616563	1	385	tags=23%, lis
GOBP_REGULATION_OF_LEUKOCYTE_APOPTOTIC_PROCESS	16	0.206	0.757	0.76171875	0.93719447	1	494	tags=31%, lis
HP_DOWNSLANTED_PALPEBRAL_FISSURES	66	0.130	0.756	0.8241107	0.93669975	1	79	tags=5%, list-
GOMF_ACTIVE_TRANSMEMBRANE_TRANSPORTER_ACTIVITY	19	0.189	0.756	0.7590822	0.93673503	1	248	tags=21%, lis
HP_RECURRENT_FEVER	18	0.190	0.753	0.743295	0.9389395	1	575	tags=44%, lis
HP_POSTNATAL_MICROCEPHALY	30	0.162	0.751	0.79770994	0.941527	1	715	tags=47%, lis
HP_ABNORMAL_EXTERNAL_GENITALIA	124	0.110	0.750	0.88095236	0.94164455	1	561	tags=28%, lis
GOBP_MICROTUBULE_CYTOSKELETON_ORGANIZATION_INVOLVED_IN_MITOSIS	24	0.172	0.750	0.75384617	0.9416211	1	410	tags=29%, lis
GOBP_CELLULAR_RESPONSE_TO_ABIOTIC_STIMULUS	49	0.138	0.747	0.8203593	0.94460875	1	411	tags=27%, lis
GOBP_ACTIVATION_OF_MAPK_ACTIVITY	27	0.167	0.745	0.795501	0.94666666	1	127	tags=11%, lis
HP_ABNORMAL_INTESTINE_MORPHOLOGY	111	0.114	0.745	0.8665413	0.945971	1	368	tags=20%, lis
HP_ABNORMAL_ENZYME_COENZYME_ACTIVITY	59	0.133	0.744	0.8091603	0.9457665	1	397	tags=24%, lis
HP_DIMINISHED_MOTIVATION	19	0.183	0.742	0.75793654	0.9476313	1	324	tags=21%, lis
GOBP_RNA_LOCALIZATION	54	0.131	0.740	0.83914727	0.95003504	1	661	tags=37%, lis
HP_ABNORMAL_T_CELL_MORPHOLOGY	15	0.200	0.739	0.7757937	0.9508405	1	548	tags=40%, lis
GOBP_REGULATION_OF_MONONUCLEAR_CELL_MIGRATION	20	0.183	0.738	0.7612245	0.9508641	1	83	tags=10%, lis
HP_ABNORMAL_AUTONOMIC_NERVOUS_SYSTEM_PHYSIOLOGY	19	0.181	0.738	0.78541666	0.95006484	1	585	tags=37%, lis
GOMF_PROTEIN_SERINE_THREONINE_PHOSPHATASE_ACTIVITY	16	0.193	0.736	0.7799607	0.95111483	1	69	tags=13%, lis
HP_ABNORMAL_CARDIAC_SEPTUM_MORPHOLOGY	77	0.123	0.736	0.88291746	0.95046145	1	862	tags=55%, lis
HP_MALABSORPTION	33	0.152	0.736	0.790099	0.9496107	1	633	tags=39%, lis
HP_RIGIDITY	31	0.158	0.734	0.8180039	0.9517439	1	374	tags=26%, lis

GOBP_CELLULAR_RESPONSE_TO_EXTRACELLULAR_STIMULUS	42	0.142	0.734	0.81782943	0.95156443	1	420	tags=26%, lis
GOBP_POSITIVE_REGULATION_OF_NF_KAPPAB_TRANSCRIPTION_FACTOR_ACTIVITY	30	0.158	0.733	0.8011696	0.9512759	1	460	tags=30%, lis
GOBP_MRNA_3_END_PROCESSING	28	0.160	0.732	0.8199234	0.95157766	1	701	tags=46%, lis
HP_ENCEPHALOPATHY	36	0.147	0.730	0.8023483	0.9532096	1	337	tags=22%, lis
GOBP_REGULATION_OF_PROTEIN_POLYMERIZATION	44	0.140	0.730	0.83653843	0.9531273	1	549	tags=36%, lis
GOBP_VESICLE_MEDIATED_TRANSPORT_IN_SYNAPSE	17	0.188	0.729	0.7928994	0.9529295	1	1717	tags=100%, l
HP_MOTOR_DELAY	85	0.118	0.729	0.8650647	0.9522487	1	364	tags=20%, lis
HP_ABNORMAL_SOCIAL_BEHAVIOR	27	0.160	0.728	0.79837066	0.953286	1	484	tags=33%, lis
HP_INFANTILE_ONSET	75	0.120	0.723	0.8542435	0.95753795	1	414	tags=24%, lis
HP_ABNORMALITY_OF_COORDINATION	129	0.106	0.723	0.92120075	0.9568099	1	543	tags=28%, lis
GOBP_SULFUR_COMPOUND_BIOSYNTHETIC_PROCESS	24	0.172	0.723	0.7941176	0.9560754	1	40	tags=8%, list-
GOBP_RETROGRADE_VESICLE_MEDIATED_TRANSPORT_GOLGI_TO_ENDOPLASMIC_RETICULUM	19	0.183	0.723	0.824	0.9555588	1	707	tags=47%, lis
HALLMARK_APICAL_JUNCTION	23	0.165	0.722	0.81287724	0.95644075	1	635	tags=43%, lis
HP_ABNORMAL_MUSCLE_FIBER_MORPHOLOGY	17	0.189	0.721	0.76199615	0.95686203	1	649	tags=41%, lis
GOBP_REGULATION_OF_EXOCYTOSIS	25	0.165	0.720	0.8165681	0.95672923	1	318	tags=24%, lis
GOBP_IMMUNOGLOBULIN_PRODUCTION	20	0.177	0.720	0.7948207	0.9559573	1	198	tags=15%, lis
GOBP_ORGANOPHOSPHATE_METABOLIC_PROCESS	144	0.104	0.720	0.92038834	0.9552976	1	624	tags=35%, lis
HP_ABNORMAL_SIZE_OF_THE_PALPEBRAL_FISSURES	38	0.144	0.718	0.8380414	0.95615816	1	812	tags=50%, lis
HP_SHORT_ATTENTION_SPAN	64	0.126	0.718	0.8352713	0.95535696	1	580	tags=33%, lis
GOBP_ESTABLISHMENT_OF_PROTEIN_LOCALIZATION	317	0.089	0.718	0.94538605	0.9553497	1	534	tags=27%, lis
GOBP_DNA_BIOSYNTHETIC_PROCESS	27	0.162	0.717	0.8422091	0.9556993	1	452	tags=30%, lis
HP_MORTALITY_AGING	24	0.167	0.716	0.78571427	0.95623934	1	339	tags=25%, lis
GOBP_MEMBRANE_DOCKING	24	0.163	0.714	0.8268839	0.9573526	1	683	tags=46%, lis
HP_CEREBRAL_CORTICAL_ATROPHY	49	0.132	0.713	0.85742575	0.9582558	1	690	tags=43%, lis
GOBP_SENSORY_PERCEPTION	25	0.163	0.712	0.8248472	0.958585	1	520	tags=32%, lis
KEGG_B_CELL_RECEPTOR_SIGNALING_PATHWAY	31	0.154	0.711	0.80914515	0.95844203	1	593	tags=39%, lis
GOCC_MITOTIC_SPINDLE	22	0.174	0.710	0.8267223	0.95878047	1	1558	tags=95%, lis
GOBP_NCRNA_PROCESSING	36	0.142	0.710	0.8424242	0.95808333	1	559	tags=36%, lis
HP_HYPERTONIA	145	0.101	0.710	0.91468257	0.95729136	1	566	tags=28%, lis
HP_ABNORMAL_SYSTEMIC_ARTERIAL_MORPHOLOGY	57	0.125	0.710	0.8762089	0.95679253	1	385	tags=25%, lis
HP_DRY_SKIN	15	0.191	0.709	0.8280922	0.9564648	1	1492	tags=93%, lis
GOBP_GLYCOSYL_COMPOUND_METABOLIC_PROCESS	16	0.185	0.709	0.82542694	0.9562846	1	1722	tags=100%, l
HP_APLASIA_HYPOPLASIA_INVOLVING_BONES_OF_THE_THORAX	17	0.186	0.708	0.807393	0.95626587	1	550	tags=35%, lis
HP_HYPERACTIVITY	72	0.120	0.706	0.8967495	0.95791596	1	368	tags=22%, lis
GOCC_INTRINSIC_COMPONENT_OF_ENDOPLASMIC_RETICULUM_MEMBRANE	36	0.142	0.704	0.83966243	0.9589691	1	19	tags=6%, list-
GOBP_REGULATION_OF_RNA_SPLICING	37	0.141	0.703	0.8464567	0.95985055	1	348	tags=24%, lis
GOCC_INTRINSIC_COMPONENT_OF_ORGANELLE_MEMBRANE	62	0.122	0.702	0.8664047	0.9600939	1	377	tags=19%, lis
REACTOME_VESICLE_MEDIATED_TRANSPORT	138	0.102	0.701	0.9189189	0.9604418	1	632	tags=32%, lis
GOCC_MIDBODY	37	0.140	0.699	0.84501845	0.96136284	1	255	tags=16%, lis
HP_ABNORMAL_NASAL_BRIDGE_MORPHOLOGY	110	0.108	0.699	0.9222222	0.96064544	1	368	tags=17%, lis
GOCC_SPECIFIC_GRANULE_MEMBRANE	23	0.164	0.699	0.8373016	0.9598275	1	416	tags=26%, lis
GOBP_RESPONSE_TO_LIGHT_STIMULUS	40	0.141	0.697	0.82738096	0.96072894	1	206	tags=15%, lis
GOCC_NUCLEOLUS	144	0.101	0.697	0.94466406	0.9604301	1	692	tags=38%, lis
GOCC_NUCLEAR_PERIPHERY	21	0.169	0.696	0.83832335	0.9604935	1	197	tags=14%, lis
HP_RESPIRATORY_FAILURE	21	0.169	0.695	0.82077396	0.9607891	1	1608	tags=95%, lis
HP_ABNORMALITY_OF_THE_BREAST	54	0.127	0.694	0.8754864	0.9609215	1	128	tags=11%, lis
HP_HYPERMELANOTIC_MACULE	16	0.183	0.691	0.83585316	0.9637904	1	79	tags=13%, lis
GOBP_ANATOMICAL_STRUCTURE_HOMEOSTASIS	53	0.125	0.690	0.8649706	0.96376234	1	127	tags=11%, lis
GOBP_POSITIVE_REGULATION_OF_UBIQUITIN_DEPENDENT_PROTEIN_CATABOLIC_PROCESS	26	0.155	0.689	0.8413002	0.96369356	1	532	tags=35%, lis
GOMF_STEROID_BINDING	15	0.186	0.688	0.80824745	0.9641097	1	686	tags=47%, lis
GOBP_VESICLE_LOCALIZATION	34	0.143	0.687	0.8707865	0.9646355	1	1813	tags=100%, l
GOBP_NEGATIVE_REGULATION_OF_APOPTOTIC_SIGNALING_PATHWAY	40	0.135	0.687	0.8619247	0.9638658	1	326	tags=18%, lis
GOBP_IRON_ION_TRANSPORT	18	0.176	0.687	0.83501005	0.96312827	1	60	tags=11%, lis
GOCC_CELL_CORTEX	44	0.132	0.686	0.8707224	0.9628119	1	190	tags=14%, lis
HP_BILATERAL_TONIC_CLONIC_SEIZURE	29	0.149	0.686	0.86007464	0.9623284	1	672	tags=41%, lis
GOBP_POSITIVE_REGULATION_OF_PROTEASOMAL_UBIQUITIN_DEPENDENT_PROTEIN_CATABOLIC_	26	0.155	0.685	0.8800813	0.9622484	1	532	tags=35%, lis
HP_COLOBOMA	22	0.163	0.683	0.84381336	0.963492	1	1005	tags=73%, lis
GOBP_NEGATIVE_REGULATION_OF_DEPHOSPHORYLATION	19	0.170	0.680	0.85360825	0.9663489	1	503	tags=37%, lis
GOBP_POSITIVE_REGULATION_OF_MITOTIC_CELL_CYCLE	16	0.184	0.679	0.8582996	0.9662059	1	1524	tags=94%, lis
GOBP_NEGATIVE_REGULATION_OF_IMMUNE_EFFECTOR_PROCESS	21	0.164	0.678	0.82851636	0.9663144	1	349	tags=24%, lis
HP_ABNORMAL_INVOLUNTARY_EYE_MOVEMENTS	110	0.103	0.677	0.9323843	0.96685654	1	499	tags=27%, lis
GOCC_CELL_SUBSTRATE_JUNCTION	81	0.109	0.677	0.9219331	0.96609604	1	689	tags=40%, lis
GOBP_CARBOHYDRATE_BIOSYNTHETIC_PROCESS	32	0.144	0.675	0.8692153	0.9674973	1	722	tags=41%, lis
REACTOME_CLATHRIN_MEDIATED_ENDOCYTOSIS	28	0.149	0.674	0.85328186	0.96786994	1	437	tags=29%, lis
REACTOME_METABOLISM_OF_CARBOHYDRATES	36	0.137	0.672	0.88469183	0.968138	1	373	tags=22%, lis
GOBP_MITOTIC_SPINDLE_ORGANIZATION	21	0.162	0.670	0.86262625	0.969366	1	410	tags=29%, lis
GOMF_HELICASE_ACTIVITY	25	0.148	0.669	0.8574468	0.96980506	1	559	tags=36%, lis
GOBP_ESTABLISHMENT_OF_ORGANELLE_LOCALIZATION	65	0.114	0.669	0.89279115	0.9689969	1	1732	tags=95%, lis
GOMF_RNA_POLYMERASE_BINDING	18	0.168	0.669	0.8745098	0.9685789	1	708	tags=50%, lis
GOCC_CYTOPLASMIC_STRESS_GRANULE	20	0.160	0.668	0.8559499	0.96825767	1	476	tags=30%, lis
HP_APLASIA_HYPOPLASIA_OF_THE_CEREBELLAR_VERMIS	28	0.147	0.665	0.8562874	0.97042847	1	173	tags=14%, lis
HP_PHOTOPHOBIA	19	0.167	0.664	0.86519116	0.9701507	1	499	tags=32%, lis
HP_ABNORMAL_TESTIS_MORPHOLOGY	101	0.104	0.664	0.93726236	0.969947	1	689	tags=36%, lis
GOBP_TRANSPORT_ALONG_MICROTUBULE	21	0.158	0.659	0.8674948	0.9740338	1	552	tags=33%, lis
REACTOME_CELL_CELL_COMMUNICATION	17	0.165	0.656	0.8654618	0.9760588	1	690	tags=47%, lis
HP_ABNORMAL_VISUAL_ELECTROPHYSIOLOGY	21	0.158	0.655	0.8782435	0.9760332	1	520	tags=38%, lis
GOCC_LEADING_EDGE_MEMBRANE	30	0.138	0.651	0.89694655	0.9786549	1	732	tags=47%, lis
HP_SPECIFIC_LEARNING_DISABILITY	31	0.138	0.650	0.88846153	0.97836405	1	466	tags=29%, lis
GOBP_CYTOSKELETON_DEPENDENT_INTRACELLULAR_TRANSPORT	28	0.144	0.650	0.85	0.9776313	1	552	tags=32%, lis

HP_IMPAIRMENT_OF_ACTIVITIES_OF_DAILY_LIVING	29	0.142	0.649	0.8888889	0.9775505	1	284	tags=17%, lis
REACTOME_POST_TRANSLATIONAL_PROTEIN_MODIFICATION	234	0.084	0.649	0.98542804	0.9772869	1	284	tags=15%, lis
HP_GAIT_ATAXIA	24	0.153	0.648	0.9004237	0.97726995	1	1450	tags=88%, lis
GOBP_REGULATION_OF_DEPHOSPHORYLATION	34	0.135	0.646	0.888454	0.9776568	1	410	tags=26%, lis
GOMF_PHOSPHATIDYLINOSITOL_PHOSPHATE_BINDING	26	0.149	0.645	0.8637317	0.97772753	1	1799	tags=100%, l
GOCC_PHAGOCYTIC_VESICLE	40	0.129	0.644	0.8963532	0.9778332	1	213	tags=13%, lis
HP_IRIS_COLOBOMA	15	0.175	0.644	0.8636364	0.9773934	1	978	tags=73%, lis
GOBP_RESPONSE_TO_ESTRADIOL	19	0.160	0.643	0.87474746	0.977499	1	471	tags=32%, lis
GOMF_ENZYME_BINDING	337	0.079	0.641	0.99122804	0.97835743	1	425	tags=20%, lis
HP_ABNORMALITY_OF_THE_SMALL_INTESTINE	35	0.135	0.641	0.88533837	0.9778206	1	763	tags=46%, lis
GOBP_REGULATION_OF_AUTOPHAGY	68	0.109	0.641	0.92870545	0.97714293	1	69	tags=6%, list-
GOBP_T_CELL_MIGRATION	15	0.177	0.638	0.88622755	0.978449	1	732	tags=47%, lis
HP_ABNORMALITY_OF_THE_CEREBRAL_CORTEX	52	0.118	0.637	0.92115384	0.9784817	1	301	tags=17%, lis
GOBP_PROTEIN_LOCALIZATION_TO_ENDOPLASMIC_RETICULUM	17	0.162	0.637	0.890625	0.97798485	1	336	tags=24%, lis
GOBP_REGULATION_OF_APOPTOTIC_SIGNALING_PATHWAY	71	0.107	0.636	0.95057034	0.9778983	1	440	tags=23%, lis
GOMF_SINGLE_STRANDED_RNA_BINDING	19	0.158	0.634	0.86519116	0.9783521	1	374	tags=26%, lis
GOMF_UBIQUITIN_LIKE_PROTEIN_BINDING	18	0.163	0.632	0.85862786	0.97907674	1	1414	tags=89%, lis
GOBP_VACUOLAR_TRANSPORT	34	0.128	0.632	0.8956159	0.97869176	1	549	tags=32%, lis
HP_ICHTHYOSIS	19	0.156	0.631	0.88128775	0.9783374	1	683	tags=42%, lis
HP_VISUAL_FIELD_DEFECT	16	0.168	0.629	0.8770492	0.97943354	1	500	tags=38%, lis
HALLMARK_PI3K_AKT_MTOR_SIGNALING	22	0.147	0.628	0.8888889	0.9796941	1	674	tags=41%, lis
HP_LOWER_EXTREMITY_JOINT_DISLOCATION	34	0.129	0.624	0.9249531	0.98198974	1	374	tags=24%, lis
GOBP_REGULATION_OF_NIK_NF_KAPPAB_SIGNALING	20	0.158	0.622	0.9048583	0.98205864	1	187	tags=15%, lis
GOBP_REGULATION_OF_INTRACELLULAR_TRANSPORT	67	0.105	0.621	0.9364486	0.9821161	1	548	tags=28%, lis
GOBP_MRNA_TRANSPORT	38	0.125	0.618	0.9292365	0.9836896	1	661	tags=39%, lis
GOBP_ESTABLISHMENT_OF_RNA_LOCALIZATION	47	0.118	0.618	0.9462572	0.9832623	1	499	tags=28%, lis
GOBP_REGULATION_OF_PROTEIN_DEPOLYMERIZATION	15	0.172	0.617	0.9087221	0.98299265	1	640	tags=40%, lis
HP_RENAL_CYST	20	0.149	0.615	0.91386557	0.9838004	1	284	tags=20%, lis
HP_AGGRESSIVE_BEHAVIOR	25	0.145	0.612	0.92815536	0.9848836	1	487	tags=28%, lis
GOBP_CELLULAR_RESPONSE_TO_HYDROGEN_PEROXIDE	18	0.158	0.611	0.9148936	0.9845096	1	255	tags=17%, lis
HP_ABNORMAL_ATRIOVENTRICULAR_VALVE_MORPHOLOGY	19	0.150	0.611	0.9070707	0.9840423	1	841	tags=58%, lis
REACTOME_RAB_GEFS_EXCHANGE_GTP_FOR_GDP_ON_RABS	23	0.140	0.610	0.913556	0.98400867	1	1312	tags=83%, lis
GOBP_POSITIVE_REGULATION_OF_AUTOPHAGY	25	0.140	0.608	0.9282787	0.98408866	1	10	tags=4%, list-
REACTOME_MEMBRANE_TRAFFICKING	128	0.088	0.608	0.9653846	0.9834165	1	632	tags=33%, lis
REACTOME_TOLL_LIKE_RECEPTOR_TLR1_TLR2_CASCADE	29	0.130	0.606	0.9287212	0.9844592	1	344	tags=21%, lis
HP_HYPOPIGMENTATION_OF_THE_SKIN	36	0.123	0.605	0.932914	0.9839683	1	358	tags=22%, lis
GOBP_COPII_COATED_VESICLE_BUDDING	18	0.155	0.604	0.9027778	0.98400813	1	1454	tags=89%, lis
GOBP_REGULATION_OF_ALPHA_BETA_T_CELL_ACTIVATION	19	0.147	0.603	0.93110645	0.98389375	1	118	tags=11%, lis
GOBP_POSITIVE_REGULATION_OF_INTRACELLULAR_TRANSPORT	46	0.117	0.601	0.94476193	0.9844019	1	548	tags=33%, lis
GOCC_HISTONE_DEACETYLASE_COMPLEX	18	0.157	0.599	0.90263695	0.98508215	1	385	tags=28%, lis
GOBP_VESICLE_BUDDING_FROM_MEMBRANE	22	0.138	0.599	0.93700784	0.9842942	1	1822	tags=100%, l
HP_CEREBELLAR_CYST	15	0.161	0.599	0.93542075	0.983527	1	152	tags=13%, lis
GOCC_TRANSPORT_VESICLE	60	0.106	0.597	0.9527559	0.98386824	1	697	tags=37%, lis
HP_BRAIN_ATROPHY	121	0.087	0.596	0.97709924	0.9840338	1	691	tags=36%, lis
GOBP_PROTEIN_POLYMERIZATION	60	0.102	0.595	0.9491525	0.98367906	1	424	tags=25%, lis
GOBP_MONONUCLEAR_CELL_MIGRATION	34	0.121	0.592	0.9432485	0.9846906	1	116	tags=9%, list-
HP_VOMITING	24	0.138	0.587	0.93503934	0.9868652	1	138	tags=13%, lis
HP_ABNORMAL_PYRAMIDAL_SIGN	54	0.104	0.583	0.9467456	0.98825794	1	716	tags=39%, lis
HP_ACTION_TREMOR	17	0.151	0.583	0.91322315	0.98776406	1	500	tags=29%, lis
REACTOME_RECRUITMENT_OF_NUMA_TO_MITOTIC_CENTROSOMES	15	0.161	0.583	0.9367816	0.9870704	1	503	tags=33%, lis
GOBP_REGULATION_OF_MACROAUTOPHAGY	38	0.117	0.581	0.9358717	0.9874605	1	60	tags=5%, list-
GOBP_KERATINOCYTE_DIFFERENTIATION	15	0.160	0.580	0.9402391	0.98713183	1	23	tags=7%, list-
GOBP_RESPONSE_TO_STEROID_HORMONE	36	0.121	0.579	0.9506903	0.98657095	1	396	tags=22%, lis
HP_BROAD_FINGER	15	0.159	0.572	0.9268775	0.9896667	1	374	tags=27%, lis
GOBP_LYMPHOCYTE_MIGRATION	20	0.143	0.568	0.9244186	0.99138397	1	413	tags=25%, lis
REACTOME_ION_CHANNEL_TRANSPORT	26	0.129	0.565	0.9433594	0.99185526	1	580	tags=35%, lis
GOBP_MICROTUBULE_BASED_TRANSPORT	22	0.137	0.565	0.937751	0.99133986	1	552	tags=32%, lis
HP_ATROPHY_DEGENERATION_AFFECTING_THE_CENTRAL_NERVOUS_SYSTEM	135	0.080	0.559	1	0.99335176	1	374	tags=19%, lis
HP_NEOPLASM_OF_THE_LIVER	18	0.144	0.559	0.93970895	0.9928313	1	368	tags=22%, lis
GOCC_LAMELLIPODIUM	43	0.108	0.556	0.9496982	0.99319065	1	123	tags=9%, list-
GOMF_PROTEIN_MACROMOLECULE_ADAPTOR_ACTIVITY	42	0.110	0.556	0.97540987	0.9925584	1	437	tags=24%, lis
HP_OSTEOPOROSIS	23	0.128	0.555	0.9472693	0.9922431	1	374	tags=22%, lis
GOCC_HETEROCHROMATIN	15	0.148	0.554	0.9576923	0.99202085	1	869	tags=60%, lis
HP_DILATED_FOURTH_VENTRICLE	16	0.144	0.553	0.95075756	0.9919394	1	152	tags=13%, lis
HP_ATTENTION_DEFICIT_HYPERACTIVITY_DISORDER	47	0.102	0.552	0.9555985	0.9911999	1	368	tags=21%, lis
HP_DYSARTHRIA	57	0.098	0.551	0.96672827	0.99086756	1	566	tags=30%, lis
HP_ABNORMALITY_OF_THE_OVARY	22	0.133	0.550	0.94285715	0.99084026	1	231	tags=14%, lis
GOBP_RNA_EXPORT_FROM_NUCLEUS	35	0.113	0.549	0.96138996	0.99045885	1	661	tags=37%, lis
PID_MTOR_4PATHWAY	16	0.143	0.547	0.95824635	0.99068075	1	1105	tags=75%, lis
GOBP_REGULATION_OF_LEUKOCYTE_MEDIATED_IMMUNITY	48	0.100	0.546	0.96484375	0.99025214	1	349	tags=17%, lis
HP_DEVIATION_OF_TOES	22	0.130	0.544	0.9518072	0.9901156	1	98	tags=9%, list-
GOBP_POSITIVE_REGULATION_OF_IMMUNE_EFFECTOR_PROCESS	46	0.105	0.543	0.95284873	0.98976094	1	257	tags=13%, lis
GOMF_SINGLE_STRANDED_DNA_BINDING	19	0.136	0.538	0.95051545	0.9913131	1	238	tags=16%, lis
HP_BABINSKI_SIGN	38	0.104	0.535	0.96463656	0.99177593	1	165	tags=11%, lis
HP_HIRSUTISM	26	0.119	0.533	0.9596929	0.99170524	1	691	tags=42%, lis
GOBP_POSITIVE_REGULATION_OF_JNK_CASCADE	18	0.137	0.531	0.9498998	0.9917247	1	998	tags=67%, lis
HP_LOW_POSTERIOR_HAIRLINE	19	0.135	0.531	0.958498	0.99105716	1	104	tags=11%, lis
GOBP_REGULATION_OF_TRANSLATIONAL_INITIATION	16	0.138	0.530	0.9664032	0.99042565	1	1241	tags=81%, lis
GOCC_ENDOPLASMIC_RETICULUM_GOLGI_INTERMEDIATE_COMPARTMENT_MEMBRANE	20	0.131	0.524	0.96070725	0.9920888	1	1676	tags=95%, lis

GOBP_ACTIN_CYTOSKELETON_REORGANIZATION	16	0.138	0.518	0.976	0.99330336	1	371	tags=25%, lis
HP_BLEPHAROPHIMOSIS	21	0.125	0.516	0.9533074	0.9931644	1	1848	tags=100%, l
GOBP_POSITIVE_REGULATION_OF_EPITHELIAL_CELL_MIGRATION	30	0.111	0.514	0.98221344	0.9931597	1	536	tags=30%, lis
GOBP_FATTY_ACID_METABOLIC_PROCESS	38	0.103	0.506	0.97276264	0.9947775	1	904	tags=55%, lis
HP_URINARY_INCONTINENCE	22	0.118	0.505	0.9801193	0.9943599	1	19	tags=5%, list=
REACTOME_PROCESSING_OF_CAPPED_INTRON_CONTAINING_PRE_MRNA	54	0.092	0.504	0.9879518	0.99370885	1	143	tags=9%, list=
GOBP_PLACENTA_DEVELOPMENT	18	0.125	0.494	0.9782609	0.9957749	1	1482	tags=89%, lis
GOCC_SPINDLE	54	0.088	0.493	0.9830189	0.99534774	1	327	tags=17%, lis
GOBP_VASCULAR_ENDOTHELIAL_GROWTH_FACTOR_RECEPTOR_SIGNALING_PATHWAY	23	0.114	0.490	0.98582995	0.99530554	1	500	tags=30%, lis
GOBP_TRANSCRIPTION_ELONGATION_FROM_RNA_POLYMERASE_II_PROMOTER	16	0.132	0.489	0.98245615	0.9949217	1	856	tags=56%, lis
HP_HYPERMETROPIA	31	0.107	0.489	0.99029124	0.99418545	1	561	tags=32%, lis
GOCC_GOLGI_ASSOCIATED_VESICLE	24	0.113	0.488	0.98125	0.99362195	1	1544	tags=92%, lis
GOBP_TRANSCRIPTION_INITIATION_FROM_RNA_POLYMERASE_II_PROMOTER	23	0.115	0.486	0.98945147	0.99316525	1	668	tags=39%, lis
REACTOME_ER_TO_GOLGI_ANTEROGRADE_TRANSPORT	31	0.103	0.475	0.9823183	0.99492216	1	1676	tags=94%, lis
GOCC_FILOPODIUM	19	0.116	0.474	0.99021524	0.9943637	1	382	tags=21%, lis
REACTOME_TRANSLOCATION_OF_SLC2A4_GLUT4_TO_THE_PLASMA_MEMBRANE	19	0.115	0.473	0.9878049	0.9938869	1	803	tags=47%, lis
GOMF_MOLECULAR_ADAPTOR_ACTIVITY	51	0.086	0.470	0.9904215	0.99370056	1	707	tags=37%, lis
HP_ABNORMAL_NIPPLE_MORPHOLOGY	32	0.097	0.459	0.99224806	0.9950177	1	358	tags=19%, lis
HP_ENLARGED_POSTERIOR_FOSSA	18	0.116	0.446	0.9900398	0.99630696	1	152	tags=11%, lis
GOBP_DEVELOPMENTAL_MATURATION	29	0.096	0.444	0.9941061	0.9958179	1	460	tags=24%, lis
GOCC_NEURON_TO_NEURON_SYNAPSE	28	0.098	0.431	0.9980843	0.9966889	1	236	tags=14%, lis
GOMF_MODIFICATION_DEPENDENT_PROTEIN_BINDING	31	0.090	0.424	1	0.99658835	1	152	tags=10%, lis
HP_WIDE_INTERMAMILLARY_DISTANCE	17	0.098	0.363	1	0.999297	1	794	tags=47%, lis
