# Supplementary material for: Biodegradable nanoparticles induce cGAS/STING-dependent reprogramming of myeloid cells to promote tumor immunotherapy
Source: Front Immunol. 2022 Aug 18;13:887649. doi: 10.3389/fimmu.2022.887649 (PMC9433741; doi:10.3389/fimmu.2022.887649)
Supplement: Supplementary file 12 [file Table_6.pdf]

Supplemental Table 7. Signaling Pathway Analysis for Monocytes - 3 Doses Once Every 3 Days - ONP-302 vs. Saline

NAME	SIZE	ES	NES	NOM p-val	FDR q-val	FWER p-val	RANK AT MA	LEADING ED(
HALLMARK_INTERFERON_ALPHA_RESPONSE	28	0.570	2.672	0	0.003563387	0.003	239	tags=50%, lis
HALLMARK_INTERFERON_GAMMA_RESPONSE	52	0.449	2.509	0	0.007638263	0.013	311	tags=44%, lis
GOBP_DEFENSE_RESPONSE_TO_BACTERIUM	27	0.547	2.487	0	0.007806502	0.02	193	tags=44%, lis
GOBP_ANTIGEN_PROCESSING_AND_PRESENTATION_OF_EXOGENOUS_PEPTIDE_ANTIGEN_VIA_MH	24	0.552	2.462	0	0.006736316	0.023	319	tags=63%, lis
REACTOME_ANTIGEN_PROCESSING_CROSS_PRESENTATION	28	0.526	2.411	0	0.009888973	0.042	401	tags=68%, lis
REACTOME_HEDGEHOG_LIGAND_BIOGENESIS	18	0.598	2.370	0	0.012678439	0.065	361	tags=78%, lis
GOCC_PEPTIDASE_COMPLEX	22	0.527	2.325	0	0.016794458	0.096	295	tags=59%, lis
REACTOME_NEGATIVE_REGULATION_OF_NOTCH4_SIGNALING	17	0.579	2.272	0.002283105	0.02167795	0.138	361	tags=71%, lis
REACTOME_SCF_SKP2_MEDIATED_DEGRADATION_OF_P27_P21	17	0.570	2.270	0	0.019786073	0.142	361	tags=71%, lis
REACTOME_AUF1_HNRNP_D0_BINDS_AND_DESTABILIZES_MRNA	16	0.579	2.260	0.002610966	0.019943912	0.157	361	tags=75%, lis
REACTOME_DEGRADATION_OF_DVL	17	0.559	2.258	0	0.018236302	0.157	361	tags=71%, lis
HALLMARK_OXIDATIVE_PHOSPHORYLATION	40	0.440	2.254	0	0.017914025	0.165	431	tags=58%, lis
GOBP_ANTIGEN_PROCESSING_AND_PRESENTATION_OF_PEPTIDE_ANTIGEN_VIA_MHC_CLASS_I	27	0.479	2.249	0	0.017261056	0.171	319	tags=56%, lis
REACTOME_REGULATION_OF_RUNX3_EXPRESSION_AND_ACTIVITY	19	0.550	2.241	0.002450980	0.01796719	0.192	361	tags=63%, lis
GOCC_ENDOPEPTIDASE_COMPLEX	17	0.562	2.232	0	0.017880408	0.203	295	tags=65%, lis
REACTOME_DNA_REPLICATION_PRE_INITIATION	16	0.579	2.227	0.002309468	0.01749682	0.211	361	tags=75%, lis
REACTOME_ASSEMBLY_OF_THE_PRE_REPLICATIVE_COMPLEX	16	0.579	2.203	0	0.02019728	0.247	361	tags=75%, lis
GOBP_OXIDATIVE_PHOSPHORYLATION	29	0.469	2.198	0	0.019856846	0.253	392	tags=59%, lis
REACTOME_FCERI_MEDIATED_NF_KB_ACTIVATION	23	0.491	2.197	0	0.018997734	0.255	361	tags=65%, lis
GOBP_TRANSLATIONAL_INITIATION	24	0.489	2.193	0	0.018521134	0.261	276	tags=54%, lis
REACTOME_G2_M_CHECKPOINTS	20	0.511	2.164	0.002444988	0.022597812	0.331	393	tags=70%, lis
REACTOME_REGULATION_OF_RAS_BY_GAPS	19	0.524	2.157	0	0.02232418	0.339	361	tags=63%, lis
GOBP_B_CELL_RECEPTOR_SIGNALING_PATHWAY	26	0.465	2.149	0.002380952	0.023206813	0.366	151	tags=31%, lis
REACTOME_THE_ROLE_OF_GTSE1_IN_G2_M_PROGRESSION_AFTER_G2_CHECKPOINT	18	0.522	2.137	0	0.024199469	0.394	361	tags=67%, lis
HALLMARK_ALLOGRAFT_REJECTION	51	0.384	2.123	0	0.025886418	0.43	201	tags=31%, lis
REACTOME_UCH_PROTEINASES	23	0.486	2.123	0	0.025025701	0.432	390	tags=65%, lis
GOCC_INNER_MITOCHONDRIAL_MEMBRANE_PROTEIN_COMPLEX	23	0.470	2.109	0	0.02724068	0.477	389	tags=57%, lis
REACTOME_TCF_DEPENDENT_SIGNALING_IN_RESPONSE_TO_WNT	30	0.436	2.109	0	0.026479032	0.479	371	tags=57%, lis
REACTOME_DEGRADATION_OF_AXIN	18	0.525	2.106	0.005063291	0.026133792	0.49	361	tags=67%, lis
REACTOME_TNFR2_NON_CANONICAL_NF_KB_PATHWAY	19	0.518	2.099	0.002645502	0.027177082	0.519	361	tags=68%, lis
REACTOME_ORC1_REMOVAL_FROM_CHROMATIN	18	0.518	2.096	0	0.02701921	0.529	361	tags=67%, lis
REACTOME_REGULATION_OF_EXPRESSION_OF_SLITS_AND_ROBOS	19	0.504	2.093	0	0.026471915	0.535	361	tags=63%, lis
KEGG_PARKINSONS_DISEASE	26	0.447	2.087	0	0.026849998	0.551	385	tags=54%, lis
REACTOME_DECTIN_1_MEDIATED_NONCANONICAL_NF_KB_SIGNALING	17	0.535	2.080	0.002364066	0.028413838	0.584	361	tags=71%, lis
GOBP_INNATE_IMMUNE_RESPONSE	125	0.298	2.072	0	0.029773155	0.614	319	tags=34%, lis
REACTOME_PCP_CE_PATHWAY	21	0.502	2.066	0.002463054	0.030325089	0.628	361	tags=67%, lis
REACTOME_DEFECTIVE_CFTR_CAUSES_CYSTIC_FIBROSIS	18	0.509	2.064	0.002415459	0.030017234	0.633	361	tags=72%, lis
REACTOME_RESPIRATORY_ELECTRON_TRANSPORT_ATP_SYNTHESIS_BY_CHEMIOSMOTIC_COUPLIN	27	0.449	2.059	0.002610966	0.030534372	0.648	389	tags=56%, lis
REACTOME_HOST_INTERACTIONS_OF_HIV_FACTORS	28	0.446	2.058	0.00486618	0.030263783	0.653	461	tags=68%, lis
KEGG_OXIDATIVE_PHOSPHORYLATION	32	0.413	2.056	0.004962779	0.030127468	0.667	389	tags=53%, lis
REACTOME_STABILIZATION_OF_P53	18	0.508	2.051	0.002341920	0.030519327	0.681	361	tags=67%, lis
GOBP_ACTIVATION_OF_INNATE_IMMUNE_RESPONSE	30	0.429	2.046	0.002551020	0.03112697	0.699	319	tags=50%, lis
REACTOME_ASYMMETRIC_LOCALIZATION_OF_PCP_PROTEINS	18	0.499	2.037	0.002525252	0.03284129	0.725	361	tags=67%, lis
REACTOME_ABC_TRANSPORTER_DISORDERS	19	0.480	2.035	0.006896551	0.032707676	0.732	361	tags=68%, lis
KEGG_PROTEASOME	15	0.537	2.034	0.011848342	0.03232508	0.737	295	tags=60%, lis
REACTOME_MITOTIC_G1_PHASE_AND_G1_S_TRANSITION	22	0.456	2.032	0	0.03185201	0.741	361	tags=59%, lis
REACTOME_CYCLIN_A_CDK2_ASSOCIATED_EVENTS_AT_S_PHASE_ENTRY	19	0.511	2.026	0	0.032602057	0.755	361	tags=63%, lis
REACTOME_DEGRADATION_OF_GLI1_BY_THE_PROTEASOME	19	0.491	2.013	0	0.034685284	0.79	361	tags=63%, lis
GOBP_ANTIGEN_PROCESSING_AND_PRESENTATION_OF_PEPTIDE_ANTIGEN	45	0.372	2.013	0	0.034316268	0.793	319	tags=47%, lis
REACTOME_G1_S_DNA_DAMAGE_CHECKPOINTS	19	0.485	2.000	0.004938272	0.036760118	0.825	361	tags=63%, lis
REACTOME_REGULATION_OF_PTEN_STABILITY_AND_ACTIVITY	19	0.489	1.994	0	0.037850488	0.835	361	tags=63%, lis
REACTOME_RUNX1_REGULATES_TRANSCRIPTION_OF_GENES_INVOLVED_IN_DIFFERENTIATION_OF	20	0.486	1.990	0	0.038054626	0.842	361	tags=60%, lis
GOBP_ANTIGEN_PROCESSING_AND_PRESENTATION	52	0.354	1.986	0	0.038364362	0.847	319	tags=44%, lis
REACTOME_REGULATION_OF_RUNX2_EXPRESSION_AND_ACTIVITY	22	0.454	1.970	0.009876544	0.04176168	0.882	361	tags=59%, lis
REACTOME_CYTOPROTECTION_BY_HMOX1	32	0.401	1.968	0	0.041454118	0.884	361	tags=50%, lis
REACTOME_HEDGEHOG_ON_STATE	22	0.462	1.965	0.002427184	0.041790135	0.891	361	tags=59%, lis
GOBP_POSITIVE_REGULATION_OF_DEFENSE_RESPONSE	54	0.342	1.963	0.005063291	0.04160549	0.892	319	tags=39%, lis
REACTOME_APOPTOSIS	39	0.385	1.960	0.005025125	0.041927088	0.903	407	tags=59%, lis
REACTOME_DEGRADATION_OF_BETA_CATENIN_BY_THE_DESTRUCTION_COMPLEX	25	0.440	1.955	0.004975124	0.042901903	0.911	361	tags=60%, lis
GOBP_POSITIVE_REGULATION_OF_RESPONSE_TO_BIOTIC_STIMULUS	39	0.382	1.953	0	0.04281901	0.916	319	tags=44%, lis
REACTOME_SIGNALING_BY_HEDGEHOG	27	0.422	1.952	0.009411764	0.04246994	0.919	361	tags=56%, lis
GOBP_DEFENSE_RESPONSE_TO_OTHER_ORGANISM	145	0.279	1.947	0	0.043057565	0.93	319	tags=32%, lis
GOBP_TUMOR_NECROSIS_FACTOR_MEDIATED_SIGNALING_PATHWAY	27	0.431	1.945	0	0.0432797	0.935	295	tags=52%, lis
REACTOME_THE_CITRIC_ACID_TCA_CYCLE_AND_RESPIRATORY_ELECTRON_TRANSPORT	33	0.387	1.938	0.002538071	0.04462731	0.942	389	tags=48%, lis
REACTOME_SIGNALING_BY_NOTCH4	23	0.448	1.935	0.005037783	0.044759672	0.944	364	tags=61%, lis
REACTOME_CELLULAR_RESPONSE_TO_HYPOXIA	21	0.454	1.925	0.010256411	0.04761866	0.95	361	tags=57%, lis
GOBP_ESTABLISHMENT_OF_TISSUE_POLARITY	20	0.457	1.924	0.007444169	0.047152925	0.951	432	tags=65%, lis
REACTOME_CELLULAR_RESPONSE_TO_CHEMICAL_STRESS	41	0.373	1.924	0	0.046599433	0.951	361	tags=46%, lis
REACTOME_MAPK6_MAPK4_SIGNALING	23	0.431	1.918	0.004926108	0.048019215	0.956	361	tags=57%, lis
GOBP_POSITIVE_REGULATION_OF_RESPONSE_TO_EXTERNAL_STIMULUS	77	0.315	1.903	0	0.05284581	0.969	319	tags=38%, lis
GOBP_HUMORAL_IMMUNE_RESPONSE	25	0.427	1.901	0.002710027	0.052878667	0.97	318	tags=48%, lis
KEGG_ANTIGEN_PROCESSING_AND_PRESENTATION	16	0.494	1.897	0.007058823	0.05386673	0.972	170	tags=44%, lis
GOBP_REGULATION_OF_CELLULAR_AMINO_ACID_METABOLIC_PROCESS	17	0.478	1.886	0.002506265	0.057505544	0.981	295	tags=53%, lis
GOBP_RESPONSE_TO_INTERFERON_GAMMA	31	0.394	1.885	0.007444169	0.056793466	0.982	318	tags=39%, lis
GOBP_POSITIVE_REGULATION_OF_CANONICAL_WNT_SIGNALING_PATHWAY	27	0.418	1.880	0.002518891	0.058373533	0.983	296	tags=48%, lis
GOBP_ANTIGEN_RECEPTOR_MEDIATED_SIGNALING_PATHWAY	60	0.330	1.878	0	0.058676057	0.984	151	tags=25%, lis

REACTOME_HEDGEHOG_OFF_STATE	21	0.444	1.874	0.014251782	0.059157033	0.985	361	tags=57%, lis
GOBP_REGULATION_OF_MRNA_CATABOLIC_PROCESS	50	0.344	1.864	0.002624672	0.06244481	0.99	361	tags=46%, lis
GOBP_INTERFERON_GAMMA_MEDIATED_SIGNALING_PATHWAY	20	0.447	1.848	0.009456265	0.068778776	0.995	197	tags=35%, lis
GOBP_SCF_DEPENDENT_PROTEASOMAL_UBIQUITIN_DEPENDENT_PROTEIN_CATABOLIC_PROCESS	17	0.472	1.846	0.015228426	0.06898345	0.996	295	tags=53%, lis
REACTOME_SIGNALING_BY_ROBO_RECEPTORS	28	0.398	1.844	0.009661836	0.06890824	0.996	361	tags=57%, lis
GOBP_REGULATION_OF_HEMATOPOIETIC_STEM_CELL_DIFFERENTIATION	20	0.444	1.830	0.00952381	0.075171255	0.998	295	tags=50%, lis
REACTOME_ABC_FAMILY_PROTEINS_MEDIATED_TRANSPORT	22	0.412	1.822	0.007142857	0.07805748	0.998	361	tags=64%, lis
GOBP_ADAPTIVE_IMMUNE_RESPONSE	68	0.302	1.820	0.008287293	0.07796844	0.998	180	tags=24%, lis
GOBP_MEMBRANE_INVAGINATION	19	0.439	1.820	0.01627907	0.077220194	0.998	312	tags=47%, lis
GOBP_NEGATIVE_REGULATION_OF_CELL_CYCLE_G2_M_PHASE_TRANSITION	24	0.405	1.810	0.004761905	0.08153962	0.998	295	tags=46%, lis
GOMF_PEPTIDASE_REGULATOR_ACTIVITY	18	0.446	1.801	0.011961723	0.08609068	0.999	281	tags=56%, lis
GOBP_B_CELL_MEDIATED_IMMUNITY	18	0.455	1.799	0.014851485	0.08607857	0.999	319	tags=50%, lis
REACTOME_REGULATION_OF_MRNA_STABILITY_BY_PROTEINS_THAT_BIND_AU_RICH_ELEMENTS	24	0.411	1.794	0.009324009	0.08791721	0.999	361	tags=54%, lis
GOCC_VESICLE_LUMEN	63	0.302	1.787	0	0.09140117	1	287	tags=35%, lis
GOBP_REGULATION_OF_CELLULAR_AMINE_METABOLIC_PROCESS	19	0.431	1.783	0.011547344	0.09328789	1	295	tags=47%, lis
GOBP_ACTIVATION_OF_IMMUNE_RESPONSE	96	0.275	1.779	0.002747252	0.09443386	1	319	tags=33%, lis
GOBP_RESPONSE_TO_INTERLEUKIN_1	40	0.349	1.778	0.002487562	0.09383903	1	295	tags=38%, lis
REACTOME_TRANSCRIPTIONAL_REGULATION_BY_RUNX3	23	0.411	1.776	0.013667426	0.09394957	1	361	tags=52%, lis
GOBP_MORPHOGENESIS_OF_A_POLARIZED_EPITHELIUM	24	0.386	1.771	0.007425742	0.09625899	1	432	tags=58%, lis
REACTOME_METABOLISM_OF_POLYAMINES	17	0.456	1.766	0.013123359	0.098081976	1	295	tags=53%, lis
GOBP_NEUTROPHIL_CHEMOTAXIS	19	0.435	1.758	0.015189873	0.10153142	1	39	tags=21%, lis
GOBP_NEUTROPHIL_MIGRATION	20	0.424	1.755	0.01754386	0.102537595	1	39	tags=20%, lis
GOBP_POSITIVE_REGULATION_OF_WNT_SIGNALING_PATHWAY	31	0.361	1.753	0.02387268	0.10281382	1	296	tags=42%, lis
REACTOME_BETA_CATENIN_INDEPENDENT_WNT_SIGNALING	28	0.363	1.746	0.010416667	0.10654195	1	387	tags=54%, lis
GOBP_REGULATION_OF_TRANSCRIPTION_FROM_RNA_POLYMERASE_II_PROMOTER_IN_RESPONSE	22	0.409	1.745	0.025062656	0.10572249	1	361	tags=55%, lis
GOBP_REGULATION_OF_ANIMAL_ORGAN_MORPHOGENESIS	21	0.413	1.742	0.01010101	0.10718877	1	306	tags=48%, lis
REACTOME_APC_C_CDH1_MEDIATED_DEGRADATION_OF_CDC20_AND_OTHER_APC_C_CDH1_TAR	19	0.424	1.740	0.017811704	0.10736667	1	361	tags=63%, lis
GOBP_ANAPHASE_PROMOTING_COMPLEX_DEPENDENT_CATABOLIC_PROCESS	19	0.424	1.738	0.020833334	0.10728801	1	361	tags=63%, lis
GOBP_REGULATION_OF_TRANSLATIONAL_INITIATION	16	0.440	1.736	0.016949153	0.10801819	1	276	tags=50%, lis
REACTOME_SIGNALING_BY_WNT	44	0.329	1.734	0.01305483	0.107811965	1	387	tags=48%, lis
GOBP_MITOCHONDRIAL_TRANSMEMBRANE_TRANSPORT	21	0.408	1.728	0.012048192	0.11095362	1	198	tags=33%, lis
GOCC_VACUOLAR_LUMEN	34	0.343	1.723	0.012406948	0.11325954	1	287	tags=44%, lis
GOCC_MITOCHONDRIAL_PROTEIN_CONTAINING_COMPLEX	35	0.344	1.719	0.01511335	0.11511292	1	389	tags=49%, lis
GOMF_PROTON_TRANSMEMBRANE_TRANSPORTER_ACTIVITY	23	0.393	1.718	0.015228426	0.11499142	1	109	tags=26%, lis
GOBP_REGULATION_OF_HEMATOPOIETIC_PROGENITOR_CELL_DIFFERENTIATION	21	0.404	1.717	0.022675738	0.114415444	1	295	tags=48%, lis
REACTOME_REGULATION_OF_HMOX1_EXPRESSION_AND_ACTIVITY	21	0.403	1.681	0.02962963	0.14142922	1	361	tags=57%, lis
REACTOME_DISORDERS_OF_TRANSMEMBRANE_TRANSPORTERS	23	0.379	1.669	0.035264485	0.15121587	1	361	tags=57%, lis
GOBP_MITOCHONDRIAL_MEMBRANE_ORGANIZATION	24	0.370	1.661	0.019704433	0.15615264	1	393	tags=58%, lis
REACTOME_APC_C_MEDIATED_DEGRADATION_OF_CELL_CYCLE_PROTEINS	20	0.395	1.660	0.027210884	0.15653376	1	361	tags=60%, lis
GOBP_IMMUNE_RESPONSE_REGULATING_SIGNALING_PATHWAY	95	0.253	1.654	0.002754821	0.16081569	1	180	tags=21%, lis
GOBP_HEMATOPOIETIC_STEM_CELL_DIFFERENTIATION	22	0.394	1.653	0.023136247	0.16104311	1	295	tags=45%, lis
GOBP_REGULATION_OF_CELL_CYCLE_G2_M_PHASE_TRANSITION	36	0.332	1.651	0.025252525	0.16086504	1	295	tags=42%, lis
GOBP_DEFENSE_RESPONSE	202	0.219	1.647	0.003058104	0.16350676	1	319	tags=28%, lis
GOBP_REGULATION_OF_RESPONSE_TO_CYTOKINE_STIMULUS	35	0.330	1.644	0.024038462	0.16443682	1	197	tags=29%, lis
GOBP_REGULATION_OF_INNATE_IMMUNE_RESPONSE	57	0.289	1.641	0.011173184	0.16637506	1	319	tags=37%, lis
GOBP_GRANULOCYTE_CHEMOTAXIS	22	0.390	1.640	0.028503563	0.16621496	1	39	tags=18%, lis
REACTOME_SWITCHING_OF_ORIGINS_TO_A_POST_REPLICATIVE_STATE	21	0.384	1.627	0.04187192	0.17735243	1	361	tags=57%, lis
GOBP_RESPONSE_TO_BACTERIUM	73	0.269	1.625	0.011527377	0.1778225	1	281	tags=30%, lis
GOBP_RESPONSE_TO_TUMOR_NECROSIS_FACTOR	41	0.314	1.623	0.017156864	0.17844167	1	295	tags=37%, lis
GOBP_CELLULAR_AMINO_ACID_METABOLIC_PROCESS	26	0.358	1.620	0.034739453	0.18025497	1	295	tags=42%, lis
GOBP_REGULATION_OF_DEFENSE_RESPONSE	93	0.251	1.615	0.002688172	0.18321857	1	319	tags=31%, lis
GOBP_GRANULOCYTE_MIGRATION	25	0.362	1.612	0.025943397	0.18521608	1	39	tags=16%, lis
GOBP_POSITIVE_REGULATION_OF_IMMUNE_RESPONSE	117	0.236	1.611	0.007978723	0.18501477	1	319	tags=29%, lis
REACTOME_DEUBIQUITINATION	41	0.303	1.603	0.019753087	0.19166861	1	361	tags=44%, lis
REACTOME_PROGRAMMED_CELL_DEATH	46	0.294	1.601	0.024154589	0.19240537	1	407	tags=50%, lis
GOCC_FICOLIN_1_RICH_GRANULE_LUMEN	32	0.328	1.601	0.04534005	0.19109225	1	231	tags=31%, lis
REACTOME_UB_SPECIFIC_PROCESSING_PROTEASES	29	0.333	1.599	0.034031413	0.19184595	1	361	tags=48%, lis
GOBP_REGULATION_OF_RESPONSE_TO_BIOTIC_STIMULUS	69	0.265	1.598	0.021634616	0.19122532	1	319	tags=35%, lis
REACTOME_TRANSCRIPTIONAL_REGULATION_BY_RUNX2	26	0.356	1.594	0.032407407	0.1940472	1	361	tags=50%, lis
GOBP_RESPONSE_TO_BIOTIC_STIMULUS	195	0.212	1.592	0.005797101	0.19454353	1	204	tags=20%, lis
GOBP_CIRCADIAN_RHYTHM	20	0.388	1.589	0.035	0.1960118	1	679	tags=85%, lis
REACTOME_CLASS_I_MHC_MEDIATED_ANTIGEN_PROCESSING_PRESENTATION	60	0.278	1.589	0.022099448	0.19498461	1	304	tags=35%, lis
GOBP_REGULATION_OF_RESPONSE_TO_EXTERNAL_STIMULUS	124	0.232	1.586	0.018229166	0.1965816	1	319	tags=31%, lis
GOBP_POSITIVE_REGULATION_OF_TRANSLATION	17	0.406	1.583	0.04118993	0.19922797	1	297	tags=53%, lis
REACTOME_SIGNALING_BY_THE_B_CELL_RECEPTOR_BCR	37	0.313	1.580	0.03883495	0.20092203	1	295	tags=35%, lis
REACTOME_METABOLISM_OF_AMINO_ACIDS_AND_DERIVATIVES	26	0.343	1.575	0.034912717	0.20535125	1	295	tags=42%, lis
GOBP_RESPONSE_TO_INORGANIC_SUBSTANCE	70	0.260	1.572	0.012755102	0.20691015	1	231	tags=24%, lis
HALLMARK_APOPTOSIS	33	0.318	1.572	0.038647342	0.20549858	1	229	tags=30%, lis
REACTOME_TRANSCRIPTIONAL_REGULATION_BY_RUNX1	36	0.316	1.567	0.037037037	0.20996225	1	295	tags=36%, lis
GOCC_ACTIN_FILAMENT	16	0.405	1.562	0.057279237	0.21396424	1	337	tags=50%, lis
GOBP_REGULATION_OF_BINDING	58	0.269	1.560	0.015345269	0.21504286	1	211	tags=26%, lis
GOBP_POSTTRANSCRIPTIONAL_REGULATION_OF_GENE_EXPRESSION	105	0.237	1.557	0.018372703	0.21655992	1	299	tags=32%, lis
GOMF_ION_CHANNEL_BINDING	15	0.419	1.553	0.055813953	0.21915889	1	393	tags=67%, lis
GOCC_EXTERNAL_SIDE_OF_PLASMA_MEMBRANE	41	0.300	1.552	0.041666668	0.21881989	1	151	tags=22%, lis
REACTOME_ADAPTIVE_IMMUNE_SYSTEM	138	0.224	1.552	0.010989011	0.21825895	1	241	tags=24%, lis
REACTOME_TCR_SIGNALING	30	0.329	1.542	0.049140047	0.2286258	1	361	tags=50%, lis
KEGG_HUNTINGTONS_DISEASE	34	0.307	1.540	0.032258064	0.22912224	1	109	tags=21%, lis
GOMF_ENDOPEPTIDASE_ACTIVITY	42	0.301	1.540	0.038560413	0.22771785	1	284	tags=36%, lis
GOMF_TRANSLATION_REGULATOR_ACTIVITY	23	0.358	1.538	0.0548926	0.22843535	1	866	tags=91%, lis

REACTOME_DNA_REPLICATION	22	0.361	1.536	0.044554457	0.23021123	1	361	tags=55%, lis
GOBP_MRNA_METABOLIC_PROCESS	148	0.218	1.535	0.011594203	0.22943611	1	299	tags=30%, lis
GOBP_NON_CANONICAL_WNT_SIGNALING_PATHWAY	26	0.344	1.530	0.059382424	0.2337082	1	387	tags=50%, lis
GOBP_LYMPHOCYTE_MEDIATED_IMMUNITY	37	0.300	1.528	0.035897437	0.23589016	1	319	tags=35%, lis
GOCC_PROTON_TRANSPORTING_TWO_SECTOR_ATPASE_COMPLEX	17	0.379	1.525	0.066176474	0.23760949	1	76	tags=24%, lis
GOBP_PEPTIDE_BIOSYNTHETIC_PROCESS	79	0.244	1.525	0.020100502	0.23636927	1	299	tags=33%, lis
GOBP_RESPONSE_TO_CYTOKINE	174	0.210	1.522	0.008174387	0.23935139	1	370	tags=32%, lis
GOCC_LYSOSOMAL_LUMEN	15	0.402	1.511	0.052896727	0.2513079	1	126	tags=33%, lis
GOMF_PEPTIDASE_ACTIVITY	65	0.255	1.507	0.036649216	0.25583005	1	284	tags=32%, lis
REACTOME_INTERLEUKIN_1_SIGNALING	27	0.327	1.503	0.046035804	0.25888842	1	361	tags=48%, lis
REACTOME_DOWNSTREAM_SIGNALING_EVENTS_OF_B_CELL_RECEPTOR_BCR	26	0.329	1.499	0.06	0.26325378	1	361	tags=46%, lis
GOBP_AMIDE_BIOSYNTHETIC_PROCESS	89	0.238	1.491	0.030303031	0.27264768	1	299	tags=33%, lis
GOMF_TRANSLATION_REGULATOR_ACTIVITY_NUCLEIC_ACID_BINDING	20	0.355	1.490	0.06741573	0.2722486	1	520	tags=65%, lis
GOBP_REGULATION_OF_B_CELL_ACTIVATION	28	0.323	1.483	0.06419753	0.28155163	1	186	tags=25%, lis
GOMF_SINGLE_STRANDED_DNA_BINDING	16	0.381	1.480	0.072864324	0.2829929	1	178	tags=31%, lis
GOBP_CELL_CYCLE_G2_M_PHASE_TRANSITION	42	0.282	1.480	0.042105265	0.2815461	1	295	tags=38%, lis
REACTOME_MITOTIC_G2_G2_M_PHASES	33	0.307	1.480	0.08076009	0.28022552	1	295	tags=39%, lis
GOBP_RNA_CATABOLIC_PROCESS	69	0.248	1.480	0.038004752	0.27928588	1	299	tags=32%, lis
GOBP_INNATE_IMMUNE_RESPONSE_ACTIVATING_SIGNAL_TRANSDUCTION	22	0.348	1.478	0.07524272	0.2800317	1	361	tags=50%, lis
GOBP_INTERLEUKIN_1_MEDIATED_SIGNALING_PATHWAY	26	0.328	1.478	0.058679707	0.27894753	1	361	tags=50%, lis
REACTOME_SIGNALING_BY_NOTCH	36	0.283	1.475	0.0825	0.28110608	1	364	tags=44%, lis
REACTOME_INTERFERON_SIGNALING	32	0.300	1.473	0.07692308	0.28252518	1	398	tags=47%, lis
REACTOME_TP53_REGULATES_METABOLIC_GENES	16	0.387	1.470	0.088541664	0.28489125	1	314	tags=44%, lis
GOBP_ACTIN_POLYMERIZATION_OR_DEPOLYMERIZATION	37	0.291	1.469	0.048469387	0.2845786	1	400	tags=46%, lis
GOBP_NEGATIVE_REGULATION_OF_CANONICAL_WNT_SIGNALING_PATHWAY	27	0.315	1.468	0.061333332	0.28435183	1	295	tags=41%, lis
GOBP_CANONICAL_WNT_SIGNALING_PATHWAY	44	0.280	1.468	0.054320987	0.2836476	1	296	tags=36%, lis
GOCC_SIDE_OF_MEMBRANE	69	0.245	1.467	0.057142857	0.28281823	1	83	tags=14%, lis
GOBP_RESPIRATORY_ELECTRON_TRANSPORT_CHAIN	19	0.359	1.465	0.087677725	0.28422657	1	392	tags=47%, lis
GOBP_REGULATION_OF_PEPTIDASE_ACTIVITY	57	0.250	1.464	0.04134367	0.2840764	1	257	tags=26%, lis
GOCC_ORGANELLE_INNER_MEMBRANE	58	0.259	1.463	0.047222223	0.28334102	1	355	tags=34%, lis
GOBP_REGULATION_OF_STEM_CELL_DIFFERENTIATION	25	0.326	1.462	0.07848101	0.2838913	1	295	tags=40%, lis
GOBP_FC_EPSILON_RECEPTOR_SIGNALING_PATHWAY	34	0.292	1.459	0.0906801	0.28686574	1	319	tags=38%, lis
GOCC_RIBONUCLEOPROTEIN_COMPLEX	87	0.232	1.457	0.045454547	0.28770855	1	292	tags=30%, lis
GOMF_ELECTRON_TRANSFER_ACTIVITY	20	0.351	1.451	0.091566265	0.29596418	1	250	tags=35%, lis
GOBP_POST_TRANSLATIONAL_PROTEIN_MODIFICATION	50	0.258	1.450	0.04556962	0.2956119	1	295	tags=36%, lis
GOBP_ATP_SYNTHESIS_COUPLED_ELECTRON_TRANSPORT	19	0.359	1.450	0.101298705	0.29424375	1	392	tags=47%, lis
GOBP_POSITIVE_REGULATION_OF_IMMUNE_SYSTEM_PROCESS	163	0.204	1.449	0.028169014	0.29289633	1	151	tags=17%, lis
GOBP_RIBONUCLEOPROTEIN_COMPLEX_SUBUNIT_ORGANIZATION	34	0.292	1.441	0.06403941	0.30404648	1	290	tags=38%, lis
GOBP_PROTON_TRANSMEMBRANE_TRANSPORT	26	0.326	1.439	0.079254076	0.30477524	1	198	tags=27%, lis
GOBP_ELECTRON_TRANSPORT_CHAIN	25	0.314	1.436	0.079896905	0.3082627	1	392	tags=44%, lis
GOBP_POSITIVE_REGULATION_OF_B_CELL_ACTIVATION	16	0.372	1.429	0.090243906	0.3180587	1	151	tags=31%, lis
GOMF_ACTIN_FILAMENT_BINDING	19	0.336	1.426	0.09975669	0.32046387	1	978	tags=95%, lis
GOBP_RESPONSE_TO_TYPE_I_INTERFERON	16	0.375	1.425	0.101149425	0.32054773	1	398	tags=50%, lis
GOBP_PROTEIN_MODIFICATION_BY_SMALL_PROTEIN_REMOVAL	48	0.263	1.424	0.072916664	0.32052353	1	361	tags=42%, lis
GOBP_NEGATIVE_REGULATION_OF_IMMUNE_RESPONSE	24	0.323	1.419	0.0862069	0.32723346	1	160	tags=25%, lis
GOBP_ENDOCYTOSIS	90	0.222	1.415	0.033898305	0.33284023	1	214	tags=20%, lis
GOBP_POSITIVE_REGULATION_OF_APOPTOTIC_SIGNALING_PATHWAY	16	0.361	1.411	0.12412178	0.33723605	1	341	tags=50%, lis
REACTOME_S_PHASE	31	0.301	1.408	0.085574575	0.3403084	1	361	tags=45%, lis
REACTOME_FC_EPSILON_RECEPTOR_FCERI_SIGNALING	38	0.278	1.407	0.08974359	0.3410025	1	361	tags=42%, lis
GOBP_CELLULAR_OXIDANT_DETOXIFICATION	16	0.360	1.405	0.10941476	0.3435226	1	19	tags=13%, lis
GOBP_REGULATION_OF_IMMUNE_RESPONSE	169	0.197	1.404	0.046376813	0.34198073	1	180	tags=17%, lis
GOBP_REGULATION_OF_WNT_SIGNALING_PATHWAY	55	0.251	1.403	0.07526882	0.3424872	1	296	tags=33%, lis
REACTOME_SEPARATION_OF_SISTER_CHROMATIDS	37	0.276	1.398	0.072164945	0.34985462	1	295	tags=38%, lis
GOBP_MITOCHONDRIAL_TRANSPORT	41	0.267	1.393	0.073569484	0.35588774	1	393	tags=44%, lis
GOBP_RESPONSE_TO_HYDROGEN_PEROXIDE	26	0.308	1.393	0.09975669	0.35509092	1	307	tags=31%, lis
GOBP_REGULATION_OF_MRNA_METABOLIC_PROCESS	80	0.225	1.392	0.059850372	0.3542865	1	299	tags=33%, lis
GOBP_REGULATION_OF_LEUKOCYTE_CHEMOTAXIS	19	0.333	1.391	0.124087594	0.3539259	1	173	tags=32%, lis
REACTOME_CLEC7A_DECTIN_1_SIGNALING	28	0.293	1.390	0.11734694	0.35418856	1	361	tags=46%, lis
GOMF_OXIDOREDUCTASE_ACTIVITY	60	0.243	1.387	0.074742265	0.3582615	1	328	tags=30%, lis
GOBP_NEGATIVE_REGULATION_OF_PEPTIDASE_ACTIVITY	23	0.314	1.384	0.12195122	0.36135712	1	257	tags=30%, lis
GOBP_CELL_RECOGNITION	20	0.325	1.384	0.11002445	0.3598049	1	151	tags=25%, lis
GOCC_ENDOPLASMIC_RETICULUM_LUMEN	29	0.298	1.383	0.11084906	0.35892123	1	275	tags=41%, lis
GOCC_MITOCHONDRIAL_ENVELOPE	86	0.219	1.383	0.08101266	0.35757166	1	392	tags=37%, lis
GOBP_VIRAL_GENOME_REPLICATION	17	0.357	1.381	0.1097852	0.3592686	1	275	tags=35%, lis
REACTOME_ANTIGEN_PROCESSING_UBIQUITINATION_PROTEASOME_DEGRADATION	47	0.252	1.379	0.082706764	0.36183396	1	296	tags=36%, lis
REACTOME_METABOLISM_OF_RNA	99	0.211	1.378	0.057788946	0.36130902	1	295	tags=28%, lis
GOBP_INTERLEUKIN_1_PRODUCTION	17	0.346	1.372	0.1255814	0.3710182	1	444	tags=59%, lis
REACTOME_INTERLEUKIN_1_FAMILY_SIGNALING	32	0.289	1.371	0.1080402	0.36944935	1	407	tags=50%, lis
GOMF_TRANSLATION_FACTOR_ACTIVITY_RNA_BINDING	19	0.330	1.370	0.13425925	0.3710461	1	520	tags=63%, lis
GOBP_POSITIVE_REGULATION_OF_CELLULAR_AMIDE_METABOLIC_PROCESS	21	0.323	1.367	0.11335012	0.37404904	1	297	tags=43%, lis
GOBP_CELLULAR_RESPIRATION	27	0.301	1.364	0.1076555	0.3781643	1	414	tags=44%, lis
GOBP_NEGATIVE_REGULATION_OF_CELL_CYCLE_PHASE_TRANSITION	41	0.267	1.364	0.089108914	0.37692046	1	299	tags=32%, lis
GOBP_ATP_BIOSYNTHETIC_PROCESS	16	0.352	1.363	0.13443395	0.37639713	1	431	tags=56%, lis
GOCC_ENDOPLASMIC_RETICULUM_PROTEIN_CONTAINING_COMPLEX	29	0.294	1.360	0.11137441	0.3801836	1	355	tags=41%, lis
GOCC_RIBONUCLEOPROTEIN_GRANULE	46	0.252	1.358	0.11111111	0.3813064	1	491	tags=52%, lis
GOCC_SPLICEOSOMAL_COMPLEX	35	0.269	1.355	0.11922141	0.3846485	1	290	tags=37%, lis
GOBP_LEUKOCYTE_CHEMOTAXIS	38	0.264	1.352	0.12531969	0.38796693	1	44	tags=13%, lis
GOBP_RHYTHMIC_PROCESS	28	0.289	1.351	0.12868632	0.3894606	1	699	tags=79%, lis
GOBP_POSITIVE_REGULATION_OF_LEUKOCYTE_CHEMOTAXIS	17	0.340	1.351	0.1475827	0.38803467	1	138	tags=29%, lis

GOBP_GENERATION_OF_PRECURSOR_METABOLITES_AND_ENERGY	70	0.226	1.349	0.08309455	0.38883188	1	250	tags=26%, lis
GOCC_NUCLEAR_PERIPHERY	21	0.316	1.348	0.15107913	0.38951716	1	457	tags=57%, lis
GOBP_RECEPTOR_MEDIATED_ENDOCYTOSIS	44	0.255	1.348	0.12289157	0.38822654	1	119	tags=16%, lis
GOBP_RNA_SPLICING	89	0.211	1.347	0.0726817	0.38774073	1	294	tags=30%, lis
GOMF_GUANYL_NUCLEOTIDE_BINDING	45	0.250	1.346	0.11244019	0.3885339	1	110	tags=16%, lis
GOCC_ENDOCYTIC_VESICLE	50	0.239	1.339	0.12740384	0.40043885	1	39	tags=10%, lis
GOBP_CYTOKINE_MEDIATED_SIGNALING_PATHWAY	124	0.199	1.336	0.061827958	0.40299535	1	361	tags=32%, lis
GOMF_LIPID_BINDING	72	0.215	1.336	0.11488251	0.4023863	1	106	tags=15%, lis
GOCC_EARLY_ENDOSOME_MEMBRANE	19	0.322	1.333	0.13399504	0.40506193	1	115	tags=21%, lis
GOBP_CELLULAR_AMIDE_METABOLIC_PROCESS	122	0.194	1.332	0.07235142	0.40656075	1	304	tags=30%, lis
GOMF_CIS_REGULATORY_REGION_SEQUENCE_SPECIFIC_DNA_BINDING	83	0.213	1.330	0.1027027	0.40791127	1	178	tags=20%, lis
GOMF_PROTEIN_C_TERMINUS_BINDING	24	0.305	1.330	0.13221154	0.4063689	1	166	tags=25%, lis
GOBP_DETOXIFICATION	19	0.324	1.330	0.14527845	0.4053512	1	19	tags=11%, lis
GOBP_AMINE_METABOLIC_PROCESS	24	0.305	1.329	0.13975903	0.40446487	1	295	tags=38%, lis
GOBP_CELLULAR_RESPONSE_TO_TOXIC_SUBSTANCE	19	0.324	1.329	0.14150943	0.40344936	1	19	tags=11%, lis
GOBP_CELL_CHEMOTAXIS	45	0.249	1.328	0.10997442	0.4033327	1	44	tags=13%, lis
GOBP_RESPONSE_TO_UV	15	0.351	1.327	0.13592233	0.40390337	1	1067	tags=100%, l
REACTOME_RESPIRATORY_ELECTRON_TRANSPORT	19	0.318	1.326	0.13065326	0.40355742	1	314	tags=37%, lis
GOBP_REGULATION_OF_DNA_TEMPLATED_TRANSCRIPTION_IN_RESPONSE_TO_STRESS	31	0.277	1.324	0.14285715	0.40509948	1	361	tags=42%, lis
GOCC_AZUROPHIL_GRANULE_LUMEN	23	0.299	1.324	0.1383812	0.4039115	1	287	tags=43%, lis
GOBP_NEGATIVE_REGULATION_OF_IMMUNE_SYSTEM_PROCESS	65	0.226	1.321	0.11165048	0.40750206	1	202	tags=20%, lis
GOBP_NEGATIVE_REGULATION_OF_WNT_SIGNALING_PATHWAY	29	0.284	1.320	0.15075377	0.4081296	1	295	tags=38%, lis
GOBP_CELL_CELL_SIGNALING	132	0.189	1.319	0.07584269	0.40779757	1	241	tags=22%, lis
GOBP_ADAPTIVE_IMMUNE_RESPONSE_BASED_ON_SOMATIC_RECOMBINATION_OF_IMMUNE_RE	41	0.247	1.316	0.11405835	0.41192943	1	342	tags=32%, lis
GOBP_RIBONUCLEOSIDE_TRIPHOSPHATE_BIOSYNTHETIC_PROCESS	18	0.331	1.315	0.14177215	0.41235974	1	437	tags=56%, lis
KEGG_ALZHEIMERS_DISEASE	36	0.265	1.314	0.16129032	0.41306847	1	209	tags=25%, lis
GOBP_NEGATIVE_REGULATION_OF_LYMPHOCYTE_ACTIVATION	33	0.267	1.313	0.13012049	0.41281077	1	49	tags=12%, lis
GOBP_PROTEIN_POLYUBIQUITINATION	55	0.234	1.310	0.13333334	0.41800797	1	303	tags=33%, lis
REACTOME_INTERFERON_GAMMA_SIGNALING	19	0.319	1.309	0.14356436	0.417885	1	197	tags=26%, lis
GOBP_MYELOID_LEUKOCYTE_MIGRATION	39	0.253	1.307	0.1298077	0.42018282	1	44	tags=13%, lis
GOBP_REGULATION_OF_IMMUNE_SYSTEM_PROCESS	232	0.169	1.307	0.055072464	0.41921324	1	204	tags=18%, lis
GOBP_DNA_CONFORMATION_CHANGE	22	0.297	1.299	0.15228426	0.43214086	1	461	tags=55%, lis
REACTOME_HIV_INFECTION	44	0.240	1.297	0.11025641	0.43380886	1	361	tags=36%, lis
REACTOME_MHC_CLASS_II_ANTIGEN_PRESENTATION	21	0.301	1.297	0.17199017	0.43285573	1	180	tags=29%, lis
GOBP_NUCLEOSIDE_PHOSPHATE_BIOSYNTHETIC_PROCESS	31	0.267	1.289	0.14778325	0.44597563	1	77	tags=16%, lis
GOBP_NEGATIVE_REGULATION_OF_NUCLEOBASE_CONTAINING_COMPOUND_METABOLIC_PROCES	159	0.178	1.287	0.07672634	0.44890028	1	179	tags=18%, lis
GOCC_NUCLEAR_SPECK	73	0.212	1.285	0.13022113	0.45170772	1	310	tags=30%, lis
GOCC_MITOCHONDRIAL_MATRIX	32	0.271	1.281	0.16829269	0.45689994	1	217	tags=25%, lis
GOBP_REGULATION_OF_CELLULAR_KETONE_METABOLIC_PROCESS	27	0.279	1.279	0.1460396	0.4594431	1	295	tags=37%, lis
GOBP_NIK_NF_KAPPAB_SIGNALING	34	0.259	1.277	0.16707617	0.4614368	1	295	tags=35%, lis
GOMF_LIGASE_ACTIVITY	17	0.325	1.274	0.14442013	0.46558824	1	65	tags=18%, lis
GOBP_REGULATION_OF_MONONUCLEAR_CELL_MIGRATION	18	0.315	1.269	0.20383693	0.47549945	1	401	tags=56%, lis
GOBP_AEROBIC_RESPIRATION	17	0.317	1.263	0.19385342	0.48486456	1	231	tags=29%, lis
GOBP_LYMPHOCYTE_MIGRATION	18	0.316	1.260	0.19759037	0.48949683	1	369	tags=50%, lis
GOBP_REGULATION_OF_CYTOSKELETON_ORGANIZATION	75	0.209	1.257	0.15113351	0.49389097	1	211	tags=20%, lis
GOBP_REGULATION_OF_CELLULAR_AMIDE_METABOLIC_PROCESS	67	0.211	1.257	0.14690721	0.49373662	1	299	tags=31%, lis
GOBP_REGULATION_OF_CYSTEINE_TYPE_ENDOPEPTIDASE_ACTIVITY	38	0.241	1.256	0.16410257	0.49385557	1	42	tags=11%, lis
GOBP_REGULATION_OF_LYMPHOCYTE_ACTIVATION	73	0.209	1.252	0.13352273	0.4987446	1	71	tags=12%, lis
GOBP_PROTEIN_COMPLEX_OLIGOMERIZATION	19	0.297	1.250	0.18886198	0.50269216	1	320	tags=37%, lis
GOBP_REGULATION_OF_LEUKOCYTE_MIGRATION	33	0.255	1.241	0.1923077	0.51901835	1	202	tags=27%, lis
GOMF_IMMUNE_RECEPTOR_ACTIVITY	21	0.286	1.241	0.20140515	0.5178631	1	676	tags=76%, lis
GOBP_DIVALENT_INORGANIC_CATION_HOMEOSTASIS	38	0.243	1.239	0.18933333	0.519314	1	273	tags=29%, lis
GOCC_COLLAGEN_CONTAINING_EXTRACELLULAR_MATRIX	26	0.277	1.239	0.19385342	0.5190473	1	300	tags=35%, lis
REACTOME_TRANSLATION	29	0.264	1.237	0.19194312	0.5198331	1	284	tags=31%, lis
GOCC_PHAGOCYTIC_VESICLE_MEMBRANE	21	0.296	1.235	0.18527316	0.522391	1	110	tags=19%, lis
GOCC_EXTERNAL_ENCAPSULATING_STRUCTURE	27	0.269	1.234	0.21447721	0.5227944	1	300	tags=33%, lis
GOBP_CELL_CELL_SIGNALING_BY_WNT	73	0.205	1.234	0.16957606	0.52232486	1	234	tags=23%, lis
REACTOME_C_TYPE_LECTIN_RECEPTORS_CLRS	32	0.257	1.232	0.19700748	0.5239578	1	361	tags=44%, lis
GOCC_CELL_LEADING_EDGE	71	0.203	1.232	0.16666667	0.52387923	1	353	tags=34%, lis
GOBP_LEUKOCYTE_PROLIFERATION	46	0.229	1.231	0.17759563	0.52252465	1	47	tags=11%, lis
GOBP_CELL_SURFACE_RECEPTOR_SIGNALING_PATHWAY_INVOLVED_IN_CELL_CELL_SIGNALING	76	0.203	1.231	0.16944444	0.5215969	1	306	tags=29%, lis
GOBP_T_CELL_RECEPTOR_SIGNALING_PATHWAY	42	0.230	1.230	0.18579236	0.5210508	1	318	tags=33%, lis
GOBP_NUCLEOSIDE_TRIPHOSPHATE_BIOSYNTHETIC_PROCESS	19	0.306	1.228	0.23201856	0.5242428	1	437	tags=53%, lis
GOBP_NEGATIVE_REGULATION_OF_DEFENSE_RESPONSE	23	0.280	1.228	0.20822622	0.52295107	1	160	tags=26%, lis
GOBP_FC_RECEPTOR_SIGNALING_PATHWAY	56	0.214	1.227	0.17005076	0.52275705	1	337	tags=32%, lis
GOBP_REGULATION_OF_ACTIN_FILAMENT_BASED_PROCESS	58	0.214	1.227	0.18717949	0.52114505	1	211	tags=21%, lis
GOCC_LAMELLIPODIUM	37	0.240	1.226	0.21303259	0.52180517	1	337	tags=38%, lis
GOBP_ATP_METABOLIC_PROCESS	50	0.223	1.225	0.18258427	0.5222584	1	392	tags=40%, lis
GOBP_POSITIVE_REGULATION_OF_LEUKOCYTE_MIGRATION	25	0.270	1.219	0.2016129	0.53336996	1	312	tags=40%, lis
HALLMARK_MYC_TARGETS_V1	47	0.223	1.218	0.18564357	0.534142	1	440	tags=47%, lis
GOCC_CATALYTIC_STEP_2_SPLICEOSOME	18	0.307	1.215	0.22169812	0.5382068	1	290	tags=39%, lis
GOBP_POSITIVE_REGULATION_OF_CHEMOTAXIS	22	0.283	1.214	0.20714286	0.5392723	1	44	tags=18%, lis
REACTOME_CELLULAR_RESPONSES_TO_EXTERNAL_STIMULI	90	0.192	1.214	0.14910026	0.53763944	1	361	tags=31%, lis
REACTOME_INNATE_IMMUNE_SYSTEM	212	0.164	1.213	0.12615384	0.5396746	1	307	tags=25%, lis
GOCC_FICOLIN_1_RICH_GRANULE	46	0.225	1.212	0.1892583	0.5402216	1	231	tags=24%, lis
GOBP_B_CELL_ACTIVATION	60	0.209	1.209	0.17322835	0.54437906	1	151	tags=15%, lis
GOCC_CELL_SURFACE	86	0.190	1.207	0.18302387	0.54644436	1	242	tags=21%, lis
GOMF_PHOSPHOLIPID_BINDING	48	0.221	1.207	0.22192514	0.5453978	1	77	tags=15%, lis
GOBP_REGULATION_OF_ACTIN_FILAMENT_BUNDLE_ASSEMBLY	15	0.321	1.206	0.23831776	0.5455783	1	526	tags=67%, lis

GOMF_RIBONUCLEOPROTEIN_COMPLEX_BINDING	21	0.290	1.205	0.25829384	0.5466238	1	238	tags=33%, lis
REACTOME_CELL_CYCLE_CHECKPOINTS	38	0.235	1.199	0.2319202	0.5577799	1	295	tags=34%, lis
GOBP_ENERGY_DERIVATION_BY_OXIDATION_OF_ORGANIC_COMPOUNDS	37	0.240	1.196	0.20941177	0.562944	1	414	tags=41%, lis
GOMF_DNA_BINDING_TRANSCRIPTION_REPRESSOR_ACTIVITY	21	0.281	1.196	0.225	0.5624728	1	168	tags=24%, lis
GOCC_P_BODY	19	0.290	1.188	0.2617801	0.5786269	1	1167	tags=100%, l
GOCC_MEMBRANE_MICRODOMAIN	54	0.213	1.187	0.21012658	0.57846224	1	142	tags=17%, lis
GOBP_PEPTIDYL_THREONINE_MODIFICATION	17	0.301	1.186	0.26024097	0.5780772	1	533	tags=59%, lis
REACTOME_PTEN_REGULATION	30	0.247	1.185	0.2382134	0.5796208	1	361	tags=40%, lis
GOBP_CHROMATIN_REMODELING	23	0.279	1.184	0.24881516	0.58047205	1	111	tags=17%, lis
GOCC_RNA_POLYMERASE_II_TRANSCRIPTION_REGULATOR_COMPLEX	19	0.281	1.179	0.24657534	0.5891847	1	839	tags=84%, lis
GOMF_SIGNALING_RECEPTOR_BINDING	151	0.168	1.179	0.16879795	0.5882063	1	162	tags=15%, lis
GOBP_PEPTIDE_METABOLIC_PROCESS	105	0.181	1.178	0.21079692	0.5886132	1	304	tags=30%, lis
GOBP_ANTIGEN_PROCESSING_AND_PRESENTATION_OF_PEPTIDE_OR_POLYSACCHARIDE_ANTIGEN	21	0.282	1.177	0.24880382	0.5887526	1	135	tags=24%, lis
GOMF_CATION_CHANNEL_ACTIVITY	17	0.297	1.175	0.25480768	0.5925347	1	65	tags=18%, lis
GOMF_SEQUENCE_SPECIFIC_DNA_BINDING	125	0.175	1.172	0.20786516	0.59700894	1	178	tags=18%, lis
GOBP_NEGATIVE_REGULATION_OF_CELL_CELL_ADHESION	34	0.235	1.172	0.2751196	0.59531283	1	49	tags=12%, lis
GOBP_POSITIVE_REGULATION_OF_CELL_DEATH	87	0.185	1.172	0.21315789	0.59415084	1	173	tags=15%, lis
GOCC_ENVELOPE	132	0.166	1.169	0.20053476	0.5992389	1	392	tags=33%, lis
GOBP_MEMBRANE_ORGANIZATION	132	0.168	1.169	0.18987341	0.59746253	1	210	tags=17%, lis
GOCC_RESPIRASOME	16	0.301	1.168	0.26126125	0.5966747	1	314	tags=38%, lis
GOBP_RIBONUCLEOSIDE_TRIPHOSPHATE_METABOLIC_PROCESS	20	0.275	1.168	0.27543426	0.59657145	1	437	tags=50%, lis
REACTOME_MITOTIC_METAPHASE_AND_ANAPHASE	45	0.215	1.166	0.24087591	0.60000896	1	379	tags=40%, lis
GOBP_ION_HOMEOSTASIS	78	0.187	1.165	0.21657754	0.5986068	1	273	tags=26%, lis
GOBP_POSITIVE_REGULATION_OF_CELL_GROWTH	17	0.300	1.162	0.2800926	0.605787	1	1043	tags=94%, lis
GOCC_COPII_COATED_ER_TO_GOLGI_TRANSPORT_VESICLE	15	0.302	1.159	0.2524272	0.6093118	1	318	tags=40%, lis
GOBP_RESPONSE_TO_REACTIVE_OXYGEN_SPECIES	39	0.229	1.156	0.2770781	0.6168674	1	221	tags=21%, lis
GOBP_NEGATIVE_REGULATION_OF_CELL_ACTIVATION	37	0.224	1.154	0.2985782	0.6201336	1	49	tags=11%, lis
GOBP_REGULATION_OF_CELL_CYCLE_PHASE_TRANSITION	63	0.196	1.152	0.2512563	0.62314004	1	312	tags=29%, lis
GOBP_LEUKOCYTE_MIGRATION	79	0.186	1.151	0.24598931	0.62205374	1	142	tags=15%, lis
GOBP_BIOLOGICAL_PROCESS_INVOLVED_IN_SYMBIOTIC_INTERACTION	155	0.162	1.150	0.21311475	0.6242498	1	440	tags=38%, lis
GOBP_LIPOPROTEIN_METABOLIC_PROCESS	17	0.289	1.149	0.28534704	0.6239439	1	126	tags=24%, lis
GOCC_NUCLEOLUS	106	0.175	1.149	0.25779036	0.62289536	1	266	tags=23%, lis
GOBP_NEGATIVE_REGULATION_OF_BINDING	24	0.262	1.146	0.28092784	0.6268284	1	211	tags=25%, lis
GOBP_NEGATIVE_REGULATION_OF_TRANSCRIPTION_BY_RNA_POLYMERASE_II	94	0.176	1.145	0.23015873	0.62869483	1	179	tags=17%, lis
GOBP_PURINE_CONTAINING_COMPOUND_BIOSYNTHETIC_PROCESS	27	0.247	1.142	0.2878412	0.632352	1	77	tags=15%, lis
GOBP_REGULATION_OF_ACTIN_FILAMENT_LENGTH	31	0.233	1.142	0.27602905	0.6323256	1	400	tags=39%, lis
GOBP_LEUKOCYTE_MEDIATED_IMMUNITY	155	0.158	1.141	0.22922637	0.63123494	1	265	tags=20%, lis
GOBP_RIBOSE_PHOSPHATE_BIOSYNTHETIC_PROCESS	27	0.244	1.135	0.30147058	0.6448121	1	77	tags=15%, lis
KEGG_VIRAL_MYOCARDITIS	17	0.283	1.132	0.30215827	0.6518073	1	337	tags=41%, lis
GOBP_NEGATIVE_REGULATION_OF_MITOTIC_CELL_CYCLE	47	0.203	1.131	0.3147208	0.6515798	1	299	tags=28%, lis
REACTOME_MRNA_SPLICING	44	0.209	1.131	0.29104477	0.6502146	1	294	tags=30%, lis
GOCC_ENDOCYTIC_VESICLE_MEMBRANE	31	0.237	1.130	0.32054794	0.6503675	1	39	tags=13%, lis
HALLMARK_UV_RESPONSE_UP	28	0.240	1.129	0.31094527	0.65209734	1	373	tags=36%, lis
GOBP_REGULATION_OF_VIRAL_LIFE_CYCLE	21	0.263	1.128	0.3138686	0.65183496	1	324	tags=33%, lis
GOBP_MORPHOGENESIS_OF_AN_EPITHELIUM	50	0.200	1.122	0.3233945	0.6651996	1	361	tags=36%, lis
GOBP_REGULATION_OF_CHEMOTAXIS	26	0.247	1.122	0.30232558	0.6634029	1	173	tags=23%, lis
GOBP_DEVELOPMENT_OF_PRIMARY_SEXUAL_CHARACTERISTICS	20	0.271	1.121	0.28846154	0.664402	1	1062	tags=95%, lis
GOBP_NEGATIVE_REGULATION_OF_CELL_CYCLE_PROCESS	57	0.198	1.120	0.2868421	0.66417986	1	299	tags=26%, lis
GOBP_CELLULAR_KETONE_METABOLIC_PROCESS	30	0.236	1.120	0.32041344	0.66365296	1	295	tags=33%, lis
GOBP_REGULATION_OF_PEPTIDYL_SERINE_PHOSPHORYLATION	18	0.274	1.119	0.31516588	0.6638618	1	129	tags=22%, lis
GOCC_U2_TYPE_SPLICEOSOMAL_COMPLEX	20	0.270	1.119	0.28199053	0.66258264	1	609	tags=70%, lis
GOBP_POSITIVE_REGULATION_OF_INFLAMMATORY_RESPONSE	19	0.273	1.116	0.30120483	0.66819316	1	291	tags=32%, lis
GOBP_POSITIVE_REGULATION_OF_CELL_POPULATION_PROLIFERATION	101	0.171	1.113	0.26923078	0.67238533	1	203	tags=20%, lis
GOCC_MEMBRANE_PROTEIN_COMPLEX	150	0.154	1.111	0.27671233	0.67660975	1	401	tags=33%, lis
GOBP_NEGATIVE_REGULATION_OF_GENE_EXPRESSION	167	0.152	1.110	0.27440634	0.6779043	1	306	tags=25%, lis
HALLMARK_MTORC1_SIGNALING	32	0.231	1.108	0.32696897	0.6801112	1	505	tags=53%, lis
GOBP_NEGATIVE_REGULATION_OF_BIOSYNTHETIC_PROCESS	172	0.152	1.108	0.26259947	0.6784997	1	211	tags=18%, lis
GOBP_RESPONSE_TO_METAL_ION	42	0.219	1.103	0.33004925	0.6888848	1	203	tags=21%, lis
GOBP_NEGATIVE_REGULATION_OF_CELLULAR_AMIDE_METABOLIC_PROCESS	26	0.242	1.103	0.32741117	0.6877914	1	491	tags=58%, lis
GOBP_TISSUE_HOMEOSTASIS	22	0.256	1.102	0.33333334	0.6882551	1	71	tags=18%, lis
GOBP_REGULATION_OF_LEUKOCYTE_PROLIFERATION	37	0.214	1.102	0.33	0.6867158	1	47	tags=11%, lis
GOCC_CHROMATIN	109	0.168	1.102	0.28791773	0.6856556	1	168	tags=16%, lis
REACTOME_ESTROGEN_DEPENDENT_GENE_EXPRESSION	22	0.258	1.101	0.34396356	0.6849428	1	1026	tags=91%, lis
GOBP_ORGANIC_CYCLIC_COMPOUND_CATABOLIC_PROCESS	85	0.175	1.101	0.29234973	0.68408686	1	299	tags=27%, lis
GOCC_ACTIN_BASED_CELL_PROJECTION	25	0.242	1.099	0.2985437	0.68580425	1	129	tags=16%, lis
GOMF_ION_TRANSMEMBRANE_TRANSPORTER_ACTIVITY	69	0.185	1.099	0.31593406	0.68407923	1	109	tags=13%, lis
GOBP_REGULATION_OF_RESPONSE_TO_STRESS	168	0.149	1.097	0.2797784	0.68930185	1	324	tags=24%, lis
GOBP_RESPONSE_TO_TOXIC_SUBSTANCE	34	0.221	1.096	0.3137255	0.6900942	1	272	tags=24%, lis
GOBP_STEM_CELL_DIFFERENTIATION	33	0.228	1.094	0.32637075	0.69109666	1	295	tags=30%, lis
GOBP_RIBONUCLEOPROTEIN_COMPLEX_BIOGENESIS	49	0.201	1.091	0.30917874	0.69764	1	290	tags=31%, lis
GOMF_CATION_TRANSMEMBRANE_TRANSPORTER_ACTIVITY	44	0.203	1.090	0.33417085	0.69860613	1	144	tags=16%, lis
GOMF_TRANSITION_METAL_ION_BINDING	86	0.169	1.084	0.31524548	0.7133913	1	146	tags=13%, lis
GOBP_POSITIVE_REGULATION_OF_PEPTIDASE_ACTIVITY	34	0.215	1.075	0.379717	0.7327427	1	180	tags=18%, lis
GOBP_RESPONSE_TO_NUTRIENT	17	0.267	1.072	0.3537736	0.740978	1	1052	tags=94%, lis
GOBP_RNA_SPLICING_VIA_TRANSESTERIFICATION_REACTIONS	72	0.185	1.071	0.32707775	0.7409677	1	310	tags=29%, lis
GOBP_ACTIVATION_OF_CYSTEINE_TYPE_ENDOPEPTIDASE_ACTIVITY_INVOLVED_IN_APOPTOTIC_PR	15	0.286	1.071	0.37288135	0.7391267	1	373	tags=40%, lis
GOMF_GTPASE_ACTIVITY	40	0.209	1.068	0.35731414	0.7438583	1	110	tags=13%, lis
GOBP_CELLULAR_RESPONSE_TO_OXYGEN_LEVELS	42	0.209	1.068	0.37007874	0.7423674	1	491	tags=52%, lis
GOBP_NUCLEOSIDE_TRIPHOSPHATE_METABOLIC_PROCESS	22	0.250	1.068	0.3652393	0.7414148	1	65	tags=14%, lis

GOBP_REGULATION_OF_INTRINSIC_APOPTOTIC_SIGNALING_PATHWAY	26	0.238	1.068	0.38918918	0.73984593	1	349	tags=35%, lis
GOBP_REGULATION_OF_DNA_REPLICATION	19	0.260	1.065	0.37558687	0.74416304	1	306	tags=37%, lis
GOBP_REGULATION_OF_SECRETION	55	0.187	1.065	0.34986946	0.74316734	1	80	tags=11%, lis
GOBP_LEUKOCYTE_MEDIATED_CYTOTOXICITY	15	0.280	1.065	0.34708738	0.74133563	1	1010	tags=93%, lis
GOBP_INTRINSIC_APOPTOTIC_SIGNALING_PATHWAY	48	0.194	1.064	0.35917312	0.7406992	1	83	tags=10%, lis
HALLMARK_EPITHELIAL_MESENCHYMAL_TRANSITION	21	0.253	1.064	0.36792454	0.74049807	1	335	tags=43%, lis
GOBP_REGULATION_OF_GROWTH	68	0.178	1.062	0.34005037	0.7427293	1	161	tags=16%, lis
GOCC_INTRINSIC_COMPONENT_OF_ENDOPLASMIC_RETICULUM_MEMBRANE	17	0.268	1.062	0.37563452	0.74233603	1	318	tags=35%, lis
REACTOME_M_PHASE	57	0.183	1.060	0.35786802	0.7454709	1	295	tags=30%, lis
GOBP_REGULATION_OF_CELL_ACTIVATION	88	0.170	1.059	0.3385013	0.7442639	1	71	tags=10%, lis
GOBP_REGULATION_OF_CELLULAR_CATABOLIC_PROCESS	145	0.148	1.059	0.33161953	0.74327374	1	303	tags=24%, lis
GOBP_RESPONSE_TO_CAMP	15	0.271	1.055	0.3959276	0.7522516	1	173	tags=27%, lis
GOBP_REGULATION_OF_ACTIN_FILAMENT_ORGANIZATION	43	0.202	1.053	0.39036146	0.7556205	1	339	tags=30%, lis
GOBP_REACTIVE_OXYGEN_SPECIES_METABOLIC_PROCESS	44	0.201	1.052	0.38059703	0.7551448	1	39	tags=7%, list
GOBP_DNA_REPLICATION	27	0.224	1.051	0.3598015	0.7555917	1	191	tags=22%, lis
GOBP_ORGANONITROGEN_COMPOUND_BIOSYNTHETIC_PROCESS	176	0.143	1.051	0.38636363	0.755608	1	277	tags=22%, lis
GOBP_REGULATION_OF_PEPTIDE_SECRETION	28	0.230	1.045	0.4117647	0.7673324	1	33	tags=11%, lis
HALLMARK_UNFOLDED_PROTEIN_RESPONSE	24	0.234	1.044	0.37871286	0.7700719	1	1259	tags=100%, l
GOBP_REGULATION_OF_DNA_BINDING	22	0.238	1.043	0.4192771	0.770312	1	91	tags=14%, lis
GOMF_DNA_BINDING_TRANSCRIPTION_FACTOR_ACTIVITY	84	0.166	1.042	0.40106952	0.76962036	1	168	tags=17%, lis
GOBP_LEUKOCYTE_CELL_CELL_ADHESION	71	0.174	1.042	0.36461127	0.76851887	1	71	tags=10%, lis
GOCC_CYTOPLASMIC_STRESS_GRANULE	18	0.256	1.042	0.39225182	0.7678662	1	231	tags=28%, lis
GOMF_RECEPTOR_REGULATOR_ACTIVITY	17	0.269	1.041	0.4053398	0.7665866	1	401	tags=47%, lis
REACTOME_ESR_MEDIATED_SIGNALING	29	0.223	1.035	0.40944883	0.7821581	1	1278	tags=100%, l
GOCC_PHAGOCYTIC_VESICLE	34	0.209	1.029	0.43448275	0.796422	1	170	tags=18%, lis
GOMF_ACTIVE_ION_TRANSMEMBRANE_TRANSPORTER_ACTIVITY	15	0.278	1.029	0.40875912	0.7951883	1	144	tags=20%, lis
GOMF_TRANSCRIPTION_REGULATOR_ACTIVITY	158	0.143	1.028	0.42896935	0.7952309	1	183	tags=15%, lis
GOBP_POSITIVE_REGULATION_OF_GROWTH	23	0.232	1.027	0.39903846	0.7961581	1	1262	tags=100%, l
REACTOME_PROCESSING_OF_CAPPED_INTRON_CONTAINING_PRE_MRNA	46	0.191	1.026	0.41469815	0.7973443	1	294	tags=28%, lis
GOBP_NEGATIVE_REGULATION_OF_RESPONSE_TO_EXTERNAL_STIMULUS	31	0.211	1.023	0.43157893	0.8009999	1	173	tags=23%, lis
GOBP_POSITIVE_REGULATION_OF_TRANSCRIPTION_BY_RNA_POLYMERASE_II	130	0.148	1.023	0.41558442	0.80012524	1	178	tags=15%, lis
GOBP_POSITIVE_REGULATION_OF_PROTEIN_SERINE_THREONINE_KINASE_ACTIVITY	42	0.192	1.021	0.39896372	0.80293083	1	71	tags=12%, lis
GOBP_ANIMAL_ORGAN_MORPHOGENESIS	80	0.167	1.019	0.4235925	0.80645317	1	229	tags=21%, lis
GOBP_MRNA_PROCESSING	97	0.157	1.016	0.4237726	0.8134955	1	294	tags=27%, lis
GOBP_FC_RECEPTOR_MEDIATED_STIMULATORY_SIGNALING_PATHWAY	32	0.210	1.014	0.44285715	0.8175097	1	169	tags=19%, lis
GOBP_LYMPHOCYTE_ACTIVATION	128	0.148	1.012	0.43650794	0.82004815	1	49	tags=8%, list
GOMF_RNA_BINDING	271	0.127	1.006	0.42153847	0.83276117	1	297	tags=23%, lis
GOBP_TAXIS	72	0.167	1.006	0.45738637	0.8321523	1	143	tags=14%, lis
GOBP_DEFENSE_RESPONSE_TO_VIRUS	31	0.214	1.004	0.4538835	0.83428586	1	291	tags=29%, lis
GOBP_PHAGOCYTOSIS	71	0.168	1.002	0.4631579	0.8376025	1	169	tags=17%, lis
GOBP_CELL_CYCLE_ARREST	24	0.223	1.000	0.43523315	0.8415528	1	1277	tags=100%, l
GOMF_KINASE_REGULATOR_ACTIVITY	32	0.205	0.997	0.45833334	0.84679824	1	138	tags=16%, lis
GOBP_POSITIVE_REGULATION_OF_CYSTEINE_TYPE_ENDOPEPTIDASE_ACTIVITY	25	0.222	0.995	0.46268657	0.85041356	1	314	tags=28%, lis
GOBP_RNA_EXPORT_FROM_NUCLEUS	18	0.252	0.994	0.464191	0.8513918	1	424	tags=50%, lis
PID_MTOR_4PATHWAY	16	0.257	0.993	0.45232275	0.853472	1	393	tags=50%, lis
GOBP_PEPTIDE_SECRETION	35	0.196	0.992	0.472	0.8524016	1	152	tags=14%, lis
GOBP_MYELOID_LEUKOCYTE_ACTIVATION	140	0.141	0.992	0.48051947	0.85107505	1	319	tags=24%, lis
GOCC_FILOPODIUM	16	0.255	0.991	0.44362745	0.851313	1	1087	tags=94%, lis
GOBP_PROTEIN_CONTAINING_COMPLEX_DISASSEMBLY	35	0.202	0.991	0.44974875	0.849453	1	120	tags=14%, lis
REACTOME_MAPK_FAMILY_SIGNALING_CASCADES	58	0.170	0.990	0.47619048	0.851562	1	341	tags=31%, lis
GOBP_TISSUE_MORPHOGENESIS	57	0.174	0.985	0.45967743	0.86374897	1	361	tags=33%, lis
GOBP_TYPE_I_INTERFERON_PRODUCTION	25	0.225	0.984	0.4516129	0.86263096	1	168	tags=20%, lis
GOBP_CHEMICAL_HOMEOSTASIS	117	0.146	0.984	0.49171272	0.861155	1	257	tags=21%, lis
GOBP_BONE_DEVELOPMENT	17	0.245	0.981	0.479798	0.86753106	1	486	tags=59%, lis
GOBP_CHEMOKINE_PRODUCTION	22	0.231	0.981	0.48866498	0.8668608	1	185	tags=18%, lis
GOBP_REGULATION_OF_CYTOSOLIC_CALCIUM_ION_CONCENTRATION	23	0.229	0.981	0.4728682	0.86503106	1	401	tags=43%, lis
GOBP_NEGATIVE_REGULATION_OF_PROTEOLYSIS	32	0.199	0.980	0.4842932	0.8648997	1	282	tags=25%, lis
GOBP_ACTIN_FILAMENT_ORGANIZATION	62	0.167	0.980	0.49046323	0.8630542	1	315	tags=26%, lis
GOBP_NEGATIVE_REGULATION_OF_HYDROLASE_ACTIVITY	43	0.185	0.979	0.47188264	0.86201215	1	257	tags=21%, lis
GOBP_REGULATION_OF_CELL_PROJECTION_ASSEMBLY	24	0.221	0.978	0.4755784	0.863743	1	83	tags=13%, lis
GOBP_NEGATIVE_REGULATION_OF_LEUKOCYTE_CELL_CELL_ADHESION	30	0.203	0.975	0.5186104	0.8696249	1	49	tags=10%, lis
GOBP_PROTEIN_DNA_COMPLEX_SUBUNIT_ORGANIZATION	23	0.227	0.974	0.491358	0.8711298	1	463	tags=48%, lis
GOBP_RESPONSE_TO_ORGANOPHOSPHORUS	16	0.253	0.972	0.49526066	0.8745136	1	173	tags=25%, lis
GOBP_REGULATION_OF_EPITHELIAL_CELL_DIFFERENTIATION	18	0.244	0.971	0.502451	0.87482494	1	783	tags=83%, lis
GOBP_INTRINSIC_APOPTOTIC_SIGNALING_PATHWAY_IN_RESPONSE_TO_DNA_DAMAGE	16	0.254	0.969	0.49238577	0.87769336	1	28	tags=13%, lis
GOBP_RESPONSE_TO_VIRUS	48	0.176	0.969	0.5012854	0.87632656	1	226	tags=21%, lis
GOBP_CELLULAR_HOMEOSTASIS	99	0.148	0.968	0.4895288	0.87732905	1	257	tags=20%, lis
GOBP_POSITIVE_REGULATION_OF_SIGNALING	203	0.128	0.968	0.5488506	0.87548673	1	310	tags=23%, lis
GOBP_RESPONSE_TO_TEMPERATURE_STIMULUS	19	0.241	0.967	0.4832536	0.87552667	1	262	tags=32%, lis
GOBP_POSITIVE_REGULATION_OF_GENE_EXPRESSION	128	0.141	0.966	0.54366195	0.87606835	1	297	tags=23%, lis
GOBP_REGULATION_OF_BIOLOGICAL_PROCESS_INVOLVED_IN_SYMBIOTIC_INTERACTION	31	0.202	0.958	0.48586118	0.89381933	1	164	tags=16%, lis
GOCC_SPINDLE	40	0.183	0.957	0.49753696	0.8951968	1	205	tags=23%, lis
GOBP_POSITIVE_REGULATION_OF_PLASMA_MEMBRANE_BOUNDED_CELL_PROJECTION_ASSEMBL	18	0.236	0.955	0.49339208	0.89997774	1	39	tags=11%, lis
GOCC_NUCLEAR_BODY	115	0.143	0.952	0.53351206	0.9039601	1	310	tags=24%, lis
REACTOME_SIGNALING_BY_PTK6	17	0.241	0.952	0.5192308	0.902817	1	407	tags=47%, lis
GOBP_REGULATION_OF_CELL_DEATH	202	0.127	0.951	0.62739724	0.9034035	1	310	tags=22%, lis
GOBP_REGULATION_OF_PROTEIN_BINDING	33	0.196	0.950	0.50611246	0.90355617	1	142	tags=18%, lis
GOBP_CELLULAR_PROTEIN_CONTAINING_COMPLEX_ASSEMBLY	131	0.136	0.950	0.5748663	0.90318924	1	315	tags=24%, lis
REACTOME_NEUTROPHIL_DEGRANULATION	117	0.138	0.949	0.54617417	0.9040497	1	306	tags=23%, lis

GOCC_SITE_OF_POLARIZED_GROWTH	29	0.203	0.949	0.5423729	0.9023021	1	162	tags=21%, lis
GOBP_IMMUNE_EFFECTOR_PROCESS	225	0.124	0.947	0.5892351	0.90444213	1	265	tags=18%, lis
GOBP_REGULATION_OF_MACROAUTOPHAGY	23	0.215	0.947	0.50127226	0.90332186	1	76	tags=13%, lis
GOBP_CELLULAR_ION_HOMEOSTASIS	68	0.159	0.946	0.5611702	0.9022951	1	273	tags=24%, lis
GOBP_IMPORT_INTO_NUCLEUS	19	0.230	0.946	0.51300234	0.9021975	1	152	tags=21%, lis
REACTOME_UNFOLDED_PROTEIN_RESPONSE_UPR	19	0.233	0.944	0.5219512	0.9040194	1	1259	tags=100%, l
GOMF_TRANSCRIPTION_FACTOR_BINDING	93	0.149	0.943	0.5734072	0.90428203	1	183	tags=15%, lis
GOBP_NEGATIVE_REGULATION_OF_ORGANELLE_ORGANIZATION	31	0.201	0.943	0.52987015	0.9036772	1	203	tags=23%, lis
GOMF_MRNA_3_UTR_BINDING	20	0.226	0.942	0.55445546	0.90495026	1	842	tags=80%, lis
REACTOME_TOLL_LIKE_RECEPTOR_TLR1_TLR2_CASCADE	20	0.223	0.940	0.5125	0.90737975	1	129	tags=15%, lis
REACTOME_CYTOKINE_SIGNALING_IN_IMMUNE_SYSTEM	119	0.135	0.939	0.5899471	0.9073056	1	407	tags=33%, lis
GOBP_POSITIVE_REGULATION_OF_CELL_CYCLE_PROCESS	29	0.198	0.937	0.5595855	0.9110731	1	611	tags=66%, lis
GOBP_REGULATION_OF_SYNAPSE_STRUCTURE_OR_ACTIVITY	19	0.225	0.936	0.5289855	0.91025454	1	264	tags=32%, lis
GOBP_EPITHELIUM_DEVELOPMENT	107	0.140	0.936	0.59947646	0.9092681	1	371	tags=30%, lis
GOMF_CYSTEINE_TYPE_PEPTIDASE_ACTIVITY	27	0.201	0.935	0.5245098	0.9113824	1	281	tags=30%, lis
GOCC_CILIUM	30	0.199	0.932	0.56264776	0.9154726	1	143	tags=17%, lis
GOMF_HELICASE_ACTIVITY	19	0.225	0.931	0.55609757	0.91650814	1	1142	tags=95%, lis
REACTOME_DISEASES_OF_SIGNAL_TRANSDUCTION_BY_GROWTH_FACTOR_RECEPTORS_AND_SEC	81	0.149	0.929	0.5605263	0.9184855	1	238	tags=21%, lis
GOBP_POSITIVE_REGULATION_OF_PEPTIDE_SECRETION	16	0.242	0.929	0.5550351	0.91825694	1	3	tags=6%, list
GOBP_RESPONSE_TO_ABIOTIC_STIMULUS	147	0.131	0.927	0.60742706	0.92171913	1	307	tags=23%, lis
GOBP_CELL_KILLING	20	0.224	0.924	0.54285717	0.92564124	1	264	tags=30%, lis
REACTOME_RHO_GTPASE_EFFECTORS	46	0.170	0.924	0.5756824	0.9255281	1	342	tags=28%, lis
GOBP_HEMATOPOIETIC_PROGENITOR_CELL_DIFFERENTIATION	35	0.186	0.922	0.58171743	0.92687345	1	295	tags=29%, lis
GOBP_CELLULAR_RESPONSE_TO_RADIATION	24	0.208	0.921	0.5829146	0.9278124	1	166	tags=17%, lis
GOMF_PROTEIN_HOMODIMERIZATION_ACTIVITY	65	0.158	0.921	0.58056873	0.9270678	1	73	tags=9%, list
GOBP_REGULATION_OF_IMMUNE_EFFECTOR_PROCESS	62	0.159	0.919	0.6210526	0.93056893	1	148	tags=13%, lis
GOBP_NEGATIVE_REGULATION_OF_RNA_CATABOLIC_PROCESS	15	0.245	0.918	0.54846334	0.9292781	1	165	tags=27%, lis
GOBP_NEGATIVE_REGULATION_OF_CELL_ADHESION	49	0.167	0.917	0.58914727	0.9312275	1	202	tags=16%, lis
GOCC_PHOSPHATASE_COMPLEX	15	0.247	0.917	0.5579196	0.93014544	1	314	tags=40%, lis
GOBP_REGULATION_OF_T_CELL_DIFFERENTIATION	20	0.224	0.916	0.5569307	0.9296563	1	49	tags=10%, lis
GOBP_RESPONSE_TO_OXIDATIVE_STRESS	65	0.152	0.916	0.5945946	0.928709	1	324	tags=25%, lis
GOBP_POSITIVE_REGULATION_OF_MRNA_METABOLIC_PROCESS	19	0.228	0.914	0.5640327	0.93056405	1	870	tags=84%, lis
GOBP_CELLULAR_RESPONSE_TO_ABIOTIC_STIMULUS	47	0.166	0.913	0.5883777	0.9313734	1	203	tags=17%, lis
GOCC_NUCLEAR_CHROMOSOME	20	0.218	0.911	0.5555556	0.93352306	1	238	tags=25%, lis
GOBP_INFLAMMATORY_RESPONSE	83	0.148	0.910	0.64285713	0.9344715	1	312	tags=23%, lis
GOMF_HYDROLASE_ACTIVITY_ACTING_ON_ACID_ANHYDRIDES	97	0.141	0.908	0.64483625	0.9383645	1	110	tags=9%, list
GOMF_PASSIVE_TRANSMEMBRANE_TRANSPORTER_ACTIVITY	24	0.210	0.908	0.6	0.93666345	1	65	tags=13%, lis
GOBP_CATION_TRANSPORT	88	0.143	0.908	0.64324325	0.935533	1	205	tags=17%, lis
GOBP_POSITIVE_REGULATION_OF_CELL_MORPHOGENESIS_INVOLVED_IN_DIFFERENTIATION	16	0.238	0.907	0.5714286	0.9346746	1	432	tags=50%, lis
GOBP_TISSUE_REMODELING	17	0.232	0.907	0.5714286	0.93295705	1	211	tags=24%, lis
GOBP_PRODUCTION_OF_MOLECULAR_MEDIATOR_OF_IMMUNE_RESPONSE	30	0.189	0.905	0.5985401	0.9353389	1	44	tags=10%, lis
GOBP_RNA_PROCESSING	134	0.132	0.905	0.6290801	0.9341425	1	294	tags=24%, lis
GOBP_NEGATIVE_REGULATION_OF_GROWTH	26	0.193	0.904	0.5829146	0.93470275	1	143	tags=19%, lis
HALLMARK_FATTY_ACID_METABOLISM	16	0.233	0.903	0.5738095	0.9363432	1	500	tags=56%, lis
GOBP_PROTEIN_CONTAINING_COMPLEX_SUBUNIT_ORGANIZATION	218	0.120	0.900	0.70844686	0.9415248	1	341	tags=25%, lis
GOBP_EMBRYONIC_MORPHOGENESIS	42	0.169	0.899	0.643038	0.9400638	1	221	tags=21%, lis
GOMF_PROTEIN_DIMERIZATION_ACTIVITY	97	0.138	0.899	0.62362635	0.9400731	1	73	tags=8%, list
GOBP_SPLICEOSOMAL_COMPLEX_ASSEMBLY	17	0.232	0.898	0.5750636	0.9409197	1	290	tags=35%, lis
GOCC_GOLGI_ASSOCIATED_VESICLE	18	0.220	0.897	0.5770171	0.9394404	1	189	tags=22%, lis
GOBP_CARBOHYDRATE_BIOSYNTHETIC_PROCESS	25	0.199	0.897	0.6119792	0.93850523	1	166	tags=20%, lis
GOBP_MONOVALENT_INORGANIC_CATION_HOMEOSTASIS	21	0.216	0.897	0.59661835	0.9375995	1	214	tags=24%, lis
GOBP_SUBSTRATE_ADHESION_DEPENDENT_CELL_SPREADING	17	0.227	0.894	0.58554214	0.94224876	1	1268	tags=100%, l
HALLMARK_P53_PATHWAY	31	0.185	0.893	0.61097854	0.94128925	1	210	tags=19%, lis
KEGG_B_CELL_RECEPTOR_SIGNALING_PATHWAY	21	0.214	0.891	0.6188119	0.94579864	1	39	tags=10%, lis
GOBP_CELL_CELL_ADHESION	101	0.136	0.890	0.66219836	0.9457015	1	71	tags=8%, list
GOBP_REGULATION_OF_CELLULAR_COMPONENT_SIZE	55	0.159	0.890	0.6083551	0.9444096	1	120	tags=13%, lis
GOBP_CELL_GROWTH	57	0.153	0.890	0.62983423	0.9429048	1	161	tags=14%, lis
REACTOME_POST_TRANSLATIONAL_PROTEIN_MODIFICATION	161	0.121	0.889	0.6875	0.9416953	1	259	tags=20%, lis
GOBP_MITOCHONDRION_ORGANIZATION	69	0.148	0.888	0.6531165	0.94343036	1	393	tags=36%, lis
GOBP_NUCLEAR_TRANSCRIBED_MRNA_CATABOLIC_PROCESS_DEADENYLATION_DEPENDENT_DEC	18	0.218	0.887	0.6028708	0.9429563	1	824	tags=83%, lis
GOCC_FIBRILLAR_CENTER	19	0.218	0.887	0.60850114	0.94141954	1	812	tags=79%, lis
GOBP_GASTRULATION	16	0.232	0.887	0.6128266	0.9398986	1	221	tags=25%, lis
GOCC_COATED_VESICLE_MEMBRANE	23	0.198	0.885	0.59134614	0.942057	1	189	tags=22%, lis
GOCC_CHROMOSOME	170	0.120	0.885	0.713881	0.94199735	1	174	tags=14%, lis
GOBP_MULTICELLULAR_ORGANISMAL_HOMEOSTASIS	46	0.165	0.884	0.64075065	0.94268614	1	180	tags=17%, lis
GOBP_REGULATION_OF_CELL_MORPHOGENESIS_INVOLVED_IN_DIFFERENTIATION	17	0.224	0.882	0.61165047	0.9436574	1	324	tags=35%, lis
GOBP_EPITHELIAL_CELL_DEVELOPMENT	18	0.218	0.881	0.59090906	0.9439613	1	371	tags=39%, lis
GOBP_GLIAL_CELL_DIFFERENTIATION	22	0.206	0.881	0.6299766	0.94231135	1	129	tags=14%, lis
GOCC_LEADING_EDGE_MEMBRANE	26	0.189	0.880	0.61323154	0.94413644	1	339	tags=31%, lis
GOBP_MYELOID_LEUKOCYTE_DIFFERENTIATION	32	0.184	0.879	0.6179487	0.94518524	1	143	tags=16%, lis
GOBP_NEGATIVE_REGULATION_OF_PROTEIN_METABOLIC_PROCESS	134	0.124	0.877	0.7295082	0.9475242	1	299	tags=22%, lis
GOBP_PROTEIN_PHOSPHOPANTETHEINYLATION	33	0.176	0.877	0.60598505	0.94590104	1	170	tags=18%, lis
GOBP_MYELOID_LEUKOCYTE_MEDIATED_IMMUNITY	130	0.126	0.876	0.690411	0.94468904	1	306	tags=22%, lis
GOCC_TERTIARY_GRANULE_LUMEN	17	0.224	0.876	0.57425743	0.9447861	1	192	tags=24%, lis
GOBP_SIGNAL_TRANSDUCTION_BY_P53_CLASS_MEDIATOR	27	0.190	0.875	0.64721483	0.9436278	1	28	tags=7%, list
GOMF_TRANSPORTER_ACTIVITY	86	0.137	0.873	0.6818182	0.94670963	1	109	tags=10%, lis
GOBP_PROTEIN_LOCALIZATION_TO_CHROMOSOME	17	0.213	0.873	0.6426799	0.945073	1	231	tags=24%, lis
GOBP_REGULATION_OF_PROTEOLYSIS	96	0.134	0.872	0.6607595	0.94493675	1	282	tags=22%, lis
REACTOME_NEDDYLATION	37	0.175	0.871	0.63037974	0.94579667	1	295	tags=30%, lis

GOBP_SECRETION	202	0.117	0.870	0.73142856	0.946503	1	267	tags=18%, lis
GOBP_SENSORY_PERCEPTION	19	0.210	0.869	0.6188235	0.94651467	1	400	tags=42%, lis
GOBP_REGULATION_OF_APOPTOTIC_SIGNALING_PATHWAY	45	0.163	0.867	0.68367344	0.9493158	1	349	tags=29%, lis
REACTOME_CELL_CYCLE	76	0.142	0.867	0.7098446	0.94887877	1	295	tags=25%, lis
REACTOME_VESICLE_MEDIATED_TRANSPORT	99	0.131	0.867	0.70341206	0.9472928	1	259	tags=19%, lis
GOBP_METAL_ION_HOMEOSTASIS	57	0.154	0.866	0.68333334	0.94729257	1	273	tags=23%, lis
GOBP_INSULIN_SECRETION	16	0.215	0.865	0.65869564	0.948106	1	152	tags=19%, lis
GOCC_RECYCLING_ENDOSOME	25	0.191	0.864	0.6361186	0.9468437	1	170	tags=20%, lis
GOBP_REGULATION_OF_INFLAMMATORY_RESPONSE	38	0.168	0.864	0.6593674	0.9457887	1	291	tags=24%, lis
GOCC_CELL_PROJECTION_MEMBRANE	36	0.175	0.863	0.6277916	0.94655174	1	1356	tags=100%, l
GOBP_REGULATION_OF_CELL_CYCLE_G1_S_PHASE_TRANSITION	20	0.210	0.861	0.62009805	0.94964296	1	1026	tags=90%, lis
KEGG_ADHERENS_JUNCTION	15	0.232	0.861	0.6056338	0.9485597	1	526	tags=60%, lis
GOMF_ACTIN_BINDING	43	0.165	0.859	0.6827411	0.9499862	1	400	tags=37%, lis
GOCC_COATED_VESICLE	37	0.169	0.859	0.69095474	0.9483379	1	189	tags=19%, lis
GOBP_MITOTIC_CELL_CYCLE_CHECKPOINT	19	0.214	0.856	0.64248705	0.952891	1	1026	tags=89%, lis
GOBP_IMMUNOGLOBULIN_PRODUCTION	17	0.218	0.856	0.6824645	0.951362	1	44	tags=12%, lis
GOCC_RUFFLE	36	0.169	0.856	0.6780488	0.9504072	1	339	tags=33%, lis
GOBP_PEPTIDYL_TYROSINE_MODIFICATION	48	0.161	0.855	0.6622691	0.9501486	1	141	tags=15%, lis
REACTOME_NUCLEOTIDE_BINDING_DOMAIN_LEUCINE_RICH_REPEAT_CONTAINING_RECEPTOR_NI	15	0.230	0.853	0.64066195	0.95207155	1	341	tags=40%, lis
GOMF_PHOSPHATIDYLINOSITOL_BINDING	28	0.183	0.853	0.63709676	0.95114094	1	53	tags=11%, lis
GOBP_POSITIVE_REGULATION_OF_NF_KAPPAB_TRANSCRIPTION_FACTOR_ACTIVITY	26	0.185	0.852	0.66169155	0.9519943	1	129	tags=12%, lis
GOBP_REGULATION_OF_DENDRITE_DEVELOPMENT	18	0.211	0.850	0.6223278	0.9537884	1	161	tags=22%, lis
GOBP_EXOCYTOSIS	165	0.117	0.847	0.7616438	0.95847166	1	267	tags=18%, lis
GOBP_SIGNAL_TRANSDUCTION_IN_RESPONSE_TO_DNA_DAMAGE	15	0.222	0.844	0.6528736	0.96336025	1	1277	tags=100%, l
GOCC_CYTOPLASMIC_SIDE_OF_MEMBRANE	26	0.183	0.843	0.6515151	0.962361	1	83	tags=12%, lis
GOBP_ADAPTIVE_THERMOGENESIS	15	0.229	0.843	0.65831435	0.96245426	1	221	tags=27%, lis
GOBP_CYTOKINE_PRODUCTION	120	0.123	0.842	0.7780612	0.9613231	1	186	tags=13%, lis
GOCC_EXOCYTIC_VESICLE	17	0.215	0.842	0.69922876	0.96004397	1	129	tags=18%, lis
GOMF_TRANSCRIPTION_COREPRESSOR_ACTIVITY	27	0.183	0.839	0.7173397	0.96583766	1	179	tags=19%, lis
GOBP_CELL_ACTIVATION_INVOLVED_IN_IMMUNE_RESPONSE	159	0.118	0.838	0.78350514	0.9644416	1	265	tags=18%, lis
GOBP_PATTERN_RECOGNITION_RECEPTOR_SIGNALING_PATHWAY	35	0.168	0.837	0.6847826	0.96472573	1	83	tags=9%, list-
GOBP_CELLULAR_EXTRAVASATION	15	0.225	0.836	0.6302326	0.9666618	1	239	tags=27%, lis
GOBP_POSITIVE_REGULATION_OF_BIOSYNTHETIC_PROCESS	227	0.108	0.833	0.8227147	0.97064215	1	282	tags=19%, lis
GOBP_NUCLEAR_TRANSPORT	48	0.154	0.832	0.7135417	0.9712883	1	424	tags=40%, lis
GOBP_GLUCOSE_METABOLIC_PROCESS	20	0.199	0.831	0.68719214	0.97091657	1	166	tags=20%, lis
REACTOME_IMMUNOREGULATORY_INTERACTIONS_BETWEEN_A_LYMPHOID_AND_A_NON_LYMPH	16	0.214	0.830	0.65384614	0.9703299	1	638	tags=69%, lis
GOBP_POSITIVE_REGULATION_OF_DNA_BINDING_TRANSCRIPTION_FACTOR_ACTIVITY	36	0.167	0.830	0.6956522	0.9700587	1	129	tags=11%, lis
GOCC_ACTIN_CYTOSKELETON	64	0.139	0.828	0.72295517	0.97116417	1	337	tags=27%, lis
GOMF_IDENTICAL_PROTEIN_BINDING	200	0.110	0.828	0.8270893	0.9696015	1	73	tags=7%, list-
GOBP_CELLULAR_RESPONSE_TO_REACTIVE_OXYGEN_SPECIES	24	0.183	0.828	0.6789216	0.96851027	1	221	tags=21%, lis
GOBP_CELLULAR_GLUCOSE_HOMEOSTASIS	15	0.221	0.828	0.64483625	0.9677407	1	604	tags=60%, lis
GOMF_DNA_BINDING_TRANSCRIPTION_FACTOR_BINDING	61	0.145	0.826	0.74300253	0.96827984	1	166	tags=15%, lis
GOBP_CELL_CYCLE_PHASE_TRANSITION	78	0.133	0.826	0.74005306	0.966647	1	312	tags=24%, lis
REACTOME_CELL_CYCLE_MITOTIC	68	0.139	0.826	0.7595908	0.9651054	1	295	tags=25%, lis
GOBP_REGULATION_OF_AUTOPHAGY	48	0.152	0.826	0.7104623	0.9646986	1	76	tags=8%, list-
PID_BCR_5PATHWAY	22	0.190	0.825	0.6818182	0.96391386	1	71	tags=9%, list-
GOBP_CATION_TRANSMEMBRANE_TRANSPORT	69	0.138	0.824	0.7574124	0.9640644	1	147	tags=13%, lis
HALLMARK_INFLAMMATORY_RESPONSE	30	0.176	0.824	0.6968974	0.9636252	1	316	tags=30%, lis
GOMF_CHROMATIN_BINDING	73	0.134	0.823	0.77807486	0.96467984	1	148	tags=12%, lis
GOBP_SPHINGOLIPID_METABOLIC_PROCESS	17	0.207	0.820	0.6911392	0.968196	1	249	tags=29%, lis
GOBP_CENTRAL_NERVOUS_SYSTEM_DEVELOPMENT	88	0.129	0.819	0.7744361	0.9678662	1	407	tags=33%, lis
GOCC_RECEPTOR_COMPLEX	36	0.164	0.818	0.70153064	0.9675814	1	151	tags=14%, lis
GOBP_REGULATION_OF_GENE_EXPRESSION_EPIGENETIC	21	0.191	0.818	0.6666667	0.966867	1	75	tags=10%, lis
REACTOME_INFECTIOUS_DISEASE	129	0.119	0.818	0.8045326	0.9659594	1	310	tags=24%, lis
REACTOME_ASPARAGINE_N_LINKED_GLYCOSYLATION	42	0.156	0.817	0.7255937	0.9646529	1	259	tags=24%, lis
GOBP_REGULATION_OF_PEPTIDYL_TYROSINE_PHOSPHORYLATION	41	0.157	0.817	0.72704715	0.96313554	1	141	tags=15%, lis
GOBP_RESPONSE_TO_OXYGEN_CONTAINING_COMPOUND	195	0.110	0.816	0.86627907	0.96460694	1	231	tags=15%, lis
GOBP_NEGATIVE_REGULATION_OF_LEUKOCYTE_PROLIFERATION	16	0.205	0.815	0.6782178	0.9643509	1	6	tags=6%, list-
GOCC_MITOCHONDRION	153	0.114	0.815	0.8179348	0.96308357	1	68	tags=7%, list-
GOBP_MONONUCLEAR_CELL_MIGRATION	30	0.172	0.814	0.7468672	0.96297	1	451	tags=47%, lis
GOBP_SEX_DIFFERENTIATION	24	0.184	0.810	0.7361419	0.9690506	1	1062	tags=92%, lis
GOBP_RESPONSE_TO_KETONE	24	0.182	0.807	0.73536897	0.9727845	1	1025	tags=88%, lis
GOCC_NEURON_PROJECTION	97	0.124	0.806	0.7931937	0.9721345	1	166	tags=14%, lis
GOMF_MRNA_BINDING	57	0.145	0.804	0.77427185	0.97518724	1	292	tags=25%, lis
GOCC_AXON	51	0.145	0.803	0.7609255	0.9745366	1	162	tags=16%, lis
GOBP_POSITIVE_REGULATION_OF_PEPTIDYL_TYROSINE_PHOSPHORYLATION	27	0.173	0.802	0.7149877	0.97485876	1	186	tags=19%, lis
PID_ERA_GENOMIC_PATHWAY	15	0.220	0.802	0.7089552	0.9734847	1	251	tags=27%, lis
GOMF_ACTIVE_TRANSMEMBRANE_TRANSPORTER_ACTIVITY	21	0.188	0.800	0.7325301	0.975541	1	33	tags=10%, lis
REACTOME_INTRACELLULAR_SIGNALING_BY_SECOND_MESSENGERS	53	0.146	0.799	0.77002585	0.9758263	1	234	tags=21%, lis
GOBP_CYTOPLASMIC_PATTERN_RECOGNITION_RECEPTOR_SIGNALING_PATHWAY	15	0.211	0.797	0.6952141	0.9770489	1	83	tags=13%, lis
GOBP_REGULATION_OF_CATABOLIC_PROCESS	163	0.110	0.797	0.8698061	0.97645855	1	303	tags=21%, lis
GOCC_AZUROPHIL_GRANULE	44	0.151	0.797	0.75637394	0.97531784	1	287	tags=25%, lis
GOBP_MODULATION_OF_PROCESS_OF_OTHER_ORGANISM_INVOLVED_IN_SYMBIOTIC_INTERACTI	15	0.211	0.796	0.71229696	0.9744401	1	152	tags=20%, lis
GOBP_NEGATIVE_REGULATION_OF_MOLECULAR_FUNCTION	139	0.112	0.796	0.8123393	0.9737877	1	241	tags=17%, lis
GOBP_PROTEIN_STABILIZATION	29	0.172	0.793	0.75609756	0.9775799	1	80	tags=10%, lis
GOMF_TRANSCRIPTION_COACTIVATOR_ACTIVITY	43	0.147	0.790	0.7670886	0.97988296	1	214	tags=16%, lis
GOBP_CHROMATIN_ORGANIZATION	82	0.126	0.790	0.8312655	0.9791869	1	127	tags=11%, lis
GOMF_STRUCTURAL_MOLECULE_ACTIVITY	31	0.166	0.790	0.7480315	0.9779741	1	354	tags=32%, lis
GOMF_KINASE_BINDING	117	0.117	0.789	0.8426966	0.97665745	1	186	tags=15%, lis

GOBP_APOPTOTIC_SIGNALING_PATHWAY	79	0.129	0.787	0.8135593	0.97853446	1	47	tags=6%, list
GOMF_PHOSPHATASE_BINDING	30	0.168	0.787	0.75376886	0.9783835	1	1366	tags=100%, l
GOCC_ENDOPLASMIC_RETICULUM	197	0.106	0.782	0.90625	0.9850799	1	308	tags=23%, lis
GOBP_APOPTOTIC_PROCESS	249	0.101	0.781	0.9279539	0.98464864	1	234	tags=15%, lis
GOBP_HEAD_DEVELOPMENT	68	0.132	0.781	0.8125	0.98335856	1	403	tags=34%, lis
GOBP_PROCESS_UTILIZING_AUTOPHAGIC_MECHANISM	77	0.125	0.780	0.8037135	0.98396873	1	76	tags=6%, list
GOBP_POSITIVE_REGULATION_OF_CYTOKINE_PRODUCTION	66	0.130	0.779	0.81313133	0.9833072	1	185	tags=14%, lis
GOBP_PEPTIDE_HORMONE_SECRETION	17	0.197	0.779	0.74647886	0.98232305	1	152	tags=18%, lis
GOMF_HISTONE_BINDING	29	0.167	0.778	0.7310705	0.98198956	1	148	tags=14%, lis
GOCC_TRANSPORT_VESICLE	41	0.152	0.774	0.7839196	0.98648447	1	189	tags=17%, lis
GOBP_RESPONSE_TO_LIGHT_STIMULUS	29	0.161	0.771	0.7595908	0.9905503	1	173	tags=17%, lis
GOBP_RESPONSE_TO_PURINE_CONTAINING_COMPOUND	19	0.189	0.770	0.77803737	0.98957914	1	173	tags=21%, lis
GOMF_SH3_DOMAIN_BINDING	25	0.171	0.770	0.78516626	0.98849994	1	315	tags=28%, lis
REACTOME_ER_TO_GOLGI_ANTEROGRADE_TRANSPORT	21	0.178	0.769	0.7583732	0.9880918	1	259	tags=29%, lis
GOCC_GOLGI_APPARATUS_SUBCOMPARTMENT	112	0.114	0.768	0.8696808	0.98773736	1	211	tags=15%, lis
GOBP_LAMELLIPODIUM_ORGANIZATION	18	0.191	0.767	0.7555012	0.98923	1	39	tags=11%, lis
GOBP_CELLULAR_PROTEIN_COMPLEX_DISASSEMBLY	19	0.192	0.766	0.75736964	0.9882863	1	400	tags=37%, lis
REACTOME_TRANSCRIPTIONAL_REGULATION_OF_WHITE_ADIPOCYTE_DIFFERENTIATION	15	0.211	0.766	0.72979796	0.9867936	1	85	tags=13%, lis
GOBP_NEGATIVE_REGULATION_OF_CELL_CYCLE	81	0.122	0.766	0.8643617	0.9857814	1	299	tags=22%, lis
GOBP_REGULATION_OF_PROTEIN_POLYMERIZATION	36	0.151	0.765	0.8064516	0.985685	1	330	tags=28%, lis
GOBP_MODULATION_OF_PROCESS_OF_OTHER_ORGANISM	15	0.211	0.763	0.74413145	0.9872376	1	152	tags=20%, lis
BIOCARTA_MAPK_PATHWAY	16	0.198	0.762	0.7642487	0.98667383	1	1315	tags=100%, l
GOBP_PROTEIN_HOMOOLIGOMERIZATION	16	0.200	0.759	0.75888324	0.9908517	1	1148	tags=94%, lis
GOBP_REGULATION_OF_PH	18	0.192	0.758	0.7672811	0.98975736	1	214	tags=22%, lis
GOBP_REGULATION_OF_ANATOMICAL_STRUCTURE_SIZE	65	0.129	0.758	0.84514433	0.9891543	1	120	tags=11%, lis
REACTOME_HEMOSTASIS	89	0.120	0.756	0.8695652	0.99101263	1	142	tags=12%, lis
HALLMARK_ESTROGEN_RESPONSE_LATE	19	0.188	0.755	0.7642487	0.99061704	1	1018	tags=89%, lis
REACTOME_NERVOUS_SYSTEM_DEVELOPMENT	72	0.123	0.755	0.8772846	0.9892329	1	364	tags=29%, lis
GOBP_POSITIVE_REGULATION_OF_NUCLEOBASE_CONTAINING_COMPOUND_METABOLIC_PROCES	230	0.096	0.754	0.9299191	0.989002	1	270	tags=18%, lis
GOBP_POSITIVE_REGULATION_OF_CYTOSKELETON_ORGANIZATION	40	0.144	0.752	0.79949236	0.9899862	1	100	tags=10%, lis
KEGG_TIGHT_JUNCTION	15	0.199	0.752	0.8108108	0.98884284	1	88	tags=13%, lis
GOCC_TRANS_GOLGI_NETWORK_MEMBRANE	15	0.198	0.751	0.74168795	0.9880788	1	129	tags=13%, lis
GOBP_PROTEIN_LOCALIZATION_TO_MITOCHONDRION	20	0.179	0.751	0.76600987	0.9878481	1	393	tags=45%, lis
GOCC_SECRETORY_VESICLE	151	0.106	0.749	0.899705	0.9889311	1	267	tags=18%, lis
REACTOME_MITOTIC_PROMETAPHASE	24	0.170	0.749	0.7562189	0.9875166	1	269	tags=29%, lis
GOBP_HORMONE_TRANSPORT	18	0.188	0.749	0.7803618	0.98607236	1	152	tags=17%, lis
GOCC_CELL_SUBSTRATE_JUNCTION	78	0.121	0.749	0.85820895	0.98476535	1	1448	tags=100%, l
GOMF_TRANSFERASE_ACTIVITY_TRANSFERRING_GLYCOSYL_GROUPS	27	0.163	0.748	0.8062954	0.98360944	1	249	tags=22%, lis
GOBP_PROTEIN_FOLDING	31	0.157	0.747	0.84299517	0.9842936	1	334	tags=29%, lis
KEGG_SPLICEOSOME	34	0.149	0.745	0.7985258	0.9856306	1	290	tags=26%, lis
GOMF_TRANSCRIPTION_COREGULATOR_ACTIVITY	74	0.121	0.744	0.86340207	0.9850214	1	183	tags=14%, lis
GOBP_CELLULAR_RESPONSE_TO_DNA_DAMAGE_STIMULUS	100	0.114	0.744	0.8792135	0.98382276	1	115	tags=9%, list
REACTOME_SIGNALING_BY_TGF_BETA_RECEPTOR_COMPLEX	17	0.187	0.740	0.8213457	0.9873805	1	982	tags=88%, lis
GOBP_EPITHELIAL_TO_MESENCHYMAL_TRANSITION	16	0.193	0.740	0.79425836	0.9863176	1	75	tags=13%, lis
REACTOME_NEGATIVE_REGULATION_OF_MAPK_PATHWAY	15	0.201	0.739	0.7803738	0.98634934	1	234	tags=27%, lis
GOBP_POSITIVE_REGULATION_OF_BINDING	32	0.151	0.738	0.82793015	0.9866599	1	210	tags=19%, lis
GOMF_PROTEIN_PHOSPHATASE_BINDING	24	0.168	0.737	0.8064516	0.9857436	1	1366	tags=100%, l
GOBP_RNA_LOCALIZATION	41	0.139	0.733	0.84158415	0.99073863	1	1415	tags=100%, l
GOBP_OSTEOBLAST_DIFFERENTIATION	15	0.197	0.731	0.8042453	0.9919936	1	623	tags=67%, lis
GOBP_MEMBRANE_LIPID_METABOLIC_PROCESS	19	0.177	0.728	0.82	0.9934305	1	249	tags=26%, lis
GOBP_ACTIN_FILAMENT_BUNDLE_ORGANIZATION	22	0.169	0.728	0.8142077	0.991985	1	339	tags=32%, lis
GOBP_STEROID_HORMONE_MEDIATED_SIGNALING_PATHWAY	18	0.179	0.726	0.79611653	0.9931349	1	732	tags=78%, lis
GOBP_RESPONSE_TO_RADIATION	51	0.131	0.726	0.85983825	0.9919865	1	173	tags=14%, lis
GOBP_ORGANOPHOSPHATE_BIOSYNTHETIC_PROCESS	63	0.122	0.723	0.8888889	0.9940225	1	77	tags=8%, list
REACTOME_PLATELET_ACTIVATION_SIGNALING_AND_AGGREGATION	44	0.138	0.723	0.8622449	0.9928582	1	142	tags=14%, lis
REACTOME_DISEASES_OF_METABOLISM	18	0.180	0.722	0.7914692	0.99228185	1	356	tags=33%, lis
GOCC_SYNAPSE	93	0.114	0.722	0.89238846	0.99096334	1	176	tags=14%, lis
GOBP_ACTIN_CYTOSKELETON_REORGANIZATION	20	0.173	0.722	0.8070588	0.989602	1	1357	tags=100%, l
REACTOME_SIGNALING_BY_TGFB_FAMILY_MEMBERS	17	0.187	0.721	0.8074074	0.9890126	1	982	tags=88%, lis
GOCC_PRESYNAPSE	38	0.142	0.721	0.83709276	0.9876236	1	129	tags=13%, lis
GOBP_POSITIVE_REGULATION_OF_SECRETION	32	0.150	0.721	0.81796116	0.986969	1	53	tags=6%, list
GOBP_RESPONSE_TO_MOLECULE_OF_BACTERIAL_ORIGIN	49	0.132	0.718	0.865285	0.98936725	1	281	tags=20%, lis
GOBP_REGULATION_OF_PROTEIN_STABILITY	42	0.135	0.716	0.842246	0.9906207	1	371	tags=29%, lis
GOBP_REGULATION_OF_DNA_METABOLIC_PROCESS	39	0.137	0.714	0.8613861	0.9911386	1	178	tags=15%, lis
GOMF_NUCLEAR_RECEPTOR_BINDING	18	0.177	0.714	0.8324873	0.9901032	1	746	tags=78%, lis
GOBP_INTRACELLULAR_STEROID_HORMONE_RECEPTOR_SIGNALING_PATHWAY	18	0.179	0.713	0.81971157	0.9892851	1	732	tags=78%, lis
GOMF_CALCIUM_ION_BINDING	48	0.131	0.713	0.85117495	0.98840827	1	143	tags=10%, lis
GOBP_REGULATION_OF_CELL_CYCLE_PROCESS	94	0.109	0.704	0.93850267	0.99745256	1	312	tags=22%, lis
GOBP_ACTIN_FILAMENT_BASED_PROCESS	99	0.107	0.703	0.9166667	0.9963394	1	382	tags=27%, lis
GOCC_EXTRINSIC_COMPONENT_OF_PLASMA_MEMBRANE	16	0.182	0.703	0.82819384	0.9959015	1	83	tags=13%, lis
GOBP_ANATOMICAL_STRUCTURE_MATURATION	18	0.173	0.702	0.8417266	0.99522364	1	140	tags=17%, lis
GOBP_POSITIVE_REGULATION_OF_PROTEIN_LOCALIZATION_TO_NUCLEUS	17	0.181	0.701	0.8310502	0.99450076	1	152	tags=18%, lis
GOBP_REGULATION_OF_RNA_SPLICING	32	0.148	0.701	0.85532993	0.9933164	1	1399	tags=100%, l
GOCC_SECRETORY_GRANULE	142	0.100	0.700	0.9496021	0.99365056	1	287	tags=19%, lis
GOBP_ESTABLISHMENT_OF_RNA_LOCALIZATION	32	0.146	0.699	0.8502538	0.9930745	1	310	tags=28%, lis
GOMF_TRANSFERASE_ACTIVITY_TRANSFERRING_HEXOSYL_GROUPS	16	0.180	0.695	0.8507853	0.9962996	1	249	tags=25%, lis
REACTOME_SARS_COV_2_INFECTION	17	0.175	0.694	0.8310502	0.9963771	1	234	tags=24%, lis
REACTOME_RAC2_GTPASE_CYCLE	23	0.162	0.691	0.8324873	0.99797225	1	44	tags=9%, list
GOBP_POSITIVE_REGULATION_OF_ACTIN_FILAMENT_POLYMERIZATION	18	0.173	0.688	0.8555305	0.9994391	1	315	tags=28%, lis

GOCC_RUFFLE_MEMBRANE	17	0.173	0.684	0.8516746	1	1	1356	tags=100%, l
KEGG_CHEMOKINE_SIGNALING_PATHWAY	38	0.133	0.683	0.89041096	1	1	148	tags=13%, lis
GOMF_PHOSPHATIDYLINOSITOL_PHOSPHATE_BINDING	19	0.169	0.683	0.8507109	1	1	53	tags=11%, lis
PID_P53_DOWNSTREAM_PATHWAY	20	0.164	0.683	0.8682927	0.9996957	1	299	tags=25%, lis
REACTOME_TRANSCRIPTIONAL_REGULATION_BY_TP53	51	0.126	0.683	0.9055118	0.9984327	1	111	tags=10%, lis
GOBP_REGULATION_OF_SUPRAMOLECULAR_FIBER_ORGANIZATION	58	0.120	0.681	0.9052632	0.99830073	1	211	tags=16%, lis
GOMF_ATPASE_ACTIVITY	52	0.120	0.678	0.9104859	1	1	95	tags=8%, list-
GOCC_MEMBRANE_COAT	20	0.161	0.678	0.8682927	0.99932116	1	1230	tags=95%, lis
GOMF_PEPTIDE_BINDING	30	0.143	0.670	0.8965517	1	1	107	tags=10%, lis
GOBP_REGULATION_OF_ANTIGEN_RECEPTOR_MEDIATED_SIGNALING_PATHWAY	17	0.168	0.669	0.87344915	1	1	996	tags=88%, lis
GOBP_SKELETAL_SYSTEM_DEVELOPMENT	38	0.129	0.669	0.88859415	1	1	343	tags=26%, lis
GOBP_ENDOPLASMIC_RETICULUM_TO_GOLGI_VESICLE_MEDIATED_TRANSPORT	29	0.140	0.668	0.872449	1	1	259	tags=24%, lis
GOBP_PROTEIN_LOCALIZATION_TO_NUCLEUS	37	0.130	0.667	0.8863636	1	1	152	tags=11%, lis
GOBP_NEGATIVE_REGULATION_OF_CATALYTIC_ACTIVITY	102	0.099	0.666	0.9415042	1	1	281	tags=19%, lis
GOBP_POSITIVE_REGULATION_OF_CELLULAR_COMPONENT_BIOGENESIS	77	0.107	0.666	0.9383378	1	1	100	tags=8%, list-
PID_FCER1_PATHWAY	16	0.166	0.662	0.8766067	1	1	173	tags=19%, lis
GOBP_EPITHELIAL_CELL_DIFFERENTIATION	56	0.116	0.661	0.9232673	1	1	221	tags=16%, lis
GOBP_REGULATION_OF_HORMONE_LEVELS	22	0.154	0.661	0.8768844	1	1	203	tags=18%, lis
GOBP_REGULATION_OF_ORGANELLE_ORGANIZATION	166	0.091	0.659	0.9836956	1	1	211	tags=13%, lis
GOMF_STEROID_HORMONE_RECEPTOR_BINDING	16	0.172	0.655	0.8779343	1	1	699	tags=75%, lis
GOBP_REGULATION_OF_MRNA_SPLICING_VIA_SPLICEOSOME	24	0.147	0.655	0.9032258	1	1	1399	tags=100%, l
GOMF_CYSTEINE_TYPE_ENDOPEPTIDASE_ACTIVITY	18	0.162	0.654	0.8944844	1	1	281	tags=28%, lis
GOBP_DOUBLE_STRAND_BREAK_REPAIR	23	0.145	0.654	0.9012658	1	1	115	tags=13%, lis
REACTOME_PARASITE_INFECTION	24	0.147	0.653	0.905569	0.99999535	1	169	tags=17%, lis
GOCC_INTRINSIC_COMPONENT_OF_ORGANELLE_MEMBRANE	39	0.129	0.653	0.91099477	0.99923605	1	170	tags=13%, lis
GOBP_CORTICAL_CYTOSKELETON_ORGANIZATION	15	0.172	0.652	0.886747	0.9987915	1	400	tags=40%, lis
GOBP_MONOSACCHARIDE_METABOLIC_PROCESS	25	0.142	0.651	0.88249403	0.99827635	1	166	tags=16%, lis
GOBP_RESPONSE_TO_OXYGEN_LEVELS	62	0.112	0.647	0.9312977	1	1	392	tags=31%, lis
KEGG_FC_GAMMA_R_MEDIATED_PHAGOCYTOSIS	26	0.139	0.647	0.908642	0.99920404	1	321	tags=27%, lis
GOBP_NUCLEAR_CHROMOSOME_SEGREGATION	26	0.142	0.646	0.89242053	0.99853617	1	945	tags=81%, lis
PID_ERBB1_DOWNSTREAM_PATHWAY	29	0.139	0.645	0.9368932	0.99767435	1	1292	tags=97%, lis
GOBP_PROTEIN_POLYMERIZATION	46	0.122	0.644	0.929471	0.99740714	1	330	tags=26%, lis
GOBP_REGULATION_OF_CELLULAR_COMPONENT_BIOGENESIS	125	0.095	0.643	0.9583333	0.9970507	1	129	tags=9%, list-
GOBP_MRNA_TRANSPORT	22	0.143	0.637	0.8910891	0.9999957	1	1405	tags=100%, l
REACTOME_RESOLUTION_OF_SISTER_CHROMATID_COHESION	18	0.157	0.636	0.89805824	0.9994511	1	233	tags=22%, lis
GOMF_DOUBLE_STRANDED_RNA_BINDING	17	0.161	0.636	0.90841585	0.9985168	1	842	tags=76%, lis
GOBP_CELLULAR_RESPONSE_TO_CHEMICAL_STRESS	49	0.115	0.635	0.9419192	0.99733526	1	324	tags=24%, lis
KEGG_REGULATION_OF_ACTIN_CYTOSKELETON	47	0.117	0.628	0.9393064	1	1	1450	tags=100%, l
GOBP_MESENCHYMAL_CELL_DIFFERENTIATION	21	0.151	0.628	0.9192399	1	1	292	tags=24%, lis
GOBP_RESPONSE_TO_MONOSACCHARIDE	17	0.158	0.628	0.89786226	0.99940366	1	1194	tags=94%, lis
GOBP_DNA_RECOMBINATION	27	0.137	0.627	0.9211196	0.99819064	1	104	tags=11%, lis
REACTOME_RNA_POLYMERASE_II_TRANSCRIPTION_TERMINATION	15	0.168	0.626	0.91415316	0.99819934	1	1364	tags=100%, l
GOBP_CYTOSKELETON_DEPENDENT_INTRACELLULAR_TRANSPORT	20	0.147	0.625	0.9164557	0.9976051	1	371	tags=35%, lis
REACTOME_RESPONSE_TO_ELEVATED_PLATELET_CYTOSOLIC_CA2	20	0.148	0.624	0.912114	0.9966882	1	1272	tags=95%, lis
GOCC_TERTIARY_GRANULE	39	0.119	0.619	0.94513714	0.9987582	1	511	tags=51%, lis
GOBP_RESPONSE_TO_MECHANICAL_STIMULUS	22	0.144	0.616	0.9179487	0.9993008	1	144	tags=14%, lis
HALLMARK_KRAS_SIGNALING_UP	31	0.126	0.615	0.9493671	0.9990513	1	386	tags=32%, lis
HALLMARK_APICAL_JUNCTION	29	0.135	0.614	0.9133858	0.9979308	1	1172	tags=90%, lis
GOBP_NEURON_PROJECTION_GUIDANCE	21	0.145	0.612	0.9070905	0.9982129	1	143	tags=14%, lis
KEGG_LEISHMANIA_INFECTION	24	0.140	0.610	0.93842363	0.9985423	1	241	tags=21%, lis
GOCC_MICROBODY	15	0.159	0.598	0.9513382	1	1	19	tags=7%, list-
REACTOME_RHO_GTPASES_ACTIVATE_WASPS_AND_WAVES	16	0.151	0.595	0.94216865	1	1	337	tags=31%, lis
GOBP_REGULATION_OF_EXTRINSIC_APOPTOTIC_SIGNALING_PATHWAY	18	0.153	0.592	0.93719804	1	1	170	tags=17%, lis
REACTOME_NEURONAL_SYSTEM	23	0.138	0.590	0.9460094	1	1	1414	tags=100%, l
GOBP_CELL_MORPHOGENESIS_INVOLVED_IN_DIFFERENTIATION	77	0.095	0.589	0.96464646	1	1	405	tags=32%, lis
GOCC_ENDOPLASMIC_RETICULUM_GOLGI_INTERMEDIATE_COMPARTMENT	23	0.136	0.586	0.94683546	1	1	324	tags=26%, lis
REACTOME_FCGAMMA_RECEPTOR_FCGR_DEPENDENT_PHAGOCYTOSIS	27	0.128	0.586	0.94962215	1	1	169	tags=15%, lis
REACTOME_LEISHMANIA_INFECTION	40	0.113	0.583	0.969863	1	1	337	tags=28%, lis
GOBP_MESENCHYME_DEVELOPMENT	24	0.128	0.579	0.9468599	1	1	148	tags=13%, lis
GOBP_NEGATIVE_REGULATION_OF_CELLULAR_CATABOLIC_PROCESS	39	0.110	0.570	0.9660574	1	1	235	tags=18%, lis
GOBP_CELLULAR_COMPONENT_DISASSEMBLY	68	0.094	0.567	0.9840425	1	1	401	tags=29%, lis
GOBP_DNA_METABOLIC_PROCESS	94	0.087	0.565	0.9842932	1	1	178	tags=12%, lis
HALLMARK_UV_RESPONSE_DN	20	0.135	0.563	0.9551122	1	1	624	tags=60%, lis
GOBP_PLATELET_DEGRANULATION	16	0.147	0.560	0.9338061	1	1	400	tags=38%, lis
GOMF_UNFOLDED_PROTEIN_BINDING	19	0.137	0.560	0.9536585	1	1	1415	tags=100%, l
GOBP_POSITIVE_REGULATION_OF_PROTEIN_POLYMERIZATION	26	0.121	0.553	0.9594595	1	1	330	tags=27%, lis
REACTOME_PROTEIN_LOCALIZATION	24	0.123	0.552	0.96125907	1	1	414	tags=38%, lis
GOBP_SUPRAMOLECULAR_FIBER_ORGANIZATION	94	0.085	0.548	1	1	1	339	tags=22%, lis
GOBP_RESPONSE_TO_IONIZING_RADIATION	24	0.124	0.545	0.95522386	1	1	104	tags=8%, list-
GOBP_SKELETAL_MUSCLE_ORGAN_DEVELOPMENT	19	0.131	0.542	0.9634146	1	1	127	tags=11%, lis
GOBP_CYTOSOLIC_CALCIUM_ION_TRANSPORT	17	0.134	0.541	0.96891195	1	1	1257	tags=94%, lis
GOBP_CELL_CYCLE_PROCESS	147	0.076	0.536	0.99425286	1	1	312	tags=20%, lis
GOBP_MRNA_EXPORT_FROM_NUCLEUS	15	0.143	0.535	0.97652584	1	1	1405	tags=100%, l
GOBP_CELL_DEATH_IN_RESPONSE_TO_OXIDATIVE_STRESS	15	0.140	0.533	0.9798995	1	1	1410	tags=100%, l
GOBP_NEGATIVE_REGULATION_OF_INTRINSIC_APOPTOTIC_SIGNALING_PATHWAY	17	0.136	0.532	0.9742389	1	1	28	tags=6%, list-
GOCC_TRANSCRIPTION_REGULATOR_COMPLEX	49	0.097	0.532	0.9898219	1	1	179	tags=12%, lis
GOBP_POSITIVE_REGULATION_OF_PROTEIN_CONTAINING_COMPLEX_ASSEMBLY	39	0.102	0.531	0.9748111	1	1	382	tags=31%, lis
GOBP_VIRAL_GENE_EXPRESSION	22	0.122	0.530	0.9754902	1	1	234	tags=18%, lis
GOBP_REGULATION_OF_LEUKOCYTE_MEDIATED_IMMUNITY	32	0.110	0.529	0.992629	0.99961984	1	1131	tags=88%, lis

GOBP_NEGATIVE_REGULATION_OF_NF_KAPPAB_TRANSCRIPTION_FACTOR_ACTIVITY	15	0.141	0.529	0.9768519	0.9984235	1	1407	tags=100%, l
GOMF_PROTEIN_CONTAINING_COMPLEX_BINDING	162	0.073	0.528	1	0.99726856	1	324	tags=20%, lis
PID_HIF1_TFPATHWAY	16	0.135	0.521	0.9722222	0.9982527	1	619	tags=63%, lis
GOBP_REGULATION_OF_CELL_SHAPE	33	0.103	0.507	0.9811765	1	1	1378	tags=97%, lis
KEGG_FOCAL_ADHESION	39	0.098	0.504	0.97761196	1	1	1479	tags=100%, l
GOBP_REGULATION_OF_ORGANELLE_ASSEMBLY	24	0.114	0.501	0.9804878	0.9997576	1	1453	tags=100%, l
KEGG_OOCYTE_MEIOSIS	20	0.122	0.499	0.97943443	0.99896115	1	257	tags=20%, lis
REACTOME_EPH_EPHRIN_SIGNALING	20	0.117	0.488	0.9851852	1	1	959	tags=80%, lis
GOBP_DNA_INTEGRITY_CHECKPOINT	18	0.112	0.459	0.98994976	1	1	1277	tags=94%, lis
GOBP_CELL_CYCLE_CHECKPOINT	23	0.104	0.458	0.9809524	1	1	299	tags=22%, lis
GOBP_MAINTENANCE_OF_CELL_NUMBER	21	0.106	0.454	0.997561	1	1	143	tags=10%, lis
GOBP_SISTER_CHROMATID_SEGREGATION	22	0.102	0.427	0.99271846	1	1	945	tags=77%, lis
GOBP_CHROMOSOME_SEGREGATION	32	0.086	0.426	0.9951338	1	1	191	tags=13%, lis
GOCC_ANCHORING_JUNCTION	109	0.062	0.423	0.9972603	1	1	1508	tags=99%, lis
GOBP_RESPONSE_TO_CARBOHYDRATE	20	0.092	0.374	1	1	1	604	tags=55%, lis
GOBP_ESTABLISHMENT_OF_PROTEIN_LOCALIZATION_TO_MEMBRANE	43	0.069	0.374	1	1	1	110	tags=7%, list=
GOBP_POSITIVE_REGULATION_OF_STRESS_ACTIVATED_PROTEIN_KINASE_SIGNALING_CASCADE	15	0.092	0.346	1	0.9998472	1	1279	tags=93%, lis
