# Supplementary material for: Biodegradable nanoparticles induce cGAS/STING-dependent reprogramming of myeloid cells to promote tumor immunotherapy
Source: Front Immunol. 2022 Aug 18;13:887649. doi: 10.3389/fimmu.2022.887649 (PMC9433741; doi:10.3389/fimmu.2022.887649)
Supplement: Supplementary file 14 [file Table_8.pdf]

Supplemental Table 8. Signaling Pathway Analysis for Neutrophils - 3 Consecutive Doses - ONP-302 vs. Saline

NAME	SIZE	ES	NES	NOM p-val	FDR q-val	FWER p-val	RANK AT MA	LEADING EDG
HALLMARK_INTERFERON_GAMMA_RESPONSE	63	0.696	3.421	0	0	0	124	tags=62%, lis
HALLMARK_INTERFERON_ALPHA_RESPONSE	42	0.737	3.251	0	0	0	180	tags=79%, lis
GOBP_RESPONSE_TO_VIRUS	58	0.654	3.182	0	0	0	111	tags=50%, lis
GOBP_DEFENSE_RESPONSE_TO_VIRUS	47	0.683	3.114	0	0	0	122	tags=57%, lis
GOBP_INNATE_IMMUNE_RESPONSE	110	0.566	3.110	0	0	0	138	tags=45%, lis
GOBP_REGULATION_OF_RESPONSE_TO_BIOTIC_STIMULUS	57	0.632	3.108	0	0	0	137	tags=51%, lis
REACTOME_INTERFERON_SIGNALING	34	0.727	3.107	0	0	0	127	tags=68%, lis
GOBP_DEFENSE_RESPONSE_TO_OTHER_ORGANISM	125	0.544	3.032	0	0	0	138	tags=42%, lis
GOBP_RESPONSE_TO_TYPE_I_INTERFERON	25	0.759	2.963	0	0	0	84	tags=60%, lis
GOBP_RESPONSE_TO_INTERFERON_GAMMA	34	0.682	2.887	0	0	0	127	tags=56%, lis
GOBP_REGULATION_OF_INNATE_IMMUNE_RESPONSE	47	0.621	2.828	0	0	0	137	tags=51%, lis
REACTOME_INTERFERON_ALPHA_BETA_SIGNALING	20	0.769	2.815	0	0	0	84	tags=65%, lis
GOBP_RESPONSE_TO_BIOTIC_STIMULUS	159	0.487	2.795	0	0	0	138	tags=36%, lis
GOBP_INTERFERON_GAMMA_MEDIATED_SIGNALING_PATHWAY	18	0.787	2.780	0	0	0	127	tags=78%, lis
GOBP_DEFENSE_RESPONSE	160	0.474	2.702	0	0	0	151	tags=36%, lis
GOBP_CYTOKINE_MEDIATED_SIGNALING_PATHWAY	90	0.510	2.685	0	0	0	160	tags=42%, lis
GOBP_RESPONSE_TO_CYTOKINE	122	0.488	2.682	0	0	0	154	tags=38%, lis
GOBP_REGULATION_OF_DEFENSE_RESPONSE	74	0.524	2.672	0	0	0	151	tags=42%, lis
HALLMARK_ALLOGRAFT_REJECTION	22	0.706	2.672	0	0	0	164	tags=73%, lis
GOBP_NEGATIVE_REGULATION_OF_VIRAL_PROCESS	18	0.740	2.628	0	8.82E-05	0.002	107	tags=67%, lis
GOBP_REGULATION_OF_IMMUNE_RESPONSE	95	0.497	2.625	0	8.40E-05	0.002	140	tags=38%, lis
GOBP_REGULATION_OF_RESPONSE_TO_CYTOKINE_STIMULUS	28	0.655	2.623	0	8.02E-05	0.002	154	tags=61%, lis
REACTOME_INTERFERON_GAMMA_SIGNALING	15	0.782	2.603	0	7.67E-05	0.002	127	tags=80%, lis
REACTOME_CYTOKINE_SIGNALING_IN_IMMUNE_SYSTEM	82	0.492	2.542	0	1.49E-04	0.004	136	tags=40%, lis
GOBP_POSITIVE_REGULATION_OF_RESPONSE_TO_BIOTIC_STIMULUS	39	0.570	2.534	0	1.43E-04	0.004	137	tags=44%, lis
GOBP_REGULATION_OF_VIRAL_LIFE_CYCLE	18	0.712	2.482	0	1.72E-04	0.005	107	tags=61%, lis
GOBP_REGULATION_OF_IMMUNE_SYSTEM_PROCESS	136	0.439	2.478	0	1.65E-04	0.005	154	tags=35%, lis
GOBP_REGULATION_OF_RESPONSE_TO_EXTERNAL_STIMULUS	94	0.462	2.457	0	2.87E-04	0.009	151	tags=36%, lis
GOBP_REGULATION_OF_BIOLOGICAL_PROCESS_INVOLVED_IN_SYMBIOTIC_INTERACTION	23	0.652	2.428	0	3.70E-04	0.012	107	tags=52%, lis
GOBP_BIOLOGICAL_PROCESS_INVOLVED_IN_SYMBIOTIC_INTERACTION	95	0.450	2.410	0	4.46E-04	0.015	166	tags=38%, lis
GOBP_TYPE_I_INTERFERON_PRODUCTION	20	0.643	2.348	0	0.001199838	0.041	122	tags=55%, lis
GOBP_POSITIVE_REGULATION_OF_IMMUNE_RESPONSE	68	0.465	2.322	0	0.001717467	0.061	140	tags=35%, lis
GOBP_REGULATION_OF_RESPONSE_TO_STRESS	121	0.416	2.302	0	0.002122079	0.078	191	tags=39%, lis
GOBP_ADAPTIVE_IMMUNE_RESPONSE	31	0.537	2.279	0	0.002685738	0.099	138	tags=45%, lis
GOBP_RESPONSE_TO_BACTERIUM	53	0.482	2.270	0	0.002891756	0.11	76	tags=28%, lis
GOBP_POSITIVE_REGULATION_OF_RESPONSE_TO_CYTOKINE_STIMULUS	15	0.669	2.260	0	0.002959874	0.116	134	tags=53%, lis
GOBP_NEGATIVE_REGULATION_OF_IMMUNE_SYSTEM_PROCESS	29	0.548	2.255	0	0.002976018	0.12	149	tags=48%, lis
GOBP_PATTERN_RECOGNITION_RECEPTOR_SIGNALING_PATHWAY	29	0.537	2.237	0	0.003457920	0.141	122	tags=38%, lis
GOBP_POSITIVE_REGULATION_OF_RESPONSE_TO_EXTERNAL_STIMULUS	59	0.447	2.172	0	0.006796359	0.265	151	tags=36%, lis
GOBP_VIRAL_LIFE_CYCLE	33	0.522	2.171	0	0.006626451	0.265	149	tags=45%, lis
GOBP_DEFENSE_RESPONSE_TO_BACTERIUM	22	0.572	2.155	0.001367989	0.007851442	0.309	76	tags=32%, lis
GOBP_POSITIVE_REGULATION_OF_IMMUNE_SYSTEM_PROCESS	94	0.398	2.129	0	0.010398489	0.398	140	tags=32%, lis
GOBP_RESPONSE_TO_TUMOR_NECROSIS_FACTOR	31	0.518	2.121	0.001322751	0.011085019	0.424	124	tags=42%, lis
GOBP_POSITIVE_REGULATION_OF_DEFENSE_RESPONSE	47	0.460	2.090	0.001253132	0.015251222	0.537	151	tags=36%, lis
GOBP_CELLULAR_RESPONSE_TO_MOLECULE_OF_BACTERIAL_ORIGIN	19	0.583	2.078	0.001436781	0.01675029	0.575	44	tags=26%, lis
REACTOME_CLASS_I_MHC_MEDIATED_ANTIGEN_PROCESSING_PRESENTATION	45	0.452	2.036	0.002528445	0.024635319	0.719	130	tags=38%, lis
GOMF_GTPASE_ACTIVITY	24	0.513	2.027	0.008571428	0.026414523	0.759	28	tags=17%, lis
GOBP_CYTOPLASMIC_PATTERN_RECOGNITION_RECEPTOR_SIGNALING_PATHWAY	16	0.588	2.024	0.002936857	0.026509667	0.769	122	tags=44%, lis
GOMF_PROTEIN_HOMODIMERIZATION_ACTIVITY	37	0.472	2.016	0.001310616	0.027742835	0.793	164	tags=41%, lis
GOBP_TUMOR_NECROSIS_FACTOR_MEDIATED_SIGNALING_PATHWAY	22	0.530	2.001	0.002844950	0.030567007	0.826	328	tags=82%, lis
GOCC_PHAGOCYTIC_VESICLE	23	0.519	1.993	0.002706359	0.032246962	0.852	67	tags=26%, lis
REACTOME_ANTIGEN_PROCESSING_CROSS_PRESENTATION	22	0.525	1.981	0.005772005	0.035643175	0.884	73	tags=36%, lis
GOMF_HYDROLASE_ACTIVITY_ACTING_ON_ACID_ANHYDRIDES	53	0.404	1.968	0.002472187	0.03886692	0.909	87	tags=23%, lis
GOBP_CYTOKINE_PRODUCTION	72	0.384	1.963	0.002293577	0.039609667	0.918	176	tags=35%, lis
GOBP_IMMUNE_EFFECTOR_PROCESS	150	0.337	1.923	0	0.054672427	0.967	110	tags=23%, lis
GOBP_RESPONSE_TO_INTERLEUKIN_1	25	0.500	1.918	0.001364256	0.055836428	0.97	124	tags=40%, lis
GOBP_ANTIGEN_PROCESSING_AND_PRESENTATION_OF_EXOGENOUS_PEPTIDE_ANTIGEN_VIA_MH	19	0.544	1.916	0.005822416	0.05593251	0.972	137	tags=47%, lis
GOMF_PROTEIN_DIMERIZATION_ACTIVITY	50	0.402	1.915	0.00375	0.05509128	0.972	168	tags=36%, lis
GOMF_GUANYL_NUCLEOTIDE_BINDING	25	0.498	1.910	0.012517385	0.056590915	0.978	52	tags=20%, lis
GOCC_ENDOCYTIC_VESICLE_MEMBRANE	20	0.514	1.904	0.005797101	0.058513924	0.983	67	tags=25%, lis
GOBP_POSITIVE_REGULATION_OF_CYTOKINE_PRODUCTION	44	0.417	1.893	0.010191083	0.06270104	0.988	17	tags=16%, lis
GOBP_LYMPHOCYTE_MEDIATED_IMMUNITY	16	0.552	1.892	0.004418262	0.0619318	0.988	195	tags=56%, lis
REACTOME_ANTIGEN_PROCESSING_UBIQUITINATION_PROTEASOME_DEGRADATION	32	0.450	1.884	0.01076716	0.065413244	0.993	176	tags=47%, lis
GOBP_ANTIGEN_PROCESSING_AND_PRESENTATION_OF_PEPTIDE_ANTIGEN_VIA_MHC_CLASS_I	21	0.506	1.880	0.005464480	0.06627458	0.996	137	tags=43%, lis
HALLMARK_TNFA_SIGNALING_VIA_NFKB	33	0.441	1.868	0.010943913	0.07139495	0.997	147	tags=45%, lis
GOBP_NEGATIVE_REGULATION_OF_CYTOKINE_PRODUCTION	24	0.478	1.865	0.009485095	0.07197853	0.997	191	tags=50%, lis
GOBP_ANTIGEN_PROCESSING_AND_PRESENTATION_OF_PEPTIDE_ANTIGEN	28	0.464	1.860	0.005434782	0.07359467	0.997	137	tags=39%, lis
GOBP_CELLULAR_RESPONSE_TO_BIOTIC_STIMULUS	25	0.480	1.851	0.009708738	0.07770951	0.999	44	tags=20%, lis
REACTOME_ADAPTIVE_IMMUNE_SYSTEM	75	0.367	1.851	0.002347417	0.0766347	0.999	205	tags=39%, lis
GOBP_ANTIGEN_PROCESSING_AND_PRESENTATION	29	0.452	1.827	0.007002801	0.090269946	0.999	137	tags=38%, lis
GOBP_POST_TRANSLATIONAL_PROTEIN_MODIFICATION	19	0.517	1.819	0.012765957	0.09480641	1	158	tags=42%, lis
GOBP_ADAPTIVE_IMMUNE_RESPONSE_BASED_ON_SOMATIC_RECOMBINATION_OF_IMMUNE_RE	18	0.504	1.798	0.016806724	0.10793294	1	122	tags=39%, lis
GOBP_PROTEIN_POLYUBIQUITINATION	36	0.423	1.797	0.013495277	0.1072813	1	130	tags=36%, lis
GOBP_ACTIVATION_OF_INNATE_IMMUNE_RESPONSE	25	0.465	1.788	0.018387552	0.11240895	1	73	tags=28%, lis
HALLMARK_INFLAMMATORY_RESPONSE	22	0.475	1.787	0.012482663	0.11154677	1	149	tags=41%, lis
GOBP_NEGATIVE_REGULATION_OF_MULTICELLULAR_ORGANISMAL_PROCESS	68	0.353	1.777	0.009456265	0.1192137	1	149	tags=31%, lis

GOBP_IMMUNE_SYSTEM_DEVELOPMENT	84	0.334	1.756	0.012629162	0.13567589	1	140	tags=29%, lis
GOBP_BIOLOGICAL_PROCESS_INVOLVED_IN_INTERACTION_WITH_HOST	21	0.472	1.747	0.033850495	0.1422871	1	136	tags=33%, lis
GOBP_RESPONSE_TO_MOLECULE_OF_BACTERIAL_ORIGIN	30	0.427	1.744	0.018691588	0.14288121	1	67	tags=20%, lis
GOBP_INTRACELLULAR_RECEPTOR_SIGNALING_PATHWAY	32	0.418	1.737	0.024096385	0.14802943	1	140	tags=31%, lis
GOBP_NIK_NF_KAPPAB_SIGNALING	18	0.494	1.720	0.027104137	0.16370875	1	124	tags=44%, lis
GOBP_ACTIVATION_OF_IMMUNE_RESPONSE	46	0.373	1.719	0.019181585	0.16275153	1	140	tags=30%, lis
REACTOME_HOST_INTERACTIONS_OF_HIV_FACTORS	19	0.458	1.711	0.028449502	0.16878	1	164	tags=47%, lis
GOBP_PRODUCTION_OF_MOLECULAR_MEDIATOR_OF_IMMUNE_RESPONSE	15	0.505	1.697	0.033873342	0.18273586	1	149	tags=47%, lis
GOBP_CELLULAR_RESPONSE_TO_LIPID	37	0.394	1.696	0.028947368	0.1825061	1	72	tags=19%, lis
REACTOME_DDX58_IFIH1_MEDIATED_INDUCTION_OF_INTERFERON_ALPHA_BETA	15	0.493	1.680	0.036231883	0.19774044	1	326	tags=80%, lis
REACTOME_TRANSCRIPTIONAL_REGULATION_BY_RUNX2	15	0.494	1.677	0.04040404	0.19980434	1	140	tags=47%, lis
GOBP_I_KAPPAB_KINASE_NF_KAPPAB_SIGNALING	31	0.409	1.655	0.04005525	0.2255798	1	326	tags=68%, lis
GOBP_TOLL_LIKE_RECEPTOR_SIGNALING_PATHWAY	20	0.448	1.652	0.03301238	0.22657192	1	122	tags=35%, lis
REACTOME_SARS_COV_INFECTIONS	17	0.482	1.652	0.034985423	0.22413385	1	256	tags=65%, lis
GOBP_MONONUCLEAR_CELL_DIFFERENTIATION	36	0.384	1.649	0.039267015	0.22624694	1	140	tags=33%, lis
GOBP_PROTEIN_MODIFICATION_BY_SMALL_PROTEIN_CONJUGATION	79	0.319	1.643	0.02199074	0.23151337	1	130	tags=28%, lis
GOBP_PROTEIN_MODIFICATION_BY_SMALL_PROTEIN_CONJUGATION_OR_REMOVAL	98	0.310	1.625	0.012429379	0.25399068	1	146	tags=30%, lis
GOBP_REGULATION_OF_IMMUNE_EFFECTOR_PROCESS	39	0.371	1.617	0.04045512	0.2642767	1	110	tags=26%, lis
GOBP_HOMEOSTASIS_OF_NUMBER_OF_CELLS	19	0.446	1.616	0.060029283	0.26198766	1	171	tags=47%, lis
GOBP_REGULATION_OF_AUTOPHAGY	31	0.392	1.603	0.047425475	0.27964553	1	88	tags=19%, lis
GOBP_REGULATION_OF_RESPONSE_TO_DNA_DAMAGE_STIMULUS	16	0.468	1.602	0.05331412	0.27870908	1	248	tags=63%, lis
GOBP_NEGATIVE_REGULATION_OF_RESPONSE_TO_EXTERNAL_STIMULUS	19	0.453	1.600	0.066187054	0.2787974	1	66	tags=26%, lis
GOMF_IDENTICAL_PROTEIN_BINDING	122	0.284	1.595	0.022826087	0.28433025	1	191	tags=30%, lis
GOBP_NEGATIVE_REGULATION_OF_GENE_EXPRESSION	95	0.303	1.589	0.027272727	0.29108584	1	164	tags=31%, lis
GOBP_REGULATION_OF_DNA_METABOLIC_PROCESS	22	0.422	1.586	0.06524823	0.29270366	1	250	tags=59%, lis
GOMF_TRANSFERASE_ACTIVITY_TRANSFERRING_GLYCOSYL_GROUPS	16	0.460	1.571	0.052554745	0.31378537	1	264	tags=63%, lis
GOBP_INTERLEUKIN_1_MEDIATED_SIGNALING_PATHWAY	16	0.454	1.561	0.07929515	0.32891688	1	73	tags=31%, lis
GOBP_PROTEIN_LOCALIZATION_TO_NUCLEUS	18	0.436	1.553	0.06916427	0.33932877	1	100	tags=22%, lis
GOBP_INNATE_IMMUNE_RESPONSE_ACTIVATING_SIGNAL_TRANSDUCTION	17	0.443	1.551	0.06140351	0.341137	1	416	tags=88%, lis
GOBP_REGULATION_OF_PEPTIDASE_ACTIVITY	33	0.366	1.539	0.06891892	0.35898677	1	94	tags=27%, lis
GOBP_ANTIGEN_RECEPTOR_MEDIATED_SIGNALING_PATHWAY	24	0.398	1.538	0.07765668	0.35765523	1	140	tags=38%, lis
REACTOME_PROGRAMMED_CELL_DEATH	28	0.383	1.537	0.060321715	0.35545328	1	133	tags=36%, lis
GOBP_NEGATIVE_REGULATION_OF_LYMPHOCYTE_ACTIVATION	15	0.459	1.530	0.06715328	0.3656306	1	140	tags=40%, lis
REACTOME_INFECTIOUS_DISEASE	68	0.314	1.530	0.037214886	0.36259773	1	168	tags=32%, lis
GOBP_PROTEIN_MODIFICATION_BY_SMALL_PROTEIN_REMOVAL	37	0.349	1.511	0.07187894	0.395387	1	139	tags=35%, lis
REACTOME_SIGNALING_BY_NOTCH	22	0.399	1.508	0.07909604	0.39805377	1	146	tags=36%, lis
GOCC_EXTERNAL_SIDE_OF_PLASMA_MEMBRANE	17	0.432	1.507	0.078869045	0.39664066	1	76	tags=29%, lis
GOBP_POSITIVE_REGULATION_OF_DNA_METABOLIC_PROCESS	15	0.458	1.505	0.087537095	0.39755592	1	191	tags=53%, lis
GOBP_INFLAMMATORY_RESPONSE	62	0.305	1.504	0.06532066	0.3953942	1	160	tags=24%, lis
GOBP_TISSUE_HOMEOSTASIS	18	0.428	1.500	0.082840234	0.4002701	1	138	tags=44%, lis
REACTOME_DISORDERS_OF_TRANSMEMBRANE_TRANSPORTERS	15	0.449	1.499	0.08643815	0.3977419	1	146	tags=47%, lis
REACTOME_TCR_SIGNALING	15	0.436	1.495	0.09172932	0.4035527	1	124	tags=40%, lis
GOBP_MORPHOGENESIS_OF_A_POLARIZED_EPITHELIUM	15	0.442	1.490	0.081120946	0.41005212	1	124	tags=40%, lis
GOBP_REGULATION_OF_MITOTIC_CELL_CYCLE	33	0.353	1.476	0.08549223	0.4348463	1	133	tags=36%, lis
GOBP_REGULATION_OF_CELL_CYCLE_G2_M_PHASE_TRANSITION	15	0.441	1.475	0.09375	0.43389148	1	277	tags=67%, lis
GOBP_T_CELL_DIFFERENTIATION	18	0.415	1.468	0.09117647	0.4461848	1	87	tags=28%, lis
REACTOME_UCH_PROTEINASES	16	0.428	1.464	0.10622155	0.45098498	1	124	tags=38%, lis
GOMF_KINASE_REGULATOR_ACTIVITY	22	0.386	1.461	0.10836763	0.45376444	1	217	tags=41%, lis
GOBP_CELLULAR_RESPONSE_TO_ABIOTIC_STIMULUS	25	0.362	1.459	0.087417215	0.45324436	1	81	tags=24%, lis
GOMF_ATPASE_ACTIVITY	28	0.363	1.458	0.0855615	0.4513481	1	212	tags=46%, lis
GOBP_NEGATIVE_REGULATION_OF_CELL_DEATH	68	0.288	1.451	0.08372641	0.46324795	1	261	tags=46%, lis
REACTOME_HIV_INFECTION	26	0.370	1.449	0.1042524	0.4649117	1	168	tags=38%, lis
GOBP_FC_EPSILON_RECEPTOR_SIGNALING_PATHWAY	16	0.435	1.448	0.108882524	0.46321067	1	326	tags=69%, lis
GOMF_UBIQUITIN_LIKE_PROTEIN_TRANSFERASE_ACTIVITY	37	0.339	1.444	0.07822686	0.46909162	1	176	tags=38%, lis
GOMF_RIBONUCLEOTIDE_BINDING	107	0.269	1.443	0.05934066	0.4667645	1	145	tags=22%, lis
REACTOME_UB_SPECIFIC_PROCESSING_PROTEASES	27	0.360	1.436	0.13114753	0.47916394	1	124	tags=33%, lis
GOBP_TISSUE_MORPHOGENESIS	33	0.342	1.436	0.104395606	0.47634047	1	143	tags=33%, lis
GOMF_ENDOPEPTIDASE_ACTIVITY	24	0.365	1.435	0.102920726	0.47423065	1	351	tags=71%, lis
REACTOME_C_TYPE_LECTIN_RECEPTORS_CLRS	21	0.385	1.433	0.12083333	0.47613406	1	326	tags=67%, lis
GOBP_LEUKOCYTE_DIFFERENTIATION	48	0.306	1.432	0.107365794	0.47315437	1	140	tags=27%, lis
GOBP_NEGATIVE_REGULATION_OF_CELL_ACTIVATION	18	0.413	1.430	0.13247864	0.47435796	1	140	tags=33%, lis
GOCC_ENDOCYTIC_VESICLE	34	0.334	1.429	0.10344828	0.4728692	1	67	tags=18%, lis
GOBP_REGULATION_OF_HEMOPOIESIS	37	0.323	1.426	0.1131579	0.47641337	1	151	tags=30%, lis
GOBP_NEGATIVE_REGULATION_OF_RESPONSE_TO_STIMULUS	111	0.262	1.424	0.072928175	0.47810012	1	135	tags=23%, lis
GOBP_REGULATION_OF_CATABOLIC_PROCESS	88	0.268	1.420	0.08314351	0.48312607	1	136	tags=23%, lis
GOBP_CELL_CYCLE_G1_S_PHASE_TRANSITION	18	0.398	1.418	0.11806543	0.48517123	1	144	tags=44%, lis
GOBP_CELL_CYCLE_G2_M_PHASE_TRANSITION	16	0.406	1.415	0.12650603	0.48646396	1	196	tags=50%, lis
REACTOME_DEGRADATION_OF_BETA_CATENIN_BY_THE_DESTRUCTION_COMPLEX	15	0.431	1.415	0.12166172	0.4834922	1	133	tags=40%, lis
GOBP_NEGATIVE_REGULATION_OF_CELL_CYCLE_PHASE_TRANSITION	18	0.403	1.410	0.13146234	0.49123082	1	133	tags=39%, lis
GOBP_NEGATIVE_REGULATION_OF_BINDING	16	0.418	1.405	0.1381295	0.5007505	1	67	tags=25%, lis
GOBP_LYMPHOCYTE_ACTIVATION	60	0.290	1.405	0.108591884	0.497356	1	149	tags=30%, lis
GOBP_CELLULAR_PROTEIN_CATABOLIC_PROCESS	71	0.278	1.404	0.08490566	0.49595904	1	176	tags=32%, lis
GOCC_GOLGI_APPARATUS_SUBCOMPARTMENT	52	0.296	1.403	0.1084788	0.4947753	1	34	tags=8%, list-
REACTOME_HEDGEHOG_OFF_STATE	15	0.417	1.400	0.12519084	0.49799535	1	124	tags=40%, lis
GOBP_T_CELL_ACTIVATION	34	0.331	1.399	0.13227513	0.49676624	1	140	tags=32%, lis
GOCC_NUCLEOLUS	47	0.304	1.399	0.12484395	0.4939546	1	95	tags=19%, lis
GOBP_POSITIVE_REGULATION_OF_I_KAPPAB_KINASE_NF_KAPPAB_SIGNALING	17	0.395	1.396	0.13467048	0.49585485	1	326	tags=71%, lis
GOCC_SIDE_OF_MEMBRANE	36	0.324	1.380	0.13642384	0.5278024	1	93	tags=22%, lis
HALLMARK_APOPTOSIS	25	0.352	1.377	0.14265335	0.5319202	1	95	tags=28%, lis

GOBP_REGULATION_OF_GENE_EXPRESSION_EPIGENETIC	16	0.398	1.377	0.1438849	0.5298576	1	105	tags=25%, lis
GOBP_CALCIUM_MEDIATED_SIGNALING	19	0.388	1.377	0.14161849	0.52660024	1	2	tags=5%, list-
GOBP_REGULATION_OF_CELL_CYCLE_PHASE_TRANSITION	28	0.338	1.370	0.14919356	0.5371399	1	133	tags=36%, lis
REACTOME_BETA_CATENIN_INDEPENDENT_WNT_SIGNALING	16	0.408	1.369	0.1420205	0.5384092	1	124	tags=38%, lis
GOBP_REGULATION_OF_MYELOID_CELL_DIFFERENTIATION	24	0.358	1.368	0.15238096	0.5373792	1	151	tags=33%, lis
GOBP_POSITIVE_REGULATION_OF_CATABOLIC_PROCESS	44	0.308	1.366	0.13544303	0.53758085	1	133	tags=25%, lis
GOBP_MORPHOGENESIS_OF_AN_EPITHELIUM	29	0.336	1.366	0.14498645	0.5344041	1	143	tags=31%, lis
GOBP_FC_RECEPTOR_SIGNALING_PATHWAY	22	0.367	1.366	0.13091922	0.5316604	1	124	tags=32%, lis
GOBP_REGULATION_OF_PROTEIN_MODIFICATION_BY_SMALL_PROTEIN_CONJUGATION_OR_REMO	25	0.352	1.366	0.14738292	0.5286882	1	127	tags=28%, lis
GOBP_NEGATIVE_REGULATION_OF_CELL_CYCLE_PROCESS	23	0.355	1.365	0.1392405	0.52694243	1	196	tags=43%, lis
GOBP_T_CELL_RECEPTOR_SIGNALING_PATHWAY	18	0.380	1.359	0.17030568	0.53612876	1	335	tags=67%, lis
GOMF_TRANSCRIPTION_FACTOR_BINDING	44	0.299	1.358	0.13572343	0.5351913	1	171	tags=30%, lis
GOBP_POSITIVE_REGULATION_OF_MULTICELLULAR_ORGANISMAL_PROCESS	98	0.257	1.358	0.11187214	0.533467	1	140	tags=21%, lis
GOBP_PROTEIN_CATABOLIC_PROCESS	79	0.265	1.352	0.11622555	0.5422275	1	176	tags=32%, lis
GOBP_POSITIVE_REGULATION_OF_GENE_EXPRESSION	82	0.264	1.350	0.12847222	0.5434231	1	17	tags=9%, list-
GOBP_RESPONSE_TO_LIPID	54	0.284	1.349	0.14681107	0.54335487	1	72	tags=13%, lis
GOBP_CELL_CYCLE_PHASE_TRANSITION	34	0.315	1.348	0.14942528	0.5420458	1	164	tags=38%, lis
GOBP_REGULATION_OF_CYSTEINE_TYPE_ENDOPEPTIDASE_ACTIVITY	24	0.345	1.342	0.1587517	0.55257994	1	94	tags=25%, lis
GOBP_MYELOID_CELL_DIFFERENTIATION	36	0.315	1.341	0.1630847	0.55216706	1	171	tags=31%, lis
GOBP_REGULATION_OF_CELLULAR_CATABOLIC_PROCESS	79	0.257	1.340	0.1244186	0.54985714	1	130	tags=20%, lis
GOBP_RESPONSE_TO_TEMPERATURE_STIMULUS	17	0.386	1.337	0.15080526	0.55393773	1	82	tags=29%, lis
GOMF_RNA_BINDING	125	0.238	1.333	0.11803278	0.5597457	1	202	tags=30%, lis
GOBP_POSITIVE_REGULATION_OF_PEPTIDASE_ACTIVITY	19	0.368	1.326	0.1814346	0.5724537	1	94	tags=26%, lis
GOBP_CELLULAR_MACROMOLECULE_CATABOLIC_PROCESS	96	0.248	1.325	0.13828571	0.5716577	1	176	tags=30%, lis
GOCC_GOLGI_APPARATUS	86	0.253	1.323	0.12788019	0.5724133	1	72	tags=10%, lis
GOBP_NEGATIVE_REGULATION_OF_CELL_ADHESION	16	0.387	1.316	0.18740849	0.5860495	1	140	tags=31%, lis
REACTOME_FC_EPSILON_RECEPTOR_FCERI_SIGNALING	19	0.358	1.312	0.18867925	0.5918716	1	326	tags=63%, lis
REACTOME_DEUBIQUITINATION	38	0.296	1.306	0.17889318	0.60245955	1	133	tags=29%, lis
GOBP_NEGATIVE_REGULATION_OF_WNT_SIGNALING_PATHWAY	25	0.332	1.305	0.16757493	0.60238963	1	133	tags=32%, lis
GOBP_NEGATIVE_REGULATION_OF_CELL_POPULATION_PROLIFERATION	52	0.275	1.303	0.18237454	0.6034963	1	114	tags=19%, lis
GOBP_NEGATIVE_REGULATION_OF_MITOTIC_CELL_CYCLE	20	0.356	1.303	0.19040902	0.60064805	1	133	tags=35%, lis
REACTOME_DOWNSTREAM_SIGNALING_EVENTS_OF_B_CELL_RECEPTOR_BCR	15	0.385	1.293	0.2042042	0.61968565	1	326	tags=67%, lis
GOBP_CELLULAR_RESPONSE_TO_DNA_DAMAGE_STIMULUS	62	0.264	1.292	0.1575179	0.6198493	1	179	tags=31%, lis
REACTOME_HEDGEHOG_ON_STATE	15	0.379	1.288	0.19389313	0.6249456	1	124	tags=33%, lis
GOMF_HISTONE_BINDING	17	0.361	1.286	0.20058565	0.6261415	1	166	tags=35%, lis
GOBP_REGULATION_OF_PROTEIN_STABILITY	19	0.360	1.286	0.2103064	0.62297463	1	2	tags=5%, list-
GOBP_NEGATIVE_REGULATION_OF_DEVELOPMENTAL_PROCESS	55	0.266	1.285	0.18137848	0.62265176	1	108	tags=22%, lis
GOMF_CYSTEINE_TYPE_PEPTIDASE_ACTIVITY	16	0.382	1.284	0.20494185	0.62117326	1	351	tags=75%, lis
GOBP_ORGANONITROGEN_COMPOUND_CATABOLIC_PROCESS	95	0.238	1.276	0.17893545	0.6376431	1	176	tags=29%, lis
GOBP_PROTEASOMAL_PROTEIN_CATABOLIC_PROCESS	36	0.291	1.274	0.19762845	0.6375477	1	158	tags=33%, lis
REACTOME_CLEC7A_DECTIN_1_SIGNALING	15	0.388	1.274	0.22518519	0.635635	1	326	tags=67%, lis
GOBP_REGULATION_OF_CELLULAR_RESPONSE_TO_STRESS	49	0.275	1.273	0.18518518	0.6351631	1	265	tags=45%, lis
GOBP_MODIFICATION_DEPENDENT_MACROMOLECULE_CATABOLIC_PROCESS	63	0.260	1.268	0.21863799	0.64236176	1	176	tags=32%, lis
REACTOME_TCF_DEPENDENT_SIGNALING_IN_RESPONSE_TO_WNT	19	0.350	1.263	0.21176471	0.65262634	1	133	tags=32%, lis
GOBP_STEM_CELL_DIFFERENTIATION	15	0.379	1.260	0.22238372	0.65572476	1	140	tags=40%, lis
GOMF_PEPTIDASE_ACTIVITY	35	0.293	1.253	0.21784776	0.6705107	1	351	tags=63%, lis
REACTOME_SIGNALING_BY_ROBO_RECEPTORS	15	0.374	1.252	0.22974963	0.66852486	1	124	tags=33%, lis
GOBP_CELLULAR_RESPONSE_TO_EXTERNAL_STIMULUS	21	0.342	1.251	0.21745351	0.6671085	1	72	tags=19%, lis
GOBP_POSITIVE_REGULATION_OF_SIGNALING	130	0.229	1.251	0.17458564	0.6657608	1	149	tags=22%, lis
GOBP_NEGATIVE_REGULATION_OF_SIGNALING	103	0.235	1.249	0.1805869	0.6674129	1	135	tags=22%, lis
GOBP_PEPTIDYL_SERINE_MODIFICATION	23	0.324	1.248	0.23731138	0.6665044	1	2	tags=4%, list-
GOBP_HEMATOPOIETIC_PROGENITOR_CELL_DIFFERENTIATION	16	0.366	1.245	0.22551928	0.669233	1	140	tags=38%, lis
GOBP_NEGATIVE_REGULATION_OF_MOLECULAR_FUNCTION	78	0.248	1.241	0.21835075	0.6755386	1	100	tags=19%, lis
REACTOME_APOPTOSIS	25	0.315	1.240	0.24200279	0.67604935	1	133	tags=32%, lis
GOBP_REGULATION_OF_BINDING	35	0.283	1.238	0.2435724	0.6766267	1	100	tags=20%, lis
GOBP_PROTEIN_CONTAINING_COMPLEX_DISASSEMBLY	22	0.333	1.237	0.2398374	0.6746894	1	2	tags=5%, list-
GOBP_REGULATION_OF_PROTEOLYSIS	53	0.258	1.234	0.22699386	0.67874634	1	149	tags=26%, lis
GOBP_REGULATION_OF_PROTEIN_CONTAINING_COMPLEX_ASSEMBLY	32	0.298	1.234	0.23866667	0.67730343	1	14	tags=6%, list-
GOBP_PROTEOLYSIS	123	0.222	1.233	0.20022124	0.67524934	1	158	tags=25%, lis
GOBP_SECOND_MESSENGER_MEDIATED_SIGNALING	25	0.315	1.228	0.24523161	0.68576455	1	2	tags=4%, list-
GOBP_SIGNAL_TRANSDUCTION_BY_P53_CLASS_MEDIATOR	16	0.360	1.227	0.25726745	0.68345547	1	88	tags=25%, lis
GOBP_MACROMOLECULE_CATABOLIC_PROCESS	107	0.227	1.225	0.20022246	0.68539494	1	176	tags=29%, lis
GOBP_MYELOID_LEUKOCYTE_DIFFERENTIATION	20	0.337	1.222	0.2382311	0.6889273	1	140	tags=30%, lis
KEGG_PATHWAYS_IN_CANCER	29	0.294	1.221	0.25344354	0.6899526	1	151	tags=31%, lis
GOBP_REGULATION_OF_VASCULATURE_DEVELOPMENT	21	0.340	1.218	0.25134408	0.6925662	1	205	tags=48%, lis
REACTOME_SIGNALING_BY_HEDGEHOG	20	0.334	1.213	0.27	0.7023116	1	146	tags=35%, lis
GOBP_RNA_CATABOLIC_PROCESS	33	0.289	1.209	0.25896415	0.7084375	1	164	tags=33%, lis
GOBP_NEGATIVE_REGULATION_OF_APOPTOTIC_SIGNALING_PATHWAY	19	0.334	1.209	0.26368877	0.70683604	1	275	tags=58%, lis
GOBP_CHROMATIN_REMODELING	17	0.352	1.208	0.27705628	0.7040171	1	398	tags=76%, lis
HALLMARK_COMPLEMENT	20	0.330	1.208	0.2752809	0.70293164	1	132	tags=30%, lis
GOBP_REGULATION_OF_CELLULAR_KETONE_METABOLIC_PROCESS	18	0.346	1.207	0.26509574	0.701443	1	73	tags=28%, lis
REACTOME_TRANSCRIPTIONAL_REGULATION_BY_RUNX3	17	0.347	1.203	0.26811594	0.7085142	1	140	tags=35%, lis
GOBP_IMMUNE_RESPONSE_REGULATING_SIGNALING_PATHWAY	41	0.276	1.198	0.27139366	0.7157763	1	426	tags=71%, lis
GOBP_PROCESS_UTILIZING_AUTOPHAGIC_MECHANISM	48	0.260	1.195	0.26918238	0.7214239	1	88	tags=13%, lis
REACTOME_INTERLEUKIN_1_FAMILY_SIGNALING	16	0.357	1.195	0.27311522	0.7186196	1	124	tags=31%, lis
GOMF_KINASE_BINDING	58	0.245	1.193	0.2449465	0.7198582	1	136	tags=22%, lis
GOMF_ENZYME_INHIBITOR_ACTIVITY	27	0.300	1.190	0.260631	0.723399	1	164	tags=33%, lis
REACTOME_CELL_CYCLE_CHECKPOINTS	16	0.347	1.190	0.27988338	0.7203895	1	124	tags=31%, lis
REACTOME_TRANSCRIPTIONAL_REGULATION_BY_RUNX1	21	0.324	1.185	0.26911315	0.7294764	1	140	tags=33%, lis

GOBP_RNA_EXPORT_FROM_NUCLEUS	22	0.308	1.183	0.27260083	0.7309044	1	397	tags=73%, lis
GOBP_REGULATION_OF_DNA_TEMPLATED_TRANSCRIPTION_IN_RESPONSE_TO_STRESS	15	0.360	1.183	0.31295487	0.7278375	1	124	tags=33%, lis
GOBP_REGULATION_OF_CELLULAR_COMPONENT_BIOGENESIS	57	0.250	1.181	0.2766467	0.73018855	1	105	tags=16%, lis
GOBP_REGULATION_OF_HYDROLASE_ACTIVITY	80	0.230	1.181	0.26248547	0.7273219	1	136	tags=23%, lis
REACTOME_SIGNALING_BY_THE_B_CELL_RECEPTOR_BCR	18	0.331	1.180	0.28759125	0.7254125	1	329	tags=61%, lis
GOCC_INTRINSIC_COMPONENT_OF_ENDOPLASMIC_RETICULUM_MEMBRANE	15	0.352	1.179	0.26872247	0.72479486	1	67	tags=20%, lis
GOMF_UBIQUITIN_LIKE_PROTEIN_LIGASE_ACTIVITY	22	0.320	1.179	0.28781793	0.7218612	1	130	tags=32%, lis
REACTOME_NEDDYLATION	17	0.342	1.177	0.29013255	0.72444636	1	158	tags=35%, lis
GOMF_ENZYME_ACTIVATOR_ACTIVITY	40	0.268	1.177	0.281602	0.7215892	1	80	tags=13%, lis
GOBP_POSITIVE_REGULATION_OF_PROTEOLYSIS	31	0.282	1.176	0.2805851	0.720697	1	130	tags=26%, lis
GOBP_ESTABLISHMENT_OF_RNA_LOCALIZATION	23	0.316	1.175	0.3	0.72000545	1	397	tags=74%, lis
GOBP_REGULATION_OF_PROTEIN_BINDING	16	0.338	1.169	0.28654125	0.7302808	1	88	tags=25%, lis
GOBP_NEGATIVE_REGULATION_OF_CELL_CYCLE	37	0.267	1.168	0.28403142	0.7302826	1	248	tags=43%, lis
GOBP_APOPTOTIC_PROCESS	149	0.205	1.165	0.2790445	0.7338084	1	191	tags=26%, lis
GOBP_DNA_METABOLIC_PROCESS	56	0.242	1.164	0.2809406	0.73360026	1	261	tags=41%, lis
GOBP_FAT_CELL_DIFFERENTIATION	18	0.328	1.160	0.31149927	0.7387137	1	127	tags=33%, lis
GOBP_REGULATION_OF_CELL_DIFFERENTIATION	106	0.213	1.158	0.28539577	0.74117416	1	140	tags=23%, lis
GOBP_POSITIVE_REGULATION_OF_PROTEIN_METABOLIC_PROCESS	110	0.215	1.155	0.27716187	0.7449995	1	135	tags=20%, lis
GOBP_NEGATIVE_REGULATION_OF_CANONICAL_WNT_SIGNALING_PATHWAY	24	0.299	1.153	0.29737207	0.7460584	1	133	tags=29%, lis
GOMF_SIGNALING_RECEPTOR_BINDING	91	0.218	1.151	0.29625425	0.7484069	1	138	tags=21%, lis
GOBP_POSITIVE_REGULATION_OF_WNT_SIGNALING_PATHWAY	17	0.333	1.151	0.2934473	0.74603564	1	124	tags=29%, lis
GOBP_REGULATION_OF_CELL_CYCLE_PROCESS	46	0.254	1.149	0.31835207	0.74784017	1	133	tags=28%, lis
GOBP_NEGATIVE_REGULATION_OF_CELL_DIFFERENTIATION	44	0.254	1.148	0.30955413	0.74642396	1	108	tags=20%, lis
GOBP_MULTICELLULAR_ORGANISMAL_HOMEOSTASIS	37	0.262	1.147	0.31406045	0.74621844	1	138	tags=30%, lis
GOBP_ORGANIC_CYCLIC_COMPOUND_CATABOLIC_PROCESS	44	0.254	1.147	0.3149808	0.7436785	1	164	tags=30%, lis
GOBP_REGULATION_OF_LYMPHOCYTE_ACTIVATION	39	0.259	1.146	0.31084657	0.742198	1	114	tags=23%, lis
GOBP_MACROAUTOPHAGY	27	0.285	1.145	0.308311	0.7418485	1	81	tags=11%, lis
GOBP_REGULATION_OF_ANATOMICAL_STRUCTURE_MORPHOGENESIS	65	0.228	1.143	0.2872596	0.7447565	1	151	tags=26%, lis
GOBP_POSITIVE_REGULATION_OF_MOLECULAR_FUNCTION	118	0.206	1.142	0.28985506	0.74378926	1	136	tags=19%, lis
GOBP_DNA_REPAIR	37	0.268	1.139	0.3124183	0.7478132	1	100	tags=22%, lis
GOMF_DNA_BINDING_TRANSCRIPTION_FACTOR_ACTIVITY	48	0.246	1.139	0.30958232	0.745373	1	184	tags=29%, lis
GOCC_ORGANELLE_SUBCOMPARTMENT	101	0.211	1.135	0.3011111	0.7522055	1	16	tags=5%, list=
GOMF_ENZYME_REGULATOR_ACTIVITY	85	0.219	1.131	0.31034482	0.7577551	1	164	tags=24%, lis
GOBP_B_CELL_ACTIVATION	27	0.282	1.130	0.32384825	0.7566825	1	382	tags=67%, lis
GOMF_PROTEIN_DOMAIN_SPECIFIC_BINDING	60	0.228	1.129	0.3112745	0.758339	1	241	tags=35%, lis
GOBP_CELL_POPULATION_PROLIFERATION	124	0.201	1.126	0.3131202	0.7602197	1	151	tags=22%, lis
GOMF_UBIQUITIN_LIKE_PROTEIN_LIGASE_BINDING	39	0.254	1.125	0.32948717	0.7610393	1	268	tags=44%, lis
GOBP_REGULATION_OF_CELL_CYCLE	72	0.226	1.125	0.32332563	0.7587897	1	179	tags=31%, lis
GOBP_PROTEIN_PHOSPHOPANTETHEINYLATION	21	0.309	1.123	0.34405595	0.7604708	1	351	tags=67%, lis
REACTOME_TRANSPORT_OF_MATURE_TRANSCRIPT_TO_CYTOPLASM	15	0.334	1.123	0.34179103	0.7582615	1	379	tags=73%, lis
GOBP_CELLULAR_KETONE_METABOLIC_PROCESS	20	0.310	1.122	0.33572453	0.75595826	1	73	tags=25%, lis
GOBP_RESPONSE_TO_ABIOTIC_STIMULUS	88	0.215	1.118	0.32690126	0.7636241	1	93	tags=17%, lis
GOBP_DNA_BIOSYNTHETIC_PROCESS	16	0.321	1.118	0.34532374	0.7610692	1	191	tags=38%, lis
REACTOME_SIGNALING_BY_WNT	26	0.282	1.115	0.35752687	0.7642277	1	133	tags=27%, lis
REACTOME_RNA_POLYMERASE_II_TRANSCRIPTION	84	0.214	1.113	0.33760187	0.7651772	1	257	tags=39%, lis
GOBP_NUCLEAR_EXPORT	27	0.284	1.112	0.36510068	0.76630235	1	397	tags=70%, lis
GOMF_RNA_POLYMERASE_II_SPECIFIC_DNA_BINDING_TRANSCRIPTION_FACTOR_BINDING	25	0.286	1.112	0.3552812	0.76371187	1	168	tags=28%, lis
GOMF_NUCLEAR_HORMONE_RECEPTOR_BINDING	16	0.334	1.109	0.3638889	0.76683515	1	84	tags=19%, lis
GOBP_REGULATION_OF_LYMPHOCYTE_DIFFERENTIATION	17	0.325	1.109	0.34632683	0.7645926	1	140	tags=29%, lis
GOBP_REGULATION_OF_CELL_DEATH	124	0.201	1.108	0.32603937	0.76413625	1	261	tags=37%, lis
GOBP_LEUKOCYTE_PROLIFERATION	26	0.277	1.108	0.34788734	0.76185894	1	149	tags=27%, lis
GOBP_REGULATION_OF_CELLULAR_PROTEIN_CATABOLIC_PROCESS	20	0.305	1.106	0.35643566	0.7625241	1	328	tags=60%, lis
GOBP_REGULATION_OF_LEUKOCYTE_DIFFERENTIATION	25	0.277	1.105	0.34828496	0.7615576	1	140	tags=28%, lis
GOBP_NEGATIVE_REGULATION_OF_PROTEIN_MODIFICATION_PROCESS	52	0.237	1.105	0.35555556	0.76035744	1	135	tags=21%, lis
GOBP_NUCLEOBASE_CONTAINING_COMPOUND_TRANSPORT	27	0.275	1.104	0.35444745	0.7599403	1	255	tags=48%, lis
GOBP_LEUKOCYTE_CELL_CELL_ADHESION	33	0.265	1.101	0.37157756	0.7638135	1	151	tags=30%, lis
GOCC_CELL_SURFACE	37	0.260	1.101	0.36491677	0.7615516	1	200	tags=32%, lis
GOBP_PROTEIN_PROCESSING	15	0.325	1.098	0.35407406	0.76448524	1	29	tags=13%, lis
GOBP_REGULATION_OF_DNA_BINDING_TRANSCRIPTION_FACTOR_ACTIVITY	33	0.263	1.096	0.36604774	0.7658344	1	74	tags=18%, lis
GOBP_CELLULAR_AMINO_ACID_METABOLIC_PROCESS	20	0.297	1.096	0.37762237	0.7634355	1	190	tags=40%, lis
HALLMARK_P53_PATHWAY	19	0.307	1.096	0.3649635	0.76141685	1	130	tags=32%, lis
GOCC_PERINUCLEAR_REGION_OF_CYTOPLASM	49	0.238	1.094	0.3799505	0.764268	1	43	tags=8%, list=
GOBP_COGNITION	16	0.322	1.091	0.37702504	0.7684996	1	163	tags=38%, lis
REACTOME_INNATE_IMMUNE_SYSTEM	127	0.194	1.088	0.35986915	0.7706687	1	74	tags=13%, lis
GOBP_REGULATION_OF_LEUKOCYTE_PROLIFERATION	23	0.279	1.085	0.39305556	0.7762411	1	149	tags=26%, lis
GOMF_TRANSCRIPTION_REGULATOR_ACTIVITY	98	0.202	1.084	0.37246048	0.7749373	1	184	tags=26%, lis
GOBP_POSITIVE_REGULATION_OF_GTPASE_ACTIVITY	23	0.284	1.076	0.36076817	0.7900284	1	513	tags=87%, lis
GOBP_REGULATION_OF_MRNA_CATABOLIC_PROCESS	25	0.279	1.075	0.38493723	0.7901836	1	124	tags=28%, lis
REACTOME_TOLL_LIKE_RECEPTOR_CASCADES	18	0.303	1.074	0.38686132	0.7910244	1	122	tags=28%, lis
GOBP_REGULATION_OF_GTPASE_ACTIVITY	27	0.269	1.072	0.36997318	0.7912236	1	513	tags=85%, lis
REACTOME_METABOLISM_OF_AMINO_ACIDS_AND_DERIVATIVES	17	0.308	1.072	0.37151247	0.7903574	1	190	tags=41%, lis
GOMF_HORMONE_RECEPTOR_BINDING	17	0.309	1.066	0.39442816	0.8004327	1	84	tags=18%, lis
REACTOME_SEPARATION_OF_SISTER_CHROMATIDS	17	0.306	1.063	0.38709676	0.80446076	1	124	tags=29%, lis
GOBP_MICROTUBULE_CYTOSKELETON_ORGANIZATION	25	0.278	1.062	0.39888424	0.80448794	1	198	tags=40%, lis
GOBP_POSITIVE_REGULATION_OF_BINDING	16	0.314	1.059	0.407355	0.8075379	1	182	tags=38%, lis
GOBP_DOUBLE_STRAND_BREAK_REPAIR	15	0.320	1.058	0.35347432	0.80813897	1	100	tags=27%, lis
HALLMARK_MYC_TARGETS_V1	19	0.297	1.057	0.388102	0.80593675	1	397	tags=79%, lis
GOMF_CYTOKINE_RECEPTOR_BINDING	15	0.317	1.055	0.4092219	0.8090025	1	134	tags=27%, lis
REACTOME_MAPK_FAMILY_SIGNALING_CASCADES	24	0.277	1.055	0.42318437	0.80647135	1	191	tags=33%, lis

REACTOME_INTRACELLULAR_SIGNALING_BY_SECOND_MESSENGERS	22	0.281	1.053	0.414966	0.807269	1	124	tags=27%, lis
GOBP_REGULATION_OF_INFLAMMATORY_RESPONSE	31	0.255	1.052	0.40662253	0.8074829	1	151	tags=26%, lis
GOCC_SPLICEOSOMAL_COMPLEX	16	0.309	1.052	0.40263543	0.80572087	1	179	tags=38%, lis
GOMF_HYDROLASE_ACTIVITY_ACTING_ON_ESTER_BONDS	20	0.281	1.047	0.4188406	0.8127376	1	108	tags=25%, lis
GOBP_MITOTIC_CELL_CYCLE	60	0.215	1.044	0.43878788	0.8162476	1	277	tags=45%, lis
GOBP_POSITIVE_REGULATION_OF_PROTEIN_SERINE_THREONINE_KINASE_ACTIVITY	29	0.258	1.043	0.40730718	0.81646216	1	2	tags=3%, list-
GOBP_POSITIVE_REGULATION_OF_PROTEIN_MODIFICATION_PROCESS	70	0.205	1.040	0.41028708	0.8213772	1	135	tags=19%, lis
GOBP_RNA_LOCALIZATION	26	0.261	1.039	0.4180328	0.8200808	1	397	tags=69%, lis
GOMF_CIS_REGULATORY_REGION_SEQUENCE_SPECIFIC_DNA_BINDING	51	0.218	1.037	0.42177722	0.8223044	1	181	tags=25%, lis
GOMF_ZINC_ION_BINDING	49	0.221	1.036	0.4241677	0.821098	1	127	tags=22%, lis
GOBP_REGULATION_OF_WNT_SIGNALING_PATHWAY	35	0.242	1.034	0.43297586	0.82282823	1	140	tags=26%, lis
GOBP_AMEBOIDAL_TYPE_CELL_MIGRATION	24	0.267	1.032	0.41747573	0.82515484	1	151	tags=33%, lis
GOBP_CELLULAR_COMPONENT_DISASSEMBLY	39	0.233	1.030	0.43401015	0.828298	1	29	tags=5%, list-
GOMF_MOLECULAR_TRANSDUCER_ACTIVITY	33	0.246	1.025	0.43341708	0.8353212	1	339	tags=52%, lis
GOBP_ANIMAL_ORGAN_MORPHOGENESIS	47	0.225	1.021	0.4534005	0.84163177	1	143	tags=23%, lis
GOBP_REGULATION_OF_T_CELL_ACTIVATION	26	0.261	1.020	0.44836956	0.84223354	1	140	tags=27%, lis
GOBP_PROTEIN_MATURATION	18	0.290	1.018	0.44714287	0.8425436	1	29	tags=11%, lis
GOBP_NEGATIVE_REGULATION_OF_HYDROLASE_ACTIVITY	24	0.264	1.016	0.4451962	0.8450118	1	191	tags=29%, lis
GOMF_NUCLEOSIDE_TRIPHOSPHATASE_REGULATOR_ACTIVITY	28	0.245	1.014	0.46206897	0.8456154	1	513	tags=82%, lis
HALLMARK_KRAS_SIGNALING_UP	18	0.292	1.014	0.45123726	0.8439466	1	182	tags=39%, lis
GOCC_NUCLEAR_OUTER_MEMBRANE_ENDOPLASMIC_RETICULUM_MEMBRANE_NETWORK	67	0.203	1.011	0.46457607	0.84773093	1	70	tags=9%, list-
GOCC_RECYCLING_ENDOSOME	17	0.296	1.010	0.43993995	0.8472746	1	319	tags=53%, lis
REACTOME_SIGNALING_BY_INTERLEUKINS	49	0.217	1.005	0.47361964	0.85601985	1	136	tags=22%, lis
GOCC_PLASMA_MEMBRANE_PROTEIN_COMPLEX	24	0.265	1.004	0.4627072	0.85534316	1	328	tags=58%, lis
GOCC_VACUOLAR_MEMBRANE	36	0.232	1.003	0.4587766	0.85439205	1	38	tags=6%, list-
GOBP_REGULATION_OF_T_CELL_DIFFERENTIATION	15	0.302	1.001	0.45545977	0.8559756	1	140	tags=27%, lis
HALLMARK_UV_RESPONSE_UP	17	0.282	1.001	0.45857987	0.85356534	1	17	tags=12%, lis
GOCC_INTRACELLULAR_PROTEIN_CONTAINING_COMPLEX	50	0.213	0.998	0.47788697	0.8582079	1	176	tags=30%, lis
GOMF_DNA_BINDING_TRANSCRIPTION_FACTOR_BINDING	32	0.239	0.997	0.46575344	0.8566857	1	168	tags=25%, lis
GOBP_INTEGRIN_MEDIATED_SIGNALING_PATHWAY	15	0.298	0.995	0.48040637	0.8581222	1	136	tags=27%, lis
HALLMARK_IL2_STAT5_SIGNALING	16	0.295	0.995	0.4664723	0.8561657	1	245	tags=50%, lis
GOBP_CELL_MIGRATION	92	0.188	0.994	0.48735633	0.8564619	1	151	tags=25%, lis
GOBP_ANATOMICAL_STRUCTURE_HOMEOSTASIS	28	0.250	0.993	0.46549392	0.85617036	1	138	tags=29%, lis
REACTOME_POST_TRANSLATIONAL_PROTEIN_MODIFICATION	90	0.191	0.991	0.485482	0.8569368	1	279	tags=39%, lis
GOBP_NEGATIVE_REGULATION_OF_TRANSFERASE_ACTIVITY	27	0.251	0.989	0.47606018	0.85871685	1	164	tags=30%, lis
REACTOME_MITOTIC_METAPHASE_AND_ANAPHASE	19	0.275	0.989	0.46501458	0.8563019	1	73	tags=21%, lis
GOBP_AGING	20	0.268	0.988	0.47099447	0.85675037	1	256	tags=45%, lis
GOBP_CELL_CYCLE_PROCESS	74	0.196	0.988	0.47287735	0.85449886	1	144	tags=24%, lis
GOCC_CHROMATIN	61	0.199	0.981	0.48410758	0.86559916	1	234	tags=34%, lis
GOMF_MRNA_BINDING	27	0.247	0.980	0.4893048	0.86610776	1	445	tags=78%, lis
GOBP_MRNA_EXPORT_FROM_NUCLEUS	19	0.273	0.978	0.4861111	0.8677735	1	255	tags=47%, lis
GOBP_ORGANELLE_ASSEMBLY	40	0.222	0.977	0.47727272	0.8667466	1	256	tags=33%, lis
GOMF_ADENYL_NUCLEOTIDE_BINDING	83	0.187	0.974	0.51724136	0.8700227	1	212	tags=30%, lis
GOBP_MRNA_TRANSPORT	19	0.273	0.974	0.4837341	0.8690122	1	255	tags=47%, lis
GOBP_PROTEIN_CONTAINING_COMPLEX_LOCALIZATION	30	0.237	0.972	0.4820442	0.8696927	1	255	tags=43%, lis
GOBP_POSITIVE_REGULATION_OF_CATALYTIC_ACTIVITY	95	0.184	0.972	0.49369988	0.86752754	1	136	tags=17%, lis
GOBP_NUCLEOSIDE_PHOSPHATE_BIOSYNTHETIC_PROCESS	20	0.268	0.969	0.48751837	0.8705227	1	44	tags=15%, lis
GOBP_CELLULAR_RESPONSE_TO_OXYGEN_CONTAINING_COMPOUND	80	0.186	0.966	0.5221843	0.87419593	1	81	tags=10%, lis
GOBP_REGULATION_OF_CELLULAR_COMPONENT_MOVEMENT	63	0.199	0.966	0.50924784	0.87180805	1	151	tags=25%, lis
GOBP_POSITIVE_REGULATION_OF_CELL_ACTIVATION	32	0.233	0.963	0.521682	0.87657267	1	108	tags=22%, lis
GOBP_CELL_CYCLE_ARREST	15	0.282	0.959	0.4845815	0.88199866	1	133	tags=27%, lis
KEGG_FOCAL_ADHESION	19	0.270	0.958	0.50214595	0.88207275	1	205	tags=37%, lis
KEGG_CHEMOKINE_SIGNALING_PATHWAY	16	0.280	0.957	0.51363635	0.8820381	1	487	tags=81%, lis
GOBP_CELLULAR_RESPONSE_TO_ORGANIC_CYCLIC_COMPOUND	36	0.223	0.956	0.5260417	0.8805451	1	110	tags=17%, lis
GOBP_POSITIVE_REGULATION_OF_NUCLEOBASE_CONTAINING_COMPOUND_METABOLIC_PROCES	129	0.173	0.955	0.5491892	0.88099784	1	194	tags=25%, lis
GOBP_REGULATION_OF_PROTEIN_MODIFICATION_PROCESS	116	0.174	0.953	0.5471698	0.88219297	1	135	tags=16%, lis
GOBP_REGULATION_OF_CELL_ACTIVATION	49	0.204	0.949	0.5253399	0.88882846	1	114	tags=18%, lis
GOCC_VACUOLE	66	0.188	0.945	0.54513067	0.89351034	1	38	tags=6%, list-
REACTOME_DEATH_RECEPTOR_SIGNALLING	19	0.268	0.944	0.53089887	0.8932543	1	449	tags=79%, lis
GOCC_INTRINSIC_COMPONENT_OF_PLASMA_MEMBRANE	45	0.204	0.942	0.5378788	0.8944934	1	339	tags=51%, lis
GOBP_NEGATIVE_REGULATION_OF_CATALYTIC_ACTIVITY	54	0.200	0.939	0.535	0.8978236	1	205	tags=28%, lis
GOBP_POSITIVE_REGULATION_OF_CYSTEINE_TYPE_ENDOPEPTIDASE_ACTIVITY	17	0.271	0.939	0.509887	0.8955684	1	94	tags=18%, lis
GOBP_POSITIVE_REGULATION_OF_CELL_CELL_ADHESION	23	0.247	0.937	0.534413	0.8975688	1	238	tags=43%, lis
GOBP_GENE_SILENCING	18	0.271	0.937	0.50814813	0.8952869	1	504	tags=83%, lis
GOBP_RNA_3_END_PROCESSING	19	0.254	0.935	0.52110624	0.89706534	1	379	tags=68%, lis
GOCC_MITOCHONDRION	96	0.175	0.935	0.57126695	0.89482474	1	46	tags=6%, list-
GOBP_INTRINSIC_APOPTOTIC_SIGNALING_PATHWAY	31	0.226	0.933	0.5431607	0.89528155	1	94	tags=16%, lis
GOBP_NEGATIVE_REGULATION_OF_INTRACELLULAR_SIGNAL_TRANSDUCTION	44	0.207	0.931	0.5495495	0.89659667	1	94	tags=16%, lis
GOBP_DNA_CONFORMATION_CHANGE	18	0.265	0.931	0.5386819	0.89554954	1	166	tags=33%, lis
GOBP_EPITHELIAL_CELL_PROLIFERATION	23	0.244	0.930	0.5284672	0.89445394	1	251	tags=39%, lis
GOMF_TRANSCRIPTION_COREPRESSOR_ACTIVITY	25	0.243	0.930	0.54432136	0.89258796	1	127	tags=24%, lis
REACTOME_RNA_POLYMERASE_II_TRANSCRIPTION_TERMINATION	16	0.273	0.929	0.5281899	0.89116675	1	255	tags=50%, lis
GOMF_PEPTIDE_BINDING	20	0.258	0.929	0.5467626	0.8904238	1	78	tags=15%, lis
REACTOME_CELL_CYCLE_MITOTIC	31	0.224	0.927	0.5643045	0.8921259	1	124	tags=23%, lis
GOBP_REGULATION_OF_MULTICELLULAR_ORGANISMAL_DEVELOPMENT	85	0.179	0.925	0.5735805	0.8934888	1	140	tags=20%, lis
GOBP_POSITIVE_REGULATION_OF_PROTEIN_PHOSPHORYLATION	51	0.199	0.924	0.55995053	0.8927251	1	114	tags=14%, lis
GOBP_REGULATION_OF_INTRINSIC_APOPTOTIC_SIGNALING_PATHWAY	20	0.251	0.922	0.54741377	0.8933605	1	261	tags=45%, lis
REACTOME_M_PHASE	27	0.229	0.921	0.5542328	0.89372665	1	78	tags=19%, lis
GOBP_POSITIVE_REGULATION_OF_HYDROLASE_ACTIVITY	49	0.200	0.920	0.55651104	0.89321053	1	136	tags=20%, lis

GOCC_ENDOPLASMIC_RETICULUM	104	0.169	0.919	0.60045147	0.8936639	1	279	tags=35%, lis
GOMF_CELL_ADHESION_MOLECULE_BINDING	35	0.218	0.919	0.57088125	0.89167136	1	99	tags=17%, lis
GOBP_REGULATION_OF_SMALL_MOLECULE_METABOLIC_PROCESS	33	0.224	0.919	0.55510205	0.8894721	1	78	tags=18%, lis
GOBP_POSITIVE_REGULATION_OF_CELL_ADHESION	32	0.221	0.916	0.5560054	0.89149225	1	385	tags=66%, lis
GOCC_CHROMOSOME	96	0.171	0.916	0.5899198	0.89015555	1	184	tags=26%, lis
GOBP_POSITIVE_REGULATION_OF_ORGANELLE_ORGANIZATION	41	0.206	0.916	0.5606258	0.88802385	1	105	tags=12%, lis
GOBP_SYNAPTIC_SIGNALING	22	0.248	0.915	0.55	0.8878311	1	501	tags=82%, lis
GOMF_RECEPTOR_REGULATOR_ACTIVITY	15	0.276	0.915	0.5511696	0.8860134	1	134	tags=27%, lis
GOBP_CELL_SURFACE_RECEPTOR_SIGNALING_PATHWAY_INVOLVED_IN_CELL_CELL_SIGNALING	48	0.201	0.912	0.5942928	0.88784564	1	140	tags=23%, lis
GOBP_NEGATIVE_REGULATION_OF_PROTEOLYSIS	15	0.274	0.911	0.5418502	0.8885135	1	258	tags=40%, lis
GOBP_REGULATION_OF_PROTEIN_CATABOLIC_PROCESS	28	0.231	0.909	0.54407716	0.8902027	1	328	tags=54%, lis
GOBP_NEGATIVE_REGULATION_OF_NUCLEOBASE_CONTAINING_COMPOUND_METABOLIC_PROCES	98	0.171	0.909	0.6142534	0.8882188	1	127	tags=16%, lis
GOBP_NUCLEAR_TRANSPORT	35	0.216	0.909	0.5884666	0.88612837	1	397	tags=63%, lis
GOBP_CELLULAR_RESPONSE_TO_OXYGEN_LEVELS	28	0.222	0.908	0.5517711	0.88439506	1	73	tags=18%, lis
GOBP_POSITIVE_REGULATION_OF_LEUKOCYTE_CELL_CELL_ADHESION	21	0.242	0.906	0.55965906	0.886692	1	238	tags=43%, lis
GOBP_NUCLEOBASE_CONTAINING_SMALL_MOLECULE_METABOLIC_PROCESS	41	0.204	0.902	0.5966709	0.8923769	1	100	tags=17%, lis
GOBP_POSITIVE_REGULATION_OF_CELL_DIFFERENTIATION	53	0.192	0.901	0.59445846	0.8921775	1	151	tags=23%, lis
GOMF_GTPASE_ACTIVATOR_ACTIVITY	15	0.269	0.900	0.56231004	0.8906934	1	513	tags=87%, lis
GOCC_CATALYTIC_COMPLEX	107	0.165	0.898	0.6074972	0.89284205	1	176	tags=25%, lis
GOBP_REGULATION_OF_PEPTIDYL_TYROSINE_PHOSPHORYLATION	17	0.254	0.897	0.5829726	0.89179075	1	334	tags=53%, lis
GOBP_REGULATION_OF_CELL_CELL_ADHESION	32	0.216	0.895	0.588	0.8948583	1	238	tags=38%, lis
GOBP_POSITIVE_REGULATION_OF_TRANSCRIPTION_BY_RNA_POLYMERASE_II	87	0.172	0.889	0.61403507	0.9028385	1	235	tags=30%, lis
REACTOME_DISEASES_OF_SIGNAL_TRANSDUCTION_BY_GROWTH_FACTOR_RECEPTORS_AND_SEC	43	0.196	0.888	0.58698374	0.90219265	1	146	tags=21%, lis
GOBP_NEGATIVE_REGULATION_OF_PROTEIN_METABOLIC_PROCESS	81	0.170	0.887	0.61264366	0.9018391	1	71	tags=10%, lis
GOCC_VESICLE_MEMBRANE	73	0.176	0.884	0.6260355	0.9060657	1	67	tags=8%, list-
GOBP_CELL_JUNCTION_ASSEMBLY	16	0.260	0.881	0.59379613	0.90823644	1	645	tags=100%, l
GOBP_TUBE_MORPHOGENESIS	48	0.189	0.880	0.6126582	0.90863943	1	151	tags=25%, lis
GOMF_AMIDE_BINDING	23	0.232	0.880	0.6260274	0.9072772	1	78	tags=13%, lis
GOCC_NUCLEAR_MEMBRANE	21	0.237	0.876	0.57434404	0.91064185	1	95	tags=24%, lis
REACTOME_RHO_GTPASE_CYCLE	33	0.212	0.875	0.5944518	0.9103721	1	291	tags=48%, lis
GOBP_CANONICAL_WNT_SIGNALING_PATHWAY	32	0.210	0.873	0.623803	0.91372526	1	133	tags=22%, lis
GOCC_EARLY_ENDOSOME	28	0.219	0.868	0.6225166	0.9189423	1	75	tags=14%, lis
GOBP_POSITIVE_REGULATION_OF_CELLULAR_PROTEIN_LOCALIZATION	22	0.232	0.861	0.61699164	0.9303031	1	240	tags=41%, lis
GOCC_TRANSFERASE_COMPLEX_TRANSFERRING_PHOSPHORUS_CONTAINING_GROUPS	18	0.243	0.860	0.62297297	0.93011755	1	337	tags=56%, lis
GOBP_BIOLOGICAL_ADHESION	71	0.168	0.857	0.6674556	0.93284214	1	151	tags=23%, lis
GOBP_NEGATIVE_REGULATION_OF_PHOSPHORYLATION	38	0.193	0.857	0.6571056	0.9306833	1	205	tags=29%, lis
GOBP_CELLULAR_RESPONSE_TO_STEROID_HORMONE_STIMULUS	16	0.249	0.855	0.62554425	0.9322886	1	72	tags=19%, lis
GOBP_RESPONSE_TO_STARVATION	16	0.247	0.855	0.63304096	0.9307237	1	71	tags=13%, lis
GOBP_BEHAVIOR	21	0.226	0.846	0.61695904	0.9447085	1	159	tags=29%, lis
GOBP_CELL_CELL_SIGNALING_BY_WNT	46	0.187	0.839	0.67114913	0.9532939	1	140	tags=22%, lis
GOBP_PEPTIDYL_TYROSINE_MODIFICATION	22	0.225	0.837	0.64534074	0.9546137	1	414	tags=64%, lis
GOBP_POSITIVE_REGULATION_OF_PROTEIN_KINASE_ACTIVITY	42	0.186	0.834	0.67665416	0.95710886	1	2	tags=2%, list-
GOBP_POSITIVE_REGULATION_OF_BIOSYNTHETIC_PROCESS	125	0.150	0.830	0.7169604	0.962723	1	194	tags=23%, lis
GOBP_LOCOMOTION	103	0.156	0.829	0.71657145	0.9617626	1	151	tags=22%, lis
GOBP_REGULATION_OF_B_CELL_ACTIVATION	16	0.246	0.828	0.6520509	0.9616224	1	382	tags=63%, lis
GOBP_HUMORAL_IMMUNE_RESPONSE	18	0.234	0.828	0.660485	0.9596881	1	138	tags=28%, lis
GOBP_REGULATION_OF_PROTEIN_SERINE_THREONINE_KINASE_ACTIVITY	42	0.188	0.827	0.6722581	0.9588002	1	2	tags=2%, list-
GOCC_NUCLEAR_BODY	71	0.164	0.827	0.6940211	0.95710874	1	302	tags=41%, lis
GOBP_MULTI_ORGANISM_PROCESS	50	0.178	0.825	0.66219515	0.95845324	1	151	tags=22%, lis
GOBP_POSITIVE_REGULATION_OF_TRANSFERASE_ACTIVITY	52	0.172	0.821	0.6695332	0.9621334	1	71	tags=8%, list-
GOCC_INTRINSIC_COMPONENT_OF_ORGANELLE_MEMBRANE	26	0.209	0.821	0.64305174	0.96072185	1	94	tags=15%, lis
GOBP_POSITIVE_REGULATION_OF_PHOSPHORUS_METABOLIC_PROCESS	60	0.169	0.820	0.6738609	0.96000797	1	135	tags=15%, lis
GOCC_TRANSFERASE_COMPLEX	54	0.176	0.820	0.6979294	0.9583556	1	176	tags=28%, lis
GOBP_PROTEIN_LOCALIZATION_TO_ORGANELLE	58	0.166	0.819	0.7121951	0.9567752	1	133	tags=14%, lis
GOMF_SEQUENCE_SPECIFIC_DNA_BINDING	73	0.163	0.817	0.7047059	0.95861125	1	184	tags=23%, lis
GOMF_TRANSCRIPTION_COREGULATOR_ACTIVITY	52	0.171	0.815	0.6885645	0.9599101	1	278	tags=37%, lis
GOBP_RECEPTOR_METABOLIC_PROCESS	16	0.238	0.813	0.68240345	0.9608189	1	136	tags=25%, lis
GOBP_REGULATION_OF_APOPTOTIC_SIGNALING_PATHWAY	33	0.188	0.811	0.66101694	0.96220815	1	275	tags=42%, lis
GOBP_POSITIVE_REGULATION_OF_HEMOPOIESIS	18	0.225	0.808	0.6676177	0.9642307	1	140	tags=28%, lis
GOBP_MRNA_3_END_PROCESSING	18	0.232	0.807	0.6853147	0.9650248	1	379	tags=67%, lis
REACTOME_METABOLISM_OF_RNA	52	0.171	0.806	0.7141089	0.96466357	1	179	tags=25%, lis
GOBP_HOMEOSTATIC_PROCESS	121	0.145	0.805	0.73752713	0.9626709	1	159	tags=21%, lis
GOBP_REGULATION_OF_LEUKOCYTE_MEDIATED_IMMUNITY	18	0.227	0.805	0.6862464	0.96068215	1	149	tags=22%, lis
GOBP_RESPONSE_TO_ORGANIC_CYCLIC_COMPOUND	62	0.165	0.803	0.7381818	0.9621311	1	93	tags=11%, lis
GOBP_RESPONSE_TO_OXYGEN_CONTAINING_COMPOUND	112	0.147	0.801	0.74585634	0.96371233	1	98	tags=11%, lis
GOBP_VESICLE_ORGANIZATION	29	0.198	0.800	0.67556745	0.96228313	1	245	tags=34%, lis
GOBP_REGULATION_OF_TRANSFERASE_ACTIVITY	74	0.155	0.797	0.7175481	0.9652844	1	134	tags=15%, lis
GOBP_NEGATIVE_REGULATION_OF_BIOSYNTHETIC_PROCESS	108	0.147	0.794	0.77130044	0.9684867	1	127	tags=15%, lis
GOBP_GLYCEROLIPID_METABOLIC_PROCESS	28	0.198	0.792	0.7031464	0.9698672	1	190	tags=29%, lis
GOBP_CELLULAR_RESPONSE_TO_REACTIVE_OXYGEN_SPECIES	16	0.232	0.790	0.680597	0.9707476	1	262	tags=50%, lis
GOCC_UBIQUITIN_LIGASE_COMPLEX	16	0.229	0.785	0.71198833	0.97584903	1	176	tags=38%, lis
GOBP_POSITIVE_REGULATION_OF_PROTEIN_CATABOLIC_PROCESS	20	0.216	0.784	0.7128713	0.9761467	1	158	tags=30%, lis
GOCC_ENDOSOME	71	0.155	0.784	0.7491124	0.9743791	1	319	tags=42%, lis
GOBP_REGULATION_OF_CELL_ADHESION	47	0.169	0.780	0.73433584	0.9786426	1	151	tags=21%, lis
GOBP_POSITIVE_REGULATION_OF_PROTEIN_CONTAINING_COMPLEX_ASSEMBLY	16	0.229	0.779	0.71363634	0.9778536	1	105	tags=19%, lis
GOBP_NEGATIVE_REGULATION_OF_KINASE_ACTIVITY	26	0.201	0.775	0.71922547	0.98148334	1	164	tags=27%, lis
GOBP_POSITIVE_REGULATION_OF_CELLULAR_COMPONENT_ORGANIZATION	69	0.155	0.775	0.74282295	0.9794475	1	14	tags=3%, list-
GOBP_TRANSMEMBRANE_TRANSPORT	75	0.153	0.770	0.7790974	0.984516	1	207	tags=27%, lis
REACTOME_MRNA_SPLICING	23	0.200	0.767	0.7275321	0.986845	1	412	tags=65%, lis

GOMF_ENZYME_BINDING	145	0.137	0.765	0.8133188	0.9883046	1	136	tags=15%, lis
GOBP_CELLULAR_RESPONSE_TO_HORMONE_STIMULUS	42	0.170	0.765	0.75609756	0.9869696	1	140	tags=19%, lis
REACTOME_CELL_CYCLE	39	0.174	0.764	0.75	0.98594564	1	198	tags=28%, lis
GOBP_POSITIVE_REGULATION_OF_CELLULAR_COMPONENT_BIOGENESIS	23	0.200	0.763	0.74441344	0.98556226	1	133	tags=22%, lis
GOBP_REGULATION_OF_MRNA_SPLICING_VIA_SPLICEOSOME	17	0.221	0.760	0.729927	0.9871497	1	424	tags=71%, lis
GOCC_NUCLEAR_SPECK	49	0.164	0.758	0.7432432	0.98882216	1	261	tags=35%, lis
GOBP_CELL_CYCLE	106	0.140	0.756	0.8006757	0.98916787	1	144	tags=20%, lis
REACTOME_NERVOUS_SYSTEM_DEVELOPMENT	32	0.180	0.755	0.7483703	0.988302	1	136	tags=22%, lis
GOBP_SMALL_GTPASE_MEDIATED_SIGNAL_TRANSDUCTION	36	0.176	0.754	0.7534766	0.98810095	1	492	tags=75%, lis
GOBP_SMALL_MOLECULE_METABOLIC_PROCESS	93	0.144	0.750	0.797282	0.9924412	1	108	tags=15%, lis
GOBP_RHYTHMIC_PROCESS	18	0.214	0.747	0.742532	0.9935696	1	416	tags=67%, lis
GOBP_ORGANOPHOSPHATE_METABOLIC_PROCESS	66	0.152	0.747	0.8030842	0.9922359	1	108	tags=15%, lis
GOCC_CENTROSOME	37	0.174	0.744	0.77922076	0.9935216	1	173	tags=24%, lis
GOCC_CYTOPLASMIC_SIDE_OF_MEMBRANE	17	0.210	0.742	0.7381295	0.99543244	1	513	tags=82%, lis
GOBP_RESPONSE_TO_HORMONE	58	0.154	0.739	0.7976048	0.9964675	1	72	tags=10%, lis
GOBP_POSITIVE_REGULATION_OF_CELL_POPULATION_PROLIFERATION	64	0.152	0.737	0.8135593	0.99761784	1	251	tags=33%, lis
GOBP_RESPONSE_TO_OXYGEN_LEVELS	42	0.164	0.734	0.80559087	0.9993133	1	124	tags=19%, lis
GOCC_CELL_PROJECTION_MEMBRANE	15	0.223	0.732	0.74057317	1	1	230	tags=40%, lis
GOBP_REGULATION_OF_REACTIVE_OXYGEN_SPECIES_METABOLIC_PROCESS	21	0.197	0.732	0.76230663	0.998848	1	99	tags=19%, lis
GOBP_CELLULAR_RESPONSE_TO_CHEMICAL_STRESS	25	0.186	0.731	0.7671233	0.9977334	1	262	tags=44%, lis
REACTOME_DNA_REPAIR	15	0.219	0.730	0.76	0.997304	1	176	tags=27%, lis
GOBP_POSITIVE_REGULATION_OF_LOCOMOTION	35	0.169	0.728	0.7930142	0.99791443	1	151	tags=23%, lis
GOMF_ION_TRANSMEMBRANE_TRANSPORTER_ACTIVITY	32	0.176	0.728	0.77470356	0.9961892	1	72	tags=13%, lis
HALLMARK_DNA_REPAIR	15	0.217	0.726	0.73738873	0.9967993	1	613	tags=93%, lis
GOBP_CATION_TRANSMEMBRANE_TRANSPORT	37	0.170	0.726	0.79715765	0.99496835	1	207	tags=30%, lis
GOBP_PEPTIDYL_AMINO_ACID_MODIFICATION	82	0.139	0.724	0.79516685	0.99522376	1	416	tags=54%, lis
GOBP_RESPONSE_TO_LIGHT_STIMULUS	18	0.204	0.721	0.7529586	0.9976711	1	164	tags=28%, lis
GOCC_CILIUM	20	0.200	0.716	0.77729887	1	1	254	tags=40%, lis
GOBP_REGULATION_OF_ION_TRANSPORT	69	0.145	0.713	0.8275058	1	1	94	tags=13%, lis
GOBP_ORGANOPHOSPHATE_BIOSYNTHETIC_PROCESS	39	0.165	0.712	0.7895425	1	1	108	tags=15%, lis
GOCC_NUCLEAR_ENVELOPE	27	0.177	0.712	0.77284944	1	1	95	tags=19%, lis
GOBP_CELL_CELL_SIGNALING	85	0.135	0.711	0.83959043	1	1	140	tags=19%, lis
GOCC_ENDOSOME_MEMBRANE	35	0.166	0.710	0.8134034	0.9997096	1	123	tags=17%, lis
GOBP_NEGATIVE_REGULATION_OF_ANION_TRANSPORT	16	0.210	0.710	0.79100144	0.99854463	1	78	tags=13%, lis
GOBP_ORGANIC_HYDROXY_COMPOUND_METABOLIC_PROCESS	17	0.206	0.708	0.79532164	0.99892855	1	117	tags=24%, lis
GOBP_RNA_SPLICING_VIA_TRANSESTERIFICATION_REACTIONS	43	0.156	0.707	0.7966752	0.9984525	1	424	tags=63%, lis
GOBP_NERVOUS_SYSTEM_PROCESS	35	0.167	0.706	0.81155777	0.996766	1	223	tags=31%, lis
GOBP_PROTON_TRANSMEMBRANE_TRANSPORT	15	0.213	0.706	0.77205884	0.99564046	1	152	tags=27%, lis
GOBP_GLYCEROPHOSPHOLIPID_METABOLIC_PROCESS	21	0.188	0.703	0.79036826	0.996605	1	190	tags=29%, lis
GOBP_NEGATIVE_REGULATION_OF_PHOSPHORUS_METABOLIC_PROCESS	43	0.157	0.700	0.8203125	0.99916774	1	135	tags=19%, lis
GOBP_RESPONSE_TO_PEPTIDE_HORMONE	29	0.172	0.698	0.82608694	0.99902606	1	133	tags=17%, lis
GOBP_REGULATION_OF_PROTEIN_LOCALIZATION_TO_MEMBRANE	18	0.197	0.696	0.78152496	1	1	235	tags=39%, lis
GOBP_NEGATIVE_REGULATION_OF_TRANSCRIPTION_BY_RNA_POLYMERASE_II	56	0.147	0.692	0.82560974	1	1	140	tags=16%, lis
GOBP_MRNA_METABOLIC_PROCESS	79	0.135	0.687	0.8446262	1	1	179	tags=24%, lis
GOBP_RESPONSE_TO_STEROID_HORMONE	21	0.186	0.684	0.82828283	1	1	72	tags=14%, lis
GOCC_ANCHORING_JUNCTION	46	0.149	0.683	0.8429448	1	1	185	tags=26%, lis
REACTOME_PROCESSING_OF_CAPPED_INTRON_CONTAINING_PRE_MRNA	31	0.170	0.683	0.81241566	1	1	412	tags=61%, lis
GOBP_REGULATION_OF_ORGANELLE_ORGANIZATION	86	0.130	0.678	0.85779816	1	1	262	tags=30%, lis
GOBP_ESTABLISHMENT_OR_MAINTENANCE_OF_CELL_POLARITY	15	0.201	0.675	0.82098764	1	1	136	tags=27%, lis
GOBP_REGULATION_OF_MITOCHONDRION_ORGANIZATION	15	0.201	0.672	0.84750736	1	1	235	tags=40%, lis
GOBP_REGULATION_OF_PROTEIN_PHOSPHORYLATION	81	0.131	0.668	0.8761468	1	1	135	tags=14%, lis
GOMF_DNA_BINDING_TRANSCRIPTION_ACTIVATOR_ACTIVITY	21	0.179	0.667	0.8086957	1	1	184	tags=29%, lis
GOCC_MICROTUBULE_ORGANIZING_CENTER	40	0.151	0.666	0.83524907	1	1	173	tags=23%, lis
GOBP_APOPTOTIC_SIGNALING_PATHWAY	55	0.137	0.664	0.87198067	1	1	275	tags=35%, lis
GOBP_MICROTUBULE_BASED_PROCESS	35	0.159	0.663	0.8544974	1	1	198	tags=29%, lis
GOCC_CELL_CELL_JUNCTION	17	0.189	0.658	0.84615386	1	1	133	tags=24%, lis
GOCC_DENDRITIC_TREE	31	0.158	0.656	0.8473684	1	1	530	tags=77%, lis
GOBP_POSITIVE_REGULATION_OF_DEVELOPMENTAL_PROCESS	75	0.129	0.653	0.8806683	1	1	151	tags=19%, lis
GOBP_REGULATION_OF_MRNA_METABOLIC_PROCESS	45	0.142	0.652	0.88065326	1	1	449	tags=64%, lis
GOCC_LATE_ENDOSOME	23	0.172	0.650	0.85335195	1	1	346	tags=52%, lis
GOCC_MEMBRANE_PROTEIN_COMPLEX	71	0.131	0.649	0.8943662	1	1	163	tags=20%, lis
GOBP_CELL_PROJECTION_ASSEMBLY	26	0.166	0.649	0.85055864	1	1	291	tags=42%, lis
GOBP_CELL_SUBSTRATE_ADHESION	18	0.191	0.646	0.8526012	1	1	395	tags=61%, lis
GOBP_RESPONSE_TO_EXTRACELLULAR_STIMULUS	25	0.165	0.646	0.85851645	1	1	7	tags=4%, list
GOBP_CELL_GROWTH	18	0.178	0.644	0.866571	1	1	715	tags=100%, l
GOBP_ORGANONITROGEN_COMPOUND_BIOSYNTHETIC_PROCESS	89	0.124	0.641	0.8975834	1	1	44	tags=4%, list
GOBP_EPITHELIUM_DEVELOPMENT	59	0.133	0.641	0.9140436	1	1	143	tags=17%, lis
GOMF_TRANSPORTER_ACTIVITY	39	0.144	0.638	0.88131315	1	1	72	tags=10%, lis
GOCC_SOMATODENDRITIC_COMPARTMENT	34	0.147	0.637	0.8736842	1	1	530	tags=76%, lis
GOBP_CIRCADIAN_RHYTHM	16	0.183	0.632	0.87042683	1	1	71	tags=13%, lis
GOBP_REGULATION_OF_PHOSPHORUS_METABOLIC_PROCESS	104	0.116	0.630	0.92522323	1	1	135	tags=13%, lis
GOBP_REGULATION_OF_PROTEIN_LOCALIZATION	56	0.130	0.626	0.88480395	1	1	136	tags=16%, lis
GOBP_CIRCULATORY_SYSTEM_PROCESS	25	0.157	0.613	0.8967391	1	1	105	tags=12%, lis
GOBP_POSTTRANSCRIPTIONAL_REGULATION_OF_GENE_EXPRESSION	52	0.130	0.611	0.9079445	1	1	135	tags=17%, lis
GOBP_CHROMATIN_ORGANIZATION	54	0.129	0.609	0.89605737	1	1	416	tags=56%, lis
GOCC_MEMBRANE_MICRODOMAIN	24	0.160	0.608	0.9	1	1	149	tags=25%, lis
GOBP_NEGATIVE_REGULATION_OF_NEURON_DEATH	15	0.185	0.606	0.8898551	1	1	261	tags=40%, lis
GOBP_CELL_ACTIVATION	143	0.109	0.605	0.94935346	1	1	140	tags=15%, lis
GOBP_RESPONSE_TO_MECHANICAL_STIMULUS	18	0.169	0.604	0.88986784	1	1	72	tags=11%, lis

GOBP_PEPTIDYL_LYSINE_MODIFICATION	31	0.146	0.604	0.9065934	1	1	416	tags=58%, lis
GOMF_TRANSCRIPTION_COACTIVATOR_ACTIVITY	26	0.153	0.604	0.8972973	1	1	738	tags=100%, l
GOMF_CHROMATIN_BINDING	38	0.137	0.604	0.9101412	1	1	164	tags=18%, lis
GOCC_AXON	25	0.156	0.603	0.886921	1	1	9	tags=4%, list
GOCC_OUTER_MEMBRANE	20	0.166	0.603	0.86618704	1	1	222	tags=30%, lis
REACTOME_HEMOSTASIS	47	0.130	0.600	0.90186334	1	1	205	tags=28%, lis
GOBP_POSITIVE_REGULATION_OF_APOPTOTIC_SIGNALING_PATHWAY	17	0.175	0.598	0.87941176	1	1	94	tags=18%, lis
GOBP_REGULATION_OF_INTRACELLULAR_SIGNAL_TRANSDUCTION	124	0.107	0.593	0.95756257	1	1	94	tags=11%, lis
GOBP_REGULATION_OF_BODY_FLUID_LEVELS	25	0.154	0.590	0.8992908	1	1	43	tags=8%, list
GOMF_PROTEIN_KINASE_ACTIVITY	30	0.147	0.588	0.91764706	1	1	419	tags=63%, lis
GOBP_REGULATION_OF_RAS_PROTEIN_SIGNAL_TRANSDUCTION	16	0.172	0.586	0.89349115	1	1	720	tags=100%, l
GOBP_MUSCLE_CELL_DIFFERENTIATION	23	0.151	0.585	0.91597795	1	1	179	tags=26%, lis
GOBP_GLYCOPROTEIN_METABOLIC_PROCESS	16	0.173	0.581	0.9098966	1	1	307	tags=50%, lis
GOBP_PEPTIDE_SECRETION	20	0.156	0.578	0.91715115	1	1	13	tags=5%, list
GOBP_PHOSPHOLIPID_METABOLIC_PROCESS	23	0.156	0.577	0.89972144	1	1	190	tags=26%, lis
GOBP_INORGANIC_ION_TRANSMEMBRANE_TRANSPORT	35	0.134	0.577	0.9146184	1	1	207	tags=29%, lis
GOMF_PROTEIN_SERINE_THREONINE_KINASE_ACTIVITY	25	0.146	0.575	0.9205674	1	1	419	tags=64%, lis
GOBP_SEXUAL_REPRODUCTION	32	0.134	0.569	0.9196891	1	1	131	tags=19%, lis
GOBP_STRESS_ACTIVATED_PROTEIN_KINASE_SIGNALING_CASCADE	20	0.154	0.565	0.91642654	1	1	468	tags=75%, lis
GOMF_PROTEIN_CONTAINING_COMPLEX_BINDING	85	0.106	0.558	0.9613583	1	1	179	tags=20%, lis
GOBP_CATION_TRANSPORT	55	0.115	0.555	0.94987774	1	1	172	tags=22%, lis
GOBP_EXTRINSIC_APOPTOTIC_SIGNALING_PATHWAY	22	0.147	0.551	0.9171348	1	1	275	tags=41%, lis
GOBP_REGULATION_OF_GROWTH	32	0.132	0.550	0.9329759	1	1	149	tags=19%, lis
GOBP_ANION_TRANSMEMBRANE_TRANSPORT	19	0.152	0.547	0.9357664	1	1	16	tags=5%, list
GOBP_PROTEIN_CONTAINING_COMPLEX_SUBUNIT_ORGANIZATION	124	0.098	0.545	0.9725576	1	1	166	tags=15%, lis
GOBP_CELLULAR_RESPONSE_TO_NITROGEN_COMPOUND	47	0.117	0.544	0.94919455	1	1	133	tags=13%, lis
GOCC_POSTSYNAPSE	28	0.132	0.543	0.94164455	1	1	132	tags=18%, lis
GOBP_REGULATION_OF_DEVELOPMENTAL_GROWTH	17	0.154	0.543	0.9335303	1	1	179	tags=24%, lis
GOCC_APICAL_PART_OF_CELL	20	0.151	0.541	0.9303338	1	1	211	tags=30%, lis
GOCC_APICAL_PLASMA_MEMBRANE	17	0.153	0.539	0.94011974	1	1	211	tags=29%, lis
GOBP_PROTEIN_PHOSPHORYLATION	103	0.101	0.539	0.96860987	1	1	145	tags=14%, lis
GOBP_REGULATION_OF_CELLULAR_PROTEIN_LOCALIZATION	42	0.120	0.536	0.93911916	1	1	240	tags=31%, lis
KEGG_SPLICEOSOME	16	0.155	0.534	0.9398827	1	1	378	tags=56%, lis
KEGG_INSULIN_SIGNALING_PATHWAY	17	0.156	0.531	0.93342775	1	1	114	tags=18%, lis
GOCC_RIBONUCLEOPROTEIN_GRANULE	27	0.134	0.530	0.9331476	1	1	79	tags=11%, lis
GOBP_CELLULAR_RESPONSE_TO_PEPTIDE_HORMONE_STIMULUS	22	0.141	0.529	0.9394366	1	1	133	tags=18%, lis
GOBP_MEMBRANE_ORGANIZATION	71	0.104	0.518	0.95769686	1	1	94	tags=10%, lis
REACTOME_SIGNALING_BY_RHO_GTPASES_MIRO_GTPASES_AND_RHOBTB3	46	0.114	0.518	0.96	1	1	291	tags=39%, lis
GOBP_REPRODUCTIVE_SYSTEM_DEVELOPMENT	26	0.128	0.512	0.946281	1	1	101	tags=15%, lis
GOBP_REGULATION_OF_SMALL_GTPASE_MEDIATED_SIGNAL_TRANSDUCTION	24	0.135	0.510	0.95753425	1	1	252	tags=33%, lis
GOBP_RNA_SPLICING	48	0.110	0.507	0.96333754	1	1	424	tags=58%, lis
GOBP_NEURON_DEATH	22	0.136	0.506	0.95815295	1	1	261	tags=36%, lis
GOCC_EXTRINSIC_COMPONENT_OF_MEMBRANE	24	0.132	0.502	0.9529737	1	1	75	tags=13%, lis
GOBP_CHEMICAL_HOMEOSTASIS	72	0.098	0.501	0.9861432	1	1	573	tags=78%, lis
GOBP_REGULATION_OF_CYTOSKELETON_ORGANIZATION	37	0.117	0.500	0.96437997	1	1	164	tags=22%, lis
GOMF_TRANSFERASE_ACTIVITY_TRANSFERRING_PHOSPHORUS_CONTAINING_GROUPS	47	0.109	0.495	0.9691358	1	1	419	tags=57%, lis
GOBP_REGULATION_OF_TRANSPORT	105	0.091	0.489	0.9829739	1	1	116	tags=12%, lis
GOBP_COVALENT_CHROMATIN_MODIFICATION	36	0.115	0.488	0.96847415	1	1	771	tags=100%, l
GOBP_HEAD_DEVELOPMENT	34	0.117	0.486	0.9525692	1	1	206	tags=26%, lis
GOCC_NUCLEAR_PROTEIN_CONTAINING_COMPLEX	78	0.095	0.485	0.98343194	1	1	416	tags=54%, lis
GOBP_PROTEIN_ACYLATION	23	0.127	0.485	0.97115386	1	1	759	tags=100%, l
GOBP_REGULATION_OF_CATION_TRANSMEMBRANE_TRANSPORT	15	0.145	0.483	0.95839524	1	1	205	tags=27%, lis
GOBP_RNA_PROCESSING	65	0.095	0.472	0.98086125	1	1	424	tags=57%, lis
GOBP_REGULATION_OF_ANION_TRANSPORT	49	0.100	0.464	0.97625	1	1	94	tags=10%, lis
GOBP_CELLULAR_COMPONENT_MORPHOGENESIS	27	0.115	0.460	0.98044693	1	1	646	tags=89%, lis
GOBP_REGULATION_OF_TRANSMEMBRANE_TRANSPORT	26	0.119	0.460	0.97534245	1	1	329	tags=42%, lis
GOBP_METAL_ION_TRANSPORT	22	0.122	0.457	0.9713506	1	1	329	tags=45%, lis
GOBP_DENDRITE_DEVELOPMENT	19	0.127	0.454	0.9843081	1	1	368	tags=53%, lis
GOBP_INTRACELLULAR_TRANSPORT	126	0.082	0.453	0.9945593	1	1	256	tags=29%, lis
GOBP_PROTEIN_ACETYLATION	19	0.126	0.452	0.9870875	1	1	759	tags=100%, l
GOBP_PEPTIDYL_LYSINE_ACETYLATION	18	0.126	0.442	0.978355	1	1	759	tags=100%, l
GOBP_RESPONSE_TO_ENDOPLASMIC_RETICULUM_STRESS	20	0.120	0.431	0.9885057	1	1	522	tags=75%, lis
GOBP_REGULATION_OF_SYSTEM_PROCESS	22	0.115	0.422	0.97884345	1	1	513	tags=73%, lis
GOBP_REGULATION_OF_ORGANELLE_ASSEMBLY	15	0.124	0.417	0.98944193	1	1	256	tags=33%, lis
GOBP_DEPHOSPHORYLATION	24	0.105	0.409	0.99858356	1	1	135	tags=17%, lis
GOBP_CELLULAR_RESPONSE_TO_PEPTIDE	25	0.105	0.407	0.98791945	1	1	133	tags=16%, lis
GOBP_POSITIVE_REGULATION_OF_DNA_BINDING_TRANSCRIPTION_FACTOR_ACTIVITY	21	0.104	0.394	0.9916318	1	1	171	tags=19%, lis
GOCC_AZUROPHIL_GRANULE	22	0.105	0.393	0.9900427	1	1	232	tags=27%, lis
GOBP_REGULATION_OF_INTRACELLULAR_PROTEIN_TRANSPORT	18	0.111	0.390	0.992722	1	1	772	tags=100%, l
GOBP_CELLULAR_RESPONSE_TO_INSULIN_STIMULUS	21	0.104	0.389	0.99291784	1	1	778	tags=100%, l
GOBP_REGULATION_OF_CELL_PROJECTION_ORGANIZATION	33	0.092	0.386	0.985034	1	1	516	tags=73%, lis
GOBP_CELLULAR_MACROMOLECULE_LOCALIZATION	135	0.067	0.379	0.99893504	1	1	255	tags=25%, lis
GOBP_LIPID_LOCALIZATION	20	0.103	0.374	0.9971671	1	1	159	tags=20%, lis
GOBP_RIBONUCLEOPROTEIN_COMPLEX_BIOGENESIS	17	0.108	0.373	0.99857956	1	1	774	tags=100%, l
GOBP_REGULATION_OF_CHROMOSOME_ORGANIZATION	19	0.096	0.351	0.9985856	1	1	256	tags=32%, lis
GOCC_MICROTUBULE_CYTOSKELETON	60	0.072	0.347	0.99880666	0.9996622	1	133	tags=13%, lis
GOCC_TRANSCRIPTION_REGULATOR_COMPLEX	21	0.092	0.335	0.9985507	0.9987229	1	632	tags=86%, lis
