# Supplementary material for: Biodegradable nanoparticles induce cGAS/STING-dependent reprogramming of myeloid cells to promote tumor immunotherapy
Source: Front Immunol. 2022 Aug 18;13:887649. doi: 10.3389/fimmu.2022.887649 (PMC9433741; doi:10.3389/fimmu.2022.887649)
Supplement: Supplementary file 15 [file Table_9.pdf]

Supplemental Table 9. Signaling Pathway Analysis for Nuetrophils - 3 Doses Once Every 3 Days - ONP-302 vs. Saline

NAME	SIZE	ES	NES	NOM p-val	FDR q-val	FWER p-val	RANK AT MA	LEADING ED
HALLMARK_MYC_TARGETS_V1	33	0.696	3.229	0	0	0	321	tags=88%, lis
GOMF_RNA_BINDING	182	0.395	2.665	0	0	0	336	tags=53%, lis
KEGG_ANTIGEN_PROCESSING_AND_PRESENTATION	16	0.755	2.663	0	0	0	194	tags=81%, lis
GOBP_B_CELL_MEDIATED_IMMUNITY	20	0.651	2.541	0	1.91E-04	0.001	49	tags=35%, lis
GOBP_B_CELL_RECEPTOR_SIGNALING_PATHWAY	19	0.640	2.496	0	8.11E-04	0.005	173	tags=58%, lis
GOCC_MITOCHONDRIAL_PROTEIN_CONTAINING_COMPLEX	19	0.624	2.491	0	6.76E-04	0.005	400	tags=95%, lis
GOCC_RIBONUCLEOPROTEIN_COMPLEX	62	0.461	2.491	0	5.79E-04	0.005	378	tags=68%, lis
GOBP_HUMORAL_IMMUNE_RESPONSE	33	0.535	2.478	0	6.03E-04	0.006	83	tags=36%, lis
REACTOME_SIGNALING_BY_THE_B_CELL_RECEPTOR_BCR	25	0.589	2.463	0	6.26E-04	0.007	344	tags=72%, lis
GOBP_DEFENSE_RESPONSE_TO_BACTERIUM	36	0.515	2.448	0	6.42E-04	0.008	155	tags=39%, lis
GOCC_INNER_MITOCHONDRIAL_MEMBRANE_PROTEIN_COMPLEX	15	0.682	2.414	0	0.001380361	0.019	400	tags=100%, l
REACTOME_TRANSLATION	15	0.649	2.411	0	0.001330895	0.02	248	tags=73%, lis
REACTOME_MHC_CLASS_II_ANTIGEN_PRESENTATION	16	0.665	2.379	0	0.001715990	0.028	195	tags=75%, lis
GOBP_TRANSLATIONAL_INITIATION	16	0.642	2.372	0	0.001877802	0.033	279	tags=75%, lis
REACTOME_PROCESSING_OF_CAPPED_INTRON_CONTAINING_PRE_MRNA	27	0.543	2.370	0.001937984	0.001805611	0.034	390	tags=81%, lis
GOBP_RNA_SPLICING_VIA_TRANSESTERIFICATION_REACTIONS	43	0.470	2.340	0	0.002592231	0.051	390	tags=72%, lis
GOBP_OXIDATIVE_PHOSPHORYLATION	18	0.597	2.296	0	0.003850943	0.08	400	tags=89%, lis
REACTOME_METABOLISM_OF_RNA	55	0.437	2.288	0	0.003816093	0.084	390	tags=73%, lis
GOBP_RIBONUCLEOPROTEIN_COMPLEX_SUBUNIT_ORGANIZATION	19	0.585	2.287	0	0.003655586	0.085	290	tags=74%, lis
REACTOME_MRNA_SPLICING	25	0.532	2.268	0	0.004421957	0.109	390	tags=80%, lis
REACTOME_RESPIRATORY_ELECTRON_TRANSPORT_ATP_SYNTHESIS_BY_CHEMIOSMOTIC_COUPLIN	18	0.586	2.266	0	0.004515356	0.114	400	tags=89%, lis
GOMF_TRANSLATION_REGULATOR_ACTIVITY_NUCLEIC_ACID_BINDING	15	0.616	2.226	0	0.006220506	0.161	279	tags=73%, lis
GOCC_SPLICEOSOMAL_COMPLEX	26	0.534	2.218	0	0.006436233	0.173	368	tags=77%, lis
GOBP_RNA_SPLICING	51	0.427	2.211	0	0.006759743	0.19	390	tags=69%, lis
GOBP_MEMBRANE_INVAGINATION	15	0.610	2.198	0	0.007854915	0.229	45	tags=33%, lis
KEGG_OXIDATIVE_PHOSPHORYLATION	18	0.574	2.187	0	0.008348484	0.244	421	tags=89%, lis
GOBP_REGULATION_OF_MRNA_SPLICING_VIA_SPLICEOSOME	19	0.552	2.173	0	0.009186647	0.269	386	tags=84%, lis
KEGG_HUNTINGTONS_DISEASE	23	0.534	2.168	0	0.009424915	0.286	400	tags=83%, lis
GOBP_REGULATION_OF_MRNA_PROCESSING	24	0.511	2.149	0	0.010629413	0.325	386	tags=79%, lis
GOBP_PROTEIN_FOLDING	19	0.542	2.133	0	0.012024399	0.366	232	tags=53%, lis
KEGG_PARKINSONS_DISEASE	18	0.550	2.119	0.002087682	0.013312055	0.405	400	tags=83%, lis
GOBP_ANTIGEN_RECEPTOR_MEDIATED_SIGNALING_PATHWAY	40	0.447	2.109	0	0.014362879	0.444	332	tags=65%, lis
GOBP_MRNA_PROCESSING	55	0.398	2.099	0	0.015582338	0.483	390	tags=67%, lis
GOMF_TRANSLATION_REGULATOR_ACTIVITY	19	0.542	2.086	0	0.01652697	0.504	330	tags=68%, lis
GOCC_EXTERNAL_SIDE_OF_PLASMA_MEMBRANE	34	0.461	2.074	0	0.01788215	0.541	103	tags=29%, lis
KEGG_SPLICEOSOME	21	0.505	2.015	0.004210526	0.02913387	0.726	390	tags=76%, lis
GOCC_ENDOPLASMIC_RETICULUM_PROTEIN_CONTAINING_COMPLEX	15	0.560	2.010	0	0.029730981	0.742	366	tags=80%, lis
GOBP_CHROMATIN_ASSEMBLY_OR_DISASSEMBLY	15	0.543	2.002	0.002061855	0.030752268	0.77	278	tags=67%, lis
GOBP_REGULATION_OF_B_CELL_ACTIVATION	25	0.477	2.000	0.004282655	0.030393006	0.773	33	tags=20%, lis
GOMF_GUANYL_NUCLEOTIDE_BINDING	29	0.447	1.989	0.004264392	0.032176852	0.799	81	tags=31%, lis
GOBP_RIBONUCLEOPROTEIN_COMPLEX_BIOGENESIS	28	0.449	1.970	0.01004016	0.03647903	0.852	290	tags=57%, lis
GOBP_ANTIGEN_PROCESSING_AND_PRESENTATION_OF_PEPTIDE_ANTIGEN	35	0.424	1.961	0.003846153	0.037919212	0.871	210	tags=46%, lis
GOBP_MITOCHONDRIAL_MEMBRANE_ORGANIZATION	16	0.525	1.943	0.004291845	0.042378474	0.904	302	tags=69%, lis
GOBP_REGULATION_OF_RNA_SPLICING	26	0.452	1.941	0.01026694	0.042155102	0.906	386	tags=73%, lis
GOMF_RIBONUCLEOPROTEIN_COMPLEX_BINDING	16	0.532	1.934	0.008264462	0.043538116	0.918	281	tags=63%, lis
GOBP_B_CELL_ACTIVATION	46	0.383	1.930	0.005928853	0.044075392	0.93	317	tags=50%, lis
GOBP_ADAPTIVE_IMMUNE_RESPONSE	66	0.354	1.921	0	0.04585157	0.943	103	tags=23%, lis
GOMF_MRNA_BINDING	36	0.401	1.896	0.001964636	0.054186344	0.967	380	tags=69%, lis
GOBP_LYMPHOCYTE_MEDIATED_IMMUNITY	34	0.418	1.888	0.004056795	0.0560674	0.971	85	tags=24%, lis
GOMF_DOUBLE_STRANDED_RNA_BINDING	15	0.531	1.879	0.010460251	0.05937084	0.977	272	tags=60%, lis
GOBP_PROTON_TRANSMEMBRANE_TRANSPORT	17	0.501	1.875	0.008213553	0.059565615	0.98	186	tags=47%, lis
GOBP_ANTIGEN_PROCESSING_AND_PRESENTATION	40	0.385	1.869	0.006012024	0.060962062	0.981	210	tags=43%, lis
GOBP_PROTEIN_DNA_COMPLEX_SUBUNIT_ORGANIZATION	15	0.524	1.863	0.008714597	0.062746406	0.987	277	tags=60%, lis
REACTOME_ASPARAGINE_N_LINKED_GLYCOSYLATION	21	0.453	1.842	0.024742268	0.07166664	0.991	169	tags=43%, lis
GOBP_BIOLOGICAL_PROCESS_INVOLVED_IN_SYMBIOTIC_INTERACTION	114	0.301	1.833	0.001795332	0.0748438	0.993	361	tags=52%, lis
GOBP_CELL_CYCLE_G2_M_PHASE_TRANSITION	25	0.430	1.832	0.010593221	0.07390632	0.993	402	tags=72%, lis
REACTOME_HOST_INTERACTIONS_OF_HIV_FACTORS	19	0.464	1.815	0.010416667	0.08184697	0.997	385	tags=68%, lis
GOBP_DNA_PACKAGING	15	0.507	1.812	0.010416667	0.082544506	0.997	278	tags=60%, lis
GOBP_CELL_KILLING	18	0.485	1.807	0.014344262	0.084260374	0.997	197	tags=44%, lis
GOBP_RESPONSE_TO_BACTERIUM	83	0.306	1.804	0.001930501	0.08497002	0.997	155	tags=25%, lis
GOBP_MRNA_METABOLIC_PROCESS	90	0.306	1.795	0.003891050	0.08870409	0.999	390	tags=59%, lis
REACTOME_DOWNSTREAM_SIGNALING_EVENTS_OF_B_CELL_RECEPTOR_BCR	17	0.470	1.781	0.020325202	0.0952931	0.999	385	tags=71%, lis
GOBP_INTRINSIC_APOPTOTIC_SIGNALING_PATHWAY_IN_RESPONSE_TO_DNA_DAMAGE	15	0.499	1.778	0.017167382	0.09611297	0.999	130	tags=33%, lis
GOBP_RNA_PROCESSING	79	0.312	1.773	0.009259259	0.09813716	1	390	tags=57%, lis
REACTOME_THE_CITRIC_ACID_TCA_CYCLE_AND_RESPIRATORY_ELECTRON_TRANSPORT	22	0.442	1.772	0.014314928	0.09688335	1	400	tags=73%, lis
REACTOME_MITOTIC_G2_G2_M_PHASES	18	0.458	1.768	0.008639309	0.09810542	1	321	tags=61%, lis
GOBP_INNATE_IMMUNE_RESPONSE	119	0.289	1.765	0.003731343	0.09869967	1	154	tags=24%, lis
GOBP_DEFENSE_RESPONSE_TO_OTHER_ORGANISM	137	0.275	1.746	0	0.11023022	1	155	tags=23%, lis
GOBP_ADAPTIVE_IMMUNE_RESPONSE_BASED_ON_SOMATIC_RECOMBINATION_OF_IMMUNE_RE	44	0.347	1.742	0.00952381	0.111096464	1	103	tags=20%, lis
GOBP_REGULATION_OF_CELL_CYCLE_G2_M_PHASE_TRANSITION	20	0.449	1.737	0.027083334	0.11363982	1	402	tags=75%, lis
GOBP_POSTTRANSCRIPTIONAL_REGULATION_OF_GENE_EXPRESSION	79	0.300	1.735	0.003616636	0.11388691	1	330	tags=49%, lis
HALLMARK_ALLOGRAFT_REJECTION	48	0.342	1.734	0.003773585	0.11260564	1	126	tags=27%, lis
GOBP_PEPTIDE_BIOSYNTHETIC_PROCESS	61	0.319	1.733	0.006122449	0.1120871	1	330	tags=51%, lis
GOBP_ACTIVATION_OF_IMMUNE_RESPONSE	75	0.303	1.730	0.005639097	0.11295612	1	173	tags=27%, lis
GOBP_FC_RECEPTOR_MEDIATED_STIMULATORY_SIGNALING_PATHWAY	20	0.434	1.722	0.0186722	0.116913415	1	67	tags=15%, lis
GOCC_VACUOLAR_LUMEN	27	0.393	1.709	0.016494846	0.12529983	1	217	tags=41%, lis

GOBP_TUMOR_NECROSIS_FACTOR_MEDIATED_SIGNALING_PATHWAY	19	0.435	1.705	0.01768172	0.12706646	1	321	tags=58%, lis
GOCC_PIGMENT_GRANULE	20	0.431	1.703	0.01367187	0.1263447	1	315	tags=60%, lis
GOBP_REGULATION_OF_DNA_METABOLIC_PROCESS	27	0.400	1.701	0.03483606	0.12644921	1	244	tags=48%, lis
GOBP_RESPONSE_TO_BIOTIC_STIMULUS	182	0.250	1.700	0.00176056	0.1258113	1	157	tags=23%, lis
GOMF_PROTON_TRANSMEMBRANE_TRANSPORTER_ACTIVITY	16	0.464	1.694	0.02941176	0.12890059	1	374	tags=69%, lis
GOCC_ENDOCYTIC_VESICLE	43	0.342	1.693	0.02057613	0.12810366	1	195	tags=33%, lis
GOCC_ENDOCYTIC_VESICLE_MEMBRANE	21	0.420	1.687	0.03536345	0.13174534	1	195	tags=38%, lis
GOBP_PURINE_CONTAINING_COMPOUND_BIOSYNTHETIC_PROCESS	15	0.473	1.682	0.02685950	0.13450047	1	366	tags=67%, lis
GOBP_REGULATION_OF_MRNA_METABOLIC_PROCESS	55	0.316	1.672	0.02127659	0.14089228	1	386	tags=62%, lis
GOCC_SIDE_OF_MEMBRANE	65	0.302	1.669	0.01119403	0.14190495	1	103	tags=18%, lis
GOBP_PEPTIDE_METABOLIC_PROCESS	74	0.296	1.663	0.02702702	0.14522044	1	330	tags=50%, lis
HALLMARK_OXIDATIVE_PHOSPHORYLATION	27	0.375	1.661	0.02136752	0.1455867	1	444	tags=70%, lis
GOMF_CYSTEINE_TYPE_PEPTIDASE_ACTIVITY	17	0.448	1.661	0.03696098	0.14415699	1	317	tags=59%, lis
REACTOME_CELL_CYCLE_CHECKPOINTS	15	0.473	1.661	0.03696098	0.14260893	1	443	tags=80%, lis
HALLMARK_INTERFERON_GAMMA_RESPONSE	49	0.318	1.656	0.01397205	0.14467825	1	254	tags=39%, lis
GOBP_ESTABLISHMENT_OF_RNA_LOCALIZATION	22	0.407	1.653	0.03149606	0.14594738	1	390	tags=68%, lis
GOBP_POSITIVE_REGULATION_OF_IMMUNE_RESPONSE	99	0.274	1.644	0.01369863	0.15208147	1	71	tags=15%, lis
GOBP_FC_EPSILON_RECEPTOR_SIGNALING_PATHWAY	25	0.388	1.635	0.03658536	0.15865259	1	388	tags=60%, lis
GOBP_DNA_CONFORMATION_CHANGE	22	0.404	1.633	0.03118908	0.1586799	1	278	tags=50%, lis
GOBP_POSITIVE_REGULATION_OF_TRANSLATION	15	0.448	1.629	0.04761905	0.16135642	1	313	tags=60%, lis
REACTOME_ANTIGEN_PROCESSING_CROSS_PRESENTATION	24	0.396	1.628	0.02636916	0.16071165	1	245	tags=46%, lis
GOBP_INTERFERON_GAMMA_MEDIATED_SIGNALING_PATHWAY	20	0.412	1.612	0.03667953	0.17423609	1	126	tags=40%, lis
GOMF_ENDOPEPTIDASE_ACTIVITY	26	0.367	1.604	0.05052631	0.18032837	1	321	tags=62%, lis
GOBP_RNA_LOCALIZATION	25	0.380	1.602	0.03689320	0.18070437	1	390	tags=64%, lis
GOBP_ANTIGEN_PROCESSING_AND_PRESENTATION_OF_PEPTIDE_ANTIGEN_VIA_MHC_CLASS_I	24	0.376	1.592	0.03441682	0.19016083	1	385	tags=67%, lis
GOBP_NUCLEAR_EXPORT	21	0.404	1.588	0.03238866	0.19202937	1	331	tags=62%, lis
GOBP_CELLULAR_RESPIRATION	15	0.442	1.587	0.04339250	0.19154367	1	400	tags=73%, lis
GOBP_TYPE_I_INTERFERON_PRODUCTION	20	0.393	1.575	0.05108055	0.20240897	1	180	tags=30%, lis
GOCC_INTRINSIC_COMPONENT_OF_ENDOPLASMIC_RETICULUM_MEMBRANE	19	0.407	1.573	0.05462185	0.20256338	1	169	tags=37%, lis
GOBP_DNA_METABOLIC_PROCESS	61	0.291	1.572	0.02691511	0.20159514	1	313	tags=46%, lis
REACTOME_SEPARATION_OF_SISTER_CHROMATIDS	15	0.446	1.564	0.054	0.20976299	1	685	tags=100%, l
GOBP_NUCLEOBASE_CONTAINING_COMPOUND_TRANSPORT	26	0.367	1.552	0.04646464	0.22140448	1	390	tags=65%, lis
REACTOME_ORGANELLE_BIOGENESIS_AND_MAINTENANCE	20	0.387	1.551	0.06012024	0.22034492	1	249	tags=50%, lis
GOCC_CELL_SURFACE	72	0.274	1.551	0.02935420	0.21841386	1	83	tags=17%, lis
GOBP_NON_CANONICAL_WNT_SIGNALING_PATHWAY	18	0.399	1.550	0.05894737	0.21834344	1	433	tags=72%, lis
GOBP_INTRINSIC_APOPTOTIC_SIGNALING_PATHWAY	43	0.317	1.543	0.03288201	0.224271	1	190	tags=28%, lis
GOBP_VIRAL_LIFE_CYCLE	36	0.327	1.542	0.04518664	0.22370219	1	101	tags=28%, lis
HALLMARK_UNFOLDED_PROTEIN_RESPONSE	15	0.433	1.540	0.06680585	0.22404863	1	189	tags=40%, lis
KEGG_ALZHEIMERS_DISEASE	24	0.373	1.534	0.07692308	0.22916102	1	412	tags=71%, lis
GOMF_CADHERIN_BINDING	53	0.295	1.534	0.02968460	0.2276937	1	279	tags=40%, lis
REACTOME_ADAPTIVE_IMMUNE_SYSTEM	109	0.251	1.533	0.02559415	0.22597939	1	195	tags=26%, lis
HALLMARK_INTERFERON_ALPHA_RESPONSE	28	0.342	1.520	0.06882591	0.23934583	1	173	tags=39%, lis
GOBP_ATP_METABOLIC_PROCESS	36	0.326	1.517	0.054	0.24100687	1	800	tags=97%, lis
GOBP_RIBOSE_PHOSPHATE_BIOSYNTHETIC_PROCESS	16	0.412	1.512	0.07039337	0.24578449	1	366	tags=63%, lis
GOBP_RESPONSE_TO_TOPOLOGICALLY_INCORRECT_PROTEIN	20	0.379	1.507	0.05781584	0.25048685	1	169	tags=35%, lis
GOBP_AMIDE_BIOSYNTHETIC_PROCESS	72	0.269	1.503	0.0331384	0.25332776	1	338	tags=46%, lis
GOBP_POSITIVE_REGULATION_OF_DNA_METABOLIC_PROCESS	17	0.400	1.499	0.07739308	0.2561381	1	274	tags=53%, lis
GOBP_ANTIGEN_PROCESSING_AND_PRESENTATION_OF_EXOGENOUS_PEPTIDE_ANTIGEN_VIA_MH	22	0.359	1.473	0.06719368	0.29101092	1	245	tags=45%, lis
GOBP_NUCLEAR_TRANSPORT	34	0.325	1.473	0.086	0.2889493	1	331	tags=56%, lis
GOBP_ANATOMICAL_STRUCTURE_HOMEOSTASIS	37	0.310	1.472	0.06732673	0.28824255	1	237	tags=38%, lis
GOBP_POSITIVE_REGULATION_OF_CELLULAR_AMIDE_METABOLIC_PROCESS	21	0.373	1.463	0.08502024	0.29842266	1	329	tags=52%, lis
GOBP_B_CELL_PROLIFERATION	17	0.390	1.452	0.0982906	0.31273267	1	314	tags=59%, lis
GOBP_FC_RECEPTOR_SIGNALING_PATHWAY	38	0.305	1.444	0.07083333	0.32475597	1	388	tags=50%, lis
REACTOME_FCGAMMA_RECEPTOR_FCGR_DEPENDENT_PHAGOCYTOSIS	16	0.396	1.442	0.10389610	0.32479587	1	67	tags=13%, lis
GOBP_VIRAL_GENOME_REPLICATION	16	0.385	1.441	0.08506224	0.3241022	1	101	tags=38%, lis
GOBP_DEFENSE_RESPONSE	198	0.210	1.440	0.01865671	0.32337818	1	190	tags=23%, lis
GOBP_REGULATION_OF_RESPONSE_TO_CYTOKINE_STIMULUS	28	0.328	1.435	0.1009901	0.32864	1	180	tags=32%, lis
REACTOME_POST_TRANSLATIONAL_PROTEIN_MODIFICATION	88	0.242	1.434	0.05222437	0.32842758	1	347	tags=43%, lis
GOBP_IMMUNE_RESPONSE_REGULATING_SIGNALING_PATHWAY	76	0.257	1.434	0.04500978	0.326182	1	173	tags=22%, lis
REACTOME_BETA_CATENIN_INDEPENDENT_WNT_SIGNALING	25	0.344	1.430	0.08884297	0.32902882	1	433	tags=68%, lis
GOCC_NUCLEOLUS	74	0.249	1.430	0.04892368	0.32737887	1	272	tags=35%, lis
GOBP_PRODUCTION_OF_MOLECULAR_MEDIATOR_OF_IMMUNE_RESPONSE	20	0.356	1.427	0.11422846	0.32923087	1	49	tags=20%, lis
REACTOME_CLASS_I_MHC_MEDIATED_ANTIGEN_PROCESSING_PRESENTATION	46	0.288	1.423	0.06719368	0.33370197	1	396	tags=54%, lis
REACTOME_CELLULAR_RESPONSE_TO_CHEMICAL_STRESS	30	0.309	1.414	0.10208333	0.34605944	1	167	tags=27%, lis
GOBP_ENERGY_DERIVATION_BY_OXIDATION_OF_ORGANIC_COMPOUNDS	22	0.347	1.413	0.12227074	0.3460522	1	416	tags=68%, lis
GOBP_LEUKOCYTE_MEDIATED_IMMUNITY	151	0.217	1.412	0.03499079	0.34390116	1	97	tags=14%, lis
REACTOME_CELL_SURFACE_INTERACTIONS_AT_THE_VASCULAR_WALL	28	0.315	1.411	0.10495049	0.3440642	1	78	tags=18%, lis
GOCC_MEMBRANE_PROTEIN_COMPLEX	100	0.236	1.404	0.0662768	0.3537417	1	493	tags=61%, lis
GOCC_PERINUCLEAR_REGION_OF_CYTOPLASM	62	0.259	1.403	0.08113591	0.3525744	1	194	tags=26%, lis
GOBP_MEMBRANE_ORGANIZATION	93	0.231	1.396	0.06403013	0.36218607	1	120	tags=15%, lis
GOBP_PURINE_CONTAINING_COMPOUND_METABOLIC_PROCESS	32	0.300	1.380	0.11928429	0.3885721	1	136	tags=25%, lis
GOBP_PROTEIN_CONTAINING_COMPLEX_SUBUNIT_ORGANIZATION	159	0.211	1.377	0.04823747	0.39124963	1	281	tags=33%, lis
GOBP_REGULATION_OF_CELLULAR_AMIDE_METABOLIC_PROCESS	57	0.256	1.372	0.09746589	0.3978998	1	330	tags=46%, lis
GOBP_POSITIVE_REGULATION_OF_CELL_ACTIVATION	53	0.266	1.370	0.08969466	0.3981798	1	49	tags=13%, lis
GOMF_ION_CHANNEL_BINDING	15	0.389	1.369	0.14016737	0.3970716	1	501	tags=80%, lis
REACTOME_TCR_SIGNALING	23	0.332	1.368	0.1390593	0.3965011	1	321	tags=52%, lis
GOMF_PROTEIN_DIMERIZATION_ACTIVITY	62	0.253	1.362	0.09437751	0.40421447	1	114	tags=19%, lis
GOBP_CELLULAR_AMIDE_METABOLIC_PROCESS	90	0.232	1.361	0.06756756	0.4043455	1	338	tags=43%, lis
PID_MYC_ACTIV_PATHWAY	17	0.363	1.360	0.1364562	0.40229112	1	202	tags=35%, lis

GOBP_TISSUE_HOMEOSTASIS	23	0.326	1.352	0.136	0.41521138	1	83	tags=22%, lis
KEGG_LYSOSOME	15	0.371	1.349	0.16969697	0.41863006	1	229	tags=53%, lis
GOCC_PHAGOCYTIC_VESICLE	27	0.315	1.348	0.15187377	0.4173909	1	152	tags=30%, lis
GOMF_ATPASE_ACTIVITY	33	0.303	1.347	0.12219959	0.41617692	1	398	tags=61%, lis
GOMF_PEPTIDASE_ACTIVITY	40	0.269	1.347	0.11290322	0.41505423	1	327	tags=50%, lis
GOBP_CELLULAR_MACROMOLECULE_CATABOLIC_PROCESS	117	0.216	1.345	0.07854406	0.41490728	1	326	tags=41%, lis
GOMF_PEPTIDE_BINDING	28	0.316	1.342	0.125	0.41765147	1	100	tags=21%, lis
REACTOME_TRANSCRIPTIONAL_REGULATION_BY_RUNX1	26	0.308	1.341	0.13461539	0.41735786	1	332	tags=50%, lis
GOBP_ELECTRON_TRANSPORT_CHAIN	15	0.368	1.341	0.1529175	0.41561058	1	403	tags=73%, lis
REACTOME_CYTOPROTECTION_BY_HMOX1	21	0.338	1.341	0.14115308	0.41317296	1	210	tags=33%, lis
GOCC_COATED_VESICLE_MEMBRANE	16	0.372	1.340	0.15866388	0.41227892	1	195	tags=31%, lis
GOBP_REGULATION_OF_VIRAL_LIFE_CYCLE	17	0.355	1.337	0.1532567	0.41534212	1	101	tags=35%, lis
GOBP_POSITIVE_REGULATION_OF_IMMUNE_SYSTEM_PROCESS	133	0.208	1.337	0.08269231	0.41367885	1	71	tags=12%, lis
GOBP_NUCLEOSIDE_PHOSPHATE_BIOSYNTHETIC_PROCESS	18	0.358	1.336	0.1431579	0.412028	1	366	tags=61%, lis
GOBP_CELLULAR_PROTEIN_CATABOLIC_PROCESS	75	0.237	1.335	0.09423077	0.41127244	1	321	tags=43%, lis
GOCC_PHAGOCYTIC_VESICLE_MEMBRANE	15	0.378	1.335	0.15271966	0.40943563	1	152	tags=33%, lis
GOBP_RESPONSE_TO_INTERFERON_GAMMA	31	0.294	1.334	0.15311909	0.40839496	1	154	tags=32%, lis
GOBP_PROTEASOMAL_PROTEIN_CATABOLIC_PROCESS	39	0.277	1.334	0.12734865	0.40614823	1	385	tags=56%, lis
GOBP_MODULATION_OF_PROCESS_OF_OTHER_ORGANISM_INVOLVED_IN_SYMBIOTIC_INTERACTI	16	0.366	1.330	0.1611479	0.41146988	1	360	tags=63%, lis
GOBP_REGULATION_OF_BIOLOGICAL_PROCESS_INVOLVED_IN_SYMBIOTIC_INTERACTION	24	0.312	1.325	0.16764133	0.4177866	1	101	tags=29%, lis
REACTOME_MITOTIC_METAPHASE_AND_ANAPHASE	18	0.347	1.325	0.15843621	0.41647857	1	736	tags=94%, lis
GOCC_TERTIARY_GRANULE_LUMEN	18	0.348	1.320	0.15139443	0.42265812	1	193	tags=33%, lis
REACTOME_CELL_CYCLE	50	0.265	1.315	0.13238288	0.4293932	1	418	tags=56%, lis
GOBP_INTRACELLULAR_TRANSPORT	152	0.201	1.315	0.1027027	0.42736065	1	333	tags=38%, lis
GOBP_POST_TRANSLATIONAL_PROTEIN_MODIFICATION	20	0.329	1.312	0.14947368	0.4301685	1	321	tags=55%, lis
GOBP_PHAGOCYTOSIS	52	0.250	1.312	0.125	0.42782706	1	67	tags=13%, lis
GOMF_PROTEIN_CONTAINING_COMPLEX_BINDING	131	0.206	1.311	0.09576427	0.42800027	1	281	tags=34%, lis
REACTOME_REGULATION_OF_MRNA_STABILITY_BY_PROTEINS_THAT_BIND_AU_RICH_ELEMENTS	15	0.363	1.311	0.16904277	0.4261841	1	385	tags=73%, lis
GOBP_CELLULAR_PROTEIN_CONTAINING_COMPLEX_ASSEMBLY	92	0.217	1.305	0.11047619	0.43446326	1	307	tags=38%, lis
GOBP_POSITIVE_REGULATION_OF_WNT_SIGNALING_PATHWAY	16	0.354	1.303	0.16630669	0.43634984	1	796	tags=100%, l
GOBP_IMMUNE_EFFECTOR_PROCESS	203	0.188	1.302	0.07517482	0.435793	1	101	tags=13%, lis
REACTOME_UB_SPECIFIC_PROCESSING_PROTEASES	18	0.339	1.300	0.17849898	0.43656686	1	321	tags=56%, lis
GOBP_CELLULAR_GLUCOSE_HOMEOSTASIS	15	0.371	1.300	0.1919192	0.4346588	1	87	tags=27%, lis
GOBP_ENDOCYTOSIS	74	0.225	1.298	0.13670412	0.43552217	1	78	tags=11%, lis
GOCC_VESICLE_LUMEN	59	0.242	1.296	0.14398421	0.43657994	1	91	tags=17%, lis
GOBP_LYMPHOCYTE_ACTIVATION	109	0.210	1.294	0.11111111	0.43810573	1	52	tags=11%, lis
GOCC_NUCLEAR_PROTEIN_CONTAINING_COMPLEX	97	0.215	1.283	0.12878788	0.4569463	1	386	tags=49%, lis
GOMF_PASSIVE_TRANSMEMBRANE_TRANSPORTER_ACTIVITY	16	0.348	1.280	0.1764706	0.4604269	1	182	tags=31%, lis
GOCC_RECEPTOR_COMPLEX	29	0.286	1.278	0.1764706	0.4627161	1	67	tags=17%, lis
GOBP_REGULATION_OF_IMMUNE_RESPONSE	140	0.200	1.269	0.11330935	0.4767005	1	71	tags=11%, lis
GOBP_REGULATION_OF_DNA_BINDING	17	0.341	1.269	0.19665273	0.474609	1	55	tags=18%, lis
GOBP_RESPONSE_TO_INORGANIC_SUBSTANCE	55	0.237	1.269	0.15705766	0.4724454	1	66	tags=15%, lis
GOMF_CATALYTIC_ACTIVITY_ACTING_ON_RNA	17	0.342	1.269	0.19381443	0.47082615	1	316	tags=53%, lis
GOMF_HISTONE_BINDING	26	0.297	1.266	0.17842324	0.474227	1	382	tags=54%, lis
GOMF_PROTEIN_HOMODIMERIZATION_ACTIVITY	46	0.247	1.263	0.16865079	0.476911	1	114	tags=20%, lis
GOMF_KINASE_REGULATOR_ACTIVITY	33	0.270	1.263	0.19057377	0.47485614	1	83	tags=18%, lis
GOBP_CELLULAR_RESPONSE_TO_TOPOLOGICALLY_INCORRECT_PROTEIN	19	0.329	1.263	0.19467214	0.47404557	1	169	tags=32%, lis
GOBP_RESPONSE_TO_ENDOPLASMIC_RETICULUM_STRESS	33	0.271	1.261	0.1875	0.47392032	1	333	tags=48%, lis
GOBP_REGULATION_OF_RESPONSE_TO_BIOTIC_STIMULUS	68	0.220	1.259	0.15267175	0.47643587	1	321	tags=41%, lis
GOBP_RECEPTOR_MEDIATED_ENDOCYTOSIS	41	0.259	1.259	0.17928287	0.47487235	1	6	tags=7%, list-
GOCC_TRANS_GOLGI_NETWORK	23	0.310	1.254	0.19077568	0.48231694	1	32	tags=13%, lis
GOBP_SUPEROXIDE_METABOLIC_PROCESS	16	0.341	1.253	0.2053388	0.48062932	1	39	tags=19%, lis
GOCC_PLASMA_MEMBRANE_PROTEIN_COMPLEX	50	0.251	1.251	0.17601547	0.48345578	1	76	tags=14%, lis
GOBP_PROTEIN_STABILIZATION	21	0.309	1.250	0.19959678	0.48153573	1	353	tags=52%, lis
REACTOME_CELL_CYCLE_MITOTIC	41	0.262	1.248	0.19726562	0.48426592	1	418	tags=56%, lis
GOMF_IDENTICAL_PROTEIN_BINDING	162	0.189	1.248	0.14642857	0.48209086	1	192	tags=21%, lis
REACTOME_ANTIGEN_PROCESSING_UBIQUITINATION_PROTEASOME_DEGRADATION	29	0.279	1.247	0.1971831	0.48111895	1	784	tags=93%, lis
GOBP_REGULATION_OF_BINDING	48	0.245	1.247	0.18108653	0.4789352	1	231	tags=31%, lis
GOCC_CHROMOSOMAL_REGION	28	0.284	1.247	0.20481928	0.47744468	1	307	tags=46%, lis
REACTOME_CLEC7A_DECTIN_1_SIGNALING	16	0.332	1.241	0.22088353	0.48639905	1	385	tags=69%, lis
GOBP_MITOCHONDRIAL_TRANSPORT	24	0.303	1.240	0.20883535	0.48613515	1	366	tags=54%, lis
REACTOME_HIV_INFECTION	24	0.299	1.240	0.23236515	0.48486167	1	385	tags=54%, lis
REACTOME_SIGNALING_BY_WNT	30	0.278	1.235	0.21428572	0.49180785	1	433	tags=60%, lis
GOCC_ORGANELLE_INNER_MEMBRANE	38	0.257	1.226	0.20588236	0.50594026	1	400	tags=55%, lis
GOMF_CELL_ADHESION_MOLECULE_BINDING	73	0.219	1.223	0.19092627	0.5116416	1	298	tags=37%, lis
HALLMARK_G2M_CHECKPOINT	23	0.296	1.217	0.22553192	0.52067274	1	343	tags=57%, lis
GOBP_RNA_CATABOLIC_PROCESS	49	0.240	1.213	0.21031746	0.526022	1	326	tags=45%, lis
GOBP_NUCLEOTIDE_PHOSPHORYLATION	15	0.340	1.212	0.22869022	0.5271247	1	136	tags=27%, lis
GOBP_RESPONSE_TO_VIRUS	43	0.239	1.211	0.2035928	0.5274856	1	146	tags=26%, lis
GOCC_INTRINSIC_COMPONENT_OF_ORGANELLE_MEMBRANE	29	0.273	1.203	0.25051335	0.54098696	1	169	tags=28%, lis
GOBP_CELLULAR_RESPONSE_TO_DNA_DAMAGE_STIMULUS	71	0.214	1.202	0.17181467	0.5400258	1	293	tags=34%, lis
GOBP_NEGATIVE_REGULATION_OF_CELL_CYCLE_PROCESS	41	0.251	1.198	0.252505	0.5472424	1	347	tags=51%, lis
GOMF_GTPASE_ACTIVITY	28	0.281	1.198	0.22406639	0.5451847	1	66	tags=18%, lis
GOCC_CATALYTIC_COMPLEX	116	0.189	1.195	0.19809523	0.5481503	1	572	tags=68%, lis
REACTOME_SARS_COV_INFECTIONS	19	0.306	1.192	0.24752475	0.5523657	1	242	tags=37%, lis
REACTOME_INFECTIOUS_DISEASE	94	0.201	1.189	0.21481481	0.5569718	1	197	tags=21%, lis
GOBP_NEGATIVE_REGULATION_OF_GENE_EXPRESSION	129	0.187	1.188	0.19056974	0.55511504	1	330	tags=40%, lis
REACTOME_M_PHASE	33	0.261	1.188	0.23173277	0.5538076	1	418	tags=58%, lis
GOMF_HYDROLASE_ACTIVITY_ACTING_ON_ACID_ANHYDRIDES	64	0.216	1.187	0.22568093	0.55412585	1	274	tags=34%, lis

GOBP_ENDOPLASMIC_RETICULUM_UNFOLDED_PROTEIN_RESPONSE	17	0.316	1.182	0.2559055	0.56218284	1	315	tags=47%, lis
GOBP_CELLULAR_RESPONSE_TO_RADIATION	19	0.296	1.178	0.25198412	0.5676009	1	17	tags=16%, lis
GOBP_ORGANONITROGEN_COMPOUND_BIOSYNTHETIC_PROCESS	129	0.186	1.176	0.18198875	0.5688592	1	214	tags=25%, lis
GOBP_REGULATION_OF_INTRINSIC_APOPTOTIC_SIGNALING_PATHWAY	26	0.274	1.176	0.25306123	0.56691325	1	190	tags=27%, lis
GOBP_POSITIVE_REGULATION_OF_I_KAPPAB_KINASE_NF_KAPPAB_SIGNALING	18	0.307	1.174	0.2774327	0.56854147	1	130	tags=22%, lis
GOBP_CHROMATIN_REMODELING	18	0.309	1.174	0.28367347	0.56681645	1	301	tags=44%, lis
GOBP_DNA_REPAIR	32	0.263	1.174	0.24590164	0.5648821	1	308	tags=44%, lis
GOBP_SIGNAL_TRANSDUCTION_BY_P53_CLASS_MEDIATOR	23	0.285	1.173	0.27004218	0.5637317	1	222	tags=35%, lis
GOBP_GENERATION_OF_PRECURSOR_METABOLITES_AND_ENERGY	52	0.223	1.171	0.25390625	0.5648926	1	417	tags=54%, lis
GOCC_MITOCHONDRIAL_ENVELOPE	49	0.233	1.171	0.26213592	0.5627996	1	400	tags=49%, lis
GOBP_NUCLEOBASE_CONTAINING_SMALL_MOLECULE_METABOLIC_PROCESS	39	0.246	1.166	0.25576922	0.5721995	1	136	tags=23%, lis
GOCC_LATE_ENDOSOME	28	0.272	1.163	0.24593496	0.5755455	1	193	tags=25%, lis
GOBP_MACROMOLECULE_CATABOLIC_PROCESS	135	0.182	1.158	0.23369566	0.58539695	1	326	tags=39%, lis
REACTOME_ANTI_INFLAMMATORY_RESPONSE_FAVOURING_LEISHMANIA_PARASITE_INFECTION	16	0.309	1.152	0.2832981	0.595927	1	2	tags=6%, list-
GOBP_REGULATION_OF_RESPONSE_TO_DNA_DAMAGE_STIMULUS	17	0.310	1.150	0.30398324	0.59658974	1	345	tags=53%, lis
GOCC_AZUROPHIL_GRANULE_LUMEN	18	0.295	1.148	0.306	0.5990428	1	261	tags=39%, lis
GOBP_MRNA_TRANSPORT	15	0.326	1.147	0.3014553	0.5990259	1	390	tags=67%, lis
GOBP_CELLULAR_HOMEOSTASIS	97	0.187	1.144	0.2574074	0.6028473	1	87	tags=13%, lis
GOBP_ACTOMYOSIN_STRUCTURE_ORGANIZATION	18	0.296	1.141	0.2915811	0.607874	1	161	tags=28%, lis
GOCC_DENDRITIC_TREE	35	0.246	1.139	0.29258516	0.60835844	1	207	tags=26%, lis
GOMF_AMIDE_BINDING	34	0.251	1.136	0.3	0.612383	1	100	tags=18%, lis
REACTOME_FC_EPSILON_RECEPTOR_FCERI_SIGNALING	28	0.257	1.134	0.27926078	0.6146469	1	388	tags=50%, lis
GOCC_SPECIFIC_GRANULE_LUMEN	16	0.309	1.131	0.29677418	0.61875355	1	83	tags=19%, lis
GOCC_FICOLIN_1_RICH_GRANULE_LUMEN	33	0.247	1.128	0.32082552	0.6236804	1	97	tags=21%, lis
GOBP_PROTEIN_CONTAINING_COMPLEX_DISASSEMBLY	27	0.266	1.128	0.3201581	0.62164086	1	241	tags=37%, lis
GOCC_NUCLEAR_CHROMOSOME	16	0.312	1.127	0.3126294	0.6208074	1	311	tags=56%, lis
GOBP_BIOLOGICAL_PROCESS_INVOLVED_IN_INTERACTION_WITH_HOST	22	0.286	1.125	0.318	0.62370765	1	82	tags=23%, lis
REACTOME_VESICLE_MEDIATED_TRANSPORT	69	0.200	1.124	0.27416173	0.6224	1	102	tags=12%, lis
GOBP_PROTEIN_CONTAINING_COMPLEX_LOCALIZATION	22	0.282	1.119	0.32051283	0.6309019	1	390	tags=59%, lis
GOMF_STRUCTURAL_MOLECULE_ACTIVITY	29	0.252	1.119	0.3190184	0.6292931	1	212	tags=34%, lis
GOBP_ESTABLISHMENT_OR_MAINTENANCE_OF_CELL_POLARITY	17	0.301	1.118	0.3146998	0.6292914	1	67	tags=18%, lis
REACTOME_INTERFERON_GAMMA_SIGNALING	17	0.294	1.115	0.31212723	0.6328078	1	126	tags=29%, lis
REACTOME_CELLULAR_RESPONSES_TO_EXTERNAL_STIMULI	65	0.205	1.112	0.328125	0.636935	1	171	tags=20%, lis
GOBP_NEGATIVE_REGULATION_OF_BINDING	24	0.268	1.112	0.3510204	0.6351393	1	26	tags=13%, lis
GOCC_FIBRILLAR_CENTER	15	0.314	1.112	0.33966243	0.63277817	1	479	tags=73%, lis
GOCC_VACUOLAR_MEMBRANE	51	0.217	1.111	0.29540917	0.6329502	1	117	tags=16%, lis
REACTOME_SIGNALING_BY_ROBO_RECEPTORS	17	0.298	1.111	0.34388185	0.6310304	1	767	tags=94%, lis
GOCC_PLASMA_MEMBRANE_SIGNALING_RECEPTOR_COMPLEX	19	0.286	1.108	0.3551797	0.63456446	1	52	tags=16%, lis
GOBP_RESPONSE_TO_HYDROGEN_PEROXIDE	25	0.264	1.108	0.33333334	0.6330824	1	66	tags=16%, lis
GOMF_SIGNALING_RECEPTOR_BINDING	133	0.177	1.107	0.30410448	0.6311446	1	100	tags=11%, lis
GOBP_MODULATION_OF_PROCESS_OF_OTHER_ORGANISM	17	0.294	1.103	0.33737373	0.63900745	1	360	tags=59%, lis
GOCC_TRANSPORT_VESICLE_MEMBRANE	16	0.295	1.101	0.3444882	0.6397145	1	32	tags=13%, lis
REACTOME_AUTOPHAGY	15	0.312	1.100	0.35643566	0.64020485	1	91	tags=20%, lis
GOBP_RESPONSE_TO_REACTIVE_OXYGEN_SPECIES	35	0.237	1.095	0.3264151	0.6495261	1	66	tags=14%, lis
GOBP_B_CELL_DIFFERENTIATION	24	0.265	1.094	0.34697855	0.64924455	1	317	tags=46%, lis
GOBP_POSITIVE_REGULATION_OF_CYSTEINE_TYPE_ENDOPEPTIDASE_ACTIVITY	20	0.273	1.094	0.36016098	0.6474764	1	63	tags=15%, lis
GOBP_REGULATION_OF_CELL_CYCLE_PHASE_TRANSITION	42	0.224	1.087	0.361829	0.65971005	1	339	tags=50%, lis
GOBP_REGULATION_OF_MRNA_CATABOLIC_PROCESS	35	0.231	1.087	0.33333334	0.6581706	1	326	tags=46%, lis
GOBP_POSITIVE_REGULATION_OF_BINDING	22	0.263	1.086	0.35146442	0.65802944	1	117	tags=23%, lis
GOBP_CELLULAR_RESPONSE_TO_CARBOHYDRATE_STIMULUS	15	0.307	1.086	0.36790606	0.6561825	1	81	tags=20%, lis
GOBP_PROTEIN_CATABOLIC_PROCESS	84	0.188	1.085	0.32307693	0.6561464	1	321	tags=39%, lis
GOBP_REGULATION_OF_IMMUNE_EFFECTOR_PROCESS	56	0.206	1.084	0.3553875	0.6545359	1	65	tags=9%, list-
GOCC_MEMBRANE_MICRODOMAIN	49	0.214	1.081	0.35119048	0.6598936	1	79	tags=14%, lis
GOBP_ORGANIC_CYCLIC_COMPOUND_CATABOLIC_PROCESS	59	0.201	1.075	0.34579438	0.6702492	1	224	tags=29%, lis
HALLMARK_MTORC1_SIGNALING	29	0.245	1.071	0.3563715	0.67829037	1	169	tags=28%, lis
GOBP_NEGATIVE_REGULATION_OF_CELLULAR_AMIDE_METABOLIC_PROCESS	28	0.240	1.070	0.38398358	0.6768747	1	330	tags=46%, lis
GOBP_RESPONSE_TO_OXIDATIVE_STRESS	59	0.197	1.069	0.3625954	0.6765182	1	81	tags=14%, lis
GOBP_CARBOHYDRATE_HOMEOSTASIS	20	0.267	1.068	0.37669903	0.67652035	1	87	tags=20%, lis
GOBP_REGULATION_OF_CELL_CYCLE_PROCESS	70	0.192	1.067	0.36311787	0.67712826	1	308	tags=41%, lis
GOBP_GENE_SILENCING	21	0.266	1.065	0.3604888	0.67993534	1	292	tags=48%, lis
GOBP_APOPTOTIC_PROCESS	206	0.153	1.062	0.34751773	0.6832745	1	152	tags=15%, lis
GOMF_LIPID_BINDING	67	0.190	1.049	0.3859649	0.71050334	1	102	tags=15%, lis
GOCC_CHROMOSOME	129	0.164	1.043	0.37924528	0.7210672	1	312	tags=36%, lis
GOBP_HEMATOPOIETIC_PROGENITOR_CELL_DIFFERENTIATION	19	0.267	1.042	0.39793813	0.7221456	1	321	tags=47%, lis
GOBP_REGULATION_OF_PROTEIN_STABILITY	32	0.228	1.040	0.38610038	0.7233969	1	357	tags=50%, lis
GOCC_RIBONUCLEOPROTEIN_GRANULE	34	0.228	1.037	0.40862423	0.72828734	1	224	tags=35%, lis
GOBP_ANATOMICAL_STRUCTURE_MATURATION	15	0.293	1.034	0.39300412	0.7320513	1	145	tags=27%, lis
GOMF_UBIQUITIN_LIKE_PROTEIN_LIGASE_BINDING	33	0.224	1.033	0.39494163	0.7323145	1	197	tags=27%, lis
GOCC_CLATHRIN_COATED_VESICLE	21	0.254	1.030	0.416499	0.7361159	1	195	tags=24%, lis
GOBP_CELL_CYCLE_PHASE_TRANSITION	53	0.200	1.030	0.42632613	0.73373884	1	349	tags=47%, lis
GOCC_COATED_VESICLE	29	0.234	1.029	0.41586074	0.7335106	1	91	tags=14%, lis
GOMF_PROTEIN_C_TERMINUS_BINDING	17	0.276	1.028	0.4122288	0.7335641	1	358	tags=53%, lis
GOMF_PEPTIDASE_REGULATOR_ACTIVITY	19	0.266	1.028	0.42307693	0.7320478	1	238	tags=42%, lis
GOBP_NIK_NF_KAPPAB_SIGNALING	22	0.249	1.020	0.40836653	0.7467043	1	321	tags=50%, lis
GOBP_POSITIVE_REGULATION_OF_LIPID_METABOLIC_PROCESS	15	0.289	1.015	0.406639	0.75572896	1	26	tags=13%, lis
REACTOME_TRANSMISSION_ACROSS_CHEMICAL_SYNAPSES	19	0.260	1.009	0.43512973	0.7689597	1	910	tags=100%, l
GOBP_HOMEOSTATIC_PROCESS	190	0.149	1.008	0.44642857	0.7681023	1	87	tags=11%, lis
GOBP_ESTABLISHMENT_OF_PROTEIN_LOCALIZATION_TO_MEMBRANE	26	0.235	1.003	0.42857143	0.7772676	1	297	tags=38%, lis
GOBP_POSITIVE_REGULATION_OF_RESPONSE_TO_BIOTIC_STIMULUS	41	0.204	1.002	0.45383105	0.7762786	1	354	tags=46%, lis

PID_MTOR_4PATHWAY	17	0.269	1.002	0.4813278	0.77386236	1	297	tags=41%, lis
GOBP_REGULATION_OF_PEPTIDYL_SERINE_PHOSPHORYLATION	16	0.273	0.997	0.46443516	0.7828748	1	67	tags=19%, lis
GOBP_ANIMAL_ORGAN_MORPHOGENESIS	62	0.184	0.994	0.45289856	0.78775275	1	210	tags=24%, lis
GOBP_NUCLEOSIDE_DIPHOSPHATE_METABOLIC_PROCESS	17	0.269	0.993	0.46370968	0.78720504	1	136	tags=24%, lis
GOBP_ENDOPLASMIC_RETICULUM_TO_GOLGI_VESICLE_MEDIATED_TRANSPORT	15	0.276	0.991	0.47291666	0.7895089	1	102	tags=20%, lis
REACTOME_POTENTIAL_THERAPEUTICS_FOR_SARS	16	0.267	0.990	0.46566522	0.7890977	1	79	tags=19%, lis
GOMF_DNA_BINDING_TRANSCRIPTION_FACTOR_ACTIVITY	78	0.170	0.989	0.47861508	0.78963685	1	173	tags=21%, lis
PID_BCR_5PATHWAY	21	0.249	0.989	0.4663951	0.7874373	1	52	tags=14%, lis
GOBP_PROTEIN_COMPLEX_OLIGOMERIZATION	20	0.245	0.988	0.4473684	0.78612643	1	94	tags=15%, lis
GOBP_MODIFICATION_DEPENDENT_MACROMOLECULE_CATABOLIC_PROCESS	63	0.182	0.987	0.48217636	0.7862965	1	396	tags=49%, lis
GOBP_CELLULAR_RESPONSE_TO_ABIOTIC_STIMULUS	41	0.198	0.982	0.48169556	0.7958181	1	39	tags=10%, lis
GOBP_REACTIVE_OXYGEN_SPECIES_METABOLIC_PROCESS	38	0.209	0.978	0.5019231	0.8009573	1	67	tags=13%, lis
GOBP_APOPTOTIC_SIGNALING_PATHWAY	77	0.173	0.977	0.5225933	0.8018179	1	308	tags=31%, lis
GOBP_POSITIVE_REGULATION_OF_APOPTOTIC_SIGNALING_PATHWAY	23	0.234	0.975	0.47474748	0.8034839	1	207	tags=30%, lis
GOCC_CHROMOSOME_CENTROMERIC_REGION	15	0.277	0.974	0.47689074	0.8040344	1	307	tags=47%, lis
GOBP_RESPONSE_TO_TEMPERATURE_STIMULUS	16	0.271	0.972	0.5031579	0.80454534	1	91	tags=19%, lis
GOCC_TRANSPORT_VESICLE	30	0.217	0.967	0.50898206	0.8132046	1	49	tags=10%, lis
HALLMARK_COMPLEMENT	42	0.192	0.964	0.5261044	0.8185192	1	231	tags=26%, lis
GOMF_DNA_BINDING_TRANSCRIPTION_REPRESSOR_ACTIVITY	29	0.215	0.963	0.484556	0.81913465	1	149	tags=21%, lis
GOBP_CATION_TRANSMEMBRANE_TRANSPORT	53	0.184	0.954	0.5142315	0.8360237	1	302	tags=34%, lis
GOBP_POSITIVE_REGULATION_OF_MRNA_METABOLIC_PROCESS	20	0.242	0.953	0.49685535	0.8357806	1	386	tags=60%, lis
REACTOME_NEUROTRANSMITTER_RECEPTORS_AND_POSTSYNAPTIC_SIGNAL_TRANSMISSION	16	0.260	0.953	0.48775056	0.8342251	1	910	tags=100%, l
KEGG_INSULIN_SIGNALING_PATHWAY	21	0.234	0.951	0.527668	0.83609945	1	841	tags=95%, lis
GOCC_SUPRAMOLECULAR_COMPLEX	98	0.159	0.951	0.5555556	0.8344374	1	231	tags=26%, lis
GOBP_CHEMICAL_HOMEOSTASIS	103	0.155	0.949	0.5591603	0.8344733	1	87	tags=12%, lis
GOBP_CHROMOSOME_ORGANIZATION	93	0.156	0.945	0.5510204	0.84121436	1	382	tags=42%, lis
GOBP_REGULATION_OF_INNATE_IMMUNE_RESPONSE	52	0.185	0.944	0.5425743	0.8417515	1	321	tags=38%, lis
GOBP_POSITIVE_REGULATION_OF_LEUKOCYTE_PROLIFERATION	16	0.258	0.937	0.5113402	0.8538064	1	103	tags=19%, lis
GOBP_PROTEIN_LOCALIZATION_TO_NUCLEUS	26	0.216	0.934	0.5573441	0.8580663	1	183	tags=31%, lis
GOBP_POSITIVE_REGULATION_OF_PEPTIDASE_ACTIVITY	26	0.223	0.934	0.5406977	0.8571182	1	193	tags=23%, lis
GOBP_RHYTHMIC_PROCESS	26	0.217	0.931	0.53424656	0.8598336	1	329	tags=46%, lis
GOBP_PROTEIN_POLYUBIQUITINATION	33	0.206	0.930	0.53585654	0.8592882	1	396	tags=55%, lis
GOBP_DNA_RECOMBINATION	16	0.259	0.928	0.5395034	0.8627697	1	293	tags=44%, lis
GOBP_VIRAL_GENE_EXPRESSION	18	0.245	0.925	0.54303277	0.86545897	1	382	tags=61%, lis
GOBP_CARBOHYDRATE_DERIVATIVE_METABOLIC_PROCESS	73	0.167	0.924	0.57088125	0.8649965	1	195	tags=21%, lis
GOCC_VACUOLE	96	0.155	0.923	0.58666664	0.8650249	1	234	tags=24%, lis
GOBP_CATION_TRANSPORT	79	0.160	0.921	0.5610687	0.8664389	1	195	tags=20%, lis
GOBP_MITOCHONDRION_ORGANIZATION	42	0.188	0.921	0.57768923	0.86482346	1	508	tags=64%, lis
GOCC_ORGANELLE_SUBCOMPARTMENT	131	0.145	0.917	0.61567163	0.87036735	1	201	tags=19%, lis
REACTOME_TRANSCRIPTIONAL_REGULATION_BY_RUNX3	20	0.229	0.914	0.5444915	0.8745161	1	843	tags=95%, lis
GOBP_PROTEIN_LOCALIZATION_TO_ORGANELLE	75	0.165	0.912	0.60491496	0.8777228	1	333	tags=40%, lis
GOBP_TISSUE_MORPHOGENESIS	46	0.180	0.910	0.584	0.87811536	1	230	tags=28%, lis
GOCC_GOLGI_APPARATUS_SUBCOMPARTMENT	73	0.162	0.906	0.60877866	0.8854172	1	245	tags=23%, lis
GOBP_PROTEIN_MODIFICATION_BY_SMALL_PROTEIN_REMOVAL	27	0.208	0.902	0.58666664	0.89097774	1	321	tags=41%, lis
REACTOME_NEDDYLATION	15	0.256	0.899	0.59342915	0.89518595	1	321	tags=47%, lis
GOBP_REGULATION_OF_PROTEASOMAL_PROTEIN_CATABOLIC_PROCESS	18	0.240	0.898	0.5777311	0.89479035	1	183	tags=28%, lis
GOBP_INTRACELLULAR_PROTEIN_TRANSPORT	95	0.152	0.897	0.64243615	0.8935549	1	333	tags=36%, lis
GOBP_REGULATION_OF_CELL_DEATH	182	0.132	0.896	0.66487455	0.89321953	1	194	tags=18%, lis
GOBP_CELLULAR_RESPONSE_TO_REACTIVE_OXYGEN_SPECIES	24	0.217	0.894	0.5904366	0.8946667	1	46	tags=13%, lis
GOMF_CATION_TRANSMEMBRANE_TRANSPORTER_ACTIVITY	32	0.192	0.894	0.5967742	0.89237076	1	186	tags=22%, lis
GOBP_INORGANIC_ION_TRANSMEMBRANE_TRANSPORT	48	0.176	0.892	0.63257575	0.89570695	1	302	tags=33%, lis
GOBP_NCRNA_PROCESSING	16	0.251	0.890	0.5694716	0.89723486	1	254	tags=38%, lis
GOBP_REGULATION_OF_GENE_EXPRESSION_EPIGENETIC	16	0.235	0.888	0.58299595	0.8976012	1	842	tags=94%, lis
GOBP_CHROMATIN_ORGANIZATION	69	0.158	0.884	0.64314514	0.90336627	1	382	tags=42%, lis
GOCC_SOMATODENDRITIC_COMPARTMENT	48	0.175	0.879	0.64241165	0.91199446	1	207	tags=21%, lis
GOBP_REGULATION_OF_NEURON_DIFFERENTIATION	17	0.231	0.878	0.60571426	0.9125214	1	484	tags=65%, lis
GOBP_STEM_CELL_DIFFERENTIATION	19	0.229	0.877	0.6106383	0.91205233	1	622	tags=79%, lis
GOMF_SEQUENCE_SPECIFIC_DNA_BINDING	106	0.143	0.876	0.68490565	0.9102402	1	190	tags=19%, lis
GOBP_CELLULAR_RESPONSE_TO_HYDROGEN_PEROXIDE	15	0.245	0.871	0.60162604	0.918776	1	46	tags=13%, lis
GOBP_REGULATION_OF_ACTIN_FILAMENT_ORGANIZATION	34	0.184	0.868	0.6222664	0.9218463	1	280	tags=32%, lis
GOMF_TRANSCRIPTION_FACTOR_BINDING	71	0.155	0.867	0.6660305	0.9211457	1	372	tags=41%, lis
GOBP_REGULATION_OF_APOPTOTIC_SIGNALING_PATHWAY	48	0.168	0.860	0.6769826	0.9331466	1	219	tags=23%, lis
GOBP_DEVELOPMENTAL_MATURATION	18	0.230	0.857	0.60554373	0.93722004	1	145	tags=22%, lis
GOBP_INTRACELLULAR_RECEPTOR_SIGNALING_PATHWAY	30	0.195	0.855	0.65762	0.9381623	1	753	tags=83%, lis
GOBP_NEGATIVE_REGULATION_OF_CYTOSKELETON_ORGANIZATION	18	0.224	0.850	0.6380753	0.9465181	1	102	tags=17%, lis
GOBP_RESPONSE_TO_TUMOR_NECROSIS_FACTOR	33	0.187	0.849	0.64243615	0.94511664	1	238	tags=27%, lis
GOMF_CIS_REGULATORY_REGION_SEQUENCE_SPECIFIC_DNA_BINDING	79	0.149	0.849	0.7012987	0.94332016	1	177	tags=19%, lis
GOBP_T_CELL_RECEPTOR_SIGNALING_PATHWAY	25	0.202	0.848	0.6680162	0.9422183	1	321	tags=48%, lis
GOCC_DISTAL_AXON	16	0.233	0.847	0.65106386	0.94237226	1	652	tags=81%, lis
GOBP_REGULATION_OF_CYSTEINE_TYPE_ENDOPEPTIDASE_ACTIVITY	35	0.183	0.845	0.6513944	0.9436102	1	83	tags=11%, lis
GOBP_CARBOHYDRATE_CATABOLIC_PROCESS	23	0.210	0.845	0.6539924	0.94130147	1	231	tags=26%, lis
GOBP_ESTABLISHMENT_OF_PROTEIN_LOCALIZATION_TO_ORGANELLE	45	0.171	0.841	0.67241377	0.9470112	1	333	tags=42%, lis
GOMF_NUCLEAR_HORMONE_RECEPTOR_BINDING	22	0.212	0.839	0.6598778	0.94693935	1	277	tags=36%, lis
GOCC_CHROMATIN	94	0.145	0.837	0.7560074	0.94977605	1	222	tags=24%, lis
GOBP_REGULATION_OF_CYTOSKELETON_ORGANIZATION	61	0.156	0.835	0.73018867	0.95020574	1	280	tags=30%, lis
GOBP_GOLGI_VESICLE_TRANSPORT	29	0.185	0.833	0.6666667	0.9524882	1	102	tags=14%, lis
GOBP_ION_HOMEOSTASIS	75	0.148	0.832	0.7201493	0.95136106	1	83	tags=11%, lis
GOBP_REGULATION_OF_LEUKOCYTE_APOPTOTIC_PROCESS	17	0.226	0.830	0.6864754	0.9519398	1	171	tags=24%, lis
GOMF_ION_TRANSMEMBRANE_TRANSPORTER_ACTIVITY	51	0.159	0.828	0.70137525	0.95411843	1	186	tags=20%, lis

GOMF_SH3_DOMAIN_BINDING	16	0.227	0.828	0.6438356	0.95222884	1	949	tags=100%, l
GOBP_REGULATION_OF_CYTOSOLIC_CALCIUM_ION_CONCENTRATION	32	0.180	0.826	0.68875504	0.9527329	1	79	tags=13%, lis
KEGG_RENAL_CELL_CARCINOMA	15	0.230	0.824	0.66049385	0.9544223	1	799	tags=93%, lis
GOMF_KINASE_BINDING	91	0.139	0.823	0.72904485	0.95332396	1	67	tags=9%, list-
GOBP_REGULATION_OF_DEPHOSPHORYLATION	25	0.197	0.822	0.69135803	0.9535526	1	315	tags=40%, lis
REACTOME_NERVOUS_SYSTEM_DEVELOPMENT	46	0.164	0.818	0.7265306	0.9591327	1	210	tags=24%, lis
GOBP_DIVALENT_INORGANIC_CATION_HOMEOSTASIS	43	0.162	0.813	0.72267205	0.964581	1	333	tags=40%, lis
GOCC_ENDOSOME	105	0.133	0.810	0.79651165	0.9670305	1	195	tags=18%, lis
GOBP_POSITIVE_REGULATION_OF_ENDOTHELIAL_CELL_MIGRATION	16	0.223	0.809	0.6991342	0.96617913	1	152	tags=25%, lis
REACTOME_NEURONAL_SYSTEM	23	0.195	0.808	0.6854839	0.96544945	1	846	tags=91%, lis
GOBP_HOMEOSTASIS_OF_NUMBER_OF_CELLS	37	0.170	0.805	0.73745173	0.96820855	1	199	tags=22%, lis
GOBP_MORPHOGENESIS_OF_AN_EPITHELIUM	39	0.169	0.802	0.7153846	0.97197366	1	230	tags=28%, lis
GOBP_MACROPHAGE_ACTIVATION	15	0.225	0.800	0.724359	0.9730553	1	149	tags=20%, lis
GOBP_CELLULAR_COMPONENT_DISASSEMBLY	51	0.155	0.799	0.7529183	0.97227883	1	102	tags=14%, lis
GOBP_RESPONSE_TO_LIGHT_STIMULUS	26	0.183	0.799	0.6964657	0.9705167	1	17	tags=8%, list-
GOBP_ACTIN_FILAMENT_BUNDLE_ORGANIZATION	19	0.208	0.797	0.7210216	0.97094464	1	154	tags=21%, lis
GOBP_REGULATION_OF_CELLULAR_PROTEIN_CATABOLIC_PROCESS	24	0.196	0.795	0.7113821	0.9716763	1	183	tags=25%, lis
GOBP_NEGATIVE_REGULATION_OF_ORGANELLE_ORGANIZATION	32	0.171	0.795	0.73828125	0.9703226	1	307	tags=38%, lis
GOBP_TRANSMEMBRANE_TRANSPORT	103	0.126	0.791	0.8312388	0.97424823	1	376	tags=40%, lis
GOBP_SISTER_CHROMATID_SEGREGATION	15	0.222	0.791	0.7055336	0.97214246	1	443	tags=67%, lis
GOMF_HORMONE_RECEPTOR_BINDING	23	0.190	0.790	0.7169811	0.97113484	1	277	tags=35%, lis
GOMF_NUCLEAR_RECEPTOR_BINDING	16	0.214	0.787	0.72	0.97317725	1	358	tags=50%, lis
REACTOME_TP53_REGULATES_METABOLIC_GENES	18	0.209	0.787	0.7392197	0.9716529	1	402	tags=50%, lis
GOBP_NCRNA_METABOLIC_PROCESS	17	0.211	0.786	0.7209776	0.97073036	1	254	tags=35%, lis
REACTOME_DEUBIQUITINATION	24	0.187	0.786	0.749004	0.9684326	1	321	tags=42%, lis
GOBP_ERYTHROCYTE_HOMEOSTASIS	19	0.204	0.786	0.72469634	0.96648884	1	141	tags=21%, lis
GOCC_ENVELOPE	93	0.129	0.785	0.81589144	0.9653135	1	202	tags=19%, lis
GOCC_CELL_BODY	33	0.173	0.784	0.7556468	0.9643282	1	207	tags=21%, lis
GOCC_NEURON_TO_NEURON_SYNAPSE	17	0.211	0.784	0.72168905	0.9632746	1	660	tags=82%, lis
GOCC_NUCLEAR_BODY	82	0.136	0.776	0.79041916	0.97241765	1	434	tags=49%, lis
REACTOME_CHROMATIN_MODIFYING_ENZYMES	21	0.197	0.776	0.7423935	0.9706199	1	987	tags=100%, l
PID_HIF1_TFPATHWAY	18	0.204	0.775	0.7258065	0.9694824	1	46	tags=11%, lis
GOCC_ENDOPLASMIC_RETICULUM	124	0.120	0.774	0.8576642	0.9688446	1	169	tags=16%, lis
GOBP_MEMBRANE_LIPID_METABOLIC_PROCESS	17	0.206	0.774	0.73069304	0.9675888	1	975	tags=100%, l
REACTOME_DEATH_RECEPTOR_SIGNALLING	17	0.212	0.772	0.7409524	0.9680611	1	968	tags=100%, l
REACTOME_TRANSPORT_OF_SMALL_MOLECULES	54	0.146	0.770	0.757085	0.967983	1	283	tags=30%, lis
GOCC_TRANSFERASE_COMPLEX_TRANSFERRING_PHOSPHORUS_CONTAINING_GROUPS	17	0.206	0.766	0.72962224	0.97237664	1	668	tags=82%, lis
GOBP_REGULATION_OF_PROTEASOMAL_UBIQUITIN_DEPENDENT_PROTEIN_CATABOLIC_PROCESS	16	0.212	0.763	0.74590164	0.9747644	1	183	tags=25%, lis
GOBP_REGULATION_OF_WNT_SIGNALING_PATHWAY	30	0.170	0.760	0.7798354	0.97735906	1	321	tags=40%, lis
GOBP_NEGATIVE_REGULATION_OF_CELL_DEATH	106	0.126	0.760	0.86907023	0.9756071	1	365	tags=38%, lis
GOMF_CHROMATIN_BINDING	60	0.142	0.758	0.81558937	0.97491056	1	373	tags=42%, lis
GOBP_SPHINGOLIPID_METABOLIC_PROCESS	15	0.217	0.758	0.7479839	0.9735266	1	166	tags=20%, lis
GOBP_NOTCH_SIGNALING_PATHWAY	16	0.205	0.757	0.7470817	0.9718354	1	136	tags=19%, lis
GOBP_IMPORT_INTO_NUCLEUS	15	0.208	0.756	0.75102043	0.97209275	1	311	tags=47%, lis
GOMF_TRANSPORTER_ACTIVITY	58	0.143	0.755	0.8109756	0.971149	1	186	tags=19%, lis
GOBP_CELL_CYCLE_PROCESS	113	0.122	0.754	0.8807157	0.9707163	1	350	tags=38%, lis
GOBP_CELLULAR_ION_HOMEOSTASIS	66	0.139	0.754	0.8582524	0.96851236	1	83	tags=11%, lis
REACTOME_RNA_POLYMERASE_II_TRANSCRIPTION	103	0.123	0.749	0.85945946	0.97304815	1	402	tags=44%, lis
GOBP_POSITIVE_REGULATION_OF_CYTOSKELETON_ORGANIZATION	22	0.187	0.748	0.75365347	0.972834	1	191	tags=23%, lis
GOCC_NUCLEAR_SPECK	51	0.146	0.747	0.8276553	0.97106725	1	390	tags=43%, lis
GOBP_HORMONE_MEDIATED_SIGNALING_PATHWAY	15	0.211	0.746	0.7749004	0.97092754	1	300	tags=40%, lis
GOMF_RNA_POLYMERASE_II_SPECIFIC_DNA_BINDING_TRANSCRIPTION_FACTOR_BINDING	36	0.159	0.744	0.7837838	0.9718709	1	335	tags=39%, lis
GOBP_CELLULAR_RESPONSE_TO_STARVATION	17	0.197	0.743	0.7805383	0.9704136	1	91	tags=12%, lis
GOBP_REGULATION_OF_UBIQUITIN_DEPENDENT_PROTEIN_CATABOLIC_PROCESS	18	0.197	0.743	0.7663934	0.96844614	1	333	tags=44%, lis
GOBP_POSITIVE_REGULATION_OF_ORGANELLE_ORGANIZATION	59	0.138	0.742	0.8255159	0.96806526	1	297	tags=32%, lis
GOMF_PROTEIN_HETERODIMERIZATION_ACTIVITY	16	0.204	0.741	0.7601713	0.9671504	1	280	tags=38%, lis
GOBP_REGULATION_OF_CELLULAR_RESPONSE_TO_STRESS	80	0.127	0.740	0.84440225	0.96558607	1	361	tags=38%, lis
GOBP_REGULATION_OF_SUPRAMOLECULAR_FIBER_ORGANIZATION	42	0.150	0.739	0.84456927	0.96478224	1	114	tags=14%, lis
GOCC_NUCLEAR_OUTER_MEMBRANE_ENDOPLASMIC_RETICULUM_MEMBRANE_NETWORK	84	0.128	0.735	0.84774435	0.96807706	1	169	tags=15%, lis
GOBP_CARBOHYDRATE_DERIVATIVE_BIOSYNTHETIC_PROCESS	45	0.147	0.735	0.8007737	0.9659795	1	195	tags=20%, lis
GOBP_NUCLEAR_TRANSCRIBED_MRNA_CATABOLIC_PROCESS	20	0.187	0.731	0.78294575	0.96886563	1	535	tags=70%, lis
GOBP_POSITIVE_REGULATION_OF_TRANSCRIPTION_BY_RNA_POLYMERASE_II	115	0.117	0.729	0.8844697	0.9693513	1	87	tags=9%, list-
GOBP_CELL_DIVISION	57	0.137	0.727	0.8469388	0.9698593	1	231	tags=26%, lis
GOBP_CYTOSOLIC_CALCIUM_ION_TRANSPORT	17	0.190	0.722	0.8067729	0.97412604	1	296	tags=35%, lis
GOBP_LEUKOCYTE_APOPTOTIC_PROCESS	20	0.184	0.722	0.8015873	0.9725328	1	171	tags=20%, lis
GOMF_TRANSCRIPTION_COACTIVATOR_ACTIVITY	33	0.157	0.719	0.8677686	0.97384703	1	394	tags=48%, lis
GOBP_REGULATION_OF_ACTIN_FILAMENT_BASED_PROCESS	48	0.142	0.714	0.84210527	0.9782398	1	241	tags=25%, lis
GOMF_TRANSCRIPTION_REGULATOR_ACTIVITY	134	0.111	0.714	0.9255121	0.97616297	1	177	tags=16%, lis
GOBP_SEXUAL_REPRODUCTION	38	0.153	0.713	0.83953035	0.9749434	1	224	tags=26%, lis
GOBP_NEGATIVE_REGULATION_OF_APOPTOTIC_SIGNALING_PATHWAY	26	0.166	0.710	0.822	0.97686845	1	41	tags=8%, list-
GOCC_CENTROSOME	44	0.144	0.709	0.8686274	0.97557473	1	231	tags=20%, lis
GOBP_NEGATIVE_REGULATION_OF_SUPRAMOLECULAR_FIBER_ORGANIZATION	17	0.188	0.697	0.851927	0.9879799	1	102	tags=18%, lis
GOBP_PROTEIN_METHYLATION	18	0.182	0.695	0.8522073	0.98855066	1	843	tags=94%, lis
GOMF_RIBONUCLEOTIDE_BINDING	128	0.107	0.685	0.95229006	0.9966267	1	120	tags=11%, lis
REACTOME_SIGNALING_BY_NOTCH	30	0.153	0.682	0.84631145	0.9978583	1	210	tags=23%, lis
GOBP_CELLULAR_MACROMOLECULE_LOCALIZATION	161	0.103	0.681	0.9458042	0.9970872	1	333	tags=32%, lis
GOBP_MEIOTIC_CELL_CYCLE	15	0.187	0.677	0.8340517	0.9989976	1	293	tags=40%, lis
GOBP_REGULATION_OF_RESPONSE_TO_ENDOPLASMIC_RETICULUM_STRESS	15	0.188	0.677	0.83439493	0.9977103	1	157	tags=20%, lis
GOBP_REGULATION_OF_MICROTUBULE_BASED_PROCESS	16	0.185	0.674	0.8580786	0.99819785	1	278	tags=31%, lis

GOBP_POSITIVE_REGULATION_OF_CELLULAR_PROTEIN_LOCALIZATION	33	0.147	0.672	0.8935743	0.9981778	1	164	tags=18%, lis
GOBP_REGULATION_OF_PROTEIN_DEPHOSPHORYLATION	22	0.164	0.669	0.8495238	0.99866426	1	349	tags=41%, lis
GOBP_POSITIVE_REGULATION_OF_BIOSYNTHETIC_PROCESS	182	0.099	0.665	0.9657658	1	1	87	tags=8%, list-
GOBP_INTEGRIN_MEDIATED_SIGNALING_PATHWAY	17	0.175	0.657	0.85714287	1	1	1012	tags=100%, l
GOBP_REGULATION_OF_PROTEIN_MODIFICATION_BY_SMALL_PROTEIN_CONJUGATION_OR_REMO	29	0.146	0.656	0.88258314	1	1	358	tags=41%, lis
GOBP_GLAND_DEVELOPMENT	41	0.137	0.656	0.88671875	1	1	55	tags=7%, list-
GOBP_METAL_ION_HOMEOSTASIS	62	0.119	0.655	0.89980733	1	1	83	tags=10%, lis
GOBP_POSITIVE_REGULATION_OF_PEPTIDYL_TYROSINE_PHOSPHORYLATION	24	0.157	0.654	0.872549	1	1	127	tags=13%, lis
REACTOME_CLATHRIN_MEDIATED_ENDOCYTOSIS	18	0.169	0.650	0.87656903	1	1	558	tags=67%, lis
GOBP_REGULATION_OF_ORGANELLE_ORGANIZATION	120	0.103	0.649	0.95173746	1	1	297	tags=28%, lis
GOBP_SUPRAMOLECULAR_FIBER_ORGANIZATION	71	0.115	0.647	0.93402064	1	1	164	tags=15%, lis
GOBP_POSITIVE_REGULATION_OF_ESTABLISHMENT_OF_PROTEIN_LOCALIZATION	43	0.130	0.644	0.908	1	1	67	tags=9%, list-
GOBP_NEGATIVE_REGULATION_OF_MRNA_METABOLIC_PROCESS	16	0.177	0.641	0.8924949	1	1	386	tags=56%, lis
GOBP_REGULATION_OF_PROTEIN_CONTAINING_COMPLEX_ASSEMBLY	43	0.129	0.640	0.9207921	1	1	102	tags=12%, lis
GOBP_ESTABLISHMENT_OF_PROTEIN_LOCALIZATION	170	0.097	0.638	0.981378	1	1	333	tags=31%, lis
GOBP_ORGANELLE_ASSEMBLY	52	0.119	0.633	0.9207207	1	1	307	tags=31%, lis
GOCC_MITOCHONDRION	103	0.104	0.630	0.9503817	1	1	92	tags=9%, list-
GOMF_PROTEIN_DOMAIN_SPECIFIC_BINDING	79	0.110	0.629	0.9617706	1	1	231	tags=20%, lis
GOCC_SUPRAMOLECULAR_POLYMER	68	0.112	0.628	0.9411765	1	1	231	tags=21%, lis
GOBP_MYELOID_CELL_HOMEOSTASIS	23	0.155	0.625	0.90729785	1	1	141	tags=17%, lis
GOBP_POSITIVE_REGULATION_OF_NUCLEOBASE_CONTAINING_COMPOUND_METABOLIC_PROCES	184	0.092	0.621	0.98717946	1	1	335	tags=31%, lis
GOCC_POSTSYNAPSE	40	0.128	0.620	0.9315069	1	1	233	tags=25%, lis
GOBP_TRANSITION_METAL_ION_HOMEOSTASIS	15	0.173	0.617	0.9005736	1	1	83	tags=13%, lis
GOBP_TRANSITION_METAL_ION_TRANSPORT	15	0.170	0.611	0.9042969	1	1	83	tags=13%, lis
GOCC_MICROTUBULE_ORGANIZING_CENTER	52	0.116	0.610	0.91769546	1	1	231	tags=19%, lis
GOBP_REGULATION_OF_AUTOPHAGY	28	0.142	0.608	0.9166667	1	1	60	tags=7%, list-
GOCC_SPINDLE	33	0.135	0.604	0.914405	1	1	307	tags=33%, lis
GOBP_PROTEIN_LOCALIZATION_TO_PLASMA_MEMBRANE	26	0.142	0.604	0.9124236	1	1	114	tags=12%, lis
GOBP_CELL_SURFACE_RECEPTOR_SIGNALING_PATHWAY_INVOLVED_IN_CELL_CELL_SIGNALING	47	0.118	0.602	0.935743	0.99960023	1	321	tags=34%, lis
GOCC_NEURON_PROJECTION	72	0.107	0.599	0.9628099	0.99966604	1	102	tags=10%, lis
GOCC_CELL_CORTEX	29	0.137	0.596	0.92870903	0.999872	1	358	tags=41%, lis
REACTOME_APOPTOSIS	29	0.135	0.591	0.9221311	1	1	402	tags=48%, lis
GOBP_REGULATION_OF_ACTIN_FILAMENT_LENGTH	23	0.144	0.591	0.9191919	0.9992469	1	275	tags=30%, lis
GOBP_PROTEIN_LOCALIZATION_TO_CELL_PERIPHERY	27	0.134	0.588	0.93801653	0.99885374	1	114	tags=11%, lis
GOBP_POSITIVE_REGULATION_OF_TRANSMEMBRANE_TRANSPORT	18	0.154	0.585	0.9298597	0.99873334	1	365	tags=50%, lis
GOBP_CELL_CELL_SIGNALING_BY_WNT	44	0.118	0.583	0.9472656	0.99771404	1	321	tags=34%, lis
GOBP_POSITIVE_REGULATION_OF_CHROMOSOME_ORGANIZATION	18	0.152	0.583	0.9539749	0.9957782	1	372	tags=50%, lis
GOCC_OUTER_MEMBRANE	16	0.156	0.559	0.9287169	1	1	92	tags=13%, lis
GOMF_DNA_BINDING_TRANSCRIPTION_FACTOR_BINDING	46	0.112	0.554	0.95728153	1	1	256	tags=24%, lis
GOBP_PROTEIN_TARGETING	37	0.113	0.537	0.9745763	1	1	365	tags=38%, lis
GOBP_CALCIUM_ION_TRANSMEMBRANE_TRANSPORT	21	0.135	0.521	0.96862745	1	1	296	tags=33%, lis
GOCC_AXON	38	0.106	0.519	0.9714286	1	1	102	tags=11%, lis
GOBP_MAINTENANCE_OF_CELL_NUMBER	16	0.144	0.517	0.94401544	1	1	619	tags=75%, lis
GOCC_PLASMA_MEMBRANE_RAFT	16	0.139	0.517	0.9657258	1	1	28	tags=6%, list-
REACTOME_DISEASES_OF_SIGNAL_TRANSDUCTION_BY_GROWTH_FACTOR_RECEPTORS_AND_SEC	57	0.097	0.510	0.98043054	1	1	1034	tags=96%, lis
GOBP_RESPIRATORY_SYSTEM_DEVELOPMENT	18	0.133	0.497	0.97849464	1	1	230	tags=28%, lis
REACTOME_RHO_GTPASE_CYCLE	63	0.091	0.497	0.98778003	1	1	280	tags=24%, lis
GOBP_MICROTUBULE_CYTOSKELETON_ORGANIZATION	32	0.110	0.496	0.9875	1	1	59	tags=6%, list-
REACTOME_RAC3_GTPASE_CYCLE	17	0.130	0.494	0.9771784	1	1	280	tags=29%, lis
GOBP_REGULATION_OF_CELL_SIZE	17	0.134	0.487	0.97808766	1	1	1062	tags=100%, l
REACTOME_SIGNALING_BY_RHO_GTPASES_MIRO_GTPASES_AND_RHOBTB3	81	0.084	0.487	0.99416345	1	1	280	tags=23%, lis
GOBP_MICROTUBULE_BASED_PROCESS	45	0.097	0.483	0.98085105	1	1	218	tags=18%, lis
GOCC_POLYMERIC_CYTOSKELETAL_FIBER	59	0.091	0.483	0.99182004	1	1	231	tags=20%, lis
REACTOME_RHO_GTPASE_EFFECTORS	34	0.102	0.465	0.97884613	1	1	94	tags=9%, list-
GOCC_MICROTUBULE_CYTOSKELETON	97	0.077	0.464	0.99638987	1	1	293	tags=25%, lis
GOBP_PROCESS_UTILIZING_AUTOPHAGIC_MECHANISM	44	0.094	0.460	0.9941748	1	1	126	tags=9%, list-
GOBP_REGULATION_OF_PHOSPHATASE_ACTIVITY	19	0.117	0.459	0.9920792	1	1	412	tags=47%, lis
GOCC_SYNAPSE	83	0.079	0.455	0.9941748	1	1	234	tags=22%, lis
REACTOME_RAC2_GTPASE_CYCLE	15	0.127	0.450	0.98550725	1	1	137	tags=13%, lis
GOBP_CELLULAR_MONOVALENT_INORGANIC_CATION_HOMEOSTASIS	15	0.127	0.449	0.9896907	0.9996164	1	39	tags=7%, list-
GOBP_MONOVALENT_INORGANIC_CATION_HOMEOSTASIS	16	0.121	0.447	0.9846154	0.9979366	1	1077	tags=100%, l
GOCC_EARLY_ENDOSOME_MEMBRANE	16	0.121	0.443	0.9935484	0.99671036	1	933	tags=94%, lis
GOBP_PROTEIN_LOCALIZATION_TO_MEMBRANE	51	0.080	0.413	0.9959267	0.99820095	1	297	tags=25%, lis
GOCC_SITE_OF_POLARIZED_GROWTH	16	0.090	0.325	1	0.9998265	1	979	tags=94%, lis
